# Molecular and metabolomic changes in the proximal colon of pigs infected with *Trichuris suis*

**DOI:** 10.1038/s41598-020-69462-5

**Published:** 2020-07-30

**Authors:** Harry D. Dawson, Celine Chen, Robert W. Li, Lauren Nicole Bell, Terez Shea-Donohue, Helene Kringel, Ethiopia Beshah, Dolores E. Hill, Joseph F. Urban

**Affiliations:** 1 United States Department of Agriculture, Agricultural Research Service, Beltsville Human Nutrition Research Center, Diet, Genomics, and Immunology Laboratory, Beltsville, USA; 2 Beltsville Agricultural Research Center, Animal Parasitic Diseases Laboratory, Beltsville, MD USA; 3 Metabolon, Inc., Morrisville, NC USA; 4 University of Maryland School of Medicine, Baltimore, MD USA; 5 Department of Veterinary Disease Biology, Faculty of Health and Medical Sciences, University of Copenhagen, Copenhagen, Denmark

**Keywords:** Parasitic infection, Mucosal immunology

## Abstract

The pig whipworm *Trichuris suis* is important in swine production because of its negative effects on pig performance and, notably, to some humans with inflammatory bowel disease as a therapeutic agent that modulates inflammation. The proximal colon of *T. suis*-infected pigs exhibited general inflammation around day 21 after inoculation with infective eggs that is transcriptionally characterized by markers of type-2 immune activation, inflammation, cellular infiltration, tissue repair enzymes, pathways of oxidative stress, and altered intestinal barrier function. Prominent gene pathways involved the Th2-response, de novo cholesterol synthesis, fructose and glucose metabolism, basic amino acid metabolism, and bile acid transport. Upstream regulatory factor analysis implicated the bile acid/farnesoid X receptor in some of these processes. Metabolic analysis indicated changes in fatty acids, antioxidant capacity, biochemicals related to methylation, protein glycosylation, extracellular matrix structure, sugars, Krebs cycle intermediates, microbe-derived metabolites and altered metabolite transport. Close to 1,200 differentially expressed genes were modulated in the proximal colon of pigs with a persistent adult worm infection that was nearly 90% lower in pigs that had expelled worms. The results support a model to test diets that favorably alter the microbiome and improve host intestinal health in both pigs and humans exposed to *Trichuris*.

## Introduction

*Trichuris suis* is a pig gastrointestinal nematode that parasitizes the cecum and proximal colon after oral ingestion of infective eggs^1^. Fertilized eggs released with the feces of infected pigs undergo embryogenesis and develop into first stage larvae (L_1_) within an environmentally stable and long-lived infective egg. Larvae from the fecal–oral transmission of infective eggs emerge in the ileum of pigs and then the parasitic larvae advance through four molts (L_2_, L_3_, and L_4_) and develop into the fecund adult stage (L_5_) over a period of 40–45 day in the cecum and proximal colon^2^. *Trichuris suis* is morphologically and genetically similar to the human whipworm *T. trichiura*^3,4^ but generally produces an abbreviated infection when inoculated into humans^3^. This has proven advantageous because therapeutic exposure of some humans to *T. suis* eggs showed beneficial effects on Inflammatory bowel Disease (IBD) without the production of potentially infectious eggs^5,6^.

Pigs acquire protective immunity from experimental inoculation with eggs^7–9^ and express an age-related resistance to natural infection^10^ similar to that seen in humans^11^. Increased parasite-specific antibody^12^ and pathology at the site of infection in the cecum and proximal colon^13,14^ is often accompanied by secondary bacterial infection of intestinal tissue^15^. In addition, the intestinal microbiome and metabolome are altered in the pig proximal colon by *T. suis*^16^, and these changes persist for a time after natural expulsion of adult worms^17^. Worm-induced changes in the host intestinal microbiome also inhibit the hatching of infective whipworm eggs from secondary infections that appears to regulate the dynamics of worm accumulation in the host^18^.

Infection with parasitic gastrointestinal nematodes polarize host immunity towards a Th2 response, which contributes to worm expulsion^19^ and also tends to reduce Th1- and Th17-mediated responses, increase production of IL-10 and TGF-β by regulatory T (T_reg_) cells, and activate regulatory dendritic cells and alternatively-activated macrophages (AAMФs); which may be partially responsible for modulating parasite-induced host local and bystander inflammation intensity^20^. Mice infected with *T. muris* express resistance or susceptibility to infection through an interplay of Th1 and Th2 associated cytokines and their effects on epithelial cell turnover at the site where worms reside^21–23^. These mouse models are instructive as the Th2 response is protective against whipworm in mice and similarly associated with resistance in pigs^17,24^ but extrapolation of many features of whipworm infection in mice to pigs and humans is limited by differences in comparative immunology and physiology^25–28^ . Thus, characterization of *T. suis* infection in the natural host can better inform approaches to integrated control procedures to improve pig health and production qualities, and can more closely represent features of the natural infection in humans and when infection is applied therapeutically.

We evaluated the transcriptome of the proximal colon of *T. suis* infected pigs at two time points in the infection, one early during third-stage larval (L3) development at 21 days after inoculation (DAI) and a second with fecund adult worms at 52 DAI to identify differentially expressed genes (DEGs) in pigs with a persistent infection versus those that had expelled the adult worms. Information on the later time point was supported by real-time PCR analysis of both local intestinal tissue and draining lymph nodes. In addition, a metabolomic analysis of the luminal contents and tissue of the proximal colon was used to characterize host and microbial metabolites that are altered by infection as well as physiological changes in epithelial barrier resistance and basal secretion. A comprehensive evaluation of the changes induced by larval infection and following the expulsion of adult worms suggested that alteration of tissue metabolism through diet may improve the health of the intestine as it provides metabolites to enhance host immune function, anti-oxidant capacity and tissue repair, and directly and indirectly modulate bacterial populations that effect epithelial cell vigor and improved barrier function.

## Results

### Recovery of *T. suis* larvae and adults from outbred pigs showed resistant and susceptible phenotypes

**Figure 1:**
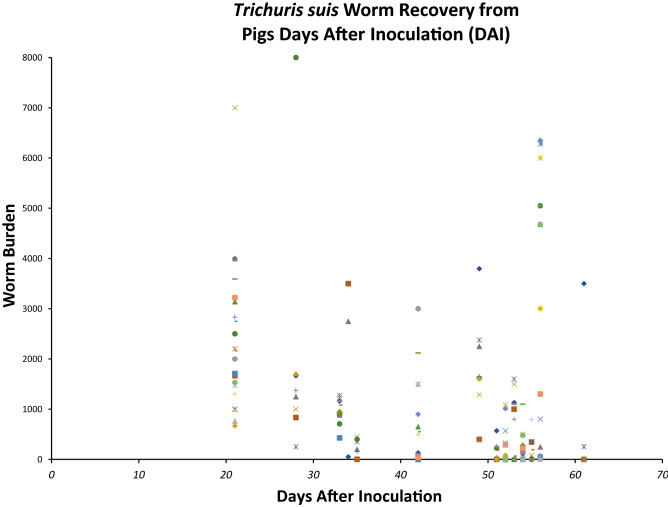
*Trichuris suis* worm recovery from pigs days after inoculation. The data points indicate recovery of *T. suis* larval and adult stages from individual pigs at various days after inoculation (DAI) with *T. suis* infective eggs. The unique symbols indicate recovery from individual pigs.

Larval and adult stages of *T. suis* were recovered from the cecum and proximal colon of pigs at various times after inoculation with infective eggs (Fig. 1). The number of infected pigs with zero worms recovered in any particular period after experimental egg inoculation began to increase at 35–42 DAI and consistently increased at 52 DAI and later. This feature was apparent regardless of the infective egg preparation that was used for inoculation, the sex of the pigs, and the source of pigs produced locally at the Beltsville Agricultural Research Center (BARC) or purchased from an outside supplier (Oakhill) (Supplemental Table S1). The intensity of the localized tissue hemorrhagic response and mucus production in the proximal colon varied but was observed in pigs as early as 21 DAI and later, and generally appeared as normal^15^ in infected pigs that had cleared the worms (Supplemental Table S1).

### Physiological changes in epithelial cell resistance and basal secretion in the proximal colon

**Figure 2:**
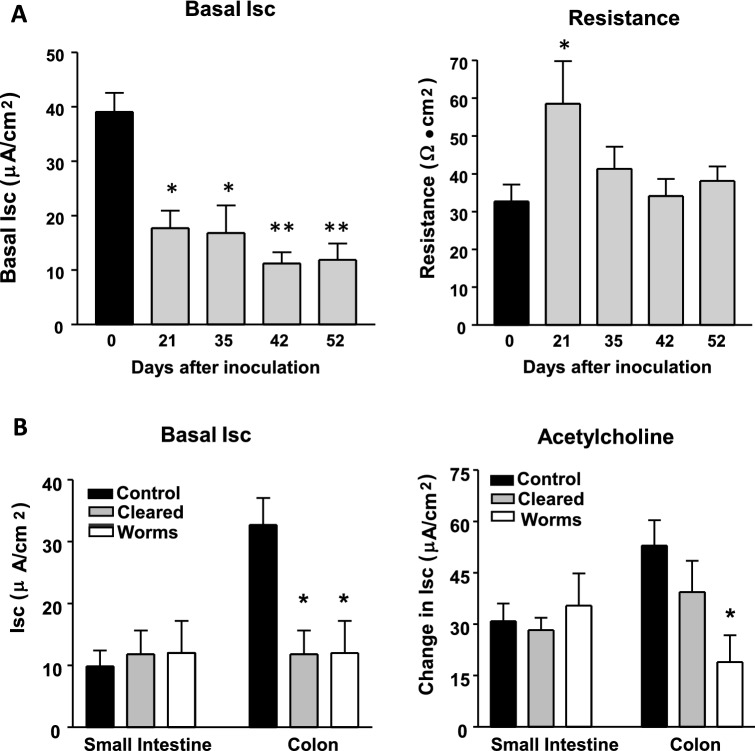
Changes in net ion flux and tissue permeability in the proximal colon post inoculation with *Trichuris suis*. (**A**) Basal flow of ions across the mucosa of the proximal colon of uninfected pigs (day 0) and pigs infected for 21, 35, 42, and 52 days after inoculation with infective eggs;. six pigs were analyzed at each time interval after an inoculation with the same batch of *T. suis* eggs at a fixed time point. (**B**) Altered secretion in the colon (proximal tissue) of uninfected pigs (Control) and those with resident worms (worms) versus those that were worm-free (cleared) at 52/53 days after inoculation with *T. suis* eggs, and in response to the secretogogue acetylcholine***.*** Five pigs were analyzed at each time point (Supplemental Table S1).

Parasitic nematode infection in the small intestine of both mice and pigs is characterized by increased secretion locally in response to potent secretagogues like acetylcholine (Ach) and reduced transport of glucose across the mucosa that are associated with worm expulsion^29–31^. These physiological changes have not been critically evaluated in the colon of pigs infected with *T. suis* but should reflect local functional changes in gene expression^17,24^. Thus, changes in net ion flux and tissue resistance or permeability were evaluated in the proximal colon of pigs inoculated with infective *T. suis* eggs. There was a significant reduction in basal secretion detected in pigs from 21 through 52 DAI and an increased resistance at 21 DAI that quickly returned to normal (Fig. 2A). Similar changes were not observed in tissue removed from the small intestine (jejunum) of infected pigs; although tissue resistance was transiently increased at day 21 DAI with *T. suis* eggs (data not shown). An analysis was then focused on the proximal colon of infected pigs that had cleared worms (worm-free) at 52 DAI versus those with a persistent adult worm infection. The decrease in basal secretion in the proximal colon was not different in the presence or absence of worms compared to tissue from uninfected pigs at 52 DAI, but secretion induced by acetylcholine (Ach) was significantly lower in pigs with worms than in pigs that were worm-free or in tissue from uninfected control pigs (Fig. 2B).

### Cluster of type-2 immune-related gene expression activated at the site of *T. suis* infection

**Figure 3:**
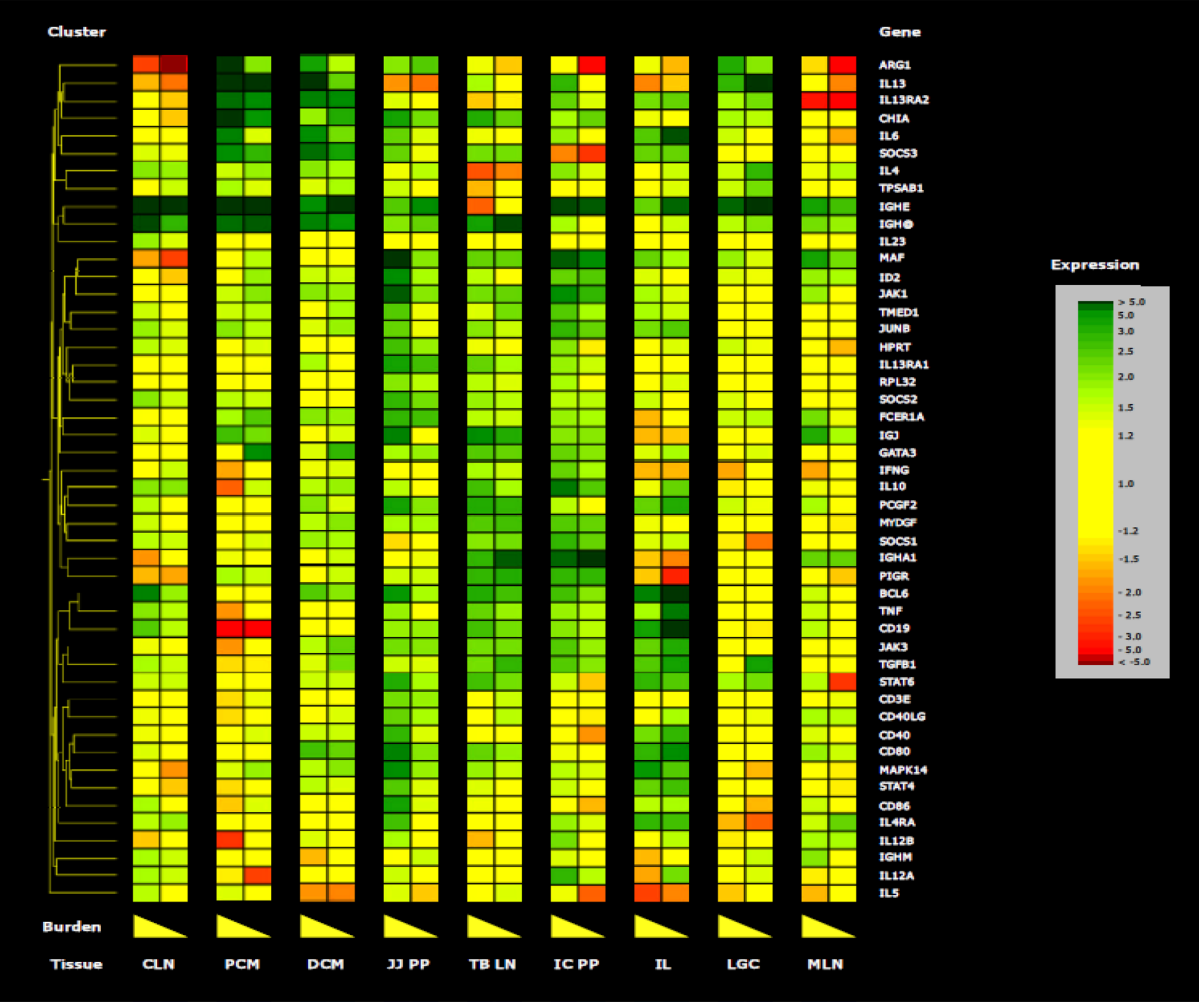
Gene expression in pigs with or without worms at day 53 after inoculation with *Trichuris suis* eggs. Tissue sections from the small intestine ileum (IL), large intestine proximal colon (PCM) and distal colon (DCM), mesenteric (MLN), colonic (CLN), and tracheal-bronchial lymph nodes (TBLN), jejunum Peyer’s patch (JJ PP), ileal-cecal Peyer’s patch (IC PP), and lympho-glandular complexes (LGC) were collected at necropsy. Quantitative real time PCR was performed on cDNA synthesized from each sample using 10 μg of total RNA. Up-regulation is shown in green, down-regulation in red, and low lever modulation in yellow. The high end of the right triangle symbol represents high worm burden and the low end represents low worm burden. Data is derived from three pigs/group (Supplemental Table S1).

Protective immunity to *T. muris* in the mouse is dependent on expression of a type-2 response that include a cluster of genes that activate Th2-mediated pathways dependent on STAT6. A similar association has been indicated in pigs infected with *T. suis*^17,24^. A composite analysis of intestinal tissues and draining lymph nodes localized at the site of infection in pigs where adult worms persist versus those that expelled worms at 52 DAI was evaluated by RT-PCR (Fig. 3 and Supplemental Table S2). A cluster of genes responsible for activation of Th2 immunity (IL13, IL4) and expression of pathways related to worm expulsion (ARG1, IL13RA, CHIA, SOCS3, FCER1A) were significantly up-regulated in the proximal colon (PCM) and expressed to a greater intensity when worms persisted in the tissue compared to pigs that had expelled adult *T. suis*. As anticipated, genes related to Th1-related pathways such as IFNG, TNF and IL12A were down-regulated, especially in pigs where worms persisted. This response was largely reflected in the distal colon tissue (DCM) that is near the mucosal site of infection in the large intestine. Expression of the genes for the constant regions of immunoglobulin IgE and IgA were more generally activated across mucosal and gut-associated lymphoid tissues except for a reduction in the more distal tracheal-bronchial lymph node (TBLN). Notably, these genes were largely down-regulated in associated lymphoid tissues like the mesenteric (MLN) and colonic (CLN) lymph nodes.

### Measurement of the proximal colon transcriptome during *T. suis* infection at day 21

**Figure 4:**
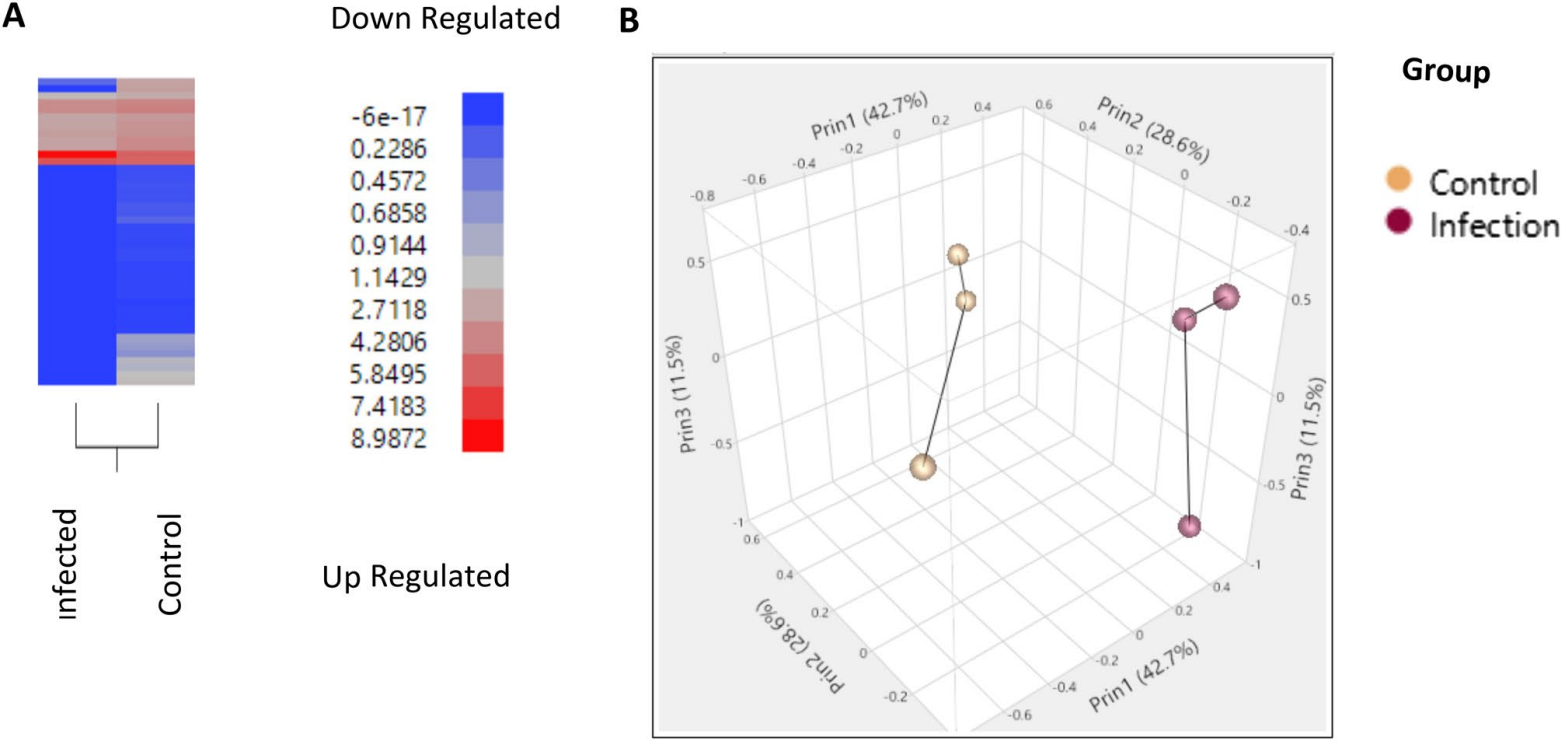
Principle component analysis of pigs infected with *T. suis* for 21 days versus uninfected pigs. Weighted average proportion across principal components as a hierarchical cluster analysis of significantly differentially expressed genes (**A**) and 3D plot (**B**) of individual samples from control uninfected and *T. suis*-infected pigs. Analysis was done on three *T. suis*-infected pigs (Supplemental Table S1) and three un-infected control pigs. Data on *T. suis*-induced changes in the intestinal microbiome has been reported previously for these pigs^16^.

Two critical time points in the infection were selected to evaluate the local tissue response to *T. suis* worms by RNAseq. Developing larvae activate local changes in physiology and tissue pathology at 21 DAI, and tissue isolated from pigs at 52 DAI can be clearly distinguished between those pigs with and without a persistent adult worm infection and associated changes in the local production of mucus and tissue inflammation. Principal component analysis (PCA) showed the separation of the naïve uninfected pigs compared to those infected with *T. suis* for 21 DAI (Fig. 4A and B). These same pigs were used as a source of intestinal lumen contents for analysis of *T. suis*-induced changes in the intestinal microbiome in previous studies^16,17^. There were 210 DEG that were significantly down-regulated and 210 that were significantly up-regulated greater than 1.7 fold in the proximal colon of pigs infected with *T. suis* at 21 DAI. The top 20 up and down regulated genes are shown in Table 1. The full set of genes are shown in Supplemental Table S3. There was a prominent group of up-regulated genes related to the type 2 immune response including Interleukin 4 induced 1 (IL4I1)^32,33^ (438 fold), arginase 1 ARG1^34^ (30.1 fold), the cytokine, IL11^35^ (21.6 fold) and serpin family B member 2 (SERPINB2)^36^ (16.2 fold) (Table 1). Other molecules, previously described as being associated with the response to helminth infection, were induced including trefoil factor 1 (TFF1) (25.9 fold)^37^, TFF2 (37.1 fold)^38^, regenerating islet-derived family, member 4 (REG4) (11.1 fold)^39^, the regulatory cytokine, amphiregulin (AREG) (2.2 fold)^40^, the goblet cell differentiation marker^41,42^ chloride channel accessory 1 (CLCA1, 3.5 fold) and mucin 2 (MUC2, 6.3 fold)^43^.

**Table 1:** Top 20 down- and up-regulated differentially expressed genes at 21 days after inoculation.

Feature ID	Fold change	FDR corrected *p*-value	Feature ID	Fold change	FDR corrected *p*-value
PLA2G2A	− 1633.2	1.33E−05	S100A12	12.8	4.09E−05
OLIG2	− 143.0	1.09E−03	PDZK1IP1*	13.6	1.24E−05
ZFP42	− 89.1	3.67E−11	AKR1C1	13.8	3.93E−07
DNAH14	− 60.4	1.56E−02	GABRP	15.7	1.59E−03
COL22A1	− 56.8	1.34E−02	A2ML1	16.0	2.14E−03
ADAM1A	− 52.9	1.87E−02	SERPINB2	16.2	4.90E−32
ASTL	− 49.0	2.75E−02	SLC6A2	18.7	1.53E−02
FABP3L2*	− 44.6	1.84E−04	PSAT1	20.6	4.69E−11
ABCC4L3	− 23.7	8.32E−19	IL11	21.6	6.84E−03
LIPM	− 19.0	2.62E−02	TFF1	25.9	2.62E−06
ZSCAN9	− 18.9	4.68E−02	MMP13	27.5	9.71E−29
CD40	− 17.7	4.10E−02	ARG1	30.1	2.28E−07
MIR1279	− 16.9	4.50E−05	IL36B	30.8	1.02E−02
SLC22A10L3*	− 14.0	1.75E−03	ANXA8	34.8	6.97E−40
HOXC9	− 12.3	5.27E−03	TFF2	37.1	6.93E−10
ABCC4L4	− 12.2	7.35E−07	MMP7	53.1	4.09E−05
FCER2	− 12.0	1.34E−02	IFITM1L3*	63.2	1.59E−02
AGTR2	− 11.5	3.94E−03	HOXB13	132.7	1.59E−02
ALDOB	− 10.6	8.40E−19	SLC7A3L11*	188.2	1.59E−03
CCL3L2	− 10.6	5.90E−03	IL4I1L	437.7	1.59E−14

Data from a comparison of three *Trichuris suis*-infected and three uninfected control pigs. Asterisk denotes pig (or artiodactyl) specific para-logs with no corresponding human genes.

Several S100 proteins were induced including the eosinophil chemoattractant, S100 calcium binding protein A2 (S100A2, 10.0 fold)^44^, the mast cell activator, S100A12 (12.8 fold)^45^ and the bioindicators of neutrophilic intestinal inflammation^46^, S100A8 (8.2 fold) and S100A9 (9.8 fold). Several genes for enzymes related to extra-cellular matrix modification and tissue repair^47^ were induced including matrix metalloproteinase 1 (MMP1, 7.3 fold), MMP3 (4.9 fold), MMP7 (53.1 fold), MMP9 (9.3 fold), MMP12 (9.7 fold), MMP13 (27.5 fold), tissue inhibitor of metalloproteinase 1 (TIMP1, 3.9 fold) and plasminogen activator; urokinase receptor (PLAUR, 4.8 fold). Several genes in the IL1/IL1R axis including the decoy receptor/interleukin 1 receptor, type II (IL1R2, 6.1 fold), IL1A (2.4 fold), IL36A (7.2 fold) and IL36B (30.8 fold) were upregulated as were the neurotransmitter receptors, 5-hydroxytryptamine (serotonin) receptor 1B (HTR1B, 11.8 fold) and gamma-aminobutyric acid (GABA) A receptor, pi (GABRP, 15.7 fold).

Several markers of colonic inflammation were down-regulated including the cytokine TNFSF11^48^ (− 11.1 fold), the positive acute phase reactant, PLA2G2A^49^ (− 1633 fold), the type 2 interferon regulated gene IDO1^50^ (− 3.8) and the type 2 interferon regulated chemokine, CXCL9^51^ (− 6.1 fold). Other downregulated chemokines genes included CCL2 (− 2.0 fold), CCL3L1 (− 5.6 fold), CCL3L2 (− 10.6 fold), CCL8 (− 2.8 fold), CXCL12 (− 2.3 fold), CXCL13 (− 5.8 fold), PF4 (− 3.2 fold) and the gut-tropic chemokine receptor CCR9^52^ (− 5.8 fold). Several metabolic-related genes that were down-regulated include; the chenodeoxycholic acid/bile acid/farnesoid receptor^53^, nuclear receptor subfamily 1, group H, member 4 (NR1H4, − 3.7 fold), the glycolysis-associated enzyme, aldolase B (ALDOB, − 10.6 fold), lipase M (LIPM, − 19.0 fold) and the cholesterol transporter, ATP binding cassette subfamily A member 9 (ABCA9, − 5.5 fold).

### Measurement of the local tissue transcriptome during *T. suis* infection at day 52

**Figure 5:**
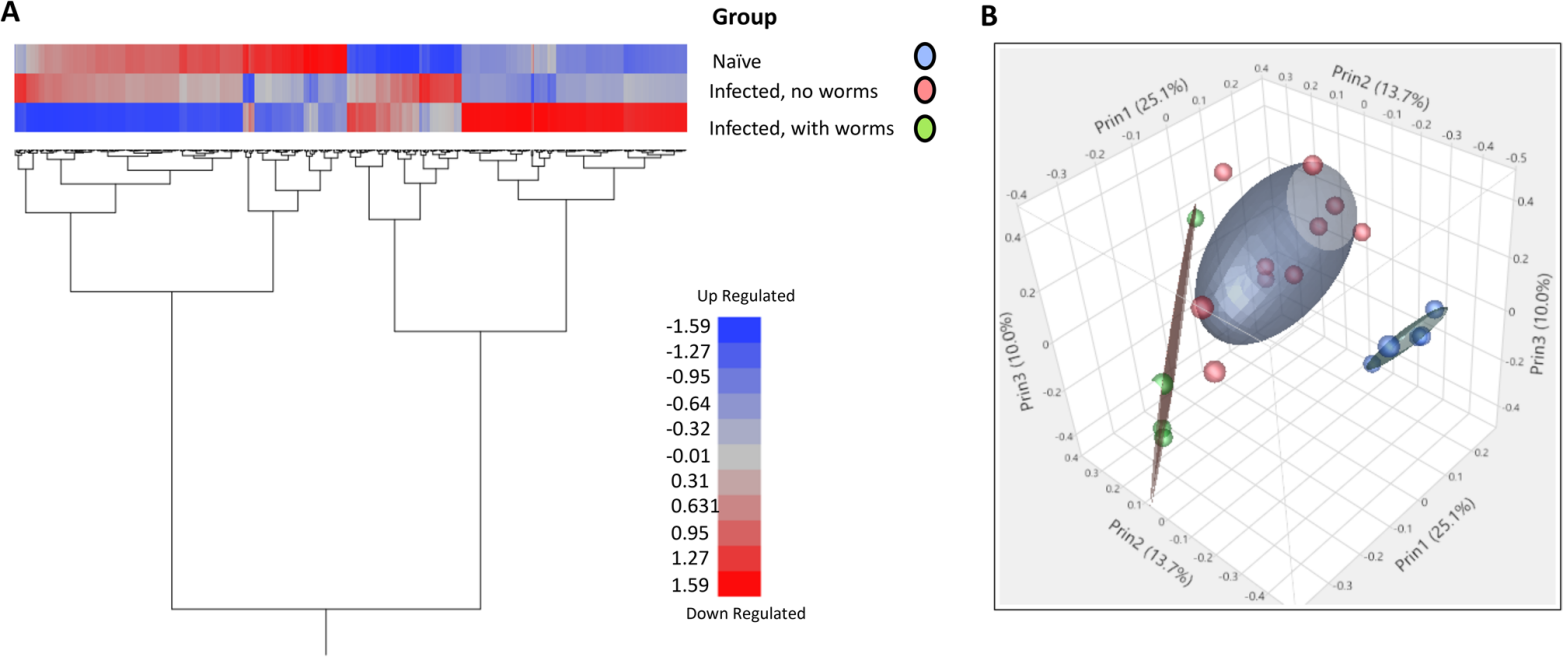
Principal component analysis of proximal colon from uninfected and *T. suis*-infected pigs with and without worms at day 52 after inoculation. (**A**) Principle component analysis reveals clear separation of the three populations: (1) infected, no worms present (colored red) and (2) infected, worms present (colored green) and (3) naïve (colored blue). (**B**) Hierarchical cluster analysis of significantly differentially expressed genes induced by *T. suis* : (a) naive (b) infected, no worms present and (c) infected, worms present. Gene expression values were clustered based on their log2 based expression values using JMP Genomics 9.0. Negative numbers (colored blue) indicate less relative gene expression, and positive numbers (colored red) indicate greater relative gene expression. Analysis was done on three infected pigs with worms, five infected pigs without worms, and three un-infected control pigs (Supplemental Table S1). Data on *T. suis*-induced changes in the intestinal microbiome has been reported previously for these pigs^17^.

The proximal colon tissue was analyzed by RNAseq at 52 DAI from pigs with a persistent worm infection and those that had cleared the infection. The PCA for these two infected groups and the uninfected control group is shown in Fig. 5b and heat map of expressed genes (Fig. 5a). A comparison of genes that were significantly modulated > 1.5 or <  − 1.5 fold for infected worm-free pigs versus uninfected controls showed 86 up-regulated and 69 down-regulated genes (Supplemental Table S4); the top 20 up and down-regulated genes are found in Table 2. Infected pigs with worms showed 679 up-regulated and 542 down-regulated genes versus uninfected controls (Supplemental Table S5); the top 20 up and down-regulated genes are found in Table 3. A comparison of infected worm-free pigs versus infected pigs with worms showed 392 up-regulated and 245 down-regulated (Supplemental Table S6); the top 20 up- and down-regulated genes are found in Table 4. It is clear from this evaluation that the presence of worms in infected pigs drives a dramatic modulation of local gene expression that is reduced by about 86% in infected worm-free pigs where the tissue appears to re-establish normalcy. A discussion of these changes based upon functional classification of these DEGs follows. Functional determination of these DEGs were derived from the literature and are contained in the Porcine Translational Database^27^ (https://tinyurl.com/hxxq3ur). Functional annotations are noted, as PubMed IDs in parenthesis in the Tables, or cited as references in the text.

**Table 2:** Top 20 down- and up-regulated differentially expressed genes at 52 days after inoculation in pigs with no worms versus control.

Feature ID	Fold change	FDR corrected *p*-value	Feature ID	Fold change	FDR corrected *p*-value
AQP8	− 171.3	6.09E−10	RHCG	17.1	7.24E−05
SLC30A10	− 156.1	1.98E−25	AQP5	17.3	2.57E−09
BTNL2	− 154.1	2.31E−03	WNT7A	17.9	3.75E−02
INMT	− 124.7	1.85E−03	S100A12	17.9	4.03E−06
SLC10A2	− 95.9	2.09E−02	AVPR1B	19.1	3.40E−08
BTN1A1	− 67.0	5.00E−03	WT1	19.2	8.42E−06
ALDOB	− 40.7	8.53E−05	TRIM6	19.9	1.29E−04
ADH4	− 34.4	4.42E−02	ARG1	20.0	2.86E−05
FLNB	− 27.9	4.63E−11	EPYC	20.1	1.38E−06
NXPE2	− 22.2	1.97E−07	SCGB2A1	21.6	1.47E−06
SLC26A3	− 22.0	1.50E−11	RMRP	22.2	1.57E−06
AICDA	− 20.4	1.87E−03	COMP	22.4	4.09E−09
TNN	− 20.0	1.38E−03	PCSK9	22.5	3.18E−09
SCGB3A1	− 18.2	2.02E−02	TFF2	30.1	7.52E−05
GUCA2B	− 17.2	2.26E−16	CAPNS2	30.8	2.00E−03
IL13	− 16.9	1.26E−03	CLEC18A	39.3	3.65E−06
MOXD2P	− 15.1	2.86E−03	PADI1	51.1	3.15E−04
DNASE1L3	− 14.9	1.85E−07	IGKJ2	84.4	1.50E−05
TRPV6	− 14.5	2.89E−03	GATA5	87.1	1.19E−05
ALPI	− 14.5	1.96E−04	TFF1	124.6	4.52E−10

Data from a comparison of five *Trichuris suis*-infected and three uninfected control pigs.

**Table 3:** Top 20 down- and up-regulated differentially expressed genes at 52 days after inoculation in pigs with worms versus control.

Feature ID	Fold change	FDR corrected *p*-value	Feature ID	Fold change	FDR corrected *p*-value
SLC10A2	− 1,285.8	8.30E−08	S100A12	38.4	4.48E−04
ALPI	− 159.7	2.09E−10	SDSL	39.6	1.38E−02
EVX1	− 38.1	2.47E−02	A3GALT2	40.6	5.54E−03
ASTL	− 37.2	2.34E−02	NTRK1	42.0	2.62E−07
CLCA4	− 27.9	1.34E−07	S100A9	44.8	2.59E−04
ALDOB	− 27.7	2.35E−07	SPINK6	46.2	5.38E−03
GSTA2	− 26.6	8.94E−06	LTF	52.9	5.95E−03
CYP2B22	− 26.4	2.38E−06	TCN1	54.1	1.15E−04
BTNL2	− 21.7	9.02E−03	PADI3	55.7	1.75E−02
HMGCS2	− 1.2	0.00E + 00	SERPINA3-2	60.8	2.96E−06
NXPE2	− 20.1	2.40E−06	TNN	61.3	4.18E−05
NR1H4	− 17.1	2.31E−11	AVPR1B	62.6	5.42E−03
PCK1	− 16.9	1.62E−07	GNLY	68.8	2.42E−09
FRMD1	− 15.5	9.59E−05	MMP12	101.0	2.26E−14
OASL	− 14.4	5.68E−03	SCGB2A2	105.1	3.14E−07
SLC14A1	− 13.9	1.46E−02	ARG1	122.6	4.56E−05
FOXO6	− 13.7	8.20E−06	IL4I1L	161.2	3.98E−05
AQP8	− 13.7	2.47E−03	REG3A	275.6	2.48E−03
SLC26A3	− 12.7	8.30E−08	PADI1	296.4	3.51E−03
NCR2	− 12.1	1.81E−05	TFF1	705.1	2.48E−05

Data from a comparison of three *Trichuris suis*-infected and three uninfected control pigs.

**Table 4:** Top 20 down- and up-regulated diffentially expressed genes at 52 days after inoculation in pigs with no worms versus pigs with worms.

Gene	Fold change	FDR corrected *p*-value	Gene	Fold change	FDR corrected *p*-value
SLC10A2	− 297.1	1.18E−04	MMP8	11.4	3.47E−03
ALPI	− 32.9	4.94E−06	MMP13	11.4	2.65E−07
ALDOB	− 21.0	1.66E−06	TFF2	12.2	3.25E−02
BTNL2	− 19.6	1.37E−02	SLC6A2	13.1	2.14E−02
AQP8	− 15.1	2.08E−03	S100A12	13.4	7.36E−04
NXPE2	− 13.8	2.92E−05	PRG4	13.5	3.43E−04
THAP12	− 13.4	2.27E−02	MMP12	13.9	7.63E−10
CLCA4	− 13.2	2.57E−05	C1QL2	15.4	1.39E−04
HMGCS2	− 12.8	0.00E + 00	SERPINA3-2	16.2	1.05E−06
OASL	− 12.1	1.20E−02	ARG1	16.6	8.41E−04
SLC26A3	− 12.0	9.52E−08	SCGB2A2	17.9	4.89E−06
FRMD1	− 9.6	1.51E−03	POU5F1	19.0	3.78E−02
FOXO6	− 9.1	1.78E−04	PADI3	19.0	4.69E−03
NR1H4	− 7.6	1.96E−06	RHCG	19.8	5.18E−04
GSTA2	− 7.4	7.07E−03	SPINK6	21.5	4.16E−04
CYP2B22	− 6.8	5.35E−03	WNT7A	25.9	4.23E−02
SLC30A10	− 6.3	2.38E−02	IL4I1L	26.0	1.39E−04
SI	− 6.1	2.79E−02	CLEC18A	46.3	9.53E−05
COL6A6	− 5.7	3.03E−06	PADI1	51.6	6.25E−04
CXCL9	− 5.7	3.65E−03	TFF1	93.2	3.12E−06

Data from a comparison of *Trichuris suis*-infected pigs with (three) and without (five) adult worms.

### Clustering gene expression related to physiological and immunological responses in the proximal colon

Patterns of DEGs were empirically grouped as pathways of host physiology and immunology (Supplemental Tables S7 and S8) and metabolism (Supplemental Tables S9 and S10) obtained from our database, and through the application of Ingenuity Pathway Analysis (IPA) (Table 5) to better conceptualize the response to *T. suis*. IPA is a useful tool; however, pathways like Antiparasitic Responses, Alternatively-Activated Macrophages and other pathways involving Th2 responses tend to be under represented.

Responses in pigs with worms versus controls at 52 DAI generally resembled those at 21 DAI more than pigs without (w/o) worms except there was an expansion of members of each category driven by the developing adult worms. Genes related to the Th2-associated, antiparasitic immune response that were up-regulated at 21 DAI were also up-regulated at 52 DAI (Supplemental Table S7) including AREG (2.1 fold), ARG1 (122.6 fold), IL11 (19.4 fold), SERPINB2 (9.8 fold), TFF1 (705.1 fold), TFF2 (25.5 fold) and REG4 (15.8 fold), CLCA1 (3.2 fold) and MUC2 (5.2 fold). Similarly, several genes for enzymes, related to extra-cellular matrix modification and tissue repair were induced including matrix metalloproteinase 1 (MMP1, 9.4 fold), MMP3 (3.5 fold), MMP7 (33.4 fold), MMP9 (8.9 fold), MMP12 (101 fold), MMP13 (20.2 fold), tissue inhibitor of metalloproteinase 1 (TIMP1, 4.3 fold) and plasminogen activator; urokinase receptor (PLAUR, 4.6 fold), and SERPINB11 (11.3 fold), SERPINB5 (1.9 fold), and SERPINB8 (2.7 fold). The mRNA for some cytokine [IL33 (2.0 fold)] and chemokine [CXCL14 (3.5 fold)] genes and genes in the IL1/IL1R axis including, IL1R2 (10.3 fold), IL1A (3.6 fold), IL1B (5.5 fold), and IL36A (22.7 fold) were also up-regulated.

Several of these genes partially overlapped with the list of genes associated with M2a or alternatively activated macrophages (AAMФs) including ARG1, IL1R2, MMP8, MMP9, MMP12, MMP20, SERPINB2, and TIMP1. Additional genes in this category include prostaglandin-endoperoxide synthase 2/cyclooxygenase-2 (COX-2/PTGS2)^54^ (6.5 fold), acidic chitinase (CHIA)^55^ (2.3 fold), interferon regulatory factor 4 (IRF4)^56^ (2.1 fold), and the universal marker of M2a macrophages^57,58^, transglutaminase 2 (TGM2) (2.2 fold). These are not exclusive markers of M2a macrophages but are consistent with activation of AAMФs in the colon of macaques infected with *T. trichuria* and mice challenged with *T. muris*^59,60^.

In contrast, a large number of genes found in classically activated/interferon stimulated macrophages, involved in antigen processing and presentation, were modestly down-regulated (< 5-fold) in pigs with worms at 52 DAI (Supplemental Table S8) including B2M, CTSD, PSMB10, PSMB8, PSMB9, PSME1, MHC class I (SLA-3, SLA-8, SLA-11) and MHC class II (SLA-DMA, SLA-DMB, SLA-DOA, SLA-DQA1, SLA-DQB1, SLA-DRA and SLA-DRB1) and the peptide transporters TAP1 and TAP2. Some of these (SLA-3, SLA-DMA, SLA-DMB, and SLA-DQB1) were also downregulated at 21 DAI. Although the relative level of inhibition is rather low, the large number of these gene changes in the same direction indicate that the level or function of macrophages, involved in antigen processing and presentation, may be reduced. Additional genes stimulated by type 1 or type 2 interferons and down-regulated at 52 DAI in pigs with a high worm burden include the chemokines CXCL9 (− 7.5 fold), CXCL10 (− 3.4 fold) CXCL11 (− 4.8 fold) and CXCL12 (− 2.7 fold) and their receptor CXCR3 (− 2.2 fold). Other genes associated with the Th1 response were down-regulated included the cytokine, IL12B (− 4.7 fold) and its receptor IL12RB2 (− 2.3 fold) and the regulators of type 1 and 2 IFN signaling^61^; IRF8 (− 1.6 fold) and IFR9 (− 1.8 fold).

### Clustering gene expression related to metabolism in the proximal colon

The group of genes under the broad area of metabolism were placed in the categories of (1) Amino Acid Metabolism or Transport, (2) Carbohydrate Metabolism or Transport, (3) Lipid Metabolism or Transport, (4) Mineral Metabolism or Transport, (5) Vitamin Metabolism or Transport, and (6) Miscellaneous Metabolism or Transport. A very large number of DEGs were assigned to the Lipid Metabolism or Transport category by our own analysis (Supplemental Tables S9 and S10) and by IPA (Table 5). According to IPA analysis, the metabolic pathway that was influenced the most by *T. suis* infection was the super-pathway of cholesterol metabolism (IPA significance level, *p* = 1.585E − 19). This was associated with up regulation in pigs with worms of nearly every gene (MVD, SQLE, NSDHL, IDI1, MSMO1, SC5D, FDPS, FDFT1, DHCR7, EBP, LSS, HMGCS1, CYP51A1) involved in cholesterol synthesis, including the rate limiting step, 3-hydroxy-3-methylglutaryl-CoA reductase (HMGCR, 1.9 fold), a target of anti-cholesterol drugs like statins.

**Table 5:** Selected IPA pathways—metabolism DEGs.

Ingenuity canonical pathways	*p* value
Super-pathway of cholesterol biosynthesis	1.58E−19
LXR/RXR activation	1.66E−05
PXR/RXR activation	7.08E−04
Glycolysis I	7.08E−03
Xenobiotic metabolism signaling	9.00E−03
Urea cycle	1.30E−02
Iron homeostasis signaling pathway	2.20E−02
Aryl hydrocarbon receptor signaling	2.60E−02
Ketolysis	3.50E−02
FXR/RXR activation	3.60E−02
Glucose and Glucose-1-phosphate degradation	4.20E−02
Gluconeogenesis I	4.20E−02

An additional lipid-related metabolic pathway activated by *T. suis* infection and identified by IPA (Table 5) and our own classification (Supplemental Table S9) was the LXR pathway. Although mRNA levels of the 2 receptors (NR1H2, NR1H3) that initiate this pathway did not change, a fairly large number of the genes regulated by them were elevated in pigs that still had *T. suis* worms at 52 DAI including ARG2 (1.7 fold), CYP7A1 (12.7 fold), CYP51A1 (2.1 fold), FDFT1 (1.9 fold), HMGCR (1.9 fold), LDLR (2.8 fold), SCD (3.9 fold) and SREBF1 (2.0 fold).

Another prominent pathway identified by IPA (Table 5) and our own classification involved the bile acid/farnesoid receptor^53^ NR1H4 whose expression was down-regulated − 17.1 fold in pigs with a high worm burden versus uninfected controls (Table 3). Differentially down-regulated NR1H4 target genes include (Supplemental Table S10) the apical bile acid transporter^62^, solute carrier family 10 (sodium/bile acid cotransporter family), member 2 (SLC10A2, − 1,286 fold), the basolateral bile acid transporter^63^, solute carrier family 51 (SLC51A, − 6.9 fold), the phospholipid transporter^64^, member 4 (ABCB4, − 2.7 fold), the cholesterol transporter^65^, phospholipid transfer protein (PLTP, − 1.7 fold) and the glucose transporter (SLC2A4 or GLUT4, − 1.8 fold). Conversely, cytochrome P450, family 7, subfamily A, polypeptide 1 (CYP7A1) that catalyzes the biosynthesis of 7-alpha-hydroxycholesterol, the rate limiting step in the classic bile acid biosynthesis pathway and whose expression is repressed by NR1H4^66^, was up-regulated 12.7 fold (Supplemental Table S9).

### Comparison of gene expressed in the absence of worms versus those driven by worms

A more robust comparative analysis was done on genes expressed at 52 DAI in the proximal colon of infected pigs with worms versus those that were worm-free to look for potential physiological and immunological markers of resistance and tissue remodeling (Supplemental Table S7). Seventy-nine genes were persistently up-regulated in pigs 52 DAI with worms versus pigs that had cleared the worms. Forty two genes were up-regulated in pigs with no worms versus control and 250 genes were up-regulated in pigs with worms versus controls. One hundred and forty-three genes were persistently down-regulated (Supplemental Table S8) in pigs 52 DAI with no worms versus with worms. Thirty-three genes were down-regulated in pigs with no worms versus controls and 266 genes were down-regulated in pigs with worms versus control only. There were no genes that were down-regulated in 52 DAI with worms and up-regulated in pigs with no worms. Similarly, there were no genes that were up-regulated at 52 DAI in pigs with worms and down-regulated in pigs with no worms.

Genes related to Th2 immunity that were differentially expressed in pigs with worms but not in worm-free pigs (Supplemental Table S7) included ARG1 (16.6 fold in infected with worms versus infected with no worms), CHI3L1 (5.2 fold), IL11 (8.3 fold), as well as markers of worm-induced inflammation like TFF1 (93.2 fold), TFF2 (12.2 fold), TFF3 (2.4 fold), SERPINB2 (3.9 fold), SERPINA3-2 (16.2 fold), S100A2 (4.2 fold), S100A8 (10.8 fold), S100A9 (11.0 fold), S100A12 (13.4 fold), REG4 (9.9 fold), MUC2 (3.8 fold), and a series of gene regulating extra-cellular matrix modifications such as MMP1 (3.1 fold), MMP7 (10.5 fold), MMP8 (11.4 fold), MMP9 (2.6 fold), MMP12 (13.9 fold), and MMP13 (10.4-fold). Expression of the genes appears to be inadequate for timely clearance of worms.

### Metabolomic analysis of proximal colon tissue and luminal contents

Uninfected control and *T. suis*-infected pigs were individually housed in confinement on sealed concrete floor and fed a grower pig soybean and corn-based diet that was nutritionally complete. Changes in the metabolites measured in the proximal colon tissue and contents would thus reflect diet independent metabolic turnover of the host tissue, parasitic worms and microbiome, respectively. Metabolites in the tissue and contents were analyzed by both GC/MS and LC/MS at 33 DAI, a time chosen between the two periods analyzed for the host transcriptomic response to *T. suis* at 21 and 52 DAI. The number of L4 isolated from 11 infected pigs sampled at this time was 995.3 ± 200 compared to none from nine control un-inoculated pigs. The analysis showed a total of 341 compounds of known identity (named biochemicals) in colon tissue and 373 in colon contents. Biochemicals that differed significantly between experimental groups (infected versus uninfected) in the tissue and contents were analyzed. The numbers of biochemicals that achieved statistical significance (*p* ≤ 0.05) between infected compared to uninfected treatment groups was 75 (34 higher and 41 lower) in the tissue (Supplemental Table S11) and 111 (52 higher and 59 lower) in the contents (Supplemental Table S12), as well as those approaching significance (0.05 < *p* < 0.10) with 31 in tissue (15 higher and 16 lower) and 35 in the contents (24 higher and 11 lower).

### Elevated methylation pathway metabolites in colon tissue and contents

**Figure 6:**
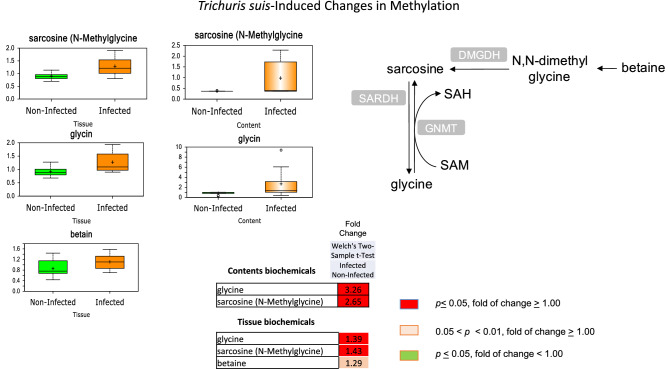
*Trichuris suis*-induced changes in methylation in the proximal colon tissue and contents at day 33 after inoculation. Accumulation of the methyl group scavenger glycine and methylated form of glycine, sarcosine, in colon tissue and contents from pigs infected with *T suis.* Data are shown as “Box and Whiskers” plots with “ + ” as the mean value, “---" as the median value, and “o” as extreme data points. The box indicates upper and lower quartile, while the whiskers represent the maximum and minimum of the distribution. Statistically significant (*p* ≤ 0.05, fold of change ≥ 1.00) increased fold changes in biochemicals are shown in columns highlighted in red and (*p* ≤ 0.05, fold of change < 1.00) decreased fold changes highlighted in green; fold changes at the level of 0.05 < *p* < 0.01, fold of change ≥ 1.00 that approach significance are shown in light red. Analysis was done on 11 T*. suis*-infected pigs (Supplemental Table S1) and nine un-infected control pigs.

Alterations in the methylation of DNA and other cellular constituents can modulate regulatory mechanisms and control various processes including gene expression as a result of parasitic larval invasion and development in the tissues. Significant elevations in the methyl group scavenger glycine and the methylated form of glycine, sarcosine (N-methylglycine), were noted in both colon tissue and contents from pigs infected with *T. suis* at 33 DAI (Fig. 6). In addition, a trending elevation in the tri-methylated version of glycine, betaine, was also observed in tissue from infected pigs. These findings suggest that infection of the colonic mucosa may alter availability of methyl group donors and scavengers and related methylation patterns in the tissue, and these changes are also apparent in contents from infected pigs. These metabolic changes were supported by increased expression of the glycine transporter pathway gene SLC6A9 (1.8-fold) at 52 DAI (Supplemental Table S9).

### *Trichuris suis*-induced increase in antioxidant capacity

**Figure 7:**
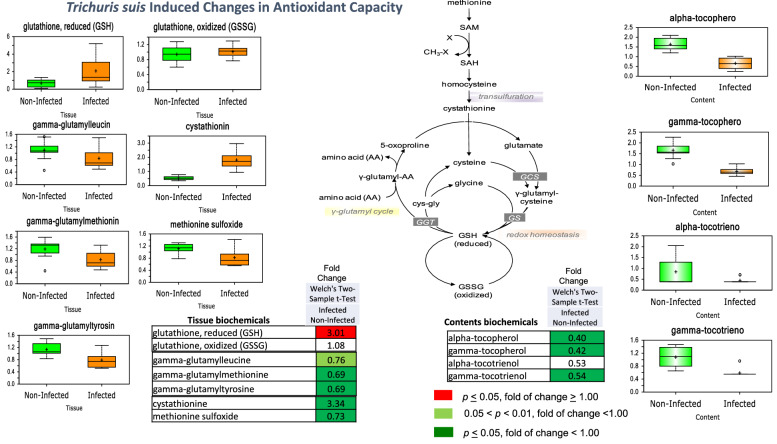
*Trichuris suis* induced changes in antioxidant capacity at day 33 after inoculation. *Trichuris suis* increased antioxidant capacity through increased reduced glutathione (GSH) and decreased fecal loss of several forms of vitamin E. Lower γ-glutamyl amino acids suggested a reduced γ-glutamyl cycle activity associated with decreased glutathione turnover. Data are shown as “Box and Whiskers” bar plots with “ + ” as the mean value, “---" as the median value, “o” as extreme data points, box indicates upper and lower quartile with maximum and minimum of distribution range. Statistically significant (*p* ≤ 0.05, fold of change ≥ 1.00) increased fold change in biochemicals are shown in columns highlighted in red and (*p* ≤ 0.05, fold of change < 1.00) decreased fold change highlighted in green; fold change at the level of 0.05 < *p* < 0.01, fold of change ≥ 1.00 that approach significance is shown in light green. Analysis was done on 11 T*. suis*-infected pigs (Supplemental Table S1) and nine uninfected control pigs.

Antioxidants can ameliorate the oxidative stress and related tissue and cellular damage resulting from infection. Parasitic *T. suis* infection induced a threefold elevation in the antioxidant reduced glutathione (GSH) that was accompanied by unchanged levels of oxidized glutathione (GSSG) in colon tissue (Fig. 7). With the exception of higher levels of the cysteine precursor cystathionine, no biochemicals related to glutathione synthesis in colon tissue were significantly changed by *T suis* infection. However, reductions [some trending (0.10 < *p* < 0.05) or significant (*p* < 0.05)] in several γ-glutamyl amino acids (γ-glutamylleucine, γ-glutamylmethionine, and γ-glutamyltyrosine) were noted in colon tissue from infected pigs, suggestive of lower γ-glutamyl cycle activity and therefore reduced glutathione turnover. A significant reduction in an oxidized form of the amino acid methionine, methionione sulfoxide, in colon tissue and decreased levels of several forms of the antioxidant vitamin E (α-tocopherol, γ-tocopherol, and γ-tocotrienol) in the contents reflects an enhanced antioxidant capacity in the region induced by infection.

### Changes in the proximal colon contents and tissue fatty acid and lipid profiles

**Figure 8:**
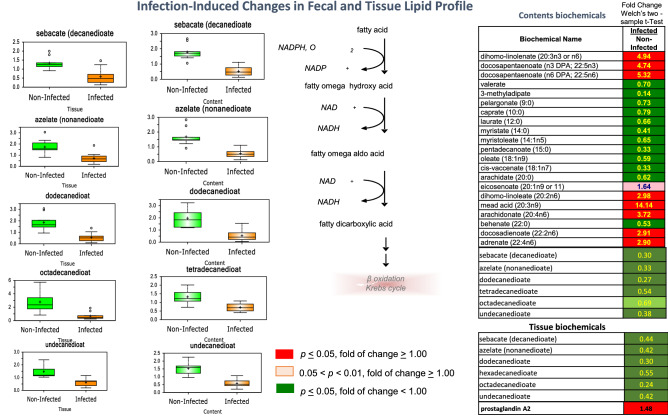
Infection-induced changes in fecal and tissue lipid profile at day 33 after inoculation. *Trichruis suis*-induced changes in dicarboxylate fatty acids are shown as a “Box and Whiskers” bar grafts with “ + ” as the mean value, “---" as the median value, “o” as extreme data points, box indicates upper and lower quartile with maximum and minimum of distribution range, as well as essential and long chained fatty acids shown in columns of fold change. Statistically significant (*p* ≤ 0.05, fold of change ≥ 1.00) increased fold change in biochemicals are shown highlighted in red and (*p* ≤ 0.05, fold of change < 1.00) decreased fold change highlighted in green; fold change at the level of 0.05 < *p* < 0.01, fold of change ≥ 1.00 that approach significance are shown in light red. Analysis was done on 11 T*. suis*-infected pigs (Supplemental Table S1) and nine uninfected control pigs.

There was a significant reduction in dicarboxylic acids such as sebacate (decanedioate), azelate (nonanedioate), dodecanedioate, octadecanedioate, and undecanedioate in the colon tissue and contents (Fig. 8). Dicarboxylic fatty acids can be produced when very long-chain fatty acids are metabolized via the minor endoplasmic reticulum/peroxisomal-mediated ω-oxidation pathway and are subsequently degraded through mitochondrial β-oxidation. Therefore, the consistent reduction in dicarboxylic acids may be indicative of reduced entry of very long-chain fatty acids into the ω-oxidation pathway or increased entry of dicarboxylic acids into the β-oxidation pathway. In addition, accumulation of some essential fatty acids and many unsaturated long-chain fatty acids (≥ 20 carbons) with concurrent reductions in medium-chain fatty acids and a number of saturated long-chain fatty acids was observed. This pattern may be indicative of altered transport of unsaturated and saturated fatty acids across the gut wall and could also be the result of changes in gut microbial metabolism of fatty acids in the colon after infection. Other lipid-related changes included an increase endocannabinoids, monoacylglycerols, sphingolipids (sphingosine, N,N-dimethylsphingosine, and palmitoyl sphingomyelin), and cholesterol in the colon contents. Changes in genes expression related to lipid metabolism were prominently represented in Supplemental Tables S9 and S10.

### Infection-induced accumulation of amino sugars in colon tissue and contents

**Figure 9:**
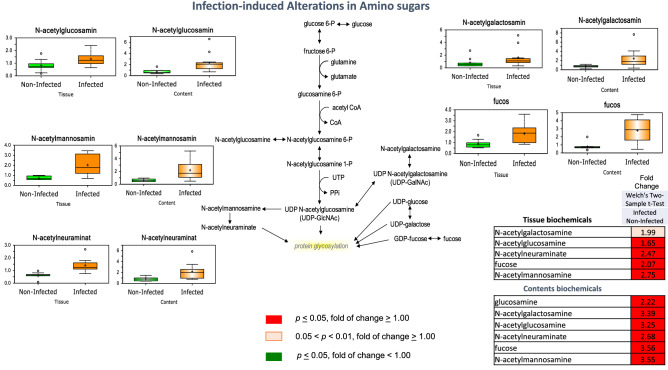
Infection-induced alterations in Amino sugars at day 33 after inoculation. Glucose-derived amino sugars accumulated in colon tissue and contents from pigs infected with *T suis*. These changes are shown as a “Box and Whiskers” bar grafts with “ + ” as the mean value, “---” as the median value, “o” as extreme data points, box indicates upper and lower quartile with maximum and minimum of distribution range. Statistically significant (*p* ≤ 0.05, fold of change ≥ 1.00) increased fold change in biochemicals are shown highlighted in red and (*p* ≤ 0.05, fold of change < 1.00) decreased fold change highlighted in green; significant increased fold change at the level of 0.05 < *p* < 0.01, fold of change ≥ 1.00 is shown in light red; fold change at the level of 0.05 < *p* < 0.01, fold of change ≥ 1.00 that approach significance are shown in light green. Analysis was done on 11 T*. suis*-infected pigs (Supplemental Table S1) and nine uninfected control pigs.

Amino sugars are important for glycosylation of proteins and extracellular matrix structure. A consistent and significant increase in many glucose-derived amino sugars, including N-acetylglucosamine, N-acetylmannosamine, N-acetylneuraminate, N-acetylgalactosamine, and fucose was observed in colon tissue and contents from pigs infected with *T. suis* (Fig. 9). Similar to potential alterations in methylation patterns, changes in amino sugars may be related to altered protein glycosylation and changes in gene expression or extracellular matrix structure associated with the *T. suis* infection process.

### Differential changes in various sugars and Krebs cycle intermediates

**Figure 10:**
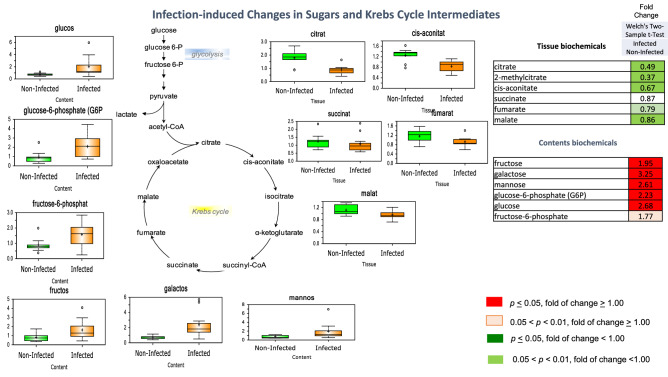
Infection-induced changes in sugars and Krebs cycle intermediates at day 33 after inoculation. Infection with *T suis* facilitated accumulation of carbohydrates and glycolytic intermediates in colon content and reduced Krebs cycle intermediates in colon tissue shown as a “Box and Whiskers” bar grafts with “ + ” as the mean value, “---" as the median value, “o” as extreme data points, box indicates upper and lower quartile with maximum and minimum of distribution range. Statistically significant (*p* ≤ 0.05, fold of change ≥ 1.00) increased fold change in biochemicals are shown highlighted in red and (*p* ≤ 0.05, fold of change < 1.00) decreased fold change highlighted in green; fold change at the level of 0.05 < *p* < 0.01, fold of change ≥ 1.00 that approach significance are shown in light red. Analysis was done on 11 T*. suis*-infected pigs (Supplemental Table S1) and nine uninfected control pigs.

Liberation of energy from carbohydrates in the form of ATP through glycolysis and mitochondrial oxidative phosphorylation is a fundamental biochemical pathway important for maintenance of energy demands. Levels of various sugars and glycolytic intermediates were not greatly affected by *T. suis* infection in colon tissue, but a pronounced reduction in nearly all Krebs cycle intermediates, including citrate, cis-aconitate, fumarate, and malate, and elevations in several monosaccharides (fructose, galactose, mannose, and glucose) and glycolytic intermediates (glucose 6-phosphate and fructose 6-phosphate) with a reduction in only one Krebs cycle intermediate, alpha-ketoglutarate, were noted in the contents from infected pigs (Fig. 10). The global reduction in Krebs cycle metabolites in colon tissue may be indicative of either increased cycle activity (and resulting depletion of intermediates) or decreased cycle activity (associated with lower levels of intermediates). Accumulation of various carbohydrates and glycolytic intermediates in the contents could be attributable to altered uptake from the gut lumen and changes in utilization by bacteria that comprise the gut microbiome. This is clearly indicative of significant alterations in key biochemical pathways of energy metabolism in response to *T. suis* infection. Gene expression for HK2 was up-regulated 3.3-fold at 21 DAI and several other genes related to carbohydrate synthesis were up-regulated at 52 DAI in the presence of worms (Supplemental Table S9).

### Infection-altered composition and/or activity of intestinal bacteria

**Figure 11:**
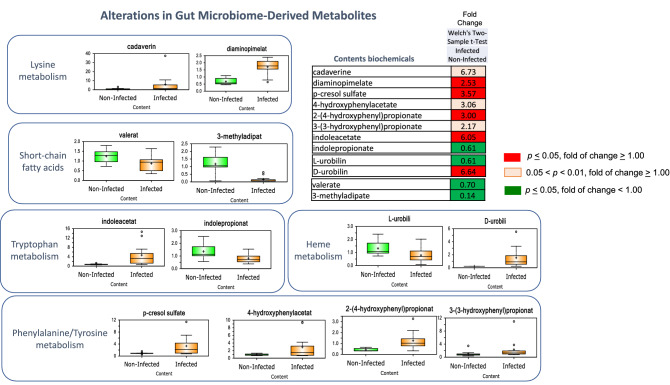
Alterations in gut microbiome-derived metabolites at day 33 after inoculation. *Trichuris suis* induced substantial changes in metabolites derived from gut microbiome activityand in colon tissue. Differences are shown as a “Box and Whiskers” bar grafts with “ + ” as the mean value, “---" as the median value, “o” as extreme data points, box indicates upper and lower quartile with maximum and minimum of distribution range. Statistically significant (*p* ≤ 0.05, fold of change ≥ 1.00) increased fold change in biochemicals are shown highlighted in red and (*p* ≤ 0.05, fold of change < 1.00) decreased fold change highlighted in green; fold change at the level of 0.05 < *p* < 0.01, fold of change ≥ 1.00 that approach significance are shown in light red. Analysis was done on 11 T*. suis*-infected pigs (Supplemental Table S1) and nine uninfected control pigs.

*Trichuris suis* infection altered the profile of several bacterial metabolites, including biochemicals derived from amino acids, heme, and short-chain fatty acids. Accumulation of the bacterial-derived lysine metabolites cadaverine and diaminopimelate and phenylalanine/tyrosine metabolites p-cresol sulfate, 4-hydroxyphenylacetate, 2-(4-hydroxyphenyl)propionate, and 3-(3-hydroxyphenylpropionate) was observed in colon contents from infected pigs (Fig. 11). The tryptophan-derived metabolites indoleacetate and indoleproprionate and heme-derived metabolites L-urobilin and D-urobilin were differentially affected (i.e., one increased and one decreased) and depletion of the short-chain fatty acids derived from gut bacteria, valerate and 3-methyladipate, was also observed. Some of the changes were replicated in colon tissue, with trending or significant changes noted in p-cresol sulfate, 4-hydroxyphenylacetate, and indole acetate. These findings suggest altered transport of these molecules in the intestine of pigs infected with *T. suis.* Several of these changes are also supported by Kyoto Encyclopedia of Genes and Genomes (KEGG) and Gene Ontology (GO) analysis of the metabolic potential of the microbiome in pigs infected with *T. suis* at 21 DAI^16^.

## Discussion

Hatching of infective *T. suis* eggs in the pig intestine and the parasitic invasion of the mucosal layer and epithelial cells in the proximal colon to initiate intracellular larval feeding and development perturbs the cytoskeleton matrix and cell junctions^67^. These early events alter the local microbiome as parasite mucolytic components released during larval invasion provide substrates to expand bacteria such as the *Mucispirillum*^16^, and parasite products that facilitate tissue penetration and worm metabolism activate and react to host immune protective responses^68^. The interplay of host, parasite, and microbiome has consequences for parasite survival and the intensity and persistence of host inflammation^16–18^ The earliest events recorded in this current study showed that at 21 DAI there was an induction of gene expression to activate type 2 related immune responses including M2a macrophage that have been shown to regulate intestinal smooth muscle function via SERPINB2^36^, and that has been linked to a STAT6-dependent effects on smooth muscle hyper-contractility associated with protection against *T. muris* in mice (Madden et al., unpublished). In addition, expression of an array of genes for enzymes involved in extra-cellular matrix modification and tissue repair, mucosal integrity, inflammation, and oxidative stress (Supplemental Table S9), as well as a group of lipid mediators and cholesterol synthesis genes are up-regulated and complemented by metabolic changes in the proximal colon tissue and contents (Figs. 6, 7, 8). There is, however, an apparent fine level of control of local inflammation that may be most appropriate to the immediate need to repair tissue since several genes associated with colonic inflammation and chemokines used for infiltration of B and T cells, T reg and NK are initially down-regulated (Supplemental Table S8).

*Trichuris suis*-induced gene expression changes were consistent with goblet cell hyperplasia, influx or in situ alternative activation of macrophages and mast cells and molecules associated with tissue remodeling/wound healing. The early reduction in basal flux of ions across the proximal colon mucosa (Fig. 2a) following whipworm infection and the changing energy needs in the tissue and contents (Figs. 8, 9, 10) could favor an activation of innate cellular protective mechanisms^69^ and modulate local bacterial diversity^16^ as changing nutrients from host and parasite metabolism become available. The observed reduction in basal flux in the proximal colon persisted through 52 DAI even in pigs that were worm-free, but a further reduction in basal flux in response to acetylcholine was worm-dependent (Fig. 2b), microbiome related^17^, and different from that observed in the small intestine of pigs^70^ and mice^71^ infected with lumen dwelling parasitic worms. The protective response to whipworm as an intracellular parasite in the colon is associated with altered tryptophan metabolism and a broad induction of genes related to cholesterol and vitamin B12 metabolism reflective of rapid cell turnover^72^.

Trefoil factor 1 (TFF1) and TFF2, two STAT6-dependent, goblet cell-associated proteins^73^, were among the most highly regulated *T. suis*-induced genes. Furthermore, TFF2 was among the genes differentially expressed by *T. suis*-infected pigs with a high and low worm burden. In rodent models, TFF1 and TFF2 protect the mucosa from insult, stabilize the mucus layer, and promote healing of the epithelium^38,74^. TFF2 is thought to modulate Th2 responses by inducing the expression of IL-33^38^. Interleukin 33 was modestly upregulated by *T. suis* infection. By PCR, MUC5AC was also among the genes differentially expressed by *T. suis* infected pigs with a high and low worm burden as well as in *T. muris* infected mice^24,75^. MUC5AC a secreted gel forming mucin, is required for in the expulsion of the enteric nematodes *T. muris, Trichinella spiralis and Nippostrongylus brasiliensis* in mice^76^. In addition, human MUC5AC inhibits the growth of *T. muris* in culture^76^.

Gene expression analysis strongly indicated that de novo cholesterol synthesis was initiated by *T. suis* infection. These findings are important for several reasons. First, it suggests that regulation/limiting de novo cholesterol synthesis by agents like statins may prove effective in worm control either by themselves or in conjunction with other agents. Although one *Trichuris* species, *T. globulosa*, can reportedly synthesize cholesterol^77^, the consensus is that parasitic nematodes are dependent on host cholesterol^78,79^. Statins have proven effective against schistosomiasis in experimental models^80^. It is not clear whether the effects are direct (parasite metabolism) or indirect (effects on the host). Infection with *T. suis* has the potential to affect whole body lipid metabolism. Slightly less than half of the cholesterol in the body derives from de novo biosynthesis of which liver and intestines for 10% and 15% of the amount produced each day, respectively. Under basal conditions, extra hepatic cells obtain sufficient cholesterol from the plasma rather than de novo synthesis. However, low-level cholesterol synthesis has been observed in human, rodent and pig intestine^81–83^. A previous human study^84^ showed a significant inverse correlation between worm egg excretion and plasma HDL levels and *T. trichiura* infection. The authors of that study speculated that a mechanism for this may have included reduced HDL synthesis by the gut wall. Notably, our metabolomic analysis revealed no changes in colon tissue cholesterol or its precursors, squalene or lathosterol, at 33 DAI. However, a 1.4 fold increase in luminal content of cholesterol, but not lathosterol, and a decrease in the phytosterols, beta-sitosterol and campesterol was observed.

The bile acid/farnesoid receptor pathway was also significantly altered by *T. suis* infection at 21 and 52 DAI. Elevated bile acid levels activate NR1H4 to decrease bile acid biosynthesis. Of note, our metabolic analysis did not find a change in the colon tissue or intraluminal content levels of 7-alpha-hydroxycholesterol between infected and uninfected pigs at 33 DAI. Among tissue bile acid precursors or metabolites, only alpha-muricholate was increased in the *T. suis*-infected pigs. Analysis of luminal contents indicated a 4.6 fold increase in hyocholate and a decrease in dehydrolithocholate (2.6 fold) and 6-oxolithocholate (1.7 fold). Several lines of evidence strongly suggest that NR1H4 is a key mediator of intestinal immune homeostasis. Disruption of the *Nr1h4 gene* in mice leads to increased intestinal epithelial cell proliferation^85^ and increased levels of bacteria and a compromised epithelial barrier^86^. Colon inflammation in rodent models of colitis and in Crohn’s disease patients, is associated with a reduced expression of NR1H4 mRNA^87^. Finally, genetic variations in the *NR1H4* gene are associated with a predisposition to human IBD^88^.

Several, pro or anti-inflammatory bioactive lipid mediators were elevated in the proximal colon content or colon tissue of *T. suis*-infected animals (Table 6). Docospentaenoate (n3 DPA; 22:5n3) was up-regulated 4.7 fold in proximal colon content of infected pigs at 33 DAI. It was non-significantly decreased 1.5 fold in tissue. Docosapentaenoate is a precursor to a class of mediators known as resolvins and protectins^89^. Protectin D1 and resolvin D5 protected against colitis and intestinal inflammation in experimental colitis in mice and explanted human colon tissue^90^.

**Table 6:** Metabolomics—bio-active lipids. Analysis of the proximal colon contents was done on 11 *T. suis*-infected pigs (Supplemental Table S12) and nine un-infected control pigs.

Sub-pathway	Metabolite	Fold change	Significance
Long chain fatty acid	Mead acid (20:3n9)	14.1	0.0001
Essential fatty acid	Docosapentaenoate (n6 DPA; 22:5n6)	5.3	0.0001
Essential fatty acid	Docosapentaenoate (n3 DPA; 22:5n3)	4.7	0.0004
Endocannabinoid	Palmitoyl ethanolamide (PEA)	4.4	0.0069
Endocannabinoid	Oleic ethanolamide (OEA)	3.2	0.0220
Endocannabinoid	Stearoyl ethanolamide	2.1	0.0303
**Colon tissue**			
Eicosanoid	Prostaglandin A2	1.5	0.0344

Receptors for endocannabinoids lipids have been identified throughout the gastrointestinal tract as well as on neurons comprising the enteric nervous system that can regulate intestinal motility and secretions and play a role in inflammation. The endocannabinoid, palmitoyl ethanolamide (PEA), a PPAR-alpha agonist^91^ was significantly up-regulated 4.4 fold in the luminal contents of infected pigs. PEA exhibits anti-inflammatory effects in chemically-induced colitis in mice^92^ and explanted human colon tissue^93^. Two additional endocannabinoids, oleic ethanolamide (OEA) a PPAR-α agonist^94^ and stearoyl ethanolamide (SEA), a non-PPAR-α agonist^91^, were elevated in colon contents. A large in increase in mead acid (14 fold) was noted in the colon contents from infected pigs (Table 6). Mead acid is converted to leukotrienes C3 and D3, potent mediators of smooth muscle contractions^95^. Mead acid is also metabolized by 5-lipoxygenase (ALOX5) to form 5-oxoeicosatrienoic acid (5-oxo-ETrE), a highly potent eosinophil chemoattractant^96^. An increase in prostaglandin A2 (PGA2) was also noted in colon tissue. PGA2 is a negative regulator of inflammation in various models^97–99^. Prostaglandin A2 is generated by (COX-2, PTGS2) from PGE2. The mRNA for PTGS2 was significantly up-regulated 6.5 fold in the tissue of *T. suis* infected pigs.

A large number of genes involved in glycolysis, gluconeogenesis or the TCA cycle were regulated by *T. suis* infection. By numerical count *T. suis* infection had the greatest effect on genes involved in glycolysis, up-regulating 6 of 25 genes involved in the pathway including the enzyme, hexokinase 2 (HK2, 8.6 fold) the performs the rate-limiting, first step, in glycolysis^100^. Phosphorylation of glucose to form glucose-6-phosphate (G6P), traps glucose inside of the cell and commits it to the glycolytic pathway. Interestingly, only colon luminal contents, but not tissue, revealed greater levels of glucose and G6P. In contrast, the expression of phosphoenolpyruvate carboxykinase 1 (PCK1), the first, rate-limiting, step in gluconeogenesis, was down-regulated − 16.9 fold. Three genes involved in the synthesis of acetyl CoA, AACS (2.0 fold), ACAT2 (3.5 fold) and ACLY (2.2 fold) were up-regulated by infection; however, this compound and it precursors or metabolites were largely unaltered by infection. In contrast, infection led to a decrease in several TCA cycle associated metabolites; citrate (2.0 fold), 2-methylcitrate (2.7 fold), cis-aconitate (1.5 fold) and malate (1.2 fold).

Also notable were changes in amino acid metabolism indicated by strong induction of ARG1 in *T. suis*-infected pigs with either a high and low worm burden. We and others have previously reported that ARG1 was up-regulated (from week 3 to week 11) in the proximal colon of *T. suis*-infected pigs^24,75^. Arginase 2 (ARG2, 1.7 fold) and the arginine transporters^101^ SLC7A1 (1.6 fold) , SLC7A2 (1.9) and SLC7A6 (1.7 fold) were up-regulated, at a low level, by *T. suis* infection. However, our metabolomic data shows that neither arginine nor the great majority of its metabolites were altered in colon tissue or colon luminal contents.

Diet, especially the fermentability and structure of carbohydrates, has been shown in several studies to limit the infectivity and fecundity of whipworm in pigs, as well as the intestinal morphology and mucin biosynthesis in the colon^102–104^. This has been attributed to short-chain fatty acids as fermentation end products that lower the pH in the colon and also enhance epithelial cell vigor^105^. Evidence is mounting that these metabolic features are microbiome-dependent and contribute to the control of infection and level of inflammation in pigs^16,17^ but are also relevant to intestinal inflammation in humans^106,107^. Recent evidence has shown that other dietary components such as omega-3-enriched krill oil can enhance the diversity of the intestinal microbiome and level of inflammation in pigs infected with *T. suis*^108^. Dietary interventions that modulate metabolic and infectious diseases that are also important in humans have been used successfully in pigs supplemented with defined micro- and macro-nutrients^109,110^, phytonutrients^111^ and probiotics^112^ as well as more complex food matrices that also effect change in microbiome linked to disease outcome (Solano-Aguilar et al., in review). Recent evidence showing that *T. muris* acquire an endogenous intestinal microbiome from the host^18^ and that parasite-induced changes in the host microbiome suppress infective larval release from eggs given in challenge infections illustrates the interactive complexity of the host/parasite/microbiome and the potential of these interaction to reduce infection and host inflammation that would benefit the health of both pigs and humans.

## Materials and methods

### Isolation of T. suis life cycle stages

Pig (*Sus scrofa*) management and handling procedures were approved by the Beltsville Area Animal Care and Use Committee. Mixed-sex pigs (crossbred: Landrace × Yorkshire × Poland China) of approximately three months of age were inoculated as previously described^17^ with a single dose of infective *T. suis* eggs ( pig dosing shown in Supplemental Table S1). *Trichuris suis* larval stages and adult worms were collected at various time points, between day 21 to 56 post-inoculation, from the cecum and proximal colon^17^. The pigs were free of inadvertent *Ascaris suum* infection (except where noted in Supplemental Table S1) based on the absence of worms from the small intestines and white spot lesions on the liver. Pigs used in these studies were free of other helminth infections as measured by routine screening of the herd and the absence of worms in the intestinal contents and liver lesions at necropsy. All animal studies were reviewed and approved by the Beltsville Area [Institutional] Animal Care and Use Committee (IACUC) under protocols #07-011, #10-001 and 012 and #13-019 and 020, and all methods were carried out in accordance with relevant guidelines and regulations.

### Relative mRNA host gene expression by quantitative real time PCR

Approximately 3 mm^3^ tissue sections from the small intestine ilium (IL), large intestine proximal colon (PCM) and distal colon (DCM), mesenteric (MLN), colonic (CLN), and tracheal-bronchial lymph nodes (TBLN), jejunum (JJ PP) and ileal-cecal Peyer’s patch (IC PP), and lympho-glandular complexes (LGC) were collected at necropsy and frozen immediately in liquid nitrogen and stored at − 70 °C until RNA extraction. RNA was extracted from cells using according to manufacturers’ instructions and processed as previously described^24^. Briefly, all RNA samples were DNAase treated in the presence of an RNAase inhibitor (DNA-free, Ambion) to remove genomic DNA. RNA integrity, quality, concentration and DNA contamination were assessed using Agilent (RNA 6,000 Nano Assay, Agilent Technologies). Total RNA was used for first strand cDNA synthesis using SuperScript II (Invitrogen, Life Technologies) and oligo(dT). This cDNA was used as a template for target-specific amplification by real-time PCR to evaluate the expression of a panel of cytokine, antibody and receptor genes. All primers and Taqman probes (5′ TET- and 3′ BHQ1 labelled, Biosource, Camarillo, CA) were designed across adjacent exons when possible using Primer Express software v1.5 (Applied Biosystems, Foster City, CA). The sequences of the assays and concentrations are found in the Porcine Translational Research Database maintained by this laboratory^27^ (https://tinyurl.com/hxxq3ur). PCR was performed using a commercial kit (Brilliant kit, Stratagene) on an ABI PRISM 7,700 Sequence Detector System (Applied Biosystems). Fluorescence signals measured during amplification were processed post-amplification and were regarded as positive if the fluorescence intensity was 20-fold greater than the standard deviation of the baseline fluorescence (Ct-value). For each experiment several housekeeping genes are tested for their lack of change due to treatment, data for gene expression are then adjusted for the appropriate housekeeping gene using the 40 − ΔCT method^113^.

### *Measurement of epithelial cell function *ex vivo

Four segments of mucosa (0.126 cm^2^) were stripped of muscle and mounted in Ussing chambers exposed to 10 mL of Kreb’s buffer and the tissues further processed as previously described^70^. All responses from treated-tissue segments from control and *T. suis* infected-pigs were averaged to yield a mean response per treatment group. Mucosal segments from jejunum also were collected for measurement of similar physiological responses (data not shown).

### RNA sequencing (RNASeq) analysis

A cDNA Library for each mRNA sample was prepared with mRNA-Seq 8 sample prep kit from Illumina (San Diego, CA) as previously described^58^. Briefly, polyadenylated mRNA was isolated from 10 µg total RNA by poly-T beads and fragmented chemically. Complementary DNA (cDNA) was synthesized from fragmented RNA followed by end repair and adenylation. Ligation was performed with adapters from Illumina. The 200 bp adenylated cDNA was separated by gel electrophoresis and isolated by manual extraction of the band and elution from gel material. PCR amplification was carried out to enrich the cDNA in the library. The size and quality of amplified cDNA were validated by Experion DNA Analysis Kits (BioRad). Samples were sequenced on a Genome Analyzer II (Illumina) at the Bovine Functional Genomics Laboratory, Beltsville Agricultural Research Center, Beltsville, MD**.** An average of 25–30 million 80-bp reads from three pigs was obtained. Reads with a Phred quality score below 30 or containing more than 2 ambiguous nucleotides were filtered and discarded before data analysis with the CLC Genomics Workbench version 11.0 (QIAGEN Bioinformatics, Redwood City CA)). FASTA files of all reads from experiment will be submitted to NCBI Short Read Archive database under the accession numbers. Gene expression was normalized using the “reads per kilobase of exon model per million mapped reads” model (RPKM)^114^. Mapping of reads to the current assembly of the pig genome build 11.1 (WG) or a custom, non-redundant (NR) 9,757 gene library^58^ was performed with the RNA-Seq program of CLC. The sequences for these genes are found in the Porcine Translational Research Database maintained by this laboratory (https://tinyurl.com/hxxq3ur) ^27^. Inclusion of the custom library was necessary because the current version of the porcine genome (11.1) contains a significant number of errors and underrepresentation of 5′ and 3′ region of genes^27,58^. This custom library includes full 5′ and 3′ representation of the vast majority of known immune system components and genes involved in macro and micronutrient metabolism.

Principle component analysis (PCA) and Analysis of Variance (ANOVA) using treatment and worm number as fixed effects were performed using JMP Genomics, Version 9. Differentially expressed genes (DEGs) (> or < 1.5 fold) with an adjusted FDR of 0.05 or less by EDGE R analysis were chosen for downstream analysis. Principle component analysis (PCA) and mixed model analysis of variance (ANOVA) using worm number as a covariate were performed using JMP Genomics, Version 12.1. Differentially expressed genes were further investigated via Ingenuity Pathway Analysis software (IPA, Ingenuity Systems, CA) for discovery of biological significance and their association with major canonical pathways, function and disease.

### Liquid chromatography-coupled tandem-mass spectrometry (LC–MS/MS)

All samples were processed by Metabolon, Inc. (Morrisville, NC). Briefly, sample preparation involved protein precipitation and removal with methanol, shaking and centrifugation. The resulting extracts were divided into fractions for analysis on three independent platforms: ultrahigh-performance liquid chromatography/tandem mass spectrometry (UHPLC/MS/MS) optimized for basic species, UHPLC/MS/MS optimized for acidic species, and GC/MS. The details of this platform have been described previously^115^. Metabolites were identified by automated comparison of the ion features in the experimental samples to a reference library of chemical standard entries that included retention time, molecular weight (*m/z*), preferred adducts, and in-source fragments as well as associated MS spectra, and were curated by visual inspection for quality control using software developed at Metabolon^116^.

### Statistical analyses

Where applicable, statistical significance was determined using one-way ANOVA followed by Bonferroni post-hoc test; calculations were performed using Prism software (GraphPad Software, La Jolla CA). Differences were considered significant when *p < 0.05, **p < 0.01, ***p < 0.001, while not significant differences are indicated by “*ns*”.

Metabolomic data was analyzed using two types of statistical analyses. (1) The Welch’s t-tests and ANOVA procedures (e.g., repeated measures ANOVA) where appropriate. (2) Statistical analyses are performed with the program “R” https://cran.r-project.org/.

## Supplementary information

Supplementary information 1.

Supplementary information 2.

Supplementary Table S3.

Supplementary Table S4.

Supplementary Table S5.

Supplementary Table S6.

Supplementary Table S7.

Supplementary Table S8.

Supplementary Table S9.

Supplementary Table S10.

Supplementary Table S11.

Supplementary Table S12.

## Data Availability

Raw reads from transcriptomic analyses were deposited in the GenBank Sequence Read Archive under Bioproject PRJNA493809. Genomic sequencing and gene annotation data are available on the NCBI sequence read archive (Bio-sample) or our online database (https://tinyurl.com/hxxq3ur). All other datasets generated and analyzed during the current study are available upon request from qualified researchers.
