# Supplementary material for: Molecular and metabolomic changes in the proximal colon of pigs infected with Trichuris suis
Source: Sci Rep. 2020 Jul 30;10:12853. doi: 10.1038/s41598-020-69462-5 (PMC7393168; doi:10.1038/s41598-020-69462-5)
Supplement: Supplementary file 1 — Supplementary information 1. [file 41598_2020_69462_MOESM1_ESM.pdf]

Molecular and metabolomic changes in the proximal colon of pigs infected with *Trichuris suis*

Harry D. Dawson[1], Celine Chen[1], Robert W. Li[2], Lauren Nicole Bell[3], Terez Shea-Donohue[4], Helene Kringel[5], Ethiopia Beshah[1], Dolores E. Hill[2], Joseph F. Urban Jr[1,2].

[1]United States Department of Agriculture, Agricultural Research Service, Northeast Area, Beltsville Human Nutrition Research Center, Diet Genomics and Immunology Laboratory, [2] Beltsville Agricultural Research Center, Animal Parasitic Disease Laboratory, Beltsville, MD; [3]Metabolon, Inc., Morrisville, NC; [4]University of Maryland School of Medicine, Baltimore, MD, USA; [5]Department of Veterinary Disease Biology, Faculty of Health and Medical Sciences, University of Copenhagen, Copenhagen, Denmark

Supplemental Tables S1, S3, S4, S5, S6, S7, S8, S9, S10, S11, S12

Supplemental Table S1. Isolation of larval and adult stages of *Trichuris suis* from the cecum and proximal colon.

Exp #/Source	Pig ID/sex	Sow ID/date of birth	*Trichuris* batch # / ~egg dose	DPI	*Trichuris* recovery	*Ascaris* adults/ liver spots	Proximal colon mucosa pathology/colon contents	Molecular/metabolic Analysis
#15-19/BARC	1092/B	689/08/07/2015	#15-01 - 10,000	42	900	0/0	hemorrhagic/loose	
	1096/B	163/08/05/2015	#15-01 - 10,000	42	0	0/0	lightly hemorrhagic/semi-solid	
	1128/B	1181/08/14/2015	#15-01 - 10,000	42	1900	0/0	hemorrhagic/loose	
	1103/B	61/08/06/2015	#15-01 - 10,000	42	5435	0/0	hemorrhagic/loose	
	notag/B		#15-01 - 10,000	42	730	0/0	hemorrhagic/loose	
	1088/B	1672/08/08/2015	#15-01 - 10,000	42	711	0/0	lightly hemorrhagic/loose	
	1125/B	1185/08/14/2015	#15-01 - 10,000	42	2775	0/0	hemorhhagic	
	notag/B		#15-01 - 10,000	42	1010	0/0	lightly hemorrhagic/loose	
	1115/B	721/08/11/2015	#15-01 - 10,000	42	76	0/0	lightly hemorrhagic/semi-solid	
	1116/B	861/08/12/2015	#15-01 - 10,000	42	1740	0/0	hemorrhagic/loose	
#15-19/BARC	1102/B	868/08/05/2015	#15-01 - 10,000	49	3795	0/0	lightly hemorrhagic/loose	
	1118/B	490/08/14/2015	#15-01 - 10,000	49	400	0/0	hemorhhagic/loose/bloody	
	1095/B	689/08/07/2015	#15-01 - 10,000	49	2250	0/0	lightly hemorrhagic/loose	
	1104/B	1535/08/06/2015	#15-01 - 10,000	49	1286	0/0	lightly hemorrhagic/loose	
	1123/B	721/08/11/2015	#15-01 - 10,000	49	2372	0/0	lightly hemorrhagic/loose	
	1119/B	861/08/12/2015	#15-01 - 10,000	49	1615	0/0	lightly hemorrhagic/loose	
	1114/B	861/08/12/2015	#15-01 - 10,000	49	1652	0/0	lightly hemorrhagic/loose	
	1111/B	861/08/12/2015	#15-01 - 10,000	49	415	0/0	hemorrhagic/loose	
	1105/B	861/08/12/2015	#15-01 - 10,000	49	355	0/0	lightly hemorrhagic/loose	
	1107/B	1181/08/14/2015	#15-01 - 10,000	49	1602	0/0	lightly hemorrhagic/loose	
#15-11/Oakhill	64376/B	108/05/04/2015	#13-02 - 15,000	56	0	0/0	lightly hemorrhagic/loose	
	64370/B	756/05/02/2015	#13-02 - 15,000	56	6000*	0/0	hemorrhagic/loose	
	64398/B	702/05/01/2015	#13-02 - 15,000	56	6280	0/0	hemorrhagic/loose	
	64378/B	910/05/02/2015	#13-02 - 15,000	56	5050	0/0	hemorrhagic/loose	
	64366/B	46/04/30/2015	#13-02 - 15,000	56	6350	0/0	hemorrhagic/loose	
	64405/B	890/05/02/2015	#13-02 - 15,000	56	1300*	0/0	hemorrhagic/loose	
	64415/B	756/05/02/2015	#13-02 - 15,000	56	10	0/0	normal/loose	
	64375/B	768/04/28/2015	#13-02 - 15,000	56	3,000	0/0	hemorrhagic/loose	
	64380/B	120/05/01/2015	#13-02 - 15,000	56	50	0/0	normal/loose	
	64379/B	768/04/28/2015	#13-02 - 15,000	56	4680	0/0	hemorrhagic/loose	
	64388/B	863/04/30/2015	#13-02 - 15,000	56	800	0/0	hemorrhagic/loose	
#15-1/Oakhill	23824/B	532/12/05/2014	#13-02 - 10,000	28	9500	0/0	hemorrhagic/liquid	
	23796/B	1023/11/28/2014	#13-02 - 10,000	28	15,000	0/0	hemorrhagic/liquid	
	23802/B	1221/11/29/2014	#13-02 - 10,000	28	1,700	0/0	strongly hemorrhagic/liquid	
#15-1/Oakhill	23816/B	1550/12/05/2014	#13-02 - 10,000	53	1130	0/0	normal/normal	
	23828/B	Oakhill/	#13-02 - 10,000	53	1000	0/0	lightly hemorrhagic/loose	
	23829/B	Oakhill/	#13-02 - 10,000	53	0	0/0	normal/loose	
	23837/B	Oakhill/	#13-02 - 10,000	53	1500*	0/0	lightly hemorrhagic/loose	
	23831/B	Oakhill/	#13-02 - 10,000	53	1600	0/0	lightly hemorrhagic/loose	
	23844/B	Oakhill/	#13-02 - 10,000	53	0	0/0	normal/normal	
	23845/B	Oakhill/	#13-02 - 10,000	53	800	0/0	normal/normal	
	23848/B	Oakhill/	#13-02 - 10,000	53	65	0/0	normal/normal	
	23849/B	Oakhill/	#13-02 - 10,000	53	1100	0/0	lightly hemorrhagic/loose	
#15-1/Oakhill	64376/B	108/05/04/2015	#13-02 - 10,000	56	0	0/0	lightly hemorrhagic/normal	
	64370/B	756/05/02/2015	#13-02 - 10,000	56	6000*	0/0	hemorrhagic/normal	
	64398/B	702/05/01/2015	#13-02 - 10,000	56	6280	0/0	hemorrhagic/normal	
	64378/B	910/05/02/2015	#13-02 - 10,000	56	5050	0/0	hemorrhagic/normal	
	64366/B	46/04/30/2015	#13-02 - 10,000	56	6350	0/0	hemorrhagic/normal	
	64405/B	890/05/02/2015	#13-02 - 10,000	56	1300*	0/0	hemorrhagic/normal	
	64415/B	756/05/02/2015	#13-02 - 10,000	56	10	0/0	normal/normal	
	64375/B	768/04/28/2015	#13-02 - 10,000	56	3,000	0/0	hemorrhagic/normal	
	64380/B	120/05/01/2015	#13-02 - 10,000	56	50	0/0	normal/normal	
	64379/B	768/04/28/2015	#13-02 - 10,000	56	4680	0/0	hemorrhagic/normal	
	64388/B	863/4/30/2015	#13-02 - 10,000	56	800	0/0	hemorrhagic/normal	
#13-2/BARC	161/M	334/06/23/2013	#10-01 - 10,000	28	1250	2F/0		
	182/F	283/06/25/2013	#10-01 - 10,000	28	1000	1F/0		
	172/F	339/06/25/2013	#10-01 - 10,000	28	250	1F/1		
	170/M	339/06/25/2013	#13-01 - 10,000	28	1375	1F/0		
	7698/F	320/06/13/2013	#13-01 - 10,000	28	8000	0/0		
#13-2/BARC	190M	313/06/27/2013	#10-01 -10,000	54	0	1F, 2M/0	hemorrhagic/loose	
	615/F	334/06/23/2013	#10-01 -10,000	54	150	0/0	hemorrhagic/loose	
	221/M		#10-01 -10,000	54	1100	2F/0	normal/normal	
	222/M	312/07/14/2013	#10-01 -10,000	54	154	0/0	hemorrhagic/loose	
	20/M	312/07/14/2013	#10-01 -10,000	54	222	0/0	hemorrhagic/loose	
	221/M	312/07/14/2013	#10-01 -10,000	54	0	0/0	hemorrhagic/loose	
	7077/F	343/08/04/2013	#10-01 -10,000	54	0	0/0	lightly hemorrhagic/normal	

	7075/F	343/08/04/2013	#10-01 -10,000	54	0	0/0	normal/normal	
	7071/F	343/08/04/2013	#10-01 -10,000	54	480	0/0	normal/normal	
	7076/F	343/08/04/2013	#10-01 -10,000	54	0	0/0	hemorrhagic/loose	
#13-2/BARC	7419/F	302/11/05/2012	#12-01 - 10,000	54	107	0/0		
	418/F	302/11/05/2012	#12-01 - 10,000	54	240	0/0		
	7420/F	231/11/06/2012	#12-01 - 10,000	54	289	0/0		
	7421/M	231/11/06/2012	#12-01 - 10,000	54	500	0/0		
	7411/M	302/11/05/2012	#12-01 - 10,000	54	190	0/0		
	7427/M	231/11/06/2012	#12-01 - 10,000	54	160	0/0		
	7429/F	231/11/06/2012	#12-01 - 10,000	54	1	0/0		
	7423/F	231/11/06/2012	#12-01 - 10,000	54	147	0/0		
	7412/F	231/11/06/2012	#12-01 - 10,000	54	105	0/0		
	7410/M	302/11/05/2012	#12-01 - 10,000	54	170	0/0		
#13-2/BARC	96/F	311/01/07/2013	#12-01 - 10,000	56	0	1F/0		
	554/F	367/01/13/2013	#12-01 - 10,000	56	0	3M/0		
	546/F	345/01/13/2013	#12-01 - 10,000	56	230	0/0		
	538/F	348/01/12/2013	#12-01 - 10,000	56	187	0/0		
	7536/F	348/01/12/2013	#12-01 - 10,000	56	55	0/0		
	7541/F	345/01/13/2013	#12-01 - 10,000	56	67	0/0		
	7555/F	367/01/13/2013	#12-01 - 10,000	56	33	0/0		
	7537/F	348/01/12/2013	#12-01 - 10,000	56	240	1F/0		
#12-7/BARC	122/F	8088/03/17/2012	#07-01 - 10,000	21	2750	0/0	normal/normal	
	126/F	8088/03/17/2012	#07-01 - 10,000	21	3593	0/0	normal/normal	
	126/M	306/03/11/2012	#10-01 - 10,000	21	1530	0/0	normal/normal	
	130/F	306/03/11/2012	#10-01 - 10,000	21	3220	0/0	normal/normal	
	119/M	8088/03/17/2012	#12-01 - 10,000	21	765	0/0	normal/normal	
	112/M	690/03/16/2012	#12-01 - 10,000	21	980	0/0	normal/normal	
	113/F	690/03/16/2012	#07-02 - 10,000	21	1470	0/0	normal/normal	
	115/F	690/03/16/2012	#07-02 - 10,000	21	1530	0/0	normal/normal	
#12-7/BARC	7324/M	5070/08/12/2012	#07-02 - 10,000	28	1,666	0/0	abundant mucus and hemorrhagic	
	7320/M	5070/08/12/2012	#10-01 - 10,000	28 833*		0/0	abundant mucus and hemorrhagic	
#12-7/BARC	7364		#10-01 - 10,000	34	51	0/0	hemorrhagic/diarrhea	
	7383/M	0547/09/15/2012	#07-02 - 10,000	34	3500	0/0	hemorrhagic/diarrhea	
	7381/M	0547/09/15/2012	#07-02 - 10,000	34	2750	0/0	hemorrhagic/diarrhea	
#12-7/BARC	7321/F	5070/08/12/2012	#07-02 - 10,000	42	110	0/0	normal/soft	
	7322/F	5070/08/12/2012	#07-02 - 10,000	42	0	0/0	normal/soft	
	7327/F	5070/08/12/2012	#10-01 - 10,000	42	0	0/0	normal/soft	
#12-7/BARC	7282/M	302/05/21/2012	#12-01 - 10,000	51	570	0/0	lightlyhemorrhagic/normal	
	7255/M	5077/05/16/2012	#12-01 - 10,000	51	0	0/0	normal/normal	
	7275/M	322/05/17/2012	#12-01 - 10,000	51	32	0/0	normal/normal	
	7243/M	317/05/16/2012	#12-01 - 10,000	51	2	0/0	normal/normal	
	7306/M	182/05/23/2012	#12-01 - 10,000	51	0	0/0	normal/normal	
	7291/M	182/05/23/2012	#12-01 - 10,000	51	225	0/0	normal/normal	
	7302/M	182/05/23/2012	#12-01 - 10,000	51	255	0/0	normal/normal	
	7326/M	188/05/24/2012	#12-01 - 10,000	51	185	0/0	normal/normal	
	7324/M	188/05/24/2012	#12-01 - 10,000	51	250	0/0	normal/normal	
	7321/M	188/05/24/2012	#12-01 - 10,000	51	0	0/0	normal/normal	
#12-7/BARC	7367/F	318/09/13/2012	#12-01 - 10,000	55	0	0/0	normal/normal	
		0547/09/18/2012	#12-01 - 10,000	55	345	0/0	lightly hemorrhagic/normal	
		0547/09/18/2012	#12-01 - 10,000	55	31	0/0	hemorrhagic/diarrhea; serosal fragil	
		0547/09/18/2012	#12-01 - 10,000	55	78	0/0	hemorrhagic/diarrhea; serosal fragil	
	7389/F	0547/09/18/2012	#12-01 - 10,000	55	350	0/0	normal/loose	
		0547/09/18/2012	#12-01 - 10,000	55	0	0/0	normal/normal	
		4088/09/20/2012	#12-01 - 10,000	55	38	0/0	hemorrhagic/diarrhea; serosal fragil	
		4088/09/20/2012	#12-01 - 10,000	55	192	0/0	hemorrhagic/diarrhea; serosal fragil	
		0245/09/21/2012	#12-01 - 10,000	55	0	0/0	normal/normal	
		230/08/13/2012	#12-01 - 10,000	55	0	0/0	normal/normal	
#12-2/BARC	660/F	194/11/22/2011	#11-01 - 10,000	21	1710	0/0	lightly hemorrhagic/semi-solid	Physiology Fig. 2a; 2b
	662/M	194/11/22/2011	#11-01 - 10,000	21	3140	0/0	lightly hemorrhagic/semi-solid	Physiology Fig. 2a; 2b
	650/F	185/11/05/2011	#11-01 - 10,000	21	2200	0/0	lightly hemorrhagic/semi-solid	Physiology Fig. 2a; 2b
	673/F	182/11/24/2011	#11-01 - 10,000	21	2220	0/0	mildly pathology/loose	Physiology Fig. 2a; 2b
	651/F	185/11/05/2011	#11-01 - 10,000	21	2000	0/0	mildly pathology/loose	Physiology Fig. 2a; 2b
	658/F	185/11/05/2011	#11-01 - 10,000	21	1,300	0/0	lightly hemorrhagic/semi-solid	Physiology Fig. 2a; 2b
#12-2/BARC	7044/M	199/01/07/2012	11:01 - 10,000	35	1	0/0	normal/normal	Physiology Fig. 2a; 2b
	7070/M	1895/01/10/2012	11:01 - 10,000	35	2	0/0	normal/normal	Physiology Fig. 2a; 2b
	7091/M	5001/01/11/2012	11:01 - 10,000	35	200	0/0	normal/normal	Physiology Fig. 2a; 2b
	7031/M	6001/01/06/2012	11:01 - 10,000	35 450*		0/0	lightly hemorrhagic/loose	Physiology Fig. 2a; 2b

	7300/M	206/01/12/2012	11:01 - 10,000	35	350	0/0	lightly hemorrhagic/loose	Physiology Fig. 2a; 2b
	7050/M	316/01/10/2012	11:01 - 10,000	35	400	0/0	lightly hemorrhagic/loose	Physiology Fig. 2a; 2b
	7034/M	6001/01/06/2012	11:01 - 10,000	35	150	0/0	normal/semi-solid	Physiology Fig. 2a; 2b
	7036/M	6001/01/06/2012	11:01 - 10,000	35	4	0/0	normal/semi-solid	Physiology Fig. 2a; 2b
#12-2/BARC	7081/M	321/01/10/2012	11:01 - 10,000	42	130	0/0	lightly hemorrhagic/loose	Physiology Fig. 2a; 2b
	7023/M	1088/01/06/2012	11:01 - 10,000	42	44	0/0	normal/semi-solid	Physiology Fig. 2a; 2b
	7040/M	199/01/07/2012	11:01 - 10,000	42	0	0/0	normal/semi-solid	Physiology Fig. 2a; 2b
	7044/M	199/01/07/2012	11:01 - 10,000	42	3	0/0	normal/semi-solid	Physiology Fig. 2a; 2b
	7092/M	5001/01/11/2012	11:01 - 10,000	42	79	0/0	normal/semi-solid	Physiology Fig. 2a; 2b
	7069/M	1977/01/10/2012	11:01 - 10,000	42	52	0/0	normal/normal	Physiology Fig. 2a; 2b
	7021/M	1088/01/06/2012	11:01 - 10,000	42	37	0/0	normal/semi-solid	Physiology Fig. 2a; 2b
	7043/M	199/01/07/2012	11:01 - 10,000	42	5	0/0	normal/semi-solid	Physiology Fig. 2a; 2b
#12-2/BARC	659/F	185/11/05/2011	11:01 - 10,000	52	0	0/0	normal/normal	Physiology Fig. 2a; 2b
	695/F	1950/11/29/2011	11:01 - 10,000	52	61*	0/0	lightly hemorrhagic/semi-solid	Physiology Fig. 2a; 2b
	671/F	182/11/24/2011	11:01 - 10,000	52	0	0/0	normal/normal	Physiology Fig. 2a; 2b
	692/M	1950/11/29/2011	11:01 - 10,000	52	0	0/0	normal/normal	Physiology Fig. 2a; 2b
	55/F	185/11/05/2011	11:01 - 10,000	52	1009	0/0	mildly hemorrhagic/loose	Physiology Fig. 2a; 2b
#12-2/BARC	653/F	185/11/05/2011	11:01 - 10,000	53	291	0/0	fragile tissue/loose	Physiology Fig. 2a; 2b
	685/F	6009/11/24/2011	11:01 - 10,000	53	6	0/0	normal/normal	Physiology Fig. 2a; 2b
	682/F	6009/11/24/2011	11:01 - 10,000	53	0	0/0	normal/normal	Physiology Fig. 2a; 2b
	683/M	6009/11/24/2011	11:01 - 10,000	53	328	0/0	lightly hemorrhagic/semi-solid	Physiology Fig. 2a; 2b
	698/F	1950/11/29/2011	11:01 - 10,000	53	0	0/0	normal/normal	Physiology Fig. 2a; 2b
#10-6/BARC	31/F	1942/08/15/2010	#10-06 - 10,000	33	1158	0/0		Metabolomics; Fig. 6-11
	35/F	1942/08/15/2010	#10-06 - 10,000	33	914	0/0		Metabolomics; Fig. 6-11
	9066/F	1948/08/06/2010	#10-06 - 10,000	33	884	0/0		Metabolomics; Fig. 6-11
	9069/F	1948/08/06/2010	#10-06 - 10,000	33	1225	0/0		Metabolomics; Fig. 6-11
	30/F	1942/08/15/2010	#10-06 - 10,000	33	1275	0/0		Metabolomics; Fig. 6-11
	37/F	1942/08/15/2010	#10-06 - 10,000	33	708	0/0		Metabolomics; Fig. 6-11
	35/M	1942/08/15/2010	#10-06 - 10,000	33	1176	0/0		Metabolomics; Fig. 6-11
	9050/F	1329/08/05/2010	#10-06 - 10,000	33	1080	0/0		Metabolomics; Fig. 6-11
	9068/F	1948/08/06/2010	#10-06 - 10,000	33	1160	0/0		Metabolomics; Fig. 6-11
	9085/F	1977/08/07/2010	#10-06 - 10,000	33	956	3F;1M/1		ND
	9070/F	484/08/07/2010	#10-06 - 10,000	33	428*	0/0		Metabolomics; Fig. 6-11
	9072/F	484/08/07/2010	#10-06 - 10,000	33	940	0/0		Metabolomics; Fig. 6-11
#10-6/BARC	9074/F	484/08/07/2010	#10-01 -10,000	52	0	0/0	normal/normal	
	9071/F	484/08/07/2010	#10-01 -10,000	52	0	0/0	normal/normal	
	9081/F	1977/08/07/2010	#10-01 -10,000	52	0	1F/0	normal/normal	
	33/F	1942/08/15/2010	#10-01 -10,000	52	1089	0/0	heavy mucus; hemorrhagic/normal	Transcriptomics; Fig. 5
	39/F	1942/08/15/2010	#10-01 -10,000	52	323	0/0	heavy mucus; hemorrhagic/normal	Transcriptomics; Fig. 5
	30/F	1942/08/15/2010	#10-01 -10,000	52	0	0/0	normal/normal	Transcriptomics; Fig. 5
	9061/F	1948/08/06/2010	#10-01 -10,000	52	0	0/0	normal/normal	Transcriptomics; Fig. 5
#10-6/BARC	30/M	1942/08/15/2010	#10-01 -10,000	53	0	0/0	normal/normal	
	9057/F	1329/08/05/2010	#10-01 -10,000	53	0	0/0	normal/normal	Transcriptomics; Fig. 5
	9052/F	1329/08/05/2010	#10-01 -10,000	53	83	0/0	normal/normal	
	12/F	1907/08/10/2010	#10-01 -10,000	53	0	0/0	normal/normal	Transcriptomics; Fig. 5
	9063/F	1948/08/06/2010	#10-01 -10,000	53	0	0/0	normal/normal	Transcriptomics; Fig. 5
	9067/F	1948/08/06/2010	#10-01 -10,000	53	567	0/0	heavy mucus; hemorrhagic/normal	Transcriptomics; Fig. 5
	9/F	1437/08/10/2010	#10-01 -10,000	53	0	1F;4M/0	normal/normal	
#09-7/BARC	3057/	427/06/01/2009	#09-01/-02 - 30,000	21	4000	0/0	hemorrhagic/loose	Transcriptomics; Fig. 4
	3073/F	300/06/04/2009	#09-01/-02 - 30,000	21	1666	0/0	hemorrhagic/loose	Transcriptomics; Fig. 4
	3087/F	107/06/06/2009	#09-01/-02 - 30,000	21	4000	0/0	strongly hemorrhagic/loose/blood	
	4041/	438/06/19/2009	#09-01/-02 - 30,000	21	7000	0/0	hemorrhagic/loose	
	4062/		#09-01/-02 - 30,000	21	1000	0/0	hemorrhagic/loose	
	3094/M	466/06/09/2009	#09-01/-02 - 30,000	21	2500	0/0	hemorrhagic/loose	
	4040/M	438/06/19/2009	#09-01/-02 - 30,000	21	2833	0/0	hemorrhagic/loose	
	4044/M	438/06/19/2009	#09-01/-02 - 30,000	21	2166	0/0	hemorrhagic/loose	
	3058/	427/06/01/2009	#09-01/-02 - 30,000	21	1666	0/0	hemorrhagic/loose	Transcriptomics; Fig. 4
	3086/	107/06/06/2009	#09-01/-02 - 30,000	21	666	0/0	mildly hemorrhagic/normal	
#173/BARC	9002	20025/02/01/2002	#01-01 - 3,750	53	1880	0/0		qPCR; Fig 3
	7714	20024/1/22/2002	#01-01 - 3,750	53	1410	0/0		ND
	7713	20024/1/22/2002	#01-01 - 3,750	53	6	0/0		qPCR; Fig 3
	7712	20024/1/22/2002	#01-01 - 3,750	53	2030	0/0		qPCR; Fig 3
	8406	20023/1/18/2002	#01-01 - 3,750	53	1220	0/0		ND
	8205	20022/1/16/2002	#01-01 - 3,750	53	2	0/0		qPCR; Fig 3
	7719	20024/1/22/2002	#01-01 - 3,750	53	10	0/0		qPCR; Fig 3
	8202	20022/1/16/2002	#01-01 - 3,750	53	2000	0/0		qPCR; Fig 3
	8404	20022/1/16/2002	#01-01 - 3,750	53	1560	0/0		ND
	8107	20021/1/7/2002	#01-01 - 3,750	53	986	0/0		ND

Pig Sex - male (M); female (F); barrow (B)
*indicates worms smaller than average within the group
Ascaris adult females (F) or males (M)

Supplemental Table S3. DEGs in the poximal colon of infected over control pigs at 21 days after inoculation.

Feature ID	Fold change	EDGE test: FDR p-value correction
PLA2G2A	-1633.2	1.33E-05
OLIG2	-143.0	1.09E-03
ZFP42	-89.1	3.67E-11
DNAH14	-60.4	1.56E-02
COL22A1	-56.8	1.34E-02
ADAM1A	-52.9	1.87E-02
ASTL	-49.0	2.75E-02
FABP3L2*	-44.6	1.84E-04
ABCC4L3	-23.7	8.32E-19
LIPM	-19.0	2.62E-02
ZSCAN9	-18.9	4.68E-02
CD40	-17.7	4.10E-02
MIR1279	-16.9	4.50E-05
SLC22A10L3	-14.0	1.75E-03
HOXC9	-12.3	5.27E-03
ABCC4L4	-12.2	7.35E-07
FCER2	-12.0	1.34E-02
AGTR2	-11.5	3.94E-03
ALDOB	-10.6	8.40E-19
CCL3L2	-10.6	5.90E-03
MIR1244-1	-9.7	6.08E-03
SLC30A10	-9.4	2.71E-13
OAZ3	-8.8	2.23E-02
SLC14A1	-7.9	9.93E-11
TRPV6	-7.7	1.24E-06
HMGCS2	-7.2	0.00E+00
CD200R1L	-6.9	7.81E-03
ABCA8	-6.8	4.42E-10
PHEX	-6.8	1.81E-02
ABCA6	-6.4	3.40E-08
CXCL9	-6.1	7.08E-08
CCR9	-5.8	1.14E-02
CXCL13	-5.8	5.77E-08
COL6A6	-5.7	3.51E-08
COL25A1	-5.6	7.28E-04
AGT	-5.6	1.62E-05
S100G	-5.6	3.09E-05
CCL3L1	-5.6	2.84E-03
ABCA9	-5.5	3.06E-08
MOGAT2	-5.4	8.78E-03
AICDA	-5.3	8.16E-04
CR2	-5.2	3.00E-14
FCRL1	-5.0	1.34E-02
SLC24A4	-4.9	8.53E-03
CYP26B1	-4.8	9.76E-03

MUSK	-4.8	5.12E-05
ZNF354B	-4.7	1.05E-02
PDK4	-4.6	5.99E-03
PLIN1	-4.3	4.50E-05
CD5L	-4.2	2.41E-02
GPR174	-4.1	4.81E-08
HAS3	-4.0	4.07E-05
TNFSF11	-4.0	1.83E-03
CYP1B1	-3.9	2.93E-04
IDO1	-3.8	6.52E-05
FCRLA	-3.8	3.49E-02
NR1H4	-3.7	1.44E-02
SLC7A3L7*	-3.6	4.06E-04
CLEC17A	-3.6	4.30E-03
ABCG2	-3.5	9.73E-07
SLC25A53	-3.5	2.19E-02
NXPE2	-3.4	2.01E-03
KLRK1	-3.4	6.23E-09
ABCC4L2	-3.4	2.05E-10
MAMDC2	-3.4	3.09E-05
GHR	-3.4	2.10E-05
ART3	-3.3	3.07E-02
PNLIPRP2	-3.3	7.77E-06
ADORA3	-3.3	5.03E-03
GCNT4	-3.3	6.50E-03
AQP8	-3.3	3.30E-04
XCL1	-3.3	3.63E-03
AGTR1	-3.2	6.35E-04
ABCA13	-3.2	3.49E-02
PF4	-3.2	1.71E-02
CD28	-3.2	1.08E-02
CLCA4	-3.1	1.16E-02
FBXO32	-3.1	9.94E-05
ABI3BP	-3.0	8.43E-06
DDIT4L	-3.0	2.84E-04
EDIL3	-3.0	3.93E-07
CR1	-3.0	1.86E-02
CHRM2	-3.0	7.15E-03
LY9L1*	-3.0	1.09E-02
SNORA32	-3.0	1.95E-02
KLF12	-2.9	1.06E-03
CHP2	-2.9	1.12E-05
PAX5	-2.8	2.30E-02
RPL11L	-2.8	7.05E-03
CCL8	-2.8	4.24E-02
ASPA	-2.8	1.01E-02
NGLY1	-2.8	4.52E-07

HDAC9	-2.7	7.28E-04
IL15	-2.7	3.09E-02
AR	-2.7	4.53E-02
ADAMTS19	-2.6	1.33E-02
LIFR	-2.6	3.03E-05
RHOH	-2.6	2.84E-03
HOXD10	-2.6	1.85E-02
NEGR1	-2.6	9.36E-03
ADGRE4	-2.6	1.56E-03
CTBS	-2.5	3.96E-02
CELF2	-2.5	4.04E-06
CD1D	-2.5	2.89E-03
GPNMB	-2.5	3.04E-05
BMP3	-2.5	2.94E-03
SLC26A3	-2.5	8.05E-03
PADI2	-2.5	1.60E-04
TNFSF13B	-2.5	2.69E-04
KCNJ2	-2.5	2.94E-04
NT5E	-2.5	1.78E-04
DAPK1	-2.5	8.50E-03
PRSS12	-2.5	5.62E-03
SLA-DQB1	-2.5	1.55E-03
BTC	-2.4	1.84E-02
LYPD6	-2.4	1.56E-02
SLA-DMB	-2.4	3.61E-04
PPFIA4	-2.4	2.68E-02
C4BPAL	-2.4	3.78E-02
IPCEF1	-2.3	2.46E-02
DMD	-2.3	4.69E-05
C3AR1	-2.3	5.27E-03
KIT	-2.3	2.04E-02
PDGFC	-2.3	2.70E-03
RPL35A	-2.3	1.95E-03
CXCL12	-2.3	5.03E-03
CNTN1	-2.3	2.41E-02
CST3	-2.3	3.93E-04
GPR82	-2.2	1.35E-02
AHRR	-2.2	2.98E-02
LPXN	-2.2	1.56E-03
SLC16A1	-2.2	1.59E-03
PTPN7	-2.2	1.83E-03
EGLN3	-2.2	1.65E-03
CD4	-2.2	1.61E-02
ADIPOQ	-2.2	4.06E-03
ADAMTS5	-2.2	1.84E-02
APH1B	-2.2	3.53E-02
TMTC1	-2.2	2.39E-02

SESN3	-2.2	5.41E-04
CHL1	-2.2	1.49E-02
SMAD9	-2.2	3.79E-04
ZNF672	-2.1	1.41E-02
TLR8	-2.1	1.41E-02
PGM5	-2.1	8.60E-04
DTNA	-2.1	2.37E-02
MPEG1	-2.1	7.99E-03
SLC8A1	-2.1	2.28E-03
CD302	-2.1	6.84E-03
CD3G	-2.1	8.80E-03
SLAMF6	-2.1	4.40E-02
HPSE	-2.1	4.45E-03
MAF	-2.1	1.40E-02
RARRES1	-2.0	2.81E-03
PIK3AP1	-2.0	6.93E-03
PLXNC1	-2.0	1.18E-03
CCL2	-2.0	1.38E-03
ZNF568	-2.0	1.33E-02
FAM65B	-2.0	2.68E-02
LRIG2	-2.0	5.03E-03
SDPR	-2.0	4.88E-03
SLC22A5	-2.0	4.83E-02
TLR7	-2.0	4.66E-02
RDH10	-2.0	2.25E-02
PCMTD2	-2.0	2.03E-02
CALD1	-2.0	5.64E-03
CYP4V2	-2.0	1.52E-02
COL6A5	-2.0	1.50E-02
SLC4A7	-2.0	2.41E-02
NPL	-1.9	2.41E-02
MAFB	-1.9	5.99E-03
ABCA1	-1.9	5.62E-03
C5	-1.9	1.14E-02
NFATC2	-1.9	4.03E-02
CD48	-1.9	1.09E-02
LRP12	-1.9	2.33E-02
ALDH6A1	-1.9	5.11E-03
SLC25A3	-1.9	2.19E-02
FGL2	-1.9	2.08E-02
DOCK10	-1.9	1.58E-02
SLA-DMA	-1.9	1.99E-02
NREP	-1.9	1.71E-02
SATB1	-1.9	1.64E-02
CFL2	-1.9	2.19E-02
CD2	-1.8	9.24E-03
BANK1	-1.8	4.81E-02

LY9	-1.8	4.79E-02
ERAP1	-1.8	3.18E-02
PPAP2B	-1.8	1.44E-02
RBMS1	-1.8	1.68E-02
FRZB	-1.8	3.93E-02
DIXDC1	-1.8	2.08E-02
IGJ	-1.8	1.92E-02
AHCYL2	-1.8	2.15E-02
IGSF5	-1.8	1.18E-02
SLA-3	-1.8	3.72E-02
ITGAE	-1.8	4.27E-02
PTCH1	-1.8	3.72E-02
ITGA4	-1.7	4.03E-02
SCARA5	-1.7	3.55E-02
CDC42EP3	-1.7	4.01E-02
PAG1	-1.7	4.72E-02
SYNE1	-1.7	3.52E-02
KCTD12	-1.7	4.66E-02
CRLF3	-1.7	4.24E-02
COL14A1	-1.7	3.72E-02
MPDZ	-1.7	3.53E-02
CYP7B1	-1.7	4.83E-02
CD109	-1.7	4.15E-02
TSPAN7	-1.7	4.78E-02
SEC11C	1.7	4.83E-02
MIF	1.7	4.03E-02
PLA2G16	1.8	4.03E-02
GLUL	1.8	4.41E-02
SLC35C1	1.8	4.89E-02
TARS	1.8	3.43E-02
MAZ	1.8	3.53E-02
S100A14	1.8	2.70E-02
SMARCE1	1.8	1.98E-02
SERPINB8	1.8	2.66E-02
FOXA1	1.8	3.01E-02
PIK3C2A	1.8	2.22E-02
PMPCA	1.8	3.50E-02
WDR1	1.9	4.04E-02
MIR1282	1.9	2.53E-02
CFL1	1.9	4.46E-02
IGFBP5	1.9	2.01E-02
MCF2L	1.9	1.98E-02
PRTFDC1	1.9	1.86E-02
NDUFA6	1.9	1.39E-02
DDX54	1.9	4.32E-02
MRPL51	1.9	1.72E-02
LSS	1.9	3.03E-02

LRRC59	1.9	3.60E-02
MKI67	1.9	1.58E-02
SUMO4	1.9	3.77E-02
STARD10	1.9	1.91E-02
BOLA2	1.9	2.95E-02
PSMB7	1.9	7.05E-03
SLC39A7	1.9	9.92E-03
MAGOH	1.9	1.71E-02
PTN	1.9	1.03E-02
CNOT3	2.0	3.50E-02
CENPJ	2.0	4.45E-02
SKA2	2.0	9.87E-03
ATOH1	2.0	2.41E-02
HSD11B1	2.0	2.19E-02
SMPDL3B	2.0	1.67E-02
FDPS	2.0	3.53E-02
SERPINB6	2.0	1.67E-02
TIMM17A	2.0	1.40E-02
SLC25A1	2.0	4.03E-02
CXCL16	2.0	2.89E-03
LRRC8A	2.0	1.64E-02
SEMA7A	2.1	2.41E-02
PSTPIP2	2.1	5.66E-03
GADD45B	2.1	4.75E-02
SMAGP	2.1	4.78E-02
ARHGDIA	2.1	1.87E-02
HDLBP	2.1	1.81E-02
UAP1L1	2.1	1.98E-02
ERN2	2.1	1.54E-02
DPP9	2.1	5.03E-03
TGM2	2.2	7.15E-03
SERPINB5	2.2	1.56E-03
PYCRL	2.2	4.03E-02
BCL3	2.2	2.84E-03
PKM	2.2	2.62E-02
DPRX	2.2	1.71E-02
TRAPPC6A	2.2	4.81E-02
TUBA1C	2.2	3.01E-02
TMEM54	2.2	1.11E-02
CDH1	2.2	3.28E-03
AREG	2.2	8.37E-03
ROS1	2.2	8.87E-04
RIPK3	2.3	1.33E-02
UBE2S	2.3	2.19E-02
CCNB2	2.3	3.45E-04
ADAMTS1	2.3	4.06E-04
B3GNT6	2.3	7.21E-03

ABCA12	2.3	4.40E-02
CABIN1	2.3	1.96E-03
FFAR4	2.3	2.68E-02
MYBL2	2.4	3.34E-03
P2RX4	2.4	8.14E-04
CYP2B22	2.4	4.65E-02
ND1	2.4	1.59E-03
LIPH	2.4	1.88E-04
TACC3	2.4	6.84E-03
DEPDC7	2.4	3.67E-02
GCNT3	2.4	4.06E-04
IL1A	2.4	1.55E-03
HMGA1	2.4	5.03E-03
FAM20C	2.4	2.79E-02
BGN	2.5	1.81E-02
HSPA1L	2.5	4.83E-02
PFKFB3	2.5	7.15E-03
GALNT6	2.5	5.55E-03
TNFRSF26	2.5	2.44E-02
NT5DC2	2.5	2.37E-02
CSRP2	2.5	1.20E-02
PTPRN2	2.5	3.72E-02
BIRC5	2.6	1.37E-03
KCNE3	2.6	3.51E-05
FGF7	2.6	5.67E-04
TUBB3	2.6	2.11E-03
ITGB6	2.6	7.63E-03
GSTA1	2.7	4.73E-02
CDX1	2.7	1.73E-03
PNP	2.7	1.83E-03
KCNJ11	2.7	3.31E-02
TPSB2	2.8	2.68E-02
RRBP1	2.8	8.06E-05
PHLDA2	2.8	2.79E-03
MDK	2.8	4.44E-04
FCN2	2.9	9.94E-05
CAPNS2	2.9	2.12E-03
PLA2G3	2.9	4.90E-03
SPDEF	3.0	1.13E-04
BPIFB2	3.0	3.18E-02
LAMB3	3.0	6.87E-04
ME1	3.1	9.19E-03
FUT2	3.1	1.38E-03
F3	3.1	4.09E-05
ETV4	3.1	4.03E-02
ABAT	3.2	1.73E-05
SLC7A5	3.3	1.30E-03

FABP3	3.3	3.51E-08
HK2	3.3	1.33E-05
STEAP4	3.4	5.33E-03
CLDN8	3.5	1.41E-02
CLCA1	3.5	9.80E-09
PRKCG	3.5	4.38E-02
SERPINB10	3.5	2.87E-04
DIO2	3.5	1.15E-03
DNAH8	3.7	3.59E-02
IGFBP4	3.7	6.25E-07
TNFSF18	3.8	3.17E-02
NRG1	3.8	2.41E-02
TIMP1	3.9	2.94E-04
LOC396756	4.0	1.51E-06
PTGS2	4.0	1.30E-07
TNFRSF8	4.0	2.04E-02
SLC9C1	4.1	7.81E-04
CHI3L2	4.2	4.97E-04
GNMT	4.3	2.10E-02
AKR1C4	4.3	2.30E-05
SLC7A11	4.3	6.75E-07
TLE6	4.4	1.34E-02
CXCL14	4.4	2.73E-06
PDZK1IP1L	4.5	1.44E-02
CXCL17	4.5	7.44E-06
TNIP3	4.5	5.90E-06
BAALC	4.5	3.96E-03
BDKRB1	4.6	1.70E-02
PI3	4.6	2.55E-08
KRT18	4.7	7.48E-10
SAPCD2	4.7	1.19E-03
PLAUR	4.8	6.11E-06
SLPI	4.8	2.22E-11
COL28A1	4.9	5.31E-06
MMP3	4.9	3.31E-10
PHGDH	5.0	9.01E-04
IL17C	5.0	7.15E-03
SLC5A8	5.0	1.13E-04
GSTA2	5.4	4.30E-03
BTN1A1L2*	5.4	2.54E-02
CA4	5.5	9.51E-10
CBS	5.5	7.18E-05
AGR2	5.6	1.73E-11
SDS	5.7	8.06E-05
MIR6087	6.0	1.14E-02
IL1R2	6.1	1.51E-06
MUC2	6.3	2.16E-16

CHI3L1	6.4	1.03E-13
B3GNT7	6.4	1.16E-05
UPK1B	6.5	1.59E-03
TAT	6.9	3.49E-02
GSTP1L2*	6.9	3.53E-02
RNF39	7.0	1.51E-06
IL36A	7.2	2.58E-03
MMP1	7.3	6.53E-13
TCN1	8.0	1.44E-10
SPINK4	8.1	9.43E-22
S100A8	8.2	1.02E-02
MPTX	8.3	4.03E-15
SERPINB11	8.4	7.38E-04
SLCO1A2	8.6	6.00E-04
SP6	9.3	1.01E-02
HCAR2	9.3	6.76E-05
MMP9	9.3	1.00E-02
ADAM30	9.6	8.58E-03
MMP12	9.7	4.45E-16
S100A9	9.8	1.83E-03
TGM3	10.0	3.03E-05
S100A2	10.0	3.03E-27
REG4	11.1	8.84E-20
IGHG1	11.1	3.80E-06
HTR1B	11.8	2.40E-02
GYS2	11.9	5.62E-03
S100A12	12.8	4.09E-05
PDZK1IP1*	13.6	1.24E-05
AKR1C1	13.8	3.93E-07
GABRP	15.7	1.59E-03
A2ML1	16.0	2.14E-03
SERPINB2	16.2	4.90E-32
SLC6A2	18.7	1.53E-02
PSAT1	20.6	4.69E-11
IL11	21.6	6.84E-03
TFF1	25.9	2.62E-06
MMP13	27.5	9.71E-29
ARG1	30.1	2.28E-07
IL36B	30.8	1.02E-02
ANXA8	34.8	6.97E-40
TFF2	37.1	6.93E-10
MMP7	53.1	4.09E-05
IFITM1L3*	63.2	1.59E-02
HOXB13	132.7	1.59E-02
SLC7A3L11*	188.2	1.59E-03
IL4I1L	437.7	1.59E-14

Supplemental Table S4. Comparison of pigs at 52 days after inoculation with no worms compared to control uninfected pigs

Feature ID	FC Naive vs Infection No Worms	EDGE test: FDR p-value correction
LCN15	-32.1	3.66E-07
ALPI	-4.9	5.20E-03
CD5L	-4.7	6.80E-03
PON1	-4.5	4.13E-02
SLC10A2	-4.3	4.13E-02
SDR9C7	-4.2	2.26E-02
CYP2B22	-3.9	2.23E-02
ACSL6	-3.7	9.52E-03
GSTA2	-3.6	5.23E-02
GDF5	-3.3	1.09E-03
SCNN1B	-3.3	2.26E-02
ALPIL1	-3.1	3.58E-02
GAL	-3.1	1.57E-02
PCK1	-3.1	1.86E-02
PDK4	-3.0	3.61E-02
NCR1	-2.9	2.48E-04
SLC24A1	-2.9	1.16E-03
RDH16	-2.8	4.89E-02
ACE2	-2.6	4.67E-02
SLC38A4	-2.6	5.46E-04
VGF	-2.6	2.96E-03
ANPEP	-2.5	4.13E-02
GPIHBP1	-2.5	3.06E-02
NCR2	-2.5	4.13E-02
TREM2	-2.5	4.89E-02
GPR162	-2.3	3.71E-02
NKPD1	-2.3	2.48E-03
NR1H4	-2.3	2.51E-02
EOMES	-2.2	3.65E-02
THNSL2	-2.2	2.04E-04
CIRBP	-2.1	6.57E-05
KLRD1L*	-2.1	2.36E-02
ANGPTL4	-2.0	4.70E-02
CA12	-2.0	3.03E-03
KLF11	-2.0	4.37E-02
SIRPB2	-2.0	3.09E-02
GSTM1	-1.9	9.10E-03
SMIM6	-1.9	4.13E-02
GSTM4	-1.8	5.07E-03
GSTT1	-1.8	4.13E-02
PYCARD	-1.8	1.54E-02
SLC24A4	-1.8	4.67E-02
SLC46A3	-1.8	2.05E-03
BDH1	-1.7	5.79E-03
CD8A	-1.7	5.31E-02
CES1B*	-1.7	7.07E-03
CLEC3B	-1.7	7.15E-03
H1F0	-1.7	6.99E-03
KCNE3	-1.7	1.16E-03
METTL7A	-1.7	3.83E-03
SLC16A14	-1.7	1.09E-02
THAP8	-1.7	4.86E-02
A1CF	-1.6	1.73E-02
ADGRE4	-1.6	2.26E-02
C1QB	-1.6	5.22E-02
GSTZ1	-1.6	1.03E-02
PDK2	-1.6	1.50E-02
PLCD1	-1.6	1.46E-02

PNPLA2	-1.6	1.01E-02
SLC43A2	-1.6	2.36E-02
TMEM140	-1.6	1.73E-02
UGT2C1*	-1.6	4.13E-02
COQ8A	-1.5	1.73E-02
ARRDC3	-1.5	4.39E-02
FDXR	-1.5	2.76E-02
HADHA	-1.5	1.16E-03
ODC1	-1.5	1.27E-02
SULT1B1	-1.5	3.48E-02
TSPAN7	-1.5	3.87E-02
SNX19	1.5	3.48E-02
RASA2	1.5	8.65E-03
SKIL	1.5	1.47E-02
SLC12A4	1.5	1.59E-03
EPAS1	1.5	1.29E-02
HSPA2	1.6	5.31E-02
COL12A1	1.6	4.13E-02
PRDM2	1.6	7.04E-03
GJA1	1.7	4.40E-02
APOBR	1.7	3.48E-02
SAMSN1	1.7	1.85E-02
PIK3C2A	1.7	5.31E-02
CD33	1.7	2.79E-02
AOAH	1.7	2.02E-02
PTGS1	1.7	6.80E-03
CDR2	1.7	1.47E-02
ANKRD12	1.7	2.76E-02
HYOU1	1.7	1.73E-02
NFIL3	1.7	1.50E-04
SERPINH1	1.8	4.70E-02
GRAP	1.8	2.00E-02
SLC26A2	1.8	5.24E-02
CHORDC1	1.8	8.64E-03
GPR68	1.8	6.99E-03
LPCAT2	1.9	2.33E-03
GPR65	1.9	3.09E-02
HSPA5	1.9	2.26E-02
ALOX5AP	2.0	3.09E-02
NOV	2.0	3.48E-02
TNFRSF12A	2.0	2.26E-02
DOCK2	2.0	7.80E-03
GATA2	2.0	5.31E-02
COL8A1	2.0	3.85E-02
SFRP1	2.1	2.80E-03
DNAJA1	2.1	5.20E-03
SLCO4A1	2.1	1.73E-02
F13A1	2.1	7.07E-03
CD69	2.1	4.85E-03
SRGN	2.1	1.18E-05
ARNTL2	2.1	3.38E-02
GFI1	2.2	1.03E-02
RUNX3	2.2	5.12E-03
IGHE	2.3	4.86E-02
KIT	2.3	1.73E-02
IPCEF1	2.4	1.03E-02
SLC16A3	2.4	2.50E-03
CD244	2.4	1.29E-02
NLRC3	2.5	1.97E-02
SELE	2.5	3.53E-02

MAP4K1	2.5	2.73E-04
GZMAL	2.5	1.01E-02
TDRP1	2.6	5.04E-03
HSPA1L	2.6	5.31E-02
SLC2A3	2.7	1.71E-10
HSPH1	2.7	1.29E-02
HDC	2.7	2.39E-02
GPR97	2.8	2.26E-02
FGR	2.8	6.51E-07
LTC4S	2.8	1.24E-02
TIGIT	2.8	3.39E-04
CSF2RAL	2.9	4.86E-02
GZMA	3.0	4.54E-04
CXCL6	3.1	1.01E-02
MMP9	3.4	3.03E-03
SH2D1B	3.5	4.91E-04
FFAR2	3.6	1.18E-05
TIAM1	4.4	6.67E-08
GATA3	4.4	4.43E-04
IGFBP2	4.7	1.99E-08
SLC5A8	4.8	3.38E-02
GPRC5D	4.8	9.86E-03
DFNB31	4.9	7.93E-05
DIO2	5.8	1.16E-03
NXPH4	5.8	2.70E-06
TKTL1	6.4	1.49E-02
SLC4A11	6.9	1.04E-03
CTSG	7.2	8.45E-07
MMP12	7.3	1.09E-03
KLRJ1*	9.6	1.59E-03
SNORA73AL	10.4	2.01E-02
NTRK1	10.6	3.53E-03
GZMB	11.2	8.54E-08
TCN1	19.2	1.29E-02
TNN	83.7	5.30E-05
GNLY	132.0	2.98E-11
REG3A	168.9	2.26E-02

Supplemental Table S5. Comparison of DEGs expressed in the proximal colon of pigs at 52 days after inoculation with worms compared to uninfected controls.

Gene	Fold change	EDGE FDR p-value
SLC10A2	-1285.8	8.30E-08
ALPI	-159.7	2.09E-10
EVX1	-38.1	2.47E-02
ASTL	-37.2	2.34E-02
CLCA4	-27.9	1.34E-07
ALDOB	-27.7	2.35E-07
GSTA2	-26.6	8.94E-06
CYP2B22	-26.4	2.38E-06
BTNL2	-21.7	9.02E-03
HMGCS2	-21.2	0.00E+00
NXPE2	-20.1	2.40E-06
NR1H4	-17.1	2.31E-11
PCK1	-16.9	1.62E-07
FRMD1	-15.5	9.59E-05
OASL	-14.4	5.68E-03
SLC14A1	-13.9	1.46E-02
FOXO6	-13.7	8.20E-06
AQP8	-13.7	2.47E-03
SLC26A3	-12.7	8.30E-08
NCR2	-12.1	1.81E-05
CD5L	-10.9	8.19E-04
THAP12	-10.5	4.27E-02
ALPIL1*	-10.3	1.67E-04
SLC30A10	-10.2	3.00E-03
SDR9C7	-9.3	8.99E-03
MOGAT2	-9.2	1.14E-03
AGT	-8.7	1.64E-05
PADI2	-8.6	1.42E-09
GRIK1	-8.6	4.27E-02
HAS3	-8.4	9.20E-06
SULT1E1	-8.2	1.08E-09
SI	-7.9	8.57E-03
SLC25A34	-7.8	1.41E-09
COL6A6	-7.8	1.07E-07
SLC38A4	-7.7	3.14E-09
PDK4	-7.7	2.11E-04
LCN15	-7.6	4.90E-02
ABCG2	-7.6	6.40E-07
CXCL9	-7.5	6.02E-04
CEACAM8	-7.5	1.20E-02
NCR1	-7.2	1.19E-06
SLC51A	-6.9	2.76E-08
P2RX3	-6.5	3.20E-05
ABCA6	-6.5	3.05E-06
GUCA2A	-6.3	2.26E-05
TM6SF2	-6.3	2.26E-14
CA12	-6.2	4.33E-13
DNAI1	-6.1	1.42E-02
RDH16	-6.1	3.20E-03
TRPV6	-6.0	4.34E-02
IFI27	-5.8	7.91E-04
SSTR5	-5.7	4.20E-03
SLC16A1	-5.7	0.00E+00
CA1	-5.6	4.33E-12
CCL3L2	-5.4	6.08E-03
GABRB2	-5.4	4.12E-06
GPIHBP1	-5.3	6.94E-04
ANPEP	-5.2	3.11E-04
XCL1	-5.2	1.96E-06
CYP26B1	-5.0	2.61E-08
IGF2-AS	-5.0	1.71E-02
OPRL1	-4.9	3.95E-04
PIPOX	-4.9	6.45E-03
EOMES	-4.8	1.12E-04
FOXJ1	-4.8	4.62E-03
CXCL11	-4.8	3.33E-02
IL12B	-4.7	1.02E-02
CLEC4F	-4.6	4.26E-02
DACT2	-4.6	4.67E-02
EDN3	-4.6	7.22E-03
SLC24A4	-4.4	2.68E-06
RSAD2	-4.4	2.06E-02

SELENBP1	-4.4	1.11E-11
ADIPOQ	-4.2	5.00E-04
CD207	-4.2	4.37E-02
KLF11	-4.2	3.47E-05
CHP2	-4.1	1.38E-07
UGT2C1*	-4.1	8.74E-11
DLX4	-4.1	2.69E-03
ABCA8	-4.1	3.01E-03
CLDN10	-4.1	3.30E-03
PLIN1	-4.0	3.06E-02
PARP15	-4.0	3.90E-04
GZMK	-4.0	1.31E-02
SCNN1B	-3.8	3.19E-02
GPR162	-3.8	1.31E-03
WNT8B	-3.8	2.30E-04
DLX3	-3.8	2.11E-02
GDF5	-3.8	1.25E-02
FOXS1	-3.8	3.26E-05
FABP1	-3.7	5.00E-05
KLRD1L	-3.7	1.90E-04
HSD17B6	-3.7	1.19E-02
CD36	-3.7	8.88E-09
CIDEA	-3.6	1.99E-03
CTBS	-3.6	8.38E-03
SLC25A42	-3.6	1.96E-06
RNF112	-3.5	1.01E-03
VIP	-3.5	5.68E-03
CNTFR	-3.5	4.40E-04
IRF4L	-3.4	5.23E-03
SIGLEC15	-3.4	5.06E-02
CXCL10	-3.4	3.22E-02
SECTM1	-3.4	1.23E-03
ZBP1	-3.4	5.56E-04
DHX58	-3.3	7.59E-03
SELENOP	-3.3	2.97E-08
ISX	-3.3	2.28E-03
IGF2	-3.2	5.66E-04
PRSS12	-3.2	1.43E-04
XAF1	-3.2	5.31E-02
ABI3BP	-3.2	4.50E-05
SLC30A3	-3.1	8.38E-03
LYPD6	-3.1	7.55E-03
CLCA4L	-3.1	1.92E-02
SLC7A8	-3.1	2.76E-05
NECAB2	-3.1	1.88E-02
GSTM1	-3.0	2.39E-05
AOC1	-3.0	3.91E-07
ABCA9	-3.0	3.15E-02
GAL	-3.0	4.97E-02
CCR9	-3.0	5.47E-02
ASPA	-2.9	4.20E-03
ANGPTL4	-2.9	1.19E-03
GSTM4	-2.9	3.46E-06
IFI44	-2.9	1.80E-02
PRDX6	-2.9	1.45E-07
SLC35D3	-2.9	3.46E-02
BDH1	-2.9	4.31E-08
TMPRSS13	-2.8	1.67E-02
CARD14	-2.8	6.03E-05
AHRR	-2.8	3.02E-04
FBXO32	-2.8	1.02E-04
SIRPB2	-2.8	6.49E-03
CD209	-2.8	1.42E-03
SLC46A3	-2.8	2.40E-06
ACSBG1	-2.8	4.00E-02
SLC7A3L6	-2.8	1.70E-02
CD4	-2.8	1.41E-04
CXCL12	-2.7	6.31E-04
NMRAL1L*	-2.7	1.81E-02
FAAH	-2.7	6.61E-04
UGT1A3	-2.7	2.57E-02
CA11	-2.7	7.55E-03
FABP4	-2.7	1.86E-02
NGB	-2.7	3.12E-02

ZFP84L	-2.7	3.53E-03
ABCB4	-2.7	2.10E-02
LRP1B	-2.7	1.12E-02
MAOA	-2.7	1.11E-07
LILRB3L4	-2.7	2.84E-02
MAMDC2	-2.7	2.50E-03
WNT11	-2.7	1.51E-02
GRIK4	-2.7	1.76E-02
SLC8A2	-2.7	2.11E-02
PTPRD	-2.6	1.41E-04
ACE	-2.6	7.25E-05
SLC7A3L4	-2.6	4.44E-03
TXNIP	-2.6	5.39E-05
GBP6	-2.6	5.25E-03
GSTT1	-2.6	1.25E-03
AURKC	-2.6	3.68E-03
UBE2L6	-2.6	1.15E-04
P2RY13	-2.6	6.61E-04
ADGRB1	-2.6	1.64E-02
IFITM1	-2.6	1.45E-02
C1QB	-2.6	4.13E-05
C11orf86	-2.5	4.92E-04
SULT1C4	-2.5	1.40E-04
CD74	-2.5	2.92E-04
ZKSCAN2	-2.5	4.12E-02
KLRC1	-2.5	2.35E-02
CIRBP	-2.5	3.91E-05
IL34	-2.5	3.63E-04
ADGRE4	-2.4	9.29E-06
GBP2	-2.4	1.02E-02
AHCYL2	-2.4	5.80E-06
SLC7A3L7	-2.4	3.11E-02
SCARA5	-2.4	2.08E-02
DBP	-2.4	3.71E-03
PLCD1	-2.4	1.77E-06
GBP4	-2.4	4.83E-02
COL4A3	-2.4	4.44E-02
SLA-DRA	-2.4	1.06E-03
ADGB	-2.4	3.63E-02
TMPRSS2	-2.4	1.23E-04
SLA-DOA	-2.4	3.71E-03
TMEM140	-2.4	2.01E-06
CCL4	-2.4	1.16E-02
APOL2	-2.4	1.75E-04
RBP5	-2.4	5.79E-03
PNPLA2	-2.4	1.05E-06
ANK3	-2.4	8.67E-03
TLR8	-2.3	1.46E-03
C1QA	-2.3	4.91E-05
ANKH	-2.3	7.37E-03
C1QC	-2.3	5.39E-05
SLC39A5	-2.3	3.63E-02
IL12RB2	-2.3	4.34E-02
TUBAL3	-2.3	3.93E-02
CD83	-2.3	1.40E-02
IHH	-2.3	3.41E-05
THNSL2	-2.3	1.15E-03
HAPLN4	-2.3	1.17E-04
SLA-3	-2.3	1.89E-04
ADRB1	-2.3	1.40E-03
VGF	-2.3	3.28E-02
IFITM1L2*	-2.3	6.10E-05
SLC46A2	-2.3	6.48E-03
HOXD10	-2.3	8.74E-03
IFI44L	-2.3	3.75E-03
SLA-11	-2.3	1.08E-04
PCSK6	-2.3	1.07E-05
CD300H	-2.3	1.10E-05
LOC106509843	-2.3	1.93E-02
SLA-DQA1	-2.3	3.27E-03
SLA-DQB1	-2.3	1.13E-02
CD36L1	-2.3	2.24E-03
ABCC6	-2.2	4.92E-02
MAF	-2.2	1.08E-04

RETSAT	-2.2	4.10E-06
FCGR3A	-2.2	8.60E-05
LGALS9	-2.2	2.92E-04
SLA-DMB	-2.2	8.10E-04
CD8A	-2.2	4.54E-03
ZNF419	-2.2	3.82E-02
CD300C	-2.2	5.26E-03
LTBP4	-2.2	9.11E-05
CD27	-2.2	4.87E-02
PTPRO	-2.2	3.59E-02
EDIL3	-2.2	2.93E-03
CD7	-2.2	4.61E-02
ABCB1	-2.2	2.05E-03
PARP9	-2.2	1.66E-04
SLA-DRB1	-2.2	5.60E-03
PTCH1	-2.2	1.80E-03
EFNA3	-2.2	2.20E-02
TSPAN7	-2.2	5.07E-06
TST	-2.2	3.80E-05
TLR1	-2.2	8.67E-04
CXCR3	-2.2	1.38E-02
CYP7B1	-2.2	9.83E-03
COQ8A	-2.2	9.79E-06
MPEG1	-2.2	4.93E-03
KLHL7	-2.1	2.73E-04
NT5E	-2.1	1.30E-02
CES1B	-2.1	5.66E-04
P2RY12	-2.1	5.25E-02
ACSF2	-2.1	6.81E-06
NKPD1	-2.1	4.23E-02
LY9L1*	-2.1	4.13E-03
CYP2C91	-2.1	5.29E-02
CDKN2B	-2.1	1.87E-04
H1F0	-2.1	7.02E-04
LOC110255453	-2.1	4.34E-02
FOXH1	-2.1	3.28E-02
ADAMTSL5	-2.1	3.76E-02
CST3	-2.1	1.99E-03
ABCB6	-2.1	2.65E-05
SLC16A5	-2.1	1.59E-03
PARP14	-2.1	1.16E-02
LTB4R2	-2.1	3.08E-02
CASP1	-2.1	9.13E-04
LIFR	-2.1	4.05E-04
SLC25A20	-2.1	1.13E-04
SLC25A27	-2.1	1.55E-05
METTL7A	-2.1	1.05E-04
SLA-DMA	-2.1	4.61E-03
CTSD	-2.1	3.64E-04
ZNF548	-2.1	1.71E-02
TMPRSS4	-2.1	4.56E-05
PLCB2	-2.0	1.74E-03
MLYCD	-2.0	1.50E-03
CECR1	-2.0	4.20E-03
CSMD1	-2.0	3.76E-02
AMT	-2.0	3.33E-04
GFRA1	-2.0	4.61E-02
CPT1A	-2.0	2.94E-03
KLRG1	-2.0	5.07E-02
WNT2B	-2.0	3.42E-03
PFKFB4	-2.0	4.11E-05
COBL	-2.0	2.96E-06
EXOC3L4	-2.0	4.81E-04
PKNOX2	-2.0	2.25E-02
MMP11	-2.0	7.40E-04
LAIR1	-2.0	7.37E-03
TLR5	-2.0	4.03E-02
CDH23	-2.0	4.71E-02
B2M	-2.0	5.66E-04
MDK	-2.0	1.75E-02
ABCC4L2	-2.0	8.20E-03
UGT1A1	-2.0	1.54E-02
NADK	-2.0	5.86E-05
TLR6	-2.0	4.77E-03

EBI3	-2.0	4.52E-02
FBXW10	-2.0	2.07E-02
GBP1	-1.9	5.21E-02
SLC9A2	-1.9	1.78E-04
LGALS3BP	-1.9	3.18E-02
TSPAN11	-1.9	4.35E-03
SLC25A38	-1.9	4.57E-04
RGS10	-1.9	2.55E-02
OSBPL10	-1.9	6.89E-03
ZNF664	-1.9	6.81E-05
TAP1	-1.9	5.73E-03
CSTA	-1.9	2.56E-02
PYCARD	-1.9	8.72E-03
NCAM1	-1.9	3.06E-02
TCF7L1	-1.9	2.24E-02
BSG	-1.9	1.92E-04
ADHFE1	-1.9	4.54E-02
PIK3IP1	-1.9	1.62E-03
PGF	-1.9	4.93E-02
THAP8	-1.9	4.60E-02
ITIH5	-1.9	1.46E-02
IL10RA	-1.9	8.53E-04
CLEC3B	-1.9	2.18E-03
GLI2	-1.9	3.33E-02
ERBB2	-1.9	1.33E-03
SDPR	-1.9	1.59E-02
MT-CO3	-1.9	4.64E-03
SPIDR	-1.9	2.85E-04
OGDH	-1.9	8.27E-05
NOXO1	-1.9	1.71E-03
PDK2	-1.9	1.11E-03
TRANK1	-1.9	1.91E-02
TEF	-1.9	3.52E-03
SLC25A29	-1.9	1.01E-02
GIMAP8	-1.9	1.74E-03
CD302	-1.9	9.28E-03
AKR1E2	-1.9	3.20E-03
C7	-1.9	3.94E-02
ABCC4L5	-1.8	2.09E-02
SLC31A2	-1.8	2.06E-02
USH1C	-1.8	4.24E-04
CTSZ	-1.8	4.19E-03
SYNPO	-1.8	7.65E-03
MMP24	-1.8	2.06E-02
PSMB9	-1.8	9.65E-03
FGFRL1	-1.8	8.75E-03
MAP2K6	-1.8	3.77E-02
SLC45A4	-1.8	1.06E-03
GRINA	-1.8	1.50E-03
SLC43A2	-1.8	2.00E-03
RGL1	-1.8	1.84E-03
DAPK1	-1.8	2.09E-02
MEIS2	-1.8	4.81E-02
CD300LF	-1.8	5.14E-02
MTCP1	-1.8	4.83E-02
RNF213	-1.8	4.61E-02
ZNF709L5*	-1.8	4.65E-02
UCP2	-1.8	7.55E-03
SNX4	-1.8	7.26E-04
DTX3L	-1.8	7.69E-03
PHACTR1	-1.8	6.57E-03
HSD17B1	-1.8	4.04E-02
CLCN2	-1.8	4.79E-05
CCR5	-1.8	5.37E-03
CYP27A1	-1.8	3.99E-02
PAX8	-1.8	3.67E-02
MOCS1	-1.8	6.89E-03
CADM1	-1.8	3.02E-02
TRPV3	-1.8	1.05E-02
ADA	-1.8	1.88E-02
RALGDS	-1.8	2.82E-03
TNFAIP2	-1.8	2.30E-04
TESK2	-1.8	5.66E-03
ASAP3	-1.8	3.44E-03

SULT1B1	-1.8	1.20E-03
PSMB10	-1.8	3.20E-03
SOWAHA	-1.8	2.63E-03
SLC25A10	-1.8	2.22E-04
MMP25	-1.8	1.15E-02
IRF9	-1.8	5.24E-03
CD72	-1.8	4.52E-02
EGFLAM	-1.8	2.10E-02
VAMP2	-1.8	1.77E-03
GABBR1	-1.8	2.17E-03
FLT3LG	-1.8	3.71E-03
ADCY9	-1.8	1.41E-04
MTHFR	-1.8	7.98E-03
GPR34	-1.8	2.92E-02
GSTP1	-1.8	7.59E-03
TCF21	-1.8	4.67E-03
SLC7A3L2	-1.8	4.24E-04
SCIN	-1.8	3.67E-02
GABARAPL1	-1.8	6.54E-04
ADCY5	-1.8	4.38E-02
ACY1	-1.7	9.49E-03
PRAG1	-1.7	1.02E-02
ALDH6A1	-1.7	1.15E-02
DOCK6	-1.7	4.13E-03
MT-ATP6	-1.7	1.36E-02
TSKU	-1.7	3.98E-02
HADHA	-1.7	1.84E-04
ENO2	-1.7	3.86E-02
NPL	-1.7	2.11E-02
HSDL2	-1.7	7.04E-04
JAML	-1.7	2.72E-02
PLTP	-1.7	1.75E-02
MISP	-1.7	2.06E-03
UGT1A10	-1.7	6.00E-04
SLC7A3L8	-1.7	1.79E-02
MOV10	-1.7	1.22E-03
MERTK	-1.7	7.59E-03
N4BP2L1	-1.7	5.30E-02
AXIN2	-1.7	3.42E-02
TICAM1	-1.7	1.41E-03
CSF1R	-1.7	3.70E-03
CD68L1	-1.7	1.16E-02
MT-CO1	-1.7	7.42E-03
SLC27A1	-1.7	3.17E-02
PLA2G6	-1.7	9.77E-03
LCP2	-1.7	7.46E-03
CCL21	-1.7	4.86E-02
VDR	-1.7	7.90E-03
ACADS	-1.7	2.96E-03
APOE	-1.7	3.18E-02
LIPE	-1.7	3.48E-02
PARP3	-1.7	3.15E-02
ZNF362	-1.7	7.88E-03
MICALCL	-1.7	2.78E-02
DTX3	-1.7	4.85E-03
RFTN2	-1.7	1.70E-02
CRIP1	-1.7	2.87E-02
TSPAN4	-1.7	3.42E-02
NREP	-1.7	4.99E-02
NADSYN1	-1.7	7.62E-03
HPSE	-1.6	1.75E-02
LRIG2	-1.6	5.77E-03
DISP1	-1.6	4.68E-03
ATG2A	-1.6	4.13E-03
SPPL2B	-1.6	7.40E-04
ETHE1	-1.6	1.38E-03
ERAP2	-1.6	4.60E-02
DECR1	-1.6	1.78E-03
PSMB8	-1.6	2.91E-02
PLA2G4B	-1.6	3.68E-02
ACADSB	-1.6	1.66E-03
UGDH	-1.6	2.77E-03
HOXA11	-1.6	5.12E-02
TAP2	-1.6	1.31E-02

HGSNAT	-1.6	1.40E-02
ZNF358	-1.6	1.34E-02
COL4A6	-1.6	3.19E-02
PPARD	-1.6	4.85E-03
NAAA	-1.6	3.53E-02
LAPTM5	-1.6	3.96E-02
PNPLA7	-1.6	4.92E-03
NATD1	-1.6	4.27E-02
SLC44A4	-1.6	3.27E-03
GIMAP5	-1.6	4.42E-02
ACAD10	-1.6	1.02E-02
THRA	-1.6	2.58E-02
HS1BP3	-1.6	1.12E-02
ANKRD13A	-1.6	3.64E-03
HDAC10	-1.6	1.78E-03
IRF8	-1.6	1.94E-02
NR3C2	-1.6	2.11E-02
CDX2	-1.6	1.15E-02
TYROBP	-1.6	3.74E-02
PSME1	-1.6	5.26E-03
BCAS3	-1.6	2.27E-02
INPP5K	-1.6	7.22E-03
GMPR	-1.6	6.59E-03
LTBP3	-1.6	4.59E-02
CYB5RL	-1.6	5.37E-02
ATG4A	-1.6	1.45E-02
KLC4	-1.6	4.83E-03
CD44	-1.6	2.09E-02
ERBB3	-1.6	4.43E-02
IVD	-1.6	2.32E-02
DHRS11	-1.6	4.77E-03
CYBB	-1.6	3.34E-02
RARRES2	-1.6	4.83E-02
PDXK	-1.6	9.56E-03
SLC6A6	-1.6	5.12E-02
SCARB2	-1.6	1.71E-03
HOXA5	-1.6	1.74E-02
FBP2	-1.6	1.05E-02
CERS6	-1.6	3.04E-02
SLA-8	-1.6	1.98E-02
LRP4	-1.6	2.19E-02
GALNS	-1.6	1.45E-02
PNPLA6	-1.6	2.08E-02
ASCC2	-1.6	6.52E-04
FTL	-1.6	4.64E-02
MYH14	-1.6	1.75E-02
MNT	-1.6	1.51E-02
ACAD11	-1.6	1.82E-02
NOP53	-1.6	9.95E-03
VARS2	-1.5	1.93E-02
MT-CO2	-1.5	5.08E-02
THSD4	-1.5	2.95E-02
MAP1LC3A	-1.5	1.88E-02
TRAP1	-1.5	1.76E-02
SYNE1	-1.5	4.67E-02
VAV3	-1.5	5.02E-02
SLC9A3R1	-1.5	1.36E-02
FCER1G	-1.5	5.31E-02
CUEDC2	-1.5	2.10E-02
LIPA	-1.5	3.68E-02
TMEM94	-1.5	4.35E-03
MUL1	-1.5	1.04E-02
VSIR	-1.5	1.46E-02
NAT6	-1.5	5.14E-02
VIL1	-1.5	2.16E-02
PCBP4	-1.5	4.08E-02
CNNM4	-1.5	1.45E-02
TRIM66	-1.5	2.93E-02
TOM1L2	-1.5	3.51E-02
BBS4	-1.5	1.94E-02
CSF1	-1.5	3.58E-02
NT5C3A	-1.5	4.41E-02
ACVR1B	-1.5	1.73E-02
DIRC2	-1.5	4.91E-02

SCARF1	-1.5	4.78E-02
UVRAG	-1.5	1.97E-02
CTDSP2	-1.5	2.09E-02
IL17RA	-1.5	1.36E-02
SLC25A6	-1.5	2.26E-02
UNC93B1	-1.5	4.49E-02
BCL9L	-1.5	4.61E-02
NAV1	-1.5	3.87E-02
CLEC2D	-1.5	5.17E-02
DENND6B	-1.5	5.10E-02
PCGF2	-1.5	4.54E-02
SESN1	-1.5	2.85E-02
SLC25A5	-1.5	2.24E-02
BFAR	-1.5	1.98E-02
PSEN1	-1.5	1.41E-02
BCL2L14	-1.5	2.85E-02
TCN2	-1.5	3.88E-02
CAT	-1.5	1.33E-02
FCGRT	-1.5	2.30E-02
SLC13A1	-1.5	4.47E-02
SLC12A7	-1.5	2.28E-02
PPARA	-1.5	4.14E-02
MAFB	-1.5	3.08E-02
SP110	-1.5	5.40E-02
LENG8	-1.5	4.23E-02
ACOX1	-1.5	1.80E-02
ASPH	1.5	1.62E-02
HM13	1.5	4.05E-02
ZNF639	1.5	4.39E-02
TOR1AIP1	1.5	9.73E-03
MCF2L	1.5	4.37E-02
SLC35B1	1.5	2.46E-02
RNF115	1.5	3.78E-02
VKORC1L1	1.5	1.66E-02
ROCK1	1.5	3.08E-02
SLC39A11	1.5	5.49E-02
NUP153	1.5	2.91E-02
MAPK8	1.5	3.25E-02
MAP2K1	1.5	9.07E-03
LRRC8A	1.5	5.45E-02
UBE2N	1.5	3.17E-02
EIF5	1.5	1.51E-02
ACSL4	1.5	3.29E-02
MTHFD1	1.5	1.94E-02
FILIP1L	1.5	5.11E-02
ANP32E	1.5	2.39E-02
PTP4A1	1.5	4.70E-02
RASA2	1.5	1.67E-02
FAT1	1.5	4.34E-02
SUGT1	1.5	3.17E-02
UBA2	1.5	1.35E-02
SLC37A3	1.5	7.96E-03
TSPAN6	1.5	2.95E-02
YARS	1.5	4.20E-03
ITGB1	1.5	1.80E-02
SMAD1	1.5	1.98E-02
PSMD1	1.5	3.59E-02
HMGN3	1.5	2.23E-02
TNPO1	1.5	3.57E-02
ZW10	1.5	1.38E-02
PTPN11	1.5	1.93E-02
RAPGEF2	1.5	7.74E-03
DDX21	1.5	2.23E-02
ACKR3	1.5	4.95E-02
CEP350	1.5	2.57E-02
SEC16A	1.5	1.69E-02
LTBP1	1.5	2.73E-02
KPNB1	1.5	1.97E-02
MESDC1	1.5	1.49E-02
WDR36	1.5	2.03E-02
P2RX4	1.5	4.22E-02
MAGT1	1.5	2.43E-02
PDE4B	1.5	4.82E-02
DDX18	1.5	2.76E-02

CLTC	1.5	3.32E-02
SWAP70	1.5	2.99E-02
UBR1	1.5	1.69E-02
ARID3A	1.5	1.18E-02
NOP58	1.5	4.61E-02
PBRM1	1.5	3.12E-02
SRPR	1.5	1.16E-02
ZNF827	1.5	3.66E-02
STARD10	1.5	3.63E-02
TACC3	1.5	5.11E-02
MSH2	1.5	3.64E-02
SLC12A4	1.5	4.15E-03
SRSF10	1.5	4.44E-03
MOGS	1.5	5.43E-03
CSE1L	1.5	5.73E-03
QSER1	1.5	3.74E-02
SMC1A	1.5	3.99E-02
ASCC3	1.5	5.12E-02
SLC35C1	1.5	4.14E-02
PPIA	1.5	1.88E-02
DSC2	1.5	4.78E-02
GALNT1	1.5	4.20E-02
MAL2	1.5	1.07E-02
RAN	1.5	1.47E-02
MANSC1	1.5	2.58E-02
RACGAP1	1.5	5.25E-02
CAD	1.5	1.90E-02
HSPA4	1.5	1.23E-02
ZBTB1	1.5	3.22E-02
STARD5	1.5	4.88E-02
SELENOI	1.5	5.04E-03
CCP110	1.5	2.09E-02
TVP23B	1.5	3.30E-03
TMED2	1.5	1.25E-02
TRIP11	1.5	1.75E-02
LAMC1	1.6	2.40E-02
DDX3X	1.6	5.28E-02
ACACA	1.6	4.83E-03
CANX	1.6	2.07E-02
SEC23B	1.6	1.24E-02
BZW1	1.6	3.18E-02
IDE	1.6	7.53E-03
SELENOF	1.6	1.38E-02
SURF4	1.6	1.38E-02
APEX1	1.6	3.94E-03
GOLGA4	1.6	1.42E-02
PRDM1	1.6	2.16E-02
TOP2B	1.6	1.38E-02
NUP205	1.6	2.04E-02
BTAF1	1.6	1.43E-02
EIF2AK3	1.6	3.01E-03
ANXA2	1.6	1.42E-02
BAZ1A	1.6	7.89E-03
PDPN	1.6	4.98E-02
ATF4	1.6	1.20E-02
AARS	1.6	6.94E-03
SACS	1.6	2.04E-02
SEC61A1	1.6	9.70E-03
SEL1L3	1.6	2.74E-02
ZNF281	1.6	3.22E-03
STK39	1.6	3.52E-03
FZD6	1.6	5.05E-02
CLIC2	1.6	2.84E-02
FUT8	1.6	5.97E-03
AOAH	1.6	5.28E-02
ENTPD6	1.6	1.65E-02
WISP1	1.6	5.23E-02
SEL1L	1.6	4.20E-03
SLC39A10	1.6	5.43E-02
CASP3	1.6	2.55E-03
PAWR	1.6	2.76E-04
SERPINB1	1.6	3.13E-02
ADAMTS7	1.6	3.54E-02
ERLEC1	1.6	2.58E-02

C9	1.6	3.93E-02
ALDOC	1.6	5.08E-02
PPRC1	1.6	1.77E-02
MIS18BP1	1.6	3.18E-02
GOLGA3	1.6	2.64E-03
IQGAP1	1.6	6.60E-03
ZMYND19	1.6	3.44E-02
TUBA1B	1.6	6.88E-03
DOCK7	1.6	1.37E-02
EPRS	1.6	3.45E-03
CMAS	1.6	1.54E-02
SLC7A1	1.6	1.25E-02
FLVCR2	1.6	2.26E-02
PGM2	1.6	7.93E-03
VLDLR	1.6	1.87E-03
COL5A2	1.6	1.79E-02
S100A6	1.6	3.06E-02
AP3S1	1.7	1.36E-03
PTPN12	1.7	3.05E-04
CCDC134	1.7	4.17E-02
FOCAD	1.7	3.14E-03
ABCE1	1.7	3.69E-03
NAA15	1.7	1.71E-02
PRKAA2	1.7	3.44E-02
CARS	1.7	1.99E-03
ARG2	1.7	3.31E-02
SRP54	1.7	5.23E-03
SELENOK	1.7	1.33E-03
EIF4E	1.7	8.21E-03
PLA2G4A	1.7	3.82E-02
LTBP2	1.7	2.28E-02
ARCN1	1.7	2.11E-03
COPB2	1.7	6.68E-03
MAPK6	1.7	1.13E-02
UBE2S	1.7	1.08E-02
P4HA2	1.7	5.38E-03
CTNNB1	1.7	3.08E-03
ITGAV	1.7	7.96E-03
CDCA8	1.7	2.23E-02
ATM	1.7	1.39E-03
AMIGO3	1.7	2.55E-03
TOPBP1	1.7	3.75E-03
HSPD1	1.7	1.25E-02
SLC20A1	1.7	1.63E-02
GMPPB	1.7	8.21E-03
SOX9	1.7	1.52E-02
DNAJC25	1.7	1.46E-02
SLC30A7	1.7	2.93E-03
AMD1	1.7	3.24E-03
BRCA2	1.7	3.63E-02
THY1	1.7	1.92E-02
SOAT1	1.7	1.57E-02
BDP1	1.7	2.88E-03
UFM1	1.7	5.37E-03
MAP4K4	1.7	5.24E-04
ADAMTS9	1.7	4.78E-02
CASC4	1.7	1.90E-03
RPS6KA3	1.7	1.07E-03
MAN2A1	1.7	7.59E-03
SLC2A1	1.7	7.88E-03
TFPI2	1.7	5.42E-03
SLC7A6	1.7	2.15E-03
ATR	1.7	2.92E-02
RPN2	1.7	7.02E-03
CFI	1.7	4.15E-02
SLC39A7	1.7	1.29E-03
CBX5	1.7	9.86E-04
F2RL2	1.7	1.57E-02
PTGER2	1.7	1.84E-03
HMMR	1.7	4.64E-02
COPB1	1.7	7.69E-03
COL4A1	1.7	1.53E-02
BUB1	1.7	5.12E-02
E2F7	1.7	5.49E-02

C3	1.7	3.10E-02
TMED5	1.7	4.92E-04
SPATS2	1.7	8.34E-04
SAMSN1	1.7	1.38E-02
TNFRSF11A	1.7	3.61E-02
GALNT6	1.7	1.20E-02
DUSP5	1.7	1.26E-02
SPC24	1.7	5.41E-02
BTG2	1.7	9.85E-03
TMED10	1.7	1.41E-04
GRAP	1.7	2.12E-02
TOP2A	1.7	2.46E-02
NSDHL	1.7	4.11E-03
COL6A3	1.8	3.31E-02
CHORDC1	1.8	1.47E-02
ADM	1.8	2.21E-02
OSBPL8	1.8	5.05E-02
SKA2	1.8	1.91E-02
GARS	1.8	7.19E-04
TIFA	1.8	7.65E-03
STEAP2	1.8	2.56E-02
ECSCR	1.8	2.28E-02
SLC38A2	1.8	2.90E-02
NUDCD1	1.8	6.46E-03
SSR1	1.8	8.41E-04
TPX2	1.8	1.13E-02
MTHFD2	1.8	2.97E-03
TMED7	1.8	7.76E-04
SC5D	1.8	3.28E-03
MMP19	1.8	4.54E-02
CCT6A	1.8	4.24E-04
POLE	1.8	9.67E-03
CCND2	1.8	5.04E-03
CHEK2	1.8	3.88E-03
TNFSF15	1.8	2.54E-02
MIS18A	1.8	5.06E-02
SRGN	1.8	2.61E-03
MCTP2	1.8	1.96E-03
ERN2	1.8	1.38E-02
FGR	1.8	2.10E-02
PGK1	1.8	1.24E-03
MARS	1.8	4.34E-05
SLC16A10	1.8	3.61E-02
SLC3A2	1.8	1.83E-03
KLF15	1.8	3.84E-02
ACOT11	1.8	5.31E-02
CD24	1.8	4.34E-02
FAM20C	1.8	4.61E-03
KRT80	1.8	4.15E-02
TNFRSF10B	1.8	3.08E-03
ARF2	1.8	2.80E-03
SLC30A5	1.8	1.03E-03
TSPAN12	1.8	3.52E-04
THBD	1.8	2.09E-02
INTS2	1.8	3.06E-03
SEC61G	1.8	4.85E-03
DNA2	1.8	2.59E-02
GADD45A	1.8	2.23E-02
CD69	1.8	3.37E-02
ARF4	1.8	1.02E-03
SERP1	1.8	4.79E-04
SLC1A5	1.8	8.53E-04
IGJ	1.8	2.63E-02
TSPAN13	1.8	1.66E-03
IFRD1	1.8	2.25E-03
CCDC88B	1.8	1.78E-03
SLC6A9	1.8	1.29E-02
WFS1	1.9	1.20E-03
KIF5B	1.9	3.90E-03
GNE	1.9	2.36E-03
YARS2	1.9	2.28E-02
DHCR7	1.9	3.19E-04
PFKFB2	1.9	3.58E-02
SELENOM	1.9	2.48E-03

FDFT1	1.9	3.52E-04
HMGCR	1.9	1.25E-03
STT3A	1.9	1.19E-03
SULF2	1.9	2.64E-02
CALR	1.9	2.48E-03
ETV5	1.9	2.26E-02
CDC25C	1.9	6.70E-03
HSP90AA1	1.9	2.84E-02
METTL16	1.9	2.84E-02
PPIB	1.9	7.19E-04
PANK1	1.9	9.77E-03
CXCR4	1.9	1.45E-02
SLC23A1	1.9	3.44E-03
HSPA8	1.9	3.54E-02
ITGA1	1.9	3.71E-02
BLM	1.9	1.56E-02
SKIL	1.9	3.19E-05
SERPINB5	1.9	8.72E-03
DNAJC10	1.9	4.24E-04
SPRED1	1.9	3.24E-05
SLC7A2	1.9	4.15E-02
CD163	1.9	4.34E-02
DSE	1.9	4.61E-03
EDEM3	1.9	1.53E-02
LRRC59	1.9	1.99E-03
ADGRG6	1.9	2.65E-03
MIA3	1.9	1.23E-04
PKM	1.9	3.11E-04
ECM1	1.9	4.06E-03
HELLS	1.9	4.19E-03
GALNT7	1.9	5.18E-04
MVD	1.9	2.48E-04
RRM2	1.9	2.76E-04
MCM6	1.9	3.68E-04
FNDC3B	1.9	4.00E-03
SHMT2	2.0	2.65E-05
FOXA1	2.0	6.53E-03
SREBF1	2.0	1.67E-04
ECT2	2.0	1.69E-02
SERPINH1	2.0	7.17E-03
ST7	2.0	4.61E-03
MMD	2.0	9.89E-04
EDEM1	2.0	5.13E-05
ABCA5	2.0	4.93E-03
WNT5A	2.0	2.03E-02
OSMR	2.0	1.41E-04
XBP1	2.0	2.73E-04
IL17RD	2.0	2.03E-02
DDIT4	2.0	1.40E-04
ATOX1	2.0	3.87E-03
AACS	2.0	4.99E-05
RIPK3	2.0	5.75E-04
ACSL3	2.0	8.07E-05
BST1	2.0	9.80E-04
PLSCR4	2.0	4.20E-03
ASNSD1	2.0	1.71E-03
KIF23	2.0	5.38E-03
IL33	2.0	9.72E-04
PCK2	2.0	1.77E-03
CYP51A1	2.1	1.08E-04
TARS	2.1	7.29E-05
SLC35A3	2.1	3.51E-03
WLS	2.1	7.12E-04
CEBPB	2.1	3.29E-03
CFB	2.1	1.92E-02
WNT9B	2.1	3.98E-02
SEC24D	2.1	2.38E-06
DNAJA1	2.1	4.93E-03
SEC11C	2.1	2.82E-04
ANKRD12	2.1	1.18E-03
RRBP1	2.1	1.96E-04
COL12A1	2.1	1.41E-04
MEST	2.1	4.67E-02
TMED3	2.1	3.45E-03

AFF2	2.1	2.84E-02
PGM3	2.1	7.37E-05
GPR19	2.1	2.11E-02
COL7A1	2.1	1.26E-03
ADGRE2	2.1	2.00E-02
ANXA5	2.1	1.44E-05
CDH1	2.1	1.78E-04
PROCR	2.1	1.02E-02
SELENOS	2.1	1.21E-05
FASN	2.1	1.09E-04
DNAJC3	2.1	2.07E-05
CD14	2.1	1.23E-02
PIK3C2A	2.1	1.70E-03
CTGF	2.1	2.04E-02
AREG	2.1	2.09E-02
IRF4	2.1	2.55E-03
CSRP2	2.1	1.90E-04
ZGRF1	2.1	7.88E-03
TMEM38B	2.1	1.48E-03
ACLY	2.2	5.17E-06
SLC41A2	2.2	5.17E-02
GINS1	2.2	2.93E-03
STC1	2.2	1.42E-02
KIAA0101	2.2	1.07E-03
LSS	2.2	1.54E-05
FICD	2.2	1.96E-04
PANK3	2.2	4.27E-02
DEPDC1	2.2	2.26E-02
MYOF	2.2	3.03E-05
CD79A	2.2	1.05E-02
SLC5A1	2.2	2.73E-04
LIPH	2.2	2.78E-04
FDPS	2.2	8.28E-05
TNFRSF26	2.2	5.68E-04
GFPT1	2.2	1.21E-05
SLC17A9	2.2	1.07E-05
DERL3	2.2	2.74E-04
DIAPH3	2.2	2.40E-02
SH2D1B	2.2	5.04E-02
CDR2	2.2	1.11E-04
LOXL4	2.2	3.47E-02
ADM5	2.2	5.60E-03
ITGA8	2.2	1.15E-04
OLFM4	2.2	1.48E-02
TGM2	2.2	3.66E-05
SPDL1	2.3	2.87E-02
KNL1	2.3	4.26E-03
FCGBP	2.3	2.08E-02
IL18	2.3	7.08E-03
PHLDA1	2.3	3.66E-04
TNFAIP6	2.3	2.84E-02
VSTM1	2.3	1.95E-02
PTGER1	2.3	1.21E-02
PTGS1	2.3	9.54E-06
GPER1	2.3	1.39E-02
FFAR4	2.3	1.81E-02
MKX	2.3	2.40E-02
HYOU1	2.3	4.56E-05
HSD11B1	2.3	7.62E-03
PDIA4	2.3	1.15E-04
CCL28	2.3	4.92E-03
CHIA	2.3	3.56E-02
BCAT1	2.4	4.81E-04
PTPRN2	2.4	2.45E-02
ADAM32	2.4	1.08E-02
DEPDC7	2.4	4.79E-02
SFRP1	2.4	2.73E-04
SMOC2	2.4	3.44E-03
SCNN1D	2.4	1.45E-02
PDZK1IP1L	2.4	4.24E-02
GJA1	2.4	6.11E-05
BMP7	2.4	6.17E-05
HIF1A	2.4	9.56E-06
SLC15A2	2.4	2.11E-03

NFIL3	2.4	3.14E-09
CDK1	2.4	1.89E-03
TNFRSF6B	2.4	4.77E-03
FGF2	2.4	5.70E-04
TRIB3	2.4	1.05E-02
CHEK1	2.4	3.35E-03
AHNAK2	2.4	4.81E-02
NDUFA4L2	2.4	1.77E-03
FADS1	2.4	6.98E-08
TIGIT	2.5	3.55E-03
ATF5	2.5	2.26E-02
GABRD	2.5	4.65E-02
FOS	2.5	2.09E-02
PRDX4	2.5	3.41E-05
ATP13A3	2.5	7.31E-03
IFIT1L1	2.5	3.82E-02
HSPH1	2.5	1.97E-02
SLC1A4	2.5	1.36E-03
LMAN1	2.5	3.59E-06
SHCBP1	2.5	1.06E-03
AHSA2	2.5	3.80E-05
MSMO1	2.5	5.94E-06
SMC2	2.5	1.70E-04
"MARCH3"	2.5	5.78E-04
SNORA63	2.5	4.32E-02
SOCS3	2.5	1.07E-03
LAMB3	2.5	2.26E-06
CCR2	2.5	2.63E-03
EGF	2.5	1.10E-03
SLC16A3	2.6	1.23E-03
EBP	2.6	1.17E-05
NT5DC2	2.6	1.65E-05
ADAM9	2.6	1.62E-07
RBPJL	2.6	1.02E-02
ROS1	2.6	3.77E-02
ITGA2	2.6	3.66E-05
ADAMTS4	2.6	4.92E-03
IGFBP4	2.6	2.53E-05
KRT18	2.6	4.34E-05
LOC102161685	2.7	3.95E-04
MYBL1	2.7	2.28E-02
BDNF	2.7	1.64E-02
HSD17B7	2.7	9.57E-06
PPP2R5E	2.7	2.55E-02
HSP90B1	2.7	1.17E-05
ELOVL6	2.7	1.65E-05
SNCA	2.7	1.40E-02
SLC7A5	2.7	7.21E-06
ABAT	2.7	2.61E-02
CSF3R	2.7	1.81E-02
TFF3	2.7	2.49E-03
MANF	2.7	6.81E-06
CXCR1	2.7	1.71E-02
IGLL5L	2.7	7.30E-03
SERPINB8	2.7	2.47E-05
GCNT3	2.7	1.21E-03
PNP	2.8	6.81E-06
FADS2	2.8	2.38E-06
PHLDA2	2.8	4.07E-04
TFRC	2.8	3.47E-05
SPHK1	2.8	3.05E-03
STARD4	2.8	1.65E-06
GPAT3	2.8	1.40E-04
CXCR2	2.8	2.64E-02
LDLR	2.8	1.77E-06
UGGT1	2.9	5.68E-03
TNC	2.9	8.70E-04
SQLE	2.9	7.52E-07
CXCL1	2.9	1.56E-02
ETV4	2.9	3.43E-04
ADAM20L2	2.9	2.39E-05
IL1RAP	2.9	2.14E-03
SNORA20E	2.9	4.65E-02
LCN2	3.0	1.80E-02

BAALC	3.0	5.32E-04
SEMA7A	3.0	1.23E-05
MIR9829	3.0	5.30E-02
SLC1A2	3.0	2.04E-03
TLE6	3.0	6.63E-03
FFAR2	3.0	5.18E-04
SHISA8	3.0	2.45E-02
HSPA5	3.0	1.21E-05
B3GNT6	3.0	2.31E-03
SLCO4A1	3.1	7.29E-05
SERPINE2	3.1	1.06E-04
DHRS9	3.1	4.24E-04
IDI1	3.1	3.39E-06
INSIG1	3.1	6.97E-07
C4BPB	3.1	5.70E-06
FGF7	3.2	4.44E-05
CLCA1	3.2	1.07E-03
PFKFB3	3.2	4.64E-06
IL13RA2	3.3	5.25E-02
POU2AF1	3.3	9.54E-06
CTSG	3.3	1.58E-02
NKX2-2	3.3	5.22E-03
CBS	3.4	1.61E-03
SLC2A3	3.4	1.42E-12
CXCL8	3.4	5.32E-04
MMP3	3.5	3.34E-02
ACAT2	3.5	1.27E-10
SLC12A8	3.5	7.40E-04
CXCL14	3.5	9.85E-08
SPHKAP	3.5	8.66E-04
TIAM1	3.5	2.62E-05
RAET1EL3	3.6	4.51E-03
SYNE4	3.6	2.31E-02
PLA2G3	3.6	7.51E-05
ITLN2L*	3.6	2.78E-02
CLEC10A	3.6	2.24E-02
HHIPL1	3.6	1.65E-05
IL1A	3.6	1.30E-03
CMAH	3.7	4.42E-02
MYOM3	3.7	1.26E-03
GPRC5D	3.7	4.11E-02
KLRG2	3.7	4.54E-02
MED12L	3.8	5.39E-05
TGM1	3.8	3.08E-02
ARNTL2	3.8	6.51E-06
HMGCS1	3.9	7.11E-09
SCD	3.9	9.81E-07
SHANK2	3.9	1.44E-03
MS4A7	3.9	2.55E-03
WNT2	3.9	6.32E-03
LIPG	4.0	1.77E-03
STEAP4	4.0	5.38E-03
BDKRB1	4.0	1.65E-03
COL8A1	4.1	1.19E-06
HEPHL1	4.1	5.31E-02
ABCA12	4.1	1.91E-03
IL26	4.1	2.96E-03
SFTPD	4.1	2.73E-02
ADAM12	4.2	4.78E-04
TAT	4.2	4.00E-02
CA4	4.3	9.18E-05
TIMP1	4.3	2.48E-09
NRG1	4.3	8.21E-05
LRP2	4.3	1.38E-02
C4BPA	4.3	1.37E-03
P4HA3	4.4	3.89E-04
SPDEF	4.4	3.89E-06
OSM	4.4	1.92E-04
F13A1	4.4	1.41E-09
WAP1	4.4	5.33E-03
KIAA1199	4.6	4.92E-03
IGFBP2	4.6	3.68E-07
PLAUR	4.6	3.65E-06
TNFRSF11B	4.6	1.51E-04

RN7SL1	4.6	2.45E-02
ANKRD26L3	4.6	3.02E-02
CAPNS2	4.7	3.43E-04
COL28A1	4.7	5.24E-04
SERPINA1	4.7	5.81E-03
PI3	4.7	1.63E-02
SLC4A11	4.8	5.93E-03
RNF39	4.8	2.65E-05
IGHE	5.0	2.59E-06
TNFRSF12A	5.0	6.86E-10
CENPE	5.0	4.28E-03
PHGDH	5.1	3.92E-05
CCL23	5.1	1.56E-04
NPPC	5.1	3.89E-03
MUC2	5.2	7.10E-08
CXCL6	5.3	2.39E-05
AQP9	5.3	1.56E-02
ITGB6	5.4	8.74E-11
IL1B	5.5	1.20E-03
GZMB	5.5	2.79E-04
SDS	5.6	1.53E-02
NXPH4	5.6	3.16E-05
NEUROG3	5.7	1.61E-03
IGH@	5.7	2.70E-05
MPTX	5.8	4.12E-06
CHI3L2	6.0	7.37E-04
ADAM20L6	6.1	2.85E-02
TREM1	6.4	4.61E-03
SLC6A13	6.5	1.54E-02
AGR2	6.5	1.79E-05
IL6	6.5	1.29E-04
PTGS2	6.5	8.39E-06
DIO2	6.5	3.02E-04
SPINK4	6.6	2.11E-06
ADAMTS16	6.6	2.55E-03
BPIFB2	6.7	2.43E-03
AKR1C1	7.0	5.11E-02
C1QL2	7.0	4.51E-02
CRLF1	7.1	3.16E-05
PSAT1	7.2	3.12E-04
TNIP3	7.3	4.56E-05
MCPT3*	7.3	2.80E-02
COL26A1	7.3	2.71E-04
HOXD1	7.4	6.83E-03
S100A2	7.4	9.54E-06
MMP20	7.4	4.14E-02
ADM2	7.9	1.29E-05
S100A3	8.0	6.29E-03
RHCG	8.0	4.93E-02
FAM71E2	8.2	9.06E-03
F3	8.2	7.74E-11
HK2	8.6	2.40E-06
SCGB3A1	8.8	1.90E-02
MMP9	8.9	1.02E-08
MMP1	9.4	4.44E-05
SERPINB2	9.8	2.12E-04
SNORA20	9.8	2.20E-03
SLC7A11	9.9	9.13E-04
KLRJ1	10.0	5.18E-04
RETNLB	10.1	2.14E-03
IL1R2	10.3	2.46E-06
SNORA73AL	10.5	2.14E-02
PRG4	10.7	1.39E-02
UPK1B	10.9	1.66E-08
PADI4	11.0	7.93E-03
BPIFB6	11.0	4.32E-02
SERPINB11	11.3	9.04E-04
PCSK9	11.5	5.33E-09
SLC5A8	11.9	4.56E-05
CYP7A1	12.7	4.64E-02
LYZ	13.0	7.77E-03
HCAR2	13.3	7.26E-04
SULT2A1	13.4	4.28E-02
CLEC4D	13.4	4.10E-02

CHI3L1	13.9	2.38E-06
REG4	15.8	2.97E-08
MAGEA13P	15.8	1.76E-02
MCHR1	17.9	7.59E-03
SERPINB7	19.3	1.85E-02
IL11	19.4	3.58E-04
MMP13	20.2	1.77E-06
ANXA8	21.8	2.21E-07
IL36A	22.7	3.90E-04
XDH	22.8	3.03E-02
CHIT1	24.2	1.65E-02
TFF2	25.1	3.57E-02
MMP8	27.1	7.94E-03
CLDN9	27.7	5.43E-02
IGHG1	29.1	1.93E-04
CLEC18A	29.5	7.94E-03
MMP7	33.4	3.22E-03
AQP5	33.5	4.54E-04
S100A8	38.3	5.18E-04
S100A12	38.4	4.48E-04
SDSL	39.6	1.38E-02
A3GALT2	40.6	5.54E-03
NTRK1	42.0	2.62E-07
S100A9	44.8	2.59E-04
SPINK6	46.2	5.38E-03
LTF	52.9	5.95E-03
TCN1	54.1	1.15E-04
PADI3	55.7	1.75E-02
SERPINA3-2	60.8	2.96E-06
TNN	61.3	4.18E-05
AVPR1B	62.6	5.42E-03
GNLY	68.8	2.42E-09
MMP12	101.0	2.26E-14
SCGB2A2	105.1	3.14E-07
ARG1	122.6	4.56E-05
IL4I1L	161.2	3.98E-05
REG3A	275.6	2.48E-03
PADI1	296.4	3.51E-03
TFF1	705.1	2.48E-05

Supplemental Table S6. Comparison of DEGs expressed at 52 days after inoculation in pigs infected with Trichuris suis with no worms (worm-free) versus those with worm.

Feature ID	Fold change	EDGE test: FDR p-value correction
SLC10A2	-297.1	1.18E-04
ALPI	-32.9	4.94E-06
ALDOB	-21.0	1.66E-06
BTNL2	-19.6	1.37E-02
AQP8	-15.1	2.08E-03
NXPE2	-13.8	2.92E-05
THAP12	-13.4	2.27E-02
CLCA4	-13.2	2.57E-05
HMGCS2	-12.8	0.00E+00
OASL	-12.1	1.20E-02
SLC26A3	-12.0	9.52E-08
FRMD1	-9.6	1.51E-03
FOXO6	-9.1	1.78E-04
NR1H4	-7.6	1.96E-06
GSTA2	-7.4	7.07E-03
CYP2B22	-6.8	5.35E-03
SLC30A10	-6.3	2.38E-02
SI	-6.1	2.79E-02
COL6A6	-5.7	3.03E-06
CXCL9	-5.7	3.65E-03
DACT2	-5.4	1.74E-02
PCK1	-5.4	1.48E-03
PADI2	-5.2	1.54E-06
HAS3	-5.2	5.01E-04
SULT1E1	-5.2	7.10E-07
GUCA2A	-5.1	1.44E-04
MOGAT2	-4.9	2.97E-02
NCR2	-4.8	1.35E-02
SLC51A	-4.8	3.48E-06
SSTR5	-4.8	1.30E-02
CYP26B1	-4.6	4.16E-08
ABCA6	-4.4	1.88E-04
SLC16A1	-4.4	0.00E+00
CXCL10	-4.4	7.18E-03
RSAD2	-4.3	2.28E-02
AGT	-4.3	4.12E-03
SLC25A34	-4.2	1.12E-05
GABRB2	-4.0	1.45E-04
TM6SF2	-4.0	1.95E-09
IFI27	-3.9	1.12E-02
CA1	-3.9	1.38E-08
P2RX3	-3.9	4.22E-03
ABCG2	-3.4	2.49E-03
WNT8B	-3.1	1.99E-03
CA12	-3.1	2.51E-06
SLC38A4	-3.0	3.10E-03
SELENBP1	-3.0	1.73E-07
PARP15	-3.0	6.17E-03
CHP2	-2.9	5.14E-05
CNTFR	-2.9	3.11E-03
CTBS	-2.9	4.10E-02
CLDN10	-2.8	4.58E-02
CXCL12	-2.8	2.48E-04
SCN2A	-2.7	4.98E-02
ABCA9	-2.7	5.24E-02
PRSS12	-2.7	9.44E-04
XCL1	-2.7	5.72E-03
ABCA8	-2.7	4.77E-02
GRIK4	-2.7	9.60E-03
IFI44	-2.7	2.55E-02
DHX58	-2.7	3.59E-02
IRF4L	-2.7	3.39E-02
CCL8	-2.6	7.80E-03
LYPD6	-2.6	3.08E-02
AHRR	-2.6	6.47E-04
HOXD10	-2.6	1.40E-03
UGT2C1	-2.5	1.18E-05
FBXO32	-2.5	2.94E-04
HS3ST3A1	-2.5	4.16E-02
UBE2L6	-2.5	1.39E-04
ZBP1	-2.5	1.11E-02
FOXS1	-2.5	5.82E-03
ABI3BP	-2.5	1.33E-03
PTPRD	-2.5	2.63E-04
CD36	-2.4	7.42E-05
CD300C	-2.4	8.64E-04
SLC24A4	-2.4	1.05E-02
LILRB3L4	-2.4	5.21E-02

MAMDC2	-2.4	6.26E-03
GBP2	-2.4	9.65E-03
AOC1	-2.4	4.46E-05
FABP1	-2.4	1.03E-02
FCER1A	-2.3	3.53E-02
SELENOP	-2.3	5.83E-05
SLC7A3L7*	-2.3	4.90E-02
NT5E	-2.3	4.92E-03
C11orf86	-2.2	2.08E-03
SEMA3A	-2.2	1.38E-02
SECTM1	-2.2	4.77E-02
PRDX6	-2.2	5.44E-05
CARD14	-2.2	2.07E-03
IGF2	-2.2	2.80E-02
TLR8	-2.2	2.49E-03
SLC7A8	-2.1	5.64E-03
GBP6	-2.1	3.20E-02
AHCYL2	-2.1	5.97E-05
CTSW	-2.1	1.42E-02
DBP	-2.1	1.42E-02
PARP14	-2.1	6.98E-03
SLA-DRA	-2.1	5.53E-03
SLC25A42	-2.1	7.36E-03
GLI2	-2.1	7.08E-03
MPEG1	-2.1	5.72E-03
ANK3	-2.1	2.79E-02
SLC7A3L4*	-2.1	4.13E-02
EDIL3	-2.1	5.28E-03
CD209	-2.1	3.13E-02
IL34	-2.1	4.12E-03
RYR2	-2.0	2.80E-03
KLRG1	-2.0	4.23E-02
CYP7B1	-2.0	1.59E-02
TXNIP	-2.0	3.65E-03
ADRB1	-2.0	7.57E-03
CD4	-2.0	1.03E-02
SLA-DRB1	-2.0	1.25E-02
ALDH1A3	-2.0	1.10E-02
GBP1	-2.0	4.04E-02
SLA-DQA1	-2.0	1.33E-02
IFI44L	-2.0	1.59E-02
P2RY13	-2.0	1.81E-02
CD74	-2.0	7.72E-03
ACE	-2.0	6.94E-03
ABCB1	-2.0	7.10E-03
PARP9	-1.9	1.04E-03
PCSK6	-1.9	2.26E-04
ANKH	-1.9	4.54E-02
HOXD11	-1.9	5.02E-02
GGT1	-1.9	4.29E-02
MAF	-1.9	1.80E-03
EGFLAM	-1.9	5.37E-03
CAVIN2	-1.9	9.17E-03
SYNPO	-1.9	3.13E-03
MS4A2	-1.9	2.34E-02
ADAMTS8	-1.9	1.07E-02
COLEC12	-1.9	7.57E-03
LY9L1*	-1.9	1.61E-02
CST3	-1.9	7.80E-03
MAOA	-1.9	7.84E-04
DAPK1	-1.9	1.20E-02
FOXF2	-1.9	5.72E-03
KLHL7	-1.9	2.88E-03
SEMA4D	-1.8	3.81E-02
RNF213	-1.8	3.66E-02
HAPLN4	-1.8	5.64E-03
ACSF2	-1.8	1.98E-04
SATB1	-1.8	2.46E-02
PKNOX2	-1.8	5.16E-02
NR1D1	-1.8	3.13E-02
SLC16A5	-1.8	9.64E-03
CADM1	-1.8	2.11E-02
TEF	-1.8	3.94E-03
MEIS2	-1.8	5.29E-02
AKR1E2	-1.8	4.12E-03
ADGRL1	-1.8	4.66E-02
HIC1	-1.8	1.41E-02
MAP2K6	-1.8	4.90E-02
TMPRSS2	-1.8	1.60E-02
LGALS9	-1.8	1.10E-02

CLCN2	-1.8	3.54E-05
POU6F1	-1.8	3.37E-02
CD86	-1.8	1.93E-02
LTBP4	-1.8	5.61E-03
WNT2B	-1.8	1.98E-02
ADA	-1.7	2.04E-02
LIFR	-1.7	7.18E-03
ARHGAP6	-1.7	3.47E-02
IFITM1L2	-1.7	8.91E-03
FGFRL1	-1.7	1.45E-02
CIITA	-1.7	4.54E-02
SLA-DMB	-1.7	2.70E-02
IHH	-1.7	7.62E-03
ZBTB4	-1.7	1.22E-03
TAP1	-1.7	2.23E-02
SLC9A2	-1.7	1.92E-03
PTCH1	-1.7	4.04E-02
C1QA	-1.7	1.29E-02
CDKN2B	-1.7	7.89E-03
SLA-2	-1.7	5.02E-02
BDH1	-1.7	7.89E-03
FCGR3A	-1.7	1.21E-02
APOL2	-1.7	3.42E-02
C1QC	-1.7	1.61E-02
ABCA1	-1.7	5.31E-02
CDH24	-1.7	3.29E-02
IL2RB	-1.7	2.69E-02
JAML	-1.7	3.23E-02
TNFAIP2	-1.7	1.04E-03
ABCB6	-1.6	5.08E-03
CTSD	-1.6	1.68E-02
DOK2	-1.6	3.97E-02
SLC45A3	-1.6	2.17E-02
IGSF3	-1.6	5.40E-02
TMPRSS4	-1.6	6.34E-03
CD33	-1.6	4.32E-02
FGF11	-1.6	2.39E-02
SLA-3	-1.6	4.54E-02
TCF21	-1.6	1.33E-02
SNX4	-1.6	5.72E-03
PNPLA6	-1.6	6.03E-03
CD302	-1.6	5.19E-02
PFKFB4	-1.6	5.72E-03
RETSAT	-1.6	7.89E-03
LCP2	-1.6	1.11E-02
GIMAP8	-1.6	1.73E-02
NADK	-1.6	4.96E-03
F8	-1.6	3.01E-02
SCARB2	-1.6	5.21E-04
SLC25A27	-1.6	7.18E-03
ELMSAN1	-1.6	3.05E-02
SLA-11	-1.6	4.46E-02
ZNF664	-1.6	5.37E-03
SLC8A1	-1.6	5.29E-02
TP53INP2	-1.6	3.64E-02
RALGDS	-1.6	2.21E-02
COL4A6	-1.6	4.50E-02
SIPA1	-1.6	5.29E-02
CSF2RB	-1.5	4.36E-02
CD300H	-1.5	3.24E-02
GSTP1	-1.5	4.90E-02
PIK3IP1	-1.5	4.80E-02
TMEM140	-1.5	3.04E-02
CSF1	-1.5	1.68E-02
PLCD1	-1.5	3.39E-02
VDR	-1.5	3.04E-02
IL10RA	-1.5	3.17E-02
IRF9	-1.5	4.68E-02
ASAP3	-1.5	4.15E-02
MERTK	-1.5	4.10E-02
SYNE1	-1.5	4.69E-02
DTX3	-1.5	2.14E-02
BSG	-1.5	2.27E-02
ADCY9	-1.5	5.72E-03
MOV10	-1.5	1.20E-02
HPSE	-1.5	5.25E-02
UGT1A10	-1.5	8.65E-03
PNPLA2	-1.5	3.42E-02
TST	-1.5	5.16E-02
COBL	-1.5	1.01E-02

TSPAN7	-1.5	3.19E-02
UNC93B1	-1.5	4.34E-02
LRP4	-1.5	5.02E-02
MAP3K3	-1.5	1.38E-02
CSF1R	-1.5	4.54E-02
GABARAPL1	-1.5	2.90E-02
PCGF2	-1.5	5.29E-02
SLC30A7	1.5	3.01E-02
TMED2	1.5	1.87E-02
HMGB1	1.5	1.59E-02
ENTPD6	1.5	4.58E-02
EPRS	1.5	1.81E-02
SMAD1	1.5	1.17E-02
MAP4K4	1.5	9.03E-03
SELENOS	1.5	3.07E-02
SLC39A11	1.5	3.01E-02
EDEM1	1.5	2.46E-02
MAPK6	1.5	4.90E-02
MET	1.5	4.52E-02
SLC30A5	1.5	3.16E-02
NECTIN1	1.5	3.13E-02
MAL2	1.5	8.79E-03
CDH17	1.5	3.46E-02
COPB1	1.5	4.68E-02
SHMT2	1.5	1.06E-02
VLDLR	1.5	7.00E-03
ACTG1	1.5	2.84E-03
ARF2	1.5	4.54E-02
SLC39A7	1.5	1.20E-02
ERLEC1	1.5	4.94E-02
SLC7A1	1.5	3.05E-02
SPRED1	1.5	7.34E-03
SELENOF	1.5	1.19E-02
S100A11	1.5	3.35E-02
UFM1	1.5	2.36E-02
ANXA5	1.5	1.54E-02
TACC3	1.5	3.05E-02
PGK1	1.5	1.73E-02
HMGB2	1.5	1.72E-02
CTNNB1	1.5	8.88E-03
MYBL2	1.5	3.16E-02
MCM6	1.5	2.31E-02
STT3A	1.5	2.64E-02
C9	1.5	5.09E-02
CBFA2T2	1.5	2.97E-02
ANXA2	1.5	9.17E-03
SERP1	1.5	8.65E-03
PRTFDC1	1.5	4.54E-02
RACGAP1	1.5	2.85E-02
AMIGO3	1.6	5.61E-03
OSMR	1.6	1.24E-02
RPN2	1.6	1.62E-02
ODC1	1.6	4.12E-03
FZD6	1.6	4.12E-02
HMGN2	1.6	1.45E-03
SPATS2	1.6	3.11E-03
UBE2S	1.6	1.44E-02
PLK1	1.6	1.79E-02
SKA2	1.6	5.02E-02
TSPAN13	1.6	1.37E-02
CHEK2	1.6	1.60E-02
BNIP3	1.6	7.73E-03
KCNE3	1.6	6.59E-03
FICD	1.6	2.91E-02
SLC35C1	1.6	7.89E-03
SEC24D	1.6	1.45E-03
SELENOM	1.6	1.73E-02
TMEM38B	1.6	5.49E-02
MIA3	1.6	4.10E-03
SLC1A5	1.6	5.72E-03
SERPINB1	1.6	1.75E-02
P2RX4	1.6	4.88E-03
BIRC5	1.6	1.82E-02
GARS	1.6	1.95E-03
ADGRG6	1.6	2.36E-02
CASC4	1.6	2.07E-03
TK1	1.6	3.23E-02
CLIC2	1.6	1.22E-02
LRRC59	1.6	1.88E-02
RRBP1	1.6	1.17E-02

STARD10	1.6	4.04E-03
SLC17A9	1.6	5.72E-03
P4HA2	1.6	3.60E-03
KIF22	1.6	9.28E-03
CYP51A1	1.6	5.76E-03
C3	1.6	3.66E-02
CAD	1.6	1.54E-03
SEL1L3	1.6	7.42E-03
PCDH12	1.6	5.06E-02
ARG2	1.6	2.16E-02
S100A6	1.6	1.55E-02
CMAS	1.6	6.03E-03
CFI	1.6	4.23E-02
DHCR7	1.6	1.99E-03
POLE	1.6	1.60E-02
FOXA1	1.6	3.80E-02
MANF	1.6	3.08E-02
NSDHL	1.6	4.96E-03
WLS	1.6	1.61E-02
CDH1	1.6	8.68E-03
B4GALT1	1.6	4.04E-02
C9H1orf116	1.6	7.00E-03
GFPT1	1.7	3.70E-03
SEC11C	1.7	8.53E-03
PPIB	1.7	3.55E-03
IL33	1.7	1.40E-02
HSP90B1	1.7	2.68E-02
DNAJC10	1.7	3.09E-03
PLK2	1.7	9.11E-03
SEC61G	1.7	7.96E-03
PANK1	1.7	2.78E-02
ACSL3	1.7	2.03E-03
ARF4	1.7	1.90E-03
XBP1	1.7	3.59E-03
STARD5	1.7	4.28E-03
TSPAN12	1.7	4.45E-04
RIPK3	1.7	5.64E-03
FASN	1.7	3.60E-03
DNA2	1.7	3.13E-02
GALNT7	1.7	2.49E-03
CDC25C	1.7	1.33E-02
GNA14	1.7	5.35E-03
PKM	1.7	1.40E-03
WFS1	1.7	1.61E-03
LIPH	1.7	8.88E-03
BLM	1.7	3.03E-02
TPX2	1.7	6.03E-03
ALDOC	1.7	1.06E-02
HIF1A	1.7	3.73E-03
BST1	1.7	5.72E-03
MMD	1.7	4.10E-03
SC5D	1.7	1.42E-03
ATOH1	1.7	2.37E-02
DERL3	1.7	7.70E-03
PHLDA1	1.7	1.31E-02
YARS2	1.7	2.77E-02
DDIT4	1.7	9.24E-04
SLC5A1	1.7	6.38E-03
SLC35A3	1.7	1.61E-02
PDIA4	1.7	7.74E-03
FLVCR2	1.8	2.73E-03
MANSC1	1.8	2.51E-04
BUB1	1.8	2.17E-02
FDPS	1.8	2.52E-03
PGM3	1.8	9.17E-04
HELLS	1.8	7.06E-03
MVD	1.8	5.01E-04
CCNB2	1.8	2.21E-02
BGN	1.8	4.69E-02
ATOX1	1.8	8.67E-03
PNP	1.8	7.47E-03
NT5DC2	1.8	5.72E-03
LSS	1.8	3.41E-04
AACS	1.8	1.18E-04
ARNTL2	1.8	5.25E-02
FADS1	1.8	8.80E-05
RRM2	1.8	2.15E-04
BUB1B	1.8	9.28E-03
ECM1	1.8	3.23E-03
TOP2A	1.8	4.25E-03

DGAT2	1.8	3.05E-02
E2F7	1.8	1.34E-02
ETV5	1.8	1.26E-02
SLC50A1	1.8	9.18E-04
ECSCR	1.8	3.94E-03
MIS18A	1.9	1.76E-02
ADAM9	1.9	2.26E-04
AFF2	1.9	4.49E-02
TRAIP	1.9	5.08E-03
PHLDA2	1.9	2.92E-02
ZGRF1	1.9	2.01E-02
SMOC2	1.9	2.90E-02
CDCA8	1.9	9.35E-04
SLC24A5	1.9	4.98E-02
HMGCR	1.9	1.78E-04
GADD45A	1.9	5.02E-03
CFB	1.9	2.38E-02
GINS1	1.9	7.55E-03
KIF23	1.9	5.99E-03
ACLY	1.9	1.76E-05
BCAT1	1.9	5.18E-03
CCR2	1.9	3.11E-02
MYOF	1.9	1.39E-04
CD163	1.9	2.29E-02
THBD	1.9	3.32E-03
COL7A1	1.9	1.47E-03
BMP7	1.9	1.22E-03
SMC2	1.9	4.42E-03
FDFT1	1.9	1.14E-05
MASTL	1.9	4.54E-02
SULT1A1	1.9	1.57E-02
PRDX4	1.9	1.03E-03
"MARCH3"	1.9	8.78E-03
SLC7A5	1.9	1.40E-03
CHEK1	1.9	2.00E-02
CD24	1.9	6.90E-03
STARD4	1.9	8.33E-04
HSD17B7	1.9	1.22E-03
LMAN1	2.0	1.88E-04
SERPINE2	2.0	1.59E-02
PKD1L2	2.0	4.19E-02
SLC25A35	2.0	9.64E-03
DEPDC1	2.0	3.05E-02
ADM5	2.0	7.68E-03
GPR19	2.0	1.76E-02
GNE	2.0	5.66E-05
COL8A1	2.0	1.40E-02
CD14	2.0	7.89E-03
CDK1	2.0	7.19E-03
IGLL5L	2.0	5.49E-02
SERPINB5	2.0	9.60E-04
AZGP1	2.0	3.20E-02
KIAA0101	2.0	7.84E-04
KRT18	2.0	1.04E-03
PCK2	2.0	3.22E-04
ADAM20L2	2.0	2.60E-03
TMED3	2.0	1.16E-03
SHCBP1	2.0	5.72E-03
HMMR	2.0	1.22E-03
LDLR	2.1	2.26E-04
DHRS9	2.1	1.73E-02
SEMA7A	2.1	1.51E-03
MEST	2.1	2.12E-02
CXCR4	2.1	5.27E-04
SPDL1	2.1	2.79E-02
F13A1	2.1	1.19E-03
ZNF215	2.1	2.39E-02
KNL1	2.1	2.84E-03
SLC1A2	2.1	2.44E-02
ASGR2	2.1	3.46E-02
GCNT3	2.1	7.34E-03
ITGA2	2.2	1.50E-04
SERPINB8	2.2	2.45E-04
IGHE	2.2	1.73E-02
CFHR2	2.2	4.39E-02
MYBL1	2.2	4.61E-02
STC1	2.2	3.42E-03
HIST1H3A	2.2	9.33E-03
EGF	2.2	1.60E-03
PFKFB3	2.2	5.86E-04

MSMO1	2.2	4.56E-06
GALNT6	2.2	3.33E-06
IDI1	2.2	2.17E-04
FABP3	2.3	1.30E-03
VSTM1	2.3	7.89E-03
FADS2	2.3	1.63E-05
CFAP74	2.3	3.65E-03
MED12L	2.3	6.53E-03
SQLE	2.3	8.68E-06
SPHKAP	2.3	1.77E-02
HP	2.3	1.38E-02
FFAR4	2.3	5.35E-03
C4BPB	2.3	1.66E-04
LOC102161685	2.3	4.59E-04
EBP	2.3	4.62E-06
LIPG	2.3	4.46E-02
TMEM158	2.4	4.39E-02
OSM	2.4	1.97E-02
PTGER1	2.4	2.11E-03
LAMB3	2.4	1.73E-07
ELOVL6	2.4	1.46E-05
IFIT1L1	2.4	2.12E-02
SCNN1D	2.4	4.59E-03
FGF7	2.4	6.47E-04
IGFBP4	2.4	1.00E-05
CXCL14	2.4	2.69E-05
TFF3	2.4	2.78E-03
POU2AF1	2.4	1.75E-04
CACNA1F	2.4	4.37E-02
TNFRSF12A	2.5	1.07E-04
TFRC	2.5	2.06E-05
PPP2R5E	2.5	1.68E-02
HSD11B1	2.5	5.23E-04
ROS1	2.5	2.01E-02
SPHK1	2.5	2.43E-03
ACAT2	2.5	4.51E-08
FCN2	2.5	2.74E-03
SCD	2.5	1.62E-04
GPAT3	2.5	7.03E-05
BAALC	2.6	9.35E-04
PTPRN2	2.6	3.00E-03
MMP9	2.6	6.92E-03
IGH@	2.6	1.24E-02
ME1	2.6	5.20E-03
HHIPL1	2.6	1.78E-04
ETV4	2.7	1.36E-04
SNCA	2.7	4.96E-03
PLA2G3	2.7	4.13E-04
HMGCS1	2.8	3.97E-07
ADGRE1	2.8	3.05E-02
PLAUR	2.8	4.01E-04
WAP1*	2.8	3.59E-02
CLCA1	2.8	6.25E-04
TMEM190	2.8	4.80E-02
NKX2-2	2.8	5.20E-03
CLEC10A	2.9	3.05E-02
LCN2	2.9	6.98E-03
B3GNT6	2.9	5.44E-04
SLC11A1	2.9	2.78E-02
TIMP1	2.9	2.65E-07
INSIG1	2.9	1.64E-08
ALDH3B2	2.9	1.18E-02
TGM3	3.0	3.20E-02
TREM2	3.0	7.72E-03
MMP1	3.1	2.86E-02
SLC12A8	3.1	3.65E-04
ABAT	3.1	1.97E-03
ADAM12	3.1	1.29E-03
PDZK1IP1L	3.1	8.64E-04
ITGB6	3.1	2.10E-07
CCL23	3.1	2.69E-03
TLE6	3.2	5.27E-04
PHGDH	3.3	5.01E-04
PTGS2	3.4	8.84E-04
GABRD	3.4	4.54E-04
CBS	3.5	7.42E-05
SPDEF	3.5	2.29E-06
RN7SL1	3.5	3.19E-02
TNIP3	3.5	3.16E-03
LRP2	3.6	1.26E-02

SHANK2	3.6	4.23E-04
SYNE4	3.6	4.65E-03
SLC7A11	3.7	3.60E-02
P4HA3	3.7	1.39E-04
STRA6	3.8	6.03E-03
MUC2	3.8	1.60E-07
IL1R2	3.8	1.61E-03
NEUROG3	3.8	3.07E-03
TNFRSF11B	3.9	5.64E-05
SERPINB2	3.9	1.24E-02
NPC1L1	3.9	4.56E-02
PSAT1	3.9	3.89E-03
NTRK1	4.0	2.12E-02
COL28A1	4.0	1.89E-04
CAPNS2	4.1	7.03E-05
S100A2	4.2	1.75E-04
HK2	4.2	1.78E-04
MYOM3	4.2	1.12E-05
SAA3	4.5	3.27E-02
PADI4	4.5	4.99E-02
RNF39	4.5	6.41E-07
SERPINA1	4.5	9.03E-04
S100A3	4.5	2.35E-02
AADAC	4.6	3.59E-03
HOXD1	4.6	9.07E-03
ADM2	4.7	7.03E-05
WNT2	4.7	1.78E-04
MPTX	4.9	2.10E-07
UPK1B	5.0	2.36E-06
BPIFB2	5.0	1.64E-03
FAM71E2	5.0	3.52E-02
F3	5.2	1.85E-10
SNORA48	5.2	2.03E-03
CHI3L1	5.2	2.51E-04
PI3	5.3	1.15E-03
NPPC	5.7	3.92E-04
AGR2	5.7	6.15E-07
COL26A1	5.8	6.82E-05
SPINK4	5.8	1.64E-08
BPIFB6	6.0	4.61E-02
GYS2	6.0	4.66E-02
MCHR1	6.1	3.59E-02
SERPINB11	6.3	3.20E-03
IL36A	6.8	6.44E-03
SULT2A1	7.5	5.36E-02
ANXA8	7.6	1.18E-05
TRIM6	7.8	9.11E-03
ADAM20L6	8.1	1.07E-03
CRLF1	8.2	1.09E-08
ADAMTS16	8.3	2.23E-05
IL11	8.3	1.22E-03
PCSK9	9.0	1.11E-11
A3GALT2	9.7	1.38E-02
REG4	9.9	1.95E-09
AQP5	10.2	1.63E-03
IGHG1	10.3	8.11E-04
MMP7	10.5	7.62E-03
S100A8	10.8	2.17E-03
S100A9	11.0	2.03E-03
MMP8	11.4	3.47E-03
MMP13	11.4	2.65E-07
TFF2	12.2	3.25E-02
SLC6A2	13.1	2.14E-02
S100A12	13.4	7.36E-04
PRG4	13.5	3.43E-04
MMP12	13.9	7.63E-10
C1QL2	15.4	1.39E-04
SERPINA3-2	16.2	1.05E-06
ARG1	16.6	8.41E-04
SCGB2A2	17.9	4.89E-06
POU5F1	19.0	3.78E-02
PADI3	19.0	4.69E-03
RHCG	19.8	5.18E-04
SPINK6	21.5	4.16E-04
WNT7A	25.9	4.23E-02
IL4I1L	26.0	1.39E-04
CLEC18A	46.3	9.53E-05
PADI1	51.6	6.25E-04
TFF1	93.2	3.12E-06

Supplemental Table S7: Clustered Up-regulation of Physiology and Immunology DEGs

Gene	Functional Categorization	52 DPI w worms vs Control Fold	EDGE FDR Adj p value		52 DPI w/o worms vs Control Fold	EDGE FDR Adj p value		(52 DPI Inf w/o worms vs w Worms Fold	EDGE FDR Adj p value		(21 DPI Inf vs Control) Fold	EDGE FDR Adj p value
	**Antibacterial Response**											
BPIFB2	Bacterial Binding (12185532); BPI SF	6.7	2.43E-03					5.0	1.64E-03			
BPIFB6	Bacterial Binding (12185532); BPI SF	11.0	4.32E-02					6.0	4.61E-02			
GNLY	Antimicrobial Peptide (15996224); SapB 2 SF	68.8	2.42E-09		132.0	2.98E-11						
HMGN2	Antimicrobial Peptide (16204630); HMG14/17 SF										1.6	1.45E-03
LCN2	Antibacterial Response (15531878, 25398327); Lipocalin SF	3.0	1.80E-02					2.9	6.98E-03			
LTF	Antibacterial Response (8423097); Transferrin SF	52.9	5.95E-03									
LYZ	Antibacterial Response (28804131); LYZ1 SF	13.0	7.77E-03									
NTRK1	Antibacterial Response (25359976); Allergic Inflammation (25389033); Ig SF; LRRCT SF	42.0	2.62E-07		10.6	3.53E-03		4.0	2.12E-02			
OLFM4	(-) Regulator of Antibacterial Responses (20534456, 22844115); OLF SF	2.2	1.48E-02									
PADI4	Antibacterial Responses (20733033); PAD SF	11.0	7.93E-03					4.5	4.99E-02			
PI3	Antimicrobial Peptide Activity (17964057); WAP SF	4.7	1.63E-02					5.3	1.15E-03		4.6	2.55E-08
S100A14	Antibacterial Response (15568027); S100 SF (17625598)										1.8	2.70E-02
SLPI	Antimicrobial Peptide Activity (18075823); WAP SF										4.8	2.22E-11
SPHK1	Antibacterial Response (20634980, 26113114); DAGK Cat SF							2.5	2.43E-03		2.8	3.05E-03
UPK1B	E. coli Attachment factor (9822381); Tetraspanin LEL SF	10.9	1.66E-08					5.0	2.36E-06			
	**Antiparasitic Response**											
AREG	Antiparasitic Response (17170297); Th2-Associated (17170297); PHA02887 SF	2.1	2.09E-02								2.2	8.37E-03
ARG1	Antiparasitic Response (19764983); M2-associated (5879104); Arginase HDAC SF	122.6	4.56E-05					16.6	8.41E-04		30.1	2.28E-07
CHIA	Antiparasitic Response (15618176); Th2-Associated (11553626); GH18 Chitinase Like SF	2.3	3.56E-02									
GATA3	(+) Regulator of Th2-Cell/ILC2 Differentiation (19375293, 23733962); ZnF GATA TF SF				4.4	4.43E-04						
IGHE	Antiparasitic Response (24592267); Ig SF; Ig Heavy Chain Constant Region SF	5.0	2.59E-06		2.3	4.86E-02		2.2	1.73E-02			
IRF4	Antiparasitic Response (20729857); M2-associated (20729857); IRF TF SF	2.1	2.55E-03									
REG3A	Antiparasitic Response (25240019); PRR (16931762)	275.6	2.48E-03		168.9	2.26E-02						
REG4	Antiparasitic Response (25240019); PRR (20692269); CLECT SF	15.8	2.97E-08					9.9	1.95E-09		11.1	8.84E-20
RETNLB	Antiparasitic Responses (15340149); Th2-Associated (12574343); Resistin SF	10.1	2.14E-03									
SERPINB2	Antiparasitic Response (23630350) M2-associated (23355731); SERPIN SF	9.8	2.12E-04					3.9	1.24E-02		16.2	4.90E-32
TFF1	Antiparasitic Response (21569362); Trefoil SF	705.1	2.48E-05					93.2	3.12E-06		25.9	2.62E-06
TFF2	Antiparasitic Response (22329990); Trefoil SF	25.1	3.57E-02					12.2	3.25E-02		37.1	6.93E-10
TFF3	Epithelial Wound Healing (16410243); IL-4 Induced (15004182); Trefoil SF	2.7	2.49E-03					2.4	2.78E-03			
	**Complement Related**											
FCN1	Complement Activation (20375620); PRR (21490156); FReD SF											
FCN2	Complement Activation (19632990); PRR (15331601); FReD SF							2.5	2.74E-03		2.9	9.94E-05
C1QL2	Complement Fixation; BAI3 Ligand (21262840); C1q SF	7.0	4.51E-02					15.4	1.39E-04			
C3	Complement Component; A2M SF; ANATO SF; IsoPREN C2-like SF; MG1 SF; NTR-like SF	1.7	3.10E-02					1.6	3.66E-02			
C4BPA	Complement Component; CCP SF	4.3	1.37E-03									
C4BPB	Complement Component; CCP SF	3.1	5.70E-06					2.3	1.66E-04			
C9	Complement Component; MACPF SF	1.6	3.93E-02					1.5	5.09E-02			
CFB	Alternative Complement Activation Pathway; CCP SF; TSP-1 SF; vWFA SF	2.1	1.92E-02					1.9	2.38E-02			
CFI	Complement factor; SRCR SF	1.7	4.15E-02									
CFHR2	Factor H (fH)-related Protein Family; CCP SF							2.2	4.39E-02			
	**Cytokines, Chemokines, Growth Factors and Receptors**											
ACKR3	CXCL12 Receptor (16107333); CXC Chemokine R SF	1.5	4.95E-02									
AZGP1	Adipocytokine (18663996); MHCI SF; Immunoglobulin SF							2.0	3.20E-02			
BDNF	Cytokine (19348610); Neurotrophic; NGF SF	2.7	1.64E-02									
BMP7	Cytokine; Development; TGFB SF	2.4	6.17E-05					1.9	1.22E-03			
CCL23	CCR1 Ligand (15927850); CCL Chemokine SF	5.1	1.56E-04					3.1	2.69E-03			
CCL28	CCR3 Ligand (10975800); CCL Chemokine SF	2.3	4.92E-03									
CCR1	CCL14 Receptor; CCL16 Receptor; CCL23 Receptor; CCL Chemokine R SF											
CCR2	CCL2 Receptor; CCL7 Receptor; CCL13 Receptor; CCL Chemokine R SF	2.5	2.63E-03					1.9	3.11E-02			
CRLF1	Cytokine (19933857); IL6 Receptor Ligand (19933857); Ig SF; FN3 SF	7.1	3.16E-05					8.2	1.09E-08			
CSF3R	Cytokine Receptor; Lep Receptor Ig SF	2.7	1.81E-02									
CXCL1	CXCR2 Ligand; CXC Chemokine SF	2.9	1.56E-02									
CXCL14	CXCR4 Ligand (28360196); CXC Chemokine SF	3.5	9.85E-08					2.4	2.69E-05		4.4	2.73E-06
CXCL16	CXCR6 Ligand; CXC Chemokine SF										2.0	2.89E-03
CXCL17	GPR35 Ligand (25411203); CXC Chemokine SF										4.5	7.44E-06
CXCL6	CXCR1 Ligand; CXCR2 Ligand; CXC Chemokine SF	5.3	2.39E-05		3.1	1.01E-02						
CXCL8	CXCR1 Ligand; CXCR2 Ligand; CXC Chemokine SF	3.4	5.32E-04									
CXCR1	CXCL1 R; CXCL2 R; CXCL3 R; CXCL6 R; CXCL8 R; CXC Chemokine R SF	2.7	1.71E-02									
CXCR2	CXCL1 R; CXCL2 R; CXCL3 R; CXCL6 R; CXCL8 R; CXC Chemokine R SF	2.8	2.64E-02									
CXCR4	CXCL12 R; CXC Chemokine R SF	1.9	1.45E-02					2.1	5.27E-04			
EGF	Wound Healing (2045428); LY SF; vWFA SF; EGF CA SF; PHA03099 SF	2.5	1.10E-03					2.2	1.60E-03			
IL11	Cytokine (27312790); TH2-Associated (15699166); IL11 SF; IL6 SF	19.4	3.58E-04					8.3	1.22E-03		21.6	6.84E-03
IL13RA2	(-) Regulator of IL-13 Signaling (12642602); Th2-Associated (17697639) IL6Ra-bind SF	3.3	5.25E-02									
IL17C	IL-17RE Ligand (21993849); IL17 SF										5.0	7.15E-03
IL17RD	Positive Regulator of IL17 Receptor Signaling; IL17 R DN SF	2.0	2.03E-02									
IL18	Th1-Associated (10679398); IL1 SF	2.3	7.08E-03									
IL1A	Positive Regulator of Inflammation; IL1 SF	3.6	1.30E-03								2.4	1.55E-03
IL1B	Positive Regulator of Inflammation; IL1 SF	5.5	1.20E-03									
IL1R2	IL1B Decoy Receptor; M2-associated (8786316); I-set SF; Ig SF	10.3	2.46E-06					3.8	1.61E-03		6.1	1.51E-06
IL1RAP	Th1-Associated (10925275); Ig SF; TIR 2 SF	2.9	2.14E-03									
IL26	STAT1 Signaling (14764663); STAT3 Signaling (14764663); Th17-Associated (17676044)	4.1	2.96E-03									
IL33	Th2-Associated (16286016); IL1 SF; IL33 SF	2.0	9.72E-04					1.7	1.40E-02			
IL36A	Positive Regulator of Inflammation (23029241); IL1 SF	22.7	3.90E-04					6.8	6.44E-03		7.2	2.58E-03
IL36B	Positive Regulator of Inflammation (16646978); IL1 SF										30.8	1.02E-02
IL6	Positive Regulator of Inflammation; TH2-Associated (12431386); IL6 SF	6.5	1.29E-04									
MDK	Cytokine; PTN MK N SF										2.8	4.44E-04
NRG1	Cytokine (12204892); Ig SF; EGF CA SF; Neuregulin SF	4.3	8.21E-05								3.8	2.41E-02
OSM	Positive Regulator of Inflammation (12496442); LIF OSM SF	4.4	1.92E-04					2.4	1.97E-02			
OSMR	Type I Cytokine Receptor Family; IL6Ra-bind SF	2.0	1.41E-04					1.6	1.24E-02			
PTN	Cytokine; PTN MK N SF										1.9	1.03E-02
TNFRSF10B	Positive Regulator of Apoptosis (9311998); DD SF; TNFR SF	1.8	3.08E-03									
TNFRSF11A	TNFR SF	1.7	3.61E-02									
TNFRSF11B	Crohn's Disease (15803021); Decoy Receptor; DD SF; TNFR SF	4.6	1.51E-04					3.9	5.64E-05			
TNFRSF12A	Positive Regulator of Apoptosis (21525013); Wound Healing (11728344); TNFR SF	5.0	6.86E-10		2.0	2.26E-02		2.5	1.07E-04			
TNFRSF26	TNFR SF										2.5	2.44E-02
TNFRSF6B	Decoy Receptor; Th2-Associated (14657214); TNFR SF	2.4	4.77E-03									
TNFRSF8	TNFSF8 Receptor; Th2-associated (9036953); TNFR SF										4.0	2.04E-02
TNFSF15	Th1-Associated (14568967); TNF SF	1.8	2.54E-02									
TNFSF18	TNFRSF18/GITR Ligand; TNF SF										3.8	3.17E-02

	**Extracellular Matrix and Remodeling**											
A2ML1	Protease Inhibitor (16298998); A2M SF										16.0	2.14E-03
ADAM12	Metalloprotease; Pep M12B Propep; ZnMc SF; Disintegrin SF; ADAM CR SF	4.2	4.78E-04					3.1	1.29E-03			
ADAM20L2*	Metalloprotease; Pep M12B Propep SF; ZnMc SF; ADAM CR SF	2.9	2.39E-05					2.0	2.60E-03			
ADAM20L6*	Metalloprotease; Pep M12B Propep SF; ZnMc SF; ADAM CR SF	6.1	2.85E-02					8.1	1.07E-03			
ADAM30	Metalloprotease; Pep M12B Propep; ZnMc SF; Disintegrin SF; ADAM CR SF										9.6	8.58E-03
ADAM32	Metalloprotease; Pep M12B Propep; ZnMc SF; Disintegrin SF; ADAM CR SF	2.4	1.08E-02									
ADAM9	Metalloprotease; Pep M12B Propep SF; ZnMc SF; Disintegrin SF; ADAM CR SF	2.6	1.62E-07					1.9	2.26E-04			
ADAMTS1	Metalloprotease; Pep M12B Propep SF; ZnMc SF; ADAM CR SF; TSP 1 SF										2.3	4.06E-04
ADAMTS16	Metalloprotease; Pep M12B Propep SF; ZnMc SF; ADAM CR SF; TSP 1 SF	6.6	2.55E-03					8.3	2.23E-05			
ADAMTS4	Metalloprotease; Pep M12B Propep SF; ZnMc SF; ADAM CR SF; TSP 1 SF	2.6	4.92E-03									
ADAMTS7	Metalloprotease; Pep M12B Propep SF; ZnMc SF; ADAM CR SF; TSP 1 SF	1.6	3.54E-02									
ADAMTS9	Metalloprotease; Pep M12B Propep SF; ZnMc SF; ADAM CR SF; TSP 1 SF	1.7	4.78E-02									
B3GNT7	Keratan Sulfate Biosynthesis (100739508); Galactosyl T SF										6.4	1.16E-05
BGN	Alarmin (24257755); Collagen Fibril Assembly; LRRNT SF							1.8	4.69E-02		2.5	1.81E-02
BLM	Peptidase C1 SF							1.7	3.03E-02			
COL12A1	Collagen SF	2.1	1.41E-04		1.6	4.13E-02						
COL26A1	Collagen SF	7.3	2.71E-04					5.8	6.82E-05			
COL28A1	vWFA SF; Collagen SF; KU SF	4.7	5.24E-04					4.0	1.89E-04		4.9	5.31E-06
COL4A1	Collagen SF	1.7	1.53E-02									
COL5A2	Collagen SF	1.6	1.79E-02									
COL6A3	Collagen SF	1.8	3.31E-02									
COL7A1	Collagen SF; KU SF; vWFA SF	2.1	1.26E-03					1.9	1.47E-03			
COL8A1	C1q SF; Collagen SF	4.1	1.19E-06		2.0	3.85E-02		2.0	1.40E-02			
COMP	ECM Protein; TSPcc SF; EGF CA; SF; TSP 3 SF; TSP C SF											
CTGF	Wound Healing (19128254); IGFBP SF; VWC SF; TSP 1 SF; GHB-like SF	2.1	2.04E-02									
CTSG	Cathepsin; Tryp SPc SF	3.3	1.58E-02		7.2	8.45E-07						
DSE	Dermatan Sulfate Biosynthesis (19687302); DUF4962 SF	1.9	4.61E-03									
ECM1	ECM Protein; ECM1 SF	1.9	4.06E-03					1.8	3.23E-03			
FGF2	Wound Healing (19128254); FGF SF											
FGF7	(+) Regulator of Enterocyte Differentiation (19326389); FGF SF	3.2	4.44E-05					2.4	6.47E-04		2.6	5.67E-04
HPN	Serine Protease (16908524); Hepsin-SRCR SF; Tryp SPc SF											
KRT18	Enterocyte Differentiation Marker (22943429); Intermediate Filament Protein; Keratin SF	2.6	4.34E-05					2.0	1.04E-03		4.7	7.48E-10
KRT80	Intermediate Filament Protein; Keratin SF	1.8	4.15E-02									
LAMB3	Basement Membrane Protein; Laminin N SF; EGF Lam SF	2.5	2.26E-06					2.4	1.73E-07		3.0	6.87E-04
LAMC1	ECM Glycoprotein (22961762); Laminin N SF; Laminin B SF	1.6	2.40E-02									
LOXL4	Collagen Biosynthesis; SRCR SF; Lysyl Oxidase SF	2.2	3.47E-02									
MMP1	Metalloprotease; M1-associated (22880008); HX SF; ZnMc SF; PG-binding-1 SF	9.4	4.44E-05					3.1	2.86E-02		7.3	6.53E-13
MMP12	Metalloprotease; Th2/M2-Associated (12842850, 22880008); HX SF; ZnMc SF	101.0	2.26E-14		7.3	1.09E-03		13.9	7.63E-10		9.7	4.45E-16
MMP13	Metalloprotease; M2-Associated (12842850); HX SF; ZnMc SF	20.2	1.77E-06					11.4	2.65E-07		27.5	9.71E-29
MMP19	Metalloproteinase; HX SF; ZnMc SF; PG-binding-1 SF	1.8	4.54E-02									
MMP20	Metalloproteinase; M2-associated (22880008); HX SF; ZnMc; SF; PG-binding-1 SF	7.4	4.14E-02									
MMP3	Metalloproteinase; M1-associated (22880008); HX SF; ZnMc SF; PG-binding-1 SF	3.5	3.34E-02								4.9	3.31E-10
MMP7	Metalloproteinase; M1-associated (22880008); PG binding 1 SF; ZnMc SF	33.4	3.22E-03					10.5	7.62E-03		53.1	4.09E-05
MMP8	Metalloprotease; M2-associated (26092731); PG binding 1 SF; ZnMc SF; HX SF	27.1	7.94E-03					11.4	3.47E-03			
MMP9	Metalloprotease; M2-associated (26377769); PG Binding 1 SF; ZnMc SF; PT SF; HX SF	8.9	1.02E-08		3.4	3.03E-03		2.6	6.92E-03		9.3	1.00E-02
P4HA2	Positive Regulator of Collagen Biosynthesis (9211872); P4Ha N SF; 2OG-FeII Oxy 3 SF	1.7	5.38E-03					1.6	3.60E-03			
P4HA3	Positive Regulator of Collagen Biosynthesis (14500733); P4Ha N SF; 2OG-FeII Oxy 3 SF	4.4	3.89E-04					3.7	1.39E-04			
PLAUR	Wound Healing (15769664); LU SF	4.6	3.65E-06					2.8	4.01E-04		4.8	6.11E-06
PLOD1	Positive Regulator of Collagen Biosynthesis (25277362); 2OG-FeII Oxy 3 SF											
PRSS27	Wound Re-epithelialization (18948266); Tryp SPc SF											
PSMB7	Proteasome subunit; NTN Hydrolase SF										1.9	7.05E-03
PSMD1	PC Rep SF	1.5	3.59E-02									
PSMD14	26S Proteasome Subunit; MPN SF											
PSMD5	26S Proteasome Subunit; PSMB SF											
SERPINA1	Serine Protease Inhibitor; SERPIN SF	4.7	5.81E-03					4.5	9.03E-04			
SERPINA3	Serine Protease Inhibitor; SERPIN SF											
SERPINA3-2	Serine Protease Inhibitor; SERPIN SF	60.8	2.96E-06					16.2	1.05E-06			
SERPINB1	Serine Protease Inhibitor; SERPIN SF	1.6	3.13E-02					1.6	1.75E-02			
SERPINB10	Serine Protease Inhibitor; SERPIN SF										3.5	2.87E-04
SERPINB11	Serine Protease Inhibitor; SERPIN SF	11.3	9.04E-04					6.3	3.20E-03		8.4	7.38E-04
SERPINB2	Antiparasitic Response (23630350) M2-associated (23355731); SERPIN SF	9.8	2.12E-04					3.9	1.24E-02		16.2	4.90E-32
SERPINB5	Serine Protease Inhibitor; SERPIN SF	1.9	8.72E-03					2.0	9.60E-04		2.2	1.56E-03
SERPINB6	M2-associated (23293084); Serine Protease Inhibitor; SERPIN SF										2.0	1.67E-02
SERPINB7	Extracellular Matrix Degradation (18580857); Serine Protease Inhibitor; SERPIN SF	19.3	1.85E-02									
SERPINB8	Serine Protease Inhibitor; SERPIN SF	2.7	2.47E-05					2.2	2.45E-04		1.8	2.66E-02
SERPINE2	Serine Protease Inhibitor; SERPIN SF	3.1	1.06E-04					2.0	1.59E-02			
SERPINH1	Serine Protease Inhibitor; SERPIN SF	2.0	7.17E-03		1.8	4.70E-02						
SPINK4	Serine Protease Inhibitor; KAZAL FS	6.6	2.11E-06					5.8	1.64E-08		8.1	9.43E-22
SPINK6	Serine Protease Inhibitor; KAZAL FS	46.2	5.38E-03					21.5	4.16E-04			
SULF2	Heparan Sulfate Endosulfatase; Sulfatase SF; DUF3740 SF	1.9	2.64E-02									
TFPI2	Serine Protease Inhibitor; KU FS	1.7	5.42E-03									
TGM3	Transglutaminase SF							3.0	3.20E-02		10.0	3.03E-05
TIMP1	Metallopeptidase Inhibitor; M2-associated (21926236); NTR-like SF	4.3	2.48E-09					2.9	2.65E-07		3.9	2.94E-04
TNC	ECM Protein; FReD SF; FN3 SF	2.9	8.70E-04									
TNN	ECM Protein (10842355)	61.3	4.18E-05		83.7	5.30E-05						
VCAN	ECM Protein; CLECT SF; EGF CA SF; Ig SF; Link Domain SF											
	**Pattern (PR) and Damage-associated Recognition (DR) Receptors**											
BGN	Alarmin (24257755); LRRNT SF							1.8	4.69E-02		2.5	1.81E-02
CD14	PRR (7534618); LRR RI	2.1	1.23E-02					2.0	7.89E-03			
CD163	PRR (18849484); M2b-associated (15491976); SRCR SF	1.9	4.34E-02					1.9	2.29E-02			
CLEC10A	M2-associated (15591125); PRR (15802303); CLECT SF	3.6	2.24E-02					2.9	3.05E-02			
CLEC18A	PRR (26170455); CLECT SF	29.5	7.94E-03					46.3	9.53E-05			
CLEC4D	PRR (25674984); CLECT SF	13.4	4.10E-02									
DDX21	PRR (21703541); GUCT SF; DEXDc SF; EIF-3 Zeta SF	1.5	2.23E-02									
FCN2	Complement Activation (19632990); PRR (15331601); FReD SF							2.5	2.74E-03		2.9	9.94E-05
HMGB1	Alarmin (16966386); HMG-box SF							1.5	1.59E-02			
MPTX	Mucosal pentraxin; LamG SF	5.8	4.12E-06					4.9	2.10E-07		8.3	4.03E-15
SFTPD	PRR (15950451); CLECT SF	4.1	2.73E-02									
SNCA	Alarmin (21747756); Synuclein SF	2.7	1.40E-02					2.7	4.96E-03			
TREM1	PRR (25595774); Ig SF	6.4	4.61E-03									
TREM2	PRR (12847223); Ig SF				-2.5	4.89E-02		3.0	7.72E-03			
	**Mast Cell Associated**											

SRGN	Mast Cell Secretory Granule Protein (15231821); Serglycin SF	1.8	2.61E-03	2.1	1.18E-05				
MCPT3*	Mast Cell Protease; Tryp SPc SF	7.3	2.80E-02						
TPSB2	Mast Cell Protease; Tryp SPc SF							2.8	2.68E-02
HDC	Biosynthesis of Histamine; Mast Cell Associated; AAT I SF			2.7	2.39E-02				
KIT	(+) Positive Regulator of Mast Cell Development (25062998); Ig SF PKC-like SF			2.3	1.73E-02				
	**M2-Macrophage Associated**								
ARG1	Antiparasitic Response (19764983); M2-associated (5879104); Arginase HDAC SF	122.6	4.56E-05			16.6	8.41E-04	30.1	2.28E-07
CD163	PRR (18849484); M2b-associated (15491976); SRCR SF	1.9	4.34E-02			1.9	2.29E-02		
CHIA	Antiparasitic Response (15618176); Th2-Associated (11553626); GH18 Chitinase Like SF	2.3	3.56E-02						
CLEC10A	M2-associated (15591125); PRR (15802303); CLECT SF	3.6	2.24E-02			2.9	3.05E-02		
F13A1	Coagulation; M2-associated (19105661); Transglutaminase SF	4.4	1.41E-09	2.1	7.07E-03	2.1	1.19E-03		
IL1R2	IL1B Decoy Receptor; M2-associated (8786316); I-set SF; Ig SF	10.3	2.46E-06			3.8	1.61E-03	6.1	1.51E-06
IRAK3	IL-1 Receptor-associated Kinase; M2-associated (2109822); DD SF; PKC-like SF								
IRF4	Antiparasitic Response (20729857); M2-associated (20729857); IRF TF SF	2.1	2.55E-03						
MMP12	Metalloprotease; Th2/M2-Associated (12842850, 22880008); HX SF; ZnMc SF	101.0	2.26E-14	7.3	1.09E-03	13.9	7.63E-10	9.7	4.45E-16
MMP13	Metalloprotease; M2-Associated (12842850); HX SF; ZnMc SF	20.2	1.77E-06			11.4	2.65E-07	27.5	9.71E-29
MMP20	Metalloproteinase; M2-associated (22880008); HX SF; ZnMc; SF; PG-binding-1 SF	7.4	4.14E-02						
MMP8	Metalloprotease; M2-associated (26092731); PG binding 1 SF; ZnMc SF; HX SF	27.1	7.94E-03			11.4	3.47E-03		
MMP9	Metalloprotease; M2-associated (26377769); PG Binding 1 SF; ZnMc SF; PT SF; HX SF	8.9	1.02E-08	3.4	3.03E-03	2.6	6.92E-03	9.3	1.00E-02
ODC1	M2-associated (19726720); Ornithine Metabolism; Polyamine Metabolism; PLPDE III SF			-1.5	1.27E-02	1.6	4.12E-03		
OSM	M2a-associated (26801095); LIF OSM SF	4.4	1.92E-04			2.4	1.97E-02		
PTGS1	Prostaglandin E2 Biosynthesis; M2-associated (17082649); EGF CA; An Peroxidase-like SF	2.3	9.54E-06	1.7	6.80E-03				
PTGS2	Prostaglandin H2 Biosynthesis; M2-associated (28684424); EGF CA; An Peroxidase-like SF	6.5	8.39E-06			3.4	8.84E-04	4.0	1.30E-07
SERPINB2	Antiparasitic Response (23630350) M2-associated (23355731); SERPIN SF	9.8	2.12E-04			3.9	1.24E-02	16.2	4.90E-32
SERPINB6	M2-associated (23293084); Serine Protease Inhibitor; SERPIN SF							2.0	1.67E-02
SLC7A2	Arginine Transport (8954799), M2-associated (16670299); Solute Carrier SF	1.9	4.15E-02						
TGM2	M2-associated (23293084); Transglutaminase SF	2.2	3.66E-05					2.2	7.15E-03
TIMP1	Metallopeptidase Inhibitor; M2-associated (21926236); NTR-like SF	4.3	2.48E-09			2.9	2.65E-07	3.9	2.94E-04
TREM2	PRR (12847223); Ig SF			-2.5	4.89E-02	3.0	7.72E-03		
	**Mucus and Fluid Production**								
A3GALT2	Galactosyltransferase; Glyco Tranf GTA Type SF	40.6	5.54E-03			9.7	1.38E-02		
AGR2	Mucin Biosynthesis (24942678); Thioredoxin-like SF	6.5	1.79E-05			5.7	6.15E-07	5.6	1.73E-11
AQP3	Glycerol Transport (16079275); Water Transport (16079275); MIP SF								
AQP5	Water Transporter (9195910); MIP SF	33.5	4.54E-04			10.2	1.63E-03		
AQP9	Water Transport; Glycerol Transport (19096786); MIP SF	5.3	1.56E-02						
B3GNT6	Mucin Biosynthesis (26274979); Galactosyl T SF	3.0	2.31E-03			2.9	5.44E-04	2.3	7.21E-03
CLCA1	Chloride Ion Transport (8769986); Th2-associated (17898169); CLCA N SF; vWFA SF	3.2	1.07E-03			2.8	6.25E-04	3.5	9.80E-09
FUT2	Fucosyltransferase (24062455); Th2-associated (12652082); O-FucT-like SF							3.1	1.38E-03
FUT8	Mucin Biosynthesis (28122822); O-FucT-like SF; SH3 SF	1.6	5.97E-03						
GALNT1	Mucin Biosynthesi; Glyco Tranf GTA-type SF; RICIN SF	1.5	4.20E-02						
GALNT6	Mucin Biosynthesis (20215525); Glyco Tranf GTA-type SF	1.7	1.20E-02			2.2	3.33E-06	2.5	5.55E-03
GALNT7	Mucin Biosynthesis Glyco Tranf GTA-type SF; RICIN SF	1.9	5.18E-04			1.7	2.49E-03		
GCNT3	IL-13 Induced (17303715); Mucin Biosynthesis (20816165); Branch SF	2.7	1.21E-03			2.1	7.34E-03	2.4	4.06E-04
MUC2	Secreted Gel Forming Mucin (24942678); Th2-associated (14594655); Mucin SF	5.2	7.10E-08			3.8	1.60E-07	6.3	2.16E-16
	**Miscellaneous**								
ANXA8	Anticoagulant (2530088); Annexin SF	21.8	2.21E-07			7.6	1.18E-05	34.8	6.97E-40
CHI3L1	GH18 Chitinase Like SF	13.9	2.38E-06			5.2	2.51E-04	6.4	1.03E-13
CHI3L2	GH18 Chitinase Like SF	6.0	7.37E-04					4.2	4.97E-04
CHIT1	GH18 Chitinase Like SF	24.2	1.65E-02						
CLTC	(-) Regulator of NFk-B Activation (21364927); Clathrin SF	1.5	3.32E-02						
FCGBP	Ig Receptor; VWD SF; C8 SF; TIL SF; VWC SF	2.3	2.08E-02						
GJA1	Epithelial Wound Repair (19528242); Connexin SF; Connexin CCC SF; Connexin 43 SF	2.4	6.11E-05						
GZMA	Tryp SPc SF			3.0	4.54E-04				
GZMB	Tryp SPc SF	5.5	2.79E-04	11.2	8.54E-08				
IGH@	Ig SF; Ig Heavy Chain Constant Region SF	5.7	2.70E-05			2.6	1.24E-02		
IGHG1	Ig SF; Ig Heavy Chain Constant Region SF	29.1	1.93E-04			10.3	8.11E-04	11.1	3.80E-06
IGJ	Immunoglobulin J polypeptide; Ig J-Chain SF	1.8	2.63E-02						
KLRJ1*	CLECT SF	10.0	5.18E-04	9.6	1.59E-03				
MYOF	Membrane Regeneration and Repair; C2 SF; FerA SF; FerB SF; DysFN SF	2.2	3.03E-05			1.9	1.39E-04		
NEUROG3	(+) Regulator of Enteroendocrine Cell Differentiation (17706959); HLH SF	5.7	1.61E-03			3.8	3.07E-03		
NLRC3	Inflammasome Associated (21191067); LRR RI SF; P-loop NTPase SF			2.5	1.97E-02				
NTRK1	Allergic Inflammation (25389033); IL-4 Induced; Ig SF; LRRCT SF	42.0	2.62E-07	10.6	3.53E-03				
PADI1	(-) Regulator of Inflammation (18710930); PAD SF	296.4	3.51E-03			51.6	6.25E-04		
PADI3	PAD SF	55.7	1.75E-02			19.0	4.69E-03		
PRG4	HX SF; Somatomedin B SF	10.7	1.39E-02			13.5	3.43E-04		
S100A10	Inflammation (16373665); S100 SF								
S100A11	RAGE Ligand (18331229); S100 SF					1.5	3.35E-02		
S100A12	RAGE Ligand (18331229); Inflammation (12740341); S100 SF	38.4	4.48E-04			13.4	7.36E-04	12.8	4.09E-05
S100A2	Eosinophil Chemoattractant (8607858); S100 SF	7.4	9.54E-06			4.2	1.75E-04	10.0	3.03E-27
S100A3	S100 SF	8.0	6.29E-03			4.5	2.35E-02		
S100A6	RAGE Ligand (18331229); S100 SF	1.6	3.06E-02			1.6	1.55E-02		
S100A8	RAGE Ligand; Inflammation (12645005); S100 SF	38.3	5.18E-04			10.8	2.17E-03	8.2	1.02E-02
S100A9	RAGE Ligand; Inflammation (12645005); S100 SF	44.8	2.59E-04			11.0	2.03E-03	9.8	1.83E-03
SCGB2A2	Lipophilin SF Secretoglobin SF; Uteroglobin SF	105.1	3.14E-07			17.9	4.89E-06		
SCGB3A1	IL-4 Induced (16237061); Lipophilin SF Secretoglobin SF; Uteroglobin SF	8.8	1.90E-02						
SMPDL3B	(-) Regulator of TLR Signaling (26095358); MPP SF							2.0	1.67E-02
SOCS3	TH2-associated (11907070); Th17-associated (16698929)	2.5	1.07E-03						
THBD	Th2 Associated (189478630); CLECT SF; EGF CA SF; Tme5 EGF-like SF	1.8	2.09E-02			1.9	3.32E-03		
TIGIT	(-) Regulator of DC Function (19011627); TFH Cell Associated (19197944); Ig SF	2.5	3.55E-03	2.8	3.39E-04				
TNIP3	(-) Regulator of TLR4 Signaling (17088249); Tape Meas Lam C SF; BAN SF	7.3	4.56E-05			3.5	3.16E-03	4.5	5.90E-06
TRAIP	(-) Regulator of TLR Signaling (14676304); RING Ubox SF					1.9	5.08E-03		
VSTM1	(-) Regulator of Monocyte/Neutrophil Function (20375307, 23436183); Ig SF	2.3	1.95E-02			2.3	7.89E-03		
WNT7A	Wound healing (15802269); WNT SF								
XDH	(+) Regulator of Inflammation (h) (20632067); Fer2 2 SF; CO Deh Flav C SF; Ald Xan Dh C2 SF	22.8	3.03E-02						
	**Neurotransmitters and Receptors**								
ABAT	GABA aminotransferase; AAT I SF	2.7	2.61E-02			3.1	1.97E-03	3.2	1.73E-05
AVPR1B	Arginine Vasopressin Receptor; GPCR SF	62.6	5.42E-03						
BDKRB1	Bradykinin Receptor; GPCR SF	4.0	1.65E-03					4.6	1.70E-02
DOK7	Postsynaptic Differentiation; PH-like SF								
DRD2	Dopamine receptor D2; Neurotransmitter Receptor; GPCR SF								
GABRD	GABA Receptor; Neurotransmitter Receptor; Neur Chan LBD SF; Neur Chan Memb SF	2.5	4.65E-02			3.4	4.54E-04		
GABRP	GABA Receptor; Neur Chan LBD SF Neur Chan Memb SF							15.7	1.59E-03

GPR83	Neuropeptide Receptor (27117253); Treg Associated (16785516); GPCR SF											
HTR1B	Neurotransmitter Receptor; Serotonin Receptor; GPCR SF										11.8	2.40E-02
MCHR1	Neuropeptide Receptor; GPCR SF	17.9	7.59E-03									
P2RX4	Purinergic Receptor; Inflammasome Associated (24409119); P2X Receptor SF	1.5	4.22E-02					1.6	4.88E-03		2.4	8.14E-04
SLC35E1	Sodium-dependant GABA Transport; Solute Carrier SF											
SLC6A13	Sodium-dependant GABA Transport; Solute Carrier SF	6.5	1.54E-02									
SLC6A2	Sodium-dependent Noradrenaline Transport (2008212); Solute Carrier SF							13.1	2.14E-02		18.7	1.53E-02
SV2B	Neurotransmitter Release; MF SF											
	**Oxidative Stress Response**											
GSTA1	Glutathione S-Transferase; Thioredoxin-like SF; GST C Family SF										2.7	4.73E-02
GSTA2	Glutathione S-Transferase; Thioredoxin-like SF; GST C Family SF										5.4	4.30E-03
GSTP1L2*	Glutathione S-Transferase; Thioredoxin-like SF; GST C Family SF										6.9	3.53E-02
PDIA4	Superoxide Generation (1318337); Thioredoxin-like SF	2.3	1.15E-04					1.7	7.74E-03			
PRDX4	Oxidative Stress Response (12080185); Thioredoxin-like SF	2.5	3.41E-05					1.9	1.03E-03			
	**Prostaglandin and Leukotriene**											
ALOX5AP	MAPEG SF				2.0	3.09E-02						
LTC4S	Cysteinyl Leukotriene Biosynthesis; Th2-Associated (11136826); MAPEG SF				2.8	1.24E-02						
PTGER1	Prostaglandin E2 Receptor; Th1 Associated (17967902); GPCR SF	2.3	1.21E-02					2.4	2.11E-03			
PTGER2	Prostaglandin E2 Receptor; Inflammation (15075356); GPCR SF	1.7	1.84E-03									
PTGS1	Prostaglandin E2 Biosynthesis; M2-associated (17082649); EGF CA; An Peroxidase-like SF	2.3	9.54E-06		1.7	6.80E-03						
PTGS2	Prostaglandin H2 Biosynthesis; M2-associated (28684424); EGF CA; An Peroxidase-like SF	6.5	8.39E-06					3.4	8.84E-04		4.0	1.30E-07
PLA2G4A	Prostaglandin Metabolism; C2 SF; Patatin and cPLA2 SF	1.7	3.82E-02									
	**Surface Receptors and Ligands**											
ADGRE1	Th2-associated (20625511); GPCR SF							2.8	3.05E-02			
ADGRE2	(+) Regulator of Macrophage Differentiation (28421075); GPCR SF	2.1	2.00E-02									
ADGRG6	(+) Regulator of Angiogenesis (25217645); GPCR SF	1.9	2.65E-03					1.6	2.36E-02			
ADORA1	(+) Regulator of Inflammation (8360491); GPCR SF											
ASGR2	CLECT SF							2.1	3.46E-02			
CD24	(+) Regulator of T Cell Activation (15607804); SLAM SF Immunoglobulin SF	1.8	4.34E-02					1.9	6.90E-03			
CD244	(+) Regulator of T Cell Activation (15607804); SLAM SF				2.4	1.29E-02						
CD69	(+) Regulator of Inflammation (16983725); CLECT SF	1.8	3.37E-02		2.1	4.85E-03						
CD79A	B Cell Maturation Marker (15928681); Ig SF	2.2	1.05E-02									
CDH1	(+) Regulator of Paneth Cell Differentiation (21179475); Cadherin SF	2.1	1.78E-04					1.6	8.68E-03		2.2	3.28E-03
F13A1	Coagulation; M2-associated (19105661); Transglutaminase SF	4.4	1.41E-09		2.1	7.07E-03		2.1	1.19E-03			
F2	Coagulation; Th2-Associated (16081830); GPCR SF											
F2RL2	Coagulation; Positive Regulator of Inflammation (12805069, 18264801); GPCR SF	1.7	1.57E-02									
F3	Coagulation; FN3 SF; Tissue Factor SF	8.2	7.74E-11					5.2	1.85E-10		3.1	4.09E-05
ITGA1	T Cell Activation Marker; vWFA SF; VCBS SF; Integrin Alpha2 SF	1.9	3.71E-02									
ITGA2	Adhesion Molecule; VCBS SF; vWFA SF; Integrin Alpha2 SF	2.6	3.66E-05					2.2	1.50E-04			
ITGA8	Integrin; VCBS Superfamily	2.2	1.15E-04									
ITGAV	Integrin; FG-GAP Superfamily; Integrin Alpha2 Superfamily	1.7	7.96E-03									
ITGB1	Integrin; vWFA Superfamily; Integrin B Cyt Superfamily; Integrin B tail Superfamily	1.5	1.80E-02									
ITGB6	(+) Regulator of Inflammation (10025398); Integrin Beta SF; vWFA SF	5.4	8.74E-11					3.1	2.10E-07		2.6	7.63E-03
NECTIN1	Adhesion Molecule; Ig SF							1.5	3.13E-02			
SELE	Adhesion Molecule; CLECT Superfamily; EGF-CA Superfamily; CCP Superfamily				2.5	3.53E-02						
SEMA7A	(-) Regulator of T Cell Activation (16713976); Sema SF; PSI SF	3.0	1.23E-05					2.1	1.51E-03		2.1	2.41E-02
	**Transcription Factors**											
ARNTL2	HLH SF; PAS SF	3.8	6.51E-06		2.1	3.38E-02		1.8	5.25E-02			
ATF4	bZIP SF	1.6	1.20E-02									
ATF5	bZIP SF	2.5	2.26E-02									
ATOH1	(+) Regulator of Enterocyte/Enteroendocrine Cell Differentiation (17570220, 15044355); HLH SF							1.7	2.37E-02		2.0	2.41E-02
CDX1	(+) Regulator of Enterocyte Differentiation (10579974); Caudal Act SF; Homeodomain SF										2.7	1.73E-03
CEBPB	Th2-Associated (16585579); bZIP SF	2.1	3.29E-03									
E2F7	E2F TDP SF	1.7	5.49E-02					1.8	1.34E-02			
EPAS1	HIF-1 SF				1.5	1.29E-02						
ETV4	ETS SF	2.9	3.43E-04					2.7	1.36E-04		3.1	4.03E-02
ETV5	ETS SF	1.9	2.26E-02					1.8	1.26E-02			
FOS	bZIP SF	2.5	2.09E-02									
FOXA1	DAZAP2 SF; FH SF; HNF C SF	2.0	6.53E-03					1.6	3.80E-02		1.8	3.01E-02
GATA2	(+) Regulator of Eosinophil Differentiation (9279013); ZnF GATA SF				2.0	5.31E-02						
GATA3	(+) Regulator of Th2-Cell/ILC2 Differentiation (19375293, 23733962); ZnF GATA TF SF				4.4	4.43E-04						
HIF1A	Transcription Factor; HLH SF; PAS SF; HIF-1 SF	2.4	9.56E-06					1.7	3.73E-03			
HOXB13	Development; Homeodomain SF										132.7	1.59E-02
HOXD1	Development; Homeodomain SF	7.4	6.83E-03					4.6	9.07E-03			
IRF4	Antiparasitic Response (20729857); M2-associated (20729857); IRF TF SF	2.1	2.55E-03									
MAZ	(+0 Regulator of Acute Phase Response (16888022); zf-H2C2 2 SF; RING SF											
MKX	Development; Homeodomain SF; IRO SF	2.3	2.40E-02									
MYBL1	SANT SF; Cmyb C SF							2.2	4.61E-02			
MYBL2	(+) Regulator of Enterocyte Differentiation (20857481); SANT SF; Cmyb C SF							1.5	3.16E-02		2.4	3.34E-03
NFIL3	(+) Regulator of ILC Development (27705792); bZIP SF	2.4	3.14E-09		1.7	1.50E-04						
NKX2-2	(+) Regulator of Enteroendocrine Cell Differentiation (18022152); Homeodomain SF	3.3	5.22E-03					2.8	5.20E-03			
POU2AF1	Transcriptional Co-activator; PD-C2-AF1 SF	3.3	9.54E-06					2.4	1.75E-04			
POU5F1	Transcription Factor; Homeodomain SF							19.0	3.78E-02			
PRDM1	(+) Regulator of Enterocyte Reprogramming (21670299); SET SF	1.6	2.16E-02									
RUNX3	(+) Regulator of ILC1/ILC3 Development (26414766); Runxl SF				2.2	5.12E-03						
SOX9	(+) Regulator of Paneth Cell Differentiation (17698607); Sox N SF; HMG-box SF	1.7	1.52E-02									
SP6	zf-H2C2 2 SF; zf-H2C2 2 SF; COG5048 SF										9.3	1.01E-02
SPDEF	(+) Regulator of Goblet Cell/Paneth Cell Differentiation (19786015, 19549527); ETS SF	4.4	3.89E-06					3.5	2.29E-06		3.0	1.13E-04
TLE6	Transcriptional Co-repressor (17624551); WD40 SF	3.0	6.63E-03					3.2	5.27E-04		4.4	1.34E-02
XBP1	B Zip1 SF	2.0	2.73E-04					1.7	3.59E-03			
ZNF215	Transcriptional Co-repressor; SCAN SF; KRAB A-box SF; zf-H2C2 2 SF; zf-C2H2 SF							2.1	2.39E-02			

Supplemental Table S8: Clustered Down-regulation of Physiology and Immunology DEGs

Gene	Functional Categorization	52 DPI w worms vs Control Fold	EDGE FDR Adj p value	52 DPI w/o worms vs Control Fold	EDGE FDR Adj p value	(52 DPI Inf w/o worms vs w Worms Fold	EDGE FDR Adj p value	(21 DPI Inf vs Control) Fold	EDGE FDR Adj p value
	**Antigen Processing and Presentation**								
ADAM23	Positive Regulator of Antigen Presentation (27317750); ZnMc SF								
B2M	Positive Regulator of Surface Expression of MHC Class I (16181333); Ig SF	-2.0	5.66E-04						
CD74	MHC Chaperone (22306692); MHC2-interact SF	-2.5	2.92E-04			-2.0	7.72E-03		
CIITA	Positive Regulator of MHC Class I and II Expression (14678199, (11486010); LRR SF					-1.7	4.54E-02		
CLEC4F	Glycolipid Antigen Presentation (23762286)	-4.6	4.26E-02						
CTSD	Antigen Processing and Presentation (16920965); Pepsin Retropepsin-like SF	-2.1	3.64E-04			-1.6	1.68E-02		
ERAP2	Antigen Processing and Presentation (12799365); Type 2 IFN Induced (12799365); GluZincin SF	-1.6	4.60E-02						
LGALS3BP	Antigen Processing and Presentation (15231701); SRCR SF	-1.9	3.18E-02						
PLCB2	(+) Regulator of B Cell Activation (20871625); PH-like SF; EF-hand-like SF; PI-PLCc GDPD SF SF; C2 SF	-2.0	1.74E-03						
PSMB10	Immunoproteasome Protease (9551082); Ntn Hydrolase SF; Pr Beta C SF	-1.8	3.20E-03						
PSMB8	Immunoproteasome Protease; Ntn Hydrolase SF; Pr Beta C SF	-1.6	2.91E-02						
PSMB9	Immunoproteasome Protease; Ntn Hydrolase SF; Pr Beta C SF	-1.8	9.65E-03						
PSME1	Antigen Processing and Presentation (12200048); PA28 Alpha SF; PA28 Beta SF	-1.6	5.26E-03						
SLA-11	MHC Class I SF	-2.3	1.08E-04			-1.6	4.46E-02		
SLA-2	MHC Class I SF					-1.7	5.02E-02		
SLA-3	MHC Class I SF	-2.3	1.89E-04					-1.8	3.72E-02
SLA-8	MHC Class I SF	-1.6	1.98E-02			-1.6	4.54E-02		
SLA-DMA	MHC Class II SF	-2.1	4.61E-03					-1.9	1.99E-02
SLA-DMB	MHC Class II SF	-2.2	8.10E-04			-1.7	2.70E-02	-2.4	3.61E-04
SLA-DOA	MHC Class II SF	-2.4	3.71E-03						
SLA-DQA1	MHC Class II SF	-2.3	3.27E-03			-2.0	1.33E-02		
SLA-DQB1	MHC Class II SF	-2.3	1.13E-02					-2.5	1.55E-03
SLA-DRA	MHC Class II SF	-2.4	1.06E-03			-2.1	5.53E-03		
SLA-DRB1	MHC Class II SF	-2.2	5.60E-03			-2.0	1.25E-02		
TAP1	Peptide Transporter; ABC Membrane SF	-1.9	5.73E-03			-1.7	2.23E-02		
TAP2	Peptide Transporter; ABC Membrane SF	-1.6	1.31E-02						
	**Cytokines, Chemokines and Receptors**								
ADIPOQ	Adipocytokine; C1q SF	-4.2	5.00E-04					-2.2	4.06E-03
ANGPTL4	Adipocytokine; FReD SF	-2.9	1.19E-03	-2.0	4.70E-02				
BMP3	Bone Morphogenetic Protein (BMP) Family; TGFB SF							-2.5	2.94E-03
CCL2	CCR2 Ligand; CCL Chemokine SF							-2.0	1.38E-03
CCL21	CCR7 Ligand; CCL Chemokine SF	-1.7	4.86E-02						
CCL3L1	CCL Chemokine SF							-5.6	2.84E-03
CCL3L2	CCL Chemokine SF	-5.4	6.08E-03					-10.6	5.90E-03
CCL4	CCR5 Ligand; CCL Chemokine SF	-2.4	1.16E-02						
CCL8	CCR1 Ligand; CCL Chemokine SF	-2.6	7.80E-03					-2.8	4.24E-02
CCR5	CCL3 Receptor; CCL4 Receptor; CCL5 Receptor; CCL Chemokine Receptor SF	-1.8	5.37E-03						
CCR9	CCL25 Receptor; CCL Chemokine Receptor	-3.0	5.47E-02					-5.8	1.14E-02
CNTFR	Cytokine Receptor (10812968); FN3 SF; Ig SF	-3.5	4.40E-04			-2.9	3.11E-03		
CSF1	Cytokine; Positive Regulator of Macrophage Development.	-1.5	3.58E-02			-1.5	1.68E-02		
CSF1R	CSF1 Receptor	-1.7	3.70E-03			-1.5	4.54E-02		
CSF2RB	GM-CSF/IL-3/IL-5 receptor Common Beta-Chain; Erythropoietin Receptor SF; FN3 SF; IL6Ra-bind SF					-1.5	4.36E-02		
CXCL10	Type 2 IFN Induced; CXCR3 Ligand; CXC Chemokine SF	-3.4	3.22E-02			-4.4	7.18E-03		
CXCL11	Type 2 IFN Induced; CXCR3 Ligand; CXC Chemokine SF	-4.8	3.33E-02						
CXCL12	CXCR4 Ligand; CXC Chemokine SF	-2.7	6.31E-04			-2.8	2.48E-04	-2.3	5.03E-03
CXCL13	CCL10 Ligand; CXCR5 Ligand; CXC Chemokine SF							-5.8	5.77E-08
CXCL9	Type 2 IFN Induced; CXCR3 Ligand; CXC Chemokine SF	-7.5	6.02E-04			-5.7	3.65E-03	-6.1	7.08E-08
CXCR3	CXCL9 Receptor; CXCL10 Receptor; CXCL11 Receptor; CXCL Chemokine Receptor SF	-2.2	1.38E-02						
EBI3	FN3 SF	-2.0	4.52E-02						
FLT3LG	Flt3 Lig SF	-1.8	3.71E-03						
GDF5	Bone Morphogenetic Protein (BMP) Family; TGFB SF	-3.8	1.25E-02	-3.3	1.09E-03				
IGF2	IIGF-like SF	-3.2	5.66E-04			-2.2	2.80E-02		
IL10RA	Cytokine Receptor; Interferon-binding SF	-1.9	8.53E-04			-1.5	3.17E-02		
IL12B	Cytokine; IL12p40 SF	-4.7	1.02E-02						
IL12RB2	IL-12 Receptor; Th1-Associated (10666772); Lep Receptor Ig SF	-2.3	4.34E-02						
IL15	TH1-Associated (12759422); IL15 SF; IL2G Receptor Binding SF							-2.7	3.09E-02
IL17RA	IL-17 Receptor; TIR 2 SF	-1.5	1.36E-02						
IL2RB	IL2 Receptor; IL15 Receptor; FN3 SF					-1.7	2.69E-02		
IL34	Cytokine; IL34 SF	-2.5	3.63E-04			-2.1	4.12E-03		
KIT	Ig SF; PKC-like SF							-2.3	2.04E-02
LIFR	LIF Receptor; FN3 SF	-2.1	4.05E-04			-1.7	7.18E-03	-2.6	3.03E-05
LTBP3	TGFB Binding Protein EGF CA SF	-1.6	4.59E-02						
LTBP4	TGFB Binding Protein EGF CA SF	-2.2	9.11E-05			-1.8	5.61E-03		
MDK	Cytokine; (+) Regulator of Inflammation (30217121); PTN MK N SF	-2.0	1.75E-02						
PDGFC	Angiogenesis (20566880); CUB SF; PDGF SF							-2.3	2.70E-03
PF4	CXC Chemokine SF							-3.2	1.71E-02
PGF	PDGF SF	-1.9	4.93E-02						
RARRES2	Adipocytokine (17767914)	-1.6	4.83E-02						
THNSL2	Cytokine (19877052); Trp-synth-beta II SF	-2.3	1.15E-03						
TNFSF11	Positive Regulator of Microfold Cell Differentiation (22778137); TH1-Associated (14607948); TNF SF							-4.0	1.83E-03
TNFSF13B	(+) Regulator of B Cell Activation (16797504); Th1-Associated (15843552); TNF SF							-2.5	2.69E-04
XCL1	Th1-associated (14573634); XCR1 Receptor Ligand	-5.2	1.96E-06			-2.7	5.72E-03	-3.3	3.63E-03
	**Extracellular Matrix and Remodeling**								
ADAM1A	Metalloprotease;; Pep M12B Propep SF; ZnMc SF; Reprolysin SF; Disintegrin SF; ADAM CR SF							-52.9	1.87E-02
ADAMTS19	Metalloprotease; TSP 1 SF							-2.6	1.33E-02
ADAMTS5	Metalloprotease; Pep M12B Propep SF; ZnMc SF; TSP 1 SF; ADAM Spacer1 SF							-2.2	1.84E-02
ADAMTS8	Metalloprotease; TSP 1 SF					-1.9	1.07E-02		
ADAMTSL5	Metalloprotease; TSP 1 SF	-2.1	3.76E-02						
ANPEP	Aminopeptidase; GluZincin SF	-5.2	3.11E-04	-2.5	4.13E-02				
ASTL	Metalloproteinase (15087446); ZnMc SF	-37.2	2.34E-02					-49.0	2.75E-02
BSG	Extracellular Matrix Remodeling (26084217); Treg-associated (23665204); Ig SF; KU SF					-1.5	2.27E-02		
COL14A1	Collagen SF							-1.7	3.72E-02
COL22A1	Collagen SF							-56.8	1.34E-02
COL25A1	Collagen SF							-5.6	7.28E-04
COL4A3	Collagen SF	-2.4	4.44E-02						
COL4A6	Collagen SF	-1.6	3.19E-02			-1.6	4.50E-02		
COL6A5	Collagen SF							-2.0	1.50E-02
COL6A6	Collagen SF	-7.8	1.07E-07			-5.7	3.03E-06	-5.7	3.51E-08
CST3	Cysteine Protease Inhibitor; Extracellular Matrix Formation/Degradation (19468292); Cystatin (CY) SF					-1.9	7.80E-03	-2.3	3.93E-04
CSTA	Protease Inhibitor; CY SF	-1.9	2.56E-02						
CTBS	Asn-linked Glycoprotein Catabolism; GH18 Chitinase-like SF	-3.6	8.38E-03			-2.9	4.10E-02		

CTSZ	Cathepsin; Peptidase C1 SF	-1.8	4.19E-03									
GALNS	Extracellular Matrix Degradation (18285341); Sulfatase SF; DUF4976 SF	-1.6	1.45E-02									
HAPLN4	Extracellular Matrix (12663660); Ig SF; Link Domain SF	-8.4	9.20E-06					-1.8	5.64E-03			
HAS3	Hyaluronan Synthesis; Glyco Tranf GTA-type SF	-8.4	9.20E-06					-5.2	5.01E-04		-4.0	4.07E-05
HGSNAT	Extracellular Matrix Degradation (21957468); DUF5009 SF;	-1.6	1.40E-02									
HPSE	Extracellular Matrix Degradation (12213822); Heparanase; Glyco hydro 79n SF	-1.6	1.75E-02					-1.5	5.25E-02			
HS3ST3A1	Glycosaminoglycan Biosynthesis (9988767); Extracellular Matrix (9988767); Sulfotransfer 3 SF							-2.5	4.16E-02			
ITIH5	Extracellular Matrix Stabilization (25809190); VIT SF; vWFA SF; ITI HC C SF	-1.9	1.46E-02									
MMP11	Metalloprotease; ZnMc SF	-2.0	7.40E-04									
MMP24	Metalloprotease; ZnMc SF	-1.8	2.06E-02									
MMP25	Metalloprotease; ZnMc SF	-1.8	1.15E-02									
PCSK6	Metalloprotease; Peptidases S8 S53 SF; P Proprotein SF; FU SF; PLAC SF	-2.3	1.07E-05					-1.6	5.72E-03			
PRSS12	Serine Protease; SRCR SF; Tryp SPc SF; KR SF							-2.7	9.44E-04		-2.5	5.62E-03
THSD4	Metalloprotease; TSP 1 SF; ADAM Spacer 1 SF; PLAC SF	-1.5	2.95E-02									
TMPRSS13	Serine Protease; Tryp SPc SF	-2.8	1.67E-02									
TMPRSS2	Serine Protease (20382709); SRCR SF	-2.4	1.23E-04					-1.8	1.60E-02			
TMPRSS4	Serine Protease (20382709); SRCR SF	-2.1	4.56E-05					-1.6	6.34E-03			
UGDH	Glycosaminoglycan Biosynthesis (9737970); F420 Oxidored SF; UDPG MGDP DH SF	-1.6	2.77E-03									
VSIR	Extracellular Matrix Formation (20666777); (-) Regulator of of T Cell Function (24691993); B7 SF; Ig SF	-1.5	1.46E-02									
	**Pattern (PR) and Damage-associated Recognition (DR) Receptors**											
C1QA	Complement Component; PRR (19799115); C1q SF	-2.3	4.91E-05					-1.7	1.29E-02			
C1QB	Complement Component; PRR (19799115); C1q SF	-2.6	4.13E-05		-1.6	5.22E-02						
C1QC	Complement Component; PRR (19799115); C1q SF	-2.3	5.39E-05					-1.7	1.61E-02			
C3AR1	Complement Component Receptor; GPCR SF										-2.3	5.27E-03
C4BPAL*	Complement Component Binding Protein; CCP SF										-2.4	3.78E-02
C5	Complement Component; Alpha-2-macroglobulin SF; ANATO SF; IsoPREN C2-like SF; MG1 SF; NTR-like SF										-1.9	1.14E-02
C7	Complement Membrane Attack Complex (2808363); CCP SF	-1.9	3.94E-02									
CD1D	Pattern Recognition Receptor (22734667); CD1 SF; MHC I SF; Immunoglobulin SF										-2.5	2.89E-03
CD207	PRR (27903635); DC-associated (21298011); CLECT SF	-4.2	4.37E-02									
CD209	PRR (15950451); M2 Macrophage-associated (15070901); CLECT SF	-2.8	1.42E-03					-2.1	3.13E-02			
CD302	PRR; DC-associated (28106145); CLECT SF										-2.1	6.84E-03
CD36	PRR (15690042, 19342682); SRCR SF; CD36 SF	-3.7	8.88E-09									
CD5L	PRR (16030018); SRCR SF	-10.9	8.19E-04		-4.7	6.80E-03					-4.2	2.41E-02
COLEC12	PRR (11162630); CLECT SF							-1.9	7.57E-03			
CR1	Complement Component; CCP SF										-3.0	1.86E-02
CR2	Complement Component; CCP SF										-5.2	3.00E-14
DHX58	PRR (16116171); RIG-I-Like Receptor Family	-3.3	7.59E-03					-2.7	3.59E-02			
LGALS9	PRR (12684513); GLECT SF	-2.2	2.92E-04					-1.8	1.10E-02			
SIGLEC15	SIGLEC SF	-3.4	5.06E-02									
TLR1	PRR (18056480), TLR SF	-2.2	8.67E-04									
TLR5	PRR, TLR SF	-2.0	4.03E-02									
TLR6	PRR (15661917), TLR SF	-2.0	4.77E-03								-2.1	1.41E-02
TLR7	PRR, TLR SF										-2.0	4.66E-02
TLR8	PRR, TLR SF	-2.3	1.46E-03					-2.2	2.49E-03			
TLR9	PRR, TLR SF											
TREM2	(-) Regulator of TLR4 Signaling (16951310); PRR (12847223); TREM-like Receptor SF; Ig SF				-2.5	4.89E-02						
ZBP1	Antiviral Immune Response (22344284); PRR (17618271); Z-alpha SF	-3.4	5.56E-04					-2.5	1.11E-02			
	**Oxidative Stress Response**											
CYBB	Superoxide Generation; Ferric Reductase SF	-1.6	3.34E-02									
DDIT4L	Oxidative Stress Response (12453409); RTP801 C SF										-3.0	2.84E-04
FDXR	Oxidative Stress Response (12370809); NAD binding 8 SF				-1.5	2.76E-02						
GSTA2	Glutathione S-transferase; Thioredoxin-like SF	-26.6	8.94E-06		-3.6	5.23E-02		-7.4	7.07E-03			
GSTM1	Glutathione S-transferase; Thioredoxin-like SF	-3.0	2.39E-05		-1.9	9.10E-03						
GSTM4	Glutathione S-transferase; Thioredoxin-like SF	-2.9	3.46E-06		-1.8	5.07E-03						
GSTP1	Glutathione S-transferase; Thioredoxin-like SF	-1.8	7.59E-03					-1.5	4.90E-02			
GSTT1	Glutathione S-transferase; Thioredoxin-like SF	-2.6	1.25E-03		-1.8	4.13E-02						
GSTT2	Glutathione S-transferase; Thioredoxin-like SF											
GSTZ1	Glutathione S-transferase; Thioredoxin-like SF				-1.6	1.03E-02						
IPCEF1	Oxidative Stress Response (11320098); PH-like SF										-2.3	2.46E-02
NOXO1	Superoxide Generation; PX Domain SF	-1.9	1.71E-03									
OXSR1	Oxidative Stress Response (14707132); PKC-like SF											
PRDX6	Oxidative Stress Response; Thioredoxin-like SF	-2.9	1.45E-07					-2.2	5.44E-05			
	**Surface Receptors and Ligands**											
ABCC4	Prostaglandin E2 Transporter (25433169); ABC Membrane SF											
ACVR1B	Activin Receptor SF; TGF beta GS SF	-1.5	1.73E-02									
ADGRB1	Angiogenesis; GPCR SF	-2.6	1.64E-02									
ADGRE4	GPCR SF	-2.4	9.29E-06		-1.6	2.26E-02					-2.6	1.56E-03
ADORA3	(-) Regulator of Inflammation (16849509); GPCR SF										-3.3	5.03E-03
BTNL2	(-) Regulator of Inflammation (17237401); (-) Regulator of T Cell Activation (17237401); Ig SF							-19.6	1.37E-02			
CADM1	Adhesion Molecule (18684968); CRTAM Ligand; Ig SF							-1.8	2.11E-02			
CD109	T Cell Activation Marker (11861284); Alpha-2-macroglobulin SF; IsoPREN C2-like SF; MG1 SF										-1.7	4.15E-02
CD2	Positive Regulator of TCR Signaling (11160298); CD2 SF; DUF2360 SF; Immunoglobulin SF										-1.8	9.24E-03
CD200R1L	Tolerogenic Dendritic Cell Marker (18424714); Ig SF										-6.9	7.81E-03
CD27	NK Cell Development Marker (18406373); T Cell Activation Marker; Treg Associated (17425604); TNFR SF	-2.2	4.87E-02									
CD28	(+) Regulator of T Cell Activation; Immunoglobulin SF										-3.2	1.08E-02
CD300C	(-) Regulator of Dendritic Cell/NK Cell Function (18535206, 20656921); CMRF-35 Family; Ig SF	-2.2	5.26E-03					-2.4	8.64E-04			
CD300H	(+) Regulator of Inflammation (26221034); CMRF-35 Family; Ig SF	-2.3	1.10E-05					-1.5	3.24E-02			
CD300LF	(-) Regulator of T Cell Activation (16794255); Ig SF; SIT SF	-1.8	5.14E-02									
CD302	Phagocytosis (19261355); CLECT SF	-1.9	9.28E-03					-1.6	5.19E-02			
CD33	SIGLEC SF	-1.6	4.32E-02									
CD36L1*	SRCR SF; CD36 SF	-2.3	2.24E-03									
CD3G	T Cell Associated; Ig SF; ITAM SF										-2.1	8.80E-03
CD4	T Cell Associated; Ig SF	-2.8	1.41E-04					-2.0	1.03E-02		-2.2	1.61E-02
CD40	T Cell Co-stimulatory; TNF Receptor SF										-17.7	4.10E-02
CD44	Link Domain SF	-1.6	2.09E-02									
CD48	Positive Regulator of Mast Cell Activation (12759438); SLAM Family Ligand; CD2 SF; Ig SF; SLAM SF										-1.9	1.09E-02
CD68L1*	LAMP SF	-1.7	1.16E-02									
CD7	Galectin 3 Receptor (14678989, 24792331); (+) Regulator of TCR Signaling (11485208); Ig SF	-2.2	4.61E-02									
CD72	B Cell Differentiation Marker; CLECT SF	-1.8	4.52E-02									
CD83	B Cell Activation Marker (8422464); DC Maturation Marker (12403928); Ig SF	-2.3	1.40E-02									
CD86	(+) Regulator of T Cell Activation; B7 SF (15960813); Immunoglobulin SF							-1.8	1.93E-02			
CEACAM8	Ig SF	-7.5	1.20E-02									
CLCA4	Chloride Ion Transport; CLCA N SF; vWFA SF; DUF1973 SF	-27.9	1.34E-07					-13.2	2.57E-05		-3.1	1.16E-02
CLCA4L*	CLCA N SF; vWFA SF; DUF1973 SF	-3.1	1.92E-02									
CLCN2	Voltage-gated Chloride Channel; Voltage-gated ClC SF							-1.8	3.54E-05			

CLEC17A	CLECT SF										-3.6	4.30E-03
CLEC2D	(+) Regulator of IFNG Production (15104121); KLRB1 Ligand (14990792); CLECT SF	-1.5	5.17E-02									
CLEC3B	Wound Healing (9152657); CLECT SF	-1.9	2.18E-03		-1.7	7.15E-03						
ERBB2 (CD340)	Recep L Domain SF; FU SF; PKC-like SF	-1.9	1.33E-03									
ERBB3	Recep L Domain SF; FU SF; PKC-like SF	-1.6	4.43E-02									
FCER1A	IgE; Receptor; Ig SF							-2.3	3.53E-02			
FCER1G	IgE Receptor; TCR Zeta Zeta SF; ITAM SF	-1.5	5.31E-02									
FCER2	IgE Receptor; CLECT SF										-12.0	1.34E-02
FCGR3A	Ig Receptor; IgSF	-2.2	8.60E-05					-1.7	1.21E-02			
FCGRT	Ig Receptor; IgSF	-1.5	2.30E-02									
FCRL1	B Cell Differentiation Marker (15479727); Immunoglobulin SF										-5.0	1.34E-02
FCRLA	B Cell Differentiation Marker (22078318); Immunoglobulin SF										-3.8	3.49E-02
IGJ	B Cell Marker; Ig J-Chain SF										-1.8	1.92E-02
ITGA4	T Cell Activation Marker; FG-GAP SF; Integrin Alpha2 SF										-1.7	4.03E-02
ITGAE	Treg-Associated (m, h) (15845457, 17620361); vWFA SF; FG-GAP SF; Integrin Alpha2 SF										-1.8	4.27E-02
JAML	(+) Regulator of Gamma-delta T Cell Activation (21220118); Neutrophil Chemotaxis (15800062); Ig SF	-1.7	2.72E-02					-1.7	3.23E-02			
KCNJ2	Sour Taste Receptor (26627720); IRK N SF; Ion Trans 2 SF										-2.5	2.94E-04
LAPTM5	(+) Regulator of Inflammation (22733818); Mtp SF	-1.6	3.96E-02									
LRP1B	Endocytosis (11384978); Pseudomonas Exotoxin Receptor (16095885); LDLa SF; EGF CA SF; LY SF	-2.7	1.12E-02									
LTB4R2	Leukotriene B4 Receptor; GPCRSF	-2.1	3.08E-02									
LY9	(+) Regulator of Toll-like Receptor Signaling Pathway (19395959); ML SF										-1.8	4.79E-02
MS4A2	IgE Receptor; CD20 SF							-1.9	2.34E-02			
P2RX3	Purinergic Receptor SF							-3.9	4.22E-03			
PADI2	(-) Regulator of Inflammation (18645041); PAD SF							-5.2	1.54E-06			
SCN2A	Voltage-gated Na^+ Channel; Na^+ Trans Assoc SF							-2.7	4.98E-02			
SEMA3A	Negative Regulator of T Cell Activation (16380453); Sema SF; Immunoglobulin SF							-2.2	1.38E-02			
SEMA4D	Positive Regulator of B Cell/DC Cell Function (18087252); Th1 Associated (18087252); Ig SF							-1.8	3.81E-02			
SIRPB2	Ig SF	-2.8	6.49E-03		-2.0	3.09E-02						
SLAMF6	Th1-Associated (14988414); SLAM SF; CD2 SF; Immunoglobulin SF										-2.1	4.40E-02
SSTR5	Somatostatin Receptor; GPCR SF	-5.7	4.20E-03					-4.8	1.30E-02			
TRPV3	Intracellular Ca2+ Channel (30127359); Ion Trans SF	-1.8	1.05E-02									
TRPV6	Chloride Ion Transport; Epithelial Cell Calcium Transporter (26109800); ANK SF	-6.0	4.34E-02								-7.7	1.24E-06
VIP	Hormone 2 SF	-3.5	5.68E-03									
	**Transcription Factors**											
AHRR	Transcriptional Repressor; HLH SF; PAS SF; Trp Dioxygenase SF	-2.8	3.02E-04					-2.6	6.47E-04		-2.2	2.98E-02
AR	Androgen Receptor; Nuclear Receptor (NR) DNA-binding Domain (DBD) SF										-2.7	4.53E-02
CDX2	Positive Regulator of Enterocyte/Goblet Cell/Paneth Cell Differentiation (21081128) ; Homeodomain SF	-1.6	1.15E-02									
DBP	B Zip1 SF							-2.1	1.42E-02			
DLX3	Homeodomain SF	-3.8	2.11E-02									
DLX4	Homeodomain SF	-4.1	2.69E-03									
EOMES	TC1-associated (14605368); TBOX SF	-4.8	1.12E-04		-2.2	3.65E-02						
EVX1	Homeodomain SF	-38.1	2.47E-02									
FOXF2	FH SF							-1.9	5.72E-03			
FOXH1	FH SF	-2.1	3.28E-02									
FOXJ1	Negative Regulator of NFKB Signaling (16585573); TH2-Associated (14963332); FH SF	-4.8	4.62E-03									
FOXO6	Gluconeogenesis (23324123); FH SF	-13.7	8.20E-06					-9.1	1.78E-04			
FOXS1	FH SF	-3.8	3.26E-05					-2.5	5.82E-03			
HOXA11	Homeodomain SF	-1.6	5.12E-02									
HOXA5	Homeodomain SF	-1.6	1.74E-02									
HOXC9	Homeodomain SF										-12.3	5.27E-03
HOXD10	Homeodomain SF	-2.3	8.74E-03					-2.6	1.40E-03		-2.6	1.85E-02
HOXD11	Homeodomain SF							-1.9	5.02E-02			
IRF4L	IRF3 SF	-3.4	5.23E-03					-2.7	3.39E-02			
IRF8	Type 1 IFN-Induced; Type 1 IFN Signaling (15864272); IRF/IRF4 SF (11997525)	-1.6	1.94E-02									
IRF9	Antiviral Immune Response (17559358); Type 1 and 2 IFN Signaling (15864272); IRF/IRF4 SF (11997525)	-1.8	5.24E-03					-1.5	4.68E-02			
ISX	Transcriptional Repressor (23393141); Homeodomain SF	-3.3	2.28E-03									
KLF11	zf-H2C2 2 SF	-4.2	3.47E-05		-2.0	4.37E-02						
KLF12	zf-C2H2 SF; zf-H2C2 2 SF										-2.9	1.06E-03
KLHL7	Ubiquitin Ligase (21828050); BTB SF; BACK-like SF; Kelch 1 SF	-2.1	2.73E-04					-1.9	2.88E-03			
MAF	M2 Macrophages (17082649); bZIP1 SF	-2.2	1.08E-04					-1.9	1.80E-03		-2.1	1.40E-02
MAFB	Macrophage Differentiation Marker (15598817); bZIP1 SF	-1.5	3.08E-02								-1.9	5.99E-03
MEIS2	Homeodomain SF							-1.8	5.29E-02			
NFATC2	TH2-Associated (8943202); UBQ SF										-1.9	4.03E-02
NR1D1	NR DBD-like SF; NR LBD SF							-1.8	3.13E-02			
NR1H4	Bile Acid Receptor (10334992); NR DBD-like SF; NR LBD SF	-17.1	2.31E-11		-2.3	2.51E-02		-7.6	1.96E-06		-3.7	1.44E-02
NR3C2	Mineralocorticoid Receptor; NR DBD-like SF; NR LBD SF	-1.6	2.11E-02									
OLIG2	Neurogenesis (21382543.); HLH SF										-143.0	1.09E-03
PAX5	B Cell Differentiation (15469848); PRD Class Homeobox Transcription Factor; Homeodomain SF										-2.8	2.30E-02
PCGF2	Th2-Associated (11520462); Polycomb-group (PcG) SF	-1.5	4.54E-02					-1.5	5.29E-02			
PKNOX2	Homeodomain SF	-2.0	2.25E-02					-1.8	5.16E-02			
POU6F1	Pou SF; Homeodomain SF							-1.8	3.37E-02			
PPARA	Th2 Associated (18645614)); NR DBD-like SF; NR LBD SF	-1.5	4.14E-02									
PPARD	M2 Macrophages (18522830); NR DBD-like SF; NR LBD SF	-1.6	4.85E-03									
SATB1	CD8 T Cell Development (15814699); SATB1 N SF; CUT SF; Homeodomain SF							-1.8	2.46E-02		-1.9	1.64E-02
SP110	Sp100 SF; SAND SF; PHD SF; Bromodomain SF	-1.5	5.40E-02									
TCF21	HLH SF	-1.8	4.67E-03					-1.6	1.33E-02			
TCF7L1	HMG-box SF; CTNNB1-binding SF	-1.9	2.24E-02									
TEF	B Zip1 SF	-1.9	3.52E-03					-1.8	3.94E-03			
THRA	Thyroid Hormone Receptor; Nuclear Receptor (NR) DNA-binding Domain (DBD) SF; NR LBD SF	-1.6	2.58E-02									
VDR	Vitamin D Receptor; NR DBD-like SF; NR LBD SF	-1.7	7.90E-03					-1.5	3.04E-02			
	**Mitochondrial Proteins**											
COQ8A	DNA Damage Response; APH ChoK-like SF	-2.2	9.79E-06		-1.5	1.73E-02						
HMGCS2	Ketogenesis (25347470); HMG CoA Synt C SF	-21.2	0.00E+00					-12.8	0.00E+00		-7.2	0.00E+00
MT-ATP6	ATP Synthesis; ATP Synt A SF	-1.7	1.36E-02									
MT-CO1	Respiratory Electron Transport Chain; Heme Cu Oxidase I SF	-1.7	7.42E-03									
MT-CO2	Respiratory Electron Transport Chain; Heme Cu Oxidase I SF	-1.5	5.08E-02									
MT-CO3	Respiratory Electron Transport Chain; Heme Cu Oxidase I SF	-1.9	4.64E-03									
MUL1	(+) Regulator of NFk-B Activation (18213395); RING Ubox SF	-1.5	1.04E-02									
SLC25A10	Malate Transporter (16027120); Mitochondrial Carrier Protein SF Solute Carrier SF	-1.8	2.22E-04									
SLC25A27	Mitochondrial Uncoupling Protein; Mitochondrial Proton Transport; Solute Carrier SF	-2.1	1.55E-05									
SLC25A34	Mitochondrial Phosphate Transporter; Mitochondrial Carrier Protein SF Solute Carrier SF	-7.8	1.41E-09									
SLC25A6	ATP/ADP Translocase; Mitochondrial Carrier Protein SF; Solute Carrier SF	-1.5	2.26E-02									
TRAP1	Mitochondrial Heat Shock Protein (21878357); HATPase c SF	-1.5	1.76E-02									
TST	RHOD SF	-2.2	3.80E-05					-1.5	5.16E-02			
UCP2	Mitochondrial Uncoupling Protein; Mitochondrial Proton Transport; Solute Carrier SF	-1.8	7.55E-03									
VARS2	Class I Aminoacyl tRNA Synthetase; Anticodon Ia-like SF; NT Trans SF	-1.5	1.93E-02									

	**Miscellaneous**											
ACE	Angiotensin I converting enzyme; Metalloprotease; GluZincin SF	-2.6	7.25E-05					-2.0	6.94E-03			
ACE2	Angiotensin I converting enzyme				-2.6	4.67E-02						
AGT	Angiotensinogen; SERPIN SF							-4.3	4.12E-03		-5.6	1.62E-05
AGTR1	Angiotensin II receptor; (+) Regulator of Inflammation (19225144); GPCR SF										-3.2	6.35E-04
AGTR2	Angiotensin II receptor; GPCR SF										-11.5	3.94E-03
ALPI	(-) Regulator of Intestinal Inflammation (20536777); alkPPc SF	-159.7	2.09E-10		-4.9	5.20E-03		-32.9	4.94E-06			
ALPIL1*	alkPPc SF	-10.3	1.67E-04		-3.1	3.58E-02						
ARRDC3	(-) Regulator of G protein-mediated Signaling (24292823); Arrestin N SF; Arrestin C SF				-1.5	4.39E-02						
CARD14	(+) Regulator of NFKB Activation (11278692); DD SF; PDZ SF; SH3 SF; NK SF	-2.2	2.07E-03									
CHP2	(+) Regulator of Calcineurin Signaling (18815128); (+) Regulator of NFAT Signaling (18815128); EFh SF	-2.9	5.14E-05								-2.9	1.12E-05
COBL	(+) Regulator of Intestinal Microvilli Formation (25031432); Cobl SF; Atrophin-1 SF							-1.5	1.01E-02			
COQ8A	DNA Damage Response; APH ChoK-like SF	-2.2	9.79E-06									
CTBS	Asn-linked Glycoprotein Catabolism; GH18 Chitinase Like SF (17594485)										-2.5	3.96E-02
DNAH14	DHC N2 SF; AAA SF; P-loop_NTPase SF; MT SF; Dynein Heavy SF; DHC N2 SF										-60.4	1.56E-02
DOK2	(-) Regulator of TLR4 Signalling (15699069); PH-like SF							-1.6	3.97E-02			
GUCA2A	(+) Regulator of Chloride Ion Transport (14988827); Guanylin SF	-6.3	2.26E-05					-5.1	1.44E-04			
HIC1	(-) Regulator of SIRT1 Signaling (23770291); Transcriptional Repressor (16269335); BTB SF; zf-C2H2 SF							-1.8	1.41E-02			
KCNE3	Voltage-gated Potassium Channel; ISK Channel SF				-1.7	1.16E-03						
LCN15	Lipocalin SF	-7.6	4.90E-02		-32.1	3.66E-07						
LCP2	(+) Regulator of T Cell Activation (17420479); SH2 SF; Sterile Alpha Motif (SAM) SF	-1.7	7.46E-03					-1.6	1.11E-02			
MAP3K3	Positive Regulator of TLR Signaling (14661019, 18206350); PB1 SF; PKc-like SF							-1.5	1.38E-02			
MERTK	(-) Regulator of Dendritic Cell Function (19301199); GAS6 Receptor; Ig SF; FN3 SF; PKC-like SF	-1.7	7.59E-03					-1.5	4.10E-02			
METTL7A	Methyltransferase; AdoMet MTases SF				-1.7	3.83E-03						
MOV10	Antiviral Response (26842467); AAA 11 SF; UvrD C 2 SF	-1.7	1.22E-03									
NT5E	Adenosine Metabolism; MPP SF; 5 Nucleotid C SF	-2.1	1.30E-02					-2.3	4.92E-03		-2.5	1.78E-04
NXPE2	Neurexophilin SF	-20.1	2.40E-06					-13.8	2.92E-05		-3.4	2.01E-03
PADI2	Negative Regulator of Inflammation (18645041); PAD N SF; PAD M SF; PAD SF	-8.6	1.42E-09								-2.5	1.60E-04
PIK3AP1	(+) Regulator of B Cell Development (12833156); DBB SF										-2.0	6.93E-03
PIK3IP1	(-) Regulator of T Cell Activation (22706993); KR SF	-1.9	1.62E-03					-1.5	4.80E-02			
PLA2G2A	Antibacterial Response (26644377); Colorectal Cancer; (18508504); Phospholipid Metabolism; PLA2-like SF										-1633.2	1.33E-05
PLA2G4B	Antibacterial Response (11741884); C2 SF; Patatin and cPLA2 SF	-1.6	3.68E-02									
PLCD1	(-) Regulator of TLR4 Signaling (26643908)	-2.4	1.77E-06		-1.6	1.46E-02		-1.5	3.39E-02			
PTPRD	(+) Regulator of STAT3 Signaling (17360477); Immunoglbulin SF; FN3 SF; PTPc SF	-2.6	1.41E-04					-2.5	2.63E-04			
RGS10	(-) Regulator of Inflammation (18716210); (-) Regulator of NFK-B Activation (21849548); RGS SF	-1.9	2.55E-02									
SCIN	(-) Regulator of Mucin Secretion (15342343); ADF Gelsolin SF	-1.8	3.67E-02									
SPIDR	DNA Damage Response (23754376); DUF4502 SF; DUF4503 SF	-1.9	2.85E-04									
SULT1E1	Phase II Conjugating Enzyme; Sulfotransfer 3 SF	-8.2	1.08E-09					-5.2	7.10E-07			
TICAM1	(+) Regulator of Toll-like Receptor Signaling (15219996); Ripoptosome Associated; TIR 2 SF; RHIM SF	-1.7	1.41E-03									
TNFAIP2	(+) Regulator of Inflammation (16456005); TNF Induced; Sec6 SF	-1.8	2.30E-04					-1.7	1.04E-03			
TYROBP	(-) Regulator of TLR Signaling (15895090); DAP10 SF	-1.6	3.74E-02									
UNC93B1	(+) Regulator of Endosomal TLR Signaling (18082565); Major Facilitator SF	-1.5	4.49E-02					-1.5	4.34E-02			
UVRAG	DNA Damage Response (27439570); Positive Regulator of Autophagy (26565689); C2 SF	-1.5	1.97E-02									
VAV3	Phagocytosis (16546099); TCR Signaling (15886116); CH SF; RhoGEF SF; PH-like SF; C1 SF; SH3 SF; SH2 SF	-1.5	5.02E-02									
VIL1	Bacterial Resistance (12181160); ADF Gelsolin SF; VHP SF	-1.5	2.16E-02									
ZFP42	Induced Pluripotent Stem Cell Marker (25830878); zf-H2C2 2 SF										-89.1	3.67E-11
	**NK Cell Associated**											
CD8A	NK Cell Associated; T Cell Associated; Ig SF	-2.2	4.54E-03		-1.7	5.31E-02						
GZMK	NK Cell Associated; Granzyme; Tryp SPc SF	-4.0	1.31E-02									
KLRC1	NK Cell Associated; CLECT SF	-2.5	2.35E-02									
KLRD1L*	CLECT SF				-2.1	2.36E-02						
KLRG1	NK Cell Associated; CLECT SF	3.7	4.54E-02					-2.0	4.23E-02			
KLRK1	(+) Regulator of NK Cell Activity (18979516); CLECT SF										-3.4	6.23E-09
NCAM1	Adhesion Molecule; NK Cell Associated; Ig SF	-1.9	3.06E-02									
NCR1	Pattern Recognition Receptor (11234016); IgSF	-7.2	1.19E-06		-2.9	2.48E-04						
NCR2	Pattern Recognition Receptor (11536166); Ig SF	-12.1	1.81E-05		-2.5	4.13E-02		-4.8	1.35E-02			
	**Inflammasome Associated**											
CASP1	Positive Regulator of IL1B/IL18 Bioactivity (10080100); DD SF	-2.1	9.13E-04									
PYCARD	Inflammasome Associated; DD SF	-1.9	8.72E-03					-1.8	1.54E-02			
TXNIP	NLRP3-associated (20075245); Arrestin C SF	-2.6	5.39E-05					-2.0	3.65E-03			
	**Neurotransmitter and Neuropeptide Related**											
ADRB1	Epinephrine Receptor; Neurotransmitter Receptor; GPCR SF							-2.0	7.57E-03			
CHRM2	Cholinergic Receptor; GPCR SF										-3.0	7.15E-03
GABARAPL1	UBQ SF	-1.8	6.54E-04					-1.5	2.90E-02			
GABBR1	GABA Receptor; GPCR SF	-1.8	2.17E-03									
GABRB2	GABA Receptor; GPCR SF	-5.4	4.12E-06					-4.0	1.45E-04			
GAL	Neuropeptide; Galanin SF; GMAP SF	-3.0	4.97E-02		-3.1	1.57E-02						
GFRA1	Receptor; GDNF SF	-2.0	4.61E-02									
GRIK1	Neurotransmitter Receptor; Non-NMDA Ionotropic Receptor; Periplasmic-binding Protein Type 1 SF	-8.6	4.27E-02									
GRIK4	Neurotransmitter Receptor; Non-NMDA Ionotropic Receptor; Periplasmic-binding Protein Type 1 SF	-2.7	1.76E-02					-2.7	9.60E-03			
GRINA	Neurotransmitter Receptor; NMDA Ionotropic Receptor; BI-1-like SF	-1.8	1.50E-03									
MAOA	Dopamine/Norepinephrine/Serotonin Catabolism (10389141); SDR SF	-2.7	1.11E-07					-1.9	7.84E-04			
NECAB2	(+) Regulator of Adenosine Receptor Signaling (17689978); EFh SF; ABM SF	-3.1	1.88E-02									
OPRL1	Nociceptin Receptor; GPCR SF	-4.9	3.95E-04									
P2RX3	Purinergic Receptor; P2X Receptor SF	-6.5	3.20E-05					-3.9	4.22E-03			
P2RY12	Purinergic Receptor; GPCR SF	-2.1	5.25E-02									
P2RY13	Purinergic Receptor; GPCR SF	-2.6	6.61E-04					-2.0	1.81E-02			
SCNN1B	Non voltage-gated Sodium Channel; Taste Receptor (23886383); ASC SF	-3.8	3.19E-02		-3.3	2.26E-02						
	**Type 1 or Type 2 IFN-Induced Genes**											
CXCL10	Type 2 IFN Induced; CXCR3 Ligand; CXC Chemokine SF	-3.4	3.22E-02					-4.4	7.18E-03			
CXCL11	Type 2 IFN Induced; CXCR3 Ligand; CXC Chemokine SF	-4.8	3.33E-02									
CXCL9	Type 2 IFN Induced; CXCR3 Ligand; CXC Chemokine SF	-7.5	6.02E-04					-5.7	3.65E-03		-6.1	7.08E-08
DAPK1	Type 2 IFN Induced (18250155); ANK SF; Ras-like GTPase SF; DD SF	-1.9	1.20E-02								-2.5	8.50E-03
ERAP2	Type 2 IFN Induced (12799365); Antigen Processing and Presentation (12799365); GluZincin SF	-1.6	4.60E-02									
GBP1	Type 2 IFN Induced (17266443); Ras-like GTPase SF	-1.9	5.21E-02					-2.0	4.04E-02			
GBP2	Type 2 IFN Induced (17266443); Ras-like GTPase SF	-2.4	1.02E-02					-2.4	9.65E-03			
GBP4	Type 2 IFN Induced (17266443); Ras-like GTPase SF	-2.4	4.83E-02									
GBP6	Type 2 IFN Induced (17266443); Ras-like GTPase SF	-2.6	5.25E-03					-2.1	3.20E-02			
FBP2	Type 2 IFN Induced (9282826); Gluconeogenesis (18780768); FIG Superfamily	-1.6	1.05E-02									
GPNMB	Type 2 IFN Induced (17475886); (-) Regulator of T Cell Activation (17284525); E set SF										-2.5	3.04E-05
HPSE	Type I IFN Induced Response (21478870); Antiviral Response (21478870); Heparanase; Glyco hydro 79n SF	-1.6	1.75E-02					-1.5	5.25E-02		-2.1	4.45E-03
IDO1	Type 2 IFN Induced (2109605); Tolerogenic Dendritic Cells (15459668) Marker; IDO SF										-3.8	6.52E-05

IFI27	Type 1 IFN Induced (20939681); Antiviral Response (27194766); Ifi-6-16 SF	-5.8	7.91E-04					-3.9	1.12E-02			
IFI44	Type 1 IFN Induced (7925411); Antiviral Immune Response (22896602); TLD SF	-2.9	1.80E-02					-2.7	2.55E-02			
IFI44L	Type 1 IFN Induced (21478870); Antigen Processing and Presentation (11021531); Ras-like GTPase SF	-2.3	3.75E-03					-2.0	1.59E-02			
IFITM1	Type 1 IFN Induced (14980080); Antiviral Immune Response (22344284); CD225 SF	-2.6	1.45E-02									
IFITM1L2*	CD225 SF	-2.3	6.10E-05					-1.7	8.91E-03			
IRF8	Type 1 IFN-Induced; Type 1 IFN Signaling (15864272); IRF SF; IRF4 Subfamily (11997525)	-1.6	1.94E-02									
MAP2K6	Type 1 IFN Induced (15644321); Antiviral Response (15229216); PKC-like SF	-1.8	3.77E-02					-1.8	4.90E-02			
MOV10	Type 1 IFN Induced (21478870); Antiviral Response (26842467); AAA 11 SF; UvrD C 2 SF	-1.7	1.22E-03					-1.5	1.20E-02			
MPEG1	Type 1 and 2 IFN Induced (23257510); Antibacterial Responses (23257510); MACPF SF	-2.2	4.93E-03					-2.1	5.72E-03		-2.1	7.99E-03
OASL	Type 1 IFN-Induced (10087211); Antiviral Response (22531715); NT Pol-beta-like SF; OAS1 C SF; UBQ SF	-14.4	5.68E-03					-12.1	1.20E-02			
PARP9	Type 2 IFN Induced (16809771); Antiviral Response (26479788); Macro SF; ADP Ribosyl SF							-1.9	1.04E-03			
RNF213	Type I IFN Induced (10547268); RING Ubox SF	-1.8	4.61E-02					-1.8	3.66E-02			
RSAD2	Type 1 IFN-induced (9391139); Antiviral Immune Response (20308629, 22377585); Viperin SF	-4.4	2.06E-02					-4.3	2.28E-02			
THAP12	Type 1 IFN Induced (21910972); Antiviral Response (21910972) ; THAP SF; Dimer Tnp hAT SF	-10.5	4.27E-02					-13.4	2.27E-02			

Supplemental Table S9: Clustered Metabolism Genes (DEGs) Up-regulated

Gene	Functional Categorization	52 DPI w worms vs Control Fold	EDGE FDR Adj p value		52 DPI w/o worms vs Control Fold	EDGE FDR Adj p value		(52 DPI Inf w/o worms vs w Worms Fold	EDGE FDR Adj p value		(21 DPI Inf vs Control) Fold	EDGE FDR Adj p value
	**Amino Acid Metabolism or Transport**											
ARG1	Arginine Metabolism (3540966); M2-associated (5879104); Arginase HDAC SF	122.6	4.56E-05					16.6	8.41E-04			
ARG2	Arginine Metabolism (16128822); Arginase HDAC SF	1.7	3.31E-02					1.6	2.16E-02			
BCAT1	BCAA Catabolism (6933702)); Glycolysis (28699638); PRK13357 SF	2.4	4.81E-04					1.9	5.18E-03			
CBS	Cysteine Metabolism (19010420); Serine Metabolism; Trp-synth-beta II SF; CBS Pair SF	3.4	1.61E-03					3.5	7.42E-05		5.5	7.18E-05
GNMT	Homocysteine Metabolism (21411609); AdoMet MTases SF										4.3	2.10E-02
IL4I1L	Putative L-amino acid oxidase; SDR SF	161.2	3.98E-05					26.0	1.39E-04		437.7	1.59E-14
ODC1	M2-associated (19726720); Ornithine Metabolism (2317811); Polyamine Metabolism; PLPDE III SF				-1.5	1.27E-02		1.6	4.12E-03			
PHGDH	Serine Biosynthesis (27110680); SerA SF	5.1	3.92E-05					3.3	5.01E-04		5.0	9.01E-04
PSAT1	Serine Biosynthesis (12633500); AAT I SF	7.2	3.12E-04					3.9	3.89E-03		20.6	4.69E-11
SDSL	Serine Metabolism (16580895); Threonine Metabolism (16580895); Trp Synth beta II SF	39.6	1.38E-02									
SHMT2	Glycine Biosynthesis (25619277); Folate Biosynthesis; Serine Biosynthesis	2.0	2.65E-05					1.5	1.06E-02			
SLC12A8	Amino Acid Transport (19472210); Polyamine Transport (19472210); Solute Carrier SF	3.5	7.40E-04					3.1	3.65E-04			
SLC16A10	Phenylalanine/Tryptophan/Tyrosine Transport (23045339); Solute Carrier SF	1.8	3.61E-02									
SLC1A2	Glutamate Transport (7521911); SDF SF; Solute Carrier SF	3.0	2.04E-03					2.1	2.44E-02			
SLC1A4	Alanine/Cysteine/Proline Transport ((8101838, 11824937; 14502423); Solute Carrier SF	2.5	1.36E-03									
SLC1A5	Glutamate/Glutamine Transport (21757002); Solute Carrier SF	1.8	8.53E-04					1.6	5.72E-03			
SLC38A2	Alanine/Asparagine/Cysteine/Glutamine Transport (12845534); Solute Carrier SF	1.8	2.90E-02									
SLC3A2	BCAA Transport (9751058); Tryptophan Transport; Tyrosine Transport; Solute Carrier SF	1.8	1.83E-03									
SLC6A9	Glycine Transport (8183239); Solute Carrier SF	1.8	1.29E-02									
SLC7A1	Arginine Transport (21302286); Lysine Transport (21302286); Solute Carrier SF	1.6	1.25E-02					1.5	3.05E-02			
SLC7A11	Cystine Transport (10206947); Glutamate Transport (10206947); Solute Carrier SF							3.7	3.60E-02		4.3	6.75E-07
SLC7A2	Arginine Transport (8954799), M2-associated (16670299); Solute Carrier SF	1.9	4.15E-02									
SLC7A3L11*	Putative Amino Acid Transporter; Solute Carrier SF										188.2	1.59E-03
SLC7A5	BCAA Transport (9751058); Solute Carrier SF	2.7	7.21E-06					1.9	1.40E-03		3.3	1.30E-03
SLC7A6	Arginine Transport (19562367); Solute Carrier SF	1.7	2.15E-03									
SLC9C1	Sodium-hydrogen Exchanger (14634667); Solute Carrier SF										4.1	7.81E-04
TAT	Bioconversion of L-Tyrosine into p-Hydroxyphenylpyruvate; AAT I SF	4.2	4.00E-02								6.9	3.49E-02
	**Carbohydrate Metabolism or Transport**											
AACS	Biosynthesis of Acetyl-Coenzyme A (20102333); AFD Classc I SF	2.0	4.99E-05					1.8	1.18E-04			
ACAT2	Biosynthesis of Acetyl-Coenzyme A; Thiolase N SF	3.5	1.27E-10					2.5	4.51E-08			
ACLY	Glycolysis; Lipid Biosynthesis (23932781); TCA Cycle; CCL SF	2.2	5.17E-06					1.9	1.76E-05			
ALDOC	Fructose Metabolism; Glycolysis; Aldolase Class I SF	1.6	5.08E-02					1.7	1.06E-02			
BCAT1	Glycolysis (28699638); PRK13357 SF	2.4	4.81E-04					1.9	5.18E-03			
DERL3	Negative Regulator of SLC2A1 Expression (24699711); DER1 SF	2.2	2.74E-04					1.7	7.70E-03			
GFPT1	Biosynthesis of Glucosamine 6-phosphate (1460020); Gn AT II SF; SIS SF	2.2	1.21E-05					1.7	3.70E-03			
GYS2	Glycogen Synthase SF							6.0	4.66E-02		11.9	5.62E-03
PCK2	Gluconeogenesis; Biosynthesis of Phosphoenolpyruvate (4291786); TCA Cycle; PEPCK HprK SF	2.0	1.77E-03					2.0	3.22E-04			
HK2	Glycolysis; Biosynthesis of Glucose-6-phosphate (19558793); NBD Sugar Kinase SF; Hexokinase 2 SF	8.6	2.40E-06					4.2	1.78E-04		3.3	1.33E-05
PFKFB2	Biosynthesis of Fructose-2,6-bisphosphate; Glycolysis; P-loop NTPase SF; HP SF	1.9	3.58E-02									
PFKFB3	Biosynthesis of Fructose-2,6-bisphosphate; Glycolysis; P-loop NTPase SF; HP SF	3.2	4.64E-06					2.2	5.86E-04		2.5	7.15E-03
PGK1	Biosynthesis of 3-phosphoglycerate; Glycolysis; Phosphoglycerate Kinase SF	1.8	1.24E-03					1.5	1.73E-02			
PGM2	Biosynthesis of Ribose 5-phosphate; Glycolysis (17804405); Phosphohexomutase SF	1.6	7.93E-03									
PGM3	Biosythesis of UDP-N-acetylglucosamine (11004509); Phosphohexomutase SF	2.1	7.37E-05					1.8	9.17E-04			
PKM	Biosynthesis of Pyruvate; Glycolysis (12843653); Pyruvate Kinase SF	1.9	3.11E-04					1.7	1.40E-03		2.2	2.62E-02
SDS	Biosynthesis of Pyruvate; Glycolysis; Serine/Threonine Catabolism; Trp Synth Beta II SF	5.6	1.53E-02								5.7	8.06E-05
SLC25A35	Isopropylmalate Transport (18682385); Solute Carrier SF							2.0	9.64E-03			
SLC2A3	Glucose Transport (20209635); Solute Carrier SF	3.4	1.42E-12		2.7	1.71E-10						
SLC37A3	Glucose-6-phosphate Transport (21949678); Solute Carrier SF	1.5	7.96E-03									
SLC50A1	Glucose Transport (23506865); Solute Carrier SF							1.8	9.18E-04			
SLC5A1	Glucose/Fructose/Mannose Transport (22212718); Solute Carrier SF	2.2	2.73E-04					1.7	6.38E-03			
TKTL1	Glycolysis (26273330)				6.4	1.49E-02						
	**Lipid Metabolism or Transport**											
ABCA12	Cholesterol Transport (23931754); ABC ATPase SF	4.1	1.91E-03								2.3	4.40E-02
ABCA5	Macrophage Cholesterol Metabolism (20382126); ABC ATPase SF	2.0	4.93E-03									
ACACA	Fatty Acid Biosynthesis (20457939); Acetyl-CoA Carboxylase SF; Biotinyl-lipoyl Domains SF	1.6	4.83E-03									
ACSL3	Fatty Acyl-CoA Ester Biosynthesis; AFD Class I SF; Acyl-CoA Synthetase SF	2.0	8.07E-05					1.7	2.03E-03			
ACSL4	Fatty Acyl-CoA Ester Biosynthesis; AFD Class I SF; Acyl-CoA Synthetase SF	1.5	3.29E-02									
AOAH	Lipid Metabolism (1883828); SGNH Hydrolase SF; SapB 2 SF	1.6	5.28E-02		1.7	2.02E-02						
APOBR	Lipoprotein Receptor; Neuromodulin N SF; Na Ca Ex SF				1.7	3.48E-02						
CYP51A1	Cholesterol Metabolism (12464255); Cytochrome P450	2.1	1.08E-04					1.6	5.76E-03			
CYP7A1	Cholesterol Metabolism (19965590); Cytochrome P450	12.7	4.64E-02									
DGAT2	Triglyceride Synthesis (11481335); NAT SF Superfamily							1.8	3.05E-02			
DHCR7	Cholesterol Biosynthesis (9465114); PEMT SF	1.9	3.19E-04					1.6	1.99E-03			
EBP	Cholesterol Biosynthesis (17498944); EBP SF	2.6	1.17E-05					2.3	4.62E-06			
ELOVL6	Fatty Acid Metabolism (19259639); ELO SF							2.4	1.46E-05			
FABP3	Long-chain Fatty Acid Transport (3421901); Lipocalin SF							2.3	1.30E-03		3.3	3.51E-08
FADS1	Fatty Acid Desaturase; Cyt-b5 SF; Membrane-FADS-like SF	2.4	6.98E-08					1.8	8.80E-05			
FADS2	Fatty Acid Desaturase; Cyt-b5 SF; Membrane-FADS-like SF	2.8	2.38E-06					2.3	1.63E-05			
FASN	Fatty Acid Biosynthesis (7835891); Acyl Transf 1 SF; NADB Rossmann SF	2.1	1.09E-04					1.7	3.60E-03			
FDFT1	Cholesterol Biosynthesis (19054015); Isoprenoid Biosynthesis C1 SF	1.9	3.52E-04					1.9	1.14E-05			
FDPS	Cholesterol Biosynthesis (8188698); Isoprenoid Biosyn C1 SF	2.2	8.28E-05					1.8	2.52E-03		2.0	3.53E-02
FFAR2	Acetate/Butyrate/Propionate Receptor (12711604, 27966553, 19574715); GPCR SF	3.0	5.18E-04		3.6	1.18E-05						
FFAR4	Fatty Acid Receptor (20813258); GPCR SF	2.3	1.81E-02					2.3	5.35E-03		2.3	2.68E-02
GPAT3	Triglyceride Biosynthesis (20181984); LPLAT SF	2.8	1.40E-04					2.5	7.03E-05			
HDLBP	Cholesterol Metabolism; KH-I SF										2.1	1.81E-02
HMGCR	Cholesterol Biosynthesis; MMPL SF; HMG-CoA Reductase SF	1.9	1.25E-03								1.9	1.78E-04
HMGCS1	Cholesterol Metabolism (7913309); HMG CoA Synt C SF	3.9	7.11E-09								2.8	3.97E-07
HSD17B7	Cholesterol Biosynthesis; NADB Rossmann SF; SDR SF	2.7	9.57E-06								1.9	1.22E-03
IDI1	Cholesterol Metabolism; Nudix Hydrolase SF	3.1	3.39E-06								2.2	2.17E-04
IFRD1	Lipid Metabolism (16085642); IFRD SF	1.8	2.25E-03									
INSIG1	Cholesterol Metabolism (12242342); INSIG SF	3.1	6.97E-07					2.9	1.64E-08			
LDLR	Cholesterol Metabolism; LDLa SF; EGF CA SF; LDL Receptor B SF	2.8	1.77E-06					2.1	2.26E-04			
LIPG	Lipid Metabolism; Lipase SF; PLAT SF	4.0	1.77E-03					2.3	4.46E-02			
LIPH	Lipid Metabolism; Lipase SF	2.2	2.78E-04					1.7	8.88E-03		2.4	1.88E-04
LPCAT2	Biosynthesis of Phosphatidylcholine (21498505); LPLAT SF; EFh SF				1.9	2.33E-03						
LRP2	Lipoprotein Metabolism (7642623); LDLa SF; EGF CA SF	4.3	1.38E-02					3.6	1.26E-02			

LSS	Cholesterol Biosynthesis; SQHop Cyclase C SF	2.2	1.54E-05					1.8	3.41E-04		1.9	3.03E-02
ME1	Fatty Acid Biosynthesis; Glycolysis; Malic SF; NAD Bind Amino Acid DH SF							2.6	5.20E-03		3.1	9.19E-03
MSMO1	Cholesterol Biosynthesis; FA Hydroxylase SF	2.5	5.94E-06								2.2	4.56E-06
MVD	Cholesterol Metabolism (18823933); MVD1 SF	1.9	2.48E-04					1.8	5.01E-04			
NPC1L1	Cholesterol Transport (15173162); SecD SecF SF; Patched SF							3.9	4.56E-02			
NSDHL	Cholesterol Biosynthesis (14506130); NADB Rossmann SF; SDR SF	1.7	4.11E-03					1.6	4.96E-03			
OSBPL8	Cholesterol Metabolism (17991739); H-like SF; Oxysterol BP SF	1.8	5.05E-02									
PCSK9	Cholesterol Metabolism (18753623); Inhibitor I9 SF; Peptidases S8 S53 SF	11.5	5.33E-09					9.0	1.11E-11			
PLA2G3	Lipid Metabolism (10713052); PLA2-like SF	3.6	7.51E-05					2.7	4.13E-04		2.9	4.90E-03
SC5D	Cholesterol Biosynthesis; FA Hydroxylase SF	1.8	3.28E-03					1.7	1.42E-03			
SCD	Lipid Metabolism; Vitamin A Metabolism (21573029); Membrane-FADS-like SF	3.9	9.81E-07					2.5	1.62E-04			
SOAT1	Cholesterol Ester Biosynthesis (16647063); MBOAT SF	1.7	1.57E-02									
SQLE	Cholesterol Biosynthesis (10666321); NADB Rossmann SF; SE SF	2.9	7.52E-07					2.3	8.68E-06			
STARD10	Phospholipid Transport (15911624); SRPBCC SF	1.5	3.63E-02					1.6	4.04E-03		1.9	1.91E-02
STARD4	Cholesterol Transport (23033213); SRPBCC SF	2.8	1.65E-06					1.9	8.33E-04			
STARD5	Cholesterol Transport (15897605); SRPBCC SF	1.5	4.88E-02					1.7	4.28E-03			
VLDLR	Lipid Transport; LDLa SF; EGF CA SF; Ldl Recep B SF	1.6	1.87E-03					1.5	7.00E-03			
	**Mineral Metabolism or Transport**											
ATOX1	Cu Metabolism (18685091); HMA SF	2.0	3.87E-03					1.8	8.67E-03			
FLVCR2	Ca Transport; MFS SF	1.6	2.26E-02					1.8	2.73E-03			
HEPHL1	Fe Metabolism (20685892); Cupredoxin SF	4.1	5.31E-02									
HP	Fe Metabolism; CCP SF							2.3	1.38E-02			
LTF	Fe Metabolism; Transferrin SF	52.9	5.95E-03									
MAGT1	Magnesium Transporter (21796205); Thioredoxin-like SF; OST3 OST6 SF	1.5	2.43E-02									
SELENOF	Selenoprotein; Sep15 SelM SF	1.6	1.38E-02					1.5	1.19E-02			
SELENOI	Selenoprotein (12775843); CDP-OH P Transf SF	1.5	5.04E-03									
SELENOK	Selenoprotein; DUF2763 SF	1.7	1.33E-03									
SELENOM	Selenoprotein; Sep15 SelM SF	2.1	1.21E-05					1.6	1.73E-02			
SELENOS	(-) Regulator of Inflammation (16227999); Selenoprotein S SF	1.9	2.48E-03					1.5	3.07E-02			
SLC11A1	Antibacterial Response (25350459); Fe/Mn Transport ; MntH SF; Solute Carrier SF							2.9	2.78E-02			
SLC30A5	Zn Transport (19759014); Solute Carrier SF	1.8	1.03E-03					1.5	3.16E-02			
SLC30A7	Zn Transport (12446736); Solute Carrier SF	1.7	2.93E-03					1.5	3.01E-02			
SLC39A10	Zn Transport; Solute Carrier SF	1.6	5.43E-02									
SLC39A11	Zn Transporter; Zip SF; Solute Carrier SF	1.5	5.49E-02					1.5	3.01E-02			
SLC39A7	Zn Transport (15705588)	1.7	1.29E-03					1.5	1.20E-02		1.9	9.92E-03
SLC41A2	Magnesium Transport; Solute Carrier SF	2.2	5.17E-02									
SLC5A8	Iodide Trasnporter; Solute Carrier SF	11.9	4.56E-05		4.8	3.38E-02					5.0	1.13E-04
STC1	(-) Regulator of Intestinal Calcium Transport (9458778); Stanniocalcin SF	2.2	1.42E-02					2.2	3.42E-03			
STEAP2	Copper Metabolism (26934375); Iron Metabolism (16609065); Cytochrome b N SF	1.8	2.56E-02									
STEAP4	Copper Metabolism (16609065); Iron Metabolism (16609065); Cytochrome b N SF	4.0	5.38E-03								3.4	5.33E-03
TFRC	Iron Transport; Zinc Peptidase-like SF; TFR Dimer SF	2.8	3.47E-05					2.5	2.06E-05			
	**Vitamin Metabolism or Transport**											
AKR1C4	Vitamin A Metabolism (21851338); Aldo Ket Red SF										4.3	2.30E-05
DHRS9	Retinol Dehydrogenase; Vitamin A Metabolism (11304534); NADB Rossmann SF; SDR SF	3.1	4.24E-04					2.1	1.73E-02			
HCAR2	Negative Regulator of Inflammation (23882124); Nicotinic Acid Receptor; GPCR SF	13.3	7.26E-04								9.3	6.76E-05
MTHFD1	Folic Acid Metabolism; THF DHG CYH SF	1.5	1.94E-02									
MTHFD2	Folic Acid Metabolism; THF DHG CYH SF; NADB Rossman SF	1.8	2.97E-03									
PANK1	Coenzyme A (CoA) Biosynthesis; Pantothenate Metabolism; Fumble SF	1.9	9.77E-03					1.7	2.78E-02			
PANK3	Coenzyme A (CoA) Biosynthesis; Pantothenate Metabolism; NBD Sugar Kinase HSP70 Actin SF	2.2	4.27E-02									
SLC23A1	Ascorbic Acid Transport (23177992); Sulfate Transport SF; Solute Carrier SF	1.9	3.44E-03									
SLC2A1	Ascorbic Acid Transport (23177992); Solute Carrier SF	1.7	7.88E-03									
SLC2A3	Ascorbic Acid Transport (23177992); Solute Carrier SF	3.4	1.42E-12		2.7	1.71E-10						
STRA6	Vitamin A Metabolism (17255476) RBP Receptor SF							3.8	6.03E-03			
TCN1	Vitamin B12 Transport; Cobalamin bind SF	54.1	1.15E-04		19.2	1.29E-02					8.0	1.44E-10
	**Miscellaneous Metabolism or Transport**											
ABCC13	Orphan Transport; ABC Membrane SF; P-loop NTPase SF											
AKR1C1	Steroid Hormone Metabolism (25583233); Aldo Ket Red SF	7.0	5.11E-02								13.8	3.93E-07
CA4	Bicarbonate Transport (15563508); Alpha CA SF	4.3	9.18E-05								5.5	9.51E-10
CLIC2	Chloride Transport; Chloride Intracellular Channel Protein 1 SF	1.6	2.84E-02									
DIO2	Selenoprotein; Thyroid Hormone Metabolism; Thioredoxin-like SF	6.5	3.02E-04		5.8	1.16E-03					3.5	1.15E-03
IDE	(-) Regulator of Insulin Receptor Signaling (12773120); Peptidase M16 SF	1.6	7.53E-03									
KCNE3	Voltage-gated Potassium Channel; ISK Channel SF							1.6	6.59E-03		2.6	3.51E-05
KCNJ11	Potassium Channel; Ion Trans 2 SF										2.7	3.31E-02
RHCG	Solute Carrier SF	8.0	4.93E-02					19.8	5.18E-04			
SCNN1D	Epithelial Sodium Channel (ENaC) Family (26772908); ASC SF	2.4	1.45E-02									
SLC12A4	Potassium-chloride Cotransport; Solute Carrier SF	1.5	4.15E-03		1.5	1.59E-03						
SLC15A2	Oligopeptide Transport; Solute Carrier SF	2.4	2.11E-03									
SLC16A3	Lactate Transport; Monocarboxylate Transport; Solute Carrier SF	2.6	1.23E-03		2.4	2.50E-03						
SLC17A9	Nucleotide Transport; Solute Carrier SF	2.2	1.07E-05					1.6	5.72E-03			
SLC20A1	Phosphate Transport; Solute Carrier SF	1.7	1.63E-02									
SLC24A5	Sodium/Potassium/Calcium Exchange; Solute Carrier SF; Na Ca Ex SF							1.9	4.98E-02			
SLC25A14	Mitochondrial Carrier Protein SF; Solute Carrier SF										2.0	4.03E-02
SLC26A2	Sulfate Transport (7923357); Solute Carrier SF				1.8	5.24E-02						
SLC35A3	Nucleotide Sugar Transport; Solute Carrier SF	2.1	3.51E-03					1.7	1.61E-02			
SLC35B1	UDP-galactose Transport (9010752); Solute Carrier SF	1.5	2.46E-02									
SLC35C1	GDP-fucose Transport; EamA SF; Solute Carrier SF	1.5	4.14E-02					1.6	7.89E-03		1.8	4.89E-02
SLC4A11	Bicarbonate Transporter (15525507); Borate Transporter (15525507)	4.8	5.93E-03		6.9	1.04E-03						
SLCO1A2	Bile Acid Transport (9398014); Solute Carrier SF										8.6	6.00E-04
SLCO4A1	Steroid Sulfate Transport; Solute Carrier SF	3.1	7.29E-05		2.1	1.73E-02						
SPHK1	Biosynthesis of Sphingosine 1-phosphate; DAGK Cat SF	2.8	3.05E-03					2.8	3.05E-03			
SULT2A1	Sulfation of Bile Acids/Steroids; Sulfotransfer 3 SF	13.4	4.28E-02					7.5	5.36E-02			
	**Transcription Factors**											
SREBF1	Lipid Metabolism; Transcription Factor (12177166); HLH SF	2.0	1.67E-04									

Supplemental Table S10: Clustered Metabolism Genes (DEGs) - Down-regulated

Gene	Functional Categorization	52 DPI w worms vs Control Fold	EDGE FDR Adj p value	52 DPI w/o worms vs Control Fold	EDGE FDR Adj p value	(52 DPI Inf w/o worms vs w Worms Fold	EDGE FDR Adj p value	(21 DPI Inf vs Control) Fold	EDGE FDR Adj p value
	**Amino Acid Metabolism or Transport**								
ACADSB	BCAA Metabolism (21430231); Leucine Metabolism (21430231); ACAD SF	-1.6	1.66E-03						
AHCYL2	Homocysteine Metabolism; FDH GDH-like SF	-2.4	5.80E-06			-2.1	5.97E-05	-1.8	2.15E-02
AOC1	Arginine Metabolism; Tryptophan Metabolism; Cu Amine Oxid SF					-2.4	4.46E-05		
ASPA	Asparate Metabolism (9407392) ; Peptidase M14-like SF	-2.9	4.20E-03					-2.8	1.01E-02
IVD	Branched-chain Amino Acid Metabolism (25287287); ACAD SF	-1.6	2.32E-02						
PIPOX	Lysine Catabolism (10642506); NAD Binding 8 SF	-4.9	6.45E-03						
SLC25A34	Isopropylmalate Transporter (18682385); Solute Carrier SF	-7.8	1.41E-09			-4.2	1.12E-05		
SLC25A38	Glycine Transporter (26821380); Solute Carrier SF	-1.9	4.57E-04						
SLC38A4	Arginine/Glycine/Histidine/Lysine Transporter (11342143); Solute Carrier SF	-7.7	3.14E-09	-2.6	5.46E-04	-3.0	3.10E-03		
SLC43A2	BCAA/Methionine/Phenylalanine Transporter (15659399); Solute Carrier SF	-1.8	2.00E-03	-1.6	2.36E-02				
SLC6A6	Taurine Transporter (8010975); SLC5-6-like SBD SF; Solute Carrier SF	-1.6	5.12E-02						
SLC7A3L2*	Orphan Transporter; Spore Permease SF; Solute Carrier SF	-1.8	4.24E-04						
SLC7A3L4*	Orphan Transporter; Spore Permease SF; Solute Carrier SF	-2.6	4.44E-03			-2.1	4.13E-02		
SLC7A3L6*	Orphan Transporter; Spore Permease SF; Solute Carrier SF	-2.8	1.70E-02						
SLC7A3L7*	Orphan Transporter; Spore Permease SF; Solute Carrier SF	-2.4	3.11E-02			-2.3	4.90E-02	-3.6	4.06E-04
SLC7A3L8*	Orphan Transporter; Spore Permease SF; Solute Carrier SF	-1.7	1.79E-02						
SLC7A8	Alanine/Phenylalanine Transporter; Solute Carrier SF	-3.1	2.76E-05			-2.1	5.64E-03		
THNSL2	O-phospho-homoserine Catabolism (17034760); Trp-synth-beta II SF			-2.2	2.04E-04				
	**Carbohydrate Metabolism or Transport**								
AKR1E2	Fructose Metabolism (9504428); Aldo Ket Red SF	-1.9	3.20E-03			-1.8	4.12E-03		
ALDOB	Fructose Metabolism; Glycolysis; Aldolase Class I SF	-27.7	2.35E-07					-10.6	8.40E-19
FBP2	Fructose Metabolism (16213487); Gluconeogenesis (18780768); FIG SF	-1.6	1.05E-02						
PCK1	Gluconeogenesis (20124556); PEPCK HprK SF	-16.9	1.62E-07	-3.1	1.86E-02	-5.4	1.48E-03		
PDK2	Gluconeogenesis (12682057); BCDHK Adom3 SF; HATPase C SF	-1.9	1.11E-03	-1.6	1.50E-02				
PDK4	Gluconeogenesis; BCDHK Adom3 SF; HATPase C SF	-7.7	2.11E-04	-3.0	3.61E-02			-4.6	5.99E-03
PFKFB4	Fructose Metabolism (10095107); Glycolysis; P-loop NTPase SF	-2.0	4.11E-05						
PGM5	Biosynthesis of Glucose-6-phosphate (8586438); Phosphohexomutase SF							-2.1	8.60E-04
SI	Isomaltose/Sucrose Metabolism (7002920); Trefoil SF; GH31 SF	-7.9	8.57E-03			-6.1	2.79E-02		
SLC2A4	Ascorbic Acid/Class I Glucose Transporter Family Member (23177992); Solute Carrier SF	-1.8	1.06E-03						
SLC45A3	Glucose Transporter (22521588); Solute Carrier SF					-1.6	2.17E-02		
	**Lipid Metabolism or Transport**								
A1CF	Lipid Metabolism (29083408); RNA Recognition Motif (RRM) SF; DSRM SF			-1.6	1.73E-02				
ABCA1	Cholesterol Metabolism; Lipid Metabolism; ABC ATPase SF					-1.7	5.31E-02	-1.9	5.62E-03
ABCA6	Macrophage Cholesterol Metabolism (11478798); ABC ATPase SF	-6.5	3.05E-06			-4.4	1.88E-04	-6.4	3.40E-08
ABCA8	Bile Acid Transporter (12379217); ABC ATPase SF	-4.1	3.01E-03			-2.7	4.77E-02	-6.8	4.42E-10
ABCA9	Macrophage Cholesterol Transport (12150964); ABC ATPase SF	-2.2	2.05E-03			-2.7	5.24E-02	-5.5	3.06E-08
ABCB4	Bile Acid Phospholipid Transport; ABC ATPase SF	-2.7	2.10E-02						
ABCC6	Cholesterol Transport (25064003); ABC ATPase SF	-2.1	2.65E-05						
ACAD10	Fatty Acid Oxidation (21237683); ACAD SF	-1.6	1.02E-02						
ACADS	Fatty Acid Beta Oxidation; Mitochondrial Lipid Metabolism (18977676); ACAD SF	-1.7	2.96E-03						
ACSBG1	Very Long-chain Fatty Acid Oxidation (10954726); Acyl-CoA Synthetase SF (17762044)	-2.8	4.00E-02						
ACSL6	Lipid Metabolism; Long-chain ACS Family; AFD Class I SF; Acyl-CoA Synthetase SF (17762044)							-3.7	9.52E-03
APOE	Cholesterol Metabolism; Apolipoprotein A1/A4/E SF	-1.7	3.18E-02						
APOL2	ApoL SF	-2.4	1.75E-04			-1.7	3.42E-02		
BDH1	Fatty Acid Catabolism (8679568); NADB Rossmann SF			-1.7	5.79E-03	-1.7	7.89E-03		
CES1B*	Cholesterol Metabolism; Lipid Metabolism; Carboxylesterase Family	-2.1	5.66E-04	-1.7	7.07E-03				
CYP4V2	Fatty Acid Metabolism (19661213); Cytochrome P450 SF							-2.0	1.52E-02
CYP7B1	Bile Acid Synthesis (24927729); Cytochrome p450 SF					-2.0	1.59E-02	-1.7	4.83E-02
FAAH	Lipid Metabolism; Amidase SF	-2.7	6.61E-04						
FABP1	Lipid Metabolism (17449472); Lipocalin SF	-3.7	5.00E-05			-2.4	1.03E-02		
FABP3L2*	Lipocalin SF							-44.6	1.84E-04
FABP4	Lipid Metabolism (11891416); Lipocalin SF	-2.7	1.86E-02						
GPIHBP1	Lipoprotein Metabolism (17997385); LU SF	-5.3	6.94E-04	-2.5	3.06E-02				
HADHA	Long Chain Fatty Acid Beta Oxidation (8135828); Crotonase-like SF; AdoMet MTases SF	-1.7	1.84E-04	-1.5	1.16E-03				
LIPA	Lipid Metabolism (16131527); Abhydro Lipase SF	-1.5	3.68E-02						
LIPE	Lipid Metabolism; HSL N SF	-1.7	3.48E-02						
LIPM	Abhydro Lpase SF							-19.0	2.62E-02
MOGAT2	Triglyceride Biosynthesis (25164810); LPLAT SF	-9.2	1.14E-03			-4.9	2.97E-02	-5.4	8.78E-03
OSBPL10	Cholesterol Metabolism (19554302); H-like SF; Oxysterol BP SF	-1.9	6.89E-03						
PLIN1	Inhibition of Lipolysis (17353663); Perilipin SF	-4.0	3.06E-02					-4.3	4.50E-05
PLTP	Lipoprotein Metabolism; BPI SF	-1.7	1.75E-02						
PNPLA2	Lipid Metabolism (18445677); Patatin and cPLA2 SF	-2.4	1.05E-06	-1.6	1.01E-02	-1.5	3.42E-02		
PNPLA6	Phosphatidylcholine Catabolism (15044461); CAP ED SF; Patatin and cPLA2 SF	-1.6	2.08E-02			-1.6	6.03E-03		
PNPLA7	Lysophospholipase (18086666); CAP ED SF; Patatin and cPLA2 SF	-1.6	4.92E-03						
PON1	Lipid Metabolism (19550323); Str Synth SF			-4.5	4.13E-02				
PPAP2B	Sphingosine-1-phosphate (S1P) Metabolism (12471011); PAP2-like SF							-1.8	1.44E-02
SCARB2	Cholesterol Metabolism (14570588); SRCR SF	-1.6	1.71E-03			-1.6	5.21E-04		
SCARF1	Lipid Metabolism (15145948); EGF Lam SF	-1.5	4.78E-02						
SLC10A2	Bile Acid Transporter; Lipid Metabolism; Solute Carrier SF	-1285.8	8.30E-08	-4.3	4.13E-02	-297.1	1.18E-04		
SLC16A1	Short-chain Fatty Acid Transporter; Solute Carrier SF	-5.7	0.00E+00			-4.4	0.00E+00	-2.2	1.59E-03
SLC25A20	Carnitine Transporter; Fatty Acid Metabolism; Solute Carrier SF	-2.1	1.13E-04						
SLC25A29	Mitochondrial Carnitine Transporter (12882971, 19287344); Solute Carrier SF	-1.9	1.01E-02						
SLC25A42	Coenzyme A Transporter (19429682); Solute Carrier SF					-2.1	7.36E-03		
SLC27A1	Fatty Acid Transporter; Lipid Metabolism; Solute Carrier SF	-1.7	3.17E-02						
TM6SF2	Lipid Metabolism (24927523); DUF2781 SF	-6.3	2.26E-14			-4.0	1.95E-09		
	**Mineral Metabolism or Transport**								
ABCG2	Heme Transport (16487711); ABC ATPase SF	-7.6	6.40E-07			-3.4	2.49E-03	-3.5	9.73E-07
CLCA4	Chloride Ion Transport; CLCA N SF; vWFA SF	-27.9	1.34E-07						
FTL	Iron Storage; Ferritin-like SF	-1.6	4.64E-02						
PHEX	Phosphate Metabolism (22339660); GluZincin SF							-6.8	1.81E-02
SCARA5	Ferritin Receptor (19154717); Iron Metabolism (19154717); SRCR SF	-2.4	2.08E-02						
SELENBP1	Selenium Containing Protein; BP56 SF	-4.4	1.11E-11			-3.0	1.73E-07		
SELENOP	Selenoprotein (12423375); Selenoprotein P SF	-3.3	2.97E-08			-2.3	5.83E-05		
SLC25A3	Mitochondrial Phosphate Transporter (23266187); Solute Carrier SF							-1.9	2.19E-02
SLC26A3	Chloride Ion Transport (16715296); Solute Carrier SF	-12.7	8.30E-08			-12.0	9.52E-08	-2.5	8.05E-03
SLC30A10	Mg Transporter (22341972); Zn Transporter (22706290); Solute Carrier SF	-10.2	3.00E-03			-6.3	2.38E-02	-9.4	2.71E-13
SLC30A3	Zn Transporter; Solute Carrier SF	-3.1	8.38E-03						
SLC31A2	Copper Transporter (9207117); Solute Carrier SF	-1.8	2.06E-02						
SLC39A5	Zn Transporter (15322118); Solute Carrier SF	-2.3	3.63E-02						
SLC8A1	Intestinal Calcium Transport (26109800); Solute Carrier SF					-1.6	5.29E-02	-2.1	2.28E-03
	**Vitamin Metabolism or Transport**								
ALDH1A3	Retinoic Acid Aynthesis Vitamin A Metabolism; ALDH-SF SF					-2.0	1.10E-02		
CYP26B1	Retinoic Acid Catabolism; Vitamin A Metabolism; Cytochrome P450 SF	-5.0	2.61E-08			-4.6	4.16E-08	-4.8	9.76E-03
CYP27A1	Vitamin D Metabolism (15353333); Cytochrome P450 SF	-1.8	3.99E-02						
MTHFR	Folic Acid Metabolism; MTHFR SF	-1.8	7.98E-03						
NADK	NADPH Metabolism (17855339); nadF SF					-1.6	4.96E-03		
RBP5	Vitamin A Metabolism (11274389); Lipocalin SF	-2.4	5.79E-03						
RDH10	Retinol Dehydrogenase; Vitamin A Metabolism; NADB Rossmann SF; SDR SF							-2.0	2.25E-02
RDH16	Retinol Dehydrogenase; Vitamin A Metabolism; NADB Rossmann SF; SDR SF	-6.1	3.20E-03	-2.8	4.89E-02				
RETSAT	Vitamin A Metabolism (15358783); NADB Rossmann SF; COG1233 SF	-2.2	4.10E-06			-1.6	7.89E-03		
SDR9C7	Putative Retinol Dehydrogenase (19703561); NADB Rossmann SF; SDR SF	-9.3	8.99E-03	-4.2	2.26E-02				
SLC25A32	Folate Transporter (10978331); Solute Carrier SF								
TCN2	Vitamin B12 Transporter; Cobalamin Bind SF; DUF4430 SF	-1.5	3.88E-02						
VDR	Vitamin D3 Signaling; Transcription Factor; NR-DBD SF	-1.7	7.90E-03						
	**Miscellaneous Metabolism or Transport**								
ABCB1	ATP-dependent Xenobiotic Efflux; ABC Membrane SF; P-loop NTPase SF					-2.0	7.10E-03		
ABCB6	Coproporphyrinogen III Transporter (23792964); ABC Membrane SF					-1.6	5.08E-03		
ABCC4L2*	Orphan Transporter; ABC Membrane SF; P-loop NTPase SF							-3.4	2.05E-10

ABCC4L3*	Orphan Transporter; ABC Membrane SF; P-loop NTPase SF										-23.7	8.32E-19
ABCC4L4*	Orphan Transporter; ABC Membrane SF; P-loop NTPase SF										-12.2	7.35E-07
ADA	Adenosine Metabolism; Metallo-dependent Hydrolases SF	-1.8	1.88E-02									
GGT1	Glutathione Metabolism; G Glu Transpept SF							-1.9	4.29E-02			
HSD17B1	Biosynthesis of Estrogen (27102893); Androgen Catabolism; NADB Rossmann SF	-1.8	4.04E-02									
HSD17B6	Androgen Catabolism (9188497); NADB Rossmann SF	-3.7	1.19E-02									
INPP5K	(-) Regulator of Insulin Signaling (12556481); EEP SF	-1.6	7.22E-03									
OAZ3	Polyamine Metabolism (19076071); Cupredoxin SF; ODC AZ SF										-8.8	2.23E-02
SLC12A7	Potassium-chloride Cotransporter; Solute Carrier SF	-1.5	2.28E-02									
SLC13A1	Sulfate Transporter (11161786); Solute Carrier SF	-1.5	4.47E-02									
SLC14A1	Urea Transporter; UT SF; Solute Carrier SF	-13.9	1.46E-02								-7.9	9.93E-11
SLC16A14	Orphan Transporter; Solute Carrier SF				-1.7	1.09E-02						
SLC16A5	Orphan Monocarboxylate Transporter; Solute Carrier SF	-2.1	1.59E-03					-1.8	9.64E-03			
SLC22A10L3*	Orphan Transporter; Major Facilitator SF; Solute Carrier SF										-14.0	1.75E-03
SLC24A1	Sodium/Calcium Transport (7539424); Solute Carrier SF; Na Ca Ex SF				-2.9	1.16E-03						
SLC24A4	Sodium/Calcium Transport; Solute Carrier SF; Na Ca Ex SF	-4.4	2.68E-06		-1.8	4.67E-02		-2.4	1.05E-02		-4.9	8.53E-03
SLC25A27	Adenine Transporter; Solute Carrier SF							-1.6	7.18E-03			
SLC25A5	Mitochondrial Proton Transport; Solute Carrier SF	-1.5	2.24E-02									
SLC25A53	Mitochondrial Protein; Orphan Transporter (23266187); Solute Carrier SF										-3.5	2.19E-02
SLC25A6	Adenine Transporter; Solute Carrier SF	-1.5	2.26E-02									
SLC44A4	Choline Transporter; Solute Carrier SF	-1.6	3.27E-03									
SLC46A2	Peptidoglycan Transporter (28539433); Solute Carrier SF	-2.3	6.48E-03									
SLC46A3	Orphan Transporter; Solute Carrier SF	-2.8	2.40E-06		-1.8	2.05E-03						
SLC4A7	Bicarbonate Cotransporter; HCO3- Cotransporter SF; Solute Carrier SF										-2.0	2.41E-02
SLC51A	Bile Acid Transporter (18292224) ; Solute Carrier SF	-6.9	2.76E-08					-4.8	3.48E-06			
SLC9A2	Sodium/Hydrogen Ion Transporter (26350456); Solute Carrier SF	-1.9	1.78E-04					-1.7	1.92E-03			
SLC9A3R1	(+) Regulator of Inflammation (24196963); PDZ SF; EBP50 C-term SF	-1.5	1.36E-02									
SULT1B1	Phase II Conjugating Enzyme; Sulfotransfer 3 SF				-1.5	3.48E-02						
UGT2C1*	Phase II Conjugating Enzyme; Glycosyltransferase GTB-type Superfamily				-1.6	4.13E-02						

Supplemental Table S11. Metabolic markers of *Trichuris suis* infection proximal colon tissue 33 dpi

Heat map of statistically significant biochemicals profiled in this study.

Comparison mean values significantly different: $p \leq 0.05$, fold of change ≥ 1.00 (red); $p \leq 0.05$, fold of change < 1.00 (green)

Comparison mean values approaching significance: $0.05 < p < 0.10$, fold of change ≥ 1.00 (pink); $0.05 < p < 0.10$, fold of change < 1.00 (light green)

Pathway Sort Order	Super Pathway	Sub Pathway	Biochemical Name	Platform	Comp ID	KEGG	HMDB	Fold of Change, Welch's Two-Sample t-Test: Infected / Non-Infected	Statistical Values, Welch's Two-Sample t-Test, Infected / Non-Infected: p-Value	q-Value	Mean Values: Non-Infected	Mean Values: Infected	% Filled Values: Non-Infected	% Filled Values: Infected	Super Pathway	Sub Pathway	PubChem	CAS	RI	Mass
1		Glycine, serine and threonine metabolism	glycine	GC/MS	11777	C00037	HMDB00123	1.39	0.0099	0.0697	0.9159	1.2731	100	100	Amino acid	Glycine, serine and threonine metabolism	5257127;750	56-40-6;	1166	101.9
2			sarcosine (N-Methylglycine)	GC/MS	1516	C00213	HMDB00271	1.43	0.0054	0.0494	0.9033	1.2879	100	100	Amino acid	Glycine, serine and threonine metabolism	1088;7311726	107-97-1;	1182.9	116
8			serine	GC/MS	1648	C00065	HMDB00187	1.14	0.1446	0.3086	0.9303	1.0621	100	100	Amino acid	Glycine, serine and threonine metabolism	5951;6857581	56-45-1;	1389.1	204
17			threonine	LC/MS pos	1284	C00188	HMDB00167	0.82	0.0329	0.1354	1.0939	0.8941	100	100	Amino acid	Glycine, serine and threonine metabolism	6971019;6288	72-19-5;	713	120.1
18			N-acetylthreonine	LC/MS neg	33939	C01118		0.95	0.5938	0.6139	1.0107	0.9643	100	100	Amino acid	Glycine, serine and threonine metabolism	4651717	17093-74-2;	846	160.1
21			betaine	LC/MS pos	3141	C00719	HMDB00043	1.29	0.0925	0.2277	0.8621	1.1114	100	100	Amino acid	Glycine, serine and threonine metabolism	247	107-43-7;	721	118.2
24		Alanine and aspartate metabolism	aspartate	GC/MS	15996	C00049	HMDB00191	1.20	0.0697	0.1960	0.9144	1.0957	100	100	Amino acid	Alanine and aspartate metabolism	5960	56-84-8;	1529.7	232
25			asparagine	GC/MS	11398	C00152	HMDB00168	1.07	0.6201	0.6226	1.0459	1.1202	100	100	Amino acid	Alanine and aspartate metabolism	6267;6992089	70-47-3;	1651.2	231
28			beta-alanine	GC/MS	55	C00099	HMDB00056	0.87	0.8148	0.6977	1.1333	0.9866	100	100	Amino acid	Alanine and aspartate metabolism	239;4755801	56-41-7;107-95-9;	1451.8	174
32			alanine	GC/MS	1126	C00041	HMDB00161	1.29	0.0131	0.0840	0.8843	1.1382	100	100	Amino acid	Alanine and aspartate metabolism	5950;7311724	56-41-7;	1147.6	115.9
33			N-acetylalanine	LC/MS neg	1585	C02847	HMDB00766	1.10	0.2848	0.4400	0.9321	1.0219	100	100	Amino acid	Alanine and aspartate metabolism	88064	97-69-8;	882	130.1
35			N-acetylaspartate (NAA)	GC/MS	22185	C01042	HMDB00812	0.90	0.9791	0.7469	1.3260	1.1878	78	100	Amino acid	Alanine and aspartate metabolism	65065	997-55-7;997-55-7 ;	1648.5	274
39		Glutamate metabolism	glutamate	GC/MS	57	C00025	HMDB03339	1.21	0.0268	0.1249	0.9276	1.1234	100	100	Amino acid	Glutamate metabolism	611	56-86-0;	1611.9	246
45			glutamine	LC/MS pos	53	C00064	HMDB00641	1.02	0.6814	0.6517	0.9718	0.9914	100	100	Amino acid	Glutamate metabolism	5961	56-85-9;	684	147.2
47			gamma-aminobutyrate (GABA)	GC/MS	1416	C00334	HMDB00112	0.59	0.0072	0.0593	1.5358	0.9028	100	100	Amino acid	Glutamate metabolism	6992099;119	56-12-2;	1539.7	304.1
51			N-acetyl-aspartyl-glutamate (NAAG)	LC/MS pos	35665	C12270	HMDB01067	0.92	0.1903	0.3551	1.0181	0.9330	100	100	Amino acid	Glutamate metabolism	5255	3106-85-2;	1563	305
53		Histidine metabolism	histidine	LC/MS neg	59	C00135	HMDB00177	0.97	0.4643	0.5473	1.0232	0.9898	100	100	Amino acid	Histidine metabolism	773;3651426	5934-29-2;	757	154.1
57			1-methylhistidine	GC/MS	30460	C01152	HMDB00001	1.47	0.0286	0.1264	0.9137	1.3401	89	100	Amino acid	Histidine metabolism	92105;7020397	332-80-9;	1757.7	196
58			histamine	LC/MS pos	1574	C00388	HMDB00870	0.79	0.1525	0.3197	1.1698	0.9270	100	100	Amino acid	Histidine metabolism	774	51-45-6;	570	112.2
59			1-methylhistamine	GC/MS	32441	C05127	HMDB00898	1.19	0.0969	0.2364	0.9073	1.0816	100	100	Amino acid	Histidine metabolism	3614	501-75-7 ;	1725	174.1
65			imidazole propionate	LC/MS pos	40730		HMDB02271	4.67	0.0564	0.1852	0.3909	1.8243	11	45	Amino acid	Histidine metabolism	70630	1074-59-5;	1011.7	141.1
75		Lysine metabolism	lysine	LC/MS pos	1301	C00047	HMDB00182	0.85	0.0386	0.1490	1.0227	0.8651	100	100	Amino acid	Lysine metabolism	5962	56-87-1;	603	147.2
77			2-aminoadipate	LC/MS pos	6146	C00956	HMDB00510	0.89	0.4438	0.5366	1.1835	1.0543	100	100	Amino acid	Lysine metabolism	469	542-32-5;1118-90-7;	794	162.1
80			pipecolate	LC/MS pos	1444	C00408	HMDB00070	1.96	0.0130	0.0840	0.8857	1.7332	100	100	Amino acid	Lysine metabolism	849	4043-87-2;	1120	130.1
85			N6-acetyllysine	LC/MS pos	36752	C02727	HMDB00206	1.00	0.9708	0.7469	1.0323	1.0307	100	100	Amino acid	Lysine metabolism	6991978;92832	692-04-6;	1134	189.1
89		Phenylalanine & tyrosine metabolism	phenylalanine	LC/MS pos	64	C00079	HMDB00159	0.96	0.4453	0.5366	1.0491	1.0103	100	100	Amino acid	Phenylalanine & tyrosine metabolism	6925665;6140	63-91-2;	2056	166.1
92			phenylacetate	LC/MS neg	15958	C07086	HMDB00209	0.48	0.0011	0.0151	1.5789	0.7650	100	100	Amino acid	Phenylalanine & tyrosine metabolism		103-82-2;	2127	135.1
94			p-cresol sulfate	LC/MS neg	36103	C01468	HMDB11635	0.85	0.0828	0.2200	1.1028	0.9358	100	100	Amino acid	Phenylalanine & tyrosine metabolism	4615422	3233-57-7;	2896	187.1
106			tyrosine	LC/MS pos	1299	C00082	HMDB00158	0.96	0.3868	0.5079	1.0358	0.9895	100	100	Amino acid	Phenylalanine & tyrosine metabolism	6057;6942100	60-18-4;	1516	182.1
107			3-(4-hydroxyphenyl)lactate	LC/MS neg	32197	C03672	HMDB00755	0.88	0.2637	0.4250	1.1230	0.9836	100	100	Amino acid	Phenylalanine & tyrosine metabolism	9378	6482-98-0;	1395	181.1
124			4-hydroxyphenylacetate	GC/MS	541	C00642	HMDB00020	2.07	0.0195	0.1000	0.5715	1.1804	56	82	Amino acid	Phenylalanine & tyrosine metabolism	4693933	156-38-7;	1630.6	179
134			phenylacetylglycine	LC/MS neg	33945	C05598	HMDB00821	0.97	0.7949	0.6923	1.0824	1.0537	100	100	Amino acid	Phenylalanine & tyrosine metabolism	68144	500-98-1;	2377	192.1
138			3-(3-hydroxyphenyl)propionate	LC/MS neg	35635	C11457	HMDB00375	1.42	0.5175	0.5812	0.9896	1.4062	78	73	Amino acid	Phenylalanine & tyrosine metabolism	91	621-54-5;	2000	165.1
140			3-phenylpropionate (hydrocinnamate)	LC/MS neg	15749	C05629	HMDB00764	0.57	0.0053	0.0494	1.2867	0.7297	100	100	Amino acid	Phenylalanine & tyrosine metabolism		501-52-0;	2830	149.1
152		Tryptophan metabolism	kynurenine	LC/MS pos	15140	C00328	HMDB00684	1.09	0.5959	0.6139	1.0416	1.1307	89	91	Amino acid	Tryptophan metabolism	161166;6971029	2922-83-0;	1902	209.1
153			tryptophan	LC/MS pos	54	C00078	HMDB00929	0.88	0.0282	0.1264	1.0838	0.9509	100	100	Amino acid	Tryptophan metabolism	6923516;6305	73-22-3;	2445	205.1
156			indoleacetate	LC/MS pos	27513	C00954	HMDB00197	2.18	0.0537	0.1809	0.5530	1.2038	56	82	Amino acid	Tryptophan metabolism	802	6505-45-9;	3760	176.1
160.1			5-hydroxytryptophol	GC/MS	41498		HMDB01855	0.52	0.0868	0.2246	1.5291	0.8004	100	100	Amino acid	Tryptophan metabolism	9061	154-02-9;	1956	290.1
172			C-glycosyltryptophan*	LC/MS pos	32675			1.15	0.1651	0.3278	0.9561	1.1026	100	100	Amino acid	Tryptophan metabolism			1912	367.1
179	Amino acid		3-indoxyl sulfate	LC/MS neg	27672		HMDB00682	0.80	0.8613	0.7207	1.4758	1.1850	100	100	Amino acid	Tryptophan metabolism	10258	2642-37-7;	2258	212
190		Valine, leucine and isoleucine metabolism	isoleucine	LC/MS pos	1125	C00407	HMDB00172	0.93	0.1092	0.2554	1.0261	0.9567	100	100	Amino acid	Valine, leucine and isoleucine metabolism	791	73-32-5;	1614	132.1
191			leucine	LC/MS pos	60	C00123	HMDB00687	1.01	0.9720	0.7469	1.0249	1.0321	100	100	Amino acid	Valine, leucine and isoleucine metabolism	7045798;6106	61-90-5;	1674	132.2
200			valine	LC/MS pos	1649	C00183	HMDB00883	0.92	0.0678	0.1953	1.0450	0.9589	100	100	Amino acid	Valine, leucine and isoleucine metabolism	6971018;6287	72-18-4;	1040	118.1
210			isobutyrylcarnitine	LC/MS pos	33441		HMDB00736	1.08	0.3964	0.5108	0.9455	1.0250	100	100	Amino acid	Valine, leucine and isoleucine metabolism		25518-49-4;	1941	232.2
212			2-methylbutyrylcarnitine (C5)	LC/MS pos	35431		HMDB00378	0.95	0.9743	0.7469	1.0702	1.0206	100	100	Amino acid	Valine, leucine and isoleucine metabolism	6426901	31023-25-3;	2439	246.1
215			isovalerylcarnitine	LC/MS pos	34407		HMDB00688	0.63	0.0230	0.1131	1.4649	0.9209	100	100	Amino acid	Valine, leucine and isoleucine metabolism	6426851	31023-24-2;	2533	246.2
216			hydroxyisovaleroyl carnitine	LC/MS pos	35433			0.86	0.2096	0.3702	1.0988	0.9493	100	100	Amino acid	Valine, leucine and isoleucine metabolism		99159-87-2;	1683	262.1
224		Cysteine, methionine, SAM, taurine metabolism	cysteine	GC/MS	31453	C00097	HMDB00574	1.05	0.9059	0.7356	1.0521	1.1032	100	100	Amino acid	Cysteine, methionine, SAM, taurine metabolism	5862;6419722	52-90-4;56-89-3;	1560.1	218
227			S-methylcysteine	GC/MS	40262		HMDB02108	1.31	0.0833	0.2200	0.9961	1.3026	100	100	Amino acid	Cysteine, methionine, SAM, taurine metabolism	7058174;24417	1187-84-4;	1447.8	162.1
228			cystine	GC/MS	31454	C00491	HMDB00192	1.18	0.5837	0.6132	0.7465	0.8782	44	73	Amino acid	Cysteine, methionine, SAM, taurine metabolism	595	56-89-3;	2015.3	218
229			cystathionine	GC/MS	15705	C02291	HMDB00099	3.34	0.0000	0.0001	0.5397	1.8019	100	100	Amino acid	Cysteine, methionine, SAM, taurine metabolism	4083111;834	535-34-2;	1979.6	218.1
230			methionine sulfoxide	LC/MS pos	18374	C02989	HMDB02005	0.73	0.0084	0.0617	1.1166	0.8157	100	82	Amino acid	Cysteine, methionine, SAM, taurine metabolism	158980;1548907	3226-65-1;	729	166.1
232			hypotaurine	GC/MS	590	C00519	HMDB00965	0.96	0.9418	0.7466	1.0314	0.9928	100	100	Amino acid	Cysteine, methionine, SAM, taurine metabolism	107812	300-84-5;	1598.5	188
233			taurine	LC/MS pos	32975	C00245	HMDB00251	0.78	0.0807	0.2176	1.1358	0.8908	100	100	Amino acid	Cysteine, methionine, SAM, taurine metabolism	1123;4068592	107-35-7;	687	126.1
236			S-adenosylhomocysteine (SAH)	LC/MS neg	15948	C00021	HMDB00939	1.15	0.6484	0.6435	1.0515	1.2051	100	91	Amino acid	Cysteine, methionine, SAM, taurine metabolism		979-92-0;	1821	383.1
237			methionine	LC/MS pos	1302	C00073	HMDB00696	0.94	0.3680	0.4959	1.0270	0.9703	100	100	Amino acid	Cysteine, methionine, SAM, taurine metabolism	6992087;6137	63-68-3;	1252	150.1
239			N-acetylmethionine	LC/MS neg	1589	C02712	HMDB11745	1.13	0.0631	0.1915	0.9717	1.0940	100	100	Amino acid	Cysteine, methionine, SAM, taurine metabolism	448580	65-82-7;	1805	190.1
241			2-hydroxybutyrate (AHB)	GC/MS	21044	C05984	HMDB00008	0.91	0.2824	0.4400	0.9941	0.9029	100	82	Amino acid	Cysteine, methionine, SAM, taurine metabolism	440864	3347-90-8;	1169.4	130.9
243			homocysteine	GC/MS	40266	C00155	HMDB00742	1.20	0.3303	0.4720	0.8002	0.9623	78	100	Amino acid	Cysteine, methionine, SAM, taurine metabolism	778	454-29-5;	1648.2	234
254		Urea cycle; arginine-, proline-, metabolism	dimethylarginine (SDMA + ADMA)	LC/MS pos	36808	C03626	HMDB01539	0.99	0.8552	0.7203	0.9634	0.9539	100	100	Amino acid	Urea cycle; arginine-, proline-, metabolism	123831	102783-24-4;	812	203.2
255			arginine	LC/MS pos	1638	C00062	HMDB00517	0.96	0.2640	0.4250	1.0159	0.9754	100	100	Amino acid	Urea cycle; arginine-, proline-, metabolism	5246487;232	1119-34-2;	650	175.2
258			ornithine	GC/MS	1493	C00077	HMDB03374	1.22	0.2020	0.3647	0.9085	1.1039	100	100	Amino acid	Urea cycle; arginine-, proline-, metabolism	6262	3184-13-2;	1763.8	141.9
260			urea	GC/MS	1670	C00086	HMDB00294	1.12	0.4200	0.5220	2.7828	3.1248	89	91	Amino acid	Urea cycle; arginine-, proline-, metabolism	1176;16150869	57-13-6;	1223.9	171
261			proline	LC/MS pos	1898	C00148	HMDB00162	1.04	0.6531	0.6457	1.0059	1.0417	100	100	Amino acid	Urea cycle; arginine-, proline-, metabolism	145742;6971047	147-85-3;	796	116.1
262			5-aminovalerate	GC/MS	18319	C00431	HMDB03355	3.55	0.0007	0.0124	0.8308	2.9530	100	100	Amino acid	Urea cycle; arginine-, proline-, metabolism	6992101;138	660-88-8;	1620.8	174
263			citrulline	LC/MS pos	2132	C00327	HMDB00904	1.09	0.3811	0.5050	1.0813	1.1738	100	100	Amino acid	Urea cycle; arginine-, proline-, metabolism	833	372-75-8;	715	176.1
264			N-acetylornithine	LC/MS pos	15630	C00437	HMDB03357	1.74	0.0495	0.1688	1.0447	1.8209	100	100	Amino acid	Urea cycle; arginine-, proline-, metabolism	6992102;439232	6205-08-9;	875	175.2
267			trans-4-hydroxyproline	LC/MS pos	32306	C01157	HMDB00725	1.32	0.0446	0.1587	0.8038	1.0618	100	100	Amino acid	Urea cycle; arginine-, proline-, metabolism	5810;6971053	51-35-4;	705	132.1
270			argininosuccinate	GC/MS	15497	C03406	HMDB00052	0.82	0.3596	0.4923	1.0278	0.8449	67	73	Amino acid	Urea cycle; arginine-, proline-, metabolism	828	156637-58-0;	2157	446.3
274		Creatine metabolism	creatine	LC/MS pos	27718	C00300	HMDB00064	1.02	0.7652	0.6850	0.9872	1.0039	100	100	Amino acid	Creatine metabolism	586	57-00-1;	758	132.1
275			creatinine	LC/MS pos	513	C00791	HMDB00562	0.99	0.9960	0.7479	1.0175	1.0094	100	100	Amino acid	Creatine metabolism	588	60-27-5;	730	114.1
278		Butanoate metabolism	2-aminobutyrate	GC/MS	1577	C02261	HMDB00650	1.82	0.0007	0.0124	0.7497	1.3617	100	100	Amino acid	Butanoate metabolism	439691;6971251	1492-24-6;	1215.7	130
282		Polyamine metabolism	putrescine	GC/MS	1408	C00134	HMDB01414	0.99	0.4009	0.5129	1.3355	1.3282	100	100	Amino acid	Polyamine metabolism		110-60-1;	1705.8	174
285			spermidine	LC/MS pos	485	C00315	HMDB01257	0.77	0.0767	0.2136	1.2667	0.9703	100	100	Amino acid	Polyamine metabolism	1102	124-20-9;	533	146.2
295		Glutathione metabolism	glutathione, reduced (GSH)	LC/MS pos	2127	C00051	HMDB00125	3.01	0.0176	0.0959	0.6898	2.0767	100	100	Amino acid	Glutathione metabolism	124886	70-18-8;	1274	308.1
297			5-oxoproline	LC/MS pos	1494	C01879	HMDB00267	0.90	0.2933	0.4471	1.0976	0.9889	100	100	Amino acid	Glutathione metabolism	7405	98-79-3;	1446	130.1
298			glutathione, oxidized (GSSG)	LC/MS pos	27727	C00127	HMDB03337	1.08	0.3826	0.5050	0.9405	1.0124	100	100	Amino acid	Glutathione metabolism	65359;11215652	103239-24-3;	1535	613.1
299			cysteine-glutathione disulfide	LC/MS pos	35159		HMDB00656	0.87	0.7049	0.6611	1.0363	0.8987	100	100	Amino acid	Glutathione metabolism	4247235	13081-14-6;	821	427.1
300			ophthalmate	LC/MS pos	34592		HMDB05765	1.22	0.1520	0.3197	0.9472	1.1560	100	100	Amino acid	Glutathione metabolism	9817431	495-27-2;	1457	290.1
311		Dipeptide	glycylvaline	LC/MS pos	18357			0.90	0.5805	0.6132	1.1209	1.0079	89	100	Peptide	Dipeptide	2724807;6994979	1963-21-9;	1572	175.1
312.9			glycylmethionine	LC/MS pos	42012			1.06	0.7772	0.6875	1.0006	1.0623	100	91	Peptide	Dipeptide	96757	554-94-9;	1593	207.1
314			glycylproline	LC/MS pos	22171		HMDB00721	1.16	0.2470	0.4134	0.9161	1.0599	100	100	Peptide	Dipeptide	3013625;6993386	704-15-4;	1115	173.1
316			glycylleucine	LC/MS pos	34398	C02155	HMDB00759	1.00	0.9928	0.7479	0.9932	0.9912	100	100	Peptide	Dipeptide	92843;1548899	869-19-2;	2236	189.1
320			glycyltyrosine	LC/MS pos	33958			0.88	0.5824	0.6132	1.0775	0.9498	100	100	Peptide	Dipeptide	92829;6994980	658-79-7;	1728	239.1
333			alanylglycine	GC/MS	37075		HMDB06899	0.90	0.5925	0.6139	1.1058	0.9943	100	91	Peptide	Dipeptide	5242526;79094	687-69-4;	1412	272.1
360			leucylleucine	LC/MS pos	36756	C11332		0.49	0.0691	0.1960	1.1921	0.5783	67	45	Peptide	Dipeptide	76807;6992072	3303-31-9;	3012	245.1
363			pro-hydroxy-pro	LC/MS pos	35127		HMDB06695	0.81	0.5809	0.6132	1.3005	1.0478	100	100	Peptide	Dipeptide	11673055	18684-24-7;	960	229.2
376			cysteinylglycine	GC/MS	35637	C01419	HMDB00078	1.11	0.5632	0.6085	0.9529	1.0614	100	100	Peptide	Dipeptide	65270	19246-18-5;	1763	257
406			isoleucylglycine	LC/MS pos	40008			1.12	0.5166	0.5812	0.8638	0.9685	100	91	Peptide	Dipeptide	342532	868-28-0;	1732	189.2
413			leucylalanine	LC/MS pos	40010			0.73	0.3249	0.4720	0.9595	0.7033	44	45	Peptide	Dipeptide	259321	7298-84-2;	1760	203.2
418	Peptide		leucylglycine	LC/MS pos	40045			0.67	0.4037	0.5129	1.2188	0.8206	78	82	Peptide	Dipeptide	79070	686-50-0;	1800	189.2
438.9			tyrosylglycine	LC/MS pos	41375			1.07	0.7416	0.6826	0.9608	1.0320	100	100	Peptide	Dipeptide	259323	673-05-5;	1596.8	239.1
455		Dipeptide derivative	carnosine	LC/MS neg	1768	C00386	HMDB00033	0.87	0.3617	0.4926	1.0225	0.8887	100	100	Peptide	Dipeptide derivative	439224;6992100	305-84-0;	813	225.2
460		gamma-glutamyl	gamma-glutamylvaline	LC/MS pos	32393		HMDB11172	0.82	0.2343	0.3999	1.1251	0.9282	89	100	Peptide	gamma-glutamyl		2746-34-1;	2040	247.2
462			gamma-glutamylleucine	LC/MS pos	18369		HMDB11171	0.76	0.0982	0.2372	1.1004	0.8347	100	100	Peptide	gamma-glutamyl	151023	2566-39-4;	2744	261.2
465			gamma-glutamylglycine	GC/MS	33949		HMDB11667	1.39	0.2848	0.4400	0.8560	1.1861	78	91	Peptide	gamma-glutamyl	165527	1948-29-4;	1934	375.2
466			gamma-glutamylmethionine	LC/MS pos	37539			0.69	0.0394	0.1490	1.1887	0.8232	100	100	Peptide	gamma-glutamyl	7009567	17663-87-5;	1993	279.2
467			gamma-glutamylglutamate	LC/MS pos	36738			0.91	0.4480	0.5366	1.0561	0.9598	100	100	Peptide	gamma-glutamyl	92865	1116-22-9;	1036	277.1
468			gamma-glutamylglutamine	LC/MS pos	2730		HMDB11738	0.86	0.2358	0.3999	1.0965	0.9380	100	100	Peptide	gamma-glutamyl	150914	10148-81-9;	806	276.1
470			gamma-glutamylphenylalanine	LC/MS pos	33422		HMDB00594	0.73	0.1709	0.3366	1.1997	0.8709	100	100	Peptide	gamma-glutamyl	111299	7432-24-8;	2846	295.1
471			gamma-glutamyltyrosine	LC/MS pos	2734			0.69	0.0033	0.0354	1.1373	0.7860	100	82	Peptide	gamma-glutamyl	94340	7432-23-7;	2073	311.2
475			gamma-glutamylalanine	LC/MS pos	37063			1.14	0.3948	0.5108	0.9568	1.0946	100	100	Peptide	gamma-glutamyl	440103	5875-41-2;	1108	219
510		Aminosugars metabolism	N-acetylgalactosamine	GC/MS	2766	C01074	HMDB00835	1.99	0.0628	0.1915	0.8163	1.6259	78	91	Carbohydrate	Aminosugars metabolism	35717	14215-68-0;	1887.4	245.1
511			N-acetylglucosamine	GC/MS	15096	C00140	HMDB00215	1.65	0.0407	0.1490	0.8117	1.3391	100	100	Carbohydrate	Aminosugars metabolism	24139	7512-17-6;	1934.5	245
513			N-acetylglucosamine 6-phosphate	GC/MS	15107	C00357	HMDB02817	1.16	0.4138	0.5176	0.8159	0.9465	78	100	Carbohydrate	Aminosugars metabolism	10040501	102029-88-9;	2096.2	387.2
515			erythronate*	GC/MS	33477		HMDB00613	1.10	0.6949	0.6542	0.9912	1.0918	100	100	Carbohydrate	Aminosugars metabolism	2781043	88759-55-1;	1546.9	292.1
517			N-acetylneuraminate	GC/MS	1592	C00270	HMDB00230	2.47	0.0082	0.0617	0.5711	1.4091	89	100	Carbohydrate	Aminosugars metabolism		131-48-6;	2074.9	245
523			fucose	GC/MS	15821	C02095	HMDB00174	2.07	0.0037	0.0375	0.8793	1.8208	100	100	Carbohydrate	Aminosugars metabolism	3034656	2438-80-4;	1682.2	204
525			N-acetylmannosamine	GC/MS	15060	C00140	HMDB00835	2.75	0.0006	0.0123	0.7350	2.0220	67	100	Carbohydrate	Aminosugars metabolism	439281	7772-94-3;	1915.9	245.1
537		Fructose, mannose, galactose, starch, and sucrose metabolism	fructose	GC/MS	577	C00095	HMDB00660	1.20	0.3923	0.5108	0.8617	1.0379	89	100	Carbohydrate	ctose, mannose, galactose, starch, and sucrose metabo	5984	57-48-7;	1758	217
539			galactose	GC/MS	12055	C01582	HMDB00143	2.78	0.0028	0.0331	0.6196	1.7215	100	100	Carbohydrate	ctose, mannose, galactose, starch, and sucrose metabo	3037556	59-23-4;	1793.8	203.9
547			maltose	GC/MS	15806	C00208	HMDB00163	1.51	0.3649	0.4944	1.1267	1.6985	100	100	Carbohydrate	ctose, mannose, galactose, starch, and sucrose metabo	439341	6363-53-7;	2142.1	204.1
548			mannitol	GC/MS	15335	C00392	HMDB00765	1.08	0.8281	0.7067	0.8358	0.9047	78	82	Carbohydrate	ctose, mannose, galactose, starch, and sucrose metabo	6251	69-65-8;	1839	319.1
552			mannose	GC/MS	584	C00159	HMDB00169	1.26	0.2550	0.4213	0.9695	1.2216	100	100	Carbohydrate	ctose, mannose, galactose, starch, and sucrose metabo	161658	3458-28-4;	1753.5	203.9
554			mannose-6-phosphate	GC/MS	1470	C00275	HMDB01078	1.05	0.7464	0.6826	0.9521	0.9989	100	100	Carbohydrate	ctose, mannose, galactose, starch, and sucrose metabolism		70442-25-0;104872-94-8;	2035.9	387.2
560			sorbitol	GC/MS	15053	C00794	HMDB00247	1.42	0.0609	0.1915	0.8346	1.1854	100	100	Carbohydrate	ctose, mannose, galactose, starch, and sucrose metabo	107428	6706-59-8;	1843	319.1
583		Glycolysis, gluconeogenesis, pyruvate metabolism	1,5-anhydroglucitol (1,5-AG)	GC/MS	20675	C07326	HMDB02712	0.93	0.7318	0.6764	1.0040	0.9302	89	73	Carbohydrate	Glycolysis, gluconeogenesis, pyruvate metabolism		154-58-5;	1788.7	217.1
585	Carbohydrate		glycerate	GC/MS	1572	C00258	HMDB00139	0.76	0.1930	0.3555	1.2850	0.9777	100	100	Carbohydrate	Glycolysis, gluconeogenesis, pyruvate metabolism	752	600-19-1;	1360.7	189
587			glucose-6-phosphate (G6P)	GC/MS	31260	C00668	HMDB01401	1.04	0.6597	0.6484	0.9809	1.0226	100	100	Carbohydrate	Glycolysis, gluconeogenesis, pyruvate metabolism		103192-55-8;	2042.7	387.2
589			glucose	GC/MS	20488	C00031	HMDB00122	1.07	0.5434	0.5946	1.0269	1.0968	100	100	Carbohydrate	Glycolysis, gluconeogenesis, pyruvate metabolism	79025	50-99-7;	1866.8	217.1
592			fructose-6-phosphate	GC/MS	12021	C05345	HMDB00124	0.96	0.9357	0.7444	1.0084	0.9729	100	100	Carbohydrate	Glycolysis, gluconeogenesis, pyruvate metabolism		103213-47-4;	1994.9	315.1
597			3-phosphoglycerate	GC/MS	40264	C00597	HMDB00807	0.46	0.0250	0.1185	2.1428	0.9801	100	100	Carbohydrate	Glycolysis, gluconeogenesis, pyruvate metabolism		80731-10-8;	1754	357.1
602			phosphoenolpyruvate (PEP)	GC/MS	597	C00074	HMDB00263	0.54	0.1635	0.3270	1.2825	0.6868	89	73	Carbohydrate	Glycolysis, gluconeogenesis, pyruvate metabolism	1005	10526-80-4;	1595.4	369
604			lactate	GC/MS	527	C00186	HMDB00190	1.30	0.0061	0.0523	0.8663	1.1249	100	100	Carbohydrate	Glycolysis, gluconeogenesis, pyruvate metabolism	612	79-33-4;	1102.8	116.9
607			glucuronate	GC/MS	15443	C00191	HMDB00127	0.92	0.6126	0.6187	1.0578	0.9687	100	100	Carbohydrate	Glycolysis, gluconeogenesis, pyruvate metabolism	65041	207300-70-7;	1888	217
614		Nucleotide sugars, pentose metabolism	arabitol	GC/MS	38075	C00474	HMDB01851	1.55	0.0113	0.0760	0.7493	1.1617	78	100	Carbohydrate	Nucleotide sugars, pentose metabolism	94154	7643-75-6;	1687.5	307.1
616			ribitol	GC/MS	15772	C00474	HMDB00508	1.23	0.1548	0.3197	0.9335	1.1492	89	100	Carbohydrate	Nucleotide sugars, pentose metabolism		488-81-3;	1692.4	217
619			sedoheptulose-7-phosphate	GC/MS	35649	C05382	HMDB01068	1.17	0.1627	0.3270	0.9472	1.1068	100	100	Carbohydrate	Nucleotide sugars, pentose metabolism	616	2646-35-7;	2070	691.35
622			ribose	GC/MS	12080	C00121	HMDB00283	1.45	0.0787	0.2144	0.9995	1.4503	100	100	Carbohydrate	Nucleotide sugars, pentose metabolism	5311110	50-69-1;	1639.2	204
627			ribulose	GC/MS	35855	C00309	HMDB00621	1.11	0.5250	0.5845	0.8701	0.9645	100	100	Carbohydrate	Nucleotide sugars, pentose metabolism	79021	488-84-6;	1662	306.1
629			Isobar: ribulose 5-phosphate, xylulose 5-phosphate	GC/MS	37288			0.96	0.6665	0.6484	1.2680	1.2167	100	100	Carbohydrate	Nucleotide sugars, pentose metabolism			1935	357.2
633			xylitol	GC/MS	4966	C00379	HMDB02917	0.84	0.2023	0.3647	1.0269	0.8644	100	100	Carbohydrate	Nucleotide sugars, pentose metabolism	6912	87-99-0;	1677.6	307.2
639			xylulose	GC/MS	18344	C00310	HMDB00654	1.06	0.5244	0.5845	0.9896	1.0472	89	100	Carbohydrate	Nucleotide sugars, pentose metabolism	5289590	551-84-8;	1668	306.1
648		Krebs cycle	citrate	GC/MS	1564	C00158	HMDB00094	0.49	0.0007	0.0124	1.7883	0.8795	100	100	Energy	Krebs cycle	311	77-92-9;	1763.4	273.1
649			2-methylcitrate	GC/MS	37483	C02225	HMDB00379	0.37	0.0000	0.0008	1.6467	0.6061	100	91	Energy	Krebs cycle	5460420	6061-96-7;	1778	287.1
651			cis-aconitate	LC/MS neg	12025	C00417	HMDB00072	0.67	0.0023	0.0275	1.2541	0.8393	100	100	Energy	Krebs cycle	643757	585-84-2;	603	173.1
659			succinate	GC/MS	1437	C00042	HMDB00254	0.87	0.3438	0.4775	1.2402	1.0806	100	100	Energy	Krebs cycle	1110	110-15-6;	1348	247
663	Energy		fumarate	GC/MS	1643	C00122	HMDB00134	0.79	0.0572	0.1854	1.1675	0.9224	100	100	Energy	Krebs cycle		100-17-8;	1382.1	245
667			malate	GC/MS	1303	C00149	HMDB00156	0.86	0.0402	0.1490	1.1147	0.9576	100	100	Energy	Krebs cycle	525	6915-15-7;	1502	233
670		Oxidative phosphorylation	acetylphosphate	GC/MS	15488	C00227	HMDB01494	0.97	0.8119	0.6977	1.0555	1.0220	100	100	Energy	Oxidative phosphorylation	186	94249-01-1;	1263	211
671			phosphate	GC/MS	11438	C00009	HMDB01429	1.03	0.6932	0.6542	0.9902	1.0153	100	100	Energy	Oxidative phosphorylation	1061	7664-38-2 ;	1307.7	298.9
672			pyrophosphate (PPi)	GC/MS	2078	C00013	HMDB00250	0.91	0.9797	0.7469	0.9996	0.9076	100	100	Energy	Oxidative phosphorylation	644102	1466-09-3;2466-09-3;	1642.3	451
673		Essential fatty acid	linoleate (18:2n6)	LC/MS neg	1105	C01595	HMDB00673	1.04	0.8836	0.7286	1.0608	1.1056	100	100	Lipid	Essential fatty acid	5280450	60-33-3;	5533	279.3
677			linolenate [alpha or gamma; (18:3n3 or 6)]	LC/MS neg	34035	C06427	HMDB01388	1.15	0.8947	0.7319	1.2157	1.3937	100	100	Lipid	Essential fatty acid			5450	277.3
678			dihomo-linolenate (20:3n3 or n6)	LC/MS neg	35718	C03242	HMDB02925	0.76	0.1104	0.2554	1.2303	0.9384	100	100	Lipid	Essential fatty acid	5312529	17046-59-2 ;	5600	305.4
679			eicosapentaenoate (EPA; 20:5n3)	LC/MS neg	18467	C06428	HMDB01999	0.80	0.4485	0.5366	1.1683	0.9369	100	100	Lipid	Essential fatty acid	446284	10-2005-9;10417-94-4;	5442	301.3
680			docosapentaenoate (n3 DPA; 22:5n3)	LC/MS neg	32504	C16513	HMDB01976	0.68	0.0862	0.2246	1.4101	0.9533	100	100	Lipid	Essential fatty acid		2234-74-4 ;	5574	329.4
681			docosapentaenoate (n6 DPA; 22:5n6)	LC/MS neg	37478	C16513	HMDB13123	0.65	0.0632	0.1915	1.2759	0.8349	100	100	Lipid	Essential fatty acid	6441454	25182-74-5;	5625	329.4
682			docosahexaenoate (DHA; 22:6n3)	LC/MS neg	19323	C06429	HMDB02183	0.62	0.0141	0.0844	1.3874	0.8550	100	100	Lipid	Essential fatty acid	445580	6217-54-5;	5518	327.3
684		Short chain fatty acid	valerate	LC/MS neg	33443	C00803	HMDB00892	0.50	0.0031	0.0346	1.4306	0.7159	100	100	Lipid	Short chain fatty acid	7991	109-52-4;	1774	101.2
687		Medium chain fatty acid	caproate (6:0)	LC/MS neg	32489	C01585	HMDB00535	0.93	0.4660	0.5473	1.8109	1.6868	100	100	Lipid	Medium chain fatty acid	8892	142-62-1;	2766	115.2
688			heptanoate (7:0)	LC/MS neg	1644	C17714	HMDB00666	1.23	0.1537	0.3197	0.9145	1.1288	100	100	Lipid	Medium chain fatty acid	8094	111-14-8;	3695	129.2
689			caprylate (8:0)	LC/MS neg	32492	C06423	HMDB00482	1.03	0.6445	0.6421	0.9854	1.0155	100	100	Lipid	Medium chain fatty acid	379	124-07-2;	4367	143.2
690			pelargonate (9:0)	LC/MS neg	12035	C01601	HMDB00847	1.00	0.9516	0.7469	1.0024	1.0058	100	100	Lipid	Medium chain fatty acid	5461016	112-05-0;	4847	157.2
691			caprate (10:0)	LC/MS neg	1642	C01571	HMDB00511	1.13	0.7526	0.6834	1.1375	1.2864	100	100	Lipid	Medium chain fatty acid	2969	112-37-8;	5092	171.2
694			laurate (12:0)	LC/MS neg	1645	C02679	HMDB00638	1.15	0.4997	0.5737	1.0539	1.2159	100	100	Lipid	Medium chain fatty acid	3893	143-07-7;	5288	199.3
698		Long chain fatty acid	myristate (14:0)	LC/MS neg	1365	C06424	HMDB00806	0.97	0.6391	0.6392	1.1274	1.0913	100	100	Lipid	Long chain fatty acid	11005	544-63-8;	5439	227.3
699			myristoleate (14:1n5)	LC/MS neg	32418	C08322	HMDB02000	1.01	0.7581	0.6834	1.0757	1.0890	100	100	Lipid	Long chain fatty acid	5281119	544-64-9 ;	5338	225.3
700			pentadecanoate (15:0)	GC/MS	1361	C16537	HMDB00826	0.65	0.0154	0.0878	1.4243	0.9211	100	100	Lipid	Long chain fatty acid	13849	1002-84-2;10002-84-2;	1853.5	299.2
701			palmitate (16:0)	LC/MS neg	1336	C00249	HMDB00220	0.95	0.6138	0.6187	1.0375	0.9874	100	100	Lipid	Long chain fatty acid	985	57-10-3;	5619	255.3
702			palmitoleate (16:1n7)	LC/MS neg	33447	C08362	HMDB03229	1.08	0.9362	0.7444	1.0625	1.1465	100	100	Lipid	Long chain fatty acid	445638	373-49-9;	5477	253.3
704			margarate (17:0)	LC/MS neg	1121		HMDB02259	1.00	0.9778	0.7469	1.0824	1.0809	100	100	Lipid	Long chain fatty acid	10465	506-12-7;	5733	269.3
705			10-heptadecenoate (17:1n7)	LC/MS neg	33971			0.90	0.5493	0.5984	1.1451	1.0325	100	100	Lipid	Long chain fatty acid	5312435	29743-97-3;	5558	267.3
706			stearate (18:0)	LC/MS neg	1358	C01530	HMDB00827	1.08	0.7199	0.6702	1.0446	1.1242	100	100	Lipid	Long chain fatty acid	5281	57-11-4;	5886	283.4
708			oleate (18:1n9)	GC/MS	1359	C00712	HMDB00207	1.08	0.9802	0.7469	1.1372	1.2298	100	100	Lipid	Long chain fatty acid	445639	112-80-1;	1984.4	339.2
710			cis-vaccenate (18:1n7)	GC/MS	33970	C08367		1.01	0.9151	0.7373	1.0813	1.0911	100	100	Lipid	Long chain fatty acid	5282761	506-17-2;	1987	339.3
716			nonadecanoate (19:0)	LC/MS neg	1356	C16535	HMDB00772	1.12	0.6094	0.6187	1.1326	1.2672	100	100	Lipid	Long chain fatty acid	12591	646-30-0;	6073	297.3
717			10-nonadecenoate (19:1n9)	LC/MS neg	33972			0.92	0.5882	0.6139	1.2911	1.1828	100	100	Lipid	Long chain fatty acid	5312513	73033-09-7;	5775	295.4
719			arachidate (20:0)	GC/MS	1118	C06425	HMDB02212	1.02	0.8771	0.7267	0.9927	1.0124	100	100	Lipid	Long chain fatty acid	10467	506-30-9;	2071.2	369.3
722			eicosenoate (20:1n9 or 11)	LC/MS neg	33587		HMDB02231	1.06	0.8763	0.7267	1.1726	1.2386	100	100	Lipid	Long chain fatty acid			5955	309.4
724			dihomo-linoleate (20:2n6)	LC/MS neg	17805	C16525		0.87	0.2957	0.4480	1.1799	1.0275	100	100	Lipid	Long chain fatty acid	6439848	2091-39-6;	5722	307.3
728			arachidonate (20:4n6)	LC/MS neg	1110	C00219	HMDB01043	0.66	0.0305	0.1311	1.2654	0.8352	100	100	Lipid	Long chain fatty acid	444899	506-32-1;	5525	303.4
732			erucate (22:1n9)	LC/MS neg	1552	C08316	HMDB02068	1.09	0.3789	0.5050	0.9594	1.0467	100	100	Lipid	Long chain fatty acid	8216	112-86-7;	6273.3	337.4
735			adrenate (22:4n6)	LC/MS neg	32980	C16527	HMDB02226	0.73	0.1310	0.2844	1.4003	1.0266	100	100	Lipid	Long chain fatty acid	5282844	2091-25-0;	5684	331.3
762			4-hydroxybutyrate (GHB)	GC/MS	34585	C00989	HMDB00710	0.60	0.0333	0.1354	1.5273	0.9118	100	100	Lipid	Fatty acid, monohydroxy	10413	502-85-2;	1277	233.1
768			3-hydroxydecanoate	LC/MS neg	22053		HMDB02203	1.08	0.2802	0.4400	0.9745	1.0536	100	100	Lipid	Fatty acid, monohydroxy	26612	5561-87-5;	4681	187.2

774		Fatty acid, monohydroxy	2-hydroxystearate	LC/MS neg	17945	C03045		0.85	0.0778	0.2142	1.0803	0.9218	100	100	Lipid	Fatty acid, monohydroxy	69417	629-22-1;	5705	299.4
776			2-hydroxypalmitate	LC/MS neg	35675			0.97	0.8128	0.6977	1.1333	1.0995	100	100	Lipid	Fatty acid, monohydroxy	92836	764-67-0;	5508	271.3
780			13-HODE + 9-HODE	LC/MS neg	37752			1.51	0.3970	0.5108	1.1801	1.7793	100	100	Lipid	Fatty acid, monohydroxy			5270	295.2
782		Fatty acid, dihydroxy	12,13-hydroxyoctadec-9(Z)-enoate	LC/MS neg	38395	C14829	HMDB04705	0.69	0.0401	0.1490	1.2833	0.8828	100	100	Lipid	Fatty acid, dihydroxy	10236635	263399-35-5;	5137	313.4
786			2-hydroxyglutarate	GC/MS	37253	C02630	HMDB00606	0.94	0.3369	0.4736	1.0612	0.9940	100	100	Lipid	Fatty acid, dicarboxylate	43	40951-21-1;	1576	247
793			sebacate (decanedioate)	LC/MS neg	32398	C08277	HMDB00792	0.44	0.0014	0.0184	1.3287	0.5815	100	100	Lipid	Fatty acid, dicarboxylate	5192	111-20-6;	1778	201.2
794			azelate (nonanedioate)	LC/MS neg	18362	C08261	HMDB00784	0.42	0.0009	0.0128	1.7282	0.7244	100	100	Lipid	Fatty acid, dicarboxylate	2266	123-99-9;	1322	187.2
796		Fatty acid, dicarboxylate	dodecanedioate	LC/MS neg	32388	C02678	HMDB00623	0.30	0.0001	0.0045	1.8640	0.5499	100	100	Lipid	Fatty acid, dicarboxylate	12736	693-23-2 ;	2990	229.2
798			hexadecanedioate	LC/MS neg	35678		HMDB00672	0.55	0.0053	0.0494	1.4620	0.8015	89	91	Lipid	Fatty acid, dicarboxylate	10459	505-54-4;	4650	285.2
799			octadecanedioate	LC/MS neg	36754		HMDB00782	0.24	0.0001	0.0045	2.7751	0.6735	100	100	Lipid	Fatty acid, dicarboxylate	70095	871-70-5;	5033	313.3
800			undecanedioate	LC/MS neg	35671		HMDB00888	0.42	0.0003	0.0079	1.4663	0.6219	100	100	Lipid	Fatty acid, dicarboxylate	15816	1852-04-6;	2376	215.1
819		Fatty acid, branched	15-methylpalmitate (isobar with 2-methylpalmitate)	LC/MS neg	38768			0.92	0.4047	0.5129	1.1711	1.0809	100	100	Lipid	Fatty acid, branched			5698	269.4
821			17-methylstearate	LC/MS neg	38296			1.03	0.6127	0.6187	1.1667	1.2002	100	100	Lipid	Fatty acid, branched	3083779	2724-59-6;	5987	297.4
830			prostaglandin A2	LC/MS neg	19761	C05953	HMDB02752	1.48	0.0344	0.1375	0.8451	1.2493	100	100	Lipid	Eicosanoid	5280880	13345-50-1;	4875	333.3
831			prostaglandin B2	LC/MS neg	19499	C05954	HMDB04236	1.02	0.9646	0.7469	1.0324	1.0483	78	82	Lipid	Eicosanoid	5280881	13367-85-6;	4943	333.4
833			prostaglandin E1	LC/MS neg	19391	C04741	HMDB01442	1.05	0.6822	0.6517	0.9443	0.9897	100	91	Lipid	Eicosanoid	5280723	745-65-3;	4600	353.3
834			prostaglandin E2	LC/MS neg	7746	C00584	HMDB01220	1.26	0.1311	0.2844	0.9638	1.2118	100	100	Lipid	Eicosanoid	5280360	363-24-6;	4562	351.3
846		Eicosanoid	13,14-dihydro-15-keto-prostaglandin a2	LC/MS neg	19449		HMDB01244	1.57	0.1136	0.2596	0.7902	1.2424	67	100	Lipid	Eicosanoid		74872-89-2;	4822	333.4
865			6-keto prostaglandin F1alpha	LC/MS neg	20476	C05961	HMDB02886	1.06	0.7560	0.6834	1.0090	1.0707	100	100	Lipid	Eicosanoid	5280888	58961-34-8;58962-34-8;	4180	369.3
873			12-HETE	LC/MS neg	37536		HMDB06111	1.55	0.6653	0.6484	1.2518	1.9432	100	100	Lipid	Eicosanoid		73837-14-6;	5295	319.3
874			15-HETE	LC/MS neg	37538	C04742	HMDB02110	1.12	0.9953	0.7479	1.0007	1.1211	100	100	Lipid	Eicosanoid	5280724	54845-95-3;	5271	319.3
890		Fatty acid metabolism (also BCAA metabolism)	butyrylcarnitine	LC/MS pos	32412			1.05	0.5145	0.5812	1.0817	1.1323	100	100	Lipid	Fatty acid metabolism (also BCAA metabolism)	213144	25576-40-3;	2007	232.2
892		Fatty acid metabolism	valerylcarnitine	LC/MS pos	34406		HMDB13128	1.16	0.2084	0.3702	0.9591	1.1101	100	100	Lipid	Fatty acid metabolism	6426903		2584	246.2
894			isovalerate	LC/MS neg	34732	C08262	HMDB00718	0.31	0.0003	0.0079	1.5931	0.4932	100	100	Lipid	Fatty acid metabolism	10430	503-74-2;	1623	101.2
898			deoxycarnitine	LC/MS pos	36747	C01181	HMDB01161	1.18	0.0149	0.0865	0.9079	1.0741	100	100	Lipid	Carnitine metabolism	725	6249-56-5;	759	146.1
899			carnitine	LC/MS pos	15500	C00318		0.95	0.3223	0.4720	1.0214	0.9691	100	100	Lipid	Carnitine metabolism	10917	461-05-2;	702	162.2
900		Carnitine metabolism	3-dehydrocarnitine*	LC/MS pos	32654	C02636	HMDB12154	1.32	0.3176	0.4720	1.0207	1.3479	100	100	Lipid	Carnitine metabolism	6991982	10457-99-5;	1020	160.2
901			acetylcarnitine	LC/MS pos	32198	C02571	HMDB00201	1.29	0.0597	0.1909	0.8290	1.0699	100	100	Lipid	Carnitine metabolism	7045767	5080-50-2;	1203	204.2
902			hexanoylcarnitine	LC/MS pos	32328		HMDB00705	1.46	0.0164	0.0911	0.8401	1.2246	100	100	Lipid	Carnitine metabolism	6426853	6920-35-0;	3174	260.2
913			stearoylcarnitine	LC/MS pos	34409		HMDB00848	1.47	0.2656	0.4250	0.6083	0.8964	67	91	Lipid	Carnitine metabolism		18822-91-8;	5300	428.4
919.1			glycohyocholate	LC/MS pos	42574			0.63	0.3252	0.4720	1.8265	1.1556	89	73	Lipid	Bile acid metabolism			5115	466.2
920.1			taurohyocholate	LC/MS neg	42603			0.56	0.5382	0.5940	4.0719	2.2846	100	91	Lipid	Bile acid metabolism			5000	514.3
921			chenodeoxycholate	LC/MS neg	37789	C02528	HMDB00518	0.49	0.7115	0.6649	3.9488	1.9202	100	100	Lipid	Bile acid metabolism		474-24-9;474-25-9;	5250	391.4
922			taurochenodeoxycholate	LC/MS neg	18494	C05465	HMDB00951	0.67	0.2111	0.3702	2.0221	1.3506	89	64	Lipid	Bile acid metabolism	387316	6009-98-9;	5218	498.3
924		Bile acid metabolism	hyodeoxycholate	LC/MS neg	27531	C15517	HMDB00733	1.06	0.7440	0.6826	1.1101	1.1723	100	100	Lipid	Bile acid metabolism	5283820	83-49-8;	5074	391.4
935			lithocholate	LC/MS neg	1483	C03990	HMDB00761	0.76	0.1107	0.2554	1.1749	0.8896	100	100	Lipid	Bile acid metabolism	9903	434-13-9;	5350	375.4
939			6-oxolithocholate	LC/MS neg	31902			0.88	0.6773	0.6517	1.1532	1.0149	100	100	Lipid	Bile acid metabolism	164960	2393-61-5 ;	5040	389.4
941			6-beta-hydroxylithocholate	LC/MS neg	36807	C15515	HMDB00811	0.73	0.1872	0.3524	1.4251	1.0336	100	100	Lipid	Bile acid metabolism	5283821	668-49-5;	4826	391.3
944			hyocholate	LC/MS neg	34093	C17649	HMDB00760	0.76	0.4553	0.5412	3.6843	2.8059	100	100	Lipid	Bile acid metabolism		547-75-1;	5001	407.3
947	Lipid		alpha-muricholate	LC/MS neg	34092	C17647	HMDB00506	1.85	0.0420	0.1513	0.7084	1.3117	89	91	Lipid	Bile acid metabolism	3083761	2393-58-0;	4763	407.4
953		Fatty alcohol, long chain	1-octadecanol	GC/MS	21128	D01924	HMDB02350	1.04	0.7787	0.6875	0.9940	1.0299	100	100	Lipid	Fatty alcohol, long chain	8221	112-92-5;	1957.7	327.3
954			choline phosphate	LC/MS pos	34396	C00588		0.93	0.3242	0.4720	1.0306	0.9617	100	100	Lipid	Glycerolipid metabolism	135437	72556-74-2;	695	184.1
955			ethanolamine	GC/MS	1497	C00189	HMDB00149	0.99	0.9014	0.7350	1.1505	1.1413	100	100	Lipid	Glycerolipid metabolism		141-43-5;	1304	174.1
956			phosphoethanolamine	LC/MS neg	1600	C00346	HMDB00224	0.99	0.7704	0.6872	1.0148	1.0023	100	100	Lipid	Glycerolipid metabolism	5232324;1015	1071-23-4;	644	140.1
957			glycerophosphoethanolamine	GC/MS	37455	C01233	HMDB00114	1.62	0.0908	0.2277	0.8557	1.3857	89	100	Lipid	Glycerolipid metabolism	123874	33049-08-0;	1906	357.1
959		Glycerolipid metabolism	glycerol	GC/MS	15122	C00116	HMDB00131	0.98	0.7921	0.6922	1.0569	1.0309	100	100	Lipid	Glycerolipid metabolism	753	56-81-5;	1311	205
960			choline	LC/MS pos	15506	C00114		0.98	0.6667	0.6484	1.0411	1.0247	100	100	Lipid	Glycerolipid metabolism	170746	67-48-1;	674	104.2
961			glycerol 3-phosphate (G3P)	GC/MS	15365	C00093	HMDB00126	1.35	0.2689	0.4276	1.0489	1.4184	100	100	Lipid	Glycerolipid metabolism	754	29849-82-9;	1719.7	357.1
962			glycerophosphorylcholine (GPC)	LC/MS pos	15990	C00670	HMDB00086	1.49	0.1590	0.3258	0.9277	1.3784	100	100	Lipid	Glycerolipid metabolism	657272	28319-77-9;	694	258.1
965			cytidine 5'-diphosphocholine	LC/MS pos	34418	C00307		1.60	0.0280	0.1264	0.7770	1.2439	100	100	Lipid	Glycerolipid metabolism		33818-15-4;	777	489.1
971			myo-inositol	GC/MS	19934	C00137	HMDB00211	0.99	0.8610	0.7207	1.1023	1.0908	100	100	Lipid	Inositol metabolism		87-89-8;	1924.9	217
972			chiro-inositol	GC/MS	37112			0.91	0.8104	0.6977	1.1573	1.0541	100	100	Lipid	Inositol metabolism		643-12-9;	1857	318.1
973		Inositol metabolism	pinitol	GC/MS	37086	C03844		1.42	0.0679	0.1953	0.7953	1.1302	67	100	Lipid	Inositol metabolism		10284-63-6;	1773	318.1
974			inositol 1-phosphate (I1P)	GC/MS	1481	C04006	HMDB00213	0.84	0.1194	0.2682	1.1338	0.9500	100	100	Lipid	Inositol metabolism		106032-59-1;	2057.8	318.1
979			scyllo-inositol	GC/MS	32379	C06153	HMDB06088	1.00	0.7629	0.6850	1.2035	1.1980	100	100	Lipid	Inositol metabolism		488-59-5;	1893.8	318.2
984		Ketone bodies	3-hydroxybutyrate (BHBA)	GC/MS	542	C01089	HMDB00357	0.63	0.0136	0.0844	1.3239	0.8399	100	100	Lipid	Ketone bodies	441	625-72-9;	1203.5	116.9
986			1,2-propanediol	GC/MS	38002	C00583	HMDB01881	0.51	0.0904	0.2277	1.7654	0.9063	100	100	Lipid	Ketone bodies		57-55-6;	1041	117
990			1-palmitoylglycerophosphoethanolamine	LC/MS neg	35631		HMDB11503	0.68	0.4905	0.5683	1.5555	1.0508	100	100	Lipid	Lysolipid	9547069		5940	452.3
991			2-palmitoylglycerophosphoethanolamine*	LC/MS neg	35688			1.06	0.6914	0.6542	1.0239	1.0831	100	100	Lipid	Lysolipid			5790	452.3
995			1-stearoylglycerophosphoethanolamine	LC/MS neg	34416		HMDB11130	0.63	0.2080	0.3702	1.5796	1.0006	100	100	Lipid	Lysolipid	9547068	69747-55-3;	6200	480.4
995.1			2-stearoylglycerophosphoethanolamine*	LC/MS pos	41220			0.75	0.8317	0.7075	1.0783	0.8124	67	82	Lipid	Lysolipid			5779.6	482.2
996			1-oleoylglycerophosphoethanolamine	LC/MS neg	35628		HMDB11506	0.91	0.9338	0.7444	1.2999	1.1892	100	100	Lipid	Lysolipid	9547071	89576-29-4;	5928	478.3
997			2-oleoylglycerophosphoethanolamine*	LC/MS neg	35687			1.31	0.2395	0.4034	0.9055	1.1824	100	100	Lipid	Lysolipid			5848	478.3
999			2-linoleoylglycerophosphoethanolamine*	LC/MS neg	36593			1.25	0.3728	0.4997	0.9310	1.1610	100	100	Lipid	Lysolipid			5650	476.4
1000			1-arachidonoylglycerophosphoethanolamine*	LC/MS neg	35186		HMDB11517	1.25	0.1771	0.3385	0.9598	1.1975	100	100	Lipid	Lysolipid			5731	500.3
1001			2-arachidonoylglycerophosphoethanolamine*	LC/MS neg	32815			1.01	0.9157	0.7373	0.9558	0.9686	100	100	Lipid	Lysolipid			5674	500.3
1003			2-docosapentaenoylglycerophosphoethanolamine*	LC/MS pos	34875			1.55	0.4383	0.5366	0.5836	0.9031	56	82	Lipid	Lysolipid			5548	528.2
1006			1-stearoylglycerophosphoglycerol	LC/MS neg	34437			0.73	0.2312	0.3999	1.5857	1.1612	100	100	Lipid	Lysolipid			5826	511.4
1006.1			2-stearoylglycerophosphoglycerol*	LC/MS neg	41828			0.71	0.0636	0.1915	1.3310	0.9439	100	100	Lipid	Lysolipid			5758.4	511.4
1007			1-myristoylglycerophosphocholine	LC/MS pos	35626		HMDB10379	1.02	0.6762	0.6517	0.7858	0.8021	56	73	Lipid	Lysolipid	460604	20559-16-4;	5481	468.3
1009			1-pentadecanoylglycerophosphocholine*	LC/MS pos	37418			0.87	0.6685	0.6484	1.0030	0.8707	56	64	Lipid	Lysolipid			5563	482.3
1010			1-palmitoylglycerophosphocholine	LC/MS pos	33955			0.79	0.4272	0.5258	1.3848	1.0926	89	100	Lipid	Lysolipid	86554	17364-16-8;	5671	496.4
1011		Lysolipid	2-palmitoylglycerophosphocholine*	LC/MS pos	35253			1.63	0.3305	0.4720	0.5195	0.8493	44	91	Lipid	Lysolipid			5604	496.3
1012			1-palmitoleoylglycerophosphocholine*	LC/MS pos	33230			1.10	0.4761	0.5542	1.1586	1.2742	89	100	Lipid	Lysolipid			5524	494.3
1014			1-heptadecanoylglycerophosphocholine	LC/MS pos	33957		HMDB12108	0.66	0.7233	0.6710	1.1227	0.7397	56	82	Lipid	Lysolipid		50930-23-9;	5750	510.4
1015			1-stearoylglycerophosphocholine	LC/MS pos	33961			0.57	0.5843	0.6132	1.7747	1.0179	67	100	Lipid	Lysolipid	497299	19420-57-6;	5844	524.4
1016			2-stearoylglycerophosphocholine*	LC/MS pos	35255			0.54	0.1759	0.3385	1.1246	0.6078	56	45	Lipid	Lysolipid	10208382		5780	524.4
1017			1-oleoylglycerophosphocholine	LC/MS pos	33960			0.76	0.9911	0.7479	1.1283	0.8520	56	91	Lipid	Lysolipid	16081932	19420-56-5;	5700	522.4
1019			1-linoleoylglycerophosphocholine	LC/MS pos	34419	C04100		0.97	0.7869	0.6900	1.1229	1.0878	56	91	Lipid	Lysolipid	11988421		5574	520.4
1020			2-linoleoylglycerophosphocholine*	LC/MS pos	35257			1.96	0.2603	0.4250	0.6255	1.2261	56	91	Lipid	Lysolipid			5544	520.4
1022			1-eicosadienoylglycerophosphocholine*	LC/MS pos	33871			0.51	0.2852	0.4400	1.1152	0.5716	56	45	Lipid	Lysolipid			5696	548.3
1026			2-arachidonoylglycerophosphocholine*	LC/MS pos	35256			1.42	0.4566	0.5412	0.7531	1.0676	56	82	Lipid	Lysolipid			5524	544.3
1035			1-stearoylglycerophosphoinositol	LC/MS neg	19324			0.69	0.4262	0.5258	1.4984	1.0276	100	100	Lipid	Lysolipid		796963-93-4;	5800	599.4
1036			2-stearoylglycerophosphoinositol*	LC/MS neg	39223			0.64	0.3121	0.4674	1.5505	0.9873	100	100	Lipid	Lysolipid			5751.6	599.4
1040			1-arachidonoylglycerophosphoinositol*	LC/MS neg	34214			1.13	0.2278	0.3968	0.9148	1.0373	100	100	Lipid	Lysolipid			5479	619.4
1043			1-oleoylglycerophosphoserine	LC/MS neg	19260			0.66	0.0472	0.1632	1.4452	0.9516	100	100	Lipid	Lysolipid	9547099		5690	522.3
1044			2-oleoylglycerophosphoserine*	LC/MS neg	37948			1.53	0.0643	0.1915	0.8970	1.3684	100	100	Lipid	Lysolipid			5577	522.3
1045			1-palmitoylplasmenylethanolamine*	LC/MS neg	39270			0.56	0.0550	0.1830	1.6075	0.8970	100	100	Lipid	Lysolipid			6153	436.4
1046			1-myristoylglycerol (1-monomyristin)	LC/MS neg	35625		HMDB11561	1.69	0.6863	0.6533	1.1485	1.9376	100	100	Lipid	Monoacylglycerol	79050	75685-84-6;	5965	227.3
1047			2-myristoylglycerol (2-monomyristin)	LC/MS neg	34383			1.45	0.6032	0.6177	1.1239	1.6351	100	100	Lipid	Monoacylglycerol	137938	27214-38-6;	5798	227.3
1049			1-pentadecanoylglycerol (1-monopentadecanoin)	GC/MS	34385			1.12	0.9772	0.7469	0.8640	0.9703	33	27	Lipid	Monoacylglycerol	190750		2085	357.3
1050			1-heptadecanoylglycerol (1-monoheptadecanoin)	GC/MS	34391			1.37	0.9078	0.7356	1.0898	1.4878	78	82	Lipid	Monoacylglycerol	107036	68002-72-2;	2156	385.2
1051			1-palmitoylglycerol (1-monopalmitin)	GC/MS	21127			1.57	0.8877	0.7286	1.5048	2.3604	100	100	Lipid	Monoacylglycerol	14900	542-44-9;	2119.5	371.3
1052			2-palmitoylglycerol (2-monopalmitin)	GC/MS	33419			1.65	0.5433	0.5946	1.0887	1.7928	100	100	Lipid	Monoacylglycerol	123409	23470-00-0;	2108	129
1053		Monoacylglycerol	1-stearoylglycerol (1-monostearin)	GC/MS	21188	D01947		0.92	0.5516	0.5984	1.2417	1.1424	100	100	Lipid	Monoacylglycerol	24699	123-94-4;31566-31-1;	2186.6	399.4
1054			2-stearoylglycerol (2-monostearin)	GC/MS	34059			1.30	0.5128	0.5812	0.4851	0.6321	22	36	Lipid	Monoacylglycerol	79075	621-61-4;	2178	341.3
1055			1-oleoylglycerol (1-monoolein)	GC/MS	21184		HMDB11567	1.43	0.8878	0.7286	1.1901	1.7008	100	100	Lipid	Monoacylglycerol	5283468	111-03-5;	2181.2	397.4
1057			1-linoleoylglycerol (1-monolinolein)	LC/MS neg	27447			0.82	0.9487	0.7469	1.5959	1.3088	100	100	Lipid	Monoacylglycerol	5283469	2277-28-3;	6226	279.2
1058			2-linoleoylglycerol (2-monolinolein)	LC/MS neg	32506		HMDB11538	0.87	0.8513	0.7194	1.7696	1.5332	100	100	Lipid	Monoacylglycerol	5365676	3443-82-1;	5988	279.3
1060			1-arachidonylglycerol	LC/MS neg	34397	C13857	HMDB11572	0.77	0.1305	0.2844	1.2548	0.9669	100	100	Lipid	Monoacylglycerol	5282281	35474-99-8;	5937	303.4
1061			2-arachidonoyl glycerol	LC/MS neg	19266	C13856	HMDB04666	0.86	0.4129	0.5176	1.1902	1.0245	100	100	Lipid	Monoacylglycerol	5282280	53847-30-6;	5955	303.3
1074			sphinganine	LC/MS pos	17769	C00836	HMDB00269	1.73	0.3379	0.4736	0.6089	1.0560	56	82	Lipid	Sphingolipid	3126	3102-56-5;	5175	302.3
1077			sphingosine	LC/MS pos	17747	C00319	HMDB00252	2.13	0.1757	0.3385	0.5529	1.1766	56	100	Lipid	Sphingolipid	5353955	123-78-4;	5197	300.2
1081		Sphingolipid	N,N-dimethylsphingosine	LC/MS pos	32460	C13914		1.91	0.1252	0.2787	0.7765	1.4817	56	91	Lipid	Sphingolipid	5282309	119567-63-4;	5192	328.3
1086			palmitoyl sphingomyelin	GC/MS	37506			0.93	0.3318	0.4720	1.0692	0.9980	100	100	Lipid	Sphingolipid	9939941	6254-89-3;	2524	311.3
1087			stearoyl sphingomyelin	GC/MS	19503	C00550	HMDB01348	0.76	0.0195	0.1000	1.1523	0.8799	100	100	Lipid	Sphingolipid	6453725	85187-10-6;85187-10-6 ;	2645	311.3
1100			lathosterol	GC/MS	39864	C01189	HMDB01170	1.18	0.2331	0.3999	0.9220	1.0906	78	100	Lipid	Sterol/Steroid	65728	80-99-9 ;	2337	255.2
1101			squalene	GC/MS	1518	C00751	HMDB00256	0.77	0.1914	0.3551	1.2377	0.9567	100	100	Lipid	Sterol/Steroid	638072	111-02-4;	2205	69
1102			cholesterol	GC/MS	63	C00187	HMDB00067	0.93	0.3077	0.4635	1.0581	0.9860	100	100	Lipid	Sterol/Steroid	6432564	57-88-5;	2316.9	329.3
1105		Sterol/Steroid	7-alpha-hydroxycholesterol	GC/MS	35692	C03594	HMDB01496	1.68	0.3385	0.4736	0.4410	0.7400	33	55	Lipid	Sterol/Steroid	107722	566-27-8;	2300	456.2
1106			7-beta-hydroxycholesterol	GC/MS	35092	C03594	HMDB06119	1.08	0.9656	0.7469	0.9338	1.0055	100	91	Lipid	Sterol/Steroid	473141	566-27-8;	2340	456.4
1139			beta-sitosterol	GC/MS	27414	C01753	HMDB00852	0.63	0.1984	0.3628	1.2694	0.7970	89	55	Lipid	Sterol/Steroid	222284	83-46-5;	2380.8	357.4
1141			campesterol	GC/MS	39511	C01789	HMDB02869	1.32	0.1850	0.3510	0.8808	1.1631	89	91	Lipid	Sterol/Steroid	173183	474-62-4;	2353	343.4
1157			7-alpha-hydroxy-3-oxo-4-cholestenoate (7-Hoca)	LC/MS neg	36776	C17337	HMDB12458	0.36	0.0883	0.2261	2.6542	0.9505	100	100	Lipid	Sterol/Steroid	3081085	115538-85-7;	5268	429.4
1187			xanthine	LC/MS pos	3147	C00385	HMDB00292	1.08	0.4939	0.5696	0.9291	1.0068	100	100	Nucleotide	Purine metabolism, (hypo)xanthine/inosine containing	1188	69-89-6;	1430	153.1
1188			xanthosine	LC/MS neg	15136	C01762	HMDB00299	0.95	0.5121	0.5812	1.0647	1.0129	100	100	Nucleotide	Purine metabolism, (hypo)xanthine/inosine containing	64959	146-80-5;	1075	283.1
1190		Purine metabolism, (hypo)xanthine/inosine containing	hypoxanthine	LC/MS neg	3127	C00262	HMDB00157	1.03	0.5792	0.6132	0.9869	1.0151	100	100	Nucleotide	Purine metabolism, (hypo)xanthine/inosine containing	790	68-94-0;	1313	135.1
1192			inosine	LC/MS neg	1123	C00294	HMDB00195	0.82	0.1061	0.2515	1.0703	0.8732	100	100	Nucleotide	Purine metabolism, (hypo)xanthine/inosine containing		58-63-9;	1600	267.2
1193			2'-deoxyinosine	LC/MS neg	15076	C05512	HMDB00071	0.44	0.0004	0.0110	1.8719	0.8174	100	100	Nucleotide	Purine metabolism, (hypo)xanthine/inosine containing	65058	890-38-0;	1700	251.1
1198			adenine	GC/MS	554	C00147	HMDB00034	1.75	0.0082	0.0617	0.7386	1.2943	100	100	Nucleotide	Purine metabolism, adenine containing	190	73-24-5;	1804.2	264
1199			adenosine	LC/MS pos	555	C00212	HMDB00050	1.29	0.0312	0.1311	0.9447	1.2224	100	100	Nucleotide	Purine metabolism, adenine containing	60961	58-61-7;	1650	268.1
1206		Purine metabolism, adenine containing	adenosine 2'-monophosphate (2'-AMP)	LC/MS neg	36815	C00946	HMDB11617	1.26	0.2870	0.4401	0.8998	1.1372	89	100	Nucleotide	Purine metabolism, adenine containing		130-49-4;	1497	346.1
1207			adenosine 3'-monophosphate (3'-AMP)	LC/MS pos	35142	C01367	HMDB03540	2.58	0.0204	0.1023	0.9910	2.5578	100	100	Nucleotide	Purine metabolism, adenine containing	15938966	84-21-9;	1451	348.1
1208			adenosine 5'-monophosphate (AMP)	LC/MS pos	32342	C00020	HMDB00045	0.88	0.8715	0.7267	1.4665	1.2899	100	100	Nucleotide	Purine metabolism, adenine containing	15938965	149022-20-8;	1210	348.1
1220			guanine	LC/MS pos	32352	C00242	HMDB00132	1.13	0.2641	0.4250	0.9831	1.1111	100	100	Nucleotide	Purine metabolism, guanine containing	764	73-40-5;	1022	152.1
1224		Purine metabolism, guanine containing	guanosine	LC/MS pos	1573	C00387	HMDB00133	0.99	0.9891	0.7479	1.0157	1.0020	100	100	Nucleotide	Purine metabolism, guanine containing	6802	118-00-3;	1676	284
1225	Nucleotide		2'-deoxyguanosine	LC/MS neg	1411	C00330	HMDB00085	0.37	0.0003	0.0079	2.2047	0.8241	100	91	Nucleotide	Purine metabolism, guanine containing	187790	961-07-9;	1759	266.2
1247		Purine metabolism, urate metabolism	urate	GC/MS	1604	C00366	HMDB00289	1.13	0.5971	0.6139	0.8435	0.9550	56	82	Nucleotide	Purine metabolism, urate metabolism		69-93-2;120K5305;	1928	441.2
1248			allantoin	GC/MS	1107	C02350	HMDB00462	1.17	0.4465	0.5366	1.0810	1.2691	100	100	Nucleotide	Purine metabolism, urate metabolism	204	97-59-6;	1809.8	518.3
1251			cytidine	LC/MS pos	514	C00475	HMDB00089	0.58	0.0000	0.0021	1.3186	0.7602	100	100	Nucleotide	Pyrimidine metabolism, cytidine containing	6175	65-46-3;	1065	244
1255		Pyrimidine metabolism, cytidine containing	cytidine 5'-monophosphate (5'-CMP)	LC/MS pos	2372	C00055	HMDB00095	0.99	0.8421	0.7139	1.0299	1.0247	100	100	Nucleotide	Pyrimidine metabolism, cytidine containing	7058165	63-37-6;	887	324
1260			2'-deoxycytidine	LC/MS pos	15949	C00881	HMDB00014	0.43	0.0006	0.0123	1.9730	0.8460	100	100	Nucleotide	Pyrimidine metabolism, cytidine containing	13711	951-77-9;	1256	228
1271		Pyrimidine metabolism, thymine containing	thymine	GC/MS	604	C00178	HMDB00262	1.16	0.4715	0.5513	0.9781	1.1345	78	100	Nucleotide	Pyrimidine metabolism, thymine containing	1135	65-71-4;	1429.1	255
1272			thymidine	LC/MS neg	2183	C00214	HMDB00273	0.57	0.0017	0.0219	1.5582	0.8899	100	100	Nucleotide	Pyrimidine metabolism, thymine containing	5789	50-89-5;	1935	241.1
1277			uracil	GC/MS	605	C00106	HMDB00300	1.27	0.0308	0.1311	0.9270	1.1799	100	100	Nucleotide	Pyrimidine metabolism, uracil containing	1174	66-22-8;	1370.4	241
1278			5,6-dihydrouracil	GC/MS	1559	C00429	HMDB00076	1.00	0.8063	0.6977	1.0187	1.0149	100	100	Nucleotide	Pyrimidine metabolism, uracil containing	649	504-07-4;	1505.2	243
1279		Pyrimidine metabolism, uracil containing	uridine	LC/MS neg	606	C00299	HMDB00296	1.02	0.9189	0.7375	0.9990	1.0181	100	100	Nucleotide	Pyrimidine metabolism, uracil containing	6029	58-96-8;	1430	243.1
1280			pseudouridine	LC/MS neg	33442	C02067	HMDB00767	1.30	0.0101	0.0697	0.8371	1.0857	100	100	Nucleotide	Pyrimidine metabolism, uracil containing		1445-07-4;	1104	243.1
1281			2'-deoxyuridine	LC/MS neg	1412	C00526	HMDB00012	0.82	0.3298	0.4720	1.1094	0.9115	100	91	Nucleotide	Pyrimidine metabolism, uracil containing	13712	951-78-0;	1608	227.1
1294		Purine and pyrimidine metabolism	methylphosphate	GC/MS	37070			1.22	0.0142	0.0844	0.8715	1.0661	100	100	Nucleotide	Purine and pyrimidine metabolism	13130	7023-27-0;	1221	240.9
1298		Ascorbate and aldarate metabolism	ascorbate (Vitamin C)	GC/MS	1640	C00072	HMDB00044	1.22	0.1185	0.2682	0.8628	1.0537	100	100	Cofactors and vitamins	Ascorbate and aldarate metabolism		134-03-2;	1850.1	332.1
1299			dehydroascorbate	GC/MS	1659	C05422	HMDB01264	0.88	0.2516	0.4184	0.8942	0.7872	67	36	Cofactors and vitamins	Ascorbate and aldarate metabolism	835	490-83-5;	1800	245.1
1313		Hemoglobin and porphyrin metabolism	heme	LC/MS neg	41754	C00032	HMDB03178	0.36	0.0008	0.0124	1.7661	0.6437	100	100	Cofactors and vitamins	Hemoglobin and porphyrin metabolism		14875-96-8;	5309.6	646.1
1314			L-urobilin	LC/MS pos	40173	C05793	HMDB04159	0.64	0.1752	0.3385	2.0936	1.3317	100	100	Cofactors and vitamins	Hemoglobin and porphyrin metabolism	5280818	34217-90-8;	4091	595.4
1324	Cofactors and vitamins	Nicotinate and nicotinamide metabolism	nicotinamide	LC/MS pos	594	C00153	HMDB01406	0.95	0.3469	0.4775	1.0290	0.9761	100	100	Cofactors and vitamins	Nicotinate and nicotinamide metabolism	936	98-92-0;	1267	123.1
1338			nicotinate	LC/MS pos	1504	C00253	HMDB01488	1.17	0.7730	0.6872	1.0816	1.2701	100	100	Cofactors and vitamins	Nicotinate and nicotinamide metabolism	938	59-67-6;	1241	124.1
1347		Pantothenate and CoA metabolism	pantothenate	LC/MS neg	1508	C00864	HMDB00210	1.66	0.0000	0.0029	0.7809	1.2960	100	100	Cofactors and vitamins	Pantothenate and CoA metabolism	6613	137-08-6;	1486	218.2
1365		Riboflavin metabolism	flavin adenine dinucleotide (FAD)	LC/MS neg	2134	C00016	HMDB01248	1.44	0.3464	0.4775	0.5174	0.7455	56	73	Cofactors and vitamins	Riboflavin metabolism	643975	146-14-5;84366-81-4;	2413	784.1
1372		Tocopherol metabolism	alpha-tocopherol	GC/MS	1561	C02477	HMDB01893	0.98	0.5843	0.6132	0.9948	0.9774	100	91	Cofactors and vitamins	Tocopherol metabolism	14985	59-02-9;10191-41-0;	2305.4	502.5
1389		Vitamin B6 metabolism	pyridoxate	LC/MS neg	31555	C00847	HMDB00017	0.27	0.0083	0.0617	2.0938	0.5731	100	100	Cofactors and vitamins	Vitamin B6 metabolism	6723	82-82-6 ;	2210	182.1
1395			hippurate	LC/MS neg	15753	C01586	HMDB00714	1.02	0.9463	0.7469	0.9104	0.9305	100	100	Xenobiotics	Benzoate metabolism	464	495-69-2;	2136	178.1
1407		Benzoate metabolism	catechol sulfate	LC/MS neg	35320	C00090		1.31	0.1329	0.2860	0.8102	1.0619	67	100	Xenobiotics	Benzoate metabolism	3083879	120-80-9;	1928	188.9
1408			benzoate	GC/MS	15778	C00180	HMDB01870	0.65	0.0188	0.1000	1.3747	0.8961	100	100	Xenobiotics	Benzoate metabolism	243	65-85-0;	1291.5	179
1436		Chemical	glycerol 2-phosphate	GC/MS	27728	C02979	HMDB02520	1.52	0.1010	0.2417	0.9292	1.4170	100	100	Xenobiotics	Chemical	2526	819-83-0;	1691.8	243
1518.93			2,8-quinolinediol	LC/MS pos	42975	C06342	C06342	0.29	0.0057	0.0503	2.2972	0.6596	100	100	Xenobiotics	Chemical	97250	15450-76-7;	3305	162.2
1583		Drug	pentobarbital	LC/MS neg	38156			0.80	0.5365	0.5940	1.4383	1.1469	100	100	Xenobiotics	Drug	4737	76-74-4;	4564	225.2
1683			enterolactone	LC/MS neg	39626			0.56	0.0240	0.1160	1.4150	0.7899	100	100	Xenobiotics	Food component/Plant	10685477	78473-71-9;	4492.7	297.2
1715	Xenobiotics		equol	LC/MS neg	36774	C14131	HMDB02209	0.95	0.7829	0.6888	1.2455	1.1843	100	100	Xenobiotics	Food component/Plant	382975	94105-90-5;	4502	241.1
1729			ergothioneine	LC/MS pos	37459	C05570	HMDB03045	0.81	0.1606	0.3263	1.1974	0.9742	100	100	Xenobiotics	Food component/Plant	3032311	58511-63-0;	847	230.2
1732			N-glycolylneuraminate	GC/MS	37123	C03410	HMDB00833	1.55	0.0454	0.1591	0.9031	1.4010	100	100	Xenobiotics	Food component/Plant	4603312	1113-83-3;	2119	444.2
1753		Food component/Plant	stachydrine	LC/MS pos	34384	C10172	HMDB04827	1.79	0.0370	0.1458	0.9030	1.6193	100	100	Xenobiotics	Food component/Plant	115244	4136-37-2;	860	144.1
1754			homostachydrine*	LC/MS pos	33009	C08283		1.76	0.0651	0.1915	0.9206	1.6157	100	100	Xenobiotics	Food component/Plant	441447	1195-94-4;	1199	158.2
1759			sitostanol	GC/MS	37524	C19644		0.83	0.4144	0.5176	1.0086	0.8399	100	73	Xenobiotics	Food component/Plant		19466-47-8;	2388	215.2
1760			2-oxindole-3-acetate	LC/MS pos	40479			0.47	0.7569	0.6834	2.0702	0.9634	67	91	Xenobiotics	Food component/Plant	3080590	2971-31-5;	3217.2	192.1
1788			1H-quinolin-2-one	LC/MS pos	40464	C06415		0.38	0.0921	0.2277	1.9367	0.7281	100	91	Xenobiotics	Food component/Plant	6038	59-31-4;	3533	146.2

Supplemental Table S12. Metabolic markers of *Trichuris suis* infection colon contents 33 dpi

Heat map of statistically significant biochemicals profiled in this study.

Comparison mean values significantly different:
- $p \le 0.05$, fold of change ≥ 1.00
- $p \le 0.05$, fold of change < 1.00

Comparison mean values approaching significance:
- $0.05 < p < 0.10$, fold of change ≥ 1.00
- $0.05 < p < 0.10$, fold of change < 1.00

								Fold of Change, Welch's Two-Sample t-Test	Statistical Values, Welch's Two-Sample t-Test, Infected / Non-Infected		Mean Values		% Filled Values							
Pathway Sort Order	Super Pathway	Sub Pathway	Biochemical Name	Platform	Comp ID	KEGG	HMDB	Infected / Non-Infected	p-Value	q-Value	Non-Infected	Infected	Non-Infected	Infected	Super Pathway	Sub Pathway	PubChem	CAS	RI	Mass
1	Amino acid	Glycine, serine and threonine metabolism	glycine	GC/MS	11777	C00037	HMDB00123	3.26	0.0206	0.0525	0.8226	2.6841	100	100	Amino acid	Glycine, serine and threonine metabolism	5257127;750	56-40-6;	1166	101.9
2			sarcosine (N-Methylglycine)	GC/MS	1516	C00213	HMDB00271	2.65	0.0236	0.0583	0.3690	0.9774	22	55	Amino acid	Glycine, serine and threonine metabolism	1088;7311726	107-97-1;	1182.9	116
4			N-acetylglycine	LC/MS neg	27710		HMDB00532	2.62	0.0037	0.0154	0.8623	2.2635	100	100	Amino acid	Glycine, serine and threonine metabolism	10972	543-24-8;	735	116.1
8			serine	GC/MS	1648	C00065	HMDB00187	1.59	0.1291	0.1886	0.9149	1.4554	100	100	Amino acid	Glycine, serine and threonine metabolism	5951;6857581	56-45-1;	1389.1	204
12			homoserine	GC/MS	23642	C00263	HMDB00719	0.98	0.8015	0.5636	0.9961	0.9806	100	100	Amino acid	Glycine, serine and threonine metabolism	12647;6971022	672-15-1;	1471	218.1
17			threonine	LC/MS pos	1284	C00188	HMDB00167	0.98	0.7717	0.5517	0.9754	0.9551	100	100	Amino acid	Glycine, serine and threonine metabolism	6971019;6288	72-19-5;	713	120.1
18			N-acetylthreonine	LC/MS neg	33939	C01118		0.88	0.7487	0.5517	0.9108	0.8056	22	27	Amino acid	Glycine, serine and threonine metabolism	4651717	17093-74-2;	846	160.1
24		Alanine and aspartate metabolism	aspartate	GC/MS	15996	C00049	HMDB00191	0.95	0.9527	0.6084	1.0473	0.9982	100	100	Amino acid	Alanine and aspartate metabolism	5960	56-84-8;	1529.7	232
28			beta-alanine	GC/MS	55	C00099	HMDB00056	0.84	0.2888	0.3249	1.2327	1.0316	100	100	Amino acid	Alanine and aspartate metabolism	239;4755801	56-41-7;107-95-9;	1451.8	174
32			alanine	GC/MS	1126	C00041	HMDB00161	1.54	0.2883	0.3249	1.0721	1.6522	100	100	Amino acid	Alanine and aspartate metabolism	5950;7311724	56-41-7;	1147.6	115.9
35			N-acetylaspartate (NAA)	LC/MS pos	22185	C01042	HMDB00812	1.01	0.6056	0.4919	1.0844	1.0907	100	100	Amino acid	Alanine and aspartate metabolism	65065	997-55-7;997-55-7 ;	1222	176.1
38			2,6-pyridinedicarboxylate	GC/MS	15135			0.85	0.6821	0.5412	1.3287	1.1319	100	100	Amino acid	Alanine and aspartate metabolism	10367	499-83-2;	1721.3	296.1
39		Glutamate metabolism	glutamate	LC/MS pos	57	C00025	HMDB03339	0.68	0.0016	0.0081	1.2226	0.8305	100	100	Amino acid	Glutamate metabolism	611	56-86-0;	700	148.1
45			glutamine	LC/MS pos	53	C00064	HMDB00641	0.96	0.7529	0.5517	0.9821	0.9477	89	100	Amino acid	Glutamate metabolism	5961	56-85-9;	684	147.2
47			gamma-aminobutyrate (GABA)	GC/MS	1416	C00334	HMDB00112	1.20	0.6896	0.5412	1.0277	1.2383	100	100	Amino acid	Glutamate metabolism	6992099;119	56-12-2;	1539.7	304.1
48			carboxyethyl-GABA	LC/MS pos	40007		HMDB02201	0.64	0.8233	0.5696	1.3787	0.8867	67	91	Amino acid	Glutamate metabolism	2572	4386-03-2;	810	176.2
50			N-acetylglutamate	LC/MS pos	15720	C00624	HMDB01138	0.87	0.4558	0.4169	1.3620	1.1784	100	100	Amino acid	Glutamate metabolism	1549099	5817-08-3;	1492	190.1
53		Histidine metabolism	histidine	GC/MS	37424	C00135	HMDB00177	1.54	0.2873	0.3249	0.9744	1.4964	89	91	Amino acid	Histidine metabolism	773;3651426	5934-29-2;	1837.3	254.1
54			N-acetylhistidine	LC/MS neg	33946	C02997		1.28	0.1946	0.2511	1.1530	1.4717	100	100	Amino acid	Histidine metabolism	75619	39145-52-3;	913	196.2
55			trans-urocanate	LC/MS pos	607	C00785	HMDB00301	1.00	0.6881	0.5412	1.0356	1.0354	100	100	Amino acid	Histidine metabolism	736715	104-98-3;	1119	139.1
58			histamine	GC/MS	1574	C00388	HMDB00870	---	---	---	1.0000	1.0000	0	9	Amino acid	Histidine metabolism	774	51-45-6;	1808	174.1
64			1-methylimidazoleacetate	LC/MS pos	32350	C05828	HMDB02820	0.49	0.0147	0.0406	1.5127	0.7351	100	91	Amino acid	Histidine metabolism	75810	2625-49-2 ;	845	141.1
65			imidazole propionate	LC/MS pos	40730		HMDB02271	3.38	0.1077	0.1667	0.7791	2.6334	100	100	Amino acid	Histidine metabolism	70630	1074-59-5;	1011.7	141.1
68		Lysine metabolism	cadaverine	GC/MS	15308	C01672	HMDB02322	6.73	0.0574	0.1120	0.8773	5.9012	89	100	Amino acid	Lysine metabolism		462-94-2;1476-39-7;	1780.4	174
69			glutarate (pentanedioate)	GC/MS	396	C00489	HMDB00661	0.73	0.3336	0.3621	2.0927	1.5309	100	100	Amino acid	Lysine metabolism	4418048	110-94-1;	1433.6	261
74			diaminopimelate	GC/MS	15130	C00666	HMDB01370	2.53	0.0000	0.0008	0.6764	1.7115	100	100	Amino acid	Lysine metabolism	1550876;1550875	1476-39-7;2577-62-0;	1874.4	272.1
75			lysine	GC/MS	1301	C00047	HMDB00182	1.34	0.4623	0.4179	0.8535	1.1454	100	100	Amino acid	Lysine metabolism	5962	56-87-1;	1836.7	317.2
80			pipecolate	LC/MS pos	1444	C00408	HMDB00070	1.00	0.8588	0.5833	1.6267	1.6314	100	100	Amino acid	Lysine metabolism	849	4043-87-2;	1120	130.1
84			N2-acetyllysine	LC/MS pos	36751	C12989	HMDB00446	0.77	0.0980	0.1567	1.1892	0.9145	100	100	Amino acid	Lysine metabolism	6992697;92907	1946-82-3;	853	189.1
85			N6-acetyllysine	LC/MS pos	36752	C02727	HMDB00206	1.51	0.0684	0.1281	0.9531	1.4361	100	100	Amino acid	Lysine metabolism	6991978;92832	692-04-6;	1134	189.1
87			2-aminopentanoate	GC/MS	37426	C01826		2.29	0.4221	0.4007	1.0809	2.4804	78	100	Amino acid	Lysine metabolism	5255804;824	760-78-1;	1279	144.1
88		Phenylalanine & tyrosine metabolism	phenyllactate (PLA)	LC/MS neg	22130	C05607	HMDB00779	4.37	0.1743	0.2287	1.0717	4.6866	89	100	Amino acid	Phenylalanine & tyrosine metabolism	3848	828-01-3;	2237	165.1
89			phenylalanine	LC/MS pos	64	C00079	HMDB00159	1.26	0.3359	0.3629	0.9299	1.1720	100	100	Amino acid	Phenylalanine & tyrosine metabolism	6925665;6140	63-91-2;	2056	166.1
92			phenylacetate	LC/MS neg	15958	C07086	HMDB00209	0.62	0.0123	0.0365	1.1906	0.7390	100	100	Amino acid	Phenylalanine & tyrosine metabolism		103-82-2;	2127	135.1
94			p-cresol sulfate	LC/MS neg	36103	C01468	HMDB11635	3.57	0.0084	0.0294	0.9393	3.3520	100	100	Amino acid	Phenylalanine & tyrosine metabolism	4615422	3233-57-7;	2896	187.1
106			tyrosine	LC/MS pos	1299	C00082	HMDB00158	1.24	0.3725	0.3811	0.9451	1.1719	100	100	Amino acid	Phenylalanine & tyrosine metabolism	6057;6942100	60-18-4;	1516	182.1
107			3-(4-hydroxyphenyl)lactate	LC/MS neg	32197	C03672	HMDB00755	3.81	0.1978	0.2522	0.9775	3.7241	100	100	Amino acid	Phenylalanine & tyrosine metabolism	9378	6482-98-0;	1395	181.1
110			tyramine	LC/MS pos	1603	C00483	HMDB00306	2.26	0.7629	0.5517	1.5933	3.5977	100	100	Amino acid	Phenylalanine & tyrosine metabolism	5610	60-19-5;	1503	138.1
124			4-hydroxyphenylacetate	GC/MS	541	C00642	HMDB00020	3.06	0.0651	0.1239	0.9597	2.9359	100	100	Amino acid	Phenylalanine & tyrosine metabolism	4693933	156-38-7;	1630.6	179
125			3-hydroxyphenylacetate	LC/MS neg	1413	C05593	HMDB00440	0.41	0.0004	0.0037	1.3215	0.5415	100	91	Amino acid	Phenylalanine & tyrosine metabolism	6950813	621-37-4;	1604	151.1
131			N-acetylphenylalanine	LC/MS neg	33950	C03519	HMDB00512	1.75	0.1960	0.2515	0.9902	1.7300	100	91	Amino acid	Phenylalanine & tyrosine metabolism	74839	2018-61-3;	2590	206.2
137			2-(4-hydroxyphenyl)propionate	LC/MS neg	35632	C03080		3.00	0.0010	0.0060	0.4202	1.2590	44	91	Amino acid	Phenylalanine & tyrosine metabolism	102526	938-96-5;	1880	165.1
138			3-(3-hydroxyphenyl)propionate	LC/MS neg	35635	C11457	HMDB00375	2.17	0.0777	0.1368	1.0816	2.3441	100	100	Amino acid	Phenylalanine & tyrosine metabolism	91	621-54-5;	2000	165.1
139			3-(4-hydroxyphenyl)propionate	LC/MS neg	39587	C01744	HMDB02199	2.30	0.1017	0.1607	0.9766	2.2444	89	100	Amino acid	Phenylalanine & tyrosine metabolism	10394	501-97-3;	1658	165.1
140			3-phenylpropionate (hydrocinnamate)	LC/MS neg	15749	C05629	HMDB00764	0.76	0.0530	0.1062	1.1697	0.8918	100	100	Amino acid	Phenylalanine & tyrosine metabolism		501-52-0;	2830	149.1
143			4-hydroxycinnamate	LC/MS neg	36770	C00811	HMDB02035	0.71	0.1175	0.1796	1.3325	0.9427	100	100	Amino acid	Phenylalanine & tyrosine metabolism	637542	501-98-4;	1474	163
151		Tryptophan metabolism	kynurenate	LC/MS neg	1417	C01717	HMDB00715	1.46	0.2704	0.3125	0.8028	1.1720	78	100	Amino acid	Tryptophan metabolism	3845	492-27-3;	2243	188.1
153			tryptophan	LC/MS pos	54	C00078	HMDB00929	1.91	0.0283	0.0684	0.8759	1.6697	100	100	Amino acid	Tryptophan metabolism	6923516;6305	73-22-3;	2445	205.1
156			indoleacetate	LC/MS pos	27513	C00954	HMDB00197	6.05	0.0017	0.0082	0.7782	4.7066	100	100	Amino acid	Tryptophan metabolism	802	6505-45-9;	3760	176.1
158			indole-3-carboxylic acid	LC/MS neg	38116	C19837	HMDB03320	0.66	0.0754	0.1357	1.1320	0.7520	100	64	Amino acid	Tryptophan metabolism		771-50-6;	1713	160.1
167			tryptamine	LC/MS pos	6104	C00398	HMDB00303	0.50	0.0018	0.0087	1.4213	0.7121	100	100	Amino acid	Tryptophan metabolism	1150	61-54-1;	2323	161.1
169			serotonin (5HT)	LC/MS pos	2342	C00780	HMDB00259	0.92	0.7700	0.5517	0.9715	0.8895	89	100	Amino acid	Tryptophan metabolism	5202	153-98-0;	1660	177.1
180			indolepropionate	GC/MS	32405		HMDB02302	0.61	0.0201	0.0523	1.3605	0.8264	100	100	Amino acid	Tryptophan metabolism	3744	830-96-6 ;	1925	202.1
183		Valine, leucine and isoleucine metabolism	3-methyl-2-oxovalerate	LC/MS neg	15676	C00671	HMDB03736	1.17	0.7200	0.5442	0.7169	0.8388	44	36	Amino acid	Valine, leucine and isoleucine metabolism	47	51829-07-3;	2106	129.2
189			alpha-hydroxyisocaproate	LC/MS neg	22132	C03264	HMDB00746	5.89	0.2932	0.3251	1.1202	6.5964	100	91	Amino acid	Valine, leucine and isoleucine metabolism	83697	10303-64-7;	1854	131.2
190			isoleucine	LC/MS pos	1125	C00407	HMDB00172	1.33	0.3854	0.3859	0.9674	1.2852	100	100	Amino acid	Valine, leucine and isoleucine metabolism	791	73-32-5;	1614	132.1
191			leucine	LC/MS pos	60	C00123	HMDB00687	1.40	0.2272	0.2763	0.8858	1.2372	100	100	Amino acid	Valine, leucine and isoleucine metabolism	7045798;6106	61-90-5;	1674	132.2
194			methylsuccinate	GC/MS	15745		HMDB01844	0.53	0.0288	0.0684	1.3305	0.7089	89	91	Amino acid	Valine, leucine and isoleucine metabolism	10349	498-21-5;	1362.1	261
195			N-acetylleucine	LC/MS neg	1587	C02710	HMDB11756	1.43	0.6827	0.5412	1.0808	1.5485	78	91	Amino acid	Valine, leucine and isoleucine metabolism	70912	1188-21-2;	2385	172.1
197			N-acetylisoleucine	LC/MS neg	33967			1.51	0.3441	0.3650	1.0298	1.5530	89	100	Amino acid	Valine, leucine and isoleucine metabolism	2802421	3077-46-1;	2279	172.2
200			valine	LC/MS pos	1649	C00183	HMDB00883	1.46	0.2276	0.2763	0.9242	1.3464	100	100	Amino acid	Valine, leucine and isoleucine metabolism	6971018;6287	72-18-4;	1040	118.1
203			4-methyl-2-oxopentanoate	GC/MS	15683	C00233	HMDB00695	0.39	0.0087	0.0298	2.2565	0.8821	100	100	Amino acid	Valine, leucine and isoleucine metabolism	70	816-66-0;	1283.9	259.1
208			alpha-hydroxyisovalerate	GC/MS	33937		HMDB00407	3.20	0.4559	0.4169	1.1970	3.8256	100	91	Amino acid	Valine, leucine and isoleucine metabolism	99823	600-37-3;	1208	145.1
211			2-hydroxy-3-methylvalerate	LC/MS neg	36746		HMDB00317	3.21	0.7202	0.5442	1.2911	4.1451	78	82	Amino acid	Valine, leucine and isoleucine metabolism	164623	488-15-3;	1787	131.1
224		Cysteine, methionine, SAM, taurine metabolism	cysteine	GC/MS	31453	C00097	HMDB00574	1.14	0.8240	0.5696	1.0374	1.1805	100	100	Amino acid	Cysteine, methionine, SAM, taurine metabolism	5862;6419722	52-90-4;56-89-3;	1560.1	218
230			methionine sulfoxide	LC/MS pos	18374	C02989	HMDB02005	0.91	0.2939	0.3251	1.0526	0.9618	100	100	Amino acid	Cysteine, methionine, SAM, taurine metabolism	158980;1548907	3226-65-1;	729	166.1
231			N-formylmethionine	LC/MS neg	2829	C03145	HMDB01015	1.29	0.1464	0.2060	0.9051	1.1635	100	91	Amino acid	Cysteine, methionine, SAM, taurine metabolism	439750	4289-98-9;	1541	176.1
237			methionine	LC/MS pos	1302	C00073	HMDB00696	1.17	0.6326	0.5102	0.9335	1.0914	100	100	Amino acid	Cysteine, methionine, SAM, taurine metabolism	6992087;6137	63-68-3;	1252	150.1
239			N-acetylmethionine	LC/MS neg	1589	C02712	HMDB11745	1.64	0.1645	0.2239	0.8070	1.3240	100	100	Amino acid	Cysteine, methionine, SAM, taurine metabolism	448580	65-82-7;	1805	190.1
241			2-hydroxybutyrate (AHB)	GC/MS	21044	C05984	HMDB00008	3.45	0.1198	0.1807	0.8056	2.7816	56	82	Amino acid	Cysteine, methionine, SAM, taurine metabolism	440864	3347-90-8;	1169.4	130.9
255		Urea cycle; arginine-, proline-, metabolism	arginine	GC/MS	1638	C00062	HMDB00517	1.32	0.7772	0.5521	1.0762	1.4245	100	100	Amino acid	Urea cycle; arginine-, proline-, metabolism	5246487;232	1119-34-2;	1608.5	142
258			ornithine	GC/MS	1493	C00077	HMDB03374	2.67	0.1658	0.2239	0.8639	2.3039	100	100	Amino acid	Urea cycle; arginine-, proline-, metabolism	6262	3184-13-2;	1763.8	141.9
261			proline	LC/MS pos	1898	C00148	HMDB00162	1.20	0.4288	0.4016	0.9320	1.1166	100	100	Amino acid	Urea cycle; arginine-, proline-, metabolism	145742;6971047	147-85-3;	796	116.1
262			5-aminovalerate	GC/MS	18319	C00431	HMDB03355	5.24	0.0564	0.1111	0.9188	4.8175	100	100	Amino acid	Urea cycle; arginine-, proline-, metabolism	6992101;138	660-88-8;	1620.8	174
263			citrulline	LC/MS pos	2132	C00327	HMDB00904	0.77	0.0115	0.0347	1.1565	0.8895	100	100	Amino acid	Urea cycle; arginine-, proline-, metabolism	833	372-75-8;	715	176.1
264			N-acetylornithine	LC/MS pos	15630	C00437	HMDB03357	1.44	0.4652	0.4189	0.9454	1.3644	100	100	Amino acid	Urea cycle; arginine-, proline-, metabolism	6992102;439232	6205-08-9;	875	175.2
266			N-methyl proline	LC/MS pos	37431			3.62	0.0286	0.0684	0.6793	2.4615	67	100	Amino acid	Urea cycle; arginine-, proline-, metabolism	557	475-11-6;	833	130.1
267			trans-4-hydroxyproline	LC/MS pos	32306	C01157	HMDB00725	0.82	0.0979	0.1567	1.2242	1.0014	100	100	Amino acid	Urea cycle; arginine-, proline-, metabolism	5810;6971053	51-35-4;	705	132.1
274		Creatine metabolism	creatine	LC/MS pos	27718	C00300	HMDB00064	1.51	0.7594	0.5517	1.0560	1.5971	100	100	Amino acid	Creatine metabolism	586	57-00-1;	758	132.1
278		Butanoate metabolism	2-aminobutyrate	GC/MS	1577	C02261	HMDB00650	1.94	0.7801	0.5525	1.0746	2.0890	100	100	Amino acid	Butanoate metabolism	439691;6971251	1492-24-6;	1215.7	130
282		Polyamine metabolism	putrescine	GC/MS	1408	C00134	HMDB01414	2.30	0.4125	0.3992	0.9318	2.1469	100	100	Amino acid	Polyamine metabolism		110-60-1;	1705.8	174
283			N-acetylputrescine	LC/MS pos	37496	C02714	HMDB02064	1.61	0.1704	0.2283	0.9936	1.5967	100	100	Amino acid	Polyamine metabolism	122356	18233-70-0;	895	131.1
284			agmatine	GC/MS	15496	C00179	HMDB01432	5.56	0.2794	0.3213	1.0739	5.9760	100	82	Amino acid	Polyamine metabolism	199	2482-00-0;	1526	174
290		Guanidino and acetamido metabolism	4-guanidinobutanoate	LC/MS pos	15681	C01035	HMDB03464	0.57	0.0026	0.0116	1.6173	0.9160	100	91	Amino acid	Guanidino and acetamido metabolism	500	463-003;463-00-3;	1085	146.1
291			4-acetamidobutanoate	LC/MS pos	1558	C02946	HMDB03681	0.78	0.3534	0.3714	1.0431	0.8156	56	73	Amino acid	Guanidino and acetamido metabolism	18189	3025-96-5;	1724	146.1
297		Glutathione metabolism	5-oxoproline	LC/MS pos	1494	C01879	HMDB00267	0.82	0.1257	0.1860	1.2605	1.0359	100	100	Amino acid	Glutathione metabolism	7405	98-79-3;	1446	130.1
311	Peptide	Dipeptide	glycylvaline	LC/MS pos	18357			1.10	0.7523	0.5517	0.9983	1.0933	100	100	Peptide	Dipeptide	2724807;6994979	1963-21-9;	1572	175.1
314			glycylproline	LC/MS pos	22171		HMDB00721	1.55	0.0669	0.1263	0.9474	1.4730	100	100	Peptide	Dipeptide	3013625;6993386	704-15-4;	1115	173.1
315			glycylisoleucine	LC/MS pos	36659			1.17	0.3763	0.3818	0.9294	1.0920	100	100	Peptide	Dipeptide		19461-38-2;	2080	189.1
316			glycylleucine	LC/MS pos	34398	C02155	HMDB00759	1.15	0.6035	0.4919	1.0387	1.1969	100	100	Peptide	Dipeptide	92843;1548899	869-19-2;	2236	189.1
320			glycyltyrosine	LC/MS pos	33958			0.93	0.7719	0.5517	1.0483	0.9757	100	100	Peptide	Dipeptide	92829;6994980	658-79-7;	1728	239.1
324			alanylvaline	LC/MS pos	37084			0.95	0.4439	0.4141	1.0688	1.0158	100	100	Peptide	Dipeptide	137276	3303-45-5;	1513	189
325			alanylproline	LC/MS pos	37083			1.29	0.2891	0.3249	0.9153	1.1833	100	100	Peptide	Dipeptide	418040	13485-59-1;	1400	187.1
328			alanylleucine	LC/MS pos	37093			1.28	0.7297	0.5468	1.0573	1.3487	100	100	Peptide	Dipeptide	259583	1638-60-4;	2150	203
332			alanylglutamate	LC/MS neg	37064			0.92	0.3907	0.3895	1.0414	0.9544	100	100	Peptide	Dipeptide	656476		637	217
334			alanylisoleucine	LC/MS pos	37118			1.09	0.8142	0.5696	1.1205	1.2202	100	100	Peptide	Dipeptide	417358;5246008		2011	203.1
337.08			asparagylisoleucine	LC/MS pos	42976			0.83	0.3426	0.3650	1.0753	0.8925	100	100	Peptide	Dipeptide			2070	246.2
337.09			asparagylleucine	LC/MS pos	42980			0.86	0.4268	0.4014	1.1425	0.9820	100	100	Peptide	Dipeptide			2170	246.2
339.1			arginylproline	LC/MS pos	41394			1.24	0.5610	0.4688	1.0263	1.2773	100	100	Peptide	Dipeptide	332964	2418-69-1;	785.8	272.2
339.4			arginylvaline	LC/MS pos	41395			1.76	0.0011	0.0066	0.6913	1.2173	89	100	Peptide	Dipeptide	335257	2896-20-0;	944.9	274.2
340			aspartylphenylalanine	LC/MS pos	22175		HMDB00706	1.20	0.3633	0.3750	0.9258	1.1117	89	100	Peptide	Dipeptide	93078	13433-09-5;	2538	281.1
343.3			glutamine-leucine	LC/MS pos	42994			0.98	0.7339	0.5482	1.0780	1.0533	100	100	Peptide	Dipeptide			2180	260.2
343.4			glutamine-isoleucine	LC/MS pos	42988			1.00	0.9325	0.6080	1.0554	1.0562	100	100	Peptide	Dipeptide			2070.6	260.2
358.1			isoleucylthreonine	LC/MS pos	42968			1.30	0.0723	0.1325	0.8173	1.0592	100	100	Peptide	Dipeptide			1555.1	233.2
360			leucylleucine	LC/MS pos	36756	C11332		1.40	0.1785	0.2316	1.0809	1.5096	100	100	Peptide	Dipeptide	76807;6992072	3303-31-9;	3012	245.1
361			leucylisoleucine	LC/MS pos	36757			1.85	0.0753	0.1357	1.0688	1.9807	100	91	Peptide	Dipeptide		36077-41-5;	2800	245.1
361.5			leucylthreonine	LC/MS pos	42969			1.25	0.0693	0.1288	0.9508	1.1884	100	100	Peptide	Dipeptide			1621.9	233.2
363.85			threonylisoleucine	LC/MS pos	42972			1.01	0.9598	0.6084	1.0430	1.0567	100	100	Peptide	Dipeptide			2135	233.2
363.9			threonylvaline	LC/MS pos	42011			1.29	0.1173	0.1796	0.8767	1.1280	100	100	Peptide	Dipeptide	416721;4278432	99032-17-4;	1574.5	219.2
364			threonylphenylalanine	LC/MS pos	31530			1.27	0.2553	0.2995	0.8912	1.1319	100	100	Peptide	Dipeptide	4099799;4099798	16875-27-7;	2500	267.2
376.1			arginylphenylalanine	LC/MS pos	41485			1.34	0.0774	0.1368	0.8595	1.1527	78	100	Peptide	Dipeptide	4441256	2047-13-4;	1795	322.2
379			cyclo(leu-phe)	LC/MS pos	37101			1.05	0.8539	0.5833	0.8354	0.8779	100	73	Peptide	Dipeptide	7076347		4427	261.1
386			valylleucine	LC/MS pos	39994			1.82	0.1276	0.1875	1.0572	1.9211	100	100	Peptide	Dipeptide	352039	22906-55-4;	2503.3	231.2
387			cyclo(phe-phe)	LC/MS pos	38152			1.42	0.0934	0.1535	0.5535	0.7869	33	73	Peptide	Dipeptide		2862-51-3;	4527	295.2
388			arginylisoleucine	LC/MS pos	40070			1.05	0.7226	0.5442	0.8860	0.9347	89	91	Peptide	Dipeptide	433625	62632-70-6;	1554	288.3
389			arginylleucine	LC/MS pos	39995			1.27	0.0724	0.1325	0.8784	1.1180	100	100	Peptide	Dipeptide	333445	1188-24-5;	1620	288.3
390			aspartylleucine	LC/MS pos	40068			1.88	0.0102	0.0322	0.7449	1.4016	100	100	Peptide	Dipeptide	332962	3062-14-4;	2310	247.2
402			isoleucylalanine	LC/MS pos	40046			1.18	0.3492	0.3687	1.0315	1.2122	100	100	Peptide	Dipeptide	5246009;5246010	24787-73-3;	1686	203.2
404			isoleucylglutamate	LC/MS pos	40057			0.95	0.9312	0.6080	1.1273	1.0724	100	100	Peptide	Dipeptide		42516-53-0;	1650	261.2
406			isoleucylglycine	LC/MS pos	40008			1.24	0.1335	0.1914	0.9636	1.1913	100	100	Peptide	Dipeptide	342532	868-28-0;	1732	189.2
412			isoleucylvaline	LC/MS pos	40049			1.31	0.0843	0.1430	1.0240	1.3444	100	100	Peptide	Dipeptide	5246011;435949	41017-96-3;	2202	231.2
413			leucylalanine	LC/MS pos	40010			1.09	0.8237	0.5696	0.9720	1.0642	100	100	Peptide	Dipeptide	259321	7298-84-2;	1760	203.2
417			leucylglutamate	LC/MS pos	40021			1.08	0.5579	0.4678	0.9855	1.0643	100	100	Peptide	Dipeptide	5259589;5259590		1716	261.2
418			leucylglycine	LC/MS pos	40045			1.30	0.1390	0.1969	0.9352	1.2166	100	100	Peptide	Dipeptide	79070	686-50-0;	1800	189.2
426			lysylleucine	LC/MS pos	40020			1.35	0.1074	0.1667	0.9867	1.3306	100	100	Peptide	Dipeptide	4682588	7369-79-1;	1546	260.2
427			lysylproline	LC/MS pos	40666			2.18	0.0078	0.0286	0.7838	1.7067	100	100	Peptide	Dipeptide	3463861	52766-27-5;	775.5	244.2
427.2			lysylvaline	LC/MS pos	42099			1.52	0.0186	0.0502	0.7895	1.2020	100	100	Peptide	Dipeptide	140709	20556-11-0;	876.2	246.2
428			phenylalanylleucine	LC/MS pos	40192			1.44	0.1987	0.2522	0.9214	1.3301	78	100	Peptide	Dipeptide	4078229	3303-55-7;	3252	279.2
431			serylleucine	LC/MS pos	40066			1.24	0.3950	0.3921	1.0097	1.2503	100	100	Peptide	Dipeptide		6665-16-3;	2106	219.2
432			serylphenyalanine	LC/MS pos	40054			0.91	0.9371	0.6080	1.0502	0.9544	89	100	Peptide	Dipeptide			2348	253.2
433			threonylleucine	LC/MS pos	40051			1.05	0.7746	0.5519	1.0579	1.1108	100	100	Peptide	Dipeptide	4420322	50299-12-2;	2204	233.2
445			valylisoleucine	LC/MS pos	40050			1.34	0.2285	0.2763	1.0491	1.4039	100	100	Peptide	Dipeptide	5246012;5246013	20556-14-3;	2365	231.2
448			serylisoleucine*	LC/MS pos	40193			1.24	0.5128	0.4396	1.0194	1.2629	100	100	Peptide	Dipeptide		510-791-9560;	2021	219.2
453.1			phenylalanylproline	LC/MS neg	41392			4.38	0.3670	0.3772	1.0887	4.7719	67	64	Peptide	Dipeptide	4069131	7669-65-0;	3170	261.2
460		gamma-glutamyl	gamma-glutamylvaline	LC/MS pos	32393		HMDB11172	1.12	0.7144	0.5442	1.3066	1.4581	100	100	Peptide	gamma-glutamyl		2746-34-1;	2040	247.2
462			gamma-glutamylleucine	LC/MS pos	18369		HMDB11171	0.97	0.8896	0.5938	1.2416	1.1986	100	100	Peptide	gamma-glutamyl	151023	2566-39-4;	2744	261.2
463			gamma-glutamylisoleucine*	LC/MS pos	34456		HMDB11170	0.91	0.7411	0.5486	1.2784	1.1600	100	100	Peptide	gamma-glutamyl			2644	261.2
466			gamma-glutamylmethionine	LC/MS pos	37539			0.62	0.5871	0.4836	1.7053	1.0606	100	91	Peptide	gamma-glutamyl	7009567	17663-87-5;	1993	279.2
467			gamma-glutamylglutamate	LC/MS pos	36738			0.65	0.9909	0.6201	1.8427	1.1899	100	100	Peptide	gamma-glutamyl	92865	1116-22-9;	1036	277.1
470			gamma-glutamylphenylalanine	LC/MS pos	33422		HMDB00594	0.85	0.6851	0.5412	1.3152	1.1207	100	100	Peptide	gamma-glutamyl	111299	7432-24-8;	2846	295.1
471			gamma-glutamyltyrosine	LC/MS pos	2734			0.62	0.0508	0.1053	1.4649	0.9076	100	100	Peptide	gamma-glutamyl	94340	7432-23-7;	2073	311.2
472			gamma-glutamylthreonine*	LC/MS pos	33364			1.05	0.5411	0.4587	1.1138	1.1673	100	100	Peptide	gamma-glutamyl		5652-48-2;	977	249.1
509	Carbohydrate	Aminosugars metabolism	glucosamine	GC/MS	18534	C00329	HMDB01514	2.22	0.0267	0.0652	0.7561	1.6753	100	91	Carbohydrate	Aminosugars metabolism	441477	66-84-2;	1833	203
510			N-acetylgalactosamine	GC/MS	5842	C01074	HMDB00835	3.39	0.0095	0.0305	0.7185	2.4333	100	100	Carbohydrate	Aminosugars metabolism	35717	14215-68-0;	1895	245.1
511			N-acetylglucosamine	GC/MS	15096	C00140	HMDB00215	3.25	0.0006	0.0045	0.7027	2.2823	100	100	Carbohydrate	Aminosugars metabolism	24139	7512-17-6;	1934.5	245
517			N-acetylneuraminate	LC/MS pos	32377	C00270	HMDB00230	2.68	0.0026	0.0116	0.8154	2.1873	100	100	Carbohydrate	Aminosugars metabolism		131-48-6;	787	310
523			fucose	GC/MS	15821	C02095	HMDB00174	3.56	0.0007	0.0045	0.7840	2.7898	100	100	Carbohydrate	Aminosugars metabolism	3034656	2438-80-4;	1682.2	204
525			N-acetylmannosamine	GC/MS	15059	C00140	HMDB00835	3.55	0.0012	0.0068	0.6224	2.2081	78	100	Carbohydrate	Aminosugars metabolism	439281	7772-94-3;	1882.5	245.1
535		Fructose, mannose, galactose, starch, and sucrose metabolism	N-acetylmuramate	LC/MS neg	18354	C02713		1.63	0.1472	0.2060	0.8359	1.3620	100	100	Carbohydrate	uctose, mannose, galactose, starch, and sucrose metabol	82756	10597-89-4;	877	292.1
537			fructose	GC/MS	577	C00095	HMDB00660	1.95	0.0453	0.0975	0.8351	1.6269	100	100	Carbohydrate	uctose, mannose, galactose, starch, and sucrose metabol	5984	57-48-7;	1758	217
539			galactose	GC/MS	12055	C01582	HMDB00143	3.25	0.0013	0.0068	0.7292	2.3666	100	100	Carbohydrate	uctose, mannose, galactose, starch, and sucrose metabol	3037556	59-23-4;	1793.8	203.9
546			cellobiose	GC/MS	15782	C00185	HMDB00055	0.95	0.4105	0.3992	1.0262	0.9756	100	91	Carbohydrate	uctose, mannose, galactose, starch, and sucrose metabol	10712	528-50-7;	2179	204
547			maltose	GC/MS	15806	C00208	HMDB00163	1.10	0.9034	0.5996	0.9738	1.0696	100	100	Carbohydrate	uctose, mannose, galactose, starch, and sucrose metabol	439341	6363-53-7;	2142.1	204.1
548			mannitol	GC/MS	15335	C00392	HMDB00765	7.68	0.3409	0.3649	0.0265	0.2035	11	9	Carbohydrate	uctose, mannose, galactose, starch, and sucrose metabol	6251	69-65-8;	1839	319.1
552			mannose	GC/MS	584	C00159	HMDB00169	2.61	0.0140	0.0396	0.7408	1.9299	100	100	Carbohydrate	uctose, mannose, galactose, starch, and sucrose metabol	161658	3458-28-4;	1753.5	203.9
554			mannose-6-phosphate	GC/MS	1470	C00275	HMDB01078	1.82	0.0589	0.1141	0.7816	1.4253	89	91	Carbohydrate	uctose, mannose, galactose, starch, and sucrose metabolism		70442-25-0;104872-94-8;	2035.9	387.2
567			maltotriose	GC/MS	27723	C01835	HMDB01262	0.62	0.5464	0.4616	2.0064	1.2418	100	100	Carbohydrate	uctose, mannose, galactose, starch, and sucrose metabol	439586	1109-28-0;	2368.3	361.2
582.8		Oligosaccharide	maltotetraose	LC/MS neg	15910	C02052	HMDB01296	0.44	0.9420	0.6084	3.4393	1.5043	100	100	Carbohydrate	Oligosaccharide	446495	34612-38-9;	965	665.1
585		Glycolysis, gluconeogenesis, pyruvate metabolism	glycerate	GC/MS	1572	C00258	HMDB00139	1.66	0.0852	0.1434	0.7434	1.2368	100	100	Carbohydrate	Glycolysis, gluconeogenesis, pyruvate metabolism	752	600-19-1;	1360.7	189
587			glucose-6-phosphate (G6P)	GC/MS	31260	C00668	HMDB01401	2.23	0.0100	0.0317	0.9347	2.0815	100	100	Carbohydrate	Glycolysis, gluconeogenesis, pyruvate metabolism		103192-55-8;	2042.7	387.2
589			glucose	GC/MS	20488	C00031	HMDB00122	2.68	0.0113	0.0347	0.7500	2.0118	100	100	Carbohydrate	Glycolysis, gluconeogenesis, pyruvate metabolism	79025	50-99-7;	1866.8	217.1
592			fructose-6-phosphate	GC/MS	12021	C05345	HMDB00124	1.77	0.0819	0.1409	0.8886	1.5729	100	100	Carbohydrate	Glycolysis, gluconeogenesis, pyruvate metabolism		103213-47-4;	1994.9	315.1
604			lactate	GC/MS	527	C00186	HMDB00190	2.24	0.4916	0.4309	1.2443	2.7830	100	100	Carbohydrate	Glycolysis, gluconeogenesis, pyruvate metabolism	612	79-33-4;	1102.8	116.9
622			ribose	GC/MS	12083	C00121	HMDB00283	1.42	0.4227	0.4007	1.0094	1.4364	100	100	Carbohydrate	Nucleotide sugars, pentose metabolism	5311110	50-69-1;	1652	204
623			2-deoxyribose	GC/MS	11734	C01801	HMDB03224	4.18	0.0831	0.1420	0.8630	3.6052	78	91	Carbohydrate	Nucleotide sugars, pentose metabolism	5460005	533-67-5;	1523.8	115.9

627		Nucleotide sugars, pentose metabolism	ribulose	GC/MS	35855	C00309	HMDB00621	2.41	0.0134	0.0393	0.7012	1.6910	100	100	Carbohydrate	Nucleotide sugars, pentose metabolism	79021	488-84-6;	1662	306.1
634			arabinose	GC/MS	575	C00216	HMDB00646	1.69	0.1070	0.1667	0.8443	1.4299	100	100	Carbohydrate	Nucleotide sugars, pentose metabolism	66308	28697-53-2;	1631.6	217
637			xylose	GC/MS	15835	C00181	HMDB00098	1.19	0.9214	0.6063	1.1519	1.3747	100	100	Carbohydrate	Nucleotide sugars, pentose metabolism	95259	609-06-3;	1723.9	204
639			xylulose	GC/MS	18344	C00310	HMDB00654	3.82	0.1354	0.1929	0.7317	2.7956	67	55	Carbohydrate	Nucleotide sugars, pentose metabolism	5289590	551-84-8;	1668	306.1
657			tricarballylate	LC/MS neg	15729	C19806		0.35	0.0938	0.1535	2.9019	1.0064	89	100	Energy	Krebs cycle	14925	99-14-9;	592	175
658			alpha-ketoglutarate	GC/MS	33453	C00026	HMDB00208	0.43	0.0386	0.0870	1.6422	0.7057	89	73	Energy	Krebs cycle	51	305-72-6;328-50-7;22202-68-2;	1779	419.1
659	Energy	Krebs cycle	succinate	GC/MS	1437	C00042	HMDB00254	1.29	0.6714	0.5378	1.1720	1.5140	100	100	Energy	Krebs cycle	1110	110-15-6;	1348	247
663			fumarate	GC/MS	1643	C00122	HMDB00134	1.07	0.5096	0.4395	0.9938	1.0651	100	100	Energy	Krebs cycle		100-17-8;	1382.1	245
667			malate	GC/MS	1303	C00149	HMDB00156	1.00	0.8694	0.5854	1.0907	1.0933	100	100	Energy	Krebs cycle	525	6915-15-7;	1502	233
671		Oxidative phosphorylation	phosphate	GC/MS	11438	C00009	HMDB01429	2.80	0.0038	0.0154	0.6997	1.9560	100	100	Energy	Oxidative phosphorylation	1061	7664-38-2 ;	1307.7	298.9
673			linoleate (18:2n6)	LC/MS neg	1105	C01595	HMDB00673	0.99	0.8791	0.5885	1.1000	1.0935	100	100	Lipid	Essential fatty acid	5280450	60-33-3;	5533	279.3
677			linolenate [alpha or gamma; (18:3n3 or 6)]	LC/MS neg	34035	C06427	HMDB01388	1.03	0.9459	0.6084	1.3523	1.3891	100	100	Lipid	Essential fatty acid			5450	277.3
678		Essential fatty acid	dihomo-linolenate (20:3n3 or n6)	LC/MS neg	35718	C03242	HMDB02925	4.94	0.0001	0.0008	0.5595	2.7637	100	100	Lipid	Essential fatty acid	5312529	17046-59-2 ;	5600	305.4
680			docosapentaenoate (n3 DPA; 22:5n3)	LC/MS neg	32504	C16513	HMDB01976	4.74	0.0004	0.0034	0.6378	3.0212	100	100	Lipid	Essential fatty acid		2234-74-4 ;	5574	329.4
681			docosapentaenoate (n6 DPA; 22:5n6)	LC/MS neg	37478	C16513	HMDB13123	5.32	0.0001	0.0015	0.3916	2.0820	78	100	Lipid	Essential fatty acid	6441454	25182-74-5;	5625	329.4
684		Short chain fatty acid	valerate	LC/MS neg	33443	C00803	HMDB00892	0.70	0.0345	0.0802	1.2472	0.8770	100	100	Lipid	Short chain fatty acid	7991	109-52-4;	1774	101.2
685			3-methyladipate	GC/MS	36749		HMDB00555	0.14	0.0005	0.0040	1.1716	0.1690	89	36	Lipid	Short chain fatty acid	12292	3058-01-3;	1546	289.2
687			caproate (6:0)	LC/MS neg	32489	C01585	HMDB00535	0.68	0.3168	0.3472	5.8459	3.9802	100	100	Lipid	Medium chain fatty acid	8892	142-62-1;	2766	115.2
688			heptanoate (7:0)	LC/MS neg	1644	C17714	HMDB00666	0.88	0.9129	0.6041	2.0407	1.7973	100	100	Lipid	Medium chain fatty acid	8094	111-14-8;	3695	129.2
689		Medium chain fatty acid	caprylate (8:0)	LC/MS neg	32492	C06423	HMDB00482	0.83	0.2148	0.2683	1.0405	0.8605	100	100	Lipid	Medium chain fatty acid	379	124-07-2;	4367	143.2
690			pelargonate (9:0)	LC/MS neg	12035	C01601	HMDB00847	0.73	0.0085	0.0294	1.1673	0.8550	100	100	Lipid	Medium chain fatty acid	5461016	112-05-0;	4847	157.2
691			caprate (10:0)	LC/MS neg	1642	C01571	HMDB00511	0.79	0.0355	0.0809	1.1457	0.9089	100	100	Lipid	Medium chain fatty acid	2969	112-37-8;	5092	171.2
694			laurate (12:0)	LC/MS neg	1645	C02679	HMDB00638	0.66	0.0114	0.0347	1.3093	0.8663	100	100	Lipid	Medium chain fatty acid	3893	143-07-7;	5288	199.3
698			myristate (14:0)	GC/MS	1365	C06424	HMDB00806	0.41	0.0000	0.0007	1.6570	0.6788	100	100	Lipid	Long chain fatty acid	11005	544-63-8;	1789.7	285.2
699			myristoleate (14:1n5)	LC/MS neg	32418	C08322	HMDB02000	0.65	0.0140	0.0396	1.3187	0.8605	100	100	Lipid	Long chain fatty acid	5281119	544-64-9 ;	5338	225.3
700			pentadecanoate (15:0)	GC/MS	1361	C16537	HMDB00826	0.33	0.0000	0.0007	1.9153	0.6340	100	100	Lipid	Long chain fatty acid	13849	1002-84-2;10002-84-2;	1853.5	299.2
701			palmitate (16:0)	LC/MS neg	1336	C00249	HMDB00220	0.95	0.5938	0.4874	1.0802	1.0245	100	100	Lipid	Long chain fatty acid	985	57-10-3;	5619	255.3
702			palmitoleate (16:1n7)	LC/MS neg	33447	C08362	HMDB03229	1.42	0.4911	0.4309	0.9111	1.2974	100	100	Lipid	Long chain fatty acid	445638	373-49-9;	5477	253.3
704			margarate (17:0)	LC/MS neg	1121		HMDB02259	1.40	0.4182	0.4007	1.0620	1.4825	100	100	Lipid	Long chain fatty acid	10465	506-12-7;	5733	269.3
705			10-heptadecenoate (17:1n7)	LC/MS neg	33971			1.47	0.1322	0.1908	0.8917	1.3079	100	100	Lipid	Long chain fatty acid	5312435	29743-97-3;	5558	267.3
706			stearate (18:0)	LC/MS neg	1358	C01530	HMDB00827	0.93	0.7174	0.5442	1.0602	0.9808	100	100	Lipid	Long chain fatty acid	5281	57-11-4;	5886	283.4
708			oleate (18:1n9)	GC/MS	1359	C00712	HMDB00207	0.59	0.0024	0.0112	1.3677	0.8027	100	100	Lipid	Long chain fatty acid	445639	112-80-1;	1984.4	339.2
710			cis-vaccenate (18:1n7)	GC/MS	33970	C08367		0.33	0.0000	0.0002	2.0446	0.6774	100	100	Lipid	Long chain fatty acid	5282761	506-17-2;	1987	339.3
716			nonadecanoate (19:0)	LC/MS neg	1356	C16535	HMDB00772	2.01	0.1220	0.1817	0.9806	1.9668	100	100	Lipid	Long chain fatty acid	12591	646-30-0;	6073	297.3
717		Long chain fatty acid	10-nonadecenoate (19:1n9)	LC/MS neg	33972			1.09	0.7416	0.5486	1.0057	1.0929	100	100	Lipid	Long chain fatty acid	5312513	73033-09-7;	5775	295.4
719			arachidate (20:0)	GC/MS	1118	C06425	HMDB02212	0.62	0.0061	0.0240	1.2806	0.7918	100	91	Lipid	Long chain fatty acid	10467	506-30-9;	2071.2	369.3
722			eicosenoate (20:1n9 or 11)	LC/MS neg	33587		HMDB02231	1.64	0.0943	0.1535	0.9528	1.5607	100	100	Lipid	Long chain fatty acid			5955	309.4
724			dihomo-linoleate (20:2n6)	LC/MS neg	17805	C16525		2.98	0.0035	0.0147	0.7417	2.2113	100	100	Lipid	Long chain fatty acid	6439848	2091-39-6;	5722	307.3
727			mead acid (20:3n9)	LC/MS neg	35174		HMDB10378	14.14	0.0001	0.0015	0.1782	2.5195	78	91	Lipid	Long chain fatty acid	5312531	20590-32-3;	5642	305.4
728			arachidonate (20:4n6)	LC/MS neg	1110	C00219	HMDB01043	3.72	0.0001	0.0010	0.6181	2.2985	100	100	Lipid	Long chain fatty acid	444899	506-32-1;	5525	303.4
731			behenate (22:0)	GC/MS	12125	C08281	HMDB00944	0.53	0.0007	0.0045	1.5181	0.8012	100	100	Lipid	Long chain fatty acid	8215	112-85-6;	2143	397.4
732			erucate (22:1n9)	LC/MS neg	1552	C08316	HMDB02068	1.31	0.3627	0.3750	1.0595	1.3926	100	100	Lipid	Long chain fatty acid	8216	112-86-7;	6273.3	337.4
733			docosadienoate (22:2n6)	LC/MS neg	32415	C16533		2.91	0.0062	0.0240	0.8143	2.3720	100	100	Lipid	Long chain fatty acid	5282807	7370-49-2 ;	6017	335.4
735			adrenate (22:4n6)	LC/MS neg	32980	C16527	HMDB02226	2.90	0.0007	0.0045	0.6066	1.7611	89	100	Lipid	Long chain fatty acid	5282844	2091-25-0;	5684	331.3
737			lignocerate (24:0)	GC/MS	1364	C08320	HMDB02003	0.74	0.1753	0.2287	1.3457	0.9998	100	100	Lipid	Long chain fatty acid	11197	557-59-5;	2211	425.4
738			nervonate (24:1n9)	GC/MS	41820	C08323	HMDB02368	1.62	0.1648	0.2239	0.8058	1.3079	44	82	Lipid	Long chain fatty acid	5281120	506-37-6 ;	2201	423.4
761			3-hydroxypropanoate	GC/MS	42103	C01013	HMDB00700	0.72	0.1641	0.2239	1.4108	1.0091	100	91	Lipid	Fatty acid, monohydroxy	5459847	503-66-2;	1187.1	177.1
763			5-hydroxyhexanoate	LC/MS neg	31938		HMDB00525	0.29	0.0231	0.0577	2.7087	0.7907	100	45	Lipid	Fatty acid, monohydroxy	170748	44843-89-2 ;	1283	131.2
768		Fatty acid, monohydroxy	3-hydroxydecanoate	LC/MS neg	22053		HMDB02203	1.35	0.3406	0.3649	0.8127	1.0957	100	100	Lipid	Fatty acid, monohydroxy	26612	5561-87-5;	4681	187.2
774			2-hydroxystearate	LC/MS neg	17945	C03045		1.26	0.2537	0.2992	0.8995	1.1358	100	100	Lipid	Fatty acid, monohydroxy	69417	629-22-1;	5705	299.4
776			2-hydroxypalmitate	GC/MS	35675			0.64	0.0469	0.0991	1.4278	0.9111	100	100	Lipid	Fatty acid, monohydroxy	92836	764-67-0;	1976	299.2
780			13-HODE + 9-HODE	LC/MS neg	37752			1.01	0.4862	0.4309	1.0554	1.0625	100	100	Lipid	Fatty acid, monohydroxy			5270	295.2
783		Fatty acid, dihydroxy	9,10-hydroxyoctadec-12(Z)-enoic acid	LC/MS neg	38399	C14828	HMDB04704	0.87	0.3030	0.3336	1.1857	1.0359	100	100	Lipid	Fatty acid, dihydroxy	9966640	263399-34-4;	5177	313.3
785			adipate	LC/MS pos	21134	C06104	HMDB00448	0.31	0.0000	0.0008	2.1074	0.6622	100	100	Lipid	Fatty acid, dicarboxylate	196	124-04-9;	2460	147
786			2-hydroxyglutarate	GC/MS	37253	C02630	HMDB00606	2.26	0.7085	0.5442	1.6831	3.8091	100	100	Lipid	Fatty acid, dicarboxylate	43	40951-21-1;	1576	247
790			suberate (octanedioate)	LC/MS pos	15730	C08278	HMDB00893	0.53	0.0000	0.0008	1.3359	0.7088	100	100	Lipid	Fatty acid, dicarboxylate	10457	505-48-6;	3603	175
791			2-octenedioate	LC/MS neg	35120		HMDB00341	0.30	0.0000	0.0000	1.3657	0.4151	100	45	Lipid	Fatty acid, dicarboxylate	5354607	5698-50-0;	1291	171.1
793			sebacate (decanedioate)	LC/MS neg	32398	C08277	HMDB00792	0.30	0.0000	0.0008	1.7776	0.5285	100	100	Lipid	Fatty acid, dicarboxylate	5192	111-20-6;	1778	201.2
794		Fatty acid, dicarboxylate	azelate (nonanedioate)	LC/MS neg	18362	C08261	HMDB00784	0.33	0.0001	0.0011	1.6737	0.5493	100	100	Lipid	Fatty acid, dicarboxylate	2266	123-99-9;	1322	187.2
796			dodecanedioate	LC/MS neg	32388	C02678	HMDB00623	0.27	0.0003	0.0031	1.9700	0.5325	100	100	Lipid	Fatty acid, dicarboxylate	12736	693-23-2 ;	2990	229.2
797			tetradecanedioate	LC/MS neg	35669		HMDB00872	0.54	0.0005	0.0040	1.3234	0.7137	100	100	Lipid	Fatty acid, dicarboxylate	13185	821-38-5;	3970	257.2
798			hexadecanedioate	LC/MS neg	35678		HMDB00672	0.82	0.2017	0.2546	1.1436	0.9432	100	100	Lipid	Fatty acid, dicarboxylate	10459	505-54-4;	4650	285.2
799			octadecanedioate	LC/MS neg	36754		HMDB00782	0.69	0.0526	0.1062	1.3216	0.9091	100	100	Lipid	Fatty acid, dicarboxylate	70095	871-70-5;	5033	313.3
800			undecanedioate	LC/MS neg	35671		HMDB00888	0.38	0.0000	0.0008	1.5270	0.5874	100	100	Lipid	Fatty acid, dicarboxylate	15816	1852-04-6;	2376	215.1
816			12-methyltridecanoic acid	GC/MS	38292			0.22	0.0000	0.0002	2.4233	0.5280	100	91	Lipid	Fatty acid, branched	520298	2724-57-4;	1764	285.2
817			13-methylmyristic acid	GC/MS	38293			0.42	0.0003	0.0027	1.7329	0.7201	100	100	Lipid	Fatty acid, branched	151014	2485-71-4;	1833	299.2
819		Fatty acid, branched	15-methylpalmitate (isobar with 2-methylpalmitate)	LC/MS neg	38768			1.12	0.7239	0.5442	1.2610	1.4118	100	100	Lipid	Fatty acid, branched			5698	269.4
820			isopalmitic acid	GC/MS	38294			0.29	0.0000	0.0008	2.2970	0.6738	100	100	Lipid	Fatty acid, branched	36247	4669-02-7;	1888	313.3
821	Lipid		17-methylstearate	LC/MS neg	38296			0.98	0.9002	0.5992	1.2070	1.1829	100	100	Lipid	Fatty acid, branched	3083779	2724-59-6;	5987	297.4
880			oleic ethanolamide	LC/MS neg	38102		HMDB02088	3.20	0.0220	0.0555	0.8684	2.7752	100	100	Lipid	Endocannabinoid	5283454	11-58-0;111-58-0;	6553	324.5
881		Endocannabinoid	palmitoyl ethanolamide	LC/MS neg	38165		HMDB02100	4.38	0.0069	0.0263	0.8389	3.6785	100	100	Lipid	Endocannabinoid	4671	544-31-0;	6416	298.4
882			stearoyl ethanolamide	LC/MS pos	38625		HMDB13078	2.06	0.0303	0.0711	0.7032	1.4462	67	91	Lipid	Endocannabinoid	27902	111-57-9;	5968.9	328.3
894		Fatty acid metabolism	isovalerate	LC/MS neg	34732	C08262	HMDB00718	0.47	0.0008	0.0050	1.5760	0.7402	100	100	Lipid	Fatty acid metabolism	10430	503-74-2;	1623	101.2
897		Fatty acid synthesis	malonate (propanedioate)	LC/MS neg	15872	C00383	HMDB00691	0.99	0.9617	0.6084	0.9780	0.9639	100	100	Lipid	Fatty acid synthesis	867	141-82-2;26522-22-85-0;	606	103.2
898		Carnitine metabolism	deoxycarnitine	LC/MS pos	36747	C01181	HMDB01161	0.91	0.3549	0.3714	1.0032	0.9163	100	100	Lipid	Carnitine metabolism	725	6249-56-5;	759	146.1
900			3-dehydrocarnitine*	LC/MS pos	32654	C02636	HMDB12154	0.63	0.2041	0.2563	1.4798	0.9324	100	100	Lipid	Carnitine metabolism	6991982	10457-99-5;	1020	160.2
916			cholate	LC/MS pos	22842	C00695	HMDB00619	2.05	0.2931	0.3251	0.7191	1.4736	22	45	Lipid	Bile acid metabolism		81-25-4;	5322	426.2
919.1			glycohyocholate	LC/MS pos	42574			3.01	0.4078	0.3992	1.1247	3.3847	78	82	Lipid	Bile acid metabolism			5115	466.2
920.1			taurohyocholate	LC/MS pos	42603			5.28	0.4470	0.4154	1.9270	10.1784	100	91	Lipid	Bile acid metabolism			5000	514.3
921			chenodeoxycholate	LC/MS neg	37789	C02528	HMDB00518	3.68	0.0789	0.1377	1.1381	4.1830	100	100	Lipid	Bile acid metabolism		474-24-9;474-25-9;	5250	391.4
922			taurochenodeoxycholate	LC/MS neg	18494	C05465	HMDB00951	1.30	0.8694	0.5854	1.3980	1.8114	100	100	Lipid	Bile acid metabolism	387316	6009-98-9;	5218	498.3
924			hyodeoxycholate	LC/MS neg	27531	C15517	HMDB00733	1.43	0.2296	0.2763	0.8665	1.2390	100	100	Lipid	Bile acid metabolism	5283820	83-49-8;	5074	391.4
930			glycochenodeoxycholate	LC/MS pos	32346	C05466	HMDB00637	1.11	0.9360	0.6080	0.8893	0.9845	56	45	Lipid	Bile acid metabolism		16564-43-5;	5320	450.1
935		Bile acid metabolism	lithocholate	LC/MS neg	1483	C03990	HMDB00761	1.15	0.6246	0.5055	1.1238	1.2971	100	100	Lipid	Bile acid metabolism	9903	434-13-9;	5350	375.4
936			dehydrolithocholate	LC/MS neg	31891			0.38	0.0014	0.0076	1.5019	0.5776	100	100	Lipid	Bile acid metabolism	159623	1553-56-6 ;	5337	373.4
939			6-oxolithocholate	LC/MS neg	31902			0.59	0.0395	0.0883	1.3635	0.8011	100	100	Lipid	Bile acid metabolism	164960	2393-61-5 ;	5040	389.4
941			6-beta-hydroxylithocholate	LC/MS neg	36807	C15515	HMDB00811	0.97	0.4617	0.4179	1.1402	1.1014	100	100	Lipid	Bile acid metabolism	5283821	668-49-5;	4826	391.3
943			beta-muricholate	LC/MS pos	31885	C17726	HMDB00415	1.49	0.1718	0.2283	1.0233	1.5257	89	100	Lipid	Bile acid metabolism	3083762	2393-59-1;	5191	391.2
944			hyocholate	LC/MS neg	34093	C17649	HMDB00760	4.65	0.0146	0.0406	0.7315	3.4048	100	100	Lipid	Bile acid metabolism		547-75-1;	5001	407.3
951			tauroursodeoxycholate	LC/MS neg	39378		HMDB00874	2.03	0.7084	0.5442	0.8151	1.6552	56	82	Lipid	Bile acid metabolism		14605-22-2;	5002.6	498.4
952.1			tauro(alpha + beta)muricholate	LC/MS neg	43167			3.18	0.7410	0.5486	0.8606	2.7409	56	27	Lipid	Bile acid metabolism			4743.7	514.4
953		Fatty alcohol, long chain	1-octadecanol	GC/MS	21128	D01924	HMDB02350	0.83	0.2895	0.3249	1.1732	0.9717	100	100	Lipid	Fatty alcohol, long chain	8221	112-92-5;	1957.7	327.3
955			ethanolamine	GC/MS	1497	C00189	HMDB00149	0.95	0.2265	0.2763	1.5235	1.4485	100	100	Lipid	Glycerolipid metabolism		141-43-5;	1304	174.1
959		Glycerolipid metabolism	glycerol	GC/MS	15122	C00116	HMDB00131	0.96	0.4028	0.3965	1.1003	1.0581	100	100	Lipid	Glycerolipid metabolism	753	56-81-5;	1311	205
961			glycerol 3-phosphate (G3P)	GC/MS	15365	C00093	HMDB00126	0.65	0.0425	0.0931	1.4040	0.9135	100	100	Lipid	Glycerolipid metabolism	754	29849-82-9;	1719.7	357.1
962			glycerophosphorylcholine (GPC)	LC/MS pos	15990	C00670	HMDB00086	0.77	0.0874	0.1460	1.1190	0.8655	100	100	Lipid	Glycerolipid metabolism	657272	28319-77-9;	694	258.1
971		Inositol metabolism	myo-inositol	GC/MS	19934	C00137	HMDB00211	3.53	0.0090	0.0302	0.8705	3.0704	100	100	Lipid	Inositol metabolism		87-89-8;	1924.9	217
974			inositol 1-phosphate (I1P)	GC/MS	1481	C04006	HMDB00213	1.26	0.2533	0.2992	1.0925	1.3788	100	100	Lipid	Inositol metabolism		106032-59-1;	2057.8	318.1
984		Ketone bodies	3-hydroxybutyrate (BHBA)	GC/MS	542	C01089	HMDB00357	0.48	0.0002	0.0023	1.8950	0.9074	100	100	Lipid	Ketone bodies	441	625-72-9;	1203.5	116.9
990			1-palmitoylglycerophosphoethanolamine	LC/MS neg	35631		HMDB11503	1.19	0.4589	0.4179	0.8651	1.0281	100	100	Lipid	Lysolipid	9547069		5940	452.3
991			2-palmitoylglycerophosphoethanolamine*	LC/MS neg	35688			1.50	0.2327	0.2787	0.8206	1.2324	100	100	Lipid	Lysolipid			5790	452.3
995			1-stearoylglycerophosphoethanolamine	LC/MS neg	34416		HMDB11130	1.59	0.0459	0.0978	0.8087	1.2869	100	100	Lipid	Lysolipid	9547068	69747-55-3;	6200	480.4
1006			1-stearoylglycerophosphoglycerol	LC/MS neg	34437			0.95	0.7616	0.5517	1.6897	1.6104	100	100	Lipid	Lysolipid			5826	511.4
1010			1-palmitoylglycerophosphocholine	LC/MS pos	33955			1.08	0.8031	0.5636	1.0429	1.1257	100	100	Lipid	Lysolipid	86554	17364-16-8;	5671	496.4
1011		Lysolipid	2-palmitoylglycerophosphocholine*	LC/MS pos	35253			1.01	0.8647	0.5854	0.9176	0.9293	56	55	Lipid	Lysolipid			5604	496.3
1015			1-stearoylglycerophosphocholine	LC/MS pos	33961			1.15	0.4786	0.4276	0.8521	0.9841	100	100	Lipid	Lysolipid	497299	19420-57-6;	5844	524.4
1017			1-oleoylglycerophosphocholine	LC/MS pos	33960			0.92	0.8581	0.5833	1.0984	1.0079	89	82	Lipid	Lysolipid	16081932	19420-56-5;	5700	522.4
1019			1-linoleoylglycerophosphocholine	LC/MS pos	34419	C04100		0.67	0.9214	0.6063	1.4777	0.9919	67	82	Lipid	Lysolipid	11988421		5574	520.4
1032			1-palmitoylglycerophosphoinositol*	LC/MS neg	35305			1.05	0.9852	0.6185	1.3565	1.4275	100	100	Lipid	Lysolipid			5573	571.3
1035			1-stearoylglycerophosphoinositol	LC/MS neg	19324			1.76	0.0170	0.0464	0.6554	1.1556	67	100	Lipid	Lysolipid		796963-93-4;	5800	599.4
1045			1-palmitoylplasmenylethanolamine*	LC/MS neg	39270			1.23	0.4897	0.4309	1.1229	1.3820	100	100	Lipid	Lysolipid			6153	436.4
1049			1-pentadecanoylglycerol (1-monopentadecanoin)	LC/MS neg	34385			0.23	0.0012	0.0066	3.4322	0.7726	100	100	Lipid	Monoacylglycerol	190750		5978	241.3
1051			1-palmitoylglycerol (1-monopalmitin)	GC/MS	21127			1.08	0.8580	0.5833	1.0107	1.0954	100	100	Lipid	Monoacylglycerol	14900	542-44-9;	2119.5	371.3
1055		Monoacylglycerol	1-oleoylglycerol (1-monoolein)	LC/MS pos	21184		HMDB11567	2.78	0.0031	0.0135	0.7446	2.0711	100	100	Lipid	Monoacylglycerol	5283468	111-03-5;	5821	374.1
1056			2-oleoylglycerol (2-monoolein)	GC/MS	35848			1.44	0.2215	0.2737	0.6666	0.9600	33	73	Lipid	Monoacylglycerol	5319879	3443-84-3;	2170	410.1
1057			1-linoleoylglycerol (1-monolinolein)	LC/MS neg	27447			2.31	0.0643	0.1235	1.3035	3.0152	100	100	Lipid	Monoacylglycerol	5283469	2277-28-3;	6226	279.2
1058			2-linoleoylglycerol (2-monolinolein)	LC/MS neg	32506		HMDB11538	2.59	0.0549	0.1091	1.0517	2.7195	100	100	Lipid	Monoacylglycerol	5365676	3443-82-1;	5988	279.3
1074			sphinganine	LC/MS pos	17769	C00836	HMDB00269	1.37	0.2579	0.3011	0.8541	1.1687	100	100	Lipid	Sphingolipid	3126	3102-56-5;	5175	302.3
1077			sphingosine	LC/MS pos	17747	C00319	HMDB00252	2.79	0.0080	0.0287	0.7196	2.0070	100	100	Lipid	Sphingolipid	5353955	123-78-4;	5197	300.2
1078		Sphingolipid	phytosphingosine	LC/MS pos	1510	C12144	HMDB04610	1.35	0.3855	0.3859	0.8893	1.1983	100	100	Lipid	Sphingolipid		554-62-1;	5189	318.4
1080			N-acetylsphingosine	LC/MS neg	17772	C00195		1.03	0.5109	0.4395	1.1972	1.2385	100	100	Lipid	Sphingolipid	5497136	3102-57-6;	5905	340.4
1081			N,N-dimethylsphingosine	LC/MS pos	32460	C13914		2.32	0.0018	0.0087	0.5708	1.3218	78	100	Lipid	Sphingolipid	5282309	119567-63-4;	5192	328.3
1086			palmitoyl sphingomyelin	GC/MS	37506			2.50	0.0093	0.0305	0.7724	1.9321	89	100	Lipid	Sphingolipid	9939941	6254-89-3;	2524	311.3
1100			lathosterol	GC/MS	39864	C01189	HMDB01170	1.07	0.4000	0.3954	0.8371	0.8946	44	73	Lipid	Sterol/Steroid	65728	80-99-9 ;	2337	255.2
1102		Sterol/Steroid	cholesterol	GC/MS	63	C00187	HMDB00067	1.38	0.0437	0.0948	0.8608	1.1864	100	100	Lipid	Sterol/Steroid	6432564	57-88-5;	2316.9	329.3
1139			beta-sitosterol	GC/MS	27414	C01753	HMDB00852	0.68	0.0008	0.0051	1.2197	0.8279	100	100	Lipid	Sterol/Steroid	222284	83-46-5;	2380.8	357.4
1141			campesterol	GC/MS	39511	C01789	HMDB02869	0.60	0.0004	0.0034	1.2483	0.7476	100	100	Lipid	Sterol/Steroid	173183	474-62-4;	2353	343.4
1187			xanthine	LC/MS pos	3147	C00385	HMDB00292	1.16	0.4953	0.4325	1.0053	1.1663	100	100	Nucleotide	Purine metabolism, (hypo)xanthine/inosine containing	1188	69-89-6;	1430	153.1
1188		Purine metabolism, (hypo)xanthine/inosine containing	xanthosine	LC/MS neg	15136	C01762	HMDB00299	0.85	0.5679	0.4711	0.9843	0.8382	89	100	Nucleotide	Purine metabolism, (hypo)xanthine/inosine containing	64959	146-80-5;	1075	283.1
1190			hypoxanthine	GC/MS	3127	C00262	HMDB00157	1.36	0.6481	0.5210	1.0236	1.3957	100	100	Nucleotide	Purine metabolism, (hypo)xanthine/inosine containing	790	68-94-0;	1759.2	265
1192			inosine	GC/MS	1123	C00294	HMDB00195	1.71	0.4153	0.4003	0.7206	1.2327	44	45	Nucleotide	Purine metabolism, (hypo)xanthine/inosine containing		58-63-9;	2114.8	217
1193			2'-deoxyinosine	LC/MS pos	15076	C05512	HMDB00071	1.02	0.9639	0.6084	1.0928	1.1131	100	100	Nucleotide	Purine metabolism, (hypo)xanthine/inosine containing	65058	890-38-0;	1750	252.9
1198			adenine	LC/MS pos	554	C00147	HMDB00034	0.43	0.0034	0.0147	1.7852	0.7713	100	100	Nucleotide	Purine metabolism, adenine containing	190	73-24-5;	1003	136.1
1199			adenosine	LC/MS pos	555	C00212	HMDB00050	0.58	0.0513	0.1055	1.5949	0.9222	100	100	Nucleotide	Purine metabolism, adenine containing	60961	58-61-7;	1650	268.1
1203			2'-deoxyadenosine	LC/MS pos	1553	C00559	HMDB00101	0.45	0.0093	0.0305	1.7327	0.7723	100	91	Nucleotide	Purine metabolism, adenine containing	13730	16373-93-6;	1632	252.1
1205		Purine metabolism, adenine containing	2'-deoxyadenosine 3'-monophosphate	LC/MS pos	40663			1.20	0.4812	0.4283	0.8901	1.0702	100	100	Nucleotide	Purine metabolism, adenine containing	1467	102832-10-0;	1383.5	332.1
1206			adenosine 2'-monophosphate (2'-AMP)	LC/MS pos	36815	C00946	HMDB11617	2.46	0.0007	0.0047	0.5689	1.4001	100	100	Nucleotide	Purine metabolism, adenine containing		130-49-4;	1625	348.1
1207			adenosine 3'-monophosphate (3'-AMP)	LC/MS pos	35142	C01367	HMDB03540	0.66	0.2459	0.2930	1.5231	1.0125	100	100	Nucleotide	Purine metabolism, adenine containing	15938966	84-21-9;	1451	348.1
1208			adenosine 5'-monophosphate (AMP)	LC/MS pos	32342	C00020	HMDB00045	1.32	0.8397	0.5788	1.0684	1.4147	100	100	Nucleotide	Purine metabolism, adenine containing	15938965	149022-20-8;	1210	348.1
1214			adenosine-2',3'-cyclic monophosphate	LC/MS pos	37467	C02353		0.67	0.0815	0.1409	1.2297	0.8213	100	91	Nucleotide	Purine metabolism, adenine containing	2024	37063-35-7;	1484	330.2
1220			guanine	LC/MS pos	32352	C00242	HMDB00132	1.02	0.7190	0.5442	1.0866	1.1067	100	100	Nucleotide	Purine metabolism, guanine containing	764	73-40-5;	1022	152.1
1224			guanosine	LC/MS pos	1573	C00387	HMDB00133	1.45	0.9619	0.6084	2.0950	3.0277	89	100	Nucleotide	Purine metabolism, guanine containing	6802	118-00-3;	1676	284
1225		Purine metabolism, guanine containing	2'-deoxyguanosine	LC/MS pos	1411	C00330	HMDB00085	0.94	0.5149	0.4398	1.0101	0.9524	100	100	Nucleotide	Purine metabolism, guanine containing	187790	961-07-9;	1728	268
1228			guanosine-2',3'-cyclic monophosphate	LC/MS pos	37139	C06194	HMDB11629	0.94	0.3764	0.3818	1.0459	0.9883	100	91	Nucleotide	Purine metabolism, guanine containing	417655	15718-49-7;	1620	346
1245			guanosine 3'-monophosphate (3'-GMP)	LC/MS neg	39786			1.38	0.6843	0.5412	0.7351	1.0149	22	45	Nucleotide	Purine metabolism, guanine containing	3522	117-68-0;	792.2	362.1
1247	Nucleotide	Purine metabolism, urate metabolism	urate	GC/MS	1604	C00366	HMDB00289	0.39	0.0007	0.0045	1.6018	0.6242	100	73	Nucleotide	Purine metabolism, urate metabolism		69-93-2;120K5305;	1928	441.2
1251			cytidine	LC/MS pos	514	C00475	HMDB00089	1.08	0.9520	0.6084	0.9200	0.9907	100	100	Nucleotide	Pyrimidine metabolism, cytidine containing	6175	65-46-3;	1065	244
1255			cytidine 5'-monophosphate (5'-CMP)	LC/MS pos	2372	C00055	HMDB00095	1.49	0.4248	0.4011	0.9712	1.4466	78	82	Nucleotide	Pyrimidine metabolism, cytidine containing	7058165	63-37-6;	887	324
1256			cytidine-3'-monophosphate (3'-CMP)	LC/MS pos	2959	C05822		1.38	0.5531	0.4655	1.0415	1.4347	100	100	Nucleotide	Pyrimidine metabolism, cytidine containing	256622	84-52-6;	1181	323.9
1257		Pyrimidine metabolism, cytidine containing	cytosine-2',3'-cyclic monophosphate	LC/MS pos	37465	C02354	HMDB11691	0.77	0.2658	0.3087	1.1940	0.9245	100	100	Nucleotide	Pyrimidine metabolism, cytidine containing	417654	15718-51-1;	912	306.2
1260			2'-deoxycytidine	LC/MS pos	15949	C00881	HMDB00014	1.70	0.0203	0.0523	0.7342	1.2499	89	100	Nucleotide	Pyrimidine metabolism, cytidine containing	13711	951-77-9;	1256	228
1261			2'-deoxycytidine 3'-monophosphate	LC/MS pos	40453			0.94	0.8555	0.5833	1.0140	0.9559	100	100	Nucleotide	Pyrimidine metabolism, cytidine containing	3274948	6220-63-9;	1048	308
1263			cytosine	GC/MS	573	C00380	HMDB00630	1.83	0.5091	0.4395	1.0530	1.9261	100	100	Nucleotide	Pyrimidine metabolism, cytidine containing	597	71-30-7;	1535.5	254
1267		Pyrimidine metabolism, orotate containing	orotate	GC/MS	1505	C00295	HMDB00226	0.85	0.4192	0.4007	1.4712	1.2573	100	100	Nucleotide	Pyrimidine metabolism, orotate containing	967	50887-69-9;	1709.6	254.1
1271			thymine	GC/MS	604	C00178	HMDB00262	1.73	0.5653	0.4706	1.1594	2.0107	100	100	Nucleotide	Pyrimidine metabolism, thymine containing	1135	65-71-4;	1429.1	255
1272		Pyrimidine metabolism, thymine containing	thymidine	LC/MS neg	2183	C00214	HMDB00273	1.22	0.3570	0.3718	1.2994	1.5809	100	100	Nucleotide	Pyrimidine metabolism, thymine containing	5789	50-89-5;	1935	241.1
1274			thymidine 5'-monophosphate	LC/MS pos	12023	C00364	HMDB01227	0.89	0.7231	0.5442	1.0562	0.9360	100	100	Nucleotide	Pyrimidine metabolism, thymine containing		33430-62-5;	1598	322.9
1277			uracil	GC/MS	605	C00106	HMDB00300	1.45	0.7072	0.5442	1.0072	1.4647	100	100	Nucleotide	Pyrimidine metabolism, uracil containing	1174	66-22-8;	1370.4	241
1279			uridine	LC/MS neg	606	C00299	HMDB00296	1.17	0.7624	0.5517	1.1314	1.3201	100	100	Nucleotide	Pyrimidine metabolism, uracil containing	6029	58-96-8;	1430	243.1
1280			pseudouridine	LC/MS pos	33442	C02067	HMDB00767	1.09	0.7046	0.5442	1.0787	1.1811	100	100	Nucleotide	Pyrimidine metabolism, uracil containing		1445-07-4;	1180	245
1281		Pyrimidine metabolism, uracil containing	2'-deoxyuridine	LC/MS neg	1412	C00526	HMDB00012	1.18	0.5807	0.4800	1.2022	1.4168	89	64	Nucleotide	Pyrimidine metabolism, uracil containing	13712	951-78-0;	1608	227.1
1282			5-methyluridine (ribothymidine)	LC/MS neg	35136		HMDB00884	1.04	0.9559	0.6084	1.2518	1.2991	89	91	Nucleotide	Pyrimidine metabolism, uracil containing	445408	1463-10-1;	1774	257.1
1287			uridine monophosphate (5' or 3')	LC/MS pos	39879		HMDB00288	1.14	0.8784	0.5885	0.9719	1.1067	89	73	Nucleotide	Pyrimidine metabolism, uracil containing		58-97-9;	1079	325
1288			uridine-2',3'-cyclic monophosphate	LC/MS neg	37137	C02355	HMDB11640	1.32	0.4545	0.4169	0.9428	1.2416	56	82	Nucleotide	Pyrimidine metabolism, uracil containing	4634036	40632-52-8;	1082	304.9
1313			heme	LC/MS pos	41754	C00032	HMDB03178	1.87	0.1589	0.2211	0.3345	0.6241	0	36	Cofactors and vitamins	Hemoglobin and porphyrin metabolism		14875-96-8;	5057	616.2
1314		Hemoglobin and porphyrin metabolism	L-urobilin	LC/MS pos	40173	C05793	HMDB04159	0.61	0.0349	0.0802	1.3140	0.7954	100	100	Cofactors and vitamins	Hemoglobin and porphyrin metabolism	5280818	34217-90-8;	4091	595.4
1316			D-urobilin	LC/MS pos	40084	C05795	HMDB04161	6.64	0.0016	0.0081	0.2311	1.5334	11	73	Cofactors and vitamins	Hemoglobin and porphyrin metabolism		3947-38-4;	4621	589.3
1320			biliverdin	LC/MS neg	2137	C00500	HMDB01008	1.77	0.3183	0.3472	0.8351	1.4781	78	82	Cofactors and vitamins	Hemoglobin and porphyrin metabolism		55482-27-4;55482-27-4 ;	4671	581.2
1338		Nicotinate and nicotinamide metabolism	nicotinate	LC/MS pos	1504	C00253	HMDB01488	1.09	0.7868	0.5555	0.9846	1.0719	100	100	Cofactors and vitamins	Nicotinate and nicotinamide metabolism	938	59-67-6;	1221	122.1
1341			nicotinate ribonucleoside*	LC/MS pos	33471	C05841	HMDB06809	2.38	0.1614	0.2232	0.7024	1.6716	67	64	Cofactors and vitamins	Nicotinate and nicotinamide metabolism	161233	2625-49-2;	1105	256
1347			pantothenate	LC/MS pos	1508	C00864	HMDB00210	1.18	0.7212	0.5442	1.0302	1.2136	100	100	Cofactors and vitamins	Pantothenate and CoA metabolism	6613	137-08-6;	2218	220.1
1348	Cofactors and	Pantothenate and CoA metabolism	pantethine	LC/MS neg	39791	C12661	HMDB03828	1.49	0.0945	0.1535	0.8539	1.2734	89	91	Cofactors and vitamins	Pantothenate and CoA metabolism	452306	16816-67-4;	3605.3	553.3

1363	vitamins		2,3-dihydroxyisovalerate	GC/MS	38276	C04039		0.53	0.0490	0.1026	0.6949	0.3708	44	9	Cofactors and vitamins	Pantothenate and CoA metabolism	677	1756-18-9;	1422	131
1365		Riboflavin metabolism	flavin adenine dinucleotide (FAD)	LC/MS neg	2134	C00016	HMDB01248	1.07	0.1217	0.1817	0.8427	0.8989	11	27	Cofactors and vitamins	Riboflavin metabolism	643975	146-14-5;84366-81-4;	2413	784.1
1366			riboflavin (Vitamin B2)	LC/MS pos	1827	C00255	HMDB00244	1.11	0.6033	0.4919	0.9913	1.1029	100	100	Cofactors and vitamins	Riboflavin metabolism	493570	83-88-5;	3111	377.2
1372		Tocopherol metabolism	alpha-tocopherol	GC/MS	1561	C02477	HMDB01893	0.40	0.0001	0.0011	1.6374	0.6596	100	100	Cofactors and vitamins	Tocopherol metabolism	14985	59-02-9;10191-41-0;	2305.4	502.5
1377			gamma-tocopherol	GC/MS	42315	C02483	HMDB01492	0.42	0.0000	0.0000	1.6587	0.6899	100	100	Cofactors and vitamins	Tocopherol metabolism	14986	54-28-4;	2264	223.1
1378			alpha-tocotrienol	GC/MS	37502	C14153	HMDB06327	0.53	0.1720	0.2283	0.8461	0.4455	33	27	Cofactors and vitamins	Tocopherol metabolism	5282347	58864-81-6;	2346	237.1
1379			gamma-tocotrienol	GC/MS	37501	C14155	HMDB12958	0.54	0.0002	0.0024	1.0778	0.5872	100	18	Cofactors and vitamins	Tocopherol metabolism	5282349	14101-61-2;	2297	223.1
1389		Vitamin B6 metabolism	pyridoxate	LC/MS neg	31555	C00847	HMDB00017	0.30	0.0073	0.0275	2.4135	0.7328	100	100	Cofactors and vitamins	Vitamin B6 metabolism	6723	82-82-6 ;	2210	182.1
1408	Xenobiotics	Benzoate metabolism	benzoate	GC/MS	15778	C00180	HMDB01870	1.00	0.7691	0.5517	1.0475	1.0480	100	100	Xenobiotics	Benzoate metabolism	243	65-85-0;	1291.5	179
1409			3-hydroxybenzoate	GC/MS	15673	C00587	HMDB02466	0.97	0.4117	0.3992	0.9965	0.9709	89	55	Xenobiotics	Benzoate metabolism	7420	99-06-9;	1574.2	267
1417			p-aminobenzoate (PABA)	LC/MS pos	596	C00568	HMDB01392	0.33	0.0006	0.0045	1.6330	0.5469	100	73	Xenobiotics	Benzoate metabolism	7057990;978	150-13-0;	2120	138.1
1430		Chemical	glycolate (hydroxyacetate)	GC/MS	15737	C00160	HMDB00115	5.20	0.0201	0.0523	0.8544	4.4453	78	100	Xenobiotics	Chemical	3698251;757	79-14-1;	1119	177
1451			3-hydroxypyridine	GC/MS	21169			0.69	0.0425	0.0931	1.1564	0.7996	100	82	Xenobiotics	Chemical	7971	109-00-2;	1187.1	152
1518.93			2,8-quinolinediol	LC/MS pos	42975	C06342	C06342	0.35	0.0139	0.0396	1.8411	0.6503	100	100	Xenobiotics	Chemical	97250	15450-76-7;	3305	162.2
1583		Drug	pentobarbital	LC/MS neg	38156			1.36	0.9844	0.6185	0.9963	1.3548	100	82	Xenobiotics	Drug	4737	76-74-4;	4564	225.2
1658		Food component/Plant	sinapate	LC/MS neg	21150	C00482		1.02	0.9309	0.6080	1.1968	1.2258	100	100	Xenobiotics	Food component/Plant	637775	530-59-6;	1986	223.1
1664.2			1-methyl-beta-carboline-3-carboxylic acid	LC/MS pos	42938			0.29	0.0000	0.0002	1.4140	0.4165	100	55	Xenobiotics	Food component/Plant	5406157	22329-38-0;	3264.1	227.1
1670			daidzein	LC/MS neg	32453	C10208	HMDB03312	1.04	0.3848	0.3859	1.7050	1.7778	100	100	Xenobiotics	Food component/Plant	5281708	486-66-8 ;	3541	253.1
1672			genistein	LC/MS neg	32448	C06563	HMDB03217	1.34	0.1318	0.1908	1.6939	2.2668	100	100	Xenobiotics	Food component/Plant	5280961	446-72-0 ;	3745	269.1
1683			enterolactone	LC/MS neg	39626			0.38	0.0052	0.0208	1.9113	0.7315	100	100	Xenobiotics	Food component/Plant	10685477	78473-71-9;	4492.7	297.2
1697			apigenin	LC/MS neg	21189	C01477	HMDB02124	1.69	0.1748	0.2287	1.3909	2.3529	89	100	Xenobiotics	Food component/Plant	5280443	520-36-5;	3880	269.1
1700			ferulate	LC/MS neg	35630	C01494	HMDB00954	1.06	0.9550	0.6084	0.9383	0.9918	100	100	Xenobiotics	Food component/Plant	445858	537-98-4;	1810	193
1701			dihydroferulic acid	LC/MS neg	40481			6.28	0.0195	0.0520	0.6917	4.3451	100	100	Xenobiotics	Food component/Plant	14340	1135-23-5;	1929	195.1
1715			equol	LC/MS neg	36774	C14131	HMDB02209	0.86	0.5374	0.4573	1.1923	1.0208	100	100	Xenobiotics	Food component/Plant	382975	94105-90-5;	4502	241.1
1732			N-glycolylneuraminate	LC/MS neg	37123	C03410	HMDB00833	1.75	0.0774	0.1368	0.8648	1.5144	100	100	Xenobiotics	Food component/Plant	4603312	1113-83-3;	653	324
1736			syringic acid	LC/MS neg	37091	C10833	HMDB02085	1.09	0.8211	0.5696	0.9925	1.0782	89	82	Xenobiotics	Food component/Plant	10742	530-57-4;	1416	197
1753			stachydrine	LC/MS pos	34384	C10172	HMDB04827	1.46	0.0985	0.1567	0.8835	1.2937	100	100	Xenobiotics	Food component/Plant	115244	4136-37-2;	860	144.1
1754			homostachydrine*	LC/MS pos	33009	C08283		1.63	0.2209	0.2737	0.9423	1.5317	100	100	Xenobiotics	Food component/Plant	441447	1195-94-4;	1199	158.2
1759			sitostanol	GC/MS	37524	C19644		0.56	0.0001	0.0008	1.4580	0.8097	100	100	Xenobiotics	Food component/Plant		19466-47-8;	2388	215.2
1760			2-oxindole-3-acetate	LC/MS pos	40479			0.79	0.4677	0.4195	1.4003	1.1036	100	100	Xenobiotics	Food component/Plant	3080590	2971-31-5;	3217.2	192.1
1761.5			fumonisin B1	LC/MS pos	42597			1.56	0.1187	0.1802	1.0099	1.5752	78	91	Xenobiotics	Food component/Plant	3431	116355-83-0;	4502	722.5
1769			vanillin	GC/MS	37442	C00755	HMDB12308	0.91	0.4981	0.4333	1.0912	0.9885	78	45	Xenobiotics	Food component/Plant		121-33-5;	1550	194
1788			1H-quinolin-2-one	LC/MS pos	40464	C06415		0.44	0.0522	0.1062	1.9274	0.8502	100	100	Xenobiotics	Food component/Plant	6038	59-31-4;	3533	146.2
1796.3			Isobar: dihydrocaffeate, 3,4-dihydroxycinnamate	GC/MS	40515			3.80	0.4493	0.4158	0.8380	3.1833	56	55	Xenobiotics	Food component/Plant			1852.8	178.9
1818		Sugar, sugar substitute, starch	erythritol	GC/MS	20699	C00503	HMDB02994	1.33	0.9931	0.6201	0.8111	1.0766	78	73	Xenobiotics	Sugar, sugar substitute, starch		149-32-6;	1517.5	217
1824			galacturonate	GC/MS	20758	C08348	HMDB02545	2.51	0.0079	0.0287	0.7942	1.9930	100	100	Xenobiotics	Sugar, sugar substitute, starch	84740	91510-62-2;	1890	204
