# Supplementary material for: Molecular and metabolomic changes in the proximal colon of pigs infected with Trichuris suis
Source: Sci Rep. 2020 Jul 30;10:12853. doi: 10.1038/s41598-020-69462-5 (PMC7393168; doi:10.1038/s41598-020-69462-5)
Supplement: Supplementary file 3 — Supplementary Table S3. [file 41598_2020_69462_MOESM3_ESM.pdf]

Molecular and metabolomic changes in the proximal colon of pigs infected with *Trichuris suis*

Harry Dawson[1], Celine Chen[1], Robert Li[2], Lauren Nicki Bell[3], Terez Shea-Donohue[4], Helene Kringel[5], Ethiopia Beshah[1], Dolores E. Hill[2], Joseph F. Urban Jr[1,2].

[1]United States Department of Agriculture, Agricultural Research Service, Northeast Area, Beltsville Human Nutrition Research Center, Diet Genomics and Immunology Laboratory, [2] Beltsville Agricultural Research Center, Animal Parasitology Disease Laboratory, Beltsville, MD; [3]Metabolon, Inc., Morrisville, NC; [4]University of Maryland School of Medicine, Baltimore, MD, USA; [5]Department of Veterinary Disease Biology, Faculty of Health and Medical Sciences, University of Copenhagen, Copenhagen, Denmark

Supplemental Table S3

Supplemental Table S3. DEGs in the poximal colon of infected over control pigs at 21 days after inoculation.

Feature ID	Fold change	EDGE test: FDR p-value correction
PLA2G2A	-1633.2	1.33E-05
OLIG2	-143.0	1.09E-03
ZFP42	-89.1	3.67E-11
DNAH14	-60.4	1.56E-02
COL22A1	-56.8	1.34E-02
ADAM1A	-52.9	1.87E-02
ASTL	-49.0	2.75E-02
FABP3L2*	-44.6	1.84E-04
ABCC4L3	-23.7	8.32E-19
LIPM	-19.0	2.62E-02
ZSCAN9	-18.9	4.68E-02
CD40	-17.7	4.10E-02
MIR1279	-16.9	4.50E-05
SLC22A10L3	-14.0	1.75E-03
HOXC9	-12.3	5.27E-03
ABCC4L4	-12.2	7.35E-07
FCER2	-12.0	1.34E-02
AGTR2	-11.5	3.94E-03
ALDOB	-10.6	8.40E-19
CCL3L2	-10.6	5.90E-03
MIR1244-1	-9.7	6.08E-03
SLC30A10	-9.4	2.71E-13
OAZ3	-8.8	2.23E-02
SLC14A1	-7.9	9.93E-11
TRPV6	-7.7	1.24E-06
HMGCS2	-7.2	0.00E+00
CD200R1L	-6.9	7.81E-03
ABCA8	-6.8	4.42E-10
PHEX	-6.8	1.81E-02
ABCA6	-6.4	3.40E-08
CXCL9	-6.1	7.08E-08
CCR9	-5.8	1.14E-02
CXCL13	-5.8	5.77E-08
COL6A6	-5.7	3.51E-08
COL25A1	-5.6	7.28E-04
AGT	-5.6	1.62E-05
S100G	-5.6	3.09E-05
CCL3L1	-5.6	2.84E-03
ABCA9	-5.5	3.06E-08
MOGAT2	-5.4	8.78E-03
AICDA	-5.3	8.16E-04
CR2	-5.2	3.00E-14
FCRL1	-5.0	1.34E-02
SLC24A4	-4.9	8.53E-03
CYP26B1	-4.8	9.76E-03

MUSK	-4.8	5.12E-05
ZNF354B	-4.7	1.05E-02
PDK4	-4.6	5.99E-03
PLIN1	-4.3	4.50E-05
CD5L	-4.2	2.41E-02
GPR174	-4.1	4.81E-08
HAS3	-4.0	4.07E-05
TNFSF11	-4.0	1.83E-03
CYP1B1	-3.9	2.93E-04
IDO1	-3.8	6.52E-05
FCRLA	-3.8	3.49E-02
NR1H4	-3.7	1.44E-02
SLC7A3L7*	-3.6	4.06E-04
CLEC17A	-3.6	4.30E-03
ABCG2	-3.5	9.73E-07
SLC25A53	-3.5	2.19E-02
NXPE2	-3.4	2.01E-03
KLRK1	-3.4	6.23E-09
ABCC4L2	-3.4	2.05E-10
MAMDC2	-3.4	3.09E-05
GHR	-3.4	2.10E-05
ART3	-3.3	3.07E-02
PNLIPRP2	-3.3	7.77E-06
ADORA3	-3.3	5.03E-03
GCNT4	-3.3	6.50E-03
AQP8	-3.3	3.30E-04
XCL1	-3.3	3.63E-03
AGTR1	-3.2	6.35E-04
ABCA13	-3.2	3.49E-02
PF4	-3.2	1.71E-02
CD28	-3.2	1.08E-02
CLCA4	-3.1	1.16E-02
FBXO32	-3.1	9.94E-05
ABI3BP	-3.0	8.43E-06
DDIT4L	-3.0	2.84E-04
EDIL3	-3.0	3.93E-07
CR1	-3.0	1.86E-02
CHRM2	-3.0	7.15E-03
LY9L1*	-3.0	1.09E-02
SNORA32	-3.0	1.95E-02
KLF12	-2.9	1.06E-03
CHP2	-2.9	1.12E-05
PAX5	-2.8	2.30E-02
RPL11L	-2.8	7.05E-03
CCL8	-2.8	4.24E-02
ASPA	-2.8	1.01E-02
NGLY1	-2.8	4.52E-07

HDAC9	-2.7	7.28E-04
IL15	-2.7	3.09E-02
AR	-2.7	4.53E-02
ADAMTS19	-2.6	1.33E-02
LIFR	-2.6	3.03E-05
RHOH	-2.6	2.84E-03
HOXD10	-2.6	1.85E-02
NEGR1	-2.6	9.36E-03
ADGRE4	-2.6	1.56E-03
CTBS	-2.5	3.96E-02
CELF2	-2.5	4.04E-06
CD1D	-2.5	2.89E-03
GPNMB	-2.5	3.04E-05
BMP3	-2.5	2.94E-03
SLC26A3	-2.5	8.05E-03
PADI2	-2.5	1.60E-04
TNFSF13B	-2.5	2.69E-04
KCNJ2	-2.5	2.94E-04
NT5E	-2.5	1.78E-04
DAPK1	-2.5	8.50E-03
PRSS12	-2.5	5.62E-03
SLA-DQB1	-2.5	1.55E-03
BTC	-2.4	1.84E-02
LYPD6	-2.4	1.56E-02
SLA-DMB	-2.4	3.61E-04
PPFIA4	-2.4	2.68E-02
C4BPAL	-2.4	3.78E-02
IPCEF1	-2.3	2.46E-02
DMD	-2.3	4.69E-05
C3AR1	-2.3	5.27E-03
KIT	-2.3	2.04E-02
PDGFC	-2.3	2.70E-03
RPL35A	-2.3	1.95E-03
CXCL12	-2.3	5.03E-03
CNTN1	-2.3	2.41E-02
CST3	-2.3	3.93E-04
GPR82	-2.2	1.35E-02
AHRR	-2.2	2.98E-02
LPXN	-2.2	1.56E-03
SLC16A1	-2.2	1.59E-03
PTPN7	-2.2	1.83E-03
EGLN3	-2.2	1.65E-03
CD4	-2.2	1.61E-02
ADIPOQ	-2.2	4.06E-03
ADAMTS5	-2.2	1.84E-02
APH1B	-2.2	3.53E-02
TMTC1	-2.2	2.39E-02

SESN3	-2.2	5.41E-04
CHL1	-2.2	1.49E-02
SMAD9	-2.2	3.79E-04
ZNF672	-2.1	1.41E-02
TLR8	-2.1	1.41E-02
PGM5	-2.1	8.60E-04
DTNA	-2.1	2.37E-02
MPEG1	-2.1	7.99E-03
SLC8A1	-2.1	2.28E-03
CD302	-2.1	6.84E-03
CD3G	-2.1	8.80E-03
SLAMF6	-2.1	4.40E-02
HPSE	-2.1	4.45E-03
MAF	-2.1	1.40E-02
RARRES1	-2.0	2.81E-03
PIK3AP1	-2.0	6.93E-03
PLXNC1	-2.0	1.18E-03
CCL2	-2.0	1.38E-03
ZNF568	-2.0	1.33E-02
FAM65B	-2.0	2.68E-02
LRIG2	-2.0	5.03E-03
SDPR	-2.0	4.88E-03
SLC22A5	-2.0	4.83E-02
TLR7	-2.0	4.66E-02
RDH10	-2.0	2.25E-02
PCMTD2	-2.0	2.03E-02
CALD1	-2.0	5.64E-03
CYP4V2	-2.0	1.52E-02
COL6A5	-2.0	1.50E-02
SLC4A7	-2.0	2.41E-02
NPL	-1.9	2.41E-02
MAFB	-1.9	5.99E-03
ABCA1	-1.9	5.62E-03
C5	-1.9	1.14E-02
NFATC2	-1.9	4.03E-02
CD48	-1.9	1.09E-02
LRP12	-1.9	2.33E-02
ALDH6A1	-1.9	5.11E-03
SLC25A3	-1.9	2.19E-02
FGL2	-1.9	2.08E-02
DOCK10	-1.9	1.58E-02
SLA-DMA	-1.9	1.99E-02
NREP	-1.9	1.71E-02
SATB1	-1.9	1.64E-02
CFL2	-1.9	2.19E-02
CD2	-1.8	9.24E-03
BANK1	-1.8	4.81E-02

LY9	-1.8	4.79E-02
ERAP1	-1.8	3.18E-02
PPAP2B	-1.8	1.44E-02
RBMS1	-1.8	1.68E-02
FRZB	-1.8	3.93E-02
DIXDC1	-1.8	2.08E-02
IGJ	-1.8	1.92E-02
AHCYL2	-1.8	2.15E-02
IGSF5	-1.8	1.18E-02
SLA-3	-1.8	3.72E-02
ITGAE	-1.8	4.27E-02
PTCH1	-1.8	3.72E-02
ITGA4	-1.7	4.03E-02
SCARA5	-1.7	3.55E-02
CDC42EP3	-1.7	4.01E-02
PAG1	-1.7	4.72E-02
SYNE1	-1.7	3.52E-02
KCTD12	-1.7	4.66E-02
CRLF3	-1.7	4.24E-02
COL14A1	-1.7	3.72E-02
MPDZ	-1.7	3.53E-02
CYP7B1	-1.7	4.83E-02
CD109	-1.7	4.15E-02
TSPAN7	-1.7	4.78E-02
SEC11C	1.7	4.83E-02
MIF	1.7	4.03E-02
PLA2G16	1.8	4.03E-02
GLUL	1.8	4.41E-02
SLC35C1	1.8	4.89E-02
TARS	1.8	3.43E-02
MAZ	1.8	3.53E-02
S100A14	1.8	2.70E-02
SMARCE1	1.8	1.98E-02
SERPINB8	1.8	2.66E-02
FOXA1	1.8	3.01E-02
PIK3C2A	1.8	2.22E-02
PMPCA	1.8	3.50E-02
WDR1	1.9	4.04E-02
MIR1282	1.9	2.53E-02
CFL1	1.9	4.46E-02
IGFBP5	1.9	2.01E-02
MCF2L	1.9	1.98E-02
PRTFDC1	1.9	1.86E-02
NDUFA6	1.9	1.39E-02
DDX54	1.9	4.32E-02
MRPL51	1.9	1.72E-02
LSS	1.9	3.03E-02

LRRC59	1.9	3.60E-02
MKI67	1.9	1.58E-02
SUMO4	1.9	3.77E-02
STARD10	1.9	1.91E-02
BOLA2	1.9	2.95E-02
PSMB7	1.9	7.05E-03
SLC39A7	1.9	9.92E-03
MAGOH	1.9	1.71E-02
PTN	1.9	1.03E-02
CNOT3	2.0	3.50E-02
CENPJ	2.0	4.45E-02
SKA2	2.0	9.87E-03
ATOH1	2.0	2.41E-02
HSD11B1	2.0	2.19E-02
SMPDL3B	2.0	1.67E-02
FDPS	2.0	3.53E-02
SERPINB6	2.0	1.67E-02
TIMM17A	2.0	1.40E-02
SLC25A1	2.0	4.03E-02
CXCL16	2.0	2.89E-03
LRRC8A	2.0	1.64E-02
SEMA7A	2.1	2.41E-02
PSTPIP2	2.1	5.66E-03
GADD45B	2.1	4.75E-02
SMAGP	2.1	4.78E-02
ARHGDIA	2.1	1.87E-02
HDLBP	2.1	1.81E-02
UAP1L1	2.1	1.98E-02
ERN2	2.1	1.54E-02
DPP9	2.1	5.03E-03
TGM2	2.2	7.15E-03
SERPINB5	2.2	1.56E-03
PYCRL	2.2	4.03E-02
BCL3	2.2	2.84E-03
PKM	2.2	2.62E-02
DPRX	2.2	1.71E-02
TRAPPC6A	2.2	4.81E-02
TUBA1C	2.2	3.01E-02
TMEM54	2.2	1.11E-02
CDH1	2.2	3.28E-03
AREG	2.2	8.37E-03
ROS1	2.2	8.87E-04
RIPK3	2.3	1.33E-02
UBE2S	2.3	2.19E-02
CCNB2	2.3	3.45E-04
ADAMTS1	2.3	4.06E-04
B3GNT6	2.3	7.21E-03

ABCA12	2.3	4.40E-02
CABIN1	2.3	1.96E-03
FFAR4	2.3	2.68E-02
MYBL2	2.4	3.34E-03
P2RX4	2.4	8.14E-04
CYP2B22	2.4	4.65E-02
ND1	2.4	1.59E-03
LIPH	2.4	1.88E-04
TACC3	2.4	6.84E-03
DEPDC7	2.4	3.67E-02
GCNT3	2.4	4.06E-04
IL1A	2.4	1.55E-03
HMGA1	2.4	5.03E-03
FAM20C	2.4	2.79E-02
BGN	2.5	1.81E-02
HSPA1L	2.5	4.83E-02
PFKFB3	2.5	7.15E-03
GALNT6	2.5	5.55E-03
TNFRSF26	2.5	2.44E-02
NT5DC2	2.5	2.37E-02
CSRP2	2.5	1.20E-02
PTPRN2	2.5	3.72E-02
BIRC5	2.6	1.37E-03
KCNE3	2.6	3.51E-05
FGF7	2.6	5.67E-04
TUBB3	2.6	2.11E-03
ITGB6	2.6	7.63E-03
GSTA1	2.7	4.73E-02
CDX1	2.7	1.73E-03
PNP	2.7	1.83E-03
KCNJ11	2.7	3.31E-02
TPSB2	2.8	2.68E-02
RRBP1	2.8	8.06E-05
PHLDA2	2.8	2.79E-03
MDK	2.8	4.44E-04
FCN2	2.9	9.94E-05
CAPNS2	2.9	2.12E-03
PLA2G3	2.9	4.90E-03
SPDEF	3.0	1.13E-04
BPIFB2	3.0	3.18E-02
LAMB3	3.0	6.87E-04
ME1	3.1	9.19E-03
FUT2	3.1	1.38E-03
F3	3.1	4.09E-05
ETV4	3.1	4.03E-02
ABAT	3.2	1.73E-05
SLC7A5	3.3	1.30E-03

FABP3	3.3	3.51E-08
HK2	3.3	1.33E-05
STEAP4	3.4	5.33E-03
CLDN8	3.5	1.41E-02
CLCA1	3.5	9.80E-09
PRKCG	3.5	4.38E-02
SERPINB10	3.5	2.87E-04
DIO2	3.5	1.15E-03
DNAH8	3.7	3.59E-02
IGFBP4	3.7	6.25E-07
TNFSF18	3.8	3.17E-02
NRG1	3.8	2.41E-02
TIMP1	3.9	2.94E-04
LOC396756	4.0	1.51E-06
PTGS2	4.0	1.30E-07
TNFRSF8	4.0	2.04E-02
SLC9C1	4.1	7.81E-04
CHI3L2	4.2	4.97E-04
GNMT	4.3	2.10E-02
AKR1C4	4.3	2.30E-05
SLC7A11	4.3	6.75E-07
TLE6	4.4	1.34E-02
CXCL14	4.4	2.73E-06
PDZK1IP1L	4.5	1.44E-02
CXCL17	4.5	7.44E-06
TNIP3	4.5	5.90E-06
BAALC	4.5	3.96E-03
BDKRB1	4.6	1.70E-02
PI3	4.6	2.55E-08
KRT18	4.7	7.48E-10
SAPCD2	4.7	1.19E-03
PLAUR	4.8	6.11E-06
SLPI	4.8	2.22E-11
COL28A1	4.9	5.31E-06
MMP3	4.9	3.31E-10
PHGDH	5.0	9.01E-04
IL17C	5.0	7.15E-03
SLC5A8	5.0	1.13E-04
GSTA2	5.4	4.30E-03
BTN1A1L2*	5.4	2.54E-02
CA4	5.5	9.51E-10
CBS	5.5	7.18E-05
AGR2	5.6	1.73E-11
SDS	5.7	8.06E-05
MIR6087	6.0	1.14E-02
IL1R2	6.1	1.51E-06
MUC2	6.3	2.16E-16

CHI3L1	6.4	1.03E-13
B3GNT7	6.4	1.16E-05
UPK1B	6.5	1.59E-03
TAT	6.9	3.49E-02
GSTP1L2*	6.9	3.53E-02
RNF39	7.0	1.51E-06
IL36A	7.2	2.58E-03
MMP1	7.3	6.53E-13
TCN1	8.0	1.44E-10
SPINK4	8.1	9.43E-22
S100A8	8.2	1.02E-02
MPTX	8.3	4.03E-15
SERPINB11	8.4	7.38E-04
SLCO1A2	8.6	6.00E-04
SP6	9.3	1.01E-02
HCAR2	9.3	6.76E-05
MMP9	9.3	1.00E-02
ADAM30	9.6	8.58E-03
MMP12	9.7	4.45E-16
S100A9	9.8	1.83E-03
TGM3	10.0	3.03E-05
S100A2	10.0	3.03E-27
REG4	11.1	8.84E-20
IGHG1	11.1	3.80E-06
HTR1B	11.8	2.40E-02
GYS2	11.9	5.62E-03
S100A12	12.8	4.09E-05
PDZK1IP1*	13.6	1.24E-05
AKR1C1	13.8	3.93E-07
GABRP	15.7	1.59E-03
A2ML1	16.0	2.14E-03
SERPINB2	16.2	4.90E-32
SLC6A2	18.7	1.53E-02
PSAT1	20.6	4.69E-11
IL11	21.6	6.84E-03
TFF1	25.9	2.62E-06
MMP13	27.5	9.71E-29
ARG1	30.1	2.28E-07
IL36B	30.8	1.02E-02
ANXA8	34.8	6.97E-40
TFF2	37.1	6.93E-10
MMP7	53.1	4.09E-05
IFITM1L3*	63.2	1.59E-02
HOXB13	132.7	1.59E-02
SLC7A3L11*	188.2	1.59E-03
IL4I1L	437.7	1.59E-14
