# Supplementary material for: Molecular and metabolomic changes in the proximal colon of pigs infected with Trichuris suis
Source: Sci Rep. 2020 Jul 30;10:12853. doi: 10.1038/s41598-020-69462-5 (PMC7393168; doi:10.1038/s41598-020-69462-5)
Supplement: Supplementary file 4 — Supplementary Table S4. [file 41598_2020_69462_MOESM4_ESM.pdf]

Molecular and metabolomic changes in the proximal colon of pigs infected with *Trichuris suis*

Harry Dawson[1], Celine Chen[1], Robert Li[2], Lauren Nicki Bell[3], Terez Shea-Donohue[4], Helene Kringel[5], Ethiopia Beshah[1], Dolores E. Hill[2], Joseph F. Urban Jr[1,2].

[1]United States Department of Agriculture, Agricultural Research Service, Northeast Area, Beltsville Human Nutrition Research Center, Diet Genomics and Immunology Laboratory, [2] Beltsville Agricultural Research Center, Animal Parasitology Disease Laboratory, Beltsville, MD; [3]Metabolon, Inc., Morrisville, NC; [4]University of Maryland School of Medicine, Baltimore, MD, USA; [5]Department of Veterinary Disease Biology, Faculty of Health and Medical Sciences, University of Copenhagen, Copenhagen, Denmark

Supplemental Table S4

Supplemental Table S4. Comparison of pigs at 52 days after inoculation with no worms compared to control uninfected pigs

Feature ID	FC Naive vs Infection No Worms	EDGE test: FDR p-value correction
LCN15	-32.1	3.66E-07
ALPI	-4.9	5.20E-03
CD5L	-4.7	6.80E-03
PON1	-4.5	4.13E-02
SLC10A2	-4.3	4.13E-02
SDR9C7	-4.2	2.26E-02
CYP2B22	-3.9	2.23E-02
ACSL6	-3.7	9.52E-03
GSTA2	-3.6	5.23E-02
GDF5	-3.3	1.09E-03
SCNN1B	-3.3	2.26E-02
ALPIL1	-3.1	3.58E-02
GAL	-3.1	1.57E-02
PCK1	-3.1	1.86E-02
PDK4	-3.0	3.61E-02
NCR1	-2.9	2.48E-04
SLC24A1	-2.9	1.16E-03
RDH16	-2.8	4.89E-02
ACE2	-2.6	4.67E-02
SLC38A4	-2.6	5.46E-04
VGF	-2.6	2.96E-03
ANPEP	-2.5	4.13E-02
GPIHBP1	-2.5	3.06E-02
NCR2	-2.5	4.13E-02
TREM2	-2.5	4.89E-02
GPR162	-2.3	3.71E-02
NKPD1	-2.3	2.48E-03
NR1H4	-2.3	2.51E-02
EOMES	-2.2	3.65E-02
THNSL2	-2.2	2.04E-04
CIRBP	-2.1	6.57E-05
KLRD1L*	-2.1	2.36E-02
ANGPTL4	-2.0	4.70E-02
CA12	-2.0	3.03E-03
KLF11	-2.0	4.37E-02
SIRPB2	-2.0	3.09E-02
GSTM1	-1.9	9.10E-03
SMIM6	-1.9	4.13E-02
GSTM4	-1.8	5.07E-03
GSTT1	-1.8	4.13E-02
PYCARD	-1.8	1.54E-02
SLC24A4	-1.8	4.67E-02
SLC46A3	-1.8	2.05E-03
BDH1	-1.7	5.79E-03
CD8A	-1.7	5.31E-02
CES1B*	-1.7	7.07E-03
CLEC3B	-1.7	7.15E-03
H1F0	-1.7	6.99E-03
KCNE3	-1.7	1.16E-03
METTL7A	-1.7	3.83E-03
SLC16A14	-1.7	1.09E-02
THAP8	-1.7	4.86E-02
A1CF	-1.6	1.73E-02
ADGRE4	-1.6	2.26E-02
C1QB	-1.6	5.22E-02
GSTZ1	-1.6	1.03E-02
PDK2	-1.6	1.50E-02
PLCD1	-1.6	1.46E-02

PNPLA2	-1.6	1.01E-02
SLC43A2	-1.6	2.36E-02
TMEM140	-1.6	1.73E-02
UGT2C1*	-1.6	4.13E-02
COQ8A	-1.5	1.73E-02
ARRDC3	-1.5	4.39E-02
FDXR	-1.5	2.76E-02
HADHA	-1.5	1.16E-03
ODC1	-1.5	1.27E-02
SULT1B1	-1.5	3.48E-02
TSPAN7	-1.5	3.87E-02
SNX19	1.5	3.48E-02
RASA2	1.5	8.65E-03
SKIL	1.5	1.47E-02
SLC12A4	1.5	1.59E-03
EPAS1	1.5	1.29E-02
HSPA2	1.6	5.31E-02
COL12A1	1.6	4.13E-02
PRDM2	1.6	7.04E-03
GJA1	1.7	4.40E-02
APOBR	1.7	3.48E-02
SAMSN1	1.7	1.85E-02
PIK3C2A	1.7	5.31E-02
CD33	1.7	2.79E-02
AOAH	1.7	2.02E-02
PTGS1	1.7	6.80E-03
CDR2	1.7	1.47E-02
ANKRD12	1.7	2.76E-02
HYOU1	1.7	1.73E-02
NFIL3	1.7	1.50E-04
SERPINH1	1.8	4.70E-02
GRAP	1.8	2.00E-02
SLC26A2	1.8	5.24E-02
CHORDC1	1.8	8.64E-03
GPR68	1.8	6.99E-03
LPCAT2	1.9	2.33E-03
GPR65	1.9	3.09E-02
HSPA5	1.9	2.26E-02
ALOX5AP	2.0	3.09E-02
NOV	2.0	3.48E-02
TNFRSF12A	2.0	2.26E-02
DOCK2	2.0	7.80E-03
GATA2	2.0	5.31E-02
COL8A1	2.0	3.85E-02
SFRP1	2.1	2.80E-03
DNAJA1	2.1	5.20E-03
SLCO4A1	2.1	1.73E-02
F13A1	2.1	7.07E-03
CD69	2.1	4.85E-03
SRGN	2.1	1.18E-05
ARNTL2	2.1	3.38E-02
GFI1	2.2	1.03E-02
RUNX3	2.2	5.12E-03
IGHE	2.3	4.86E-02
KIT	2.3	1.73E-02
IPCEF1	2.4	1.03E-02
SLC16A3	2.4	2.50E-03
CD244	2.4	1.29E-02
NLRC3	2.5	1.97E-02
SELE	2.5	3.53E-02

MAP4K1	2.5	2.73E-04
GZMAL	2.5	1.01E-02
TDRP1	2.6	5.04E-03
HSPA1L	2.6	5.31E-02
SLC2A3	2.7	1.71E-10
HSPH1	2.7	1.29E-02
HDC	2.7	2.39E-02
GPR97	2.8	2.26E-02
FGR	2.8	6.51E-07
LTC4S	2.8	1.24E-02
TIGIT	2.8	3.39E-04
CSF2RAL	2.9	4.86E-02
GZMA	3.0	4.54E-04
CXCL6	3.1	1.01E-02
MMP9	3.4	3.03E-03
SH2D1B	3.5	4.91E-04
FFAR2	3.6	1.18E-05
TIAM1	4.4	6.67E-08
GATA3	4.4	4.43E-04
IGFBP2	4.7	1.99E-08
SLC5A8	4.8	3.38E-02
GPRC5D	4.8	9.86E-03
DFNB31	4.9	7.93E-05
DIO2	5.8	1.16E-03
NXPH4	5.8	2.70E-06
TKTL1	6.4	1.49E-02
SLC4A11	6.9	1.04E-03
CTSG	7.2	8.45E-07
MMP12	7.3	1.09E-03
KLRJ1*	9.6	1.59E-03
SNORA73AL	10.4	2.01E-02
NTRK1	10.6	3.53E-03
GZMB	11.2	8.54E-08
TCN1	19.2	1.29E-02
TNN	83.7	5.30E-05
GNLY	132.0	2.98E-11
REG3A	168.9	2.26E-02
