# Supplementary material for: Molecular and metabolomic changes in the proximal colon of pigs infected with Trichuris suis
Source: Sci Rep. 2020 Jul 30;10:12853. doi: 10.1038/s41598-020-69462-5 (PMC7393168; doi:10.1038/s41598-020-69462-5)
Supplement: Supplementary file 5 — Supplementary Table S5. [file 41598_2020_69462_MOESM5_ESM.pdf]

Molecular and metabolomic changes in the proximal colon of pigs infected with *Trichuris suis*

Harry Dawson[1], Celine Chen[1], Robert Li[2], Lauren Nicki Bell[3], Terez Shea-Donohue[4], Helene Kringel[5], Ethiopia Beshah[1], Dolores E. Hill[2], Joseph F. Urban Jr[1,2].

[1]United States Department of Agriculture, Agricultural Research Service, Northeast Area, Beltsville Human Nutrition Research Center, Diet Genomics and Immunology Laboratory, [2] Beltsville Agricultural Research Center, Animal Parasitology Disease Laboratory, Beltsville, MD; [3]Metabolon, Inc., Morrisville, NC; [4]University of Maryland School of Medicine, Baltimore, MD, USA; [5]Department of Veterinary Disease Biology, Faculty of Health and Medical Sciences, University of Copenhagen, Copenhagen, Denmark

Supplemental Table S5

Supplemental Table S5. Comparison of DEGs expressed in the proximal colon of pigs at 52 days after inoculation with worms compared to uninfected controls.

Gene	Fold change	EDGE FDR p-value
SLC10A2	-1285.8	8.30E-08
ALPI	-159.7	2.09E-10
EVX1	-38.1	2.47E-02
ASTL	-37.2	2.34E-02
CLCA4	-27.9	1.34E-07
ALDOB	-27.7	2.35E-07
GSTA2	-26.6	8.94E-06
CYP2B22	-26.4	2.38E-06
BTNL2	-21.7	9.02E-03
HMGCS2	-21.2	0.00E+00
NXPE2	-20.1	2.40E-06
NR1H4	-17.1	2.31E-11
PCK1	-16.9	1.62E-07
FRMD1	-15.5	9.59E-05
OASL	-14.4	5.68E-03
SLC14A1	-13.9	1.46E-02
FOXO6	-13.7	8.20E-06
AQP8	-13.7	2.47E-03
SLC26A3	-12.7	8.30E-08
NCR2	-12.1	1.81E-05
CD5L	-10.9	8.19E-04
THAP12	-10.5	4.27E-02
ALPIL1*	-10.3	1.67E-04
SLC30A10	-10.2	3.00E-03
SDR9C7	-9.3	8.99E-03
MOGAT2	-9.2	1.14E-03
AGT	-8.7	1.64E-05
PADI2	-8.6	1.42E-09
GRIK1	-8.6	4.27E-02
HAS3	-8.4	9.20E-06
SULT1E1	-8.2	1.08E-09
SI	-7.9	8.57E-03
SLC25A34	-7.8	1.41E-09
COL6A6	-7.8	1.07E-07
SLC38A4	-7.7	3.14E-09
PDK4	-7.7	2.11E-04
LCN15	-7.6	4.90E-02
ABCG2	-7.6	6.40E-07
CXCL9	-7.5	6.02E-04
CEACAM8	-7.5	1.20E-02
NCR1	-7.2	1.19E-06
SLC51A	-6.9	2.76E-08
P2RX3	-6.5	3.20E-05
ABCA6	-6.5	3.05E-06
GUCA2A	-6.3	2.26E-05
TM6SF2	-6.3	2.26E-14
CA12	-6.2	4.33E-13
DNAI1	-6.1	1.42E-02
RDH16	-6.1	3.20E-03
TRPV6	-6.0	4.34E-02
IFI27	-5.8	7.91E-04
SSTR5	-5.7	4.20E-03
SLC16A1	-5.7	0.00E+00
CA1	-5.6	4.33E-12
CCL3L2	-5.4	6.08E-03
GABRB2	-5.4	4.12E-06
GPIHBP1	-5.3	6.94E-04
ANPEP	-5.2	3.11E-04
XCL1	-5.2	1.96E-06
CYP26B1	-5.0	2.61E-08
IGF2-AS	-5.0	1.71E-02
OPRL1	-4.9	3.95E-04
PIPOX	-4.9	6.45E-03
EOMES	-4.8	1.12E-04
FOXJ1	-4.8	4.62E-03
CXCL11	-4.8	3.33E-02
IL12B	-4.7	1.02E-02
CLEC4F	-4.6	4.26E-02
DACT2	-4.6	4.67E-02
EDN3	-4.6	7.22E-03
SLC24A4	-4.4	2.68E-06
RSAD2	-4.4	2.06E-02

SELENBP1	-4.4	1.11E-11
ADIPOQ	-4.2	5.00E-04
CD207	-4.2	4.37E-02
KLF11	-4.2	3.47E-05
CHP2	-4.1	1.38E-07
UGT2C1*	-4.1	8.74E-11
DLX4	-4.1	2.69E-03
ABCA8	-4.1	3.01E-03
CLDN10	-4.1	3.30E-03
PLIN1	-4.0	3.06E-02
PARP15	-4.0	3.90E-04
GZMK	-4.0	1.31E-02
SCNN1B	-3.8	3.19E-02
GPR162	-3.8	1.31E-03
WNT8B	-3.8	2.30E-04
DLX3	-3.8	2.11E-02
GDF5	-3.8	1.25E-02
FOXS1	-3.8	3.26E-05
FABP1	-3.7	5.00E-05
KLRD1L	-3.7	1.90E-04
HSD17B6	-3.7	1.19E-02
CD36	-3.7	8.88E-09
CIDEA	-3.6	1.99E-03
CTBS	-3.6	8.38E-03
SLC25A42	-3.6	1.96E-06
RNF112	-3.5	1.01E-03
VIP	-3.5	5.68E-03
CNTFR	-3.5	4.40E-04
IRF4L	-3.4	5.23E-03
SIGLEC15	-3.4	5.06E-02
CXCL10	-3.4	3.22E-02
SECTM1	-3.4	1.23E-03
ZBP1	-3.4	5.56E-04
DHX58	-3.3	7.59E-03
SELENOP	-3.3	2.97E-08
ISX	-3.3	2.28E-03
IGF2	-3.2	5.66E-04
PRSS12	-3.2	1.43E-04
XAF1	-3.2	5.31E-02
ABI3BP	-3.2	4.50E-05
SLC30A3	-3.1	8.38E-03
LYPD6	-3.1	7.55E-03
CLCA4L	-3.1	1.92E-02
SLC7A8	-3.1	2.76E-05
NECAB2	-3.1	1.88E-02
GSTM1	-3.0	2.39E-05
AOC1	-3.0	3.91E-07
ABCA9	-3.0	3.15E-02
GAL	-3.0	4.97E-02
CCR9	-3.0	5.47E-02
ASPA	-2.9	4.20E-03
ANGPTL4	-2.9	1.19E-03
GSTM4	-2.9	3.46E-06
IFI44	-2.9	1.80E-02
PRDX6	-2.9	1.45E-07
SLC35D3	-2.9	3.46E-02
BDH1	-2.9	4.31E-08
TMPRSS13	-2.8	1.67E-02
CARD14	-2.8	6.03E-05
AHRR	-2.8	3.02E-04
FBXO32	-2.8	1.02E-04
SIRPB2	-2.8	6.49E-03
CD209	-2.8	1.42E-03
SLC46A3	-2.8	2.40E-06
ACSBG1	-2.8	4.00E-02
SLC7A3L6	-2.8	1.70E-02
CD4	-2.8	1.41E-04
CXCL12	-2.7	6.31E-04
NMRAL1L*	-2.7	1.81E-02
FAAH	-2.7	6.61E-04
UGT1A3	-2.7	2.57E-02
CA11	-2.7	7.55E-03
FABP4	-2.7	1.86E-02
NGB	-2.7	3.12E-02

ZFP84L	-2.7	3.53E-03
ABCB4	-2.7	2.10E-02
LRP1B	-2.7	1.12E-02
MAOA	-2.7	1.11E-07
LILRB3L4	-2.7	2.84E-02
MAMDC2	-2.7	2.50E-03
WNT11	-2.7	1.51E-02
GRIK4	-2.7	1.76E-02
SLC8A2	-2.7	2.11E-02
PTPRD	-2.6	1.41E-04
ACE	-2.6	7.25E-05
SLC7A3L4	-2.6	4.44E-03
TXNIP	-2.6	5.39E-05
GBP6	-2.6	5.25E-03
GSTT1	-2.6	1.25E-03
AURKC	-2.6	3.68E-03
UBE2L6	-2.6	1.15E-04
P2RY13	-2.6	6.61E-04
ADGRB1	-2.6	1.64E-02
IFITM1	-2.6	1.45E-02
C1QB	-2.6	4.13E-05
C11orf86	-2.5	4.92E-04
SULT1C4	-2.5	1.40E-04
CD74	-2.5	2.92E-04
ZKSCAN2	-2.5	4.12E-02
KLRC1	-2.5	2.35E-02
CIRBP	-2.5	3.91E-05
IL34	-2.5	3.63E-04
ADGRE4	-2.4	9.29E-06
GBP2	-2.4	1.02E-02
AHCYL2	-2.4	5.80E-06
SLC7A3L7	-2.4	3.11E-02
SCARA5	-2.4	2.08E-02
DBP	-2.4	3.71E-03
PLCD1	-2.4	1.77E-06
GBP4	-2.4	4.83E-02
COL4A3	-2.4	4.44E-02
SLA-DRA	-2.4	1.06E-03
ADGB	-2.4	3.63E-02
TMPRSS2	-2.4	1.23E-04
SLA-DOA	-2.4	3.71E-03
TMEM140	-2.4	2.01E-06
CCL4	-2.4	1.16E-02
APOL2	-2.4	1.75E-04
RBP5	-2.4	5.79E-03
PNPLA2	-2.4	1.05E-06
ANK3	-2.4	8.67E-03
TLR8	-2.3	1.46E-03
C1QA	-2.3	4.91E-05
ANKH	-2.3	7.37E-03
C1QC	-2.3	5.39E-05
SLC39A5	-2.3	3.63E-02
IL12RB2	-2.3	4.34E-02
TUBAL3	-2.3	3.93E-02
CD83	-2.3	1.40E-02
IHH	-2.3	3.41E-05
THNSL2	-2.3	1.15E-03
HAPLN4	-2.3	1.17E-04
SLA-3	-2.3	1.89E-04
ADRB1	-2.3	1.40E-03
VGF	-2.3	3.28E-02
IFITM1L2*	-2.3	6.10E-05
SLC46A2	-2.3	6.48E-03
HOXD10	-2.3	8.74E-03
IFI44L	-2.3	3.75E-03
SLA-11	-2.3	1.08E-04
PCSK6	-2.3	1.07E-05
CD300H	-2.3	1.10E-05
LOC106509843	-2.3	1.93E-02
SLA-DQA1	-2.3	3.27E-03
SLA-DQB1	-2.3	1.13E-02
CD36L1	-2.3	2.24E-03
ABCC6	-2.2	4.92E-02
MAF	-2.2	1.08E-04

RETSAT	-2.2	4.10E-06
FCGR3A	-2.2	8.60E-05
LGALS9	-2.2	2.92E-04
SLA-DMB	-2.2	8.10E-04
CD8A	-2.2	4.54E-03
ZNF419	-2.2	3.82E-02
CD300C	-2.2	5.26E-03
LTBP4	-2.2	9.11E-05
CD27	-2.2	4.87E-02
PTPRO	-2.2	3.59E-02
EDIL3	-2.2	2.93E-03
CD7	-2.2	4.61E-02
ABCB1	-2.2	2.05E-03
PARP9	-2.2	1.66E-04
SLA-DRB1	-2.2	5.60E-03
PTCH1	-2.2	1.80E-03
EFNA3	-2.2	2.20E-02
TSPAN7	-2.2	5.07E-06
TST	-2.2	3.80E-05
TLR1	-2.2	8.67E-04
CXCR3	-2.2	1.38E-02
CYP7B1	-2.2	9.83E-03
COQ8A	-2.2	9.79E-06
MPEG1	-2.2	4.93E-03
KLHL7	-2.1	2.73E-04
NT5E	-2.1	1.30E-02
CES1B	-2.1	5.66E-04
P2RY12	-2.1	5.25E-02
ACSF2	-2.1	6.81E-06
NKPD1	-2.1	4.23E-02
LY9L1*	-2.1	4.13E-03
CYP2C91	-2.1	5.29E-02
CDKN2B	-2.1	1.87E-04
H1F0	-2.1	7.02E-04
LOC110255453	-2.1	4.34E-02
FOXH1	-2.1	3.28E-02
ADAMTSL5	-2.1	3.76E-02
CST3	-2.1	1.99E-03
ABCB6	-2.1	2.65E-05
SLC16A5	-2.1	1.59E-03
PARP14	-2.1	1.16E-02
LTB4R2	-2.1	3.08E-02
CASP1	-2.1	9.13E-04
LIFR	-2.1	4.05E-04
SLC25A20	-2.1	1.13E-04
SLC25A27	-2.1	1.55E-05
METTL7A	-2.1	1.05E-04
SLA-DMA	-2.1	4.61E-03
CTSD	-2.1	3.64E-04
ZNF548	-2.1	1.71E-02
TMPRSS4	-2.1	4.56E-05
PLCB2	-2.0	1.74E-03
MLYCD	-2.0	1.50E-03
CECR1	-2.0	4.20E-03
CSMD1	-2.0	3.76E-02
AMT	-2.0	3.33E-04
GFRA1	-2.0	4.61E-02
CPT1A	-2.0	2.94E-03
KLRG1	-2.0	5.07E-02
WNT2B	-2.0	3.42E-03
PFKFB4	-2.0	4.11E-05
COBL	-2.0	2.96E-06
EXOC3L4	-2.0	4.81E-04
PKNOX2	-2.0	2.25E-02
MMP11	-2.0	7.40E-04
LAIR1	-2.0	7.37E-03
TLR5	-2.0	4.03E-02
CDH23	-2.0	4.71E-02
B2M	-2.0	5.66E-04
MDK	-2.0	1.75E-02
ABCC4L2	-2.0	8.20E-03
UGT1A1	-2.0	1.54E-02
NADK	-2.0	5.86E-05
TLR6	-2.0	4.77E-03

EBI3	-2.0	4.52E-02
FBXW10	-2.0	2.07E-02
GBP1	-1.9	5.21E-02
SLC9A2	-1.9	1.78E-04
LGALS3BP	-1.9	3.18E-02
TSPAN11	-1.9	4.35E-03
SLC25A38	-1.9	4.57E-04
RGS10	-1.9	2.55E-02
OSBPL10	-1.9	6.89E-03
ZNF664	-1.9	6.81E-05
TAP1	-1.9	5.73E-03
CSTA	-1.9	2.56E-02
PYCARD	-1.9	8.72E-03
NCAM1	-1.9	3.06E-02
TCF7L1	-1.9	2.24E-02
BSG	-1.9	1.92E-04
ADHFE1	-1.9	4.54E-02
PIK3IP1	-1.9	1.62E-03
PGF	-1.9	4.93E-02
THAP8	-1.9	4.60E-02
ITIH5	-1.9	1.46E-02
IL10RA	-1.9	8.53E-04
CLEC3B	-1.9	2.18E-03
GLI2	-1.9	3.33E-02
ERBB2	-1.9	1.33E-03
SDPR	-1.9	1.59E-02
MT-CO3	-1.9	4.64E-03
SPIDR	-1.9	2.85E-04
OGDH	-1.9	8.27E-05
NOXO1	-1.9	1.71E-03
PDK2	-1.9	1.11E-03
TRANK1	-1.9	1.91E-02
TEF	-1.9	3.52E-03
SLC25A29	-1.9	1.01E-02
GIMAP8	-1.9	1.74E-03
CD302	-1.9	9.28E-03
AKR1E2	-1.9	3.20E-03
C7	-1.9	3.94E-02
ABCC4L5	-1.8	2.09E-02
SLC31A2	-1.8	2.06E-02
USH1C	-1.8	4.24E-04
CTSZ	-1.8	4.19E-03
SYNPO	-1.8	7.65E-03
MMP24	-1.8	2.06E-02
PSMB9	-1.8	9.65E-03
FGFRL1	-1.8	8.75E-03
MAP2K6	-1.8	3.77E-02
SLC45A4	-1.8	1.06E-03
GRINA	-1.8	1.50E-03
SLC43A2	-1.8	2.00E-03
RGL1	-1.8	1.84E-03
DAPK1	-1.8	2.09E-02
MEIS2	-1.8	4.81E-02
CD300LF	-1.8	5.14E-02
MTCP1	-1.8	4.83E-02
RNF213	-1.8	4.61E-02
ZNF709L5*	-1.8	4.65E-02
UCP2	-1.8	7.55E-03
SNX4	-1.8	7.26E-04
DTX3L	-1.8	7.69E-03
PHACTR1	-1.8	6.57E-03
HSD17B1	-1.8	4.04E-02
CLCN2	-1.8	4.79E-05
CCR5	-1.8	5.37E-03
CYP27A1	-1.8	3.99E-02
PAX8	-1.8	3.67E-02
MOCS1	-1.8	6.89E-03
CADM1	-1.8	3.02E-02
TRPV3	-1.8	1.05E-02
ADA	-1.8	1.88E-02
RALGDS	-1.8	2.82E-03
TNFAIP2	-1.8	2.30E-04
TESK2	-1.8	5.66E-03
ASAP3	-1.8	3.44E-03

SULT1B1	-1.8	1.20E-03
PSMB10	-1.8	3.20E-03
SOWAHA	-1.8	2.63E-03
SLC25A10	-1.8	2.22E-04
MMP25	-1.8	1.15E-02
IRF9	-1.8	5.24E-03
CD72	-1.8	4.52E-02
EGFLAM	-1.8	2.10E-02
VAMP2	-1.8	1.77E-03
GABBR1	-1.8	2.17E-03
FLT3LG	-1.8	3.71E-03
ADCY9	-1.8	1.41E-04
MTHFR	-1.8	7.98E-03
GPR34	-1.8	2.92E-02
GSTP1	-1.8	7.59E-03
TCF21	-1.8	4.67E-03
SLC7A3L2	-1.8	4.24E-04
SCIN	-1.8	3.67E-02
GABARAPL1	-1.8	6.54E-04
ADCY5	-1.8	4.38E-02
ACY1	-1.7	9.49E-03
PRAG1	-1.7	1.02E-02
ALDH6A1	-1.7	1.15E-02
DOCK6	-1.7	4.13E-03
MT-ATP6	-1.7	1.36E-02
TSKU	-1.7	3.98E-02
HADHA	-1.7	1.84E-04
ENO2	-1.7	3.86E-02
NPL	-1.7	2.11E-02
HSDL2	-1.7	7.04E-04
JAML	-1.7	2.72E-02
PLTP	-1.7	1.75E-02
MISP	-1.7	2.06E-03
UGT1A10	-1.7	6.00E-04
SLC7A3L8	-1.7	1.79E-02
MOV10	-1.7	1.22E-03
MERTK	-1.7	7.59E-03
N4BP2L1	-1.7	5.30E-02
AXIN2	-1.7	3.42E-02
TICAM1	-1.7	1.41E-03
CSF1R	-1.7	3.70E-03
CD68L1	-1.7	1.16E-02
MT-CO1	-1.7	7.42E-03
SLC27A1	-1.7	3.17E-02
PLA2G6	-1.7	9.77E-03
LCP2	-1.7	7.46E-03
CCL21	-1.7	4.86E-02
VDR	-1.7	7.90E-03
ACADS	-1.7	2.96E-03
APOE	-1.7	3.18E-02
LIPE	-1.7	3.48E-02
PARP3	-1.7	3.15E-02
ZNF362	-1.7	7.88E-03
MICALCL	-1.7	2.78E-02
DTX3	-1.7	4.85E-03
RFTN2	-1.7	1.70E-02
CRIP1	-1.7	2.87E-02
TSPAN4	-1.7	3.42E-02
NREP	-1.7	4.99E-02
NADSYN1	-1.7	7.62E-03
HPSE	-1.6	1.75E-02
LRIG2	-1.6	5.77E-03
DISP1	-1.6	4.68E-03
ATG2A	-1.6	4.13E-03
SPPL2B	-1.6	7.40E-04
ETHE1	-1.6	1.38E-03
ERAP2	-1.6	4.60E-02
DECR1	-1.6	1.78E-03
PSMB8	-1.6	2.91E-02
PLA2G4B	-1.6	3.68E-02
ACADSB	-1.6	1.66E-03
UGDH	-1.6	2.77E-03
HOXA11	-1.6	5.12E-02
TAP2	-1.6	1.31E-02

HGSNAT	-1.6	1.40E-02
ZNF358	-1.6	1.34E-02
COL4A6	-1.6	3.19E-02
PPARD	-1.6	4.85E-03
NAAA	-1.6	3.53E-02
LAPTM5	-1.6	3.96E-02
PNPLA7	-1.6	4.92E-03
NATD1	-1.6	4.27E-02
SLC44A4	-1.6	3.27E-03
GIMAP5	-1.6	4.42E-02
ACAD10	-1.6	1.02E-02
THRA	-1.6	2.58E-02
HS1BP3	-1.6	1.12E-02
ANKRD13A	-1.6	3.64E-03
HDAC10	-1.6	1.78E-03
IRF8	-1.6	1.94E-02
NR3C2	-1.6	2.11E-02
CDX2	-1.6	1.15E-02
TYROBP	-1.6	3.74E-02
PSME1	-1.6	5.26E-03
BCAS3	-1.6	2.27E-02
INPP5K	-1.6	7.22E-03
GMPR	-1.6	6.59E-03
LTBP3	-1.6	4.59E-02
CYB5RL	-1.6	5.37E-02
ATG4A	-1.6	1.45E-02
KLC4	-1.6	4.83E-03
CD44	-1.6	2.09E-02
ERBB3	-1.6	4.43E-02
IVD	-1.6	2.32E-02
DHRS11	-1.6	4.77E-03
CYBB	-1.6	3.34E-02
RARRES2	-1.6	4.83E-02
PDXK	-1.6	9.56E-03
SLC6A6	-1.6	5.12E-02
SCARB2	-1.6	1.71E-03
HOXA5	-1.6	1.74E-02
FBP2	-1.6	1.05E-02
CERS6	-1.6	3.04E-02
SLA-8	-1.6	1.98E-02
LRP4	-1.6	2.19E-02
GALNS	-1.6	1.45E-02
PNPLA6	-1.6	2.08E-02
ASCC2	-1.6	6.52E-04
FTL	-1.6	4.64E-02
MYH14	-1.6	1.75E-02
MNT	-1.6	1.51E-02
ACAD11	-1.6	1.82E-02
NOP53	-1.6	9.95E-03
VARS2	-1.5	1.93E-02
MT-CO2	-1.5	5.08E-02
THSD4	-1.5	2.95E-02
MAP1LC3A	-1.5	1.88E-02
TRAP1	-1.5	1.76E-02
SYNE1	-1.5	4.67E-02
VAV3	-1.5	5.02E-02
SLC9A3R1	-1.5	1.36E-02
FCER1G	-1.5	5.31E-02
CUEDC2	-1.5	2.10E-02
LIPA	-1.5	3.68E-02
TMEM94	-1.5	4.35E-03
MUL1	-1.5	1.04E-02
VSIR	-1.5	1.46E-02
NAT6	-1.5	5.14E-02
VIL1	-1.5	2.16E-02
PCBP4	-1.5	4.08E-02
CNNM4	-1.5	1.45E-02
TRIM66	-1.5	2.93E-02
TOM1L2	-1.5	3.51E-02
BBS4	-1.5	1.94E-02
CSF1	-1.5	3.58E-02
NT5C3A	-1.5	4.41E-02
ACVR1B	-1.5	1.73E-02
DIRC2	-1.5	4.91E-02

SCARF1	-1.5	4.78E-02
UVRAG	-1.5	1.97E-02
CTDSP2	-1.5	2.09E-02
IL17RA	-1.5	1.36E-02
SLC25A6	-1.5	2.26E-02
UNC93B1	-1.5	4.49E-02
BCL9L	-1.5	4.61E-02
NAV1	-1.5	3.87E-02
CLEC2D	-1.5	5.17E-02
DENND6B	-1.5	5.10E-02
PCGF2	-1.5	4.54E-02
SESN1	-1.5	2.85E-02
SLC25A5	-1.5	2.24E-02
BFAR	-1.5	1.98E-02
PSEN1	-1.5	1.41E-02
BCL2L14	-1.5	2.85E-02
TCN2	-1.5	3.88E-02
CAT	-1.5	1.33E-02
FCGRT	-1.5	2.30E-02
SLC13A1	-1.5	4.47E-02
SLC12A7	-1.5	2.28E-02
PPARA	-1.5	4.14E-02
MAFB	-1.5	3.08E-02
SP110	-1.5	5.40E-02
LENG8	-1.5	4.23E-02
ACOX1	-1.5	1.80E-02
ASPH	1.5	1.62E-02
HM13	1.5	4.05E-02
ZNF639	1.5	4.39E-02
TOR1AIP1	1.5	9.73E-03
MCF2L	1.5	4.37E-02
SLC35B1	1.5	2.46E-02
RNF115	1.5	3.78E-02
VKORC1L1	1.5	1.66E-02
ROCK1	1.5	3.08E-02
SLC39A11	1.5	5.49E-02
NUP153	1.5	2.91E-02
MAPK8	1.5	3.25E-02
MAP2K1	1.5	9.07E-03
LRRC8A	1.5	5.45E-02
UBE2N	1.5	3.17E-02
EIF5	1.5	1.51E-02
ACSL4	1.5	3.29E-02
MTHFD1	1.5	1.94E-02
FILIP1L	1.5	5.11E-02
ANP32E	1.5	2.39E-02
PTP4A1	1.5	4.70E-02
RASA2	1.5	1.67E-02
FAT1	1.5	4.34E-02
SUGT1	1.5	3.17E-02
UBA2	1.5	1.35E-02
SLC37A3	1.5	7.96E-03
TSPAN6	1.5	2.95E-02
YARS	1.5	4.20E-03
ITGB1	1.5	1.80E-02
SMAD1	1.5	1.98E-02
PSMD1	1.5	3.59E-02
HMGN3	1.5	2.23E-02
TNPO1	1.5	3.57E-02
ZW10	1.5	1.38E-02
PTPN11	1.5	1.93E-02
RAPGEF2	1.5	7.74E-03
DDX21	1.5	2.23E-02
ACKR3	1.5	4.95E-02
CEP350	1.5	2.57E-02
SEC16A	1.5	1.69E-02
LTBP1	1.5	2.73E-02
KPNB1	1.5	1.97E-02
MESDC1	1.5	1.49E-02
WDR36	1.5	2.03E-02
P2RX4	1.5	4.22E-02
MAGT1	1.5	2.43E-02
PDE4B	1.5	4.82E-02
DDX18	1.5	2.76E-02

CLTC	1.5	3.32E-02
SWAP70	1.5	2.99E-02
UBR1	1.5	1.69E-02
ARID3A	1.5	1.18E-02
NOP58	1.5	4.61E-02
PBRM1	1.5	3.12E-02
SRPR	1.5	1.16E-02
ZNF827	1.5	3.66E-02
STARD10	1.5	3.63E-02
TACC3	1.5	5.11E-02
MSH2	1.5	3.64E-02
SLC12A4	1.5	4.15E-03
SRSF10	1.5	4.44E-03
MOGS	1.5	5.43E-03
CSE1L	1.5	5.73E-03
QSER1	1.5	3.74E-02
SMC1A	1.5	3.99E-02
ASCC3	1.5	5.12E-02
SLC35C1	1.5	4.14E-02
PPIA	1.5	1.88E-02
DSC2	1.5	4.78E-02
GALNT1	1.5	4.20E-02
MAL2	1.5	1.07E-02
RAN	1.5	1.47E-02
MANSC1	1.5	2.58E-02
RACGAP1	1.5	5.25E-02
CAD	1.5	1.90E-02
HSPA4	1.5	1.23E-02
ZBTB1	1.5	3.22E-02
STARD5	1.5	4.88E-02
SELENOI	1.5	5.04E-03
CCP110	1.5	2.09E-02
TVP23B	1.5	3.30E-03
TMED2	1.5	1.25E-02
TRIP11	1.5	1.75E-02
LAMC1	1.6	2.40E-02
DDX3X	1.6	5.28E-02
ACACA	1.6	4.83E-03
CANX	1.6	2.07E-02
SEC23B	1.6	1.24E-02
BZW1	1.6	3.18E-02
IDE	1.6	7.53E-03
SELENOF	1.6	1.38E-02
SURF4	1.6	1.38E-02
APEX1	1.6	3.94E-03
GOLGA4	1.6	1.42E-02
PRDM1	1.6	2.16E-02
TOP2B	1.6	1.38E-02
NUP205	1.6	2.04E-02
BTAF1	1.6	1.43E-02
EIF2AK3	1.6	3.01E-03
ANXA2	1.6	1.42E-02
BAZ1A	1.6	7.89E-03
PDPN	1.6	4.98E-02
ATF4	1.6	1.20E-02
AARS	1.6	6.94E-03
SACS	1.6	2.04E-02
SEC61A1	1.6	9.70E-03
SEL1L3	1.6	2.74E-02
ZNF281	1.6	3.22E-03
STK39	1.6	3.52E-03
FZD6	1.6	5.05E-02
CLIC2	1.6	2.84E-02
FUT8	1.6	5.97E-03
AOAH	1.6	5.28E-02
ENTPD6	1.6	1.65E-02
WISP1	1.6	5.23E-02
SEL1L	1.6	4.20E-03
SLC39A10	1.6	5.43E-02
CASP3	1.6	2.55E-03
PAWR	1.6	2.76E-04
SERPINB1	1.6	3.13E-02
ADAMTS7	1.6	3.54E-02
ERLEC1	1.6	2.58E-02

C9	1.6	3.93E-02
ALDOC	1.6	5.08E-02
PPRC1	1.6	1.77E-02
MIS18BP1	1.6	3.18E-02
GOLGA3	1.6	2.64E-03
IQGAP1	1.6	6.60E-03
ZMYND19	1.6	3.44E-02
TUBA1B	1.6	6.88E-03
DOCK7	1.6	1.37E-02
EPRS	1.6	3.45E-03
CMAS	1.6	1.54E-02
SLC7A1	1.6	1.25E-02
FLVCR2	1.6	2.26E-02
PGM2	1.6	7.93E-03
VLDLR	1.6	1.87E-03
COL5A2	1.6	1.79E-02
S100A6	1.6	3.06E-02
AP3S1	1.7	1.36E-03
PTPN12	1.7	3.05E-04
CCDC134	1.7	4.17E-02
FOCAD	1.7	3.14E-03
ABCE1	1.7	3.69E-03
NAA15	1.7	1.71E-02
PRKAA2	1.7	3.44E-02
CARS	1.7	1.99E-03
ARG2	1.7	3.31E-02
SRP54	1.7	5.23E-03
SELENOK	1.7	1.33E-03
EIF4E	1.7	8.21E-03
PLA2G4A	1.7	3.82E-02
LTBP2	1.7	2.28E-02
ARCN1	1.7	2.11E-03
COPB2	1.7	6.68E-03
MAPK6	1.7	1.13E-02
UBE2S	1.7	1.08E-02
P4HA2	1.7	5.38E-03
CTNNB1	1.7	3.08E-03
ITGAV	1.7	7.96E-03
CDCA8	1.7	2.23E-02
ATM	1.7	1.39E-03
AMIGO3	1.7	2.55E-03
TOPBP1	1.7	3.75E-03
HSPD1	1.7	1.25E-02
SLC20A1	1.7	1.63E-02
GMPPB	1.7	8.21E-03
SOX9	1.7	1.52E-02
DNAJC25	1.7	1.46E-02
SLC30A7	1.7	2.93E-03
AMD1	1.7	3.24E-03
BRCA2	1.7	3.63E-02
THY1	1.7	1.92E-02
SOAT1	1.7	1.57E-02
BDP1	1.7	2.88E-03
UFM1	1.7	5.37E-03
MAP4K4	1.7	5.24E-04
ADAMTS9	1.7	4.78E-02
CASC4	1.7	1.90E-03
RPS6KA3	1.7	1.07E-03
MAN2A1	1.7	7.59E-03
SLC2A1	1.7	7.88E-03
TFPI2	1.7	5.42E-03
SLC7A6	1.7	2.15E-03
ATR	1.7	2.92E-02
RPN2	1.7	7.02E-03
CFI	1.7	4.15E-02
SLC39A7	1.7	1.29E-03
CBX5	1.7	9.86E-04
F2RL2	1.7	1.57E-02
PTGER2	1.7	1.84E-03
HMMR	1.7	4.64E-02
COPB1	1.7	7.69E-03
COL4A1	1.7	1.53E-02
BUB1	1.7	5.12E-02
E2F7	1.7	5.49E-02

C3	1.7	3.10E-02
TMED5	1.7	4.92E-04
SPATS2	1.7	8.34E-04
SAMSN1	1.7	1.38E-02
TNFRSF11A	1.7	3.61E-02
GALNT6	1.7	1.20E-02
DUSP5	1.7	1.26E-02
SPC24	1.7	5.41E-02
BTG2	1.7	9.85E-03
TMED10	1.7	1.41E-04
GRAP	1.7	2.12E-02
TOP2A	1.7	2.46E-02
NSDHL	1.7	4.11E-03
COL6A3	1.8	3.31E-02
CHORDC1	1.8	1.47E-02
ADM	1.8	2.21E-02
OSBPL8	1.8	5.05E-02
SKA2	1.8	1.91E-02
GARS	1.8	7.19E-04
TIFA	1.8	7.65E-03
STEAP2	1.8	2.56E-02
ECSCR	1.8	2.28E-02
SLC38A2	1.8	2.90E-02
NUDCD1	1.8	6.46E-03
SSR1	1.8	8.41E-04
TPX2	1.8	1.13E-02
MTHFD2	1.8	2.97E-03
TMED7	1.8	7.76E-04
SC5D	1.8	3.28E-03
MMP19	1.8	4.54E-02
CCT6A	1.8	4.24E-04
POLE	1.8	9.67E-03
CCND2	1.8	5.04E-03
CHEK2	1.8	3.88E-03
TNFSF15	1.8	2.54E-02
MIS18A	1.8	5.06E-02
SRGN	1.8	2.61E-03
MCTP2	1.8	1.96E-03
ERN2	1.8	1.38E-02
FGR	1.8	2.10E-02
PGK1	1.8	1.24E-03
MARS	1.8	4.34E-05
SLC16A10	1.8	3.61E-02
SLC3A2	1.8	1.83E-03
KLF15	1.8	3.84E-02
ACOT11	1.8	5.31E-02
CD24	1.8	4.34E-02
FAM20C	1.8	4.61E-03
KRT80	1.8	4.15E-02
TNFRSF10B	1.8	3.08E-03
ARF2	1.8	2.80E-03
SLC30A5	1.8	1.03E-03
TSPAN12	1.8	3.52E-04
THBD	1.8	2.09E-02
INTS2	1.8	3.06E-03
SEC61G	1.8	4.85E-03
DNA2	1.8	2.59E-02
GADD45A	1.8	2.23E-02
CD69	1.8	3.37E-02
ARF4	1.8	1.02E-03
SERP1	1.8	4.79E-04
SLC1A5	1.8	8.53E-04
IGJ	1.8	2.63E-02
TSPAN13	1.8	1.66E-03
IFRD1	1.8	2.25E-03
CCDC88B	1.8	1.78E-03
SLC6A9	1.8	1.29E-02
WFS1	1.9	1.20E-03
KIF5B	1.9	3.90E-03
GNE	1.9	2.36E-03
YARS2	1.9	2.28E-02
DHCR7	1.9	3.19E-04
PFKFB2	1.9	3.58E-02
SELENOM	1.9	2.48E-03

FDFT1	1.9	3.52E-04
HMGCR	1.9	1.25E-03
STT3A	1.9	1.19E-03
SULF2	1.9	2.64E-02
CALR	1.9	2.48E-03
ETV5	1.9	2.26E-02
CDC25C	1.9	6.70E-03
HSP90AA1	1.9	2.84E-02
METTL16	1.9	2.84E-02
PPIB	1.9	7.19E-04
PANK1	1.9	9.77E-03
CXCR4	1.9	1.45E-02
SLC23A1	1.9	3.44E-03
HSPA8	1.9	3.54E-02
ITGA1	1.9	3.71E-02
BLM	1.9	1.56E-02
SKIL	1.9	3.19E-05
SERPINB5	1.9	8.72E-03
DNAJC10	1.9	4.24E-04
SPRED1	1.9	3.24E-05
SLC7A2	1.9	4.15E-02
CD163	1.9	4.34E-02
DSE	1.9	4.61E-03
EDEM3	1.9	1.53E-02
LRRC59	1.9	1.99E-03
ADGRG6	1.9	2.65E-03
MIA3	1.9	1.23E-04
PKM	1.9	3.11E-04
ECM1	1.9	4.06E-03
HELLS	1.9	4.19E-03
GALNT7	1.9	5.18E-04
MVD	1.9	2.48E-04
RRM2	1.9	2.76E-04
MCM6	1.9	3.68E-04
FNDC3B	1.9	4.00E-03
SHMT2	2.0	2.65E-05
FOXA1	2.0	6.53E-03
SREBF1	2.0	1.67E-04
ECT2	2.0	1.69E-02
SERPINH1	2.0	7.17E-03
ST7	2.0	4.61E-03
MMD	2.0	9.89E-04
EDEM1	2.0	5.13E-05
ABCA5	2.0	4.93E-03
WNT5A	2.0	2.03E-02
OSMR	2.0	1.41E-04
XBP1	2.0	2.73E-04
IL17RD	2.0	2.03E-02
DDIT4	2.0	1.40E-04
ATOX1	2.0	3.87E-03
AACS	2.0	4.99E-05
RIPK3	2.0	5.75E-04
ACSL3	2.0	8.07E-05
BST1	2.0	9.80E-04
PLSCR4	2.0	4.20E-03
ASNSD1	2.0	1.71E-03
KIF23	2.0	5.38E-03
IL33	2.0	9.72E-04
PCK2	2.0	1.77E-03
CYP51A1	2.1	1.08E-04
TARS	2.1	7.29E-05
SLC35A3	2.1	3.51E-03
WLS	2.1	7.12E-04
CEBPB	2.1	3.29E-03
CFB	2.1	1.92E-02
WNT9B	2.1	3.98E-02
SEC24D	2.1	2.38E-06
DNAJA1	2.1	4.93E-03
SEC11C	2.1	2.82E-04
ANKRD12	2.1	1.18E-03
RRBP1	2.1	1.96E-04
COL12A1	2.1	1.41E-04
MEST	2.1	4.67E-02
TMED3	2.1	3.45E-03

AFF2	2.1	2.84E-02
PGM3	2.1	7.37E-05
GPR19	2.1	2.11E-02
COL7A1	2.1	1.26E-03
ADGRE2	2.1	2.00E-02
ANXA5	2.1	1.44E-05
CDH1	2.1	1.78E-04
PROCR	2.1	1.02E-02
SELENOS	2.1	1.21E-05
FASN	2.1	1.09E-04
DNAJC3	2.1	2.07E-05
CD14	2.1	1.23E-02
PIK3C2A	2.1	1.70E-03
CTGF	2.1	2.04E-02
AREG	2.1	2.09E-02
IRF4	2.1	2.55E-03
CSRP2	2.1	1.90E-04
ZGRF1	2.1	7.88E-03
TMEM38B	2.1	1.48E-03
ACLY	2.2	5.17E-06
SLC41A2	2.2	5.17E-02
GINS1	2.2	2.93E-03
STC1	2.2	1.42E-02
KIAA0101	2.2	1.07E-03
LSS	2.2	1.54E-05
FICD	2.2	1.96E-04
PANK3	2.2	4.27E-02
DEPDC1	2.2	2.26E-02
MYOF	2.2	3.03E-05
CD79A	2.2	1.05E-02
SLC5A1	2.2	2.73E-04
LIPH	2.2	2.78E-04
FDPS	2.2	8.28E-05
TNFRSF26	2.2	5.68E-04
GFPT1	2.2	1.21E-05
SLC17A9	2.2	1.07E-05
DERL3	2.2	2.74E-04
DIAPH3	2.2	2.40E-02
SH2D1B	2.2	5.04E-02
CDR2	2.2	1.11E-04
LOXL4	2.2	3.47E-02
ADM5	2.2	5.60E-03
ITGA8	2.2	1.15E-04
OLFM4	2.2	1.48E-02
TGM2	2.2	3.66E-05
SPDL1	2.3	2.87E-02
KNL1	2.3	4.26E-03
FCGBP	2.3	2.08E-02
IL18	2.3	7.08E-03
PHLDA1	2.3	3.66E-04
TNFAIP6	2.3	2.84E-02
VSTM1	2.3	1.95E-02
PTGER1	2.3	1.21E-02
PTGS1	2.3	9.54E-06
GPER1	2.3	1.39E-02
FFAR4	2.3	1.81E-02
MKX	2.3	2.40E-02
HYOU1	2.3	4.56E-05
HSD11B1	2.3	7.62E-03
PDIA4	2.3	1.15E-04
CCL28	2.3	4.92E-03
CHIA	2.3	3.56E-02
BCAT1	2.4	4.81E-04
PTPRN2	2.4	2.45E-02
ADAM32	2.4	1.08E-02
DEPDC7	2.4	4.79E-02
SFRP1	2.4	2.73E-04
SMOC2	2.4	3.44E-03
SCNN1D	2.4	1.45E-02
PDZK1IP1L	2.4	4.24E-02
GJA1	2.4	6.11E-05
BMP7	2.4	6.17E-05
HIF1A	2.4	9.56E-06
SLC15A2	2.4	2.11E-03

NFIL3	2.4	3.14E-09
CDK1	2.4	1.89E-03
TNFRSF6B	2.4	4.77E-03
FGF2	2.4	5.70E-04
TRIB3	2.4	1.05E-02
CHEK1	2.4	3.35E-03
AHNAK2	2.4	4.81E-02
NDUFA4L2	2.4	1.77E-03
FADS1	2.4	6.98E-08
TIGIT	2.5	3.55E-03
ATF5	2.5	2.26E-02
GABRD	2.5	4.65E-02
FOS	2.5	2.09E-02
PRDX4	2.5	3.41E-05
ATP13A3	2.5	7.31E-03
IFIT1L1	2.5	3.82E-02
HSPH1	2.5	1.97E-02
SLC1A4	2.5	1.36E-03
LMAN1	2.5	3.59E-06
SHCBP1	2.5	1.06E-03
AHSA2	2.5	3.80E-05
MSMO1	2.5	5.94E-06
SMC2	2.5	1.70E-04
"MARCH3"	2.5	5.78E-04
SNORA63	2.5	4.32E-02
SOCS3	2.5	1.07E-03
LAMB3	2.5	2.26E-06
CCR2	2.5	2.63E-03
EGF	2.5	1.10E-03
SLC16A3	2.6	1.23E-03
EBP	2.6	1.17E-05
NT5DC2	2.6	1.65E-05
ADAM9	2.6	1.62E-07
RBPJL	2.6	1.02E-02
ROS1	2.6	3.77E-02
ITGA2	2.6	3.66E-05
ADAMTS4	2.6	4.92E-03
IGFBP4	2.6	2.53E-05
KRT18	2.6	4.34E-05
LOC102161685	2.7	3.95E-04
MYBL1	2.7	2.28E-02
BDNF	2.7	1.64E-02
HSD17B7	2.7	9.57E-06
PPP2R5E	2.7	2.55E-02
HSP90B1	2.7	1.17E-05
ELOVL6	2.7	1.65E-05
SNCA	2.7	1.40E-02
SLC7A5	2.7	7.21E-06
ABAT	2.7	2.61E-02
CSF3R	2.7	1.81E-02
TFF3	2.7	2.49E-03
MANF	2.7	6.81E-06
CXCR1	2.7	1.71E-02
IGLL5L	2.7	7.30E-03
SERPINB8	2.7	2.47E-05
GCNT3	2.7	1.21E-03
PNP	2.8	6.81E-06
FADS2	2.8	2.38E-06
PHLDA2	2.8	4.07E-04
TFRC	2.8	3.47E-05
SPHK1	2.8	3.05E-03
STARD4	2.8	1.65E-06
GPAT3	2.8	1.40E-04
CXCR2	2.8	2.64E-02
LDLR	2.8	1.77E-06
UGGT1	2.9	5.68E-03
TNC	2.9	8.70E-04
SQLE	2.9	7.52E-07
CXCL1	2.9	1.56E-02
ETV4	2.9	3.43E-04
ADAM20L2	2.9	2.39E-05
IL1RAP	2.9	2.14E-03
SNORA20E	2.9	4.65E-02
LCN2	3.0	1.80E-02

BAALC	3.0	5.32E-04
SEMA7A	3.0	1.23E-05
MIR9829	3.0	5.30E-02
SLC1A2	3.0	2.04E-03
TLE6	3.0	6.63E-03
FFAR2	3.0	5.18E-04
SHISA8	3.0	2.45E-02
HSPA5	3.0	1.21E-05
B3GNT6	3.0	2.31E-03
SLCO4A1	3.1	7.29E-05
SERPINE2	3.1	1.06E-04
DHRS9	3.1	4.24E-04
IDI1	3.1	3.39E-06
INSIG1	3.1	6.97E-07
C4BPB	3.1	5.70E-06
FGF7	3.2	4.44E-05
CLCA1	3.2	1.07E-03
PFKFB3	3.2	4.64E-06
IL13RA2	3.3	5.25E-02
POU2AF1	3.3	9.54E-06
CTSG	3.3	1.58E-02
NKX2-2	3.3	5.22E-03
CBS	3.4	1.61E-03
SLC2A3	3.4	1.42E-12
CXCL8	3.4	5.32E-04
MMP3	3.5	3.34E-02
ACAT2	3.5	1.27E-10
SLC12A8	3.5	7.40E-04
CXCL14	3.5	9.85E-08
SPHKAP	3.5	8.66E-04
TIAM1	3.5	2.62E-05
RAET1EL3	3.6	4.51E-03
SYNE4	3.6	2.31E-02
PLA2G3	3.6	7.51E-05
ITLN2L*	3.6	2.78E-02
CLEC10A	3.6	2.24E-02
HHIPL1	3.6	1.65E-05
IL1A	3.6	1.30E-03
CMAH	3.7	4.42E-02
MYOM3	3.7	1.26E-03
GPRC5D	3.7	4.11E-02
KLRG2	3.7	4.54E-02
MED12L	3.8	5.39E-05
TGM1	3.8	3.08E-02
ARNTL2	3.8	6.51E-06
HMGCS1	3.9	7.11E-09
SCD	3.9	9.81E-07
SHANK2	3.9	1.44E-03
MS4A7	3.9	2.55E-03
WNT2	3.9	6.32E-03
LIPG	4.0	1.77E-03
STEAP4	4.0	5.38E-03
BDKRB1	4.0	1.65E-03
COL8A1	4.1	1.19E-06
HEPHL1	4.1	5.31E-02
ABCA12	4.1	1.91E-03
IL26	4.1	2.96E-03
SFTPD	4.1	2.73E-02
ADAM12	4.2	4.78E-04
TAT	4.2	4.00E-02
CA4	4.3	9.18E-05
TIMP1	4.3	2.48E-09
NRG1	4.3	8.21E-05
LRP2	4.3	1.38E-02
C4BPA	4.3	1.37E-03
P4HA3	4.4	3.89E-04
SPDEF	4.4	3.89E-06
OSM	4.4	1.92E-04
F13A1	4.4	1.41E-09
WAP1	4.4	5.33E-03
KIAA1199	4.6	4.92E-03
IGFBP2	4.6	3.68E-07
PLAUR	4.6	3.65E-06
TNFRSF11B	4.6	1.51E-04

RN7SL1	4.6	2.45E-02
ANKRD26L3	4.6	3.02E-02
CAPNS2	4.7	3.43E-04
COL28A1	4.7	5.24E-04
SERPINA1	4.7	5.81E-03
PI3	4.7	1.63E-02
SLC4A11	4.8	5.93E-03
RNF39	4.8	2.65E-05
IGHE	5.0	2.59E-06
TNFRSF12A	5.0	6.86E-10
CENPE	5.0	4.28E-03
PHGDH	5.1	3.92E-05
CCL23	5.1	1.56E-04
NPPC	5.1	3.89E-03
MUC2	5.2	7.10E-08
CXCL6	5.3	2.39E-05
AQP9	5.3	1.56E-02
ITGB6	5.4	8.74E-11
IL1B	5.5	1.20E-03
GZMB	5.5	2.79E-04
SDS	5.6	1.53E-02
NXPH4	5.6	3.16E-05
NEUROG3	5.7	1.61E-03
IGH@	5.7	2.70E-05
MPTX	5.8	4.12E-06
CHI3L2	6.0	7.37E-04
ADAM20L6	6.1	2.85E-02
TREM1	6.4	4.61E-03
SLC6A13	6.5	1.54E-02
AGR2	6.5	1.79E-05
IL6	6.5	1.29E-04
PTGS2	6.5	8.39E-06
DIO2	6.5	3.02E-04
SPINK4	6.6	2.11E-06
ADAMTS16	6.6	2.55E-03
BPIFB2	6.7	2.43E-03
AKR1C1	7.0	5.11E-02
C1QL2	7.0	4.51E-02
CRLF1	7.1	3.16E-05
PSAT1	7.2	3.12E-04
TNIP3	7.3	4.56E-05
MCPT3*	7.3	2.80E-02
COL26A1	7.3	2.71E-04
HOXD1	7.4	6.83E-03
S100A2	7.4	9.54E-06
MMP20	7.4	4.14E-02
ADM2	7.9	1.29E-05
S100A3	8.0	6.29E-03
RHCG	8.0	4.93E-02
FAM71E2	8.2	9.06E-03
F3	8.2	7.74E-11
HK2	8.6	2.40E-06
SCGB3A1	8.8	1.90E-02
MMP9	8.9	1.02E-08
MMP1	9.4	4.44E-05
SERPINB2	9.8	2.12E-04
SNORA20	9.8	2.20E-03
SLC7A11	9.9	9.13E-04
KLRJ1	10.0	5.18E-04
RETNLB	10.1	2.14E-03
IL1R2	10.3	2.46E-06
SNORA73AL	10.5	2.14E-02
PRG4	10.7	1.39E-02
UPK1B	10.9	1.66E-08
PADI4	11.0	7.93E-03
BPIFB6	11.0	4.32E-02
SERPINB11	11.3	9.04E-04
PCSK9	11.5	5.33E-09
SLC5A8	11.9	4.56E-05
CYP7A1	12.7	4.64E-02
LYZ	13.0	7.77E-03
HCAR2	13.3	7.26E-04
SULT2A1	13.4	4.28E-02
CLEC4D	13.4	4.10E-02

CHI3L1	13.9	2.38E-06
REG4	15.8	2.97E-08
MAGEA13P	15.8	1.76E-02
MCHR1	17.9	7.59E-03
SERPINB7	19.3	1.85E-02
IL11	19.4	3.58E-04
MMP13	20.2	1.77E-06
ANXA8	21.8	2.21E-07
IL36A	22.7	3.90E-04
XDH	22.8	3.03E-02
CHIT1	24.2	1.65E-02
TFF2	25.1	3.57E-02
MMP8	27.1	7.94E-03
CLDN9	27.7	5.43E-02
IGHG1	29.1	1.93E-04
CLEC18A	29.5	7.94E-03
MMP7	33.4	3.22E-03
AQP5	33.5	4.54E-04
S100A8	38.3	5.18E-04
S100A12	38.4	4.48E-04
SDSL	39.6	1.38E-02
A3GALT2	40.6	5.54E-03
NTRK1	42.0	2.62E-07
S100A9	44.8	2.59E-04
SPINK6	46.2	5.38E-03
LTF	52.9	5.95E-03
TCN1	54.1	1.15E-04
PADI3	55.7	1.75E-02
SERPINA3-2	60.8	2.96E-06
TNN	61.3	4.18E-05
AVPR1B	62.6	5.42E-03
GNLY	68.8	2.42E-09
MMP12	101.0	2.26E-14
SCGB2A2	105.1	3.14E-07
ARG1	122.6	4.56E-05
IL4I1L	161.2	3.98E-05
REG3A	275.6	2.48E-03
PADI1	296.4	3.51E-03
TFF1	705.1	2.48E-05
