# Supplementary material for: Molecular and metabolomic changes in the proximal colon of pigs infected with Trichuris suis
Source: Sci Rep. 2020 Jul 30;10:12853. doi: 10.1038/s41598-020-69462-5 (PMC7393168; doi:10.1038/s41598-020-69462-5)
Supplement: Supplementary file 6 — Supplementary Table S6. [file 41598_2020_69462_MOESM6_ESM.pdf]

Molecular and metabolomic changes in the proximal colon of pigs infected with *Trichuris suis*

Harry Dawson[1], Celine Chen[1], Robert Li[2], Lauren Nicki Bell[3], Terez Shea-Donohue[4], Helene Kringel[5], Ethiopia Beshah[1], Dolores E. Hill[2], Joseph F. Urban Jr[1,2].

[1]United States Department of Agriculture, Agricultural Research Service, Northeast Area, Beltsville Human Nutrition Research Center, Diet Genomics and Immunology Laboratory, [2] Beltsville Agricultural Research Center, Animal Parasitology Disease Laboratory, Beltsville, MD; [3]Metabolon, Inc., Morrisville, NC; [4]University of Maryland School of Medicine, Baltimore, MD, USA; [5]Department of Veterinary Disease Biology, Faculty of Health and Medical Sciences, University of Copenhagen, Copenhagen, Denmark

Supplemental Table S6

Supplemental Table S6. Comparison of DEGs expressed at 52 days after inoculation in pigs infected with Trichuris suis with no worms (worm-free) versus those with worm.

Feature ID	Fold change	EDGE test: FDR p-value correction
SLC10A2	-297.1	1.18E-04
ALPI	-32.9	4.94E-06
ALDOB	-21.0	1.66E-06
BTNL2	-19.6	1.37E-02
AQP8	-15.1	2.08E-03
NXPE2	-13.8	2.92E-05
THAP12	-13.4	2.27E-02
CLCA4	-13.2	2.57E-05
HMGCS2	-12.8	0.00E+00
OASL	-12.1	1.20E-02
SLC26A3	-12.0	9.52E-08
FRMD1	-9.6	1.51E-03
FOXO6	-9.1	1.78E-04
NR1H4	-7.6	1.96E-06
GSTA2	-7.4	7.07E-03
CYP2B22	-6.8	5.35E-03
SLC30A10	-6.3	2.38E-02
SI	-6.1	2.79E-02
COL6A6	-5.7	3.03E-06
CXCL9	-5.7	3.65E-03
DACT2	-5.4	1.74E-02
PCK1	-5.4	1.48E-03
PADI2	-5.2	1.54E-06
HAS3	-5.2	5.01E-04
SULT1E1	-5.2	7.10E-07
GUCA2A	-5.1	1.44E-04
MOGAT2	-4.9	2.97E-02
NCR2	-4.8	1.35E-02
SLC51A	-4.8	3.48E-06
SSTR5	-4.8	1.30E-02
CYP26B1	-4.6	4.16E-08
ABCA6	-4.4	1.88E-04
SLC16A1	-4.4	0.00E+00
CXCL10	-4.4	7.18E-03
RSAD2	-4.3	2.28E-02
AGT	-4.3	4.12E-03
SLC25A34	-4.2	1.12E-05
GABRB2	-4.0	1.45E-04
TM6SF2	-4.0	1.95E-09
IFI27	-3.9	1.12E-02
CA1	-3.9	1.38E-08
P2RX3	-3.9	4.22E-03
ABCG2	-3.4	2.49E-03
WNT8B	-3.1	1.99E-03
CA12	-3.1	2.51E-06
SLC38A4	-3.0	3.10E-03
SELENBP1	-3.0	1.73E-07
PARP15	-3.0	6.17E-03
CHP2	-2.9	5.14E-05
CNTFR	-2.9	3.11E-03
CTBS	-2.9	4.10E-02
CLDN10	-2.8	4.58E-02
CXCL12	-2.8	2.48E-04
SCN2A	-2.7	4.98E-02
ABCA9	-2.7	5.24E-02
PRSS12	-2.7	9.44E-04
XCL1	-2.7	5.72E-03
ABCA8	-2.7	4.77E-02
GRIK4	-2.7	9.60E-03
IFI44	-2.7	2.55E-02
DHX58	-2.7	3.59E-02
IRF4L	-2.7	3.39E-02
CCL8	-2.6	7.80E-03
LYPD6	-2.6	3.08E-02
AHRR	-2.6	6.47E-04
HOXD10	-2.6	1.40E-03
UGT2C1	-2.5	1.18E-05
FBXO32	-2.5	2.94E-04
HS3ST3A1	-2.5	4.16E-02
UBE2L6	-2.5	1.39E-04
ZBP1	-2.5	1.11E-02
FOXS1	-2.5	5.82E-03
ABI3BP	-2.5	1.33E-03
PTPRD	-2.5	2.63E-04
CD36	-2.4	7.42E-05
CD300C	-2.4	8.64E-04
SLC24A4	-2.4	1.05E-02
LILRB3L4	-2.4	5.21E-02

MAMDC2	-2.4	6.26E-03
GBP2	-2.4	9.65E-03
AOC1	-2.4	4.46E-05
FABP1	-2.4	1.03E-02
FCER1A	-2.3	3.53E-02
SELENOP	-2.3	5.83E-05
SLC7A3L7*	-2.3	4.90E-02
NT5E	-2.3	4.92E-03
C11orf86	-2.2	2.08E-03
SEMA3A	-2.2	1.38E-02
SECTM1	-2.2	4.77E-02
PRDX6	-2.2	5.44E-05
CARD14	-2.2	2.07E-03
IGF2	-2.2	2.80E-02
TLR8	-2.2	2.49E-03
SLC7A8	-2.1	5.64E-03
GBP6	-2.1	3.20E-02
AHCYL2	-2.1	5.97E-05
CTSW	-2.1	1.42E-02
DBP	-2.1	1.42E-02
PARP14	-2.1	6.98E-03
SLA-DRA	-2.1	5.53E-03
SLC25A42	-2.1	7.36E-03
GLI2	-2.1	7.08E-03
MPEG1	-2.1	5.72E-03
ANK3	-2.1	2.79E-02
SLC7A3L4*	-2.1	4.13E-02
EDIL3	-2.1	5.28E-03
CD209	-2.1	3.13E-02
IL34	-2.1	4.12E-03
RYR2	-2.0	2.80E-03
KLRG1	-2.0	4.23E-02
CYP7B1	-2.0	1.59E-02
TXNIP	-2.0	3.65E-03
ADRB1	-2.0	7.57E-03
CD4	-2.0	1.03E-02
SLA-DRB1	-2.0	1.25E-02
ALDH1A3	-2.0	1.10E-02
GBP1	-2.0	4.04E-02
SLA-DQA1	-2.0	1.33E-02
IFI44L	-2.0	1.59E-02
P2RY13	-2.0	1.81E-02
CD74	-2.0	7.72E-03
ACE	-2.0	6.94E-03
ABCB1	-2.0	7.10E-03
PARP9	-1.9	1.04E-03
PCSK6	-1.9	2.26E-04
ANKH	-1.9	4.54E-02
HOXD11	-1.9	5.02E-02
GGT1	-1.9	4.29E-02
MAF	-1.9	1.80E-03
EGFLAM	-1.9	5.37E-03
CAVIN2	-1.9	9.17E-03
SYNPO	-1.9	3.13E-03
MS4A2	-1.9	2.34E-02
ADAMTS8	-1.9	1.07E-02
COLEC12	-1.9	7.57E-03
LY9L1*	-1.9	1.61E-02
CST3	-1.9	7.80E-03
MAOA	-1.9	7.84E-04
DAPK1	-1.9	1.20E-02
FOXF2	-1.9	5.72E-03
KLHL7	-1.9	2.88E-03
SEMA4D	-1.8	3.81E-02
RNF213	-1.8	3.66E-02
HAPLN4	-1.8	5.64E-03
ACSF2	-1.8	1.98E-04
SATB1	-1.8	2.46E-02
PKNOX2	-1.8	5.16E-02
NR1D1	-1.8	3.13E-02
SLC16A5	-1.8	9.64E-03
CADM1	-1.8	2.11E-02
TEF	-1.8	3.94E-03
MEIS2	-1.8	5.29E-02
AKR1E2	-1.8	4.12E-03
ADGRL1	-1.8	4.66E-02
HIC1	-1.8	1.41E-02
MAP2K6	-1.8	4.90E-02
TMPRSS2	-1.8	1.60E-02
LGALS9	-1.8	1.10E-02

CLCN2	-1.8	3.54E-05
POU6F1	-1.8	3.37E-02
CD86	-1.8	1.93E-02
LTBP4	-1.8	5.61E-03
WNT2B	-1.8	1.98E-02
ADA	-1.7	2.04E-02
LIFR	-1.7	7.18E-03
ARHGAP6	-1.7	3.47E-02
IFITM1L2	-1.7	8.91E-03
FGFRL1	-1.7	1.45E-02
CIITA	-1.7	4.54E-02
SLA-DMB	-1.7	2.70E-02
IHH	-1.7	7.62E-03
ZBTB4	-1.7	1.22E-03
TAP1	-1.7	2.23E-02
SLC9A2	-1.7	1.92E-03
PTCH1	-1.7	4.04E-02
C1QA	-1.7	1.29E-02
CDKN2B	-1.7	7.89E-03
SLA-2	-1.7	5.02E-02
BDH1	-1.7	7.89E-03
FCGR3A	-1.7	1.21E-02
APOL2	-1.7	3.42E-02
C1QC	-1.7	1.61E-02
ABCA1	-1.7	5.31E-02
CDH24	-1.7	3.29E-02
IL2RB	-1.7	2.69E-02
JAML	-1.7	3.23E-02
TNFAIP2	-1.7	1.04E-03
ABCB6	-1.6	5.08E-03
CTSD	-1.6	1.68E-02
DOK2	-1.6	3.97E-02
SLC45A3	-1.6	2.17E-02
IGSF3	-1.6	5.40E-02
TMPRSS4	-1.6	6.34E-03
CD33	-1.6	4.32E-02
FGF11	-1.6	2.39E-02
SLA-3	-1.6	4.54E-02
TCF21	-1.6	1.33E-02
SNX4	-1.6	5.72E-03
PNPLA6	-1.6	6.03E-03
CD302	-1.6	5.19E-02
PFKFB4	-1.6	5.72E-03
RETSAT	-1.6	7.89E-03
LCP2	-1.6	1.11E-02
GIMAP8	-1.6	1.73E-02
NADK	-1.6	4.96E-03
F8	-1.6	3.01E-02
SCARB2	-1.6	5.21E-04
SLC25A27	-1.6	7.18E-03
ELMSAN1	-1.6	3.05E-02
SLA-11	-1.6	4.46E-02
ZNF664	-1.6	5.37E-03
SLC8A1	-1.6	5.29E-02
TP53INP2	-1.6	3.64E-02
RALGDS	-1.6	2.21E-02
COL4A6	-1.6	4.50E-02
SIPA1	-1.6	5.29E-02
CSF2RB	-1.5	4.36E-02
CD300H	-1.5	3.24E-02
GSTP1	-1.5	4.90E-02
PIK3IP1	-1.5	4.80E-02
TMEM140	-1.5	3.04E-02
CSF1	-1.5	1.68E-02
PLCD1	-1.5	3.39E-02
VDR	-1.5	3.04E-02
IL10RA	-1.5	3.17E-02
IRF9	-1.5	4.68E-02
ASAP3	-1.5	4.15E-02
MERTK	-1.5	4.10E-02
SYNE1	-1.5	4.69E-02
DTX3	-1.5	2.14E-02
BSG	-1.5	2.27E-02
ADCY9	-1.5	5.72E-03
MOV10	-1.5	1.20E-02
HPSE	-1.5	5.25E-02
UGT1A10	-1.5	8.65E-03
PNPLA2	-1.5	3.42E-02
TST	-1.5	5.16E-02
COBL	-1.5	1.01E-02

TSPAN7	-1.5	3.19E-02
UNC93B1	-1.5	4.34E-02
LRP4	-1.5	5.02E-02
MAP3K3	-1.5	1.38E-02
CSF1R	-1.5	4.54E-02
GABARAPL1	-1.5	2.90E-02
PCGF2	-1.5	5.29E-02
SLC30A7	1.5	3.01E-02
TMED2	1.5	1.87E-02
HMGB1	1.5	1.59E-02
ENTPD6	1.5	4.58E-02
EPRS	1.5	1.81E-02
SMAD1	1.5	1.17E-02
MAP4K4	1.5	9.03E-03
SELENOS	1.5	3.07E-02
SLC39A11	1.5	3.01E-02
EDEM1	1.5	2.46E-02
MAPK6	1.5	4.90E-02
MET	1.5	4.52E-02
SLC30A5	1.5	3.16E-02
NECTIN1	1.5	3.13E-02
MAL2	1.5	8.79E-03
CDH17	1.5	3.46E-02
COPB1	1.5	4.68E-02
SHMT2	1.5	1.06E-02
VLDLR	1.5	7.00E-03
ACTG1	1.5	2.84E-03
ARF2	1.5	4.54E-02
SLC39A7	1.5	1.20E-02
ERLEC1	1.5	4.94E-02
SLC7A1	1.5	3.05E-02
SPRED1	1.5	7.34E-03
SELENOF	1.5	1.19E-02
S100A11	1.5	3.35E-02
UFM1	1.5	2.36E-02
ANXA5	1.5	1.54E-02
TACC3	1.5	3.05E-02
PGK1	1.5	1.73E-02
HMGB2	1.5	1.72E-02
CTNNB1	1.5	8.88E-03
MYBL2	1.5	3.16E-02
MCM6	1.5	2.31E-02
STT3A	1.5	2.64E-02
C9	1.5	5.09E-02
CBFA2T2	1.5	2.97E-02
ANXA2	1.5	9.17E-03
SERP1	1.5	8.65E-03
PRTFDC1	1.5	4.54E-02
RACGAP1	1.5	2.85E-02
AMIGO3	1.6	5.61E-03
OSMR	1.6	1.24E-02
RPN2	1.6	1.62E-02
ODC1	1.6	4.12E-03
FZD6	1.6	4.12E-02
HMGN2	1.6	1.45E-03
SPATS2	1.6	3.11E-03
UBE2S	1.6	1.44E-02
PLK1	1.6	1.79E-02
SKA2	1.6	5.02E-02
TSPAN13	1.6	1.37E-02
CHEK2	1.6	1.60E-02
BNIP3	1.6	7.73E-03
KCNE3	1.6	6.59E-03
FICD	1.6	2.91E-02
SLC35C1	1.6	7.89E-03
SEC24D	1.6	1.45E-03
SELENOM	1.6	1.73E-02
TMEM38B	1.6	5.49E-02
MIA3	1.6	4.10E-03
SLC1A5	1.6	5.72E-03
SERPINB1	1.6	1.75E-02
P2RX4	1.6	4.88E-03
BIRC5	1.6	1.82E-02
GARS	1.6	1.95E-03
ADGRG6	1.6	2.36E-02
CASC4	1.6	2.07E-03
TK1	1.6	3.23E-02
CLIC2	1.6	1.22E-02
LRRC59	1.6	1.88E-02
RRBP1	1.6	1.17E-02

STARD10	1.6	4.04E-03
SLC17A9	1.6	5.72E-03
P4HA2	1.6	3.60E-03
KIF22	1.6	9.28E-03
CYP51A1	1.6	5.76E-03
C3	1.6	3.66E-02
CAD	1.6	1.54E-03
SEL1L3	1.6	7.42E-03
PCDH12	1.6	5.06E-02
ARG2	1.6	2.16E-02
S100A6	1.6	1.55E-02
CMAS	1.6	6.03E-03
CFI	1.6	4.23E-02
DHCR7	1.6	1.99E-03
POLE	1.6	1.60E-02
FOXA1	1.6	3.80E-02
MANF	1.6	3.08E-02
NSDHL	1.6	4.96E-03
WLS	1.6	1.61E-02
CDH1	1.6	8.68E-03
B4GALT1	1.6	4.04E-02
C9H1orf116	1.6	7.00E-03
GFPT1	1.7	3.70E-03
SEC11C	1.7	8.53E-03
PPIB	1.7	3.55E-03
IL33	1.7	1.40E-02
HSP90B1	1.7	2.68E-02
DNAJC10	1.7	3.09E-03
PLK2	1.7	9.11E-03
SEC61G	1.7	7.96E-03
PANK1	1.7	2.78E-02
ACSL3	1.7	2.03E-03
ARF4	1.7	1.90E-03
XBP1	1.7	3.59E-03
STARD5	1.7	4.28E-03
TSPAN12	1.7	4.45E-04
RIPK3	1.7	5.64E-03
FASN	1.7	3.60E-03
DNA2	1.7	3.13E-02
GALNT7	1.7	2.49E-03
CDC25C	1.7	1.33E-02
GNA14	1.7	5.35E-03
PKM	1.7	1.40E-03
WFS1	1.7	1.61E-03
LIPH	1.7	8.88E-03
BLM	1.7	3.03E-02
TPX2	1.7	6.03E-03
ALDOC	1.7	1.06E-02
HIF1A	1.7	3.73E-03
BST1	1.7	5.72E-03
MMD	1.7	4.10E-03
SC5D	1.7	1.42E-03
ATOH1	1.7	2.37E-02
DERL3	1.7	7.70E-03
PHLDA1	1.7	1.31E-02
YARS2	1.7	2.77E-02
DDIT4	1.7	9.24E-04
SLC5A1	1.7	6.38E-03
SLC35A3	1.7	1.61E-02
PDIA4	1.7	7.74E-03
FLVCR2	1.8	2.73E-03
MANSC1	1.8	2.51E-04
BUB1	1.8	2.17E-02
FDPS	1.8	2.52E-03
PGM3	1.8	9.17E-04
HELLS	1.8	7.06E-03
MVD	1.8	5.01E-04
CCNB2	1.8	2.21E-02
BGN	1.8	4.69E-02
ATOX1	1.8	8.67E-03
PNP	1.8	7.47E-03
NT5DC2	1.8	5.72E-03
LSS	1.8	3.41E-04
AACS	1.8	1.18E-04
ARNTL2	1.8	5.25E-02
FADS1	1.8	8.80E-05
RRM2	1.8	2.15E-04
BUB1B	1.8	9.28E-03
ECM1	1.8	3.23E-03
TOP2A	1.8	4.25E-03

DGAT2	1.8	3.05E-02
E2F7	1.8	1.34E-02
ETV5	1.8	1.26E-02
SLC50A1	1.8	9.18E-04
ECSCR	1.8	3.94E-03
MIS18A	1.9	1.76E-02
ADAM9	1.9	2.26E-04
AFF2	1.9	4.49E-02
TRAIP	1.9	5.08E-03
PHLDA2	1.9	2.92E-02
ZGRF1	1.9	2.01E-02
SMOC2	1.9	2.90E-02
CDCA8	1.9	9.35E-04
SLC24A5	1.9	4.98E-02
HMGCR	1.9	1.78E-04
GADD45A	1.9	5.02E-03
CFB	1.9	2.38E-02
GINS1	1.9	7.55E-03
KIF23	1.9	5.99E-03
ACLY	1.9	1.76E-05
BCAT1	1.9	5.18E-03
CCR2	1.9	3.11E-02
MYOF	1.9	1.39E-04
CD163	1.9	2.29E-02
THBD	1.9	3.32E-03
COL7A1	1.9	1.47E-03
BMP7	1.9	1.22E-03
SMC2	1.9	4.42E-03
FDFT1	1.9	1.14E-05
MASTL	1.9	4.54E-02
SULT1A1	1.9	1.57E-02
PRDX4	1.9	1.03E-03
"MARCH3"	1.9	8.78E-03
SLC7A5	1.9	1.40E-03
CHEK1	1.9	2.00E-02
CD24	1.9	6.90E-03
STARD4	1.9	8.33E-04
HSD17B7	1.9	1.22E-03
LMAN1	2.0	1.88E-04
SERPINE2	2.0	1.59E-02
PKD1L2	2.0	4.19E-02
SLC25A35	2.0	9.64E-03
DEPDC1	2.0	3.05E-02
ADM5	2.0	7.68E-03
GPR19	2.0	1.76E-02
GNE	2.0	5.66E-05
COL8A1	2.0	1.40E-02
CD14	2.0	7.89E-03
CDK1	2.0	7.19E-03
IGLL5L	2.0	5.49E-02
SERPINB5	2.0	9.60E-04
AZGP1	2.0	3.20E-02
KIAA0101	2.0	7.84E-04
KRT18	2.0	1.04E-03
PCK2	2.0	3.22E-04
ADAM20L2	2.0	2.60E-03
TMED3	2.0	1.16E-03
SHCBP1	2.0	5.72E-03
HMMR	2.0	1.22E-03
LDLR	2.1	2.26E-04
DHRS9	2.1	1.73E-02
SEMA7A	2.1	1.51E-03
MEST	2.1	2.12E-02
CXCR4	2.1	5.27E-04
SPDL1	2.1	2.79E-02
F13A1	2.1	1.19E-03
ZNF215	2.1	2.39E-02
KNL1	2.1	2.84E-03
SLC1A2	2.1	2.44E-02
ASGR2	2.1	3.46E-02
GCNT3	2.1	7.34E-03
ITGA2	2.2	1.50E-04
SERPINB8	2.2	2.45E-04
IGHE	2.2	1.73E-02
CFHR2	2.2	4.39E-02
MYBL1	2.2	4.61E-02
STC1	2.2	3.42E-03
HIST1H3A	2.2	9.33E-03
EGF	2.2	1.60E-03
PFKFB3	2.2	5.86E-04

MSMO1	2.2	4.56E-06
GALNT6	2.2	3.33E-06
IDI1	2.2	2.17E-04
FABP3	2.3	1.30E-03
VSTM1	2.3	7.89E-03
FADS2	2.3	1.63E-05
CFAP74	2.3	3.65E-03
MED12L	2.3	6.53E-03
SQLE	2.3	8.68E-06
SPHKAP	2.3	1.77E-02
HP	2.3	1.38E-02
FFAR4	2.3	5.35E-03
C4BPB	2.3	1.66E-04
LOC102161685	2.3	4.59E-04
EBP	2.3	4.62E-06
LIPG	2.3	4.46E-02
TMEM158	2.4	4.39E-02
OSM	2.4	1.97E-02
PTGER1	2.4	2.11E-03
LAMB3	2.4	1.73E-07
ELOVL6	2.4	1.46E-05
IFIT1L1	2.4	2.12E-02
SCNN1D	2.4	4.59E-03
FGF7	2.4	6.47E-04
IGFBP4	2.4	1.00E-05
CXCL14	2.4	2.69E-05
TFF3	2.4	2.78E-03
POU2AF1	2.4	1.75E-04
CACNA1F	2.4	4.37E-02
TNFRSF12A	2.5	1.07E-04
TFRC	2.5	2.06E-05
PPP2R5E	2.5	1.68E-02
HSD11B1	2.5	5.23E-04
ROS1	2.5	2.01E-02
SPHK1	2.5	2.43E-03
ACAT2	2.5	4.51E-08
FCN2	2.5	2.74E-03
SCD	2.5	1.62E-04
GPAT3	2.5	7.03E-05
BAALC	2.6	9.35E-04
PTPRN2	2.6	3.00E-03
MMP9	2.6	6.92E-03
IGH@	2.6	1.24E-02
ME1	2.6	5.20E-03
HHIPL1	2.6	1.78E-04
ETV4	2.7	1.36E-04
SNCA	2.7	4.96E-03
PLA2G3	2.7	4.13E-04
HMGCS1	2.8	3.97E-07
ADGRE1	2.8	3.05E-02
PLAUR	2.8	4.01E-04
WAP1*	2.8	3.59E-02
CLCA1	2.8	6.25E-04
TMEM190	2.8	4.80E-02
NKX2-2	2.8	5.20E-03
CLEC10A	2.9	3.05E-02
LCN2	2.9	6.98E-03
B3GNT6	2.9	5.44E-04
SLC11A1	2.9	2.78E-02
TIMP1	2.9	2.65E-07
INSIG1	2.9	1.64E-08
ALDH3B2	2.9	1.18E-02
TGM3	3.0	3.20E-02
TREM2	3.0	7.72E-03
MMP1	3.1	2.86E-02
SLC12A8	3.1	3.65E-04
ABAT	3.1	1.97E-03
ADAM12	3.1	1.29E-03
PDZK1IP1L	3.1	8.64E-04
ITGB6	3.1	2.10E-07
CCL23	3.1	2.69E-03
TLE6	3.2	5.27E-04
PHGDH	3.3	5.01E-04
PTGS2	3.4	8.84E-04
GABRD	3.4	4.54E-04
CBS	3.5	7.42E-05
SPDEF	3.5	2.29E-06
RN7SL1	3.5	3.19E-02
TNIP3	3.5	3.16E-03
LRP2	3.6	1.26E-02

SHANK2	3.6	4.23E-04
SYNE4	3.6	4.65E-03
SLC7A11	3.7	3.60E-02
P4HA3	3.7	1.39E-04
STRA6	3.8	6.03E-03
MUC2	3.8	1.60E-07
IL1R2	3.8	1.61E-03
NEUROG3	3.8	3.07E-03
TNFRSF11B	3.9	5.64E-05
SERPINB2	3.9	1.24E-02
NPC1L1	3.9	4.56E-02
PSAT1	3.9	3.89E-03
NTRK1	4.0	2.12E-02
COL28A1	4.0	1.89E-04
CAPNS2	4.1	7.03E-05
S100A2	4.2	1.75E-04
HK2	4.2	1.78E-04
MYOM3	4.2	1.12E-05
SAA3	4.5	3.27E-02
PADI4	4.5	4.99E-02
RNF39	4.5	6.41E-07
SERPINA1	4.5	9.03E-04
S100A3	4.5	2.35E-02
AADAC	4.6	3.59E-03
HOXD1	4.6	9.07E-03
ADM2	4.7	7.03E-05
WNT2	4.7	1.78E-04
MPTX	4.9	2.10E-07
UPK1B	5.0	2.36E-06
BPIFB2	5.0	1.64E-03
FAM71E2	5.0	3.52E-02
F3	5.2	1.85E-10
SNORA48	5.2	2.03E-03
CHI3L1	5.2	2.51E-04
PI3	5.3	1.15E-03
NPPC	5.7	3.92E-04
AGR2	5.7	6.15E-07
COL26A1	5.8	6.82E-05
SPINK4	5.8	1.64E-08
BPIFB6	6.0	4.61E-02
GYS2	6.0	4.66E-02
MCHR1	6.1	3.59E-02
SERPINB11	6.3	3.20E-03
IL36A	6.8	6.44E-03
SULT2A1	7.5	5.36E-02
ANXA8	7.6	1.18E-05
TRIM6	7.8	9.11E-03
ADAM20L6	8.1	1.07E-03
CRLF1	8.2	1.09E-08
ADAMTS16	8.3	2.23E-05
IL11	8.3	1.22E-03
PCSK9	9.0	1.11E-11
A3GALT2	9.7	1.38E-02
REG4	9.9	1.95E-09
AQP5	10.2	1.63E-03
IGHG1	10.3	8.11E-04
MMP7	10.5	7.62E-03
S100A8	10.8	2.17E-03
S100A9	11.0	2.03E-03
MMP8	11.4	3.47E-03
MMP13	11.4	2.65E-07
TFF2	12.2	3.25E-02
SLC6A2	13.1	2.14E-02
S100A12	13.4	7.36E-04
PRG4	13.5	3.43E-04
MMP12	13.9	7.63E-10
C1QL2	15.4	1.39E-04
SERPINA3-2	16.2	1.05E-06
ARG1	16.6	8.41E-04
SCGB2A2	17.9	4.89E-06
POU5F1	19.0	3.78E-02
PADI3	19.0	4.69E-03
RHCG	19.8	5.18E-04
SPINK6	21.5	4.16E-04
WNT7A	25.9	4.23E-02
IL4I1L	26.0	1.39E-04
CLEC18A	46.3	9.53E-05
PADI1	51.6	6.25E-04
TFF1	93.2	3.12E-06
