# Supplementary material for: Molecular and metabolomic changes in the proximal colon of pigs infected with Trichuris suis
Source: Sci Rep. 2020 Jul 30;10:12853. doi: 10.1038/s41598-020-69462-5 (PMC7393168; doi:10.1038/s41598-020-69462-5)
Supplement: Supplementary file 7 — Supplementary Table S7. [file 41598_2020_69462_MOESM7_ESM.pdf]

Molecular and metabolomic changes in the proximal colon of pigs infected with *Trichuris suis*

Harry Dawson[1], Celine Chen[1], Robert Li[2], Lauren Nicki Bell[3], Terez Shea-Donohue[4], Helene Kringel[5], Ethiopia Beshah[1], Dolores E. Hill[2], Joseph F. Urban Jr[1,2].

[1]United States Department of Agriculture, Agricultural Research Service, Northeast Area, Beltsville Human Nutrition Research Center, Diet Genomics and Immunology Laboratory, [2] Beltsville Agricultural Research Center, Animal Parasitology Disease Laboratory, Beltsville, MD; [3]Metabolon, Inc., Morrisville, NC; [4]University of Maryland School of Medicine, Baltimore, MD, USA; [5]Department of Veterinary Disease Biology, Faculty of Health and Medical Sciences, University of Copenhagen, Copenhagen, Denmark

Supplemental Table S7

Supplemental Table S7: Clustered Up-regulation of Physiology and Immunology DEGs

Gene	Functional Categorization	52 DPI w worms vs Control Fold	EDGE FDR Adj p value	52 DPI w/o worms vs Control Fold	EDGE FDR Adj p value	(52 DPI Inf w/o worms vs w Worms Fold	EDGE FDR Adj p value	(21 DPI Inf vs Control) Fold	EDGE FDR Adj p value
	**Antibacterial Response**								
BPIFB2	Bacterial Binding (12185532); BPI SF	6.7	2.43E-03			5.0	1.64E-03		
BPIFB6	Bacterial Binding (12185532); BPI SF	11.0	4.32E-02			6.0	4.61E-02		
GNLY	Antimicrobial Peptide (15996224); SapB 2 SF	68.8	2.42E-09	132.0	2.98E-11				
HMGN2	Antimicrobial Peptide (16204630); HMG14/17 SF							1.6	1.45E-03
LCN2	Antibacterial Response (15531878, 25398327); Lipocalin SF	3.0	1.80E-02			2.9	6.98E-03		
LTF	Antibacterial Response (8423097); Transferrin SF	52.9	5.95E-03						
LYZ	Antibacterial Response (28804131); LYZ1 SF	13.0	7.77E-03						
NTRK1	Antibacterial Response (25359976); Allergic Inflammation (25389033); Ig SF; LRRCT SF	42.0	2.62E-07	10.6	3.53E-03	4.0	2.12E-02		
OLFM4	(-) Regulator of Antibacterial Responses (20534456, 22844115); OLF SF	2.2	1.48E-02						
PADI4	Antibacterial Responses (20733033); PAD SF	11.0	7.93E-03			4.5	4.99E-02		
PI3	Antimicrobial Peptide Activity (17964057); WAP SF	4.7	1.63E-02			5.3	1.15E-03	4.6	2.55E-08
S100A14	Antibacterial Response (15568027); S100 SF (17625598)							1.8	2.70E-02
SLPI	Antimicrobial Peptide Activity (18075823); WAP SF							4.8	2.22E-11
SPHK1	Antibacterial Response (20634980, 26113114); DAGK Cat SF					2.5	2.43E-03	2.8	3.05E-03
UPK1B	E. coli Attachment factor (9822381); Tetraspanin LEL SF	10.9	1.66E-08			5.0	2.36E-06		
	**Antiparasitic Response**								
AREG	Antiparasitic Response (17170297); Th2-Associated (17170297); PHA02887 SF	2.1	2.09E-02					2.2	8.37E-03
ARG1	Antiparasitic Response (19764983); M2-associated (5879104); Arginase HDAC SF	122.6	4.56E-05			16.6	8.41E-04	30.1	2.28E-07
CHIA	Antiparasitic Response (15618176); Th2-Associated (11553626); GH18 Chitinase Like SF	2.3	3.56E-02						
GATA3	(+) Regulator of Th2-Cell/ILC2 Differentiation (19375293, 23733962); ZnF GATA TF SF			4.4	4.43E-04				
IGHE	Antiparasitic Response (24592267); Ig SF; Ig Heavy Chain Constant Region SF	5.0	2.59E-06	2.3	4.86E-02	2.2	1.73E-02		
IRF4	Antiparasitic Response (20729857); M2-associated (20729857); IRF TF SF	2.1	2.55E-03						
REG3A	Antiparasitic Response (25240019); PRR (16931762)	275.6	2.48E-03	168.9	2.26E-02				
REG4	Antiparasitic Response (25240019); PRR (20692269); CLECT SF	15.8	2.97E-08			9.9	1.95E-09	11.1	8.84E-20
RETNLB	Antiparasitic Responses (15340149); Th2-Associated (12574343); Resistin SF	10.1	2.14E-03						
SERPINB2	Antiparasitic Response (23630350) M2-associated (23355731); SERPIN SF	9.8	2.12E-04			3.9	1.24E-02	16.2	4.90E-32
TFF1	Antiparasitic Response (21569362); Trefoil SF	705.1	2.48E-05			93.2	3.12E-06	25.9	2.62E-06
TFF2	Antiparasitic Response (22329990); Trefoil SF	25.1	3.57E-02			12.2	3.25E-02	37.1	6.93E-10
TFF3	Epithelial Wound Healing (16410243); IL-4 Induced (15004182); Trefoil SF	2.7	2.49E-03			2.4	2.78E-03		
	**Complement Related**								
FCN1	Complement Activation (20375620); PRR (21490156); FReD SF								
FCN2	Complement Activation (19632990); PRR (15331601); FReD SF					2.5	2.74E-03	2.9	9.94E-05
C1QL2	Complement Fixation; BAI3 Ligand (21262840); C1q SF	7.0	4.51E-02			15.4	1.39E-04		
C3	Complement Component; A2M SF; ANATO SF; IsoPREN C2-like SF; MG1 SF; NTR-like SF	1.7	3.10E-02			1.6	3.66E-02		
C4BPA	Complement Component; CCP SF	4.3	1.37E-03						
C4BPB	Complement Component; CCP SF	3.1	5.70E-06			2.3	1.66E-04		
C9	Complement Component; MACPF SF	1.6	3.93E-02			1.5	5.09E-02		
CFB	Alternative Complement Activation Pathway; CCP SF; TSP-1 SF; vWFA SF	2.1	1.92E-02			1.9	2.38E-02		
CFI	Complement factor; SRCR SF	1.7	4.15E-02						
CFHR2	Factor H (fH)-related Protein Family; CCP SF					2.2	4.39E-02		
	**Cytokines, Chemokines, Growth Factors and Receptors**								
ACKR3	CXCL12 Receptor (16107333); CXC Chemokine R SF	1.5	4.95E-02						
AZGP1	Adipocytokine (18663996); MHCI SF; Immunoglobulin SF					2.0	3.20E-02		
BDNF	Cytokine (19348610); Neurotrophic; NGF SF	2.7	1.64E-02						
BMP7	Cytokine; Development; TGFB SF	2.4	6.17E-05			1.9	1.22E-03		
CCL23	CCR1 Ligand (15927850); CCL Chemokine SF	5.1	1.56E-04			3.1	2.69E-03		
CCL28	CCR3 Ligand (10975800); CCL Chemokine SF	2.3	4.92E-03						
CCR1	CCL14 Receptor; CCL16 Receptor; CCL23 Receptor; CCL Chemokine R SF								
CCR2	CCL2 Receptor; CCL7 Receptor; CCL13 Receptor; CCL Chemokine R SF	2.5	2.63E-03			1.9	3.11E-02		
CRLF1	Cytokine (19933857); IL6 Receptor Ligand (19933857); Ig SF; FN3 SF	7.1	3.16E-05			8.2	1.09E-08		
CSF3R	Cytokine Receptor; Lep Receptor Ig SF	2.7	1.81E-02						
CXCL1	CXCR2 Ligand; CXC Chemokine SF	2.9	1.56E-02						
CXCL14	CXCR4 Ligand (28360196); CXC Chemokine SF	3.5	9.85E-08			2.4	2.69E-05	4.4	2.73E-06
CXCL16	CXCR6 Ligand; CXC Chemokine SF							2.0	2.89E-03
CXCL17	GPR35 Ligand (25411203); CXC Chemokine SF							4.5	7.44E-06
CXCL6	CXCR1 Ligand; CXCR2 Ligand; CXC Chemokine SF	5.3	2.39E-05	3.1	1.01E-02				
CXCL8	CXCR1 Ligand; CXCR2 Ligand; CXC Chemokine SF	3.4	5.32E-04						
CXCR1	CXCL1 R; CXCL2 R; CXCL3 R; CXCL6 R; CXCL8 R; CXC Chemokine R SF	2.7	1.71E-02						
CXCR2	CXCL1 R; CXCL2 R; CXCL3 R; CXCL6 R; CXCL8 R; CXC Chemokine R SF	2.8	2.64E-02						
CXCR4	CXCL12 R; CXC Chemokine R SF	1.9	1.45E-02			2.1	5.27E-04		
EGF	Wound Healing (2045428); LY SF; vWFA SF; EGF CA SF; PHA03099 SF	2.5	1.10E-03			2.2	1.60E-03		
IL11	Cytokine (27312790); TH2-Associated (15699166); IL11 SF; IL6 SF	19.4	3.58E-04			8.3	1.22E-03	21.6	6.84E-03
IL13RA2	(-) Regulator of IL-13 Signaling (12642602); Th2-Associated (17697639) IL6Ra-bind SF	3.3	5.25E-02						
IL17C	IL-17RE Ligand (21993849); IL17 SF							5.0	7.15E-03
IL17RD	Positive Regulator of IL17 Receptor Signaling; IL17 R DN SF	2.0	2.03E-02						
IL18	Th1-Associated (10679398); IL1 SF	2.3	7.08E-03						
IL1A	Positive Regulator of Inflammation; IL1 SF	3.6	1.30E-03					2.4	1.55E-03
IL1B	Positive Regulator of Inflammation; IL1 SF	5.5	1.20E-03						
IL1R2	IL1B Decoy Receptor; M2-associated (8786316); I-set SF; Ig SF	10.3	2.46E-06			3.8	1.61E-03	6.1	1.51E-06
IL1RAP	Th1-Associated (10925275); Ig SF; TIR 2 SF	2.9	2.14E-03						
IL26	STAT1 Signaling (14764663); STAT3 Signaling (14764663); Th17-Associated (17676044)	4.1	2.96E-03						
IL33	Th2-Associated (16286016); IL1 SF; IL33 SF	2.0	9.72E-04			1.7	1.40E-02		
IL36A	Positive Regulator of Inflammation (23029241); IL1 SF	22.7	3.90E-04			6.8	6.44E-03	7.2	2.58E-03
IL36B	Positive Regulator of Inflammation (16646978); IL1 SF							30.8	1.02E-02
IL6	Positive Regulator of Inflammation; TH2-Associated (12431386); IL6 SF	6.5	1.29E-04						
MDK	Cytokine; PTN MK N SF							2.8	4.44E-04
NRG1	Cytokine (12204892); Ig SF; EGF CA SF; Neuregulin SF	4.3	8.21E-05					3.8	2.41E-02
OSM	Positive Regulator of Inflammation (12496442); LIF OSM SF	4.4	1.92E-04			2.4	1.97E-02		
OSMR	Type I Cytokine Receptor Family; IL6Ra-bind SF	2.0	1.41E-04			1.6	1.24E-02		
PTN	Cytokine; PTN MK N SF							1.9	1.03E-02
TNFRSF10B	Positive Regulator of Apoptosis (9311998); DD SF; TNFR SF	1.8	3.08E-03						
TNFRSF11A	TNFR SF	1.7	3.61E-02						
TNFRSF11B	Crohn's Disease (15803021); Decoy Receptor; DD SF; TNFR SF	4.6	1.51E-04			3.9	5.64E-05		
TNFRSF12A	Positive Regulator of Apoptosis (21525013); Wound Healing (11728344); TNFR SF	5.0	6.86E-10	2.0	2.26E-02	2.5	1.07E-04		
TNFRSF26	TNFR SF							2.5	2.44E-02
TNFRSF6B	Decoy Receptor; Th2-Associated (14657214); TNFR SF	2.4	4.77E-03						
TNFRSF8	TNFSF8 Receptor; Th2-associated (9036953); TNFR SF							4.0	2.04E-02
TNFSF15	Th1-Associated (14568967); TNF SF	1.8	2.54E-02						
TNFSF18	TNFRSF18/GITR Ligand; TNF SF							3.8	3.17E-02

	**Extracellular Matrix and Remodeling**											
A2ML1	Protease Inhibitor (16298998); A2M SF										16.0	2.14E-03
ADAM12	Metalloprotease; Pep M12B Propep; ZnMc SF; Disintegrin SF; ADAM CR SF	4.2	4.78E-04					3.1	1.29E-03			
ADAM20L2*	Metalloprotease; Pep M12B Propep SF; ZnMc SF; ADAM CR SF	2.9	2.39E-05					2.0	2.60E-03			
ADAM20L6*	Metalloprotease; Pep M12B Propep SF; ZnMc SF; ADAM CR SF	6.1	2.85E-02					8.1	1.07E-03			
ADAM30	Metalloprotease; Pep M12B Propep; ZnMc SF; Disintegrin SF; ADAM CR SF										9.6	8.58E-03
ADAM32	Metalloprotease; Pep M12B Propep; ZnMc SF; Disintegrin SF; ADAM CR SF	2.4	1.08E-02									
ADAM9	Metalloprotease; Pep M12B Propep SF; ZnMc SF; Disintegrin SF; ADAM CR SF	2.6	1.62E-07					1.9	2.26E-04			
ADAMTS1	Metalloprotease; Pep M12B Propep SF; ZnMc SF; ADAM CR SF; TSP 1 SF										2.3	4.06E-04
ADAMTS16	Metalloprotease; Pep M12B Propep SF; ZnMc SF; ADAM CR SF; TSP 1 SF	6.6	2.55E-03					8.3	2.23E-05			
ADAMTS4	Metalloprotease; Pep M12B Propep SF; ZnMc SF; ADAM CR SF; TSP 1 SF	2.6	4.92E-03									
ADAMTS7	Metalloprotease; Pep M12B Propep SF; ZnMc SF; ADAM CR SF; TSP 1 SF	1.6	3.54E-02									
ADAMTS9	Metalloprotease; Pep M12B Propep SF; ZnMc SF; ADAM CR SF; TSP 1 SF	1.7	4.78E-02									
B3GNT7	Keratan Sulfate Biosynthesis (100739508); Galactosyl T SF										6.4	1.16E-05
BGN	Alarmin (24257755); Collagen Fibril Assembly; LRRNT SF							1.8	4.69E-02		2.5	1.81E-02
BLM	Peptidase C1 SF							1.7	3.03E-02			
COL12A1	Collagen SF	2.1	1.41E-04		1.6	4.13E-02						
COL26A1	Collagen SF	7.3	2.71E-04					5.8	6.82E-05			
COL28A1	vWFA SF; Collagen SF; KU SF	4.7	5.24E-04					4.0	1.89E-04		4.9	5.31E-06
COL4A1	Collagen SF	1.7	1.53E-02									
COL5A2	Collagen SF	1.6	1.79E-02									
COL6A3	Collagen SF	1.8	3.31E-02									
COL7A1	Collagen SF; KU SF; vWFA SF	2.1	1.26E-03					1.9	1.47E-03			
COL8A1	C1q SF; Collagen SF	4.1	1.19E-06		2.0	3.85E-02		2.0	1.40E-02			
COMP	ECM Protein; TSPcc SF; EGF CA; SF; TSP 3 SF; TSP C SF											
CTGF	Wound Healing (19128254); IGFBP SF; VWC SF; TSP 1 SF; GHB-like SF	2.1	2.04E-02									
CTSG	Cathepsin; Tryp SPc SF	3.3	1.58E-02		7.2	8.45E-07						
DSE	Dermatan Sulfate Biosynthesis (19687302); DUF4962 SF	1.9	4.61E-03									
ECM1	ECM Protein; ECM1 SF	1.9	4.06E-03					1.8	3.23E-03			
FGF2	Wound Healing (19128254); FGF SF											
FGF7	(+) Regulator of Enterocyte Differentiation (19326389); FGF SF	3.2	4.44E-05					2.4	6.47E-04		2.6	5.67E-04
HPN	Serine Protease (16908524); Hepsin-SRCR SF; Tryp SPc SF											
KRT18	Enterocyte Differentiation Marker (22943429); Intermediate Filament Protein; Keratin SF	2.6	4.34E-05					2.0	1.04E-03		4.7	7.48E-10
KRT80	Intermediate Filament Protein; Keratin SF	1.8	4.15E-02									
LAMB3	Basement Membrane Protein; Laminin N SF; EGF Lam SF	2.5	2.26E-06					2.4	1.73E-07		3.0	6.87E-04
LAMC1	ECM Glycoprotein (22961762); Laminin N SF; Laminin B SF	1.6	2.40E-02									
LOXL4	Collagen Biosynthesis; SRCR SF; Lysyl Oxidase SF	2.2	3.47E-02									
MMP1	Metalloprotease; M1-associated (22880008); HX SF; ZnMc SF; PG-binding-1 SF	9.4	4.44E-05					3.1	2.86E-02		7.3	6.53E-13
MMP12	Metalloprotease; Th2/M2-Associated (12842850, 22880008); HX SF; ZnMc SF	101.0	2.26E-14		7.3	1.09E-03		13.9	7.63E-10		9.7	4.45E-16
MMP13	Metalloprotease; M2-Associated (12842850); HX SF; ZnMc SF	20.2	1.77E-06					11.4	2.65E-07		27.5	9.71E-29
MMP19	Metalloproteinase; HX SF; ZnMc SF; PG-binding-1 SF	1.8	4.54E-02									
MMP20	Metalloproteinase; M2-associated (22880008); HX SF; ZnMc; SF; PG-binding-1 SF	7.4	4.14E-02									
MMP3	Metalloproteinase; M1-associated (22880008); HX SF; ZnMc SF; PG-binding-1 SF	3.5	3.34E-02								4.9	3.31E-10
MMP7	Metalloproteinase; M1-associated (22880008); PG binding 1 SF; ZnMc SF	33.4	3.22E-03					10.5	7.62E-03		53.1	4.09E-05
MMP8	Metalloprotease; M2-associated (26092731); PG binding 1 SF; ZnMc SF; HX SF	27.1	7.94E-03					11.4	3.47E-03			
MMP9	Metalloprotease; M2-associated (26377769); PG Binding 1 SF; ZnMc SF; PT SF; HX SF	8.9	1.02E-08		3.4	3.03E-03		2.6	6.92E-03		9.3	1.00E-02
P4HA2	Positive Regulator of Collagen Biosynthesis (9211872); P4Ha N SF; 2OG-FeII Oxy 3 SF	1.7	5.38E-03					1.6	3.60E-03			
P4HA3	Positive Regulator of Collagen Biosynthesis (14500733); P4Ha N SF; 2OG-FeII Oxy 3 SF	4.4	3.89E-04					3.7	1.39E-04			
PLAUR	Wound Healing (15769664); LU SF	4.6	3.65E-06					2.8	4.01E-04		4.8	6.11E-06
PLOD1	Positive Regulator of Collagen Biosynthesis (25277362); 2OG-FeII Oxy 3 SF											
PRSS27	Wound Re-epithelialization (18948266); Tryp SPc SF											
PSMB7	Proteasome subunit; NTN Hydrolase SF										1.9	7.05E-03
PSMD1	PC Rep SF	1.5	3.59E-02									
PSMD14	26S Proteasome Subunit; MPN SF											
PSMD5	26S Proteasome Subunit; PSMB SF											
SERPINA1	Serine Protease Inhibitor; SERPIN SF	4.7	5.81E-03					4.5	9.03E-04			
SERPINA3	Serine Protease Inhibitor; SERPIN SF											
SERPINA3-2	Serine Protease Inhibitor; SERPIN SF	60.8	2.96E-06					16.2	1.05E-06			
SERPINB1	Serine Protease Inhibitor; SERPIN SF	1.6	3.13E-02					1.6	1.75E-02			
SERPINB10	Serine Protease Inhibitor; SERPIN SF										3.5	2.87E-04
SERPINB11	Serine Protease Inhibitor; SERPIN SF	11.3	9.04E-04					6.3	3.20E-03		8.4	7.38E-04
SERPINB2	Antiparasitic Response (23630350) M2-associated (23355731); SERPIN SF	9.8	2.12E-04					3.9	1.24E-02		16.2	4.90E-32
SERPINB5	Serine Protease Inhibitor; SERPIN SF	1.9	8.72E-03					2.0	9.60E-04		2.2	1.56E-03
SERPINB6	M2-associated (23293084); Serine Protease Inhibitor; SERPIN SF										2.0	1.67E-02
SERPINB7	Extracellular Matrix Degradation (18580857); Serine Protease Inhibitor; SERPIN SF	19.3	1.85E-02									
SERPINB8	Serine Protease Inhibitor; SERPIN SF	2.7	2.47E-05					2.2	2.45E-04		1.8	2.66E-02
SERPINE2	Serine Protease Inhibitor; SERPIN SF	3.1	1.06E-04					2.0	1.59E-02			
SERPINH1	Serine Protease Inhibitor; SERPIN SF	2.0	7.17E-03		1.8	4.70E-02						
SPINK4	Serine Protease Inhibitor; KAZAL FS	6.6	2.11E-06					5.8	1.64E-08		8.1	9.43E-22
SPINK6	Serine Protease Inhibitor; KAZAL FS	46.2	5.38E-03					21.5	4.16E-04			
SULF2	Heparan Sulfate Endosulfatase; Sulfatase SF; DUF3740 SF	1.9	2.64E-02									
TFPI2	Serine Protease Inhibitor; KU FS	1.7	5.42E-03									
TGM3	Transglutaminase SF							3.0	3.20E-02		10.0	3.03E-05
TIMP1	Metallopeptidase Inhibitor; M2-associated (21926236); NTR-like SF	4.3	2.48E-09					2.9	2.65E-07		3.9	2.94E-04
TNC	ECM Protein; FReD SF; FN3 SF	2.9	8.70E-04									
TNN	ECM Protein (10842355)	61.3	4.18E-05		83.7	5.30E-05						
VCAN	ECM Protein; CLECT SF; EGF CA SF; Ig SF; Link Domain SF											
	**Pattern (PR) and Damage-associated Recognition (DR) Receptors**											
BGN	Alarmin (24257755); LRRNT SF							1.8	4.69E-02		2.5	1.81E-02
CD14	PRR (7534618); LRR RI	2.1	1.23E-02					2.0	7.89E-03			
CD163	PRR (18849484); M2b-associated (15491976); SRCR SF	1.9	4.34E-02					1.9	2.29E-02			
CLEC10A	M2-associated (15591125); PRR (15802303); CLECT SF	3.6	2.24E-02					2.9	3.05E-02			
CLEC18A	PRR (26170455); CLECT SF	29.5	7.94E-03					46.3	9.53E-05			
CLEC4D	PRR (25674984); CLECT SF	13.4	4.10E-02									
DDX21	PRR (21703541); GUCT SF; DEXDc SF; EIF-3 Zeta SF	1.5	2.23E-02									
FCN2	Complement Activation (19632990); PRR (15331601); FReD SF							2.5	2.74E-03		2.9	9.94E-05
HMGB1	Alarmin (16966386); HMG-box SF							1.5	1.59E-02			
MPTX	Mucosal pentraxin; LamG SF	5.8	4.12E-06					4.9	2.10E-07		8.3	4.03E-15
SFTPD	PRR (15950451); CLECT SF	4.1	2.73E-02									
SNCA	Alarmin (21747756); Synuclein SF	2.7	1.40E-02					2.7	4.96E-03			
TREM1	PRR (25595774); Ig SF	6.4	4.61E-03									
TREM2	PRR (12847223); Ig SF				-2.5	4.89E-02		3.0	7.72E-03			
	**Mast Cell Associated**											

SRGN	Mast Cell Secretory Granule Protein (15231821); Serglycin SF	1.8	2.61E-03		2.1	1.18E-05						
MCPT3*	Mast Cell Protease; Tryp SPc SF	7.3	2.80E-02									
TPSB2	Mast Cell Protease; Tryp SPc SF										2.8	2.68E-02
HDC	Biosynthesis of Histamine; Mast Cell Associated; AAT I SF				2.7	2.39E-02						
KIT	(+) Positive Regulator of Mast Cell Development (25062998); Ig SF PKC-like SF				2.3	1.73E-02						
	**M2-Macrophage Associated**											
ARG1	Antiparasitic Response (19764983); M2-associated (5879104); Arginase HDAC SF	122.6	4.56E-05					16.6	8.41E-04		30.1	2.28E-07
CD163	PRR (18849484); M2b-associated (15491976); SRCR SF	1.9	4.34E-02					1.9	2.29E-02			
CHIA	Antiparasitic Response (15618176); Th2-Associated (11553626); GH18 Chitinase Like SF	2.3	3.56E-02									
CLEC10A	M2-associated (15591125); PRR (15802303); CLECT SF	3.6	2.24E-02					2.9	3.05E-02			
F13A1	Coagulation; M2-associated (19105661); Transglutaminase SF	4.4	1.41E-09		2.1	7.07E-03		2.1	1.19E-03			
IL1R2	IL1B Decoy Receptor; M2-associated (8786316); I-set SF; Ig SF	10.3	2.46E-06					3.8	1.61E-03		6.1	1.51E-06
IRAK3	IL-1 Receptor-associated Kinase; M2-associated (2109822); DD SF; PKC-like SF											
IRF4	Antiparasitic Response (20729857); M2-associated (20729857); IRF TF SF	2.1	2.55E-03									
MMP12	Metalloprotease; Th2/M2-Associated (12842850, 22880008); HX SF; ZnMc SF	101.0	2.26E-14		7.3	1.09E-03		13.9	7.63E-10		9.7	4.45E-16
MMP13	Metalloprotease; M2-Associated (12842850); HX SF; ZnMc SF	20.2	1.77E-06					11.4	2.65E-07		27.5	9.71E-29
MMP20	Metalloproteinase; M2-associated (22880008); HX SF; ZnMc; SF; PG-binding-1 SF	7.4	4.14E-02									
MMP8	Metalloprotease; M2-associated (26092731); PG binding 1 SF; ZnMc SF; HX SF	27.1	7.94E-03					11.4	3.47E-03			
MMP9	Metalloprotease; M2-associated (26377769); PG Binding 1 SF; ZnMc SF; PT SF; HX SF	8.9	1.02E-08		3.4	3.03E-03		2.6	6.92E-03		9.3	1.00E-02
ODC1	M2-associated (19726720); Ornithine Metabolism; Polyamine Metabolism; PLPDE III SF				-1.5	1.27E-02		1.6	4.12E-03			
OSM	M2a-associated (26801095); LIF OSM SF	4.4	1.92E-04					2.4	1.97E-02			
PTGS1	Prostaglandin E2 Biosynthesis; M2-associated (17082649); EGF CA; An Peroxidase-like SF	2.3	9.54E-06		1.7	6.80E-03						
PTGS2	Prostaglandin H2 Biosynthesis; M2-associated (28684424); EGF CA; An Peroxidase-like SF	6.5	8.39E-06					3.4	8.84E-04		4.0	1.30E-07
SERPINB2	Antiparasitic Response (23630350) M2-associated (23355731); SERPIN SF	9.8	2.12E-04					3.9	1.24E-02		16.2	4.90E-32
SERPINB6	M2-associated (23293084); Serine Protease Inhibitor; SERPIN SF										2.0	1.67E-02
SLC7A2	Arginine Transport (8954799), M2-associated (16670299); Solute Carrier SF	1.9	4.15E-02									
TGM2	M2-associated (23293084); Transglutaminase SF	2.2	3.66E-05								2.2	7.15E-03
TIMP1	Metallopeptidase Inhibitor; M2-associated (21926236); NTR-like SF	4.3	2.48E-09					2.9	2.65E-07		3.9	2.94E-04
TREM2	PRR (12847223); Ig SF				-2.5	4.89E-02		3.0	7.72E-03			
	**Mucus and Fluid Production**											
A3GALT2	Galactosyltransferase; Glyco Tranf GTA Type SF	40.6	5.54E-03					9.7	1.38E-02			
AGR2	Mucin Biosynthesis (24942678); Thioredoxin-like SF	6.5	1.79E-05					5.7	6.15E-07		5.6	1.73E-11
AQP3	Glycerol Transport (16079275); Water Transport (16079275); MIP SF											
AQP5	Water Transporter (9195910); MIP SF	33.5	4.54E-04					10.2	1.63E-03			
AQP9	Water Transport; Glycerol Transport (19096786); MIP SF	5.3	1.56E-02									
B3GNT6	Mucin Biosynthesis (26274979); Galactosyl T SF	3.0	2.31E-03					2.9	5.44E-04		2.3	7.21E-03
CLCA1	Chloride Ion Transport (8769986); Th2-associated (17898169); CLCA N SF; vWFA SF	3.2	1.07E-03					2.8	6.25E-04		3.5	9.80E-09
FUT2	Fucosyltransferase (24062455); Th2-associated (12652082); O-FucT-like SF										3.1	1.38E-03
FUT8	Mucin Biosynthesis (28122822); O-FucT-like SF; SH3 SF	1.6	5.97E-03									
GALNT1	Mucin Biosynthesi; Glyco Tranf GTA-type SF; RICIN SF	1.5	4.20E-02									
GALNT6	Mucin Biosynthesis (20215525); Glyco Tranf GTA-type SF	1.7	1.20E-02					2.2	3.33E-06		2.5	5.55E-03
GALNT7	Mucin Biosynthesis Glyco Tranf GTA-type SF; RICIN SF	1.9	5.18E-04					1.7	2.49E-03			
GCNT3	IL-13 Induced (17303715); Mucin Biosynthesis (20816165); Branch SF	2.7	1.21E-03					2.1	7.34E-03		2.4	4.06E-04
MUC2	Secreted Gel Forming Mucin (24942678); Th2-associated (14594655); Mucin SF	5.2	7.10E-08					3.8	1.60E-07		6.3	2.16E-16
	**Miscellaneous**											
ANXA8	Anticoagulant (2530088); Annexin SF	21.8	2.21E-07					7.6	1.18E-05		34.8	6.97E-40
CHI3L1	GH18 Chitinase Like SF	13.9	2.38E-06					5.2	2.51E-04		6.4	1.03E-13
CHI3L2	GH18 Chitinase Like SF	6.0	7.37E-04								4.2	4.97E-04
CHIT1	GH18 Chitinase Like SF	24.2	1.65E-02									
CLTC	(-) Regulator of NFk-B Activation (21364927); Clathrin SF	1.5	3.32E-02									
FCGBP	Ig Receptor; VWD SF; C8 SF; TIL SF; VWC SF	2.3	2.08E-02									
GJA1	Epithelial Wound Repair (19528242); Connexin SF; Connexin CCC SF; Connexin 43 SF	2.4	6.11E-05									
GZMA	Tryp SPc SF				3.0	4.54E-04						
GZMB	Tryp SPc SF	5.5	2.79E-04		11.2	8.54E-08						
IGH@	Ig SF; Ig Heavy Chain Constant Region SF	5.7	2.70E-05					2.6	1.24E-02			
IGHG1	Ig SF; Ig Heavy Chain Constant Region SF	29.1	1.93E-04					10.3	8.11E-04		11.1	3.80E-06
IGJ	Immunoglobulin J polypeptide; Ig J-Chain SF	1.8	2.63E-02									
KLRJ1*	CLECT SF	10.0	5.18E-04		9.6	1.59E-03						
MYOF	Membrane Regeneration and Repair; C2 SF; FerA SF; FerB SF; DysFN SF	2.2	3.03E-05					1.9	1.39E-04			
NEUROG3	(+) Regulator of Enteroendocrine Cell Differentiation (17706959); HLH SF	5.7	1.61E-03					3.8	3.07E-03			
NLRC3	Inflammasome Associated (21191067); LRR RI SF; P-loop NTPase SF				2.5	1.97E-02						
NTRK1	Allergic Inflammation (25389033); IL-4 Induced; Ig SF; LRRCT SF	42.0	2.62E-07		10.6	3.53E-03						
PADI1	(-) Regulator of Inflammation (18710930); PAD SF	296.4	3.51E-03					51.6	6.25E-04			
PADI3	PAD SF	55.7	1.75E-02					19.0	4.69E-03			
PRG4	HX SF; Somatomedin B SF	10.7	1.39E-02					13.5	3.43E-04			
S100A10	Inflammation (16373665); S100 SF											
S100A11	RAGE Ligand (18331229); S100 SF							1.5	3.35E-02			
S100A12	RAGE Ligand (18331229); Inflammation (12740341); S100 SF	38.4	4.48E-04					13.4	7.36E-04		12.8	4.09E-05
S100A2	Eosinophil Chemoattractant (8607858); S100 SF	7.4	9.54E-06					4.2	1.75E-04		10.0	3.03E-27
S100A3	S100 SF	8.0	6.29E-03					4.5	2.35E-02			
S100A6	RAGE Ligand (18331229); S100 SF	1.6	3.06E-02					1.6	1.55E-02			
S100A8	RAGE Ligand; Inflammation (12645005); S100 SF	38.3	5.18E-04					10.8	2.17E-03		8.2	1.02E-02
S100A9	RAGE Ligand; Inflammation (12645005); S100 SF	44.8	2.59E-04					11.0	2.03E-03		9.8	1.83E-03
SCGB2A2	Lipophilin SF Secretoglobin SF; Uteroglobin SF	105.1	3.14E-07					17.9	4.89E-06			
SCGB3A1	IL-4 Induced (16237061); Lipophilin SF Secretoglobin SF; Uteroglobin SF	8.8	1.90E-02									
SMPDL3B	(-) Regulator of TLR Signaling (26095358); MPP SF										2.0	1.67E-02
SOCS3	TH2-associated (11907070); Th17-associated (16698929)	2.5	1.07E-03									
THBD	Th2 Associated (189478630); CLECT SF; EGF CA SF; Tme5 EGF-like SF	1.8	2.09E-02					1.9	3.32E-03			
TIGIT	(-) Regulator of DC Function (19011627); TFH Cell Associated (19197944); Ig SF	2.5	3.55E-03		2.8	3.39E-04						
TNIP3	(-) Regulator of TLR4 Signaling (17088249); Tape Meas Lam C SF; BAN SF	7.3	4.56E-05					3.5	3.16E-03		4.5	5.90E-06
TRAIP	(-) Regulator of TLR Signaling (14676304); RING Ubox SF							1.9	5.08E-03			
VSTM1	(-) Regulator of Monocyte/Neutrophil Function (20375307, 23436183); Ig SF	2.3	1.95E-02					2.3	7.89E-03			
WNT7A	Wound healing (15802269); WNT SF											
XDH	(+) Regulator of Inflammation (h) (20632067); Fer2 2 SF; CO Deh Flav C SF; Ald Xan Dh C2 SF	22.8	3.03E-02									
	**Neurotransmitters and Receptors**											
ABAT	GABA aminotransferase; AAT I SF	2.7	2.61E-02					3.1	1.97E-03		3.2	1.73E-05
AVPR1B	Arginine Vasopressin Receptor; GPCR SF	62.6	5.42E-03									
BDKRB1	Bradykinin Receptor; GPCR SF	4.0	1.65E-03								4.6	1.70E-02
DOK7	Postsynaptic Differentiation; PH-like SF											
DRD2	Dopamine receptor D2; Neurotransmitter Receptor; GPCR SF											
GABRD	GABA Receptor; Neurotransmitter Receptor; Neur Chan LBD SF; Neur Chan Memb SF	2.5	4.65E-02					3.4	4.54E-04			
GABRP	GABA Receptor; Neur Chan LBD SF Neur Chan Memb SF										15.7	1.59E-03

GPR83	Neuropeptide Receptor (27117253); Treg Associated (16785516); GPCR SF											
HTR1B	Neurotransmitter Receptor; Serotonin Receptor; GPCR SF										11.8	2.40E-02
MCHR1	Neuropeptide Receptor; GPCR SF	17.9	7.59E-03									
P2RX4	Purinergic Receptor; Inflammasome Associated (24409119); P2X Receptor SF	1.5	4.22E-02					1.6	4.88E-03		2.4	8.14E-04
SLC35E1	Sodium-dependant GABA Transport; Solute Carrier SF											
SLC6A13	Sodium-dependant GABA Transport; Solute Carrier SF	6.5	1.54E-02									
SLC6A2	Sodium-dependent Noradrenaline Transport (2008212); Solute Carrier SF							13.1	2.14E-02		18.7	1.53E-02
SV2B	Neurotransmitter Release; MF SF											
	**Oxidative Stress Response**											
GSTA1	Glutathione S-Transferase; Thioredoxin-like SF; GST C Family SF										2.7	4.73E-02
GSTA2	Glutathione S-Transferase; Thioredoxin-like SF; GST C Family SF										5.4	4.30E-03
GSTP1L2*	Glutathione S-Transferase; Thioredoxin-like SF; GST C Family SF										6.9	3.53E-02
PDIA4	Superoxide Generation (1318337); Thioredoxin-like SF	2.3	1.15E-04					1.7	7.74E-03			
PRDX4	Oxidative Stress Response (12080185); Thioredoxin-like SF	2.5	3.41E-05					1.9	1.03E-03			
	**Prostaglandin and Leukotriene**											
ALOX5AP	MAPEG SF				2.0	3.09E-02						
LTC4S	Cysteinyl Leukotriene Biosynthesis; Th2-Associated (11136826); MAPEG SF				2.8	1.24E-02						
PTGER1	Prostaglandin E2 Receptor; Th1 Associated (17967902); GPCR SF	2.3	1.21E-02					2.4	2.11E-03			
PTGER2	Prostaglandin E2 Receptor; Inflammation (15075356); GPCR SF	1.7	1.84E-03									
PTGS1	Prostaglandin E2 Biosynthesis; M2-associated (17082649); EGF CA; An Peroxidase-like SF	2.3	9.54E-06		1.7	6.80E-03						
PTGS2	Prostaglandin H2 Biosynthesis; M2-associated (28684424); EGF CA; An Peroxidase-like SF	6.5	8.39E-06					3.4	8.84E-04		4.0	1.30E-07
PLA2G4A	Prostaglandin Metabolism; C2 SF; Patatin and cPLA2 SF	1.7	3.82E-02									
	**Surface Receptors and Ligands**											
ADGRE1	Th2-associated (20625511); GPCR SF							2.8	3.05E-02			
ADGRE2	(+) Regulator of Macrophage Differentiation (28421075); GPCR SF	2.1	2.00E-02									
ADGRG6	(+) Regulator of Angiogenesis (25217645); GPCR SF	1.9	2.65E-03					1.6	2.36E-02			
ADORA1	(+) Regulator of Inflammation (8360491); GPCR SF											
ASGR2	CLECT SF							2.1	3.46E-02			
CD24	(+) Regulator of T Cell Activation (15607804); SLAM SF Immunoglobulin SF	1.8	4.34E-02					1.9	6.90E-03			
CD244	(+) Regulator of T Cell Activation (15607804); SLAM SF				2.4	1.29E-02						
CD69	(+) Regulator of Inflammation (16983725); CLECT SF	1.8	3.37E-02		2.1	4.85E-03						
CD79A	B Cell Maturation Marker (15928681); Ig SF	2.2	1.05E-02									
CDH1	(+) Regulator of Paneth Cell Differentiation (21179475); Cadherin SF	2.1	1.78E-04					1.6	8.68E-03		2.2	3.28E-03
F13A1	Coagulation; M2-associated (19105661); Transglutaminase SF	4.4	1.41E-09		2.1	7.07E-03		2.1	1.19E-03			
F2	Coagulation; Th2-Associated (16081830); GPCR SF											
F2RL2	Coagulation; Positive Regulator of Inflammation (12805069, 18264801); GPCR SF	1.7	1.57E-02									
F3	Coagulation; FN3 SF; Tissue Factor SF	8.2	7.74E-11					5.2	1.85E-10		3.1	4.09E-05
ITGA1	T Cell Activation Marker; vWFA SF; VCBS SF; Integrin Alpha2 SF	1.9	3.71E-02									
ITGA2	Adhesion Molecule; VCBS SF; vWFA SF; Integrin Alpha2 SF	2.6	3.66E-05					2.2	1.50E-04			
ITGA8	Integrin; VCBS Superfamily	2.2	1.15E-04									
ITGAV	Integrin; FG-GAP Superfamily; Integrin Alpha2 Superfamily	1.7	7.96E-03									
ITGB1	Integrin; vWFA Superfamily; Integrin B Cyt Superfamily; Integrin B tail Superfamily	1.5	1.80E-02									
ITGB6	(+) Regulator of Inflammation (10025398); Integrin Beta SF; vWFA SF	5.4	8.74E-11					3.1	2.10E-07		2.6	7.63E-03
NECTIN1	Adhesion Molecule; Ig SF							1.5	3.13E-02			
SELE	Adhesion Molecule; CLECT Superfamily; EGF-CA Superfamily; CCP Superfamily				2.5	3.53E-02						
SEMA7A	(-) Regulator of T Cell Activation (16713976); Sema SF; PSI SF	3.0	1.23E-05					2.1	1.51E-03		2.1	2.41E-02
	**Transcription Factors**											
ARNTL2	HLH SF; PAS SF	3.8	6.51E-06		2.1	3.38E-02		1.8	5.25E-02			
ATF4	bZIP SF	1.6	1.20E-02									
ATF5	bZIP SF	2.5	2.26E-02									
ATOH1	(+) Regulator of Enterocyte/Enteroendocrine Cell Differentiation (17570220, 15044355); HLH SF							1.7	2.37E-02		2.0	2.41E-02
CDX1	(+) Regulator of Enterocyte Differentiation (10579974); Caudal Act SF; Homeodomain SF										2.7	1.73E-03
CEBPB	Th2-Associated (16585579); bZIP SF	2.1	3.29E-03									
E2F7	E2F TDP SF	1.7	5.49E-02					1.8	1.34E-02			
EPAS1	HIF-1 SF				1.5	1.29E-02						
ETV4	ETS SF	2.9	3.43E-04					2.7	1.36E-04		3.1	4.03E-02
ETV5	ETS SF	1.9	2.26E-02					1.8	1.26E-02			
FOS	bZIP SF	2.5	2.09E-02									
FOXA1	DAZAP2 SF; FH SF; HNF C SF	2.0	6.53E-03					1.6	3.80E-02		1.8	3.01E-02
GATA2	(+) Regulator of Eosinophil Differentiation (9279013); ZnF GATA SF				2.0	5.31E-02						
GATA3	(+) Regulator of Th2-Cell/ILC2 Differentiation (19375293, 23733962); ZnF GATA TF SF				4.4	4.43E-04						
HIF1A	Transcription Factor; HLH SF; PAS SF; HIF-1 SF	2.4	9.56E-06					1.7	3.73E-03			
HOXB13	Development; Homeodomain SF										132.7	1.59E-02
HOXD1	Development; Homeodomain SF	7.4	6.83E-03					4.6	9.07E-03			
IRF4	Antiparasitic Response (20729857); M2-associated (20729857); IRF TF SF	2.1	2.55E-03									
MAZ	(+0 Regulator of Acute Phase Response (16888022); zf-H2C2 2 SF; RING SF											
MKX	Development; Homeodomain SF; IRO SF	2.3	2.40E-02									
MYBL1	SANT SF; Cmyb C SF							2.2	4.61E-02			
MYBL2	(+) Regulator of Enterocyte Differentiation (20857481); SANT SF; Cmyb C SF							1.5	3.16E-02		2.4	3.34E-03
NFIL3	(+) Regulator of ILC Development (27705792); bZIP SF	2.4	3.14E-09		1.7	1.50E-04						
NKX2-2	(+) Regulator of Enteroendocrine Cell Differentiation (18022152); Homeodomain SF	3.3	5.22E-03					2.8	5.20E-03			
POU2AF1	Transcriptional Co-activator; PD-C2-AF1 SF	3.3	9.54E-06					2.4	1.75E-04			
POU5F1	Transcription Factor; Homeodomain SF							19.0	3.78E-02			
PRDM1	(+) Regulator of Enterocyte Reprogramming (21670299); SET SF	1.6	2.16E-02									
RUNX3	(+) Regulator of ILC1/ILC3 Development (26414766); Runxl SF				2.2	5.12E-03						
SOX9	(+) Regulator of Paneth Cell Differentiation (17698607); Sox N SF; HMG-box SF	1.7	1.52E-02									
SP6	zf-H2C2 2 SF; zf-H2C2 2 SF; COG5048 SF										9.3	1.01E-02
SPDEF	(+) Regulator of Goblet Cell/Paneth Cell Differentiation (19786015, 19549527); ETS SF	4.4	3.89E-06					3.5	2.29E-06		3.0	1.13E-04
TLE6	Transcriptional Co-repressor (17624551); WD40 SF	3.0	6.63E-03					3.2	5.27E-04		4.4	1.34E-02
XBP1	B Zip1 SF	2.0	2.73E-04					1.7	3.59E-03			
ZNF215	Transcriptional Co-repressor; SCAN SF; KRAB A-box SF; zf-H2C2 2 SF; zf-C2H2 SF							2.1	2.39E-02			
