# Supplementary material for: Molecular and metabolomic changes in the proximal colon of pigs infected with Trichuris suis
Source: Sci Rep. 2020 Jul 30;10:12853. doi: 10.1038/s41598-020-69462-5 (PMC7393168; doi:10.1038/s41598-020-69462-5)
Supplement: Supplementary file 8 — Supplementary Table S8. [file 41598_2020_69462_MOESM8_ESM.pdf]

Molecular and metabolomic changes in the proximal colon of pigs infected with *Trichuris suis*

Harry Dawson[1], Celine Chen[1], Robert Li[2], Lauren Nicki Bell[3], Terez Shea-Donohue[4], Helene Kringel[5], Ethiopia Beshah[1], Dolores E. Hill[2], Joseph F. Urban Jr[1,2].

[1]United States Department of Agriculture, Agricultural Research Service, Northeast Area, Beltsville Human Nutrition Research Center, Diet Genomics and Immunology Laboratory, [2] Beltsville Agricultural Research Center, Animal Parasitology Disease Laboratory, Beltsville, MD; [3]Metabolon, Inc., Morrisville, NC; [4]University of Maryland School of Medicine, Baltimore, MD, USA; [5]Department of Veterinary Disease Biology, Faculty of Health and Medical Sciences, University of Copenhagen, Copenhagen, Denmark

Supplemental Table S8

Supplemental Table S8: Clustered Down-regulation of Physiology and Immunology DEGs

Gene	Functional Categorization	52 DPI w worms vs Control Fold	EDGE FDR Adj p value		52 DPI w/o worms vs Control Fold	EDGE FDR Adj p value		(52 DPI Inf w/o worms vs w Worms Fold	EDGE FDR Adj p value		(21 DPI Inf vs Control) Fold	EDGE FDR Adj p value
	**Antigen Processing and Presentation**											
ADAM23	Positive Regulator of Antigen Presentation (27317750); ZnMc SF											
B2M	Positive Regulator of Surface Expression of MHC Class I (16181333); Ig SF	-2.0	5.66E-04									
CD74	MHC Chaperone (22306692); MHC2-interact SF	-2.5	2.92E-04					-2.0	7.72E-03			
CIITA	Positive Regulator of MHC Class I and II Expression (14678199, (11486010); LRR SF							-1.7	4.54E-02			
CLEC4F	Glycolipid Antigen Presentation (23762286)	-4.6	4.26E-02									
CTSD	Antigen Processing and Presentation (16920965); Pepsin Retropepsin-like SF	-2.1	3.64E-04					-1.6	1.68E-02			
ERAP2	Antigen Processing and Presentation (12799365); Type 2 IFN Induced (12799365); GluZincin SF	-1.6	4.60E-02									
LGALS3BP	Antigen Processing and Presentation (15231701); SRCR SF	-1.9	3.18E-02									
PLCB2	(+) Regulator of B Cell Activation (20871625); PH-like SF; EF-hand-like SF; PI-PLCc GDPD SF SF; C2 SF	-2.0	1.74E-03									
PSMB10	Immunoproteasome Protease (9551082); Ntn Hydrolase SF; Pr Beta C SF	-1.8	3.20E-03									
PSMB8	Immunoproteasome Protease; Ntn Hydrolase SF; Pr Beta C SF	-1.6	2.91E-02									
PSMB9	Immunoproteasome Protease; Ntn Hydrolase SF; Pr Beta C SF	-1.8	9.65E-03									
PSME1	Antigen Processing and Presentation (12200048); PA28 Alpha SF; PA28 Beta SF	-1.6	5.26E-03									
SLA-11	MHC Class I SF	-2.3	1.08E-04					-1.6	4.46E-02			
SLA-2	MHC Class I SF							-1.7	5.02E-02			
SLA-3	MHC Class I SF	-2.3	1.89E-04								-1.8	3.72E-02
SLA-8	MHC Class I SF	-1.6	1.98E-02					-1.6	4.54E-02			
SLA-DMA	MHC Class II SF	-2.1	4.61E-03								-1.9	1.99E-02
SLA-DMB	MHC Class II SF	-2.2	8.10E-04					-1.7	2.70E-02		-2.4	3.61E-04
SLA-DOA	MHC Class II SF	-2.4	3.71E-03									
SLA-DQA1	MHC Class II SF	-2.3	3.27E-03					-2.0	1.33E-02			
SLA-DQB1	MHC Class II SF	-2.3	1.13E-02								-2.5	1.55E-03
SLA-DRA	MHC Class II SF	-2.4	1.06E-03					-2.1	5.53E-03			
SLA-DRB1	MHC Class II SF	-2.2	5.60E-03					-2.0	1.25E-02			
TAP1	Peptide Transporter; ABC Membrane SF	-1.9	5.73E-03					-1.7	2.23E-02			
TAP2	Peptide Transporter; ABC Membrane SF	-1.6	1.31E-02									
	**Cytokines, Chemokines and Receptors**											
ADIPOQ	Adipocytokine; C1q SF	-4.2	5.00E-04								-2.2	4.06E-03
ANGPTL4	Adipocytokine; FReD SF	-2.9	1.19E-03		-2.0	4.70E-02						
BMP3	Bone Morphogenetic Protein (BMP) Family; TGFB SF										-2.5	2.94E-03
CCL2	CCR2 Ligand; CCL Chemokine SF										-2.0	1.38E-03
CCL21	CCR7 Ligand; CCL Chemokine SF	-1.7	4.86E-02									
CCL3L1	CCL Chemokine SF										-5.6	2.84E-03
CCL3L2	CCL Chemokine SF	-5.4	6.08E-03								-10.6	5.90E-03
CCL4	CCR5 Ligand; CCL Chemokine SF	-2.4	1.16E-02									
CCL8	CCR1 Ligand; CCL Chemokine SF	-2.6	7.80E-03								-2.8	4.24E-02
CCR5	CCL3 Receptor; CCL4 Receptor; CCL5 Receptor; CCL Chemokine Receptor SF	-1.8	5.37E-03									
CCR9	CCL25 Receptor; CCL Chemokine Receptor	-3.0	5.47E-02								-5.8	1.14E-02
CNTFR	Cytokine Receptor (10812968); FN3 SF; Ig SF	-3.5	4.40E-04					-2.9	3.11E-03			
CSF1	Cytokine; Positive Regulator of Macrophage Development.	-1.5	3.58E-02					-1.5	1.68E-02			
CSF1R	CSF1 Receptor	-1.7	3.70E-03					-1.5	4.54E-02			
CSF2RB	GM-CSF/IL-3/IL-5 receptor Common Beta-Chain; Erythropoietin Receptor SF; FN3 SF; IL6Ra-bind SF							-1.5	4.36E-02			
CXCL10	Type 2 IFN Induced; CXCR3 Ligand; CXC Chemokine SF	-3.4	3.22E-02					-4.4	7.18E-03			
CXCL11	Type 2 IFN Induced; CXCR3 Ligand; CXC Chemokine SF	-4.8	3.33E-02									
CXCL12	CXCR4 Ligand; CXC Chemokine SF	-2.7	6.31E-04					-2.8	2.48E-04		-2.3	5.03E-03
CXCL13	CCL10 Ligand; CXCR5 Ligand; CXC Chemokine SF										-5.8	5.77E-08
CXCL9	Type 2 IFN Induced; CXCR3 Ligand; CXC Chemokine SF	-7.5	6.02E-04					-5.7	3.65E-03		-6.1	7.08E-08
CXCR3	CXCL9 Receptor; CXCL10 Receptor; CXCL11 Receptor; CXCL Chemokine Receptor SF	-2.2	1.38E-02									
EBI3	FN3 SF	-2.0	4.52E-02									
FLT3LG	Flt3 Lig SF	-1.8	3.71E-03									
GDF5	Bone Morphogenetic Protein (BMP) Family; TGFB SF	-3.8	1.25E-02		-3.3	1.09E-03						
IGF2	IIGF-like SF	-3.2	5.66E-04					-2.2	2.80E-02			
IL10RA	Cytokine Receptor; Interferon-binding SF	-1.9	8.53E-04					-1.5	3.17E-02			
IL12B	Cytokine; IL12p40 SF	-4.7	1.02E-02									
IL12RB2	IL-12 Receptor; Th1-Associated (10666772); Lep Receptor Ig SF	-2.3	4.34E-02									
IL15	TH1-Associated (12759422); IL15 SF; IL2G Receptor Binding SF										-2.7	3.09E-02
IL17RA	IL-17 Receptor; TIR 2 SF	-1.5	1.36E-02									
IL2RB	IL2 Receptor; IL15 Receptor; FN3 SF							-1.7	2.69E-02			
IL34	Cytokine; IL34 SF	-2.5	3.63E-04					-2.1	4.12E-03			
KIT	Ig SF; PKC-like SF										-2.3	2.04E-02
LIFR	LIF Receptor; FN3 SF	-2.1	4.05E-04					-1.7	7.18E-03		-2.6	3.03E-05
LTBP3	TGFB Binding Protein EGF CA SF	-1.6	4.59E-02									
LTBP4	TGFB Binding Protein EGF CA SF	-2.2	9.11E-05					-1.8	5.61E-03			
MDK	Cytokine; (+) Regulator of Inflammation (30217121); PTN MK N SF	-2.0	1.75E-02									
PDGFC	Angiogenesis (20566880); CUB SF; PDGF SF										-2.3	2.70E-03
PF4	CXC Chemokine SF										-3.2	1.71E-02
PGF	PDGF SF	-1.9	4.93E-02									
RARRES2	Adipocytokine (17767914)	-1.6	4.83E-02									
THNSL2	Cytokine (19877052); Trp-synth-beta II SF	-2.3	1.15E-03									
TNFSF11	Positive Regulator of Microfold Cell Differentiation (22778137); TH1-Associated (14607948); TNF SF										-4.0	1.83E-03
TNFSF13B	(+) Regulator of B Cell Activation (16797504); Th1-Associated (15843552); TNF SF										-2.5	2.69E-04
XCL1	Th1-associated (14573634); XCR1 Receptor Ligand	-5.2	1.96E-06					-2.7	5.72E-03		-3.3	3.63E-03
	**Extracellular Matrix and Remodeling**											
ADAM1A	Metalloprotease;; Pep M12B Propep SF; ZnMc SF; Reprolysin SF; Disintegrin SF; ADAM CR SF										-52.9	1.87E-02
ADAMTS19	Metalloprotease; TSP 1 SF										-2.6	1.33E-02
ADAMTS5	Metalloprotease; Pep M12B Propep SF; ZnMc SF; TSP 1 SF; ADAM Spacer1 SF										-2.2	1.84E-02
ADAMTS8	Metalloprotease; TSP 1 SF							-1.9	1.07E-02			
ADAMTSL5	Metalloprotease; TSP 1 SF	-2.1	3.76E-02									
ANPEP	Aminopeptidase; GluZincin SF	-5.2	3.11E-04		-2.5	4.13E-02						
ASTL	Metalloproteinase (15087446); ZnMc SF	-37.2	2.34E-02								-49.0	2.75E-02
BSG	Extracellular Matrix Remodeling (26084217); Treg-associated (23665204); Ig SF; KU SF							-1.5	2.27E-02			
COL14A1	Collagen SF										-1.7	3.72E-02
COL22A1	Collagen SF										-56.8	1.34E-02
COL25A1	Collagen SF										-5.6	7.28E-04
COL4A3	Collagen SF	-2.4	4.44E-02									
COL4A6	Collagen SF	-1.6	3.19E-02					-1.6	4.50E-02			
COL6A5	Collagen SF										-2.0	1.50E-02
COL6A6	Collagen SF	-7.8	1.07E-07					-5.7	3.03E-06		-5.7	3.51E-08
CST3	Cysteine Protease Inhibitor; Extracellular Matrix Formation/Degradation (19468292); Cystatin (CY) SF							-1.9	7.80E-03		-2.3	3.93E-04
CSTA	Protease Inhibitor; CY SF	-1.9	2.56E-02									
CTBS	Asn-linked Glycoprotein Catabolism; GH18 Chitinase-like SF	-3.6	8.38E-03					-2.9	4.10E-02			

CTSZ	Cathepsin; Peptidase C1 SF	-1.8	4.19E-03									
GALNS	Extracellular Matrix Degradation (18285341); Sulfatase SF; DUF4976 SF	-1.6	1.45E-02									
HAPLN4	Extracellular Matrix (12663660); Ig SF; Link Domain SF	-8.4	9.20E-06					-1.8	5.64E-03			
HAS3	Hyaluronan Synthesis; Glyco Tranf GTA-type SF	-8.4	9.20E-06					-5.2	5.01E-04		-4.0	4.07E-05
HGSNAT	Extracellular Matrix Degradation (21957468); DUF5009 SF;	-1.6	1.40E-02									
HPSE	Extracellular Matrix Degradation (12213822); Heparanase; Glyco hydro 79n SF	-1.6	1.75E-02					-1.5	5.25E-02			
HS3ST3A1	Glycosaminoglycan Biosynthesis (9988767); Extracellular Matrix (9988767); Sulfotransfer 3 SF							-2.5	4.16E-02			
ITIH5	Extracellular Matrix Stabilization (25809190); VIT SF; vWFA SF; ITI HC C SF	-1.9	1.46E-02									
MMP11	Metalloprotease; ZnMc SF	-2.0	7.40E-04									
MMP24	Metalloprotease; ZnMc SF	-1.8	2.06E-02									
MMP25	Metalloprotease; ZnMc SF	-1.8	1.15E-02									
PCSK6	Metalloprotease; Peptidases S8 S53 SF; P Proprotein SF; FU SF; PLAC SF	-2.3	1.07E-05					-1.6	5.72E-03			
PRSS12	Serine Protease; SRCR SF; Tryp SPc SF; KR SF							-2.7	9.44E-04		-2.5	5.62E-03
THSD4	Metalloprotease; TSP 1 SF; ADAM Spacer 1 SF; PLAC SF	-1.5	2.95E-02									
TMPRSS13	Serine Protease; Tryp SPc SF	-2.8	1.67E-02									
TMPRSS2	Serine Protease (20382709); SRCR SF	-2.4	1.23E-04					-1.8	1.60E-02			
TMPRSS4	Serine Protease (20382709); SRCR SF	-2.1	4.56E-05					-1.6	6.34E-03			
UGDH	Glycosaminoglycan Biosynthesis (9737970); F420 Oxidored SF; UDPG MGDP DH SF	-1.6	2.77E-03									
VSIR	Extracellular Matrix Formation (20666777); (-) Regulator of of T Cell Function (24691993); B7 SF; Ig SF	-1.5	1.46E-02									
	**Pattern (PR) and Damage-associated Recognition (DR) Receptors**											
C1QA	Complement Component; PRR (19799115); C1q SF	-2.3	4.91E-05					-1.7	1.29E-02			
C1QB	Complement Component; PRR (19799115); C1q SF	-2.6	4.13E-05		-1.6	5.22E-02						
C1QC	Complement Component; PRR (19799115); C1q SF	-2.3	5.39E-05					-1.7	1.61E-02			
C3AR1	Complement Component Receptor; GPCR SF										-2.3	5.27E-03
C4BPAL*	Complement Component Binding Protein; CCP SF										-2.4	3.78E-02
C5	Complement Component; Alpha-2-macroglobulin SF; ANATO SF; IsoPREN C2-like SF; MG1 SF; NTR-like SF										-1.9	1.14E-02
C7	Complement Membrane Attack Complex (2808363); CCP SF	-1.9	3.94E-02									
CD1D	Pattern Recognition Receptor (22734667); CD1 SF; MHC I SF; Immunoglobulin SF										-2.5	2.89E-03
CD207	PRR (27903635); DC-associated (21298011); CLECT SF	-4.2	4.37E-02									
CD209	PRR (15950451); M2 Macrophage-associated (15070901); CLECT SF	-2.8	1.42E-03					-2.1	3.13E-02			
CD302	PRR; DC-associated (28106145); CLECT SF										-2.1	6.84E-03
CD36	PRR (15690042, 19342682); SRCR SF; CD36 SF	-3.7	8.88E-09									
CD5L	PRR (16030018); SRCR SF	-10.9	8.19E-04		-4.7	6.80E-03					-4.2	2.41E-02
COLEC12	PRR (11162630); CLECT SF							-1.9	7.57E-03			
CR1	Complement Component; CCP SF										-3.0	1.86E-02
CR2	Complement Component; CCP SF										-5.2	3.00E-14
DHX58	PRR (16116171); RIG-I-Like Receptor Family	-3.3	7.59E-03					-2.7	3.59E-02			
LGALS9	PRR (12684513); GLECT SF	-2.2	2.92E-04					-1.8	1.10E-02			
SIGLEC15	SIGLEC SF	-3.4	5.06E-02									
TLR1	PRR (18056480), TLR SF	-2.2	8.67E-04									
TLR5	PRR, TLR SF	-2.0	4.03E-02									
TLR6	PRR (15661917), TLR SF	-2.0	4.77E-03								-2.1	1.41E-02
TLR7	PRR, TLR SF										-2.0	4.66E-02
TLR8	PRR, TLR SF	-2.3	1.46E-03					-2.2	2.49E-03			
TLR9	PRR, TLR SF											
TREM2	(-) Regulator of TLR4 Signaling (16951310); PRR (12847223); TREM-like Receptor SF; Ig SF				-2.5	4.89E-02						
ZBP1	Antiviral Immune Response (22344284); PRR (17618271); Z-alpha SF	-3.4	5.56E-04					-2.5	1.11E-02			
	**Oxidative Stress Response**											
CYBB	Superoxide Generation; Ferric Reductase SF	-1.6	3.34E-02									
DDIT4L	Oxidative Stress Response (12453409); RTP801 C SF										-3.0	2.84E-04
FDXR	Oxidative Stress Response (12370809); NAD binding 8 SF				-1.5	2.76E-02						
GSTA2	Glutathione S-transferase; Thioredoxin-like SF	-26.6	8.94E-06		-3.6	5.23E-02		-7.4	7.07E-03			
GSTM1	Glutathione S-transferase; Thioredoxin-like SF	-3.0	2.39E-05		-1.9	9.10E-03						
GSTM4	Glutathione S-transferase; Thioredoxin-like SF	-2.9	3.46E-06		-1.8	5.07E-03						
GSTP1	Glutathione S-transferase; Thioredoxin-like SF	-1.8	7.59E-03					-1.5	4.90E-02			
GSTT1	Glutathione S-transferase; Thioredoxin-like SF	-2.6	1.25E-03		-1.8	4.13E-02						
GSTT2	Glutathione S-transferase; Thioredoxin-like SF											
GSTZ1	Glutathione S-transferase; Thioredoxin-like SF				-1.6	1.03E-02						
IPCEF1	Oxidative Stress Response (11320098); PH-like SF										-2.3	2.46E-02
NOXO1	Superoxide Generation; PX Domain SF	-1.9	1.71E-03									
OXSR1	Oxidative Stress Response (14707132); PKC-like SF											
PRDX6	Oxidative Stress Response; Thioredoxin-like SF	-2.9	1.45E-07					-2.2	5.44E-05			
	**Surface Receptors and Ligands**											
ABCC4	Prostaglandin E2 Transporter (25433169); ABC Membrane SF											
ACVR1B	Activin Receptor SF; TGF beta GS SF	-1.5	1.73E-02									
ADGRB1	Angiogenesis; GPCR SF	-2.6	1.64E-02									
ADGRE4	GPCR SF	-2.4	9.29E-06		-1.6	2.26E-02					-2.6	1.56E-03
ADORA3	(-) Regulator of Inflammation (16849509); GPCR SF										-3.3	5.03E-03
BTNL2	(-) Regulator of Inflammation (17237401); (-) Regulator of T Cell Activation (17237401); Ig SF							-19.6	1.37E-02			
CADM1	Adhesion Molecule (18684968); CRTAM Ligand; Ig SF							-1.8	2.11E-02			
CD109	T Cell Activation Marker (11861284); Alpha-2-macroglobulin SF; IsoPREN C2-like SF; MG1 SF										-1.7	4.15E-02
CD2	Positive Regulator of TCR Signaling (11160298); CD2 SF; DUF2360 SF; Immunoglobulin SF										-1.8	9.24E-03
CD200R1L	Tolerogenic Dendritic Cell Marker (18424714); Ig SF										-6.9	7.81E-03
CD27	NK Cell Development Marker (18406373); T Cell Activation Marker; Treg Associated (17425604); TNFR SF	-2.2	4.87E-02									
CD28	(+) Regulator of T Cell Activation; Immunoglobulin SF										-3.2	1.08E-02
CD300C	(-) Regulator of Dendritic Cell/NK Cell Function (18535206, 20656921); CMRF-35 Family; Ig SF	-2.2	5.26E-03					-2.4	8.64E-04			
CD300H	(+) Regulator of Inflammation (26221034); CMRF-35 Family; Ig SF	-2.3	1.10E-05					-1.5	3.24E-02			
CD300LF	(-) Regulator of T Cell Activation (16794255); Ig SF; SIT SF	-1.8	5.14E-02									
CD302	Phagocytosis (19261355); CLECT SF	-1.9	9.28E-03					-1.6	5.19E-02			
CD33	SIGLEC SF	-1.6	4.32E-02									
CD36L1*	SRCR SF; CD36 SF	-2.3	2.24E-03									
CD3G	T Cell Associated; Ig SF; ITAM SF										-2.1	8.80E-03
CD4	T Cell Associated; Ig SF	-2.8	1.41E-04					-2.0	1.03E-02		-2.2	1.61E-02
CD40	T Cell Co-stimulatory; TNF Receptor SF										-17.7	4.10E-02
CD44	Link Domain SF	-1.6	2.09E-02									
CD48	Positive Regulator of Mast Cell Activation (12759438); SLAM Family Ligand; CD2 SF; Ig SF; SLAM SF										-1.9	1.09E-02
CD68L1*	LAMP SF	-1.7	1.16E-02									
CD7	Galectin 3 Receptor (14678989, 24792331); (+) Regulator of TCR Signaling (11485208); Ig SF	-2.2	4.61E-02									
CD72	B Cell Differentiation Marker; CLECT SF	-1.8	4.52E-02									
CD83	B Cell Activation Marker (8422464); DC Maturation Marker (12403928); Ig SF	-2.3	1.40E-02									
CD86	(+) Regulator of T Cell Activation; B7 SF (15960813); Immunoglobulin SF							-1.8	1.93E-02			
CEACAM8	Ig SF	-7.5	1.20E-02									
CLCA4	Chloride Ion Transport; CLCA N SF; vWFA SF; DUF1973 SF	-27.9	1.34E-07					-13.2	2.57E-05		-3.1	1.16E-02
CLCA4L*	CLCA N SF; vWFA SF; DUF1973 SF	-3.1	1.92E-02									
CLCN2	Voltage-gated Chloride Channel; Voltage-gated ClC SF							-1.8	3.54E-05			

CLEC17A	CLECT SF										-3.6	4.30E-03
CLEC2D	(+) Regulator of IFNG Production (15104121); KLRB1 Ligand (14990792); CLECT SF	-1.5	5.17E-02									
CLEC3B	Wound Healing (9152657); CLECT SF	-1.9	2.18E-03		-1.7	7.15E-03						
ERBB2 (CD340)	Recep L Domain SF; FU SF; PKC-like SF	-1.9	1.33E-03									
ERBB3	Recep L Domain SF; FU SF; PKC-like SF	-1.6	4.43E-02									
FCER1A	IgE; Receptor; Ig SF							-2.3	3.53E-02			
FCER1G	IgE Receptor; TCR Zeta Zeta SF; ITAM SF	-1.5	5.31E-02									
FCER2	IgE Receptor; CLECT SF										-12.0	1.34E-02
FCGR3A	Ig Receptor; IgSF	-2.2	8.60E-05					-1.7	1.21E-02			
FCGRT	Ig Receptor; IgSF	-1.5	2.30E-02									
FCRL1	B Cell Differentiation Marker (15479727); Immunoglobulin SF										-5.0	1.34E-02
FCRLA	B Cell Differentiation Marker (22078318); Immunoglobulin SF										-3.8	3.49E-02
IGJ	B Cell Marker; Ig J-Chain SF										-1.8	1.92E-02
ITGA4	T Cell Activation Marker; FG-GAP SF; Integrin Alpha2 SF										-1.7	4.03E-02
ITGAE	Treg-Associated (m, h) (15845457, 17620361); vWFA SF; FG-GAP SF; Integrin Alpha2 SF										-1.8	4.27E-02
JAML	(+) Regulator of Gamma-delta T Cell Activation (21220118); Neutrophil Chemotaxis (15800062); Ig SF	-1.7	2.72E-02					-1.7	3.23E-02			
KCNJ2	Sour Taste Receptor (26627720); IRK N SF; Ion Trans 2 SF										-2.5	2.94E-04
LAPTM5	(+) Regulator of Inflammation (22733818); Mtp SF	-1.6	3.96E-02									
LRP1B	Endocytosis (11384978); Pseudomonas Exotoxin Receptor (16095885); LDLa SF; EGF CA SF; LY SF	-2.7	1.12E-02									
LTB4R2	Leukotriene B4 Receptor; GPCRSF	-2.1	3.08E-02									
LY9	(+) Regulator of Toll-like Receptor Signaling Pathway (19395959); ML SF										-1.8	4.79E-02
MS4A2	IgE Receptor; CD20 SF							-1.9	2.34E-02			
P2RX3	Purinergic Receptor SF							-3.9	4.22E-03			
PADI2	(-) Regulator of Inflammation (18645041); PAD SF							-5.2	1.54E-06			
SCN2A	Voltage-gated Na$^+$ Channel; Na$^+$ Trans Assoc SF							-2.7	4.98E-02			
SEMA3A	Negative Regulator of T Cell Activation (16380453); Sema SF; Immunoglobulin SF							-2.2	1.38E-02			
SEMA4D	Positive Regulator of B Cell/DC Cell Function (18087252); Th1 Associated (18087252); Ig SF							-1.8	3.81E-02			
SIRPB2	Ig SF	-2.8	6.49E-03		-2.0	3.09E-02						
SLAMF6	Th1-Associated (14988414); SLAM SF; CD2 SF; Immunoglobulin SF										-2.1	4.40E-02
SSTR5	Somatostatin Receptor; GPCR SF	-5.7	4.20E-03					-4.8	1.30E-02			
TRPV3	Intracellular Ca2+ Channel (30127359); Ion Trans SF	-1.8	1.05E-02									
TRPV6	Chloride Ion Transport; Epithelial Cell Calcium Transporter (26109800); ANK SF	-6.0	4.34E-02								-7.7	1.24E-06
VIP	Hormone 2 SF	-3.5	5.68E-03									
	**Transcription Factors**											
AHRR	Transcriptional Repressor; HLH SF; PAS SF; Trp Dioxygenase SF	-2.8	3.02E-04					-2.6	6.47E-04		-2.2	2.98E-02
AR	Androgen Receptor; Nuclear Receptor (NR) DNA-binding Domain (DBD) SF										-2.7	4.53E-02
CDX2	Positive Regulator of Enterocyte/Goblet Cell/Paneth Cell Differentiation (21081128) ; Homeodomain SF	-1.6	1.15E-02									
DBP	B Zip1 SF							-2.1	1.42E-02			
DLX3	Homeodomain SF	-3.8	2.11E-02									
DLX4	Homeodomain SF	-4.1	2.69E-03									
EOMES	TC1-associated (14605368); TBOX SF	-4.8	1.12E-04		-2.2	3.65E-02						
EVX1	Homeodomain SF	-38.1	2.47E-02									
FOXF2	FH SF							-1.9	5.72E-03			
FOXH1	FH SF	-2.1	3.28E-02									
FOXJ1	Negative Regulator of NFKB Signaling (16585573); TH2-Associated (14963332); FH SF	-4.8	4.62E-03									
FOXO6	Gluconeogenesis (23324123); FH SF	-13.7	8.20E-06					-9.1	1.78E-04			
FOXS1	FH SF	-3.8	3.26E-05					-2.5	5.82E-03			
HOXA11	Homeodomain SF	-1.6	5.12E-02									
HOXA5	Homeodomain SF	-1.6	1.74E-02									
HOXC9	Homeodomain SF										-12.3	5.27E-03
HOXD10	Homeodomain SF	-2.3	8.74E-03					-2.6	1.40E-03		-2.6	1.85E-02
HOXD11	Homeodomain SF							-1.9	5.02E-02			
IRF4L	IRF3 SF	-3.4	5.23E-03					-2.7	3.39E-02			
IRF8	Type 1 IFN-Induced; Type 1 IFN Signaling (15864272); IRF/IRF4 SF (11997525)	-1.6	1.94E-02									
IRF9	Antiviral Immune Response (17559358); Type 1 and 2 IFN Signaling (15864272); IRF/IRF4 SF (11997525)	-1.8	5.24E-03					-1.5	4.68E-02			
ISX	Transcriptional Repressor (23393141); Homeodomain SF	-3.3	2.28E-03									
KLF11	zf-H2C2 2 SF	-4.2	3.47E-05		-2.0	4.37E-02						
KLF12	zf-C2H2 SF; zf-H2C2 2 SF										-2.9	1.06E-03
KLHL7	Ubiquitin Ligase (21828050); BTB SF; BACK-like SF; Kelch 1 SF	-2.1	2.73E-04					-1.9	2.88E-03			
MAF	M2 Macrophages (17082649); bZIP1 SF	-2.2	1.08E-04					-1.9	1.80E-03		-2.1	1.40E-02
MAFB	Macrophage Differentiation Marker (15598817); bZIP1 SF	-1.5	3.08E-02								-1.9	5.99E-03
MEIS2	Homeodomain SF							-1.8	5.29E-02			
NFATC2	TH2-Associated (8943202); UBQ SF										-1.9	4.03E-02
NR1D1	NR DBD-like SF; NR LBD SF							-1.8	3.13E-02			
NR1H4	Bile Acid Receptor (10334992); NR DBD-like SF; NR LBD SF	-17.1	2.31E-11		-2.3	2.51E-02		-7.6	1.96E-06		-3.7	1.44E-02
NR3C2	Mineralocorticoid Receptor; NR DBD-like SF; NR LBD SF	-1.6	2.11E-02									
OLIG2	Neurogenesis (21382543.); HLH SF										-143.0	1.09E-03
PAX5	B Cell Differentiation (15469848); PRD Class Homeobox Transcription Factor; Homeodomain SF										-2.8	2.30E-02
PCGF2	Th2-Associated (11520462); Polycomb-group (PcG) SF	-1.5	4.54E-02					-1.5	5.29E-02			
PKNOX2	Homeodomain SF	-2.0	2.25E-02					-1.8	5.16E-02			
POU6F1	Pou SF; Homeodomain SF							-1.8	3.37E-02			
PPARA	Th2 Associated (18645614)); NR DBD-like SF; NR LBD SF	-1.5	4.14E-02									
PPARD	M2 Macrophages (18522830); NR DBD-like SF; NR LBD SF	-1.6	4.85E-03									
SATB1	CD8 T Cell Development (15814699); SATB1 N SF; CUT SF; Homeodomain SF							-1.8	2.46E-02		-1.9	1.64E-02
SP110	Sp100 SF; SAND SF; PHD SF; Bromodomain SF	-1.5	5.40E-02									
TCF21	HLH SF	-1.8	4.67E-03					-1.6	1.33E-02			
TCF7L1	HMG-box SF; CTNNB1-binding SF	-1.9	2.24E-02									
TEF	B Zip1 SF	-1.9	3.52E-03					-1.8	3.94E-03			
THRA	Thyroid Hormone Receptor; Nuclear Receptor (NR) DNA-binding Domain (DBD) SF; NR LBD SF	-1.6	2.58E-02									
VDR	Vitamin D Receptor; NR DBD-like SF; NR LBD SF	-1.7	7.90E-03					-1.5	3.04E-02			
	**Mitochondrial Proteins**											
COQ8A	DNA Damage Response; APH ChoK-like SF	-2.2	9.79E-06		-1.5	1.73E-02						
HMGCS2	Ketogenesis (25347470); HMG CoA Synt C SF	-21.2	0.00E+00					-12.8	0.00E+00		-7.2	0.00E+00
MT-ATP6	ATP Synthesis; ATP Synt A SF	-1.7	1.36E-02									
MT-CO1	Respiratory Electron Transport Chain; Heme Cu Oxidase I SF	-1.7	7.42E-03									
MT-CO2	Respiratory Electron Transport Chain; Heme Cu Oxidase I SF	-1.5	5.08E-02									
MT-CO3	Respiratory Electron Transport Chain; Heme Cu Oxidase I SF	-1.9	4.64E-03									
MUL1	(+) Regulator of NFk-B Activation (18213395); RING Ubox SF	-1.5	1.04E-02									
SLC25A10	Malate Transporter (16027120); Mitochondrial Carrier Protein SF Solute Carrier SF	-1.8	2.22E-04									
SLC25A27	Mitochondrial Uncoupling Protein; Mitochondrial Proton Transport; Solute Carrier SF	-2.1	1.55E-05									
SLC25A34	Mitochondrial Phosphate Transporter; Mitochondrial Carrier Protein SF Solute Carrier SF	-7.8	1.41E-09									
SLC25A6	ATP/ADP Translocase; Mitochondrial Carrier Protein SF; Solute Carrier SF	-1.5	2.26E-02									
TRAP1	Mitochondrial Heat Shock Protein (21878357); HATPase c SF	-1.5	1.76E-02									
TST	RHOD SF	-2.2	3.80E-05					-1.5	5.16E-02			
UCP2	Mitochondrial Uncoupling Protein; Mitochondrial Proton Transport; Solute Carrier SF	-1.8	7.55E-03									
VARS2	Class I Aminoacyl tRNA Synthetase; Anticodon Ia-like SF; NT Trans SF	-1.5	1.93E-02									

	**Miscellaneous**											
ACE	Angiotensin I converting enzyme; Metalloprotease; GluZincin SF	-2.6	7.25E-05					-2.0	6.94E-03			
ACE2	Angiotensin I converting enzyme				-2.6	4.67E-02						
AGT	Angiotensinogen; SERPIN SF							-4.3	4.12E-03		-5.6	1.62E-05
AGTR1	Angiotensin II receptor; (+) Regulator of Inflammation (19225144); GPCR SF										-3.2	6.35E-04
AGTR2	Angiotensin II receptor; GPCR SF										-11.5	3.94E-03
ALPI	(-) Regulator of Intestinal Inflammation (20536777); alkPPc SF	-159.7	2.09E-10		-4.9	5.20E-03		-32.9	4.94E-06			
ALPIL1*	alkPPc SF	-10.3	1.67E-04		-3.1	3.58E-02						
ARRDC3	(-) Regulator of G protein-mediated Signaling (24292823); Arrestin N SF; Arrestin C SF				-1.5	4.39E-02						
CARD14	(+) Regulator of NFKB Activation (11278692); DD SF; PDZ SF; SH3 SF; NK SF	-2.2	2.07E-03									
CHP2	(+) Regulator of Calcineurin Signaling (18815128); (+) Regulator of NFAT Signaling (18815128); EFh SF	-2.9	5.14E-05								-2.9	1.12E-05
COBL	(+) Regulator of Intestinal Microvilli Formation (25031432); Cobl SF; Atrophin-1 SF							-1.5	1.01E-02			
COQ8A	DNA Damage Response; APH ChoK-like SF	-2.2	9.79E-06									
CTBS	Asn-linked Glycoprotein Catabolism; GH18 Chitinase Like SF (17594485)										-2.5	3.96E-02
DNAH14	DHC N2 SF; AAA SF; P-loop_NTPase SF; MT SF; Dynein Heavy SF; DHC N2 SF										-60.4	1.56E-02
DOK2	(-) Regulator of TLR4 Signalling (15699069); PH-like SF							-1.6	3.97E-02			
GUCA2A	(+) Regulator of Chloride Ion Transport (14988827); Guanylin SF	-6.3	2.26E-05					-5.1	1.44E-04			
HIC1	(-) Regulator of SIRT1 Signaling (23770291); Transcriptional Repressor (16269335); BTB SF; zf-C2H2 SF							-1.8	1.41E-02			
KCNE3	Voltage-gated Potassium Channel; ISK Channel SF				-1.7	1.16E-03						
LCN15	Lipocalin SF	-7.6	4.90E-02		-32.1	3.66E-07						
LCP2	(+) Regulator of T Cell Activation (17420479); SH2 SF; Sterile Alpha Motif (SAM) SF	-1.7	7.46E-03					-1.6	1.11E-02			
MAP3K3	Positive Regulator of TLR Signaling (14661019, 18206350); PB1 SF; PKc-like SF							-1.5	1.38E-02			
MERTK	(-) Regulator of Dendritic Cell Function (19301199); GAS6 Receptor; Ig SF; FN3 SF; PKC-like SF	-1.7	7.59E-03					-1.5	4.10E-02			
METTL7A	Methyltransferase; AdoMet MTases SF				-1.7	3.83E-03						
MOV10	Antiviral Response (26842467); AAA 11 SF; UvrD C 2 SF	-1.7	1.22E-03									
NT5E	Adenosine Metabolism; MPP SF; 5 Nucleotid C SF	-2.1	1.30E-02					-2.3	4.92E-03		-2.5	1.78E-04
NXPE2	Neurexophilin SF	-20.1	2.40E-06					-13.8	2.92E-05		-3.4	2.01E-03
PADI2	Negative Regulator of Inflammation (18645041); PAD N SF; PAD M SF; PAD SF	-8.6	1.42E-09								-2.5	1.60E-04
PIK3AP1	(+) Regulator of B Cell Development (12833156); DBB SF										-2.0	6.93E-03
PIK3IP1	(-) Regulator of T Cell Activation (22706993); KR SF	-1.9	1.62E-03					-1.5	4.80E-02			
PLA2G2A	Antibacterial Response (26644377); Colorectal Cancer; (18508504); Phospholipid Metabolism; PLA2-like SF										-1633.2	1.33E-05
PLA2G4B	Antibacterial Response (11741884); C2 SF; Patatin and cPLA2 SF	-1.6	3.68E-02									
PLCD1	(-) Regulator of TLR4 Signaling (26643908)	-2.4	1.77E-06		-1.6	1.46E-02		-1.5	3.39E-02			
PTPRD	(+) Regulator of STAT3 Signaling (17360477); Immunoglbulin SF; FN3 SF; PTPc SF	-2.6	1.41E-04					-2.5	2.63E-04			
RGS10	(-) Regulator of Inflammation (18716210); (-) Regulator of NFK-B Activation (21849548); RGS SF	-1.9	2.55E-02									
SCIN	(-) Regulator of Mucin Secretion (15342343); ADF Gelsolin SF	-1.8	3.67E-02									
SPIDR	DNA Damage Response (23754376); DUF4502 SF; DUF4503 SF	-1.9	2.85E-04									
SULT1E1	Phase II Conjugating Enzyme; Sulfotransfer 3 SF	-8.2	1.08E-09					-5.2	7.10E-07			
TICAM1	(+) Regulator of Toll-like Receptor Signaling (15219996); Ripoptosome Associated; TIR 2 SF; RHIM SF	-1.7	1.41E-03									
TNFAIP2	(+) Regulator of Inflammation (16456005); TNF Induced; Sec6 SF	-1.8	2.30E-04					-1.7	1.04E-03			
TYROBP	(-) Regulator of TLR Signaling (15895090); DAP10 SF	-1.6	3.74E-02									
UNC93B1	(+) Regulator of Endosomal TLR Signaling (18082565); Major Facilitator SF	-1.5	4.49E-02					-1.5	4.34E-02			
UVRAG	DNA Damage Response (27439570); Positive Regulator of Autophagy (26565689); C2 SF	-1.5	1.97E-02									
VAV3	Phagocytosis (16546099); TCR Signaling (15886116); CH SF; RhoGEF SF; PH-like SF; C1 SF; SH3 SF; SH2 SF	-1.5	5.02E-02									
VIL1	Bacterial Resistance (12181160); ADF Gelsolin SF; VHP SF	-1.5	2.16E-02									
ZFP42	Induced Pluripotent Stem Cell Marker (25830878); zf-H2C2 2 SF										-89.1	3.67E-11
	**NK Cell Associated**											
CD8A	NK Cell Associated; T Cell Associated; Ig SF	-2.2	4.54E-03		-1.7	5.31E-02						
GZMK	NK Cell Associated; Granzyme; Tryp SPc SF	-4.0	1.31E-02									
KLRC1	NK Cell Associated; CLECT SF	-2.5	2.35E-02									
KLRD1L*	CLECT SF				-2.1	2.36E-02						
KLRG1	NK Cell Associated; CLECT SF	3.7	4.54E-02					-2.0	4.23E-02			
KLRK1	(+) Regulator of NK Cell Activity (18979516); CLECT SF										-3.4	6.23E-09
NCAM1	Adhesion Molecule; NK Cell Associated; Ig SF	-1.9	3.06E-02									
NCR1	Pattern Recognition Receptor (11234016); IgSF	-7.2	1.19E-06		-2.9	2.48E-04						
NCR2	Pattern Recognition Receptor (11536166); Ig SF	-12.1	1.81E-05		-2.5	4.13E-02		-4.8	1.35E-02			
	**Inflammasome Associated**											
CASP1	Positive Regulator of IL1B/IL18 Bioactivity (10080100); DD SF	-2.1	9.13E-04									
PYCARD	Inflammasome Associated; DD SF	-1.9	8.72E-03					-1.8	1.54E-02			
TXNIP	NLRP3-associated (20075245); Arrestin C SF	-2.6	5.39E-05					-2.0	3.65E-03			
	**Neurotransmitter and Neuropeptide Related**											
ADRB1	Epinephrine Receptor; Neurotransmitter Receptor; GPCR SF							-2.0	7.57E-03			
CHRM2	Cholinergic Receptor; GPCR SF										-3.0	7.15E-03
GABARAPL1	UBQ SF	-1.8	6.54E-04					-1.5	2.90E-02			
GABBR1	GABA Receptor; GPCR SF	-1.8	2.17E-03									
GABRB2	GABA Receptor; GPCR SF	-5.4	4.12E-06					-4.0	1.45E-04			
GAL	Neuropeptide; Galanin SF; GMAP SF	-3.0	4.97E-02		-3.1	1.57E-02						
GFRA1	Receptor; GDNF SF	-2.0	4.61E-02									
GRIK1	Neurotransmitter Receptor; Non-NMDA Ionotropic Receptor; Periplasmic-binding Protein Type 1 SF	-8.6	4.27E-02									
GRIK4	Neurotransmitter Receptor; Non-NMDA Ionotropic Receptor; Periplasmic-binding Protein Type 1 SF	-2.7	1.76E-02					-2.7	9.60E-03			
GRINA	Neurotransmitter Receptor; NMDA Ionotropic Receptor; BI-1-like SF	-1.8	1.50E-03									
MAOA	Dopamine/Norepinephrine/Serotonin Catabolism (10389141); SDR SF	-2.7	1.11E-07					-1.9	7.84E-04			
NECAB2	(+) Regulator of Adenosine Receptor Signaling (17689978); EFh SF; ABM SF	-3.1	1.88E-02									
OPRL1	Nociceptin Receptor; GPCR SF	-4.9	3.95E-04									
P2RX3	Purinergic Receptor; P2X Receptor SF	-6.5	3.20E-05					-3.9	4.22E-03			
P2RY12	Purinergic Receptor; GPCR SF	-2.1	5.25E-02									
P2RY13	Purinergic Receptor; GPCR SF	-2.6	6.61E-04					-2.0	1.81E-02			
SCNN1B	Non voltage-gated Sodium Channel; Taste Receptor (23886383); ASC SF	-3.8	3.19E-02		-3.3	2.26E-02						
	**Type 1 or Type 2 IFN-Induced Genes**											
CXCL10	Type 2 IFN Induced; CXCR3 Ligand; CXC Chemokine SF	-3.4	3.22E-02					-4.4	7.18E-03			
CXCL11	Type 2 IFN Induced; CXCR3 Ligand; CXC Chemokine SF	-4.8	3.33E-02									
CXCL9	Type 2 IFN Induced; CXCR3 Ligand; CXC Chemokine SF	-7.5	6.02E-04					-5.7	3.65E-03		-6.1	7.08E-08
DAPK1	Type 2 IFN Induced (18250155); ANK SF; Ras-like GTPase SF; DD SF	-1.9	1.20E-02								-2.5	8.50E-03
ERAP2	Type 2 IFN Induced (12799365); Antigen Processing and Presentation (12799365); GluZincin SF	-1.6	4.60E-02									
GBP1	Type 2 IFN Induced (17266443); Ras-like GTPase SF	-1.9	5.21E-02					-2.0	4.04E-02			
GBP2	Type 2 IFN Induced (17266443); Ras-like GTPase SF	-2.4	1.02E-02					-2.4	9.65E-03			
GBP4	Type 2 IFN Induced (17266443); Ras-like GTPase SF	-2.4	4.83E-02									
GBP6	Type 2 IFN Induced (17266443); Ras-like GTPase SF	-2.6	5.25E-03					-2.1	3.20E-02			
FBP2	Type 2 IFN Induced (9282826); Gluconeogenesis (18780768); FIG Superfamily	-1.6	1.05E-02									
GPNMB	Type 2 IFN Induced (17475886); (-) Regulator of T Cell Activation (17284525); E set SF										-2.5	3.04E-05
HPSE	Type I IFN Induced Response (21478870); Antiviral Response (21478870); Heparanase; Glyco hydro 79n SF	-1.6	1.75E-02					-1.5	5.25E-02		-2.1	4.45E-03
IDO1	Type 2 IFN Induced (2109605); Tolerogenic Dendritic Cells (15459668) Marker; IDO SF										-3.8	6.52E-05

IFI27	Type 1 IFN Induced (20939681); Antiviral Response (27194766); Ifi-6-16 SF	-5.8	7.91E-04					-3.9	1.12E-02			
IFI44	Type 1 IFN Induced (7925411); Antiviral Immune Response (22896602); TLD SF	-2.9	1.80E-02					-2.7	2.55E-02			
IFI44L	Type 1 IFN Induced (21478870); Antigen Processing and Presentation (11021531); Ras-like GTPase SF	-2.3	3.75E-03					-2.0	1.59E-02			
IFITM1	Type 1 IFN Induced (14980080); Antiviral Immune Response (22344284); CD225 SF	-2.6	1.45E-02									
IFITM1L2*	CD225 SF	-2.3	6.10E-05					-1.7	8.91E-03			
IRF8	Type 1 IFN-Induced; Type 1 IFN Signaling (15864272); IRF SF; IRF4 Subfamily (11997525)	-1.6	1.94E-02									
MAP2K6	Type 1 IFN Induced (15644321); Antiviral Response (15229216); PKC-like SF	-1.8	3.77E-02					-1.8	4.90E-02			
MOV10	Type 1 IFN Induced (21478870); Antiviral Response (26842467); AAA 11 SF; UvrD C 2 SF	-1.7	1.22E-03					-1.5	1.20E-02			
MPEG1	Type 1 and 2 IFN Induced (23257510); Antibacterial Responses (23257510); MACPF SF	-2.2	4.93E-03					-2.1	5.72E-03		-2.1	7.99E-03
OASL	Type 1 IFN-Induced (10087211); Antiviral Response (22531715); NT Pol-beta-like SF; OAS1 C SF; UBQ SF	-14.4	5.68E-03					-12.1	1.20E-02			
PARP9	Type 2 IFN Induced (16809771); Antiviral Response (26479788); Macro SF; ADP Ribosyl SF							-1.9	1.04E-03			
RNF213	Type I IFN Induced (10547268); RING Ubox SF	-1.8	4.61E-02					-1.8	3.66E-02			
RSAD2	Type 1 IFN-induced (9391139); Antiviral Immune Response (20308629, 22377585); Viperin SF	-4.4	2.06E-02					-4.3	2.28E-02			
THAP12	Type 1 IFN Induced (21910972); Antiviral Response (21910972) ; THAP SF; Dimer Tnp hAT SF	-10.5	4.27E-02					-13.4	2.27E-02			
