# Supplementary material for: Molecular and metabolomic changes in the proximal colon of pigs infected with Trichuris suis
Source: Sci Rep. 2020 Jul 30;10:12853. doi: 10.1038/s41598-020-69462-5 (PMC7393168; doi:10.1038/s41598-020-69462-5)
Supplement: Supplementary file 9 — Supplementary Table S9. [file 41598_2020_69462_MOESM9_ESM.pdf]

Molecular and metabolomic changes in the proximal colon of pigs infected with *Trichuris suis*

Harry Dawson[1], Celine Chen[1], Robert Li[2], Lauren Nicki Bell[3], Terez Shea-Donohue[4], Helene Kringel[5], Ethiopia Beshah[1], Dolores E. Hill[2], Joseph F. Urban Jr[1,2].

[1]United States Department of Agriculture, Agricultural Research Service, Northeast Area, Beltsville Human Nutrition Research Center, Diet Genomics and Immunology Laboratory, [2] Beltsville Agricultural Research Center, Animal Parasitology Disease Laboratory, Beltsville, MD; [3]Metabolon, Inc., Morrisville, NC; [4]University of Maryland School of Medicine, Baltimore, MD, USA; [5]Department of Veterinary Disease Biology, Faculty of Health and Medical Sciences, University of Copenhagen, Copenhagen, Denmark

Supplemental Table S9

Supplemental Table S9: Clustered Metabolism Genes (DEGs) Up-regulated

Gene	Functional Categorization	52 DPI w worms vs Control Fold	EDGE FDR Adj p value		52 DPI w/o worms vs Control Fold	EDGE FDR Adj p value		(52 DPI Inf w/o worms vs w Worms Fold	EDGE FDR Adj p value		(21 DPI Inf vs Control) Fold	EDGE FDR Adj p value
	**Amino Acid Metabolism or Transport**											
ARG1	Arginine Metabolism (3540966); M2-associated (5879104); Arginase HDAC SF	122.6	4.56E-05					16.6	8.41E-04			
ARG2	Arginine Metabolism (16128822); Arginase HDAC SF	1.7	3.31E-02					1.6	2.16E-02			
BCAT1	BCAA Catabolism (6933702)); Glycolysis (28699638); PRK13357 SF	2.4	4.81E-04					1.9	5.18E-03			
CBS	Cysteine Metabolism (19010420); Serine Metabolism; Trp-synth-beta II SF; CBS Pair SF	3.4	1.61E-03					3.5	7.42E-05		5.5	7.18E-05
GNMT	Homocysteine Metabolism (21411609); AdoMet MTases SF										4.3	2.10E-02
IL4I1L	Putative L-amino acid oxidase; SDR SF	161.2	3.98E-05					26.0	1.39E-04		437.7	1.59E-14
ODC1	M2-associated (19726720); Ornithine Metabolism (2317811); Polyamine Metabolism; PLPDE III SF				-1.5	1.27E-02		1.6	4.12E-03			
PHGDH	Serine Biosynthesis (27110680); SerA SF	5.1	3.92E-05					3.3	5.01E-04		5.0	9.01E-04
PSAT1	Serine Biosynthesis (12633500); AAT I SF	7.2	3.12E-04					3.9	3.89E-03		20.6	4.69E-11
SDSL	Serine Metabolism (16580895); Threonine Metabolism (16580895); Trp Synth beta II SF	39.6	1.38E-02									
SHMT2	Glycine Biosynthesis (25619277); Folate Biosynthesis; Serine Biosynthesis	2.0	2.65E-05					1.5	1.06E-02			
SLC12A8	Amino Acid Transport (19472210); Polyamine Transport (19472210); Solute Carrier SF	3.5	7.40E-04					3.1	3.65E-04			
SLC16A10	Phenylalanine/Tryptophan/Tyrosine Transport (23045339); Solute Carrier SF	1.8	3.61E-02									
SLC1A2	Glutamate Transport (7521911); SDF SF; Solute Carrier SF	3.0	2.04E-03					2.1	2.44E-02			
SLC1A4	Alanine/Cysteine/Proline Transport ((8101838, 11824937; 14502423); Solute Carrier SF	2.5	1.36E-03									
SLC1A5	Glutamate/Glutamine Transport (21757002); Solute Carrier SF	1.8	8.53E-04					1.6	5.72E-03			
SLC38A2	Alanine/Asparagine/Cysteine/Glutamine Transport (12845534); Solute Carrier SF	1.8	2.90E-02									
SLC3A2	BCAA Transport (9751058); Tryptophan Transport; Tyrosine Transport; Solute Carrier SF	1.8	1.83E-03									
SLC6A9	Glycine Transport (8183239); Solute Carrier SF	1.8	1.29E-02									
SLC7A1	Arginine Transport (21302286); Lysine Transport (21302286); Solute Carrier SF	1.6	1.25E-02					1.5	3.05E-02			
SLC7A11	Cystine Transport (10206947); Glutamate Transport (10206947); Solute Carrier SF							3.7	3.60E-02		4.3	6.75E-07
SLC7A2	Arginine Transport (8954799), M2-associated (16670299); Solute Carrier SF	1.9	4.15E-02									
SLC7A3L11*	Putative Amino Acid Transporter; Solute Carrier SF										188.2	1.59E-03
SLC7A5	BCAA Transport (9751058); Solute Carrier SF	2.7	7.21E-06					1.9	1.40E-03		3.3	1.30E-03
SLC7A6	Arginine Transport (19562367); Solute Carrier SF	1.7	2.15E-03									
SLC9C1	Sodium-hydrogen Exchanger (14634667); Solute Carrier SF										4.1	7.81E-04
TAT	Bioconversion of L-Tyrosine into p-Hydroxyphenylpyruvate; AAT I SF	4.2	4.00E-02								6.9	3.49E-02
	**Carbohydrate Metabolism or Transport**											
AACS	Biosynthesis of Acetyl-Coenzyme A (20102333); AFD Classc I SF	2.0	4.99E-05					1.8	1.18E-04			
ACAT2	Biosynthesis of Acetyl-Coenzyme A; Thiolase N SF	3.5	1.27E-10					2.5	4.51E-08			
ACLY	Glycolysis; Lipid Biosynthesis (23932781); TCA Cycle; CCL SF	2.2	5.17E-06					1.9	1.76E-05			
ALDOC	Fructose Metabolism; Glycolysis; Aldolase Class I SF	1.6	5.08E-02					1.7	1.06E-02			
BCAT1	Glycolysis (28699638); PRK13357 SF	2.4	4.81E-04					1.9	5.18E-03			
DERL3	Negative Regulator of SLC2A1 Expression (24699711); DER1 SF	2.2	2.74E-04					1.7	7.70E-03			
GFPT1	Biosynthesis of Glucosamine 6-phosphate (1460020); Gn AT II SF; SIS SF	2.2	1.21E-05					1.7	3.70E-03			
GYS2	Glycogen Synthase SF							6.0	4.66E-02		11.9	5.62E-03
PCK2	Gluconeogenesis; Biosynthesis of Phosphoenolpyruvate (4291786); TCA Cycle; PEPCK HprK SF	2.0	1.77E-03					2.0	3.22E-04			
HK2	Glycolysis; Biosynthesis of Glucose-6-phosphate (19558793); NBD Sugar Kinase SF; Hexokinase 2 SF	8.6	2.40E-06					4.2	1.78E-04		3.3	1.33E-05
PFKFB2	Biosynthesis of Fructose-2,6-bisphosphate; Glycolysis; P-loop NTPase SF; HP SF	1.9	3.58E-02									
PFKFB3	Biosynthesis of Fructose-2,6-bisphosphate; Glycolysis; P-loop NTPase SF; HP SF	3.2	4.64E-06					2.2	5.86E-04		2.5	7.15E-03
PGK1	Biosynthesis of 3-phosphoglycerate; Glycolysis; Phosphoglycerate Kinase SF	1.8	1.24E-03					1.5	1.73E-02			
PGM2	Biosynthesis of Ribose 5-phosphate; Glycolysis (17804405); Phosphohexomutase SF	1.6	7.93E-03									
PGM3	Biosythesis of UDP-N-acetylglucosamine (11004509); Phosphohexomutase SF	2.1	7.37E-05					1.8	9.17E-04			
PKM	Biosynthesis of Pyruvate; Glycolysis (12843653); Pyruvate Kinase SF	1.9	3.11E-04					1.7	1.40E-03		2.2	2.62E-02
SDS	Biosynthesis of Pyruvate; Glycolysis; Serine/Threonine Catabolism; Trp Synth Beta II SF	5.6	1.53E-02								5.7	8.06E-05
SLC25A35	Isopropylmalate Transport (18682385); Solute Carrier SF							2.0	9.64E-03			
SLC2A3	Glucose Transport (20209635); Solute Carrier SF	3.4	1.42E-12		2.7	1.71E-10						
SLC37A3	Glucose-6-phosphate Transport (21949678); Solute Carrier SF	1.5	7.96E-03									
SLC50A1	Glucose Transport (23506865); Solute Carrier SF							1.8	9.18E-04			
SLC5A1	Glucose/Fructose/Mannose Transport (22212718); Solute Carrier SF	2.2	2.73E-04					1.7	6.38E-03			
TKTL1	Glycolysis (26273330)				6.4	1.49E-02						
	**Lipid Metabolism or Transport**											
ABCA12	Cholesterol Transport (23931754); ABC ATPase SF	4.1	1.91E-03								2.3	4.40E-02
ABCA5	Macrophage Cholesterol Metabolism (20382126); ABC ATPase SF	2.0	4.93E-03									
ACACA	Fatty Acid Biosynthesis (20457939); Acetyl-CoA Carboxylase SF; Biotinyl-lipoyl Domains SF	1.6	4.83E-03									
ACSL3	Fatty Acyl-CoA Ester Biosynthesis; AFD Class I SF; Acyl-CoA Synthetase SF	2.0	8.07E-05					1.7	2.03E-03			
ACSL4	Fatty Acyl-CoA Ester Biosynthesis; AFD Class I SF; Acyl-CoA Synthetase SF	1.5	3.29E-02									
AOAH	Lipid Metabolism (1883828); SGNH Hydrolase SF; SapB 2 SF	1.6	5.28E-02		1.7	2.02E-02						
APOBR	Lipoprotein Receptor; Neuromodulin N SF; Na Ca Ex SF				1.7	3.48E-02						
CYP51A1	Cholesterol Metabolism (12464255); Cytochrome P450	2.1	1.08E-04					1.6	5.76E-03			
CYP7A1	Cholesterol Metabolism (19965590); Cytochrome P450	12.7	4.64E-02									
DGAT2	Triglyceride Synthesis (11481335); NAT SF Superfamily							1.8	3.05E-02			
DHCR7	Cholesterol Biosynthesis (9465114); PEMT SF	1.9	3.19E-04					1.6	1.99E-03			
EBP	Cholesterol Biosynthesis (17498944); EBP SF	2.6	1.17E-05					2.3	4.62E-06			
ELOVL6	Fatty Acid Metabolism (19259639); ELO SF							2.4	1.46E-05			
FABP3	Long-chain Fatty Acid Transport (3421901); Lipocalin SF							2.3	1.30E-03		3.3	3.51E-08
FADS1	Fatty Acid Desaturase; Cyt-b5 SF; Membrane-FADS-like SF	2.4	6.98E-08					1.8	8.80E-05			
FADS2	Fatty Acid Desaturase; Cyt-b5 SF; Membrane-FADS-like SF	2.8	2.38E-06					2.3	1.63E-05			
FASN	Fatty Acid Biosynthesis (7835891); Acyl Transf 1 SF; NADB Rossmann SF	2.1	1.09E-04					1.7	3.60E-03			
FDFT1	Cholesterol Biosynthesis (19054015); Isoprenoid Biosynthesis C1 SF	1.9	3.52E-04					1.9	1.14E-05			
FDPS	Cholesterol Biosynthesis (8188698); Isoprenoid Biosyn C1 SF	2.2	8.28E-05					1.8	2.52E-03		2.0	3.53E-02
FFAR2	Acetate/Butyrate/Propionate Receptor (12711604, 27966553, 19574715); GPCR SF	3.0	5.18E-04		3.6	1.18E-05						
FFAR4	Fatty Acid Receptor (20813258); GPCR SF	2.3	1.81E-02					2.3	5.35E-03		2.3	2.68E-02
GPAT3	Triglyceride Biosynthesis (20181984); LPLAT SF	2.8	1.40E-04					2.5	7.03E-05			
HDLBP	Cholesterol Metabolism; KH-I SF										2.1	1.81E-02
HMGCR	Cholesterol Biosynthesis; MMPL SF; HMG-CoA Reductase SF	1.9	1.25E-03								1.9	1.78E-04
HMGCS1	Cholesterol Metabolism (7913309); HMG CoA Synt C SF	3.9	7.11E-09								2.8	3.97E-07
HSD17B7	Cholesterol Biosynthesis; NADB Rossmann SF; SDR SF	2.7	9.57E-06								1.9	1.22E-03
IDI1	Cholesterol Metabolism; Nudix Hydrolase SF	3.1	3.39E-06								2.2	2.17E-04
IFRD1	Lipid Metabolism (16085642); IFRD SF	1.8	2.25E-03									
INSIG1	Cholesterol Metabolism (12242342); INSIG SF	3.1	6.97E-07					2.9	1.64E-08			
LDLR	Cholesterol Metabolism; LDLa SF; EGF CA SF; LDL Receptor B SF	2.8	1.77E-06					2.1	2.26E-04			
LIPG	Lipid Metabolism; Lipase SF; PLAT SF	4.0	1.77E-03					2.3	4.46E-02			
LIPH	Lipid Metabolism; Lipase SF	2.2	2.78E-04					1.7	8.88E-03		2.4	1.88E-04
LPCAT2	Biosynthesis of Phosphatidylcholine (21498505); LPLAT SF; EFh SF				1.9	2.33E-03						
LRP2	Lipoprotein Metabolism (7642623); LDLa SF; EGF CA SF	4.3	1.38E-02					3.6	1.26E-02			

LSS	Cholesterol Biosynthesis; SQHop Cyclase C SF	2.2	1.54E-05					1.8	3.41E-04		1.9	3.03E-02
ME1	Fatty Acid Biosynthesis; Glycolysis; Malic SF; NAD Bind Amino Acid DH SF							2.6	5.20E-03		3.1	9.19E-03
MSMO1	Cholesterol Biosynthesis; FA Hydroxylase SF	2.5	5.94E-06								2.2	4.56E-06
MVD	Cholesterol Metabolism (18823933); MVD1 SF	1.9	2.48E-04					1.8	5.01E-04			
NPC1L1	Cholesterol Transport (15173162); SecD SecF SF; Patched SF							3.9	4.56E-02			
NSDHL	Cholesterol Biosynthesis (14506130); NADB Rossmann SF; SDR SF	1.7	4.11E-03					1.6	4.96E-03			
OSBPL8	Cholesterol Metabolism (17991739); H-like SF; Oxysterol BP SF	1.8	5.05E-02									
PCSK9	Cholesterol Metabolism (18753623); Inhibitor I9 SF; Peptidases S8 S53 SF	11.5	5.33E-09					9.0	1.11E-11			
PLA2G3	Lipid Metabolism (10713052); PLA2-like SF	3.6	7.51E-05					2.7	4.13E-04		2.9	4.90E-03
SC5D	Cholesterol Biosynthesis; FA Hydroxylase SF	1.8	3.28E-03					1.7	1.42E-03			
SCD	Lipid Metabolism; Vitamin A Metabolism (21573029); Membrane-FADS-like SF	3.9	9.81E-07					2.5	1.62E-04			
SOAT1	Cholesterol Ester Biosynthesis (16647063); MBOAT SF	1.7	1.57E-02									
SQLE	Cholesterol Biosynthesis (10666321); NADB Rossmann SF; SE SF	2.9	7.52E-07					2.3	8.68E-06			
STARD10	Phospholipid Transport (15911624); SRPBCC SF	1.5	3.63E-02					1.6	4.04E-03		1.9	1.91E-02
STARD4	Cholesterol Transport (23033213); SRPBCC SF	2.8	1.65E-06					1.9	8.33E-04			
STARD5	Cholesterol Transport (15897605); SRPBCC SF	1.5	4.88E-02					1.7	4.28E-03			
VLDLR	Lipid Transport; LDLa SF; EGF CA SF; Ldl Recep B SF	1.6	1.87E-03					1.5	7.00E-03			
	**Mineral Metabolism or Transport**											
ATOX1	Cu Metabolism (18685091); HMA SF	2.0	3.87E-03					1.8	8.67E-03			
FLVCR2	Ca Transport; MFS SF	1.6	2.26E-02					1.8	2.73E-03			
HEPHL1	Fe Metabolism (20685892); Cupredoxin SF	4.1	5.31E-02									
HP	Fe Metabolism; CCP SF							2.3	1.38E-02			
LTF	Fe Metabolism; Transferrin SF	52.9	5.95E-03									
MAGT1	Magnesium Transporter (21796205); Thioredoxin-like SF; OST3 OST6 SF	1.5	2.43E-02									
SELENOF	Selenoprotein; Sep15 SelM SF	1.6	1.38E-02					1.5	1.19E-02			
SELENOI	Selenoprotein (12775843); CDP-OH P Transf SF	1.5	5.04E-03									
SELENOK	Selenoprotein; DUF2763 SF	1.7	1.33E-03									
SELENOM	Selenoprotein; Sep15 SelM SF	2.1	1.21E-05					1.6	1.73E-02			
SELENOS	(-) Regulator of Inflammation (16227999); Selenoprotein S SF	1.9	2.48E-03					1.5	3.07E-02			
SLC11A1	Antibacterial Response (25350459); Fe/Mn Transport ; MntH SF; Solute Carrier SF							2.9	2.78E-02			
SLC30A5	Zn Transport (19759014); Solute Carrier SF	1.8	1.03E-03					1.5	3.16E-02			
SLC30A7	Zn Transport (12446736); Solute Carrier SF	1.7	2.93E-03					1.5	3.01E-02			
SLC39A10	Zn Transport; Solute Carrier SF	1.6	5.43E-02									
SLC39A11	Zn Transporter; Zip SF; Solute Carrier SF	1.5	5.49E-02					1.5	3.01E-02			
SLC39A7	Zn Transport (15705588)	1.7	1.29E-03					1.5	1.20E-02		1.9	9.92E-03
SLC41A2	Magnesium Transport; Solute Carrier SF	2.2	5.17E-02									
SLC5A8	Iodide Trasnporter; Solute Carrier SF	11.9	4.56E-05		4.8	3.38E-02					5.0	1.13E-04
STC1	(-) Regulator of Intestinal Calcium Transport (9458778); Stanniocalcin SF	2.2	1.42E-02					2.2	3.42E-03			
STEAP2	Copper Metabolism (26934375); Iron Metabolism (16609065); Cytochrome b N SF	1.8	2.56E-02									
STEAP4	Copper Metabolism (16609065); Iron Metabolism (16609065); Cytochrome b N SF	4.0	5.38E-03								3.4	5.33E-03
TFRC	Iron Transport; Zinc Peptidase-like SF; TFR Dimer SF	2.8	3.47E-05					2.5	2.06E-05			
	**Vitamin Metabolism or Transport**											
AKR1C4	Vitamin A Metabolism (21851338); Aldo Ket Red SF										4.3	2.30E-05
DHRS9	Retinol Dehydrogenase; Vitamin A Metabolism (11304534); NADB Rossmann SF; SDR SF	3.1	4.24E-04					2.1	1.73E-02			
HCAR2	Negative Regulator of Inflammation (23882124); Nicotinic Acid Receptor; GPCR SF	13.3	7.26E-04								9.3	6.76E-05
MTHFD1	Folic Acid Metabolism; THF DHG CYH SF	1.5	1.94E-02									
MTHFD2	Folic Acid Metabolism; THF DHG CYH SF; NADB Rossman SF	1.8	2.97E-03									
PANK1	Coenzyme A (CoA) Biosynthesis; Pantothenate Metabolism; Fumble SF	1.9	9.77E-03					1.7	2.78E-02			
PANK3	Coenzyme A (CoA) Biosynthesis; Pantothenate Metabolism; NBD Sugar Kinase HSP70 Actin SF	2.2	4.27E-02									
SLC23A1	Ascorbic Acid Transport (23177992); Sulfate Transport SF; Solute Carrier SF	1.9	3.44E-03									
SLC2A1	Ascorbic Acid Transport (23177992); Solute Carrier SF	1.7	7.88E-03									
SLC2A3	Ascorbic Acid Transport (23177992); Solute Carrier SF	3.4	1.42E-12		2.7	1.71E-10						
STRA6	Vitamin A Metabolism (17255476) RBP Receptor SF							3.8	6.03E-03			
TCN1	Vitamin B12 Transport; Cobalamin bind SF	54.1	1.15E-04		19.2	1.29E-02					8.0	1.44E-10
	**Miscellaneous Metabolism or Transport**											
ABCC13	Orphan Transport; ABC Membrane SF; P-loop NTPase SF											
AKR1C1	Steroid Hormone Metabolism (25583233); Aldo Ket Red SF	7.0	5.11E-02								13.8	3.93E-07
CA4	Bicarbonate Transport (15563508); Alpha CA SF	4.3	9.18E-05								5.5	9.51E-10
CLIC2	Chloride Transport; Chloride Intracellular Channel Protein 1 SF	1.6	2.84E-02									
DIO2	Selenoprotein; Thyroid Hormone Metabolism; Thioredoxin-like SF	6.5	3.02E-04		5.8	1.16E-03					3.5	1.15E-03
IDE	(-) Regulator of Insulin Receptor Signaling (12773120); Peptidase M16 SF	1.6	7.53E-03									
KCNE3	Voltage-gated Potassium Channel; ISK Channel SF							1.6	6.59E-03		2.6	3.51E-05
KCNJ11	Potassium Channel; Ion Trans 2 SF										2.7	3.31E-02
RHCG	Solute Carrier SF	8.0	4.93E-02					19.8	5.18E-04			
SCNN1D	Epithelial Sodium Channel (ENaC) Family (26772908); ASC SF	2.4	1.45E-02									
SLC12A4	Potassium-chloride Cotransport; Solute Carrier SF	1.5	4.15E-03		1.5	1.59E-03						
SLC15A2	Oligopeptide Transport; Solute Carrier SF	2.4	2.11E-03									
SLC16A3	Lactate Transport; Monocarboxylate Transport; Solute Carrier SF	2.6	1.23E-03		2.4	2.50E-03						
SLC17A9	Nucleotide Transport; Solute Carrier SF	2.2	1.07E-05					1.6	5.72E-03			
SLC20A1	Phosphate Transport; Solute Carrier SF	1.7	1.63E-02									
SLC24A5	Sodium/Potassium/Calcium Exchange; Solute Carrier SF; Na Ca Ex SF							1.9	4.98E-02			
SLC25A14	Mitochondrial Carrier Protein SF; Solute Carrier SF										2.0	4.03E-02
SLC26A2	Sulfate Transport (7923357); Solute Carrier SF				1.8	5.24E-02						
SLC35A3	Nucleotide Sugar Transport; Solute Carrier SF	2.1	3.51E-03					1.7	1.61E-02			
SLC35B1	UDP-galactose Transport (9010752); Solute Carrier SF	1.5	2.46E-02									
SLC35C1	GDP-fucose Transport; EamA SF; Solute Carrier SF	1.5	4.14E-02					1.6	7.89E-03		1.8	4.89E-02
SLC4A11	Bicarbonate Transporter (15525507); Borate Transporter (15525507)	4.8	5.93E-03		6.9	1.04E-03						
SLCO1A2	Bile Acid Transport (9398014); Solute Carrier SF										8.6	6.00E-04
SLCO4A1	Steroid Sulfate Transport; Solute Carrier SF	3.1	7.29E-05		2.1	1.73E-02						
SPHK1	Biosynthesis of Sphingosine 1-phosphate; DAGK Cat SF	2.8	3.05E-03					2.8	3.05E-03			
SULT2A1	Sulfation of Bile Acids/Steroids; Sulfotransfer 3 SF	13.4	4.28E-02					7.5	5.36E-02			
	**Transcription Factors**											
SREBF1	Lipid Metabolism; Transcription Factor (12177166); HLH SF	2.0	1.67E-04									
