# Supplementary material for: Molecular and metabolomic changes in the proximal colon of pigs infected with Trichuris suis
Source: Sci Rep. 2020 Jul 30;10:12853. doi: 10.1038/s41598-020-69462-5 (PMC7393168; doi:10.1038/s41598-020-69462-5)
Supplement: Supplementary file 10 — Supplementary Table S10. [file 41598_2020_69462_MOESM10_ESM.pdf]

Molecular and metabolomic changes in the proximal colon of pigs infected with *Trichuris suis*

Harry Dawson[1], Celine Chen[1], Robert Li[2], Lauren Nicki Bell[3], Terez Shea-Donohue[4], Helene Kringel[5], Ethiopia Beshah[1], Dolores E. Hill[2], Joseph F. Urban Jr[1,2].

[1]United States Department of Agriculture, Agricultural Research Service, Northeast Area, Beltsville Human Nutrition Research Center, Diet Genomics and Immunology Laboratory, [2] Beltsville Agricultural Research Center, Animal Parasitology Disease Laboratory, Beltsville, MD; [3]Metabolon, Inc., Morrisville, NC; [4]University of Maryland School of Medicine, Baltimore, MD, USA; [5]Department of Veterinary Disease Biology, Faculty of Health and Medical Sciences, University of Copenhagen, Copenhagen, Denmark

Supplemental Table S10

Supplemental Table S10: Clustered Metabolism Genes (DEGs) - Down-regulated

Gene	Functional Categorization	52 DPI w worms vs Control Fold	EDGE FDR Adj p value	52 DPI w/o worms vs Control Fold	EDGE FDR Adj p value	(52 DPI Inf w/o worms vs w Worms Fold	EDGE FDR Adj p value	(21 DPI Inf vs Control) Fold	EDGE FDR Adj p value
	**Amino Acid Metabolism or Transport**								
ACADSB	BCAA Metabolism (21430231); Leucine Metabolism (21430231); ACAD SF	-1.6	1.66E-03						
AHCYL2	Homocysteine Metabolism; FDH GDH-like SF	-2.4	5.80E-06			-2.1	5.97E-05	-1.8	2.15E-02
AOC1	Arginine Metabolism; Tryptophan Metabolism; Cu Amine Oxid SF					-2.4	4.46E-05		
ASPA	Asparate Metabolism (9407392) ; Peptidase M14-like SF	-2.9	4.20E-03					-2.8	1.01E-02
IVD	Branched-chain Amino Acid Metabolism (25287287); ACAD SF	-1.6	2.32E-02						
PIPOX	Lysine Catabolism (10642506); NAD Binding 8 SF	-4.9	6.45E-03						
SLC25A34	Isopropylmalate Transporter (18682385); Solute Carrier SF	-7.8	1.41E-09			-4.2	1.12E-05		
SLC25A38	Glycine Transporter (26821380); Solute Carrier SF	-1.9	4.57E-04						
SLC38A4	Arginine/Glycine/Histidine/Lysine Transporter (11342143); Solute Carrier SF	-7.7	3.14E-09	-2.6	5.46E-04	-3.0	3.10E-03		
SLC43A2	BCAA/Methionine/Phenylalanine Transporter (15659399); Solute Carrier SF	-1.8	2.00E-03	-1.6	2.36E-02				
SLC6A6	Taurine Transporter (8010975); SLC5-6-like SBD SF; Solute Carrier SF	-1.6	5.12E-02						
SLC7A3L2*	Orphan Transporter; Spore Permease SF; Solute Carrier SF	-1.8	4.24E-04						
SLC7A3L4*	Orphan Transporter; Spore Permease SF; Solute Carrier SF	-2.6	4.44E-03			-2.1	4.13E-02		
SLC7A3L6*	Orphan Transporter; Spore Permease SF; Solute Carrier SF	-2.8	1.70E-02						
SLC7A3L7*	Orphan Transporter; Spore Permease SF; Solute Carrier SF	-2.4	3.11E-02			-2.3	4.90E-02	-3.6	4.06E-04
SLC7A3L8*	Orphan Transporter; Spore Permease SF; Solute Carrier SF	-1.7	1.79E-02						
SLC7A8	Alanine/Phenylalanine Transporter; Solute Carrier SF	-3.1	2.76E-05			-2.1	5.64E-03		
THNSL2	O-phospho-homoserine Catabolism (17034760); Trp-synth-beta II SF			-2.2	2.04E-04				
	**Carbohydrate Metabolism or Transport**								
AKR1E2	Fructose Metabolism (9504428); Aldo Ket Red SF	-1.9	3.20E-03			-1.8	4.12E-03		
ALDOB	Fructose Metabolism; Glycolysis; Aldolase Class I SF	-27.7	2.35E-07					-10.6	8.40E-19
FBP2	Fructose Metabolism (16213487); Gluconeogenesis (18780768); FIG SF	-1.6	1.05E-02						
PCK1	Gluconeogenesis (20124556); PEPCK HprK SF	-16.9	1.62E-07	-3.1	1.86E-02	-5.4	1.48E-03		
PDK2	Gluconeogenesis (12682057); BCDHK Adom3 SF; HATPase C SF	-1.9	1.11E-03	-1.6	1.50E-02				
PDK4	Gluconeogenesis; BCDHK Adom3 SF; HATPase C SF	-7.7	2.11E-04	-3.0	3.61E-02			-4.6	5.99E-03
PFKFB4	Fructose Metabolism (10095107); Glycolysis; P-loop NTPase SF	-2.0	4.11E-05						
PGM5	Biosynthesis of Glucose-6-phosphate (8586438); Phosphohexomutase SF							-2.1	8.60E-04
SI	Isomaltose/Sucrose Metabolism (7002920); Trefoil SF; GH31 SF	-7.9	8.57E-03			-6.1	2.79E-02		
SLC2A4	Ascorbic Acid/Class I Glucose Transporter Family Member (23177992); Solute Carrier SF	-1.8	1.06E-03						
SLC45A3	Glucose Transporter (22521588); Solute Carrier SF					-1.6	2.17E-02		
	**Lipid Metabolism or Transport**								
A1CF	Lipid Metabolism (29083408); RNA Recognition Motif (RRM) SF; DSRM SF			-1.6	1.73E-02				
ABCA1	Cholesterol Metabolism; Lipid Metabolism; ABC ATPase SF					-1.7	5.31E-02	-1.9	5.62E-03
ABCA6	Macrophage Cholesterol Metabolism (11478798); ABC ATPase SF	-6.5	3.05E-06			-4.4	1.88E-04	-6.4	3.40E-08
ABCA8	Bile Acid Transporter (12379217); ABC ATPase SF	-4.1	3.01E-03			-2.7	4.77E-02	-6.8	4.42E-10
ABCA9	Macrophage Cholesterol Transport (12150964); ABC ATPase SF	-2.2	2.05E-03			-2.7	5.24E-02	-5.5	3.06E-08
ABCB4	Bile Acid Phospholipid Transport; ABC ATPase SF	-2.7	2.10E-02						
ABCC6	Cholesterol Transport (25064003); ABC ATPase SF	-2.1	2.65E-05						
ACAD10	Fatty Acid Oxidation (21237683); ACAD SF	-1.6	1.02E-02						
ACADS	Fatty Acid Beta Oxidation; Mitochondrial Lipid Metabolism (18977676); ACAD SF	-1.7	2.96E-03						
ACSBG1	Very Long-chain Fatty Acid Oxidation (10954726); Acyl-CoA Synthetase SF (17762044)	-2.8	4.00E-02						
ACSL6	Lipid Metabolism; Long-chain ACS Family; AFD Class I SF; Acyl-CoA Synthetase SF (17762044)							-3.7	9.52E-03
APOE	Cholesterol Metabolism; Apolipoprotein A1/A4/E SF	-1.7	3.18E-02						
APOL2	ApoL SF	-2.4	1.75E-04			-1.7	3.42E-02		
BDH1	Fatty Acid Catabolism (8679568); NADB Rossmann SF			-1.7	5.79E-03	-1.7	7.89E-03		
CES1B*	Cholesterol Metabolism; Lipid Metabolism; Carboxylesterase Family	-2.1	5.66E-04	-1.7	7.07E-03				
CYP4V2	Fatty Acid Metabolism (19661213); Cytochrome P450 SF							-2.0	1.52E-02
CYP7B1	Bile Acid Synthesis (24927729); Cytochrome p450 SF					-2.0	1.59E-02	-1.7	4.83E-02
FAAH	Lipid Metabolism; Amidase SF	-2.7	6.61E-04						
FABP1	Lipid Metabolism (17449472); Lipocalin SF	-3.7	5.00E-05			-2.4	1.03E-02		
FABP3L2*	Lipocalin SF							-44.6	1.84E-04
FABP4	Lipid Metabolism (11891416); Lipocalin SF	-2.7	1.86E-02						
GPIHBP1	Lipoprotein Metabolism (17997385); LU SF	-5.3	6.94E-04	-2.5	3.06E-02				
HADHA	Long Chain Fatty Acid Beta Oxidation (8135828); Crotonase-like SF; AdoMet MTases SF	-1.7	1.84E-04	-1.5	1.16E-03				
LIPA	Lipid Metabolism (16131527); Abhydro Lipase SF	-1.5	3.68E-02						
LIPE	Lipid Metabolism; HSL N SF	-1.7	3.48E-02						
LIPM	Abhydro Lpase SF							-19.0	2.62E-02
MOGAT2	Triglyceride Biosynthesis (25164810); LPLAT SF	-9.2	1.14E-03			-4.9	2.97E-02	-5.4	8.78E-03
OSBPL10	Cholesterol Metabolism (19554302); H-like SF; Oxysterol BP SF	-1.9	6.89E-03						
PLIN1	Inhibition of Lipolysis (17353663); Perilipin SF	-4.0	3.06E-02					-4.3	4.50E-05
PLTP	Lipoprotein Metabolism; BPI SF	-1.7	1.75E-02						
PNPLA2	Lipid Metabolism (18445677); Patatin and cPLA2 SF	-2.4	1.05E-06	-1.6	1.01E-02	-1.5	3.42E-02		
PNPLA6	Phosphatidylcholine Catabolism (15044461); CAP ED SF; Patatin and cPLA2 SF	-1.6	2.08E-02			-1.6	6.03E-03		
PNPLA7	Lysophospholipase (18086666); CAP ED SF; Patatin and cPLA2 SF	-1.6	4.92E-03						
PON1	Lipid Metabolism (19550323); Str Synth SF			-4.5	4.13E-02				
PPAP2B	Sphingosine-1-phosphate (S1P) Metabolism (12471011); PAP2-like SF							-1.8	1.44E-02
SCARB2	Cholesterol Metabolism (14570588); SRCR SF	-1.6	1.71E-03			-1.6	5.21E-04		
SCARF1	Lipid Metabolism (15145948); EGF Lam SF	-1.5	4.78E-02						
SLC10A2	Bile Acid Transporter; Lipid Metabolism; Solute Carrier SF	-1285.8	8.30E-08	-4.3	4.13E-02	-297.1	1.18E-04		
SLC16A1	Short-chain Fatty Acid Transporter; Solute Carrier SF	-5.7	0.00E+00			-4.4	0.00E+00	-2.2	1.59E-03
SLC25A20	Carnitine Transporter; Fatty Acid Metabolism; Solute Carrier SF	-2.1	1.13E-04						
SLC25A29	Mitochondrial Carnitine Transporter (12882971, 19287344); Solute Carrier SF	-1.9	1.01E-02						
SLC25A42	Coenzyme A Transporter (19429682); Solute Carrier SF					-2.1	7.36E-03		
SLC27A1	Fatty Acid Transporter; Lipid Metabolism; Solute Carrier SF	-1.7	3.17E-02						
TM6SF2	Lipid Metabolism (24927523); DUF2781 SF	-6.3	2.26E-14			-4.0	1.95E-09		
	**Mineral Metabolism or Transport**								
ABCG2	Heme Transport (16487711); ABC ATPase SF	-7.6	6.40E-07			-3.4	2.49E-03	-3.5	9.73E-07
CLCA4	Chloride Ion Transport; CLCA N SF; vWFA SF	-27.9	1.34E-07						
FTL	Iron Storage; Ferritin-like SF	-1.6	4.64E-02						
PHEX	Phosphate Metabolism (22339660); GluZincin SF							-6.8	1.81E-02
SCARA5	Ferritin Receptor (19154717); Iron Metabolism (19154717); SRCR SF	-2.4	2.08E-02						
SELENBP1	Selenium Containing Protein; BP56 SF	-4.4	1.11E-11			-3.0	1.73E-07		
SELENOP	Selenoprotein (12423375); Selenoprotein P SF	-3.3	2.97E-08			-2.3	5.83E-05		
SLC25A3	Mitochondrial Phosphate Transporter (23266187); Solute Carrier SF							-1.9	2.19E-02
SLC26A3	Chloride Ion Transport (16715296); Solute Carrier SF	-12.7	8.30E-08			-12.0	9.52E-08	-2.5	8.05E-03
SLC30A10	Mg Transporter (22341972); Zn Transporter (22706290); Solute Carrier SF	-10.2	3.00E-03			-6.3	2.38E-02	-9.4	2.71E-13
SLC30A3	Zn Transporter; Solute Carrier SF	-3.1	8.38E-03						
SLC31A2	Copper Transporter (9207117); Solute Carrier SF	-1.8	2.06E-02						
SLC39A5	Zn Transporter (15322118); Solute Carrier SF	-2.3	3.63E-02						
SLC8A1	Intestinal Calcium Transport (26109800); Solute Carrier SF					-1.6	5.29E-02	-2.1	2.28E-03
	**Vitamin Metabolism or Transport**								
ALDH1A3	Retinoic Acid Aynthesis Vitamin A Metabolism; ALDH-SF SF					-2.0	1.10E-02		
CYP26B1	Retinoic Acid Catabolism; Vitamin A Metabolism; Cytochrome P450 SF	-5.0	2.61E-08			-4.6	4.16E-08	-4.8	9.76E-03
CYP27A1	Vitamin D Metabolism (15353333); Cytochrome P450 SF	-1.8	3.99E-02						
MTHFR	Folic Acid Metabolism; MTHFR SF	-1.8	7.98E-03						
NADK	NADPH Metabolism (17855339); nadF SF					-1.6	4.96E-03		
RBP5	Vitamin A Metabolism (11274389); Lipocalin SF	-2.4	5.79E-03						
RDH10	Retinol Dehydrogenase; Vitamin A Metabolism; NADB Rossmann SF; SDR SF							-2.0	2.25E-02
RDH16	Retinol Dehydrogenase; Vitamin A Metabolism; NADB Rossmann SF; SDR SF	-6.1	3.20E-03	-2.8	4.89E-02				
RETSAT	Vitamin A Metabolism (15358783); NADB Rossmann SF; COG1233 SF	-2.2	4.10E-06			-1.6	7.89E-03		
SDR9C7	Putative Retinol Dehydrogenase (19703561); NADB Rossmann SF; SDR SF	-9.3	8.99E-03	-4.2	2.26E-02				
SLC25A32	Folate Transporter (10978331); Solute Carrier SF								
TCN2	Vitamin B12 Transporter; Cobalamin Bind SF; DUF4430 SF	-1.5	3.88E-02						
VDR	Vitamin D3 Signaling; Transcription Factor; NR-DBD SF	-1.7	7.90E-03						
	**Miscellaneous Metabolism or Transport**								
ABCB1	ATP-dependent Xenobiotic Efflux; ABC Membrane SF; P-loop NTPase SF					-2.0	7.10E-03		
ABCB6	Coproporphyrinogen III Transporter (23792964); ABC Membrane SF					-1.6	5.08E-03		
ABCC4L2*	Orphan Transporter; ABC Membrane SF; P-loop NTPase SF							-3.4	2.05E-10

ABCC4L3*	Orphan Transporter; ABC Membrane SF; P-loop NTPase SF										-23.7	8.32E-19
ABCC4L4*	Orphan Transporter; ABC Membrane SF; P-loop NTPase SF										-12.2	7.35E-07
ADA	Adenosine Metabolism; Metallo-dependent Hydrolases SF	-1.8	1.88E-02									
GGT1	Glutathione Metabolism; G Glu Transpept SF							-1.9	4.29E-02			
HSD17B1	Biosynthesis of Estrogen (27102893); Androgen Catabolism; NADB Rossmann SF	-1.8	4.04E-02									
HSD17B6	Androgen Catabolism (9188497); NADB Rossmann SF	-3.7	1.19E-02									
INPP5K	(-) Regulator of Insulin Signaling (12556481); EEP SF	-1.6	7.22E-03									
OAZ3	Polyamine Metabolism (19076071); Cupredoxin SF; ODC AZ SF										-8.8	2.23E-02
SLC12A7	Potassium-chloride Cotransporter; Solute Carrier SF	-1.5	2.28E-02									
SLC13A1	Sulfate Transporter (11161786); Solute Carrier SF	-1.5	4.47E-02									
SLC14A1	Urea Transporter; UT SF; Solute Carrier SF	-13.9	1.46E-02								-7.9	9.93E-11
SLC16A14	Orphan Transporter; Solute Carrier SF				-1.7	1.09E-02						
SLC16A5	Orphan Monocarboxylate Transporter; Solute Carrier SF	-2.1	1.59E-03					-1.8	9.64E-03			
SLC22A10L3*	Orphan Transporter; Major Facilitator SF; Solute Carrier SF										-14.0	1.75E-03
SLC24A1	Sodium/Calcium Transport (7539424); Solute Carrier SF; Na Ca Ex SF				-2.9	1.16E-03						
SLC24A4	Sodium/Calcium Transport; Solute Carrier SF; Na Ca Ex SF	-4.4	2.68E-06		-1.8	4.67E-02		-2.4	1.05E-02		-4.9	8.53E-03
SLC25A27	Adenine Transporter; Solute Carrier SF							-1.6	7.18E-03			
SLC25A5	Mitochondrial Proton Transport; Solute Carrier SF	-1.5	2.24E-02									
SLC25A53	Mitochondrial Protein; Orphan Transporter (23266187); Solute Carrier SF										-3.5	2.19E-02
SLC25A6	Adenine Transporter; Solute Carrier SF	-1.5	2.26E-02									
SLC44A4	Choline Transporter; Solute Carrier SF	-1.6	3.27E-03									
SLC46A2	Peptidoglycan Transporter (28539433); Solute Carrier SF	-2.3	6.48E-03									
SLC46A3	Orphan Transporter; Solute Carrier SF	-2.8	2.40E-06		-1.8	2.05E-03						
SLC4A7	Bicarbonate Cotransporter; HCO3- Cotransporter SF; Solute Carrier SF										-2.0	2.41E-02
SLC51A	Bile Acid Transporter (18292224) ; Solute Carrier SF	-6.9	2.76E-08					-4.8	3.48E-06			
SLC9A2	Sodium/Hydrogen Ion Transporter (26350456); Solute Carrier SF	-1.9	1.78E-04					-1.7	1.92E-03			
SLC9A3R1	(+) Regulator of Inflammation (24196963); PDZ SF; EBP50 C-term SF	-1.5	1.36E-02									
SULT1B1	Phase II Conjugating Enzyme; Sulfotransfer 3 SF				-1.5	3.48E-02						
UGT2C1*	Phase II Conjugating Enzyme; Glycosyltransferase GTB-type Superfamily				-1.6	4.13E-02						
