# Supplementary material for: Molecular and metabolomic changes in the proximal colon of pigs infected with Trichuris suis
Source: Sci Rep. 2020 Jul 30;10:12853. doi: 10.1038/s41598-020-69462-5 (PMC7393168; doi:10.1038/s41598-020-69462-5)
Supplement: Supplementary file 11 — Supplementary Table S11. [file 41598_2020_69462_MOESM11_ESM.pdf]

Molecular and metabolomic changes in the proximal colon of pigs infected with *Trichuris suis*

Harry Dawson[1], Celine Chen[1], Robert Li[2], Lauren Nicki Bell[3], Terez Shea-Donohue[4], Helene Kringel[5], Ethiopia Beshah[1], Dolores E. Hill[2], Joseph F. Urban Jr[1,2].

[1]United States Department of Agriculture, Agricultural Research Service, Northeast Area, Beltsville Human Nutrition Research Center, Diet Genomics and Immunology Laboratory, [2] Beltsville Agricultural Research Center, Animal Parasitology Disease Laboratory, Beltsville, MD; [3]Metabolon, Inc., Morrisville, NC; [4]University of Maryland School of Medicine, Baltimore, MD, USA; [5]Department of Veterinary Disease Biology, Faculty of Health and Medical Sciences, University of Copenhagen, Copenhagen, Denmark

Supplemental Table S11

Supplemental Table S11. Metabolic markers of *Trichuris suis* infection proximal colon tissue 33 dpi

Heat map of statistically significant biochemicals profiled in this study.

Comparison mean values significantly different:
- (red) $p \le 0.05$, fold of change ≥ 1.00
- (green) $p \le 0.05$, fold of change < 1.00

Comparison mean values approaching significance:
- (pink) $0.05 < p < 0.10$, fold of change ≥ 1.00
- (light green) $0.05 < p < 0.10$, fold of change < 1.00

Pathway Sort Order	Super Pathway	Sub Pathway	Biochemical Name	Platform	Comp ID	KEGG	HMDB	Fold of Change: Welch's Two-Sample t-Test, Infected / Non-Infected	Statistical Values: Welch's Two-Sample t-Test, Infected / Non-Infected, p-Value	q-Value	Mean Values: Non-Infected	Mean Values: Infected	% Filled Values: Non-Infected	% Filled Values: Infected	Super Pathway	Sub Pathway	PubChem	CAS	RI	Mass
1	Amino acid	Glycine, serine and threonine metabolism	glycine	GC/MS	11777	C00037	HMDB00123	1.39	0.0099	0.0697	0.9159	1.2731	100	100	Amino acid	Glycine, serine and threonine metabolism	5257127;750	56-40-6;	1166	101.9
2			sarcosine (N-Methylglycine)	GC/MS	1516	C00213	HMDB00271	1.43	0.0054	0.0494	0.9033	1.2879	100	100	Amino acid	Glycine, serine and threonine metabolism	1088;7311726	107-97-1;	1182.9	116
8			serine	GC/MS	1648	C00065	HMDB00187	1.14	0.1446	0.3086	0.9303	1.0621	100	100	Amino acid	Glycine, serine and threonine metabolism	5951;6857581	56-45-1;	1389.1	204
17			threonine	LC/MS pos	1284	C00188	HMDB00167	0.82	0.0329	0.1354	1.0939	0.8941	100	100	Amino acid	Glycine, serine and threonine metabolism	6971019;6288	72-19-5;	713	120.1
18			N-acetylthreonine	LC/MS neg	33939	C01118		0.95	0.5938	0.6139	1.0107	0.9643	100	100	Amino acid	Glycine, serine and threonine metabolism	4651717	17093-74-2;	846	160.1
21			betaine	LC/MS pos	3141	C00719	HMDB00043	1.29	0.0925	0.2277	0.8621	1.1114	100	100	Amino acid	Glycine, serine and threonine metabolism	247	107-43-7;	721	118.2
24		Alanine and aspartate metabolism	aspartate	GC/MS	15996	C00049	HMDB00191	1.20	0.0697	0.1960	0.9144	1.0957	100	100	Amino acid	Alanine and aspartate metabolism	5960	56-84-8;	1529.7	232
25			asparagine	GC/MS	11398	C00152	HMDB00168	1.07	0.6201	0.6226	1.0459	1.1202	100	100	Amino acid	Alanine and aspartate metabolism	6267;6992089	70-47-3;	1651.2	231
28			beta-alanine	GC/MS	55	C00099	HMDB00056	0.87	0.8148	0.6977	1.1333	0.9866	100	100	Amino acid	Alanine and aspartate metabolism	239;4755801	56-41-7;107-95-9;	1451.8	174
32			alanine	GC/MS	1126	C00041	HMDB00161	1.29	0.0131	0.0840	0.8843	1.1382	100	100	Amino acid	Alanine and aspartate metabolism	5950;7311724	56-41-7;	1147.6	115.9
33			N-acetylalanine	LC/MS neg	1585	C02847	HMDB00766	1.10	0.2848	0.4400	0.9321	1.0219	100	100	Amino acid	Alanine and aspartate metabolism	88064	97-69-8;	882	130.1
35			N-acetylaspartate (NAA)	GC/MS	22185	C01042	HMDB00812	0.90	0.9791	0.7469	1.3260	1.1878	78	100	Amino acid	Alanine and aspartate metabolism	65065	997-55-7;997-55-7 ;	1648.5	274
39		Glutamate metabolism	glutamate	GC/MS	57	C00025	HMDB03339	1.21	0.0268	0.1249	0.9276	1.1234	100	100	Amino acid	Glutamate metabolism	611	56-86-0;	1611.9	246
45			glutamine	LC/MS pos	53	C00064	HMDB00641	1.02	0.6814	0.6517	0.9718	0.9914	100	100	Amino acid	Glutamate metabolism	5961	56-85-9;	684	147.2
47			gamma-aminobutyrate (GABA)	GC/MS	1416	C00334	HMDB00112	0.59	0.0072	0.0593	1.5358	0.9028	100	100	Amino acid	Glutamate metabolism	6992099;119	56-12-2;	1539.7	304.1
51			N-acetyl-aspartyl-glutamate (NAAG)	LC/MS pos	35665	C12270	HMDB01067	0.92	0.1903	0.3551	1.0181	0.9330	100	100	Amino acid	Glutamate metabolism	5255	3106-85-2;	1563	305
53		Histidine metabolism	histidine	LC/MS neg	59	C00135	HMDB00177	0.97	0.4643	0.5473	1.0232	0.9898	100	100	Amino acid	Histidine metabolism	773;3651426	5934-29-2;	757	154.1
57			1-methylhistidine	GC/MS	30460	C01152	HMDB00001	1.47	0.0286	0.1264	0.9137	1.3401	89	100	Amino acid	Histidine metabolism	92105;7020397	332-80-9;	1757.7	196
58			histamine	LC/MS pos	1574	C00388	HMDB00870	0.79	0.1525	0.3197	1.1698	0.9270	100	100	Amino acid	Histidine metabolism	774	51-45-6;	570	112.2
59			1-methylhistamine	GC/MS	32441	C05127	HMDB00898	1.19	0.0969	0.2364	0.9073	1.0816	100	100	Amino acid	Histidine metabolism	3614	501-75-7 ;	1725	174.1
65			imidazole propionate	LC/MS pos	40730		HMDB02271	4.67	0.0564	0.1852	0.3909	1.8243	11	45	Amino acid	Histidine metabolism	70630	1074-59-5;	1011.7	141.1
75		Lysine metabolism	lysine	LC/MS pos	1301	C00047	HMDB00182	0.85	0.0386	0.1490	1.0227	0.8651	100	100	Amino acid	Lysine metabolism	5962	56-87-1;	603	147.2
77			2-aminoadipate	LC/MS pos	6146	C00956	HMDB00510	0.89	0.4438	0.5366	1.1835	1.0543	100	100	Amino acid	Lysine metabolism	469	542-32-5;1118-90-7;	794	162.1
80			pipecolate	LC/MS pos	1444	C00408	HMDB00070	1.96	0.0130	0.0840	0.8857	1.7332	100	100	Amino acid	Lysine metabolism	849	4043-87-2;	1120	130.1
85			N6-acetyllysine	LC/MS pos	36752	C02727	HMDB00206	1.00	0.9708	0.7469	1.0323	1.0307	100	100	Amino acid	Lysine metabolism	6991978;92832	692-04-6;	1134	189.1
89		Phenylalanine & tyrosine metabolism	phenylalanine	LC/MS pos	64	C00079	HMDB00159	0.96	0.4453	0.5366	1.0491	1.0103	100	100	Amino acid	Phenylalanine & tyrosine metabolism	6925665;6140	63-91-2;	2056	166.1
92			phenylacetate	LC/MS neg	15958	C07086	HMDB00209	0.48	0.0011	0.0151	1.5789	0.7650	100	100	Amino acid	Phenylalanine & tyrosine metabolism		103-82-2;	2127	135.1
94			p-cresol sulfate	LC/MS neg	36103	C01468	HMDB11635	0.85	0.0828	0.2200	1.1028	0.9358	100	100	Amino acid	Phenylalanine & tyrosine metabolism	4615422	3233-57-7;	2896	187.1
106			tyrosine	LC/MS pos	1299	C00082	HMDB00158	0.96	0.3868	0.5079	1.0358	0.9895	100	100	Amino acid	Phenylalanine & tyrosine metabolism	6057;6942100	60-18-4;	1516	182.1
107			3-(4-hydroxyphenyl)lactate	LC/MS neg	32197	C03672	HMDB00755	0.88	0.2637	0.4250	1.1230	0.9836	100	100	Amino acid	Phenylalanine & tyrosine metabolism	9378	6482-98-0;	1395	181.1
124			4-hydroxyphenylacetate	GC/MS	541	C00642	HMDB00020	2.07	0.0195	0.1000	0.5715	1.1804	56	82	Amino acid	Phenylalanine & tyrosine metabolism	4693933	156-38-7;	1630.6	179
134			phenylacetylglycine	LC/MS neg	33945	C05598	HMDB00821	0.97	0.7949	0.6923	1.0824	1.0537	100	100	Amino acid	Phenylalanine & tyrosine metabolism	68144	500-98-1;	2377	192.1
138			3-(3-hydroxyphenyl)propionate	LC/MS neg	35635	C11457	HMDB00375	1.42	0.5175	0.5812	0.9896	1.4062	78	73	Amino acid	Phenylalanine & tyrosine metabolism	91	621-54-5;	2000	165.1
140			3-phenylpropionate (hydrocinnamate)	LC/MS neg	15749	C05629	HMDB00764	0.57	0.0053	0.0494	1.2867	0.7297	100	100	Amino acid	Phenylalanine & tyrosine metabolism		501-52-0;	2830	149.1
152		Tryptophan metabolism	kynurenine	LC/MS pos	15140	C00328	HMDB00684	1.09	0.5959	0.6139	1.0416	1.1307	89	91	Amino acid	Tryptophan metabolism	161166;6971029	2922-83-0;	1902	209.1
153			tryptophan	LC/MS pos	54	C00078	HMDB00929	0.88	0.0282	0.1264	1.0838	0.9509	100	100	Amino acid	Tryptophan metabolism	6923516;6305	73-22-3;	2445	205.1
156			indoleacetate	LC/MS pos	27513	C00954	HMDB00197	2.18	0.0537	0.1809	0.5530	1.2038	56	82	Amino acid	Tryptophan metabolism	802	6505-45-9;	3760	176.1
160.1			5-hydroxytryptophol	GC/MS	41498		HMDB01855	0.52	0.0868	0.2246	1.5291	0.8004	100	100	Amino acid	Tryptophan metabolism	9061	154-02-9;	1956	290.1
172			C-glycosyltryptophan*	LC/MS pos	32675			1.15	0.1651	0.3278	0.9561	1.1026	100	100	Amino acid	Tryptophan metabolism			1912	367.1
179			3-indoxyl sulfate	LC/MS neg	27672		HMDB00682	0.80	0.8613	0.7207	1.4758	1.1850	100	100	Amino acid	Tryptophan metabolism	10258	2642-37-7;	2258	212
190		Valine, leucine and isoleucine metabolism	isoleucine	LC/MS pos	1125	C00407	HMDB00172	0.93	0.1092	0.2554	1.0261	0.9567	100	100	Amino acid	Valine, leucine and isoleucine metabolism	791	73-32-5;	1614	132.1
191			leucine	LC/MS pos	60	C00123	HMDB00687	1.01	0.9720	0.7469	1.0249	1.0321	100	100	Amino acid	Valine, leucine and isoleucine metabolism	7045798;6106	61-90-5;	1674	132.2
200			valine	LC/MS pos	1649	C00183	HMDB00883	0.92	0.0678	0.1953	1.0450	0.9589	100	100	Amino acid	Valine, leucine and isoleucine metabolism	6971018;6287	72-18-4;	1040	118.1
210			isobutyrylcarnitine	LC/MS pos	33441		HMDB00736	1.08	0.3964	0.5108	0.9455	1.0250	100	100	Amino acid	Valine, leucine and isoleucine metabolism		25518-49-4;	1941	232.2
212			2-methylbutyrylcarnitine (C5)	LC/MS pos	35431		HMDB00378	0.95	0.9743	0.7469	1.0702	1.0206	100	100	Amino acid	Valine, leucine and isoleucine metabolism	6426901	31023-25-3;	2439	246.1
215			isovalerylcarnitine	LC/MS pos	34407		HMDB00688	0.63	0.0230	0.1131	1.4649	0.9209	100	100	Amino acid	Valine, leucine and isoleucine metabolism	6426851	31023-24-2;	2533	246.2
216			hydroxyisovaleroyl carnitine	LC/MS pos	35433			0.86	0.2096	0.3702	1.0988	0.9493	100	100	Amino acid	Valine, leucine and isoleucine metabolism		99159-87-2;	1683	262.1
224		Cysteine, methionine, SAM, taurine metabolism	cysteine	GC/MS	31453	C00097	HMDB00574	1.05	0.9059	0.7356	1.0521	1.1032	100	100	Amino acid	Cysteine, methionine, SAM, taurine metabolism	5862;6419722	52-90-4;56-89-3;	1560.1	218
227			S-methylcysteine	GC/MS	40262		HMDB02108	1.31	0.0833	0.2200	0.9961	1.3026	100	100	Amino acid	Cysteine, methionine, SAM, taurine metabolism	7058174;24417	1187-84-4;	1447.8	162.1
228			cystine	GC/MS	31454	C00491	HMDB00192	1.18	0.5837	0.6132	0.7465	0.8782	44	73	Amino acid	Cysteine, methionine, SAM, taurine metabolism	595	56-89-3;	2015.3	218
229			cystathionine	GC/MS	15705	C02291	HMDB00099	3.34	0.0000	0.0001	0.5397	1.8019	100	100	Amino acid	Cysteine, methionine, SAM, taurine metabolism	4083111;834	535-34-2;	1979.6	218.1
230			methionine sulfoxide	LC/MS pos	18374	C02989	HMDB02005	0.73	0.0084	0.0617	1.1166	0.8157	100	82	Amino acid	Cysteine, methionine, SAM, taurine metabolism	158980;1548907	3226-65-1;	729	166.1
232			hypotaurine	GC/MS	590	C00519	HMDB00965	0.96	0.9418	0.7466	1.0314	0.9928	100	100	Amino acid	Cysteine, methionine, SAM, taurine metabolism	107812	300-84-5;	1598.5	188
233			taurine	LC/MS pos	32975	C00245	HMDB00251	0.78	0.0807	0.2176	1.1358	0.8908	100	100	Amino acid	Cysteine, methionine, SAM, taurine metabolism	1123;4068592	107-35-7;	687	126.1
236			S-adenosylhomocysteine (SAH)	LC/MS neg	15948	C00021	HMDB00939	1.15	0.6484	0.6435	1.0515	1.2051	100	91	Amino acid	Cysteine, methionine, SAM, taurine metabolism		979-92-0;	1821	383.1
237			methionine	LC/MS pos	1302	C00073	HMDB00696	0.94	0.3680	0.4959	1.0270	0.9703	100	100	Amino acid	Cysteine, methionine, SAM, taurine metabolism	6992087;6137	63-68-3;	1252	150.1
239			N-acetylmethionine	LC/MS neg	1589	C02712	HMDB11745	1.13	0.0631	0.1915	0.9717	1.0940	100	100	Amino acid	Cysteine, methionine, SAM, taurine metabolism	448580	65-82-7;	1805	190.1
241			2-hydroxybutyrate (AHB)	GC/MS	21044	C05984	HMDB00008	0.91	0.2824	0.4400	0.9941	0.9029	100	82	Amino acid	Cysteine, methionine, SAM, taurine metabolism	440864	3347-90-8;	1169.4	130.9
243			homocysteine	GC/MS	40266	C00155	HMDB00742	1.20	0.3303	0.4720	0.8002	0.9623	78	100	Amino acid	Cysteine, methionine, SAM, taurine metabolism	778	454-29-5;	1648.2	234
254		Urea cycle; arginine-, proline-, metabolism	dimethylarginine (SDMA + ADMA)	LC/MS pos	36808	C03626	HMDB01539	0.99	0.8552	0.7203	0.9634	0.9539	100	100	Amino acid	Urea cycle; arginine-, proline-, metabolism	123831	102783-24-4;	812	203.2
255			arginine	LC/MS pos	1638	C00062	HMDB00517	0.96	0.2640	0.4250	1.0159	0.9754	100	100	Amino acid	Urea cycle; arginine-, proline-, metabolism	5246487;232	1119-34-2;	650	175.2
258			ornithine	GC/MS	1493	C00077	HMDB03374	1.22	0.2020	0.3647	0.9085	1.1039	100	100	Amino acid	Urea cycle; arginine-, proline-, metabolism	6262	3184-13-2;	1763.8	141.9
260			urea	GC/MS	1670	C00086	HMDB00294	1.12	0.4200	0.5220	2.7828	3.1248	89	91	Amino acid	Urea cycle; arginine-, proline-, metabolism	1176;16150869	57-13-6;	1223.9	171
261			proline	LC/MS pos	1898	C00148	HMDB00162	1.04	0.6531	0.6457	1.0059	1.0417	100	100	Amino acid	Urea cycle; arginine-, proline-, metabolism	145742;6971047	147-85-3;	796	116.1
262			5-aminovalerate	GC/MS	18319	C00431	HMDB03355	3.55	0.0007	0.0124	0.8308	2.9530	100	100	Amino acid	Urea cycle; arginine-, proline-, metabolism	6992101;138	660-88-8;	1620.8	174
263			citrulline	LC/MS pos	2132	C00327	HMDB00904	1.09	0.3811	0.5050	1.0813	1.1738	100	100	Amino acid	Urea cycle; arginine-, proline-, metabolism	833	372-75-8;	715	176.1
264			N-acetylornithine	LC/MS pos	15630	C00437	HMDB03357	1.74	0.0495	0.1688	1.0447	1.8209	100	100	Amino acid	Urea cycle; arginine-, proline-, metabolism	6992102;439232	6205-08-9;	875	175.2
267			trans-4-hydroxyproline	LC/MS pos	32306	C01157	HMDB00725	1.32	0.0446	0.1587	0.8038	1.0618	100	100	Amino acid	Urea cycle; arginine-, proline-, metabolism	5810;6971053	51-35-4;	705	132.1
270			argininosuccinate	GC/MS	15497	C03406	HMDB00052	0.82	0.3596	0.4923	1.0278	0.8449	67	73	Amino acid	Urea cycle; arginine-, proline-, metabolism	828	156637-58-0;	2157	446.3
274		Creatine metabolism	creatine	LC/MS pos	27718	C00300	HMDB00064	1.02	0.7652	0.6850	0.9872	1.0039	100	100	Amino acid	Creatine metabolism	586	57-00-1;	758	132.1
275			creatinine	LC/MS pos	513	C00791	HMDB00562	0.99	0.9960	0.7479	1.0175	1.0094	100	100	Amino acid	Creatine metabolism	588	60-27-5;	730	114.1
278		Butanoate metabolism	2-aminobutyrate	GC/MS	1577	C02261	HMDB00650	1.82	0.0007	0.0124	0.7497	1.3617	100	100	Amino acid	Butanoate metabolism	439691;6971251	1492-24-6;	1215.7	130
282		Polyamine metabolism	putrescine	GC/MS	1408	C00134	HMDB01414	0.99	0.4009	0.5129	1.3355	1.3282	100	100	Amino acid	Polyamine metabolism		110-60-1;	1705.8	174
285			spermidine	LC/MS pos	485	C00315	HMDB01257	0.77	0.0767	0.2136	1.2667	0.9703	100	100	Amino acid	Polyamine metabolism	1102	124-20-9;	533	146.2
295		Glutathione metabolism	glutathione, reduced (GSH)	LC/MS pos	2127	C00051	HMDB00125	3.01	0.0176	0.0959	0.6898	2.0767	100	100	Amino acid	Glutathione metabolism	124886	70-18-8;	1274	308.1
297			5-oxoproline	LC/MS pos	1494	C01879	HMDB00267	0.90	0.2933	0.4471	1.0976	0.9889	100	100	Amino acid	Glutathione metabolism	7405	98-79-3;	1446	130.1
298			glutathione, oxidized (GSSG)	LC/MS pos	27727	C00127	HMDB03337	1.08	0.3826	0.5050	0.9405	1.0124	100	100	Amino acid	Glutathione metabolism	65359;11215652	103239-24-3;	1535	613.1
299			cysteine-glutathione disulfide	LC/MS pos	35159		HMDB00656	0.87	0.7049	0.6611	1.0363	0.8987	100	100	Amino acid	Glutathione metabolism	4247235	13081-14-6;	821	427.1
300			ophthalmate	LC/MS pos	34592		HMDB05765	1.22	0.1520	0.3197	0.9472	1.1560	100	100	Amino acid	Glutathione metabolism	9817431	495-27-2;	1457	290.1
311	Peptide	Dipeptide	glycylvaline	LC/MS pos	18357			0.90	0.5805	0.6132	1.1209	1.0079	89	100	Peptide	Dipeptide	2724807;6994979	1963-21-9;	1572	175.1
312.9			glycylmethionine	LC/MS pos	42012			1.06	0.7772	0.6875	1.0006	1.0623	100	91	Peptide	Dipeptide	96757	554-94-9;	1593	207.1
314			glycylproline	LC/MS pos	22171		HMDB00721	1.16	0.2470	0.4134	0.9161	1.0599	100	100	Peptide	Dipeptide	3013625;6993386	704-15-4;	1115	173.1
316			glycylleucine	LC/MS pos	34398	C02155	HMDB00759	1.00	0.9928	0.7479	0.9932	0.9912	100	100	Peptide	Dipeptide	92843;1548899	869-19-2;	2236	189.1
320			glycyltyrosine	LC/MS pos	33958			0.88	0.5824	0.6132	1.0775	0.9498	100	100	Peptide	Dipeptide	92829;6994980	658-79-7;	1728	239.1
333			alanylglycine	GC/MS	37075		HMDB06899	0.90	0.5925	0.6139	1.1058	0.9943	100	91	Peptide	Dipeptide	5242526;79094	687-69-4;	1412	272.1
360			leucylleucine	LC/MS pos	36756	C11332		0.49	0.0691	0.1960	1.1921	0.5783	67	45	Peptide	Dipeptide	76807;6992072	3303-31-9;	3012	245.1
363			pro-hydroxy-pro	LC/MS pos	35127		HMDB06695	0.81	0.5809	0.6132	1.3005	1.0478	100	100	Peptide	Dipeptide	11673055	18684-24-7;	960	229.2
376			cysteinylglycine	GC/MS	35637	C01419	HMDB00078	1.11	0.5632	0.6085	0.9529	1.0614	100	100	Peptide	Dipeptide	65270	19246-18-5;	1763	257
406			isoleucylglycine	LC/MS pos	40008			1.12	0.5166	0.5812	0.8638	0.9685	100	91	Peptide	Dipeptide	342532	868-28-0;	1732	189.2
413			leucylalanine	LC/MS pos	40010			0.73	0.3249	0.4720	0.9595	0.7033	44	45	Peptide	Dipeptide	259321	7298-84-2;	1760	203.2
418			leucylglycine	LC/MS pos	40045			0.67	0.4037	0.5129	1.2188	0.8206	78	82	Peptide	Dipeptide	79070	686-50-0;	1800	189.2
438.9			tyrosylglycine	LC/MS pos	41375			1.07	0.7416	0.6826	0.9608	1.0320	100	100	Peptide	Dipeptide	259323	673-05-5;	1596.8	239.1
455		Dipeptide derivative	carnosine	LC/MS neg	1768	C00386	HMDB00033	0.87	0.3617	0.4926	1.0225	0.8887	100	100	Peptide	Dipeptide derivative	439224;6992100	305-84-0;	813	225.2
460		gamma-glutamyl	gamma-glutamylvaline	LC/MS pos	32393		HMDB11172	0.82	0.2343	0.3999	1.1251	0.9282	89	100	Peptide	gamma-glutamyl		2746-34-1;	2040	247.2
462			gamma-glutamylleucine	LC/MS pos	18369		HMDB11171	0.76	0.0982	0.2372	1.1004	0.8347	100	100	Peptide	gamma-glutamyl	151023	2566-39-4;	2744	261.2
465			gamma-glutamylglycine	GC/MS	33949		HMDB11667	1.39	0.2848	0.4400	0.8560	1.1861	78	91	Peptide	gamma-glutamyl	165527	1948-29-4;	1934	375.2
466			gamma-glutamylmethionine	LC/MS pos	37539			0.69	0.0394	0.1490	1.1887	0.8232	100	100	Peptide	gamma-glutamyl	7009567	17663-87-5;	1993	279.2
467			gamma-glutamylglutamate	LC/MS pos	36738			0.91	0.4480	0.5366	1.0561	0.9598	100	100	Peptide	gamma-glutamyl	92865	1116-22-9;	1036	277.1
468			gamma-glutamylglutamine	LC/MS pos	2730		HMDB11738	0.86	0.2358	0.3999	1.0965	0.9380	100	100	Peptide	gamma-glutamyl	150914	10148-81-9;	806	276.1
470			gamma-glutamylphenylalanine	LC/MS pos	33422		HMDB00594	0.73	0.1709	0.3366	1.1997	0.8709	100	100	Peptide	gamma-glutamyl	111299	7432-24-8;	2846	295.1
471			gamma-glutamyltyrosine	LC/MS pos	2734			0.69	0.0033	0.0354	1.1373	0.7860	100	82	Peptide	gamma-glutamyl	94340	7432-23-7;	2073	311.2
475			gamma-glutamylalanine	LC/MS pos	37063			1.14	0.3948	0.5108	0.9568	1.0946	100	100	Peptide	gamma-glutamyl	440103	5875-41-2;	1108	219
510	Carbohydrate	Aminosugars metabolism	N-acetylgalactosamine	GC/MS	2766	C01074	HMDB00835	1.99	0.0628	0.1915	0.8163	1.6259	78	91	Carbohydrate	Aminosugars metabolism	35717	14215-68-0;	1887.4	245.1
511			N-acetylglucosamine	GC/MS	15096	C00140	HMDB00215	1.65	0.0407	0.1490	0.8117	1.3391	100	100	Carbohydrate	Aminosugars metabolism	24139	7512-17-6;	1934.5	245
513			N-acetylglucosamine 6-phosphate	GC/MS	15107	C00357	HMDB02817	1.16	0.4138	0.5176	0.8159	0.9465	78	100	Carbohydrate	Aminosugars metabolism	10040501	102029-88-9;	2096.2	387.2
515			erythronate*	GC/MS	33477		HMDB00613	1.10	0.6949	0.6542	0.9912	1.0918	100	100	Carbohydrate	Aminosugars metabolism	2781043	88759-55-1;	1546.9	292.1
517			N-acetylneuraminate	GC/MS	1592	C00270	HMDB00230	2.47	0.0082	0.0617	0.5711	1.4091	89	100	Carbohydrate	Aminosugars metabolism		131-48-6;	2074.9	245
523			fucose	GC/MS	15821	C02095	HMDB00174	2.07	0.0037	0.0375	0.8793	1.8208	100	100	Carbohydrate	Aminosugars metabolism	3034656	2438-80-4;	1682.2	204
525			N-acetylmannosamine	GC/MS	15060	C00140	HMDB00835	2.75	0.0006	0.0123	0.7350	2.0220	67	100	Carbohydrate	Aminosugars metabolism	439281	7772-94-3;	1915.9	245.1
537		Fructose, mannose, galactose, starch, and sucrose metabolism	fructose	GC/MS	577	C00095	HMDB00660	1.20	0.3923	0.5108	0.8617	1.0379	89	100	Carbohydrate	ctose, mannose, galactose, starch, and sucrose metabo	5984	57-48-7;	1758	217
539			galactose	GC/MS	12055	C01582	HMDB00143	2.78	0.0028	0.0331	0.6196	1.7215	100	100	Carbohydrate	ctose, mannose, galactose, starch, and sucrose metabo	3037556	59-23-4;	1793.8	203.9
547			maltose	GC/MS	15806	C00208	HMDB00163	1.51	0.3649	0.4944	1.1267	1.6985	100	100	Carbohydrate	ctose, mannose, galactose, starch, and sucrose metabo	439341	6363-53-7;	2142.1	204.1
548			mannitol	GC/MS	15335	C00392	HMDB00765	1.08	0.8281	0.7067	0.8358	0.9047	78	82	Carbohydrate	ctose, mannose, galactose, starch, and sucrose metabo	6251	69-65-8;	1839	319.1
552			mannose	GC/MS	584	C00159	HMDB00169	1.26	0.2550	0.4213	0.9695	1.2216	100	100	Carbohydrate	ctose, mannose, galactose, starch, and sucrose metabo	161658	3458-28-4;	1753.5	203.9
554			mannose-6-phosphate	GC/MS	1470	C00275	HMDB01078	1.05	0.7464	0.6826	0.9521	0.9989	100	100	Carbohydrate	ctose, mannose, galactose, starch, and sucrose metabolism		70442-25-0;104872-94-8;	2035.9	387.2
560			sorbitol	GC/MS	15053	C00794	HMDB00247	1.42	0.0609	0.1915	0.8346	1.1854	100	100	Carbohydrate	ctose, mannose, galactose, starch, and sucrose metabo	107428	6706-59-8;	1843	319.1
583		Glycolysis, gluconeogenesis, pyruvate metabolism	1,5-anhydroglucitol (1,5-AG)	GC/MS	20675	C07326	HMDB02712	0.93	0.7318	0.6764	1.0040	0.9302	89	73	Carbohydrate	Glycolysis, gluconeogenesis, pyruvate metabolism		154-58-5;	1788.7	217.1
585			glycerate	GC/MS	1572	C00258	HMDB00139	0.76	0.1930	0.3555	1.2850	0.9777	100	100	Carbohydrate	Glycolysis, gluconeogenesis, pyruvate metabolism	752	600-19-1;	1360.7	189
587			glucose-6-phosphate (G6P)	GC/MS	31260	C00668	HMDB01401	1.04	0.6597	0.6484	0.9809	1.0226	100	100	Carbohydrate	Glycolysis, gluconeogenesis, pyruvate metabolism		103192-55-8;	2042.7	387.2
589			glucose	GC/MS	20488	C00031	HMDB00122	1.07	0.5434	0.5946	1.0269	1.0968	100	100	Carbohydrate	Glycolysis, gluconeogenesis, pyruvate metabolism	79025	50-99-7;	1866.8	217.1
592			fructose-6-phosphate	GC/MS	12021	C05345	HMDB00124	0.96	0.9357	0.7444	1.0084	0.9729	100	100	Carbohydrate	Glycolysis, gluconeogenesis, pyruvate metabolism		103213-47-4;	1994.9	315.1
597			3-phosphoglycerate	GC/MS	40264	C00597	HMDB00807	0.46	0.0250	0.1185	2.1428	0.9801	100	100	Carbohydrate	Glycolysis, gluconeogenesis, pyruvate metabolism		80731-10-8;	1754	357.1
602			phosphoenolpyruvate (PEP)	GC/MS	597	C00074	HMDB00263	0.54	0.1635	0.3270	1.2825	0.6868	89	73	Carbohydrate	Glycolysis, gluconeogenesis, pyruvate metabolism	1005	10526-80-4;	1595.4	369
604			lactate	GC/MS	527	C00186	HMDB00190	1.30	0.0061	0.0523	0.8663	1.1249	100	100	Carbohydrate	Glycolysis, gluconeogenesis, pyruvate metabolism	612	79-33-4;	1102.8	116.9
607			glucuronate	GC/MS	15443	C00191	HMDB00127	0.92	0.6126	0.6187	1.0578	0.9687	100	100	Carbohydrate	Glycolysis, gluconeogenesis, pyruvate metabolism	65041	207300-70-7;	1888	217
614		Nucleotide sugars, pentose metabolism	arabitol	GC/MS	38075	C00474	HMDB01851	1.55	0.0113	0.0760	0.7493	1.1617	78	100	Carbohydrate	Nucleotide sugars, pentose metabolism	94154	7643-75-6;	1687.5	307.1
616			ribitol	GC/MS	15772	C00474	HMDB00508	1.23	0.1548	0.3197	0.9335	1.1492	89	100	Carbohydrate	Nucleotide sugars, pentose metabolism		488-81-3;	1692.4	217
619			sedoheptulose-7-phosphate	GC/MS	35649	C05382	HMDB01068	1.17	0.1627	0.3270	0.9472	1.1068	100	100	Carbohydrate	Nucleotide sugars, pentose metabolism	616	2646-35-7;	2070	691.35
622			ribose	GC/MS	12080	C00121	HMDB00283	1.45	0.0787	0.2144	0.9995	1.4503	100	100	Carbohydrate	Nucleotide sugars, pentose metabolism	5311110	50-69-1;	1639.2	204
627			ribulose	GC/MS	35855	C00309	HMDB00621	1.11	0.5250	0.5845	0.8701	0.9645	100	100	Carbohydrate	Nucleotide sugars, pentose metabolism	79021	488-84-6;	1662	306.1
629			Isobar: ribulose 5-phosphate, xylulose 5-phosphate	GC/MS	37288			0.96	0.6665	0.6484	1.2680	1.2167	100	100	Carbohydrate	Nucleotide sugars, pentose metabolism			1935	357.2
633			xylitol	GC/MS	4966	C00379	HMDB02917	0.84	0.2023	0.3647	1.0269	0.8644	100	100	Carbohydrate	Nucleotide sugars, pentose metabolism	6912	87-99-0;	1677.6	307.2
639			xylulose	GC/MS	18344	C00310	HMDB00654	1.06	0.5244	0.5845	0.9896	1.0472	89	100	Carbohydrate	Nucleotide sugars, pentose metabolism	5289590	551-84-8;	1668	306.1
648	Energy	Krebs cycle	citrate	GC/MS	1564	C00158	HMDB00094	0.49	0.0007	0.0124	1.7883	0.8795	100	100	Energy	Krebs cycle	311	77-92-9;	1763.4	273.1
649			2-methylcitrate	GC/MS	37483	C02225	HMDB00379	0.37	0.0000	0.0008	1.6467	0.6061	100	91	Energy	Krebs cycle	5460420	6061-96-7;	1778	287.1
651			cis-aconitate	LC/MS neg	12025	C00417	HMDB00072	0.67	0.0023	0.0275	1.2541	0.8393	100	100	Energy	Krebs cycle	643757	585-84-2;	603	173.1
659			succinate	GC/MS	1437	C00042	HMDB00254	0.87	0.3438	0.4775	1.2402	1.0806	100	100	Energy	Krebs cycle	1110	110-15-6;	1348	247
663			fumarate	GC/MS	1643	C00122	HMDB00134	0.79	0.0572	0.1854	1.1675	0.9224	100	100	Energy	Krebs cycle		100-17-8;	1382.1	245
667			malate	GC/MS	1303	C00149	HMDB00156	0.86	0.0402	0.1490	1.1147	0.9576	100	100	Energy	Krebs cycle	525	6915-15-7;	1502	233
670		Oxidative phosphorylation	acetylphosphate	GC/MS	15488	C00227	HMDB01494	0.97	0.8119	0.6977	1.0555	1.0220	100	100	Energy	Oxidative phosphorylation	186	94249-01-1;	1263	211
671			phosphate	GC/MS	11438	C00009	HMDB01429	1.03	0.6932	0.6542	0.9902	1.0153	100	100	Energy	Oxidative phosphorylation	1061	7664-38-2 ;	1307.7	298.9
672			pyrophosphate (PPi)	GC/MS	2078	C00013	HMDB00250	0.91	0.9797	0.7469	0.9996	0.9076	100	100	Energy	Oxidative phosphorylation	644102	1466-09-3;2466-09-3;	1642.3	451
673		Essential fatty acid	linoleate (18:2n6)	LC/MS neg	1105	C01595	HMDB00673	1.04	0.8836	0.7286	1.0608	1.1056	100	100	Lipid	Essential fatty acid	5280450	60-33-3;	5533	279.3
677			linolenate [alpha or gamma; (18:3n3 or 6)]	LC/MS neg	34035	C06427	HMDB01388	1.15	0.8947	0.7319	1.2157	1.3937	100	100	Lipid	Essential fatty acid			5450	277.3
678			dihomo-linolenate (20:3n3 or n6)	LC/MS neg	35718	C03242	HMDB02925	0.76	0.1104	0.2554	1.2303	0.9384	100	100	Lipid	Essential fatty acid	5312529	17046-59-2 ;	5600	305.4
679			eicosapentaenoate (EPA; 20:5n3)	LC/MS neg	18467	C06428	HMDB01999	0.80	0.4485	0.5366	1.1683	0.9369	100	100	Lipid	Essential fatty acid	446284	10-2005-9;10417-94-4;	5442	301.3
680			docosapentaenoate (n3 DPA; 22:5n3)	LC/MS neg	32504	C16513	HMDB01976	0.68	0.0862	0.2246	1.4101	0.9533	100	100	Lipid	Essential fatty acid		2234-74-4 ;	5574	329.4
681			docosapentaenoate (n6 DPA; 22:5n6)	LC/MS neg	37478	C16513	HMDB13123	0.65	0.0632	0.1915	1.2759	0.8349	100	100	Lipid	Essential fatty acid	6441454	25182-74-5;	5625	329.4
682			docosahexaenoate (DHA; 22:6n3)	LC/MS neg	19323	C06429	HMDB02183	0.62	0.0141	0.0844	1.3874	0.8550	100	100	Lipid	Essential fatty acid	445580	6217-54-5;	5518	327.3
684		Short chain fatty acid	valerate	LC/MS neg	33443	C00803	HMDB00892	0.50	0.0031	0.0346	1.4306	0.7159	100	100	Lipid	Short chain fatty acid	7991	109-52-4;	1774	101.2
687		Medium chain fatty acid	caproate (6:0)	LC/MS neg	32489	C01585	HMDB00535	0.93	0.4660	0.5473	1.8109	1.6868	100	100	Lipid	Medium chain fatty acid	8892	142-62-1;	2766	115.2
688			heptanoate (7:0)	LC/MS neg	1644	C17714	HMDB00666	1.23	0.1537	0.3197	0.9145	1.1288	100	100	Lipid	Medium chain fatty acid	8094	111-14-8;	3695	129.2
689			caprylate (8:0)	LC/MS neg	32492	C06423	HMDB00482	1.03	0.6445	0.6421	0.9854	1.0155	100	100	Lipid	Medium chain fatty acid	379	124-07-2;	4367	143.2
690			pelargonate (9:0)	LC/MS neg	12035	C01601	HMDB00847	1.00	0.9516	0.7469	1.0024	1.0058	100	100	Lipid	Medium chain fatty acid	5461016	112-05-0;	4847	157.2
691			caprate (10:0)	LC/MS neg	1642	C01571	HMDB00511	1.13	0.7526	0.6834	1.1375	1.2864	100	100	Lipid	Medium chain fatty acid	2969	112-37-8;	5092	171.2
694			laurate (12:0)	LC/MS neg	1645	C02679	HMDB00638	1.15	0.4997	0.5737	1.0539	1.2159	100	100	Lipid	Medium chain fatty acid	3893	143-07-7;	5288	199.3
698		Long chain fatty acid	myristate (14:0)	LC/MS neg	1365	C06424	HMDB00806	0.97	0.6391	0.6392	1.1274	1.0913	100	100	Lipid	Long chain fatty acid	11005	544-63-8;	5439	227.3
699			myristoleate (14:1n5)	LC/MS neg	32418	C08322	HMDB02000	1.01	0.7581	0.6834	1.0757	1.0890	100	100	Lipid	Long chain fatty acid	5281119	544-64-9 ;	5338	225.3
700			pentadecanoate (15:0)	GC/MS	1361	C16537	HMDB00826	0.65	0.0154	0.0878	1.4243	0.9211	100	100	Lipid	Long chain fatty acid	13849	1002-84-2;10002-84-2;	1853.5	299.2
701			palmitate (16:0)	LC/MS neg	1336	C00249	HMDB00220	0.95	0.6138	0.6187	1.0375	0.9874	100	100	Lipid	Long chain fatty acid	985	57-10-3;	5619	255.3
702			palmitoleate (16:1n7)	LC/MS neg	33447	C08362	HMDB03229	1.08	0.9362	0.7444	1.0625	1.1465	100	100	Lipid	Long chain fatty acid	445638	373-49-9;	5477	253.3
704			margarate (17:0)	LC/MS neg	1121		HMDB02259	1.00	0.9778	0.7469	1.0824	1.0809	100	100	Lipid	Long chain fatty acid	10465	506-12-7;	5733	269.3
705			10-heptadecenoate (17:1n7)	LC/MS neg	33971			0.90	0.5493	0.5984	1.1451	1.0325	100	100	Lipid	Long chain fatty acid	5312435	29743-97-3;	5558	267.3
706			stearate (18:0)	LC/MS neg	1358	C01530	HMDB00827	1.08	0.7199	0.6702	1.0446	1.1242	100	100	Lipid	Long chain fatty acid	5281	57-11-4;	5886	283.4
708			oleate (18:1n9)	GC/MS	1359	C00712	HMDB00207	1.08	0.9802	0.7469	1.1372	1.2298	100	100	Lipid	Long chain fatty acid	445639	112-80-1;	1984.4	339.2
710			cis-vaccenate (18:1n7)	GC/MS	33970	C08367		1.01	0.9151	0.7373	1.0813	1.0911	100	100	Lipid	Long chain fatty acid	5282761	506-17-2;	1987	339.3
716			nonadecanoate (19:0)	LC/MS neg	1356	C16535	HMDB00772	1.12	0.6094	0.6187	1.1326	1.2672	100	100	Lipid	Long chain fatty acid	12591	646-30-0;	6073	297.3
717			10-nonadecenoate (19:1n9)	LC/MS neg	33972			0.92	0.5882	0.6139	1.2911	1.1828	100	100	Lipid	Long chain fatty acid	5312513	73033-09-7;	5775	295.4
719			arachidate (20:0)	GC/MS	1118	C06425	HMDB02212	1.02	0.8771	0.7267	0.9927	1.0124	100	100	Lipid	Long chain fatty acid	10467	506-30-9;	2071.2	369.3
722			eicosenoate (20:1n9 or 11)	LC/MS neg	33587		HMDB02231	1.06	0.8763	0.7267	1.1726	1.2386	100	100	Lipid	Long chain fatty acid			5955	309.4
724			dihomo-linoleate (20:2n6)	LC/MS neg	17805	C16525		0.87	0.2957	0.4480	1.1799	1.0275	100	100	Lipid	Long chain fatty acid	6439848	2091-39-6;	5722	307.3
728			arachidonate (20:4n6)	LC/MS neg	1110	C00219	HMDB01043	0.66	0.0305	0.1311	1.2654	0.8352	100	100	Lipid	Long chain fatty acid	444899	506-32-1;	5525	303.4
732			erucate (22:1n9)	LC/MS neg	1552	C08316	HMDB02068	1.09	0.3789	0.5050	0.9594	1.0467	100	100	Lipid	Long chain fatty acid	8216	112-86-7;	6273.3	337.4
735			adrenate (22:4n6)	LC/MS neg	32980	C16527	HMDB02226	0.73	0.1310	0.2844	1.4003	1.0266	100	100	Lipid	Long chain fatty acid	5282844	2091-25-0;	5684	331.3
762			4-hydroxybutyrate (GHB)	GC/MS	34585	C00989	HMDB00710	0.60	0.0333	0.1354	1.5273	0.9118	100	100	Lipid	Fatty acid, monohydroxy	10413	502-85-2;	1277	233.1
768			3-hydroxydecanoate	LC/MS neg	22053		HMDB02203	1.08	0.2802	0.4400	0.9745	1.0536	100	100	Lipid	Fatty acid, monohydroxy	26612	5561-87-5;	4681	187.2

774		Fatty acid, monohydroxy	2-hydroxystearate	LC/MS neg	17945	C03045		0.85	0.0778	0.2142	1.0803	0.9218	100	100	Lipid	Fatty acid, monohydroxy	69417	629-22-1;	5705	299.4
776			2-hydroxypalmitate	LC/MS neg	35675			0.97	0.8128	0.6977	1.1333	1.0995	100	100	Lipid	Fatty acid, monohydroxy	92836	764-67-0;	5508	271.3
780			13-HODE + 9-HODE	LC/MS neg	37752			1.51	0.3970	0.5108	1.1801	1.7793	100	100	Lipid	Fatty acid, monohydroxy			5270	295.2
782		Fatty acid, dihydroxy	12,13-hydroxyoctadec-9(Z)-enoate	LC/MS neg	38395	C14829	HMDB04705	0.69	0.0401	0.1490	1.2833	0.8828	100	100	Lipid	Fatty acid, dihydroxy	10236635	263399-35-5;	5137	313.4
786			2-hydroxyglutarate	GC/MS	37253	C02630	HMDB00606	0.94	0.3369	0.4736	1.0612	0.9940	100	100	Lipid	Fatty acid, dicarboxylate	43	40951-21-1;	1576	247
793			sebacate (decanedioate)	LC/MS neg	32398	C08277	HMDB00792	0.44	0.0014	0.0184	1.3287	0.5815	100	100	Lipid	Fatty acid, dicarboxylate	5192	111-20-6;	1778	201.2
794			azelate (nonanedioate)	LC/MS neg	18362	C08261	HMDB00784	0.42	0.0009	0.0128	1.7282	0.7244	100	100	Lipid	Fatty acid, dicarboxylate	2266	123-99-9;	1322	187.2
796		Fatty acid, dicarboxylate	dodecanedioate	LC/MS neg	32388	C02678	HMDB00623	0.30	0.0001	0.0045	1.8640	0.5499	100	100	Lipid	Fatty acid, dicarboxylate	12736	693-23-2 ;	2990	229.2
798			hexadecanedioate	LC/MS neg	35678		HMDB00672	0.55	0.0053	0.0494	1.4620	0.8015	89	91	Lipid	Fatty acid, dicarboxylate	10459	505-54-4;	4650	285.2
799			octadecanedioate	LC/MS neg	36754		HMDB00782	0.24	0.0001	0.0045	2.7751	0.6735	100	100	Lipid	Fatty acid, dicarboxylate	70095	871-70-5;	5033	313.3
800			undecanedioate	LC/MS neg	35671		HMDB00888	0.42	0.0003	0.0079	1.4663	0.6219	100	100	Lipid	Fatty acid, dicarboxylate	15816	1852-04-6;	2376	215.1
819		Fatty acid, branched	15-methylpalmitate (isobar with 2-methylpalmitate)	LC/MS neg	38768			0.92	0.4047	0.5129	1.1711	1.0809	100	100	Lipid	Fatty acid, branched			5698	269.4
821			17-methylstearate	LC/MS neg	38296			1.03	0.6127	0.6187	1.1667	1.2002	100	100	Lipid	Fatty acid, branched	3083779	2724-59-6;	5987	297.4
830			prostaglandin A2	LC/MS neg	19761	C05953	HMDB02752	1.48	0.0344	0.1375	0.8451	1.2493	100	100	Lipid	Eicosanoid	5280880	13345-50-1;	4875	333.3
831			prostaglandin B2	LC/MS neg	19499	C05954	HMDB04236	1.02	0.9646	0.7469	1.0324	1.0483	78	82	Lipid	Eicosanoid	5280881	13367-85-6;	4943	333.4
833			prostaglandin E1	LC/MS neg	19391	C04741	HMDB01442	1.05	0.6822	0.6517	0.9443	0.9897	100	91	Lipid	Eicosanoid	5280723	745-65-3;	4600	353.3
834		Eicosanoid	prostaglandin E2	LC/MS neg	7746	C00584	HMDB01220	1.26	0.1311	0.2844	0.9638	1.2118	100	100	Lipid	Eicosanoid	5280360	363-24-6;	4562	351.3
846			13,14-dihydro-15-keto-prostaglandin a2	LC/MS neg	19449		HMDB01244	1.57	0.1136	0.2596	0.7902	1.2424	67	100	Lipid	Eicosanoid		74872-89-2;	4822	333.4
865			6-keto prostaglandin F1alpha	LC/MS neg	20476	C05961	HMDB02886	1.06	0.7560	0.6834	1.0090	1.0707	100	100	Lipid	Eicosanoid	5280888	58961-34-8;58962-34-8;	4180	369.3
873			12-HETE	LC/MS neg	37536		HMDB06111	1.55	0.6653	0.6484	1.2518	1.9432	100	100	Lipid	Eicosanoid		73837-14-6;	5295	319.3
874			15-HETE	LC/MS neg	37538	C04742	HMDB02110	1.12	0.9953	0.7479	1.0007	1.1211	100	100	Lipid	Eicosanoid	5280724	54845-95-3;	5271	319.3
890		Fatty acid metabolism (also BCAA metabolism)	butyrylcarnitine	LC/MS pos	32412			1.05	0.5145	0.5812	1.0817	1.1323	100	100	Lipid	Fatty acid metabolism (also BCAA metabolism)	213144	25576-40-3;	2007	232.2
892		Fatty acid metabolism	valerylcarnitine	LC/MS pos	34406		HMDB13128	1.16	0.2084	0.3702	0.9591	1.1101	100	100	Lipid	Fatty acid metabolism	6426903		2584	246.2
894			isovalerate	LC/MS neg	34732	C08262	HMDB00718	0.31	0.0003	0.0079	1.5931	0.4932	100	100	Lipid	Fatty acid metabolism	10430	503-74-2;	1623	101.2
898			deoxycarnitine	LC/MS pos	36747	C01181	HMDB01161	1.18	0.0149	0.0865	0.9079	1.0741	100	100	Lipid	Carnitine metabolism	725	6249-56-5;	759	146.1
899			carnitine	LC/MS pos	15500	C00318		0.95	0.3223	0.4720	1.0214	0.9691	100	100	Lipid	Carnitine metabolism	10917	461-05-2;	702	162.2
900		Carnitine metabolism	3-dehydrocarnitine*	LC/MS pos	32654	C02636	HMDB12154	1.32	0.3176	0.4720	1.0207	1.3479	100	100	Lipid	Carnitine metabolism	6991982	10457-99-5;	1020	160.2
901			acetylcarnitine	LC/MS pos	32198	C02571	HMDB00201	1.29	0.0597	0.1909	0.8290	1.0699	100	100	Lipid	Carnitine metabolism	7045767	5080-50-2;	1203	204.2
902			hexanoylcarnitine	LC/MS pos	32328		HMDB00705	1.46	0.0164	0.0911	0.8401	1.2246	100	100	Lipid	Carnitine metabolism	6426853	6920-35-0;	3174	260.2
913			stearoylcarnitine	LC/MS pos	34409		HMDB00848	1.47	0.2656	0.4250	0.6083	0.8964	67	91	Lipid	Carnitine metabolism		18822-91-8;	5300	428.4
919.1			glycohyocholate	LC/MS pos	42574			0.63	0.3252	0.4720	1.8265	1.1556	89	73	Lipid	Bile acid metabolism			5115	466.2
920.1			taurohyocholate	LC/MS neg	42603			0.56	0.5382	0.5940	4.0719	2.2846	100	91	Lipid	Bile acid metabolism			5000	514.3
921			chenodeoxycholate	LC/MS neg	37789	C02528	HMDB00518	0.49	0.7115	0.6649	3.9488	1.9202	100	100	Lipid	Bile acid metabolism		474-24-9;474-25-9;	5250	391.4
922			taurochenodeoxycholate	LC/MS neg	18494	C05465	HMDB00951	0.67	0.2111	0.3702	2.0221	1.3506	89	64	Lipid	Bile acid metabolism	387316	6009-98-9;	5218	498.3
924		Bile acid metabolism	hyodeoxycholate	LC/MS neg	27531	C15517	HMDB00733	1.06	0.7440	0.6826	1.1101	1.1723	100	100	Lipid	Bile acid metabolism	5283820	83-49-8;	5074	391.4
935			lithocholate	LC/MS neg	1483	C03990	HMDB00761	0.76	0.1107	0.2554	1.1749	0.8896	100	100	Lipid	Bile acid metabolism	9903	434-13-9;	5350	375.4
939			6-oxolithocholate	LC/MS neg	31902			0.88	0.6773	0.6517	1.1532	1.0149	100	100	Lipid	Bile acid metabolism	164960	2393-61-5 ;	5040	389.4
941			6-beta-hydroxylithocholate	LC/MS neg	36807	C15515	HMDB00811	0.73	0.1872	0.3524	1.4251	1.0336	100	100	Lipid	Bile acid metabolism	5283821	668-49-5;	4826	391.3
944			hyocholate	LC/MS neg	34093	C17649	HMDB00760	0.76	0.4553	0.5412	3.6843	2.8059	100	100	Lipid	Bile acid metabolism		547-75-1;	5001	407.3
947	Lipid		alpha-muricholate	LC/MS neg	34092	C17647	HMDB00506	1.85	0.0420	0.1513	0.7084	1.3117	89	91	Lipid	Bile acid metabolism	3083761	2393-58-0;	4763	407.4
953		Fatty alcohol, long chain	1-octadecanol	GC/MS	21128	D01924	HMDB02350	1.04	0.7787	0.6875	0.9940	1.0299	100	100	Lipid	Fatty alcohol, long chain	8221	112-92-5;	1957.7	327.3
954			choline phosphate	LC/MS pos	34396	C00588		0.93	0.3242	0.4720	1.0306	0.9617	100	100	Lipid	Glycerolipid metabolism	135437	72556-74-2;	695	184.1
955			ethanolamine	GC/MS	1497	C00189	HMDB00149	0.99	0.9014	0.7350	1.1505	1.1413	100	100	Lipid	Glycerolipid metabolism		141-43-5;	1304	174.1
956			phosphoethanolamine	LC/MS neg	1600	C00346	HMDB00224	0.99	0.7704	0.6872	1.0148	1.0023	100	100	Lipid	Glycerolipid metabolism	5232324;1015	1071-23-4;	644	140.1
957			glycerophosphoethanolamine	GC/MS	37455	C01233	HMDB00114	1.62	0.0908	0.2277	0.8557	1.3857	89	100	Lipid	Glycerolipid metabolism	123874	33049-08-0;	1906	357.1
959		Glycerolipid metabolism	glycerol	GC/MS	15122	C00116	HMDB00131	0.98	0.7921	0.6922	1.0569	1.0309	100	100	Lipid	Glycerolipid metabolism	753	56-81-5;	1311	205
960			choline	LC/MS pos	15506	C00114		0.98	0.6667	0.6484	1.0411	1.0247	100	100	Lipid	Glycerolipid metabolism	170746	67-48-1;	674	104.2
961			glycerol 3-phosphate (G3P)	GC/MS	15365	C00093	HMDB00126	1.35	0.2689	0.4276	1.0489	1.4184	100	100	Lipid	Glycerolipid metabolism	754	29849-82-9;	1719.7	357.1
962			glycerophosphorylcholine (GPC)	LC/MS pos	15990	C00670	HMDB00086	1.49	0.1590	0.3258	0.9277	1.3784	100	100	Lipid	Glycerolipid metabolism	657272	28319-77-9;	694	258.1
965			cytidine 5'-diphosphocholine	LC/MS pos	34418	C00307		1.60	0.0280	0.1264	0.7770	1.2439	100	100	Lipid	Glycerolipid metabolism		33818-15-4;	777	489.1
971			myo-inositol	GC/MS	19934	C00137	HMDB00211	0.99	0.8610	0.7207	1.1023	1.0908	100	100	Lipid	Inositol metabolism		87-89-8;	1924.9	217
972			chiro-inositol	GC/MS	37112			0.91	0.8104	0.6977	1.1573	1.0541	100	100	Lipid	Inositol metabolism		643-12-9;	1857	318.1
973		Inositol metabolism	pinitol	GC/MS	37086	C03844		1.42	0.0679	0.1953	0.7953	1.1302	67	100	Lipid	Inositol metabolism		10284-63-6;	1773	318.1
974			inositol 1-phosphate (I1P)	GC/MS	1481	C04006	HMDB00213	0.84	0.1194	0.2682	1.1338	0.9500	100	100	Lipid	Inositol metabolism		106032-59-1;	2057.8	318.1
979			scyllo-inositol	GC/MS	32379	C06153	HMDB06088	1.00	0.7629	0.6850	1.2035	1.1980	100	100	Lipid	Inositol metabolism		488-59-5;	1893.8	318.2
984		Ketone bodies	3-hydroxybutyrate (BHBA)	GC/MS	542	C01089	HMDB00357	0.63	0.0136	0.0844	1.3239	0.8399	100	100	Lipid	Ketone bodies	441	625-72-9;	1203.5	116.9
986			1,2-propanediol	GC/MS	38002	C00583	HMDB01881	0.51	0.0904	0.2277	1.7654	0.9063	100	100	Lipid	Ketone bodies		57-55-6;	1041	117
990			1-palmitoylglycerophosphoethanolamine	LC/MS neg	35631		HMDB11503	0.68	0.4905	0.5683	1.5555	1.0508	100	100	Lipid	Lysolipid	9547069		5940	452.3
991			2-palmitoylglycerophosphoethanolamine*	LC/MS neg	35688			1.06	0.6914	0.6542	1.0239	1.0831	100	100	Lipid	Lysolipid			5790	452.3
995			1-stearoylglycerophosphoethanolamine	LC/MS neg	34416		HMDB11130	0.63	0.2080	0.3702	1.5796	1.0006	100	100	Lipid	Lysolipid	9547068	69747-55-3;	6200	480.4
995.1			2-stearoylglycerophosphoethanolamine*	LC/MS pos	41220			0.75	0.8317	0.7075	1.0783	0.8124	67	82	Lipid	Lysolipid			5779.6	482.2
996			1-oleoylglycerophosphoethanolamine	LC/MS neg	35628		HMDB11506	0.91	0.9338	0.7444	1.2999	1.1892	100	100	Lipid	Lysolipid	9547071	89576-29-4;	5928	478.3
997			2-oleoylglycerophosphoethanolamine*	LC/MS neg	35687			1.31	0.2395	0.4034	0.9055	1.1824	100	100	Lipid	Lysolipid			5848	478.3
999			2-linoleoylglycerophosphoethanolamine*	LC/MS neg	36593			1.25	0.3728	0.4997	0.9310	1.1610	100	100	Lipid	Lysolipid			5650	476.4
1000			1-arachidonoylglycerophosphoethanolamine*	LC/MS neg	35186		HMDB11517	1.25	0.1771	0.3385	0.9598	1.1975	100	100	Lipid	Lysolipid			5731	500.3
1001			2-arachidonoylglycerophosphoethanolamine*	LC/MS neg	32815			1.01	0.9157	0.7373	0.9558	0.9686	100	100	Lipid	Lysolipid			5674	500.3
1003			2-docosapentaenoylglycerophosphoethanolamine*	LC/MS pos	34875			1.55	0.4383	0.5366	0.5836	0.9031	56	82	Lipid	Lysolipid			5548	528.2
1006			1-stearoylglycerophosphoglycerol	LC/MS neg	34437			0.73	0.2312	0.3999	1.5857	1.1612	100	100	Lipid	Lysolipid			5826	511.4
1006.1			2-stearoylglycerophosphoglycerol*	LC/MS neg	41828			0.71	0.0636	0.1915	1.3310	0.9439	100	100	Lipid	Lysolipid			5758.4	511.4
1007			1-myristoylglycerophosphocholine	LC/MS pos	35626		HMDB10379	1.02	0.6762	0.6517	0.7858	0.8021	56	73	Lipid	Lysolipid	460604	20559-16-4;	5481	468.3
1009			1-pentadecanoylglycerophosphocholine*	LC/MS pos	37418			0.87	0.6685	0.6484	1.0030	0.8707	56	64	Lipid	Lysolipid			5563	482.3
1010			1-palmitoylglycerophosphocholine	LC/MS pos	33955			0.79	0.4272	0.5258	1.3848	1.0926	89	100	Lipid	Lysolipid	86554	17364-16-8;	5671	496.4
1011		Lysolipid	2-palmitoylglycerophosphocholine*	LC/MS pos	35253			1.63	0.3305	0.4720	0.5195	0.8493	44	91	Lipid	Lysolipid			5604	496.3
1012			1-palmitoleoylglycerophosphocholine*	LC/MS pos	33230			1.10	0.4761	0.5542	1.1586	1.2742	89	100	Lipid	Lysolipid			5524	494.3
1014			1-heptadecanoylglycerophosphocholine	LC/MS pos	33957		HMDB12108	0.66	0.7233	0.6710	1.1227	0.7397	56	82	Lipid	Lysolipid		50930-23-9;	5750	510.4
1015			1-stearoylglycerophosphocholine	LC/MS pos	33961			0.57	0.5843	0.6132	1.7747	1.0179	67	100	Lipid	Lysolipid	497299	19420-57-6;	5844	524.4
1016			2-stearoylglycerophosphocholine*	LC/MS pos	35255			0.54	0.1759	0.3385	1.1246	0.6078	56	45	Lipid	Lysolipid	10208382		5780	524.4
1017			1-oleoylglycerophosphocholine	LC/MS pos	33960			0.76	0.9911	0.7479	1.1283	0.8520	56	91	Lipid	Lysolipid	16081932	19420-56-5;	5700	522.4
1019			1-linoleoylglycerophosphocholine	LC/MS pos	34419	C04100		0.97	0.7869	0.6900	1.1229	1.0878	56	91	Lipid	Lysolipid	11988421		5574	520.4
1020			2-linoleoylglycerophosphocholine*	LC/MS pos	35257			1.96	0.2603	0.4250	0.6255	1.2261	56	91	Lipid	Lysolipid			5544	520.4
1022			1-eicosadienoylglycerophosphocholine*	LC/MS pos	33871			0.51	0.2852	0.4400	1.1152	0.5716	56	45	Lipid	Lysolipid			5696	548.3
1026			2-arachidonoylglycerophosphocholine*	LC/MS pos	35256			1.42	0.4566	0.5412	0.7531	1.0676	56	82	Lipid	Lysolipid			5524	544.3
1035			1-stearoylglycerophosphoinositol	LC/MS neg	19324			0.69	0.4262	0.5258	1.4984	1.0276	100	100	Lipid	Lysolipid		796963-93-4;	5800	599.4
1036			2-stearoylglycerophosphoinositol*	LC/MS neg	39223			0.64	0.3121	0.4674	1.5505	0.9873	100	100	Lipid	Lysolipid			5751.6	599.4
1040			1-arachidonoylglycerophosphoinositol*	LC/MS neg	34214			1.13	0.2278	0.3968	0.9148	1.0373	100	100	Lipid	Lysolipid			5479	619.4
1043			1-oleoylglycerophosphoserine	LC/MS neg	19260			0.66	0.0472	0.1632	1.4452	0.9516	100	100	Lipid	Lysolipid	9547099		5690	522.3
1044			2-oleoylglycerophosphoserine*	LC/MS neg	37948			1.53	0.0643	0.1915	0.8970	1.3684	100	100	Lipid	Lysolipid			5577	522.3
1045			1-palmitoylplasmenylethanolamine*	LC/MS neg	39270			0.56	0.0550	0.1830	1.6075	0.8970	100	100	Lipid	Lysolipid			6153	436.4
1046			1-myristoylglycerol (1-monomyristin)	LC/MS neg	35625		HMDB11561	1.69	0.6863	0.6533	1.1485	1.9376	100	100	Lipid	Monoacylglycerol	79050	75685-84-6;	5965	227.3
1047			2-myristoylglycerol (2-monomyristin)	LC/MS neg	34383			1.45	0.6032	0.6177	1.1239	1.6351	100	100	Lipid	Monoacylglycerol	137938	27214-38-6;	5798	227.3
1049			1-pentadecanoylglycerol (1-monopentadecanoin)	GC/MS	34385			1.12	0.9772	0.7469	0.8640	0.9703	33	27	Lipid	Monoacylglycerol	190750		2085	357.3
1050			1-heptadecanoylglycerol (1-monoheptadecanoin)	GC/MS	34391			1.37	0.9078	0.7356	1.0898	1.4878	78	82	Lipid	Monoacylglycerol	107036	68002-72-2;	2156	385.2
1051			1-palmitoylglycerol (1-monopalmitin)	GC/MS	21127			1.57	0.8877	0.7286	1.5048	2.3604	100	100	Lipid	Monoacylglycerol	14900	542-44-9;	2119.5	371.3
1052			2-palmitoylglycerol (2-monopalmitin)	GC/MS	33419			1.65	0.5433	0.5946	1.0887	1.7928	100	100	Lipid	Monoacylglycerol	123409	23470-00-0;	2108	129
1053		Monoacylglycerol	1-stearoylglycerol (1-monostearin)	GC/MS	21188	D01947		0.92	0.5516	0.5984	1.2417	1.1424	100	100	Lipid	Monoacylglycerol	24699	123-94-4;31566-31-1;	2186.6	399.4
1054			2-stearoylglycerol (2-monostearin)	GC/MS	34059			1.30	0.5128	0.5812	0.4851	0.6321	22	36	Lipid	Monoacylglycerol	79075	621-61-4;	2178	341.3
1055			1-oleoylglycerol (1-monoolein)	GC/MS	21184		HMDB11567	1.43	0.8878	0.7286	1.1901	1.7008	100	100	Lipid	Monoacylglycerol	5283468	111-03-5;	2181.2	397.4
1057			1-linoleoylglycerol (1-monolinolein)	LC/MS neg	27447			0.82	0.9487	0.7469	1.5959	1.3088	100	100	Lipid	Monoacylglycerol	5283469	2277-28-3;	6226	279.2
1058			2-linoleoylglycerol (2-monolinolein)	LC/MS neg	32506		HMDB11538	0.87	0.8513	0.7194	1.7696	1.5332	100	100	Lipid	Monoacylglycerol	5365676	3443-82-1;	5988	279.3
1060			1-arachidonylglycerol	LC/MS neg	34397	C13857	HMDB11572	0.77	0.1305	0.2844	1.2548	0.9669	100	100	Lipid	Monoacylglycerol	5282281	35474-99-8;	5937	303.4
1061			2-arachidonoyl glycerol	LC/MS neg	19266	C13856	HMDB04666	0.86	0.4129	0.5176	1.1902	1.0245	100	100	Lipid	Monoacylglycerol	5282280	53847-30-6;	5955	303.3
1074			sphinganine	LC/MS pos	17769	C00836	HMDB00269	1.73	0.3379	0.4736	0.6089	1.0560	56	82	Lipid	Sphingolipid	3126	3102-56-5;	5175	302.3
1077			sphingosine	LC/MS pos	17747	C00319	HMDB00252	2.13	0.1757	0.3385	0.5529	1.1766	56	100	Lipid	Sphingolipid	5353955	123-78-4;	5197	300.2
1081		Sphingolipid	N,N-dimethylsphingosine	LC/MS pos	32460	C13914		1.91	0.1252	0.2787	0.7765	1.4817	56	91	Lipid	Sphingolipid	5282309	119567-63-4;	5192	328.3
1086			palmitoyl sphingomyelin	GC/MS	37506			0.93	0.3318	0.4720	1.0692	0.9980	100	100	Lipid	Sphingolipid	9939941	6254-89-3;	2524	311.3
1087			stearoyl sphingomyelin	GC/MS	19503	C00550	HMDB01348	0.76	0.0195	0.1000	1.1523	0.8799	100	100	Lipid	Sphingolipid	6453725	85187-10-6;85187-10-6 ;	2645	311.3
1100			lathosterol	GC/MS	39864	C01189	HMDB01170	1.18	0.2331	0.3999	0.9220	1.0906	78	100	Lipid	Sterol/Steroid	65728	80-99-9 ;	2337	255.2
1101			squalene	GC/MS	1518	C00751	HMDB00256	0.77	0.1914	0.3551	1.2377	0.9567	100	100	Lipid	Sterol/Steroid	638072	111-02-4;	2205	69
1102			cholesterol	GC/MS	63	C00187	HMDB00067	0.93	0.3077	0.4635	1.0581	0.9860	100	100	Lipid	Sterol/Steroid	6432564	57-88-5;	2316.9	329.3
1105		Sterol/Steroid	7-alpha-hydroxycholesterol	GC/MS	35692	C03594	HMDB01496	1.68	0.3385	0.4736	0.4410	0.7400	33	55	Lipid	Sterol/Steroid	107722	566-27-8;	2300	456.2
1106			7-beta-hydroxycholesterol	GC/MS	35092	C03594	HMDB06119	1.08	0.9656	0.7469	0.9338	1.0055	100	91	Lipid	Sterol/Steroid	473141	566-27-8;	2340	456.4
1139			beta-sitosterol	GC/MS	27414	C01753	HMDB00852	0.63	0.1984	0.3628	1.2694	0.7970	89	55	Lipid	Sterol/Steroid	222284	83-46-5;	2380.8	357.4
1141			campesterol	GC/MS	39511	C01789	HMDB02869	1.32	0.1850	0.3510	0.8808	1.1631	89	91	Lipid	Sterol/Steroid	173183	474-62-4;	2353	343.4
1157			7-alpha-hydroxy-3-oxo-4-cholestenoate (7-Hoca)	LC/MS neg	36776	C17337	HMDB12458	0.36	0.0883	0.2261	2.6542	0.9505	100	100	Lipid	Sterol/Steroid	3081085	115538-85-7;	5268	429.4
1187			xanthine	LC/MS pos	3147	C00385	HMDB00292	1.08	0.4939	0.5696	0.9291	1.0068	100	100	Nucleotide	Purine metabolism, (hypo)xanthine/inosine containing	1188	69-89-6;	1430	153.1
1188		Purine metabolism, (hypo)xanthine/inosine containing	xanthosine	LC/MS neg	15136	C01762	HMDB00299	0.95	0.5121	0.5812	1.0647	1.0129	100	100	Nucleotide	Purine metabolism, (hypo)xanthine/inosine containing	64959	146-80-5;	1075	283.1
1190			hypoxanthine	LC/MS neg	3127	C00262	HMDB00157	1.03	0.5792	0.6132	0.9869	1.0151	100	100	Nucleotide	Purine metabolism, (hypo)xanthine/inosine containing	790	68-94-0;	1313	135.1
1192			inosine	LC/MS neg	1123	C00294	HMDB00195	0.82	0.1061	0.2515	1.0703	0.8732	100	100	Nucleotide	Purine metabolism, (hypo)xanthine/inosine containing		58-63-9;	1600	267.2
1193			2'-deoxyinosine	LC/MS neg	15076	C05512	HMDB00071	0.44	0.0004	0.0110	1.8719	0.8174	100	100	Nucleotide	Purine metabolism, (hypo)xanthine/inosine containing	65058	890-38-0;	1700	251.1
1198			adenine	GC/MS	554	C00147	HMDB00034	1.75	0.0082	0.0617	0.7386	1.2943	100	100	Nucleotide	Purine metabolism, adenine containing	190	73-24-5;	1804.2	264
1199			adenosine	LC/MS pos	555	C00212	HMDB00050	1.29	0.0312	0.1311	0.9447	1.2224	100	100	Nucleotide	Purine metabolism, adenine containing	60961	58-61-7;	1650	268.1
1206		Purine metabolism, adenine containing	adenosine 2'-monophosphate (2'-AMP)	LC/MS neg	36815	C00946	HMDB11617	1.26	0.2870	0.4401	0.8998	1.1372	89	100	Nucleotide	Purine metabolism, adenine containing		130-49-4;	1497	346.1
1207			adenosine 3'-monophosphate (3'-AMP)	LC/MS pos	35142	C01367	HMDB03540	2.58	0.0204	0.1023	0.9910	2.5578	100	100	Nucleotide	Purine metabolism, adenine containing	15938966	84-21-9;	1451	348.1
1208			adenosine 5'-monophosphate (AMP)	LC/MS pos	32342	C00020	HMDB00045	0.88	0.8715	0.7267	1.4665	1.2899	100	100	Nucleotide	Purine metabolism, adenine containing	15938965	149022-20-8;	1210	348.1
1220			guanine	LC/MS pos	32352	C00242	HMDB00132	1.13	0.2641	0.4250	0.9831	1.1111	100	100	Nucleotide	Purine metabolism, guanine containing	764	73-40-5;	1022	152.1
1224		Purine metabolism, guanine containing	guanosine	LC/MS pos	1573	C00387	HMDB00133	0.99	0.9891	0.7479	1.0157	1.0020	100	100	Nucleotide	Purine metabolism, guanine containing	6802	118-00-3;	1676	284
1225	Nucleotide		2'-deoxyguanosine	LC/MS neg	1411	C00330	HMDB00085	0.37	0.0003	0.0079	2.2047	0.8241	100	91	Nucleotide	Purine metabolism, guanine containing	187790	961-07-9;	1759	266.2
1247		Purine metabolism, urate metabolism	urate	GC/MS	1604	C00366	HMDB00289	1.13	0.5971	0.6139	0.8435	0.9550	56	82	Nucleotide	Purine metabolism, urate metabolism		69-93-2;120K5305;	1928	441.2
1248			allantoin	GC/MS	1107	C02350	HMDB00462	1.17	0.4465	0.5366	1.0810	1.2691	100	100	Nucleotide	Purine metabolism, urate metabolism	204	97-59-6;	1809.8	518.3
1251			cytidine	LC/MS pos	514	C00475	HMDB00089	0.58	0.0000	0.0021	1.3186	0.7602	100	100	Nucleotide	Pyrimidine metabolism, cytidine containing	6175	65-46-3;	1065	244
1255		Pyrimidine metabolism, cytidine containing	cytidine 5'-monophosphate (5'-CMP)	LC/MS pos	2372	C00055	HMDB00095	0.99	0.8421	0.7139	1.0299	1.0247	100	100	Nucleotide	Pyrimidine metabolism, cytidine containing	7058165	63-37-6;	887	324
1260			2'-deoxycytidine	LC/MS pos	15949	C00881	HMDB00014	0.43	0.0006	0.0123	1.9730	0.8460	100	100	Nucleotide	Pyrimidine metabolism, cytidine containing	13711	951-77-9;	1256	228
1271		Pyrimidine metabolism, thymine containing	thymine	GC/MS	604	C00178	HMDB00262	1.16	0.4715	0.5513	0.9781	1.1345	78	100	Nucleotide	Pyrimidine metabolism, thymine containing	1135	65-71-4;	1429.1	255
1272			thymidine	LC/MS neg	2183	C00214	HMDB00273	0.57	0.0017	0.0219	1.5582	0.8899	100	100	Nucleotide	Pyrimidine metabolism, thymine containing	5789	50-89-5;	1935	241.1
1277			uracil	GC/MS	605	C00106	HMDB00300	1.27	0.0308	0.1311	0.9270	1.1799	100	100	Nucleotide	Pyrimidine metabolism, uracil containing	1174	66-22-8;	1370.4	241
1278			5,6-dihydrouracil	GC/MS	1559	C00429	HMDB00076	1.00	0.8063	0.6977	1.0187	1.0149	100	100	Nucleotide	Pyrimidine metabolism, uracil containing	649	504-07-4;	1505.2	243
1279		Pyrimidine metabolism, uracil containing	uridine	LC/MS neg	606	C00299	HMDB00296	1.02	0.9189	0.7375	0.9990	1.0181	100	100	Nucleotide	Pyrimidine metabolism, uracil containing	6029	58-96-8;	1430	243.1
1280			pseudouridine	LC/MS neg	33442	C02067	HMDB00767	1.30	0.0101	0.0697	0.8371	1.0857	100	100	Nucleotide	Pyrimidine metabolism, uracil containing		1445-07-4;	1104	243.1
1281			2'-deoxyuridine	LC/MS neg	1412	C00526	HMDB00012	0.82	0.3298	0.4720	1.1094	0.9115	100	91	Nucleotide	Pyrimidine metabolism, uracil containing	13712	951-78-0;	1608	227.1
1294		Purine and pyrimidine metabolism	methylphosphate	GC/MS	37070			1.22	0.0142	0.0844	0.8715	1.0661	100	100	Nucleotide	Purine and pyrimidine metabolism	13130	7023-27-0;	1221	240.9
1298		Ascorbate and aldarate metabolism	ascorbate (Vitamin C)	GC/MS	1640	C00072	HMDB00044	1.22	0.1185	0.2682	0.8628	1.0537	100	100	Cofactors and vitamins	Ascorbate and aldarate metabolism		134-03-2;	1850.1	332.1
1299			dehydroascorbate	GC/MS	1659	C05422	HMDB01264	0.88	0.2516	0.4184	0.8942	0.7872	67	36	Cofactors and vitamins	Ascorbate and aldarate metabolism	835	490-83-5;	1800	245.1
1313		Hemoglobin and porphyrin metabolism	heme	LC/MS neg	41754	C00032	HMDB03178	0.36	0.0008	0.0124	1.7661	0.6437	100	100	Cofactors and vitamins	Hemoglobin and porphyrin metabolism		14875-96-8;	5309.6	646.1
1314			L-urobilin	LC/MS pos	40173	C05793	HMDB04159	0.64	0.1752	0.3385	2.0936	1.3317	100	100	Cofactors and vitamins	Hemoglobin and porphyrin metabolism	5280818	34217-90-8;	4091	595.4
1324	Cofactors and vitamins	Nicotinate and nicotinamide metabolism	nicotinamide	LC/MS pos	594	C00153	HMDB01406	0.95	0.3469	0.4775	1.0290	0.9761	100	100	Cofactors and vitamins	Nicotinate and nicotinamide metabolism	936	98-92-0;	1267	123.1
1338			nicotinate	LC/MS pos	1504	C00253	HMDB01488	1.17	0.7730	0.6872	1.0816	1.2701	100	100	Cofactors and vitamins	Nicotinate and nicotinamide metabolism	938	59-67-6;	1241	124.1
1347		Pantothenate and CoA metabolism	pantothenate	LC/MS neg	1508	C00864	HMDB00210	1.66	0.0000	0.0029	0.7809	1.2960	100	100	Cofactors and vitamins	Pantothenate and CoA metabolism	6613	137-08-6;	1486	218.2
1365		Riboflavin metabolism	flavin adenine dinucleotide (FAD)	LC/MS neg	2134	C00016	HMDB01248	1.44	0.3464	0.4775	0.5174	0.7455	56	73	Cofactors and vitamins	Riboflavin metabolism	643975	146-14-5;84366-81-4;	2413	784.1
1372		Tocopherol metabolism	alpha-tocopherol	GC/MS	1561	C02477	HMDB01893	0.98	0.5843	0.6132	0.9948	0.9774	100	91	Cofactors and vitamins	Tocopherol metabolism	14985	59-02-9;10191-41-0;	2305.4	502.5
1389		Vitamin B6 metabolism	pyridoxate	LC/MS neg	31555	C00847	HMDB00017	0.27	0.0083	0.0617	2.0938	0.5731	100	100	Cofactors and vitamins	Vitamin B6 metabolism	6723	82-82-6 ;	2210	182.1
1395			hippurate	LC/MS neg	15753	C01586	HMDB00714	1.02	0.9463	0.7469	0.9104	0.9305	100	100	Xenobiotics	Benzoate metabolism	464	495-69-2;	2136	178.1
1407		Benzoate metabolism	catechol sulfate	LC/MS neg	35320	C00090		1.31	0.1329	0.2860	0.8102	1.0619	67	100	Xenobiotics	Benzoate metabolism	3083879	120-80-9;	1928	188.9
1408			benzoate	GC/MS	15778	C00180	HMDB01870	0.65	0.0188	0.1000	1.3747	0.8961	100	100	Xenobiotics	Benzoate metabolism	243	65-85-0;	1291.5	179
1436		Chemical	glycerol 2-phosphate	GC/MS	27728	C02979	HMDB02520	1.52	0.1010	0.2417	0.9292	1.4170	100	100	Xenobiotics	Chemical	2526	819-83-0;	1691.8	243
1518.93			2,8-quinolinediol	LC/MS pos	42975	C06342	C06342	0.29	0.0057	0.0503	2.2972	0.6596	100	100	Xenobiotics	Chemical	97250	15450-76-7;	3305	162.2
1583		Drug	pentobarbital	LC/MS neg	38156			0.80	0.5365	0.5940	1.4383	1.1469	100	100	Xenobiotics	Drug	4737	76-74-4;	4564	225.2
1683			enterolactone	LC/MS neg	39626			0.56	0.0240	0.1160	1.4150	0.7899	100	100	Xenobiotics	Food component/Plant	10685477	78473-71-9;	4492.7	297.2
1715	Xenobiotics		equol	LC/MS neg	36774	C14131	HMDB02209	0.95	0.7829	0.6888	1.2455	1.1843	100	100	Xenobiotics	Food component/Plant	382975	94105-90-5;	4502	241.1
1729			ergothioneine	LC/MS pos	37459	C05570	HMDB03045	0.81	0.1606	0.3263	1.1974	0.9742	100	100	Xenobiotics	Food component/Plant	3032311	58511-63-0;	847	230.2
1732			N-glycolylneuraminate	GC/MS	37123	C03410	HMDB00833	1.55	0.0454	0.1591	0.9031	1.4010	100	100	Xenobiotics	Food component/Plant	4603312	1113-83-3;	2119	444.2
1753		Food component/Plant	stachydrine	LC/MS pos	34384	C10172	HMDB04827	1.79	0.0370	0.1458	0.9030	1.6193	100	100	Xenobiotics	Food component/Plant	115244	4136-37-2;	860	144.1
1754			homostachydrine*	LC/MS pos	33009	C08283		1.76	0.0651	0.1915	0.9206	1.6157	100	100	Xenobiotics	Food component/Plant	441447	1195-94-4;	1199	158.2
1759			sitostanol	GC/MS	37524	C19644		0.83	0.4144	0.5176	1.0086	0.8399	100	73	Xenobiotics	Food component/Plant		19466-47-8;	2388	215.2
1760			2-oxindole-3-acetate	LC/MS pos	40479			0.47	0.7569	0.6834	2.0702	0.9634	67	91	Xenobiotics	Food component/Plant	3080590	2971-31-5;	3217.2	192.1
1788			1H-quinolin-2-one	LC/MS pos	40464	C06415		0.38	0.0921	0.2277	1.9367	0.7281	100	91	Xenobiotics	Food component/Plant	6038	59-31-4;	3533	146.2
