# Supplementary material for: Molecular and metabolomic changes in the proximal colon of pigs infected with Trichuris suis
Source: Sci Rep. 2020 Jul 30;10:12853. doi: 10.1038/s41598-020-69462-5 (PMC7393168; doi:10.1038/s41598-020-69462-5)
Supplement: Supplementary file 12 — Supplementary Table S12. [file 41598_2020_69462_MOESM12_ESM.pdf]

Molecular and metabolomic changes in the proximal colon of pigs infected with *Trichuris suis*

Harry Dawson[1], Celine Chen[1], Robert Li[2], Lauren Nicki Bell[3], Terez Shea-Donohue[4], Helene Kringel[5], Ethiopia Beshah[1], Dolores E. Hill[2], Joseph F. Urban Jr[1,2].

[1]United States Department of Agriculture, Agricultural Research Service, Northeast Area, Beltsville Human Nutrition Research Center, Diet Genomics and Immunology Laboratory, [2] Beltsville Agricultural Research Center, Animal Parasitology Disease Laboratory, Beltsville, MD; [3]Metabolon, Inc., Morrisville, NC; [4]University of Maryland School of Medicine, Baltimore, MD, USA; [5]Department of Veterinary Disease Biology, Faculty of Health and Medical Sciences, University of Copenhagen, Copenhagen, Denmark

Supplemental Table S12

Supplemental Table S12. Metabolic markers of *Trichuris suis* infection colon contents 33 dpi

Heat map of statistically significant biochemicals profiled in this study.

Comparison mean values significantly different:
- Red: $p \leq 0.05$, fold of change ≥ 1.00
- Green: $p \leq 0.05$, fold of change < 1.00

Comparison mean values approaching significance:
- Pink: $0.05 < p < 0.10$, fold of change ≥ 1.00
- Light green: $0.05 < p < 0.10$, fold of change < 1.00

Pathway Sort Order	Super Pathway	Sub Pathway	Biochemical Name	Platform	Comp ID	KEGG	HMDB	Fold of Change: Welch's Two-Sample t-Test, Infected / Non-Infected	Statistical Values: Welch's Two-Sample t-Test, Infected / Non-Infected: *p*-Value	*q*-Value	Mean Values: Non-Infected	Mean Values: Infected	% Filled Values: Non-Infected	% Filled Values: Infected	Super Pathway	Sub Pathway	PubChem	CAS	RI	Mass
1	Amino acid	Glycine, serine and threonine metabolism	glycine	GC/MS	11777	C00037	HMDB00123	3.26	0.0206	0.0525	0.8226	2.6841	100	100	Amino acid	Glycine, serine and threonine metabolism	5257127;750	56-40-6;	1166	101.9
2			sarcosine (N-Methylglycine)	GC/MS	1516	C00213	HMDB00271	2.65	0.0236	0.0583	0.3690	0.9774	22	55	Amino acid	Glycine, serine and threonine metabolism	1088;7311726	107-97-1;	1182.9	116
4			N-acetylglycine	LC/MS neg	27710		HMDB00532	2.62	0.0037	0.0154	0.8623	2.2635	100	100	Amino acid	Glycine, serine and threonine metabolism	10972	543-24-8;	735	116.1
8			serine	GC/MS	1648	C00065	HMDB00187	1.59	0.1291	0.1886	0.9149	1.4554	100	100	Amino acid	Glycine, serine and threonine metabolism	5951;6857581	56-45-1;	1389.1	204
12			homoserine	GC/MS	23642	C00263	HMDB00719	0.98	0.8015	0.5636	0.9961	0.9806	100	100	Amino acid	Glycine, serine and threonine metabolism	12647;6971022	672-15-1;	1471	218.1
17			threonine	LC/MS pos	1284	C00188	HMDB00167	0.98	0.7717	0.5517	0.9754	0.9551	100	100	Amino acid	Glycine, serine and threonine metabolism	6971019;6288	72-19-5;	713	120.1
18			N-acetylthreonine	LC/MS neg	33939	C01118		0.88	0.7487	0.5517	0.9108	0.8056	22	27	Amino acid	Glycine, serine and threonine metabolism	4651717	17093-74-2;	846	160.1
24		Alanine and aspartate metabolism	aspartate	GC/MS	15996	C00049	HMDB00191	0.95	0.9527	0.6084	1.0473	0.9982	100	100	Amino acid	Alanine and aspartate metabolism	5960	56-84-8;	1529.7	232
28			beta-alanine	GC/MS	55	C00099	HMDB00056	0.84	0.2888	0.3249	1.2327	1.0316	100	100	Amino acid	Alanine and aspartate metabolism	239;4755801	56-41-7;107-95-9;	1451.8	174
32			alanine	GC/MS	1126	C00041	HMDB00161	1.54	0.2883	0.3249	1.0721	1.6522	100	100	Amino acid	Alanine and aspartate metabolism	5950;7311724	56-41-7;	1147.6	115.9
35			N-acetylaspartate (NAA)	LC/MS pos	22185	C01042	HMDB00812	1.01	0.6056	0.4919	1.0844	1.0907	100	100	Amino acid	Alanine and aspartate metabolism	65065	997-55-7;997-55-7 ;	1222	176.1
38			2,6-pyridinedicarboxylate	GC/MS	15135			0.85	0.6821	0.5412	1.3287	1.1319	100	100	Amino acid	Alanine and aspartate metabolism	10367	499-83-2;	1721.3	296.1
39		Glutamate metabolism	glutamate	LC/MS pos	57	C00025	HMDB03339	0.68	0.0016	0.0081	1.2226	0.8305	100	100	Amino acid	Glutamate metabolism	611	56-86-0;	700	148.1
45			glutamine	LC/MS pos	53	C00064	HMDB00641	0.96	0.7529	0.5517	0.9821	0.9477	89	100	Amino acid	Glutamate metabolism	5961	56-85-9;	684	147.2
47			gamma-aminobutyrate (GABA)	GC/MS	1416	C00334	HMDB00112	1.20	0.6896	0.5412	1.0277	1.2383	100	100	Amino acid	Glutamate metabolism	6992099;119	56-12-2;	1539.7	304.1
48			carboxyethyl-GABA	LC/MS pos	40007		HMDB02201	0.64	0.8233	0.5696	1.3787	0.8867	67	91	Amino acid	Glutamate metabolism	2572	4386-03-2;	810	176.2
50			N-acetylglutamate	LC/MS pos	15720	C00624	HMDB01138	0.87	0.4558	0.4169	1.3620	1.1784	100	100	Amino acid	Glutamate metabolism	1549099	5817-08-3;	1492	190.1
53		Histidine metabolism	histidine	GC/MS	37424	C00135	HMDB00177	1.54	0.2873	0.3249	0.9744	1.4964	89	91	Amino acid	Histidine metabolism	773;3651426	5934-29-2;	1837.3	254.1
54			N-acetylhistidine	LC/MS neg	33946	C02997		1.28	0.1946	0.2511	1.1530	1.4717	100	100	Amino acid	Histidine metabolism	75619	39145-52-3;	913	196.2
55			trans-urocanate	LC/MS pos	607	C00785	HMDB00301	1.00	0.6881	0.5412	1.0356	1.0354	100	100	Amino acid	Histidine metabolism	736715	104-98-3;	1119	139.1
58			histamine	GC/MS	1574	C00388	HMDB00870	---	---	---	1.0000	1.0000	0	9	Amino acid	Histidine metabolism	774	51-45-6;	1808	174.1
64			1-methylimidazoleacetate	LC/MS pos	32350	C05828	HMDB02820	0.49	0.0147	0.0406	1.5127	0.7351	100	91	Amino acid	Histidine metabolism	75810	2625-49-2 ;	845	141.1
65			imidazole propionate	LC/MS pos	40730		HMDB02271	3.38	0.1077	0.1667	0.7791	2.6334	100	100	Amino acid	Histidine metabolism	70630	1074-59-5;	1011.7	141.1
68		Lysine metabolism	cadaverine	GC/MS	15308	C01672	HMDB02322	6.73	0.0574	0.1120	0.8773	5.9012	89	100	Amino acid	Lysine metabolism		462-94-2;1476-39-7;	1780.4	174
69			glutarate (pentanedioate)	GC/MS	396	C00489	HMDB00661	0.73	0.3336	0.3621	2.0927	1.5309	100	100	Amino acid	Lysine metabolism	4418048	110-94-1;	1433.6	261
74			diaminopimelate	GC/MS	15130	C00666	HMDB01370	2.53	0.0000	0.0008	0.6764	1.7115	100	100	Amino acid	Lysine metabolism	1550876;1550875	1476-39-7;2577-62-0;	1874.4	272.1
75			lysine	GC/MS	1301	C00047	HMDB00182	1.34	0.4623	0.4179	0.8535	1.1454	100	100	Amino acid	Lysine metabolism	5962	56-87-1;	1836.7	317.2
80			pipecolate	LC/MS pos	1444	C00408	HMDB00070	1.00	0.8588	0.5833	1.6267	1.6314	100	100	Amino acid	Lysine metabolism	849	4043-87-2;	1120	130.1
84			N2-acetyllysine	LC/MS pos	36751	C12989	HMDB00446	0.77	0.0980	0.1567	1.1892	0.9145	100	100	Amino acid	Lysine metabolism	6992697;92907	1946-82-3;	853	189.1
85			N6-acetyllysine	LC/MS pos	36752	C02727	HMDB00206	1.51	0.0684	0.1281	0.9531	1.4361	100	100	Amino acid	Lysine metabolism	6991978;92832	692-04-6;	1134	189.1
87			2-aminopentanoate	GC/MS	37426	C01826		2.29	0.4221	0.4007	1.0809	2.4804	78	100	Amino acid	Lysine metabolism	5255804;824	760-78-1;	1279	144.1
88		Phenylalanine & tyrosine metabolism	phenyllactate (PLA)	LC/MS neg	22130	C05607	HMDB00779	4.37	0.1743	0.2287	1.0717	4.6866	89	100	Amino acid	Phenylalanine & tyrosine metabolism	3848	828-01-3;	2237	165.1
89			phenylalanine	LC/MS pos	64	C00079	HMDB00159	1.26	0.3359	0.3629	0.9299	1.1720	100	100	Amino acid	Phenylalanine & tyrosine metabolism	6925665;6140	63-91-2;	2056	166.1
92			phenylacetate	LC/MS neg	15958	C07086	HMDB00209	0.62	0.0123	0.0365	1.1906	0.7390	100	100	Amino acid	Phenylalanine & tyrosine metabolism		103-82-2;	2127	135.1
94			p-cresol sulfate	LC/MS neg	36103	C01468	HMDB11635	3.57	0.0084	0.0294	0.9393	3.3520	100	100	Amino acid	Phenylalanine & tyrosine metabolism	4615422	3233-57-7;	2896	187.1
106			tyrosine	LC/MS pos	1299	C00082	HMDB00158	1.24	0.3725	0.3811	0.9451	1.1719	100	100	Amino acid	Phenylalanine & tyrosine metabolism	6057;6942100	60-18-4;	1516	182.1
107			3-(4-hydroxyphenyl)lactate	LC/MS neg	32197	C03672	HMDB00755	3.81	0.1978	0.2522	0.9775	3.7241	100	100	Amino acid	Phenylalanine & tyrosine metabolism	9378	6482-98-0;	1395	181.1
110			tyramine	LC/MS pos	1603	C00483	HMDB00306	2.26	0.7629	0.5517	1.5933	3.5977	100	100	Amino acid	Phenylalanine & tyrosine metabolism	5610	60-19-5;	1503	138.1
124			4-hydroxyphenylacetate	GC/MS	541	C00642	HMDB00020	3.06	0.0651	0.1239	0.9597	2.9359	100	100	Amino acid	Phenylalanine & tyrosine metabolism	4693933	156-38-7;	1630.6	179
125			3-hydroxyphenylacetate	LC/MS neg	1413	C05593	HMDB00440	0.41	0.0004	0.0037	1.3215	0.5415	100	91	Amino acid	Phenylalanine & tyrosine metabolism	6950813	621-37-4;	1604	151.1
131			N-acetylphenylalanine	LC/MS neg	33950	C03519	HMDB00512	1.75	0.1960	0.2515	0.9902	1.7300	100	91	Amino acid	Phenylalanine & tyrosine metabolism	74839	2018-61-3;	2590	206.2
137			2-(4-hydroxyphenyl)propionate	LC/MS neg	35632	C03080		3.00	0.0010	0.0060	0.4202	1.2590	44	91	Amino acid	Phenylalanine & tyrosine metabolism	102526	938-96-5;	1880	165.1
138			3-(3-hydroxyphenyl)propionate	LC/MS neg	35635	C11457	HMDB00375	2.17	0.0777	0.1368	1.0816	2.3441	100	100	Amino acid	Phenylalanine & tyrosine metabolism	91	621-54-5;	2000	165.1
139			3-(4-hydroxyphenyl)propionate	LC/MS neg	39587	C01744	HMDB02199	2.30	0.1017	0.1607	0.9766	2.2444	89	100	Amino acid	Phenylalanine & tyrosine metabolism	10394	501-97-3;	1658	165.1
140			3-phenylpropionate (hydrocinnamate)	LC/MS neg	15749	C05629	HMDB00764	0.76	0.0530	0.1062	1.1697	0.8918	100	100	Amino acid	Phenylalanine & tyrosine metabolism		501-52-0;	2830	149.1
143			4-hydroxycinnamate	LC/MS neg	36770	C00811	HMDB02035	0.71	0.1175	0.1796	1.3325	0.9427	100	100	Amino acid	Phenylalanine & tyrosine metabolism	637542	501-98-4;	1474	163
151		Tryptophan metabolism	kynurenate	LC/MS neg	1417	C01717	HMDB00715	1.46	0.2704	0.3125	0.8028	1.1720	78	100	Amino acid	Tryptophan metabolism	3845	492-27-3;	2243	188.1
153			tryptophan	LC/MS pos	54	C00078	HMDB00929	1.91	0.0283	0.0684	0.8759	1.6697	100	100	Amino acid	Tryptophan metabolism	6923516;6305	73-22-3;	2445	205.1
156			indoleacetate	LC/MS pos	27513	C00954	HMDB00197	6.05	0.0017	0.0082	0.7782	4.7066	100	100	Amino acid	Tryptophan metabolism	802	6505-45-9;	3760	176.1
158			indole-3-carboxylic acid	LC/MS neg	38116	C19837	HMDB03320	0.66	0.0754	0.1357	1.1320	0.7520	100	64	Amino acid	Tryptophan metabolism		771-50-6;	1713	160.1
167			tryptamine	LC/MS pos	6104	C00398	HMDB00303	0.50	0.0018	0.0087	1.4213	0.7121	100	100	Amino acid	Tryptophan metabolism	1150	61-54-1;	2323	161.1
169			serotonin (5HT)	LC/MS pos	2342	C00780	HMDB00259	0.92	0.7700	0.5517	0.9715	0.8895	89	100	Amino acid	Tryptophan metabolism	5202	153-98-0;	1660	177.1
180			indolepropionate	GC/MS	32405		HMDB02302	0.61	0.0201	0.0523	1.3605	0.8264	100	100	Amino acid	Tryptophan metabolism	3744	830-96-6 ;	1925	202.1
183		Valine, leucine and isoleucine metabolism	3-methyl-2-oxovalerate	LC/MS neg	15676	C00671	HMDB03736	1.17	0.7200	0.5442	0.7169	0.8388	44	36	Amino acid	Valine, leucine and isoleucine metabolism	47	51829-07-3;	2106	129.2
189			alpha-hydroxyisocaproate	LC/MS neg	22132	C03264	HMDB00746	5.89	0.2932	0.3251	1.1202	6.5964	100	91	Amino acid	Valine, leucine and isoleucine metabolism	83697	10303-64-7;	1854	131.2
190			isoleucine	LC/MS pos	1125	C00407	HMDB00172	1.33	0.3854	0.3859	0.9674	1.2852	100	100	Amino acid	Valine, leucine and isoleucine metabolism	791	73-32-5;	1614	132.1
191			leucine	LC/MS pos	60	C00123	HMDB00687	1.40	0.2272	0.2763	0.8858	1.2372	100	100	Amino acid	Valine, leucine and isoleucine metabolism	7045798;6106	61-90-5;	1674	132.2
194			methylsuccinate	GC/MS	15745		HMDB01844	0.53	0.0288	0.0684	1.3305	0.7089	89	91	Amino acid	Valine, leucine and isoleucine metabolism	10349	498-21-5;	1362.1	261
195			N-acetylleucine	LC/MS neg	1587	C02710	HMDB11756	1.43	0.6827	0.5412	1.0808	1.5485	78	91	Amino acid	Valine, leucine and isoleucine metabolism	70912	1188-21-2;	2385	172.1
197			N-acetylisoleucine	LC/MS neg	33967			1.51	0.3441	0.3650	1.0298	1.5530	89	100	Amino acid	Valine, leucine and isoleucine metabolism	2802421	3077-46-1;	2279	172.2
200			valine	LC/MS pos	1649	C00183	HMDB00883	1.46	0.2276	0.2763	0.9242	1.3464	100	100	Amino acid	Valine, leucine and isoleucine metabolism	6971018;6287	72-18-4;	1040	118.1
203			4-methyl-2-oxopentanoate	GC/MS	15683	C00233	HMDB00695	0.39	0.0087	0.0298	2.2565	0.8821	100	100	Amino acid	Valine, leucine and isoleucine metabolism	70	816-66-0;	1283.9	259.1
208			alpha-hydroxyisovalerate	GC/MS	33937		HMDB00407	3.20	0.4559	0.4169	1.1970	3.8256	100	91	Amino acid	Valine, leucine and isoleucine metabolism	99823	600-37-3;	1208	145.1
211			2-hydroxy-3-methylvalerate	LC/MS neg	36746		HMDB00317	3.21	0.7202	0.5442	1.2911	4.1451	78	82	Amino acid	Valine, leucine and isoleucine metabolism	164623	488-15-3;	1787	131.1
224		Cysteine, methionine, SAM, taurine metabolism	cysteine	GC/MS	31453	C00097	HMDB00574	1.14	0.8240	0.5696	1.0374	1.1805	100	100	Amino acid	Cysteine, methionine, SAM, taurine metabolism	5862;6419722	52-90-4;56-89-3;	1560.1	218
230			methionine sulfoxide	LC/MS pos	18374	C02989	HMDB02005	0.91	0.2939	0.3251	1.0526	0.9618	100	100	Amino acid	Cysteine, methionine, SAM, taurine metabolism	158980;1548907	3226-65-1;	729	166.1
231			N-formylmethionine	LC/MS pos	2829	C03145	HMDB01015	1.29	0.1464	0.2060	0.9051	1.1635	100	91	Amino acid	Cysteine, methionine, SAM, taurine metabolism	439750	4289-98-9;	1541	176.1
237			methionine	LC/MS pos	1302	C00073	HMDB00696	1.17	0.6326	0.5102	0.9335	1.0914	100	100	Amino acid	Cysteine, methionine, SAM, taurine metabolism	6992087;6137	63-68-3;	1252	150.1
239			N-acetylmethionine	LC/MS neg	1589	C02712	HMDB11745	1.64	0.1645	0.2239	0.8070	1.3240	100	100	Amino acid	Cysteine, methionine, SAM, taurine metabolism	448580	65-82-7;	1805	190.1
241			2-hydroxybutyrate (AHB)	GC/MS	21044	C05984	HMDB00008	3.45	0.1198	0.1807	0.8056	2.7816	56	82	Amino acid	Cysteine, methionine, SAM, taurine metabolism	440864	3347-90-8;	1169.4	130.9
255		Urea cycle; arginine-, proline-, metabolism	arginine	GC/MS	1638	C00062	HMDB00517	1.32	0.7772	0.5521	1.0762	1.4245	100	100	Amino acid	Urea cycle; arginine-, proline-, metabolism	5246487;232	1119-34-2;	1608.5	142
258			ornithine	GC/MS	1493	C00077	HMDB03374	2.67	0.1658	0.2239	0.8639	2.3039	100	100	Amino acid	Urea cycle; arginine-, proline-, metabolism	6262	3184-13-2;	1763.8	141.9
261			proline	LC/MS pos	1898	C00148	HMDB00162	1.20	0.4288	0.4016	0.9320	1.1166	100	100	Amino acid	Urea cycle; arginine-, proline-, metabolism	145742;6971047	147-85-3;	796	116.1
262			5-aminovalerate	GC/MS	18319	C00431	HMDB03355	5.24	0.0564	0.1111	0.9188	4.8175	100	100	Amino acid	Urea cycle; arginine-, proline-, metabolism	6992101;138	660-88-8;	1620.8	174
263			citrulline	LC/MS pos	2132	C00327	HMDB00904	0.77	0.0115	0.0347	1.1565	0.8895	100	100	Amino acid	Urea cycle; arginine-, proline-, metabolism	833	372-75-8;	715	176.1
264			N-acetylornithine	LC/MS pos	15630	C00437	HMDB03357	1.44	0.4652	0.4189	0.9454	1.3644	100	100	Amino acid	Urea cycle; arginine-, proline-, metabolism	6992102;439232	6205-08-9;	875	175.2
266			N-methyl proline	LC/MS pos	37431			3.62	0.0286	0.0684	0.6793	2.4615	67	100	Amino acid	Urea cycle; arginine-, proline-, metabolism	557	475-11-6;	833	130.1
267			trans-4-hydroxyproline	LC/MS pos	32306	C01157	HMDB00725	0.82	0.0979	0.1567	1.2242	1.0014	100	100	Amino acid	Urea cycle; arginine-, proline-, metabolism	5810;6971053	51-35-4;	705	132.1
274		Creatine metabolism	creatine	LC/MS pos	27718	C00300	HMDB00064	1.51	0.7594	0.5517	1.0560	1.5971	100	100	Amino acid	Creatine metabolism	586	57-00-1;	758	132.1
278		Butanoate metabolism	2-aminobutyrate	GC/MS	1577	C02261	HMDB00650	1.94	0.7801	0.5525	1.0746	2.0890	100	100	Amino acid	Butanoate metabolism	439691;6971251	1492-24-6;	1215.7	130
282		Polyamine metabolism	putrescine	GC/MS	1408	C00134	HMDB01414	2.30	0.4125	0.3992	0.9318	2.1469	100	100	Amino acid	Polyamine metabolism		110-60-1;	1705.8	174
283			N-acetylputrescine	LC/MS pos	37496	C02714	HMDB02064	1.61	0.1704	0.2283	0.9936	1.5967	100	100	Amino acid	Polyamine metabolism	122356	18233-70-0;	895	131.1
284			agmatine	GC/MS	15496	C00179	HMDB01432	5.56	0.2794	0.3213	1.0739	5.9760	100	82	Amino acid	Polyamine metabolism	199	2482-00-0;	1526	174
290		Guanidino and acetamido metabolism	4-guanidinobutanoate	LC/MS pos	15681	C01035	HMDB03464	0.57	0.0026	0.0116	1.6173	0.9160	100	91	Amino acid	Guanidino and acetamido metabolism	500	463-003;463-00-3;	1085	146.1
291			4-acetamidobutanoate	LC/MS pos	1558	C02946	HMDB03681	0.78	0.3534	0.3714	1.0431	0.8156	56	73	Amino acid	Guanidino and acetamido metabolism	18189	3025-96-5;	1724	146.1
297		Glutathione metabolism	5-oxoproline	LC/MS pos	1494	C01879	HMDB00267	0.82	0.1257	0.1860	1.2605	1.0359	100	100	Amino acid	Glutathione metabolism	7405	98-79-3;	1446	130.1
311	Peptide	Dipeptide	glycylvaline	LC/MS pos	18357			1.10	0.7523	0.5517	0.9983	1.0933	100	100	Peptide	Dipeptide	2724807;6994979	1963-21-9;	1572	175.1
314			glycylproline	LC/MS pos	22171		HMDB00721	1.55	0.0669	0.1263	0.9474	1.4730	100	100	Peptide	Dipeptide	3013625;6993386	704-15-4;	1115	173.1
315			glycylisoleucine	LC/MS pos	36659			1.17	0.3763	0.3818	0.9294	1.0920	100	100	Peptide	Dipeptide		19461-38-2;	2080	189.1
316			glycylleucine	LC/MS pos	34398	C02155	HMDB00759	1.15	0.6035	0.4919	1.0387	1.1969	100	100	Peptide	Dipeptide	92843;1548899	869-19-2;	2236	189.1
320			glycyltyrosine	LC/MS pos	33958			0.93	0.7719	0.5517	1.0483	0.9757	100	100	Peptide	Dipeptide	92829;6994980	658-79-7;	1728	239.1
324			alanylvaline	LC/MS pos	37084			0.95	0.4439	0.4141	1.0688	1.0158	100	100	Peptide	Dipeptide	137276	3303-45-5;	1513	189
325			alanylproline	LC/MS pos	37083			1.29	0.2891	0.3249	0.9153	1.1833	100	100	Peptide	Dipeptide	418040	13485-59-1;	1400	187.1
328			alanylleucine	LC/MS pos	37093			1.28	0.7297	0.5468	1.0573	1.3487	100	100	Peptide	Dipeptide	259583	1638-60-4;	2150	203
332			alanylglutamate	LC/MS neg	37064			0.92	0.3907	0.3895	1.0414	0.9544	100	100	Peptide	Dipeptide	656476		637	217
334			alanylisoleucine	LC/MS pos	37118			1.09	0.8142	0.5696	1.1205	1.2202	100	100	Peptide	Dipeptide	417358;5246008		2011	203.1
337.08			asparagylisoleucine	LC/MS pos	42976			0.83	0.3426	0.3650	1.0753	0.8925	100	100	Peptide	Dipeptide			2070	246.2
337.09			asparagylleucine	LC/MS pos	42980			0.86	0.4268	0.4014	1.1425	0.9820	100	100	Peptide	Dipeptide			2170	246.2
339.1			arginylproline	LC/MS pos	41394			1.24	0.5610	0.4688	1.0263	1.2773	100	100	Peptide	Dipeptide	332964	2418-69-1;	785.8	272.2
339.4			arginylvaline	LC/MS pos	41395			1.76	0.0011	0.0066	0.6913	1.2173	89	100	Peptide	Dipeptide	335257	2896-20-0;	944.9	274.2
340			aspartylphenylalanine	LC/MS pos	22175		HMDB00706	1.20	0.3633	0.3750	0.9258	1.1117	89	100	Peptide	Dipeptide	93078	13433-09-5;	2538	281.1
343.3			glutamine-leucine	LC/MS pos	42994			0.98	0.7339	0.5482	1.0780	1.0533	100	100	Peptide	Dipeptide			2180	260.2
343.4			glutamine-isoleucine	LC/MS pos	42988			1.00	0.9325	0.6080	1.0554	1.0562	100	100	Peptide	Dipeptide			2070.6	260.2
358.1			isoleucylthreonine	LC/MS pos	42968			1.30	0.0723	0.1325	0.8173	1.0592	100	100	Peptide	Dipeptide			1555.1	233.2
360			leucylleucine	LC/MS pos	36756	C11332		1.40	0.1785	0.2316	1.0809	1.5096	100	100	Peptide	Dipeptide	76807;6992072	3303-31-9;	3012	245.1
361			leucylisoleucine	LC/MS pos	36757			1.85	0.0753	0.1357	1.0688	1.9807	100	91	Peptide	Dipeptide		36077-41-5;	2800	245.1
361.5			leucylthreonine	LC/MS pos	42969			1.25	0.0693	0.1288	0.9508	1.1884	100	100	Peptide	Dipeptide			1621.9	233.2
363.85			threonylisoleucine	LC/MS pos	42972			1.01	0.9598	0.6084	1.0430	1.0567	100	100	Peptide	Dipeptide			2135	233.2
363.9			threonylvaline	LC/MS pos	42011			1.29	0.1173	0.1796	0.8767	1.1280	100	100	Peptide	Dipeptide	416721;4278432	99032-17-4;	1574.5	219.2
364			threonylphenylalanine	LC/MS pos	31530			1.27	0.2553	0.2995	0.8912	1.1319	100	100	Peptide	Dipeptide	4099799;4099798	16875-27-7;	2500	267.2
376.1			arginylphenylalanine	LC/MS pos	41485			1.34	0.0774	0.1368	0.8595	1.1527	78	100	Peptide	Dipeptide	4441256	2047-13-4;	1795	322.2
379			cyclo(leu-phe)	LC/MS pos	37101			1.05	0.8539	0.5833	0.8354	0.8779	100	73	Peptide	Dipeptide	7076347		4427	261.1
386			valylleucine	LC/MS pos	39994			1.82	0.1276	0.1875	1.0572	1.9211	100	100	Peptide	Dipeptide	352039	22906-55-4;	2503.3	231.2
387			cyclo(phe-phe)	LC/MS pos	38152			1.42	0.0934	0.1535	0.5535	0.7869	33	73	Peptide	Dipeptide		2862-51-3;	4527	295.2
388			arginylisoleucine	LC/MS pos	40070			1.05	0.7226	0.5442	0.8860	0.9347	89	91	Peptide	Dipeptide	433625	62632-70-6;	1554	288.3
389			arginylleucine	LC/MS pos	39995			1.27	0.0724	0.1325	0.8784	1.1180	100	100	Peptide	Dipeptide	333445	1188-24-5;	1620	288.3
390			aspartylleucine	LC/MS pos	40068			1.88	0.0102	0.0322	0.7449	1.4016	100	100	Peptide	Dipeptide	332962	3062-14-4;	2310	247.2
402			isoleucylalanine	LC/MS pos	40046			1.18	0.3492	0.3687	1.0315	1.2122	100	100	Peptide	Dipeptide	5246009;5246010	24787-73-3;	1686	203.2
404			isoleucylglutamate	LC/MS pos	40057			0.95	0.9312	0.6080	1.1273	1.0724	100	100	Peptide	Dipeptide		42516-53-0;	1650	261.2
406			isoleucylglycine	LC/MS pos	40008			1.24	0.1335	0.1914	0.9636	1.1913	100	100	Peptide	Dipeptide	342532	868-28-0;	1732	189.2
412			isoleucylvaline	LC/MS pos	40049			1.31	0.0843	0.1430	1.0240	1.3444	100	100	Peptide	Dipeptide	5246011;435949	41017-96-3;	2202	231.2
413			leucylalanine	LC/MS pos	40010			1.09	0.8237	0.5696	0.9720	1.0642	100	100	Peptide	Dipeptide	259321	7298-84-2;	1760	203.2
417			leucylglutamate	LC/MS pos	40021			1.08	0.5579	0.4678	0.9855	1.0643	100	100	Peptide	Dipeptide	5259589;5259590		1716	261.2
418			leucylglycine	LC/MS pos	40045			1.30	0.1390	0.1969	0.9352	1.2166	100	100	Peptide	Dipeptide	79070	686-50-0;	1800	189.2
426			lysylleucine	LC/MS pos	40020			1.35	0.1074	0.1667	0.9867	1.3306	100	100	Peptide	Dipeptide	4682588	7369-79-1;	1546	260.2
427			lysylproline	LC/MS pos	40666			2.18	0.0078	0.0286	0.7838	1.7067	100	100	Peptide	Dipeptide	3463861	52766-27-5;	775.5	244.2
427.2			lysylvaline	LC/MS pos	42099			1.52	0.0186	0.0502	0.7895	1.2020	100	100	Peptide	Dipeptide	140709	20556-11-0;	876.2	246.2
428			phenylalanylleucine	LC/MS pos	40192			1.44	0.1987	0.2522	0.9214	1.3301	78	100	Peptide	Dipeptide	4078229	3303-55-7;	3252	279.2
431			serylleucine	LC/MS pos	40066			1.24	0.3950	0.3921	1.0097	1.2503	100	100	Peptide	Dipeptide		6665-16-3;	2106	219.2
432			serylphenylalanine	LC/MS pos	40054			0.91	0.9371	0.6080	1.0502	0.9544	89	100	Peptide	Dipeptide			2348	253.2
433			threonylleucine	LC/MS pos	40051			1.05	0.7746	0.5519	1.0579	1.1108	100	100	Peptide	Dipeptide	4420322	50299-12-2;	2204	233.2
445			valylisoleucine	LC/MS pos	40050			1.34	0.2285	0.2763	1.0491	1.4039	100	100	Peptide	Dipeptide	5246012;5246013	20556-14-3;	2365	231.2
448			serylisoleucine*	LC/MS pos	40193			1.24	0.5128	0.4396	1.0194	1.2629	100	100	Peptide	Dipeptide		510-791-9560;	2021	219.2
453.1			phenylalanylproline	LC/MS neg	41392			4.38	0.3670	0.3772	1.0887	4.7719	67	64	Peptide	Dipeptide	4069131	7669-65-0;	3170	261.2
460		gamma-glutamyl	gamma-glutamylvaline	LC/MS pos	32393		HMDB11172	1.12	0.7144	0.5442	1.3066	1.4581	100	100	Peptide	gamma-glutamyl		2746-34-1;	2040	247.2
462			gamma-glutamylleucine	LC/MS pos	18369		HMDB11171	0.97	0.8896	0.5938	1.2416	1.1986	100	100	Peptide	gamma-glutamyl	151023	2566-39-4;	2744	261.2
463			gamma-glutamylisoleucine*	LC/MS pos	34456		HMDB11170	0.91	0.7411	0.5486	1.2784	1.1600	100	100	Peptide	gamma-glutamyl			2644	261.2
466			gamma-glutamylmethionine	LC/MS pos	37539			0.62	0.5871	0.4836	1.7053	1.0606	100	91	Peptide	gamma-glutamyl	7009567	17663-87-5;	1993	279.2
467			gamma-glutamylglutamate	LC/MS pos	36738			0.65	0.9909	0.6201	1.8427	1.1899	100	100	Peptide	gamma-glutamyl	92865	1116-22-9;	1036	277.1
470			gamma-glutamylphenylalanine	LC/MS pos	33422		HMDB00594	0.85	0.6851	0.5412	1.3152	1.1207	100	100	Peptide	gamma-glutamyl	111299	7432-24-8;	2846	295.1
471			gamma-glutamyltyrosine	LC/MS pos	2734			0.62	0.0508	0.1053	1.4649	0.9076	100	100	Peptide	gamma-glutamyl	94340	7432-23-7;	2073	311.2
472			gamma-glutamylthreonine*	LC/MS pos	33364			1.05	0.5411	0.4587	1.1138	1.1673	100	100	Peptide	gamma-glutamyl		5652-48-2;	977	249.1
509	Carbohydrate	Aminosugars metabolism	glucosamine	GC/MS	18534	C00329	HMDB01514	2.22	0.0267	0.0652	0.7561	1.6753	100	91	Carbohydrate	Aminosugars metabolism	441477	66-84-2;	1833	203
510			N-acetylgalactosamine	GC/MS	5842	C01074	HMDB00835	3.39	0.0095	0.0305	0.7185	2.4333	100	100	Carbohydrate	Aminosugars metabolism	35717	14215-68-0;	1895	245.1
511			N-acetylglucosamine	GC/MS	15096	C00140	HMDB00215	3.25	0.0006	0.0045	0.7027	2.2823	100	100	Carbohydrate	Aminosugars metabolism	24139	7512-17-6;	1934.5	245
517			N-acetylneuraminate	LC/MS pos	32377	C00270	HMDB00230	2.68	0.0026	0.0116	0.8154	2.1873	100	100	Carbohydrate	Aminosugars metabolism		131-48-6;	787	310
523			fucose	GC/MS	15821	C02095	HMDB00174	3.56	0.0007	0.0045	0.7840	2.7898	100	100	Carbohydrate	Aminosugars metabolism	3034656	2438-80-4;	1682.2	204
525			N-acetylmannosamine	GC/MS	15059	C00140	HMDB00835	3.55	0.0012	0.0068	0.6224	2.2081	78	100	Carbohydrate	Aminosugars metabolism	439281	7772-94-3;	1882.5	245.1
535		Fructose, mannose, galactose, starch, and sucrose metabolism	N-acetylmuramate	LC/MS neg	18354	C02713		1.63	0.1472	0.2060	0.8359	1.3620	100	100	Carbohydrate	uctose, mannose, galactose, starch, and sucrose metabol	82756	10597-89-4;	877	292.1
537			fructose	GC/MS	577	C00095	HMDB00660	1.95	0.0453	0.0975	0.8351	1.6269	100	100	Carbohydrate	uctose, mannose, galactose, starch, and sucrose metabol	5984	57-48-7;	1758	217
539			galactose	GC/MS	12055	C01582	HMDB00143	3.25	0.0013	0.0068	0.7292	2.3666	100	100	Carbohydrate	uctose, mannose, galactose, starch, and sucrose metabol	3037556	59-23-4;	1793.8	203.9
546			cellobiose	GC/MS	15782	C00185	HMDB00055	0.95	0.4105	0.3992	1.0262	0.9756	100	91	Carbohydrate	uctose, mannose, galactose, starch, and sucrose metabol	10712	528-50-7;	2179	204
547			maltose	GC/MS	15806	C00208	HMDB00163	1.10	0.9034	0.5996	0.9738	1.0696	100	100	Carbohydrate	uctose, mannose, galactose, starch, and sucrose metabol	439341	6363-53-7;	2142.1	204.1
548			mannitol	GC/MS	15335	C00392	HMDB00765	7.68	0.3409	0.3649	0.0265	0.2035	11	9	Carbohydrate	uctose, mannose, galactose, starch, and sucrose metabol	6251	69-65-8;	1839	319.1
552			mannose	GC/MS	584	C00159	HMDB00169	2.61	0.0140	0.0396	0.7408	1.9299	100	100	Carbohydrate	uctose, mannose, galactose, starch, and sucrose metabol	161658	3458-28-4;	1753.5	203.9
554			mannose-6-phosphate	GC/MS	1470	C00275	HMDB01078	1.82	0.0589	0.1141	0.7816	1.4253	89	91	Carbohydrate	uctose, mannose, galactose, starch, and sucrose metabolism		70442-25-0;104872-94-8;	2035.9	387.2
567			maltotriose	GC/MS	27723	C01835	HMDB01262	0.62	0.5464	0.4616	2.0064	1.2418	100	100	Carbohydrate	uctose, mannose, galactose, starch, and sucrose metabol	439586	1109-28-0;	2368.3	361.2
582.8		Oligosaccharide	maltotetraose	LC/MS neg	15910	C02052	HMDB01296	0.44	0.9420	0.6084	3.4393	1.5043	100	100	Carbohydrate	Oligosaccharide	446495	34612-38-9;	965	665.1
585		Glycolysis, gluconeogenesis, pyruvate metabolism	glycerate	GC/MS	1572	C00258	HMDB00139	1.66	0.0852	0.1434	0.7434	1.2368	100	100	Carbohydrate	Glycolysis, gluconeogenesis, pyruvate metabolism	752	600-19-1;	1360.7	189
587			glucose-6-phosphate (G6P)	GC/MS	31260	C00668	HMDB01401	2.23	0.0100	0.0317	0.9347	2.0815	100	100	Carbohydrate	Glycolysis, gluconeogenesis, pyruvate metabolism		103192-55-8;	2042.7	387.2
589			glucose	GC/MS	20488	C00031	HMDB00122	2.68	0.0113	0.0347	0.7500	2.0118	100	100	Carbohydrate	Glycolysis, gluconeogenesis, pyruvate metabolism	79025	50-99-7;	1866.8	217.1
592			fructose-6-phosphate	GC/MS	12021	C05345	HMDB00124	1.77	0.0819	0.1409	0.8886	1.5729	100	100	Carbohydrate	Glycolysis, gluconeogenesis, pyruvate metabolism		103213-47-4;	1994.9	315.1
604			lactate	GC/MS	527	C00186	HMDB00190	2.24	0.4916	0.4309	1.2443	2.7830	100	100	Carbohydrate	Glycolysis, gluconeogenesis, pyruvate metabolism	612	79-33-4;	1102.8	116.9
622			ribose	GC/MS	12083	C00121	HMDB00283	1.42	0.4227	0.4007	1.0094	1.4364	100	100	Carbohydrate	Nucleotide sugars, pentose metabolism	5311110	50-69-1;	1652	204
623			2-deoxyribose	GC/MS	11734	C01801	HMDB03224	4.18	0.0831	0.1420	0.8630	3.6052	78	91	Carbohydrate	Nucleotide sugars, pentose metabolism	5460005	533-67-5;	1523.8	115.9

627		Nucleotide sugars, pentose metabolism	ribulose	GC/MS	35855	C00309	HMDB00621	2.41	0.0134	0.0393	0.7012	1.6910	100	100	Carbohydrate	Nucleotide sugars, pentose metabolism	79021	488-84-6;	1662	306.1
634			arabinose	GC/MS	575	C00216	HMDB00646	1.69	0.1070	0.1667	0.8443	1.4299	100	100	Carbohydrate	Nucleotide sugars, pentose metabolism	66308	28697-53-2;	1631.6	217
637			xylose	GC/MS	15835	C00181	HMDB00098	1.19	0.9214	0.6063	1.1519	1.3747	100	100	Carbohydrate	Nucleotide sugars, pentose metabolism	95259	609-06-3;	1723.9	204
639			xylulose	GC/MS	18344	C00310	HMDB00654	3.82	0.1354	0.1929	0.7317	2.7956	67	55	Carbohydrate	Nucleotide sugars, pentose metabolism	5289590	551-84-8;	1668	306.1
657			tricarballylate	LC/MS neg	15729	C19806		0.35	0.0938	0.1535	2.9019	1.0064	89	100	Energy	Krebs cycle	14925	99-14-9;	592	175
658			alpha-ketoglutarate	GC/MS	33453	C00026	HMDB00208	0.43	0.0386	0.0870	1.6422	0.7057	89	73	Energy	Krebs cycle	51	305-72-6;328-50-7;22202-68-2;	1779	419.1
659	Energy	Krebs cycle	succinate	GC/MS	1437	C00042	HMDB00254	1.29	0.6714	0.5378	1.1720	1.5140	100	100	Energy	Krebs cycle	1110	110-15-6;	1348	247
663			fumarate	GC/MS	1643	C00122	HMDB00134	1.07	0.5096	0.4395	0.9938	1.0651	100	100	Energy	Krebs cycle		100-17-8;	1382.1	245
667			malate	GC/MS	1303	C00149	HMDB00156	1.00	0.8694	0.5854	1.0907	1.0933	100	100	Energy	Krebs cycle	525	6915-15-7;	1502	233
671		Oxidative phosphorylation	phosphate	GC/MS	11438	C00009	HMDB01429	2.80	0.0038	0.0154	0.6997	1.9560	100	100	Energy	Oxidative phosphorylation	1061	7664-38-2 ;	1307.7	298.9
673			linoleate (18:2n6)	LC/MS neg	1105	C01595	HMDB00673	0.99	0.8791	0.5885	1.1000	1.0935	100	100	Lipid	Essential fatty acid	5280450	60-33-3;	5533	279.3
677			linolenate [alpha or gamma; (18:3n3 or 6)]	LC/MS neg	34035	C06427	HMDB01388	1.03	0.9459	0.6084	1.3523	1.3891	100	100	Lipid	Essential fatty acid			5450	277.3
678		Essential fatty acid	dihomo-linolenate (20:3n3 or n6)	LC/MS neg	35718	C03242	HMDB02925	4.94	0.0001	0.0008	0.5595	2.7637	100	100	Lipid	Essential fatty acid	5312529	17046-59-2 ;	5600	305.4
680			docosapentaenoate (n3 DPA; 22:5n3)	LC/MS neg	32504	C16513	HMDB01976	4.74	0.0004	0.0034	0.6378	3.0212	100	100	Lipid	Essential fatty acid		2234-74-4 ;	5574	329.4
681			docosapentaenoate (n6 DPA; 22:5n6)	LC/MS neg	37478	C16513	HMDB13123	5.32	0.0001	0.0015	0.3916	2.0820	78	100	Lipid	Essential fatty acid	6441454	25182-74-5;	5625	329.4
684		Short chain fatty acid	valerate	LC/MS neg	33443	C00803	HMDB00892	0.70	0.0345	0.0802	1.2472	0.8770	100	100	Lipid	Short chain fatty acid	7991	109-52-4;	1774	101.2
685			3-methyladipate	GC/MS	36749		HMDB00555	0.14	0.0005	0.0040	1.1716	0.1690	89	36	Lipid	Short chain fatty acid	12292	3058-01-3;	1546	289.2
687			caproate (6:0)	LC/MS neg	32489	C01585	HMDB00535	0.68	0.3168	0.3472	5.8459	3.9802	100	100	Lipid	Medium chain fatty acid	8892	142-62-1;	2766	115.2
688			heptanoate (7:0)	LC/MS neg	1644	C17714	HMDB00666	0.88	0.9129	0.6041	2.0407	1.7973	100	100	Lipid	Medium chain fatty acid	8094	111-14-8;	3695	129.2
689		Medium chain fatty acid	caprylate (8:0)	LC/MS neg	32492	C06423	HMDB00482	0.83	0.2148	0.2683	1.0405	0.8605	100	100	Lipid	Medium chain fatty acid	379	124-07-2;	4367	143.2
690			pelargonate (9:0)	LC/MS neg	12035	C01601	HMDB00847	0.73	0.0085	0.0294	1.1673	0.8550	100	100	Lipid	Medium chain fatty acid	5461016	112-05-0;	4847	157.2
691			caprate (10:0)	LC/MS neg	1642	C01571	HMDB00511	0.79	0.0355	0.0809	1.1457	0.9089	100	100	Lipid	Medium chain fatty acid	2969	112-37-8;	5092	171.2
694			laurate (12:0)	LC/MS neg	1645	C02679	HMDB00638	0.66	0.0114	0.0347	1.3093	0.8663	100	100	Lipid	Medium chain fatty acid	3893	143-07-7;	5288	199.3
698			myristate (14:0)	GC/MS	1365	C06424	HMDB00806	0.41	0.0000	0.0007	1.6570	0.6788	100	100	Lipid	Long chain fatty acid	11005	544-63-8;	1789.7	285.2
699			myristoleate (14:1n5)	LC/MS neg	32418	C08322	HMDB02000	0.65	0.0140	0.0396	1.3187	0.8605	100	100	Lipid	Long chain fatty acid	5281119	544-64-9 ;	5338	225.3
700			pentadecanoate (15:0)	GC/MS	1361	C16537	HMDB00826	0.33	0.0000	0.0007	1.9153	0.6340	100	100	Lipid	Long chain fatty acid	13849	1002-84-2;10002-84-2;	1853.5	299.2
701			palmitate (16:0)	LC/MS neg	1336	C00249	HMDB00220	0.95	0.5938	0.4874	1.0802	1.0245	100	100	Lipid	Long chain fatty acid	985	57-10-3;	5619	255.3
702			palmitoleate (16:1n7)	LC/MS neg	33447	C08362	HMDB03229	1.42	0.4911	0.4309	0.9111	1.2974	100	100	Lipid	Long chain fatty acid	445638	373-49-9;	5477	253.3
704			margarate (17:0)	LC/MS neg	1121		HMDB02259	1.40	0.4182	0.4007	1.0620	1.4825	100	100	Lipid	Long chain fatty acid	10465	506-12-7;	5733	269.3
705			10-heptadecenoate (17:1n7)	LC/MS neg	33971			1.47	0.1322	0.1908	0.8917	1.3079	100	100	Lipid	Long chain fatty acid	5312435	29743-97-3;	5558	267.3
706			stearate (18:0)	LC/MS neg	1358	C01530	HMDB00827	0.93	0.7174	0.5442	1.0602	0.9808	100	100	Lipid	Long chain fatty acid	5281	57-11-4;	5886	283.4
708			oleate (18:1n9)	GC/MS	1359	C00712	HMDB00207	0.59	0.0024	0.0112	1.3677	0.8027	100	100	Lipid	Long chain fatty acid	445639	112-80-1;	1984.4	339.2
710			cis-vaccenate (18:1n7)	GC/MS	33970	C08367		0.33	0.0000	0.0002	2.0446	0.6774	100	100	Lipid	Long chain fatty acid	5282761	506-17-2;	1987	339.3
716			nonadecanoate (19:0)	LC/MS neg	1356	C16535	HMDB00772	2.01	0.1220	0.1817	0.9806	1.9668	100	100	Lipid	Long chain fatty acid	12591	646-30-0;	6073	297.3
717		Long chain fatty acid	10-nonadecenoate (19:1n9)	LC/MS neg	33972			1.09	0.7416	0.5486	1.0057	1.0929	100	100	Lipid	Long chain fatty acid	5312513	73033-09-7;	5775	295.4
719			arachidate (20:0)	GC/MS	1118	C06425	HMDB02212	0.62	0.0061	0.0240	1.2806	0.7918	100	91	Lipid	Long chain fatty acid	10467	506-30-9;	2071.2	369.3
722			eicosenoate (20:1n9 or 11)	LC/MS neg	33587		HMDB02231	1.64	0.0943	0.1535	0.9528	1.5607	100	100	Lipid	Long chain fatty acid			5955	309.4
724			dihomo-linoleate (20:2n6)	LC/MS neg	17805	C16525		2.98	0.0035	0.0147	0.7417	2.2113	100	100	Lipid	Long chain fatty acid	6439848	2091-39-6;	5722	307.3
727			mead acid (20:3n9)	LC/MS neg	35174		HMDB10378	14.14	0.0001	0.0015	0.1782	2.5195	78	91	Lipid	Long chain fatty acid	5312531	20590-32-3;	5642	305.4
728			arachidonate (20:4n6)	LC/MS neg	1110	C00219	HMDB01043	3.72	0.0001	0.0010	0.6181	2.2985	100	100	Lipid	Long chain fatty acid	444899	506-32-1;	5525	303.4
731			behenate (22:0)	GC/MS	12125	C08281	HMDB00944	0.53	0.0007	0.0045	1.5181	0.8012	100	100	Lipid	Long chain fatty acid	8215	112-85-6;	2143	397.4
732			erucate (22:1n9)	LC/MS neg	1552	C08316	HMDB02068	1.31	0.3627	0.3750	1.0595	1.3926	100	100	Lipid	Long chain fatty acid	8216	112-86-7;	6273.3	337.4
733			docosadienoate (22:2n6)	LC/MS neg	32415	C16533		2.91	0.0062	0.0240	0.8143	2.3720	100	100	Lipid	Long chain fatty acid	5282807	7370-49-2 ;	6017	335.4
735			adrenate (22:4n6)	LC/MS neg	32980	C16527	HMDB02226	2.90	0.0007	0.0045	0.6066	1.7611	89	100	Lipid	Long chain fatty acid	5282844	2091-25-0;	5684	331.3
737			lignocerate (24:0)	GC/MS	1364	C08320	HMDB02003	0.74	0.1753	0.2287	1.3457	0.9998	100	100	Lipid	Long chain fatty acid	11197	557-59-5;	2211	425.4
738			nervonate (24:1n9)	GC/MS	41820	C08323	HMDB02368	1.62	0.1648	0.2239	0.8058	1.3079	44	82	Lipid	Long chain fatty acid	5281120	506-37-6 ;	2201	423.4
761			3-hydroxypropanoate	GC/MS	42103	C01013	HMDB00700	0.72	0.1641	0.2239	1.4108	1.0091	100	91	Lipid	Fatty acid, monohydroxy	5459847	503-66-2;	1187.1	177.1
763			5-hydroxyhexanoate	LC/MS neg	31938		HMDB00525	0.29	0.0231	0.0577	2.7087	0.7907	100	45	Lipid	Fatty acid, monohydroxy	170748	44843-89-2 ;	1283	131.2
768			3-hydroxydecanoate	LC/MS neg	22053		HMDB02203	1.35	0.3406	0.3649	0.8127	1.0957	100	100	Lipid	Fatty acid, monohydroxy	26612	5561-87-5;	4681	187.2
774		Fatty acid, monohydroxy	2-hydroxystearate	LC/MS neg	17945	C03045		1.26	0.2537	0.2992	0.8995	1.1358	100	100	Lipid	Fatty acid, monohydroxy	69417	629-22-1;	5705	299.4
776			2-hydroxypalmitate	GC/MS	35675			0.64	0.0469	0.0991	1.4278	0.9111	100	100	Lipid	Fatty acid, monohydroxy	92836	764-67-0;	1976	299.2
780			13-HODE + 9-HODE	LC/MS neg	37752			1.01	0.4862	0.4309	1.0554	1.0625	100	100	Lipid	Fatty acid, monohydroxy			5270	295.2
783		Fatty acid, dihydroxy	9,10-hydroxyoctadec-12(Z)-enoic acid	LC/MS neg	38399	C14828	HMDB04704	0.87	0.3030	0.3336	1.1857	1.0359	100	100	Lipid	Fatty acid, dihydroxy	9966640	263399-34-4;	5177	313.3
785			adipate	LC/MS pos	21134	C06104	HMDB00448	0.31	0.0000	0.0008	2.1074	0.6622	100	100	Lipid	Fatty acid, dicarboxylate	196	124-04-9;	2460	147
786			2-hydroxyglutarate	GC/MS	37253	C02630	HMDB00606	2.26	0.7085	0.5442	1.6831	3.8091	100	100	Lipid	Fatty acid, dicarboxylate	43	40951-21-1;	1576	247
790			suberate (octanedioate)	LC/MS pos	15730	C08278	HMDB00893	0.53	0.0000	0.0008	1.3359	0.7088	100	100	Lipid	Fatty acid, dicarboxylate	10457	505-48-6;	3603	175
791			2-octenedioate	LC/MS neg	35120		HMDB00341	0.30	0.0000	0.0000	1.3657	0.4151	100	45	Lipid	Fatty acid, dicarboxylate	5354607	5698-50-0;	1291	171.1
793			sebacate (decanedioate)	LC/MS neg	32398	C08277	HMDB00792	0.30	0.0000	0.0008	1.7776	0.5285	100	100	Lipid	Fatty acid, dicarboxylate	5192	111-20-6;	1778	201.2
794		Fatty acid, dicarboxylate	azelate (nonanedioate)	LC/MS neg	18362	C08261	HMDB00784	0.33	0.0001	0.0011	1.6737	0.5493	100	100	Lipid	Fatty acid, dicarboxylate	2266	123-99-9;	1322	187.2
796			dodecanedioate	LC/MS neg	32388	C02678	HMDB00623	0.27	0.0003	0.0031	1.9700	0.5325	100	100	Lipid	Fatty acid, dicarboxylate	12736	693-23-2 ;	2990	229.2
797			tetradecanedioate	LC/MS neg	35669		HMDB00872	0.54	0.0005	0.0040	1.3234	0.7137	100	100	Lipid	Fatty acid, dicarboxylate	13185	821-38-5;	3970	257.2
798			hexadecanedioate	LC/MS neg	35678		HMDB00672	0.82	0.2017	0.2546	1.1436	0.9432	100	100	Lipid	Fatty acid, dicarboxylate	10459	505-54-4;	4650	285.2
799			octadecanedioate	LC/MS neg	36754		HMDB00782	0.69	0.0526	0.1062	1.3216	0.9091	100	100	Lipid	Fatty acid, dicarboxylate	70095	871-70-5;	5033	313.3
800			undecanedioate	LC/MS neg	35671		HMDB00888	0.38	0.0000	0.0008	1.5270	0.5874	100	100	Lipid	Fatty acid, dicarboxylate	15816	1852-04-6;	2376	215.1
816			12-methyltridecanoic acid	GC/MS	38292			0.22	0.0000	0.0002	2.4233	0.5280	100	91	Lipid	Fatty acid, branched	520298	2724-57-4;	1764	285.2
817			13-methylmyristic acid	GC/MS	38293			0.42	0.0003	0.0027	1.7329	0.7201	100	100	Lipid	Fatty acid, branched	151014	2485-71-4;	1833	299.2
819		Fatty acid, branched	15-methylpalmitate (isobar with 2-methylpalmitate)	LC/MS neg	38768			1.12	0.7239	0.5442	1.2610	1.4118	100	100	Lipid	Fatty acid, branched			5698	269.4
820			isopalmitic acid	GC/MS	38294			0.29	0.0000	0.0008	2.2970	0.6738	100	100	Lipid	Fatty acid, branched	36247	4669-02-7;	1888	313.3
821	Lipid		17-methylstearate	LC/MS neg	38296			0.98	0.9002	0.5992	1.2070	1.1829	100	100	Lipid	Fatty acid, branched	3083779	2724-59-6;	5987	297.4
880			oleic ethanolamide	LC/MS neg	38102		HMDB02088	3.20	0.0220	0.0555	0.8684	2.7752	100	100	Lipid	Endocannabinoid	5283454	11-58-0;111-58-0;	6553	324.5
881		Endocannabinoid	palmitoyl ethanolamide	LC/MS neg	38165		HMDB02100	4.38	0.0069	0.0263	0.8389	3.6785	100	100	Lipid	Endocannabinoid	4671	544-31-0;	6416	298.4
882			stearoyl ethanolamide	LC/MS pos	38625		HMDB13078	2.06	0.0303	0.0711	0.7032	1.4462	67	91	Lipid	Endocannabinoid	27902	111-57-9;	5968.9	328.3
894		Fatty acid metabolism	isovalerate	LC/MS neg	34732	C08262	HMDB00718	0.47	0.0008	0.0050	1.5760	0.7402	100	100	Lipid	Fatty acid metabolism	10430	503-74-2;	1623	101.2
897		Fatty acid synthesis	malonate (propanedioate)	LC/MS neg	15872	C00383	HMDB00691	0.99	0.9617	0.6084	0.9780	0.9639	100	100	Lipid	Fatty acid synthesis	867	141-82-2;26522-22-85-0;	606	103.2
898		Carnitine metabolism	deoxycarnitine	LC/MS pos	36747	C01181	HMDB01161	0.91	0.3549	0.3714	1.0032	0.9163	100	100	Lipid	Carnitine metabolism	725	6249-56-5;	759	146.1
900			3-dehydrocarnitine*	LC/MS pos	32654	C02636	HMDB12154	0.63	0.2041	0.2563	1.4798	0.9324	100	100	Lipid	Carnitine metabolism	6991982	10457-99-5;	1020	160.2
916			cholate	LC/MS pos	22842	C00695	HMDB00619	2.05	0.2931	0.3251	0.7191	1.4736	22	45	Lipid	Bile acid metabolism		81-25-4;	5322	426.2
919.1			glycohyocholate	LC/MS pos	42574			3.01	0.4078	0.3992	1.1247	3.3847	78	82	Lipid	Bile acid metabolism			5115	466.2
920.1			taurohyocholate	LC/MS neg	42603			5.28	0.4470	0.4154	1.9270	10.1784	100	91	Lipid	Bile acid metabolism			5000	514.3
921			chenodeoxycholate	LC/MS neg	37789	C02528	HMDB00518	3.68	0.0789	0.1377	1.1381	4.1830	100	100	Lipid	Bile acid metabolism		474-24-9;474-25-9;	5250	391.4
922			taurochenodeoxycholate	LC/MS neg	18494	C05465	HMDB00951	1.30	0.8694	0.5854	1.3980	1.8114	100	100	Lipid	Bile acid metabolism	387316	6009-98-9;	5218	498.3
924			hyodeoxycholate	LC/MS neg	27531	C15517	HMDB00733	1.43	0.2296	0.2763	0.8665	1.2390	100	100	Lipid	Bile acid metabolism	5283820	83-49-8;	5074	391.4
930			glycochenodeoxycholate	LC/MS pos	32346	C05466	HMDB00637	1.11	0.9360	0.6080	0.8893	0.9845	56	45	Lipid	Bile acid metabolism		16564-43-5;	5320	450.1
935		Bile acid metabolism	lithocholate	LC/MS neg	1483	C03990	HMDB00761	1.15	0.6246	0.5055	1.1238	1.2971	100	100	Lipid	Bile acid metabolism	9903	434-13-9;	5350	375.4
936			dehydrolithocholate	LC/MS neg	31891			0.38	0.0014	0.0076	1.5019	0.5776	100	100	Lipid	Bile acid metabolism	159623	1553-56-6 ;	5337	373.4
939			6-oxolithocholate	LC/MS neg	31902			0.59	0.0395	0.0883	1.3635	0.8011	100	100	Lipid	Bile acid metabolism	164960	2393-61-5 ;	5040	389.4
941			6-beta-hydroxylithocholate	LC/MS neg	36807	C15515	HMDB00811	0.97	0.4617	0.4179	1.1402	1.1014	100	100	Lipid	Bile acid metabolism	5283821	668-49-5;	4826	391.3
943			beta-muricholate	LC/MS pos	31885	C17726	HMDB00415	1.49	0.1718	0.2283	1.0233	1.5257	89	100	Lipid	Bile acid metabolism	3083762	2393-59-1;	5191	391.2
944			hyocholate	LC/MS neg	34093	C17649	HMDB00760	4.65	0.0146	0.0406	0.7315	3.4048	100	100	Lipid	Bile acid metabolism		547-75-1;	5001	407.3
951			tauroursodeoxycholate	LC/MS neg	39378		HMDB00874	2.03	0.7084	0.5442	0.8151	1.6552	56	82	Lipid	Bile acid metabolism		14605-22-2;	5002.6	498.4
952.1			tauro(alpha + beta)muricholate	LC/MS neg	43167			3.18	0.7410	0.5486	0.8606	2.7409	56	27	Lipid	Bile acid metabolism			4743.7	514.4
953		Fatty alcohol, long chain	1-octadecanol	GC/MS	21128	D01924	HMDB02350	0.83	0.2895	0.3249	1.1732	0.9717	100	100	Lipid	Fatty alcohol, long chain	8221	112-92-5;	1957.7	327.3
955			ethanolamine	GC/MS	1497	C00189	HMDB00149	0.95	0.2265	0.2763	1.5235	1.4485	100	100	Lipid	Glycerolipid metabolism		141-43-5;	1304	174.1
959		Glycerolipid metabolism	glycerol	GC/MS	15122	C00116	HMDB00131	0.96	0.4028	0.3965	1.1003	1.0581	100	100	Lipid	Glycerolipid metabolism	753	56-81-5;	1311	205
961			glycerol 3-phosphate (G3P)	GC/MS	15365	C00093	HMDB00126	0.65	0.0425	0.0931	1.4040	0.9135	100	100	Lipid	Glycerolipid metabolism	754	29849-82-9;	1719.7	357.1
962			glycerophosphorylcholine (GPC)	LC/MS pos	15990	C00670	HMDB00086	0.77	0.0874	0.1460	1.1190	0.8655	100	100	Lipid	Glycerolipid metabolism	657272	28319-77-9;	694	258.1
971		Inositol metabolism	myo-inositol	GC/MS	19934	C00137	HMDB00211	3.53	0.0090	0.0302	0.8705	3.0704	100	100	Lipid	Inositol metabolism		87-89-8;	1924.9	217
974			inositol 1-phosphate (I1P)	GC/MS	1481	C04006	HMDB00213	1.26	0.2533	0.2992	1.0925	1.3788	100	100	Lipid	Inositol metabolism		106032-59-1;	2057.8	318.1
984		Ketone bodies	3-hydroxybutyrate (BHBA)	GC/MS	542	C01089	HMDB00357	0.48	0.0002	0.0023	1.8950	0.9074	100	100	Lipid	Ketone bodies	441	625-72-9;	1203.5	116.9
990			1-palmitoylglycerophosphoethanolamine	LC/MS neg	35631		HMDB11503	1.19	0.4589	0.4179	0.8651	1.0281	100	100	Lipid	Lysolipid	9547069		5940	452.3
991			2-palmitoylglycerophosphoethanolamine*	LC/MS neg	35688			1.50	0.2327	0.2787	0.8206	1.2324	100	100	Lipid	Lysolipid			5790	452.3
995			1-stearoylglycerophosphoethanolamine	LC/MS neg	34416		HMDB11130	1.59	0.0459	0.0978	0.8087	1.2869	100	100	Lipid	Lysolipid	9547068	69747-55-3;	6200	480.4
1006			1-stearoylglycerophosphoglycerol	LC/MS neg	34437			0.95	0.7616	0.5517	1.6897	1.6104	100	100	Lipid	Lysolipid			5826	511.4
1010			1-palmitoylglycerophosphocholine	LC/MS pos	33955			1.08	0.8031	0.5636	1.0429	1.1257	100	100	Lipid	Lysolipid	86554	17364-16-8;	5671	496.4
1011		Lysolipid	2-palmitoylglycerophosphocholine*	LC/MS pos	35253			1.01	0.8647	0.5854	0.9176	0.9293	56	55	Lipid	Lysolipid			5604	496.3
1015			1-stearoylglycerophosphocholine	LC/MS pos	33961			1.15	0.4786	0.4276	0.8521	0.9841	100	100	Lipid	Lysolipid	497299	19420-57-6;	5844	524.4
1017			1-oleoylglycerophosphocholine	LC/MS pos	33960			0.92	0.8581	0.5833	1.0984	1.0079	89	82	Lipid	Lysolipid	16081932	19420-56-5;	5700	522.4
1019			1-linoleoylglycerophosphocholine	LC/MS pos	34419	C04100		0.67	0.9214	0.6063	1.4777	0.9919	67	82	Lipid	Lysolipid	11988421		5574	520.4
1032			1-palmitoylglycerophosphoinositol*	LC/MS neg	35305			1.05	0.9852	0.6185	1.3565	1.4275	100	100	Lipid	Lysolipid			5573	571.3
1035			1-stearoylglycerophosphoinositol	LC/MS neg	19324			1.76	0.0170	0.0464	0.6554	1.1556	67	100	Lipid	Lysolipid		796963-93-4;	5800	599.4
1045			1-palmitoylplasmenylethanolamine*	LC/MS neg	39270			1.23	0.4897	0.4309	1.1229	1.3820	100	100	Lipid	Lysolipid			6153	436.4
1049			1-pentadecanoylglycerol (1-monopentadecanoin)	LC/MS neg	34385			0.23	0.0012	0.0066	3.4322	0.7726	100	100	Lipid	Monoacylglycerol	190750		5978	241.3
1051			1-palmitoylglycerol (1-monopalmitin)	GC/MS	21127			1.08	0.8580	0.5833	1.0107	1.0954	100	100	Lipid	Monoacylglycerol	14900	542-44-9;	2119.5	371.3
1055		Monoacylglycerol	1-oleoylglycerol (1-monoolein)	LC/MS pos	21184		HMDB11567	2.78	0.0031	0.0135	0.7446	2.0711	100	100	Lipid	Monoacylglycerol	5283468	111-03-5;	5821	374.1
1056			2-oleoylglycerol (2-monoolein)	GC/MS	35848			1.44	0.2215	0.2737	0.6666	0.9600	33	73	Lipid	Monoacylglycerol	5319879	3443-84-3;	2170	410.1
1057			1-linoleoylglycerol (1-monolinolein)	LC/MS neg	27447			2.31	0.0643	0.1235	1.3035	3.0152	100	100	Lipid	Monoacylglycerol	5283469	2277-28-3;	6226	279.2
1058			2-linoleoylglycerol (2-monolinolein)	LC/MS neg	32506		HMDB11538	2.59	0.0549	0.1091	1.0517	2.7195	100	100	Lipid	Monoacylglycerol	5365676	3443-82-1;	5988	279.3
1074			sphinganine	LC/MS pos	17769	C00836	HMDB00269	1.37	0.2579	0.3011	0.8541	1.1687	100	100	Lipid	Sphingolipid	3126	3102-56-5;	5175	302.3
1077			sphingosine	LC/MS pos	17747	C00319	HMDB00252	2.79	0.0080	0.0287	0.7196	2.0070	100	100	Lipid	Sphingolipid	5353955	123-78-4;	5197	300.2
1078		Sphingolipid	phytosphingosine	LC/MS pos	1510	C12144	HMDB04610	1.35	0.3855	0.3859	0.8893	1.1983	100	100	Lipid	Sphingolipid		554-62-1;	5189	318.4
1080			N-acetylsphingosine	LC/MS neg	17772	C00195		1.03	0.5109	0.4395	1.1972	1.2385	100	100	Lipid	Sphingolipid	5497136	3102-57-6;	5905	340.4
1081			N,N-dimethylsphingosine	LC/MS pos	32460	C13914		2.32	0.0018	0.0087	0.5708	1.3218	78	100	Lipid	Sphingolipid	5282309	119567-63-4;	5192	328.3
1086			palmitoyl sphingomyelin	GC/MS	37506			2.50	0.0093	0.0305	0.7724	1.9321	89	100	Lipid	Sphingolipid	9939941	6254-89-3;	2524	311.3
1100			lathosterol	GC/MS	39864	C01189	HMDB01170	1.07	0.4000	0.3954	0.8371	0.8946	44	73	Lipid	Sterol/Steroid	65728	80-99-9 ;	2337	255.2
1102		Sterol/Steroid	cholesterol	GC/MS	63	C00187	HMDB00067	1.38	0.0437	0.0948	0.8608	1.1864	100	100	Lipid	Sterol/Steroid	6432564	57-88-5;	2316.9	329.3
1139			beta-sitosterol	GC/MS	27414	C01753	HMDB00852	0.68	0.0008	0.0051	1.2197	0.8279	100	100	Lipid	Sterol/Steroid	222284	83-46-5;	2380.8	357.4
1141			campesterol	GC/MS	39511	C01789	HMDB02869	0.60	0.0004	0.0034	1.2483	0.7476	100	100	Lipid	Sterol/Steroid	173183	474-62-4;	2353	343.4
1187			xanthine	LC/MS pos	3147	C00385	HMDB00292	1.16	0.4953	0.4325	1.0053	1.1663	100	100	Nucleotide	Purine metabolism, (hypo)xanthine/inosine containing	1188	69-89-6;	1430	153.1
1188			xanthosine	LC/MS neg	15136	C01762	HMDB00299	0.85	0.5679	0.4711	0.9843	0.8382	89	100	Nucleotide	Purine metabolism, (hypo)xanthine/inosine containing	64959	146-80-5;	1075	283.1
1190		Purine metabolism, (hypo)xanthine/inosine containing	hypoxanthine	GC/MS	3127	C00262	HMDB00157	1.36	0.6481	0.5210	1.0236	1.3957	100	100	Nucleotide	Purine metabolism, (hypo)xanthine/inosine containing	790	68-94-0;	1759.2	265
1192			inosine	GC/MS	1123	C00294	HMDB00195	1.71	0.4153	0.4003	0.7206	1.2327	44	45	Nucleotide	Purine metabolism, (hypo)xanthine/inosine containing		58-63-9;	2114.8	217
1193			2'-deoxyinosine	LC/MS pos	15076	C05512	HMDB00071	1.02	0.9639	0.6084	1.0928	1.1131	100	100	Nucleotide	Purine metabolism, (hypo)xanthine/inosine containing	65058	890-38-0;	1750	252.9
1198			adenine	LC/MS pos	554	C00147	HMDB00034	0.43	0.0034	0.0147	1.7852	0.7713	100	100	Nucleotide	Purine metabolism, adenine containing	190	73-24-5;	1003	136.1
1199			adenosine	LC/MS pos	555	C00212	HMDB00050	0.58	0.0513	0.1055	1.5949	0.9222	100	100	Nucleotide	Purine metabolism, adenine containing	60961	58-61-7;	1650	268.1
1203			2'-deoxyadenosine	LC/MS pos	1553	C00559	HMDB00101	0.45	0.0093	0.0305	1.7327	0.7723	100	91	Nucleotide	Purine metabolism, adenine containing	13730	16373-93-6;	1632	252.1
1205		Purine metabolism, adenine containing	2'-deoxyadenosine 3'-monophosphate	LC/MS pos	40663			1.20	0.4812	0.4283	0.8901	1.0702	100	100	Nucleotide	Purine metabolism, adenine containing	1467	102832-10-0;	1383.5	332.1
1206			adenosine 2'-monophosphate (2'-AMP)	LC/MS pos	36815	C00946	HMDB11617	2.46	0.0007	0.0047	0.5689	1.4001	100	100	Nucleotide	Purine metabolism, adenine containing		130-49-4;	1625	348.1
1207			adenosine 3'-monophosphate (3'-AMP)	LC/MS pos	35142	C01367	HMDB03540	0.66	0.2459	0.2930	1.5231	1.0125	100	100	Nucleotide	Purine metabolism, adenine containing	15938966	84-21-9;	1451	348.1
1208			adenosine 5'-monophosphate (AMP)	LC/MS pos	32342	C00020	HMDB00045	1.32	0.8397	0.5788	1.0684	1.4147	100	100	Nucleotide	Purine metabolism, adenine containing	15938965	149022-20-8;	1210	348.1
1214			adenosine-2',3'-cyclic monophosphate	LC/MS pos	37467	C02353		0.67	0.0815	0.1409	1.2297	0.8213	100	91	Nucleotide	Purine metabolism, adenine containing	2024	37063-35-7;	1484	330.2
1220			guanine	LC/MS pos	32352	C00242	HMDB00132	1.02	0.7190	0.5442	1.0866	1.1067	100	100	Nucleotide	Purine metabolism, guanine containing	764	73-40-5;	1022	152.1
1224			guanosine	LC/MS pos	1573	C00387	HMDB00133	1.45	0.9619	0.6084	2.0950	3.0277	89	100	Nucleotide	Purine metabolism, guanine containing	6802	118-00-3;	1676	284
1225		Purine metabolism, guanine containing	2'-deoxyguanosine	LC/MS pos	1411	C00330	HMDB00085	0.94	0.5149	0.4398	1.0101	0.9524	100	100	Nucleotide	Purine metabolism, guanine containing	187790	961-07-9;	1728	268
1228			guanosine-2',3'-cyclic monophosphate	LC/MS pos	37139	C06194	HMDB11629	0.94	0.3764	0.3818	1.0459	0.9883	100	91	Nucleotide	Purine metabolism, guanine containing	417655	15718-49-7;	1620	346
1245			guanosine 3'-monophosphate (3'-GMP)	LC/MS neg	39786			1.38	0.6843	0.5412	0.7351	1.0149	22	45	Nucleotide	Purine metabolism, guanine containing	3522	117-68-0;	792.2	362.1
1247	Nucleotide	Purine metabolism, urate metabolism	urate	GC/MS	1604	C00366	HMDB00289	0.39	0.0007	0.0045	1.6018	0.6242	100	73	Nucleotide	Purine metabolism, urate metabolism		69-93-2;120K5305;	1928	441.2
1251			cytidine	LC/MS pos	514	C00475	HMDB00089	1.08	0.9520	0.6084	0.9200	0.9907	100	100	Nucleotide	Pyrimidine metabolism, cytidine containing	6175	65-46-3;	1065	244
1255			cytidine 5'-monophosphate (5'-CMP)	LC/MS pos	2372	C00055	HMDB00095	1.49	0.4248	0.4011	0.9712	1.4466	78	82	Nucleotide	Pyrimidine metabolism, cytidine containing	7058165	63-37-6;	887	324
1256			cytidine-3'-monophosphate (3'-CMP)	LC/MS pos	2959	C05822		1.38	0.5531	0.4655	1.0415	1.4347	100	100	Nucleotide	Pyrimidine metabolism, cytidine containing	256622	84-52-6;	1181	323.9
1257		Pyrimidine metabolism, cytidine containing	cytosine-2',3'-cyclic monophosphate	LC/MS pos	37465	C02354	HMDB11691	0.77	0.2658	0.3087	1.1940	0.9245	100	100	Nucleotide	Pyrimidine metabolism, cytidine containing	417654	15718-51-1;	912	306.2
1260			2'-deoxycytidine	LC/MS pos	15949	C00881	HMDB00014	1.70	0.0203	0.0523	0.7342	1.2499	89	100	Nucleotide	Pyrimidine metabolism, cytidine containing	13711	951-77-9;	1256	228
1261			2'-deoxycytidine 3'-monophosphate	LC/MS pos	40453			0.94	0.8555	0.5833	1.0140	0.9559	100	100	Nucleotide	Pyrimidine metabolism, cytidine containing	3274948	6220-63-9;	1048	308
1263			cytosine	GC/MS	573	C00380	HMDB00630	1.83	0.5091	0.4395	1.0530	1.9261	100	100	Nucleotide	Pyrimidine metabolism, cytidine containing	597	71-30-7;	1535.5	254
1267		Pyrimidine metabolism, orotate containing	orotate	GC/MS	1505	C00295	HMDB00226	0.85	0.4192	0.4007	1.4712	1.2573	100	100	Nucleotide	Pyrimidine metabolism, orotate containing	967	50887-69-9;	1709.6	254.1
1271			thymine	GC/MS	604	C00178	HMDB00262	1.73	0.5653	0.4706	1.1594	2.0107	100	100	Nucleotide	Pyrimidine metabolism, thymine containing	1135	65-71-4;	1429.1	255
1272		Pyrimidine metabolism, thymine containing	thymidine	LC/MS neg	2183	C00214	HMDB00273	1.22	0.3570	0.3718	1.2994	1.5809	100	100	Nucleotide	Pyrimidine metabolism, thymine containing	5789	50-89-5;	1935	241.1
1274			thymidine 5'-monophosphate	LC/MS pos	12023	C00364	HMDB01227	0.89	0.7231	0.5442	1.0562	0.9360	100	100	Nucleotide	Pyrimidine metabolism, thymine containing		33430-62-5;	1598	322.9
1277			uracil	GC/MS	605	C00106	HMDB00300	1.45	0.7072	0.5442	1.0072	1.4647	100	100	Nucleotide	Pyrimidine metabolism, uracil containing	1174	66-22-8;	1370.4	241
1279			uridine	LC/MS neg	606	C00299	HMDB00296	1.17	0.7624	0.5517	1.1314	1.3201	100	100	Nucleotide	Pyrimidine metabolism, uracil containing	6029	58-96-8;	1430	243.1
1280			pseudouridine	LC/MS pos	33442	C02067	HMDB00767	1.09	0.7046	0.5442	1.0787	1.1811	100	100	Nucleotide	Pyrimidine metabolism, uracil containing		1445-07-4;	1180	245
1281		Pyrimidine metabolism, uracil containing	2'-deoxyuridine	LC/MS neg	1412	C00526	HMDB00012	1.18	0.5807	0.4800	1.2022	1.4168	89	64	Nucleotide	Pyrimidine metabolism, uracil containing	13712	951-78-0;	1608	227.1
1282			5-methyluridine (ribothymidine)	LC/MS neg	35136		HMDB00884	1.04	0.9559	0.6084	1.2518	1.2991	89	91	Nucleotide	Pyrimidine metabolism, uracil containing	445408	1463-10-1;	1774	257.1
1287			uridine monophosphate (5' or 3')	LC/MS neg	39879		HMDB00288	1.14	0.8784	0.5885	0.9719	1.1067	89	73	Nucleotide	Pyrimidine metabolism, uracil containing		58-97-9;	1079	325
1288			uridine-2',3'-cyclic monophosphate	LC/MS neg	37137	C02355	HMDB11640	1.32	0.4545	0.4169	0.9428	1.2416	56	82	Nucleotide	Pyrimidine metabolism, uracil containing	4634036	40632-52-8;	1082	304.9
1313			heme	LC/MS pos	41754	C00032	HMDB03178	1.87	0.1589	0.2211	0.3345	0.6241	0	36	Cofactors and vitamins	Hemoglobin and porphyrin metabolism		14875-96-8;	5057	616.2
1314		Hemoglobin and porphyrin metabolism	L-urobilin	LC/MS pos	40173	C05793	HMDB04159	0.61	0.0349	0.0802	1.3140	0.7954	100	100	Cofactors and vitamins	Hemoglobin and porphyrin metabolism	5280818	34217-90-8;	4091	595.4
1316			D-urobilin	LC/MS pos	40084	C05795	HMDB04161	6.64	0.0016	0.0081	0.2311	1.5334	11	73	Cofactors and vitamins	Hemoglobin and porphyrin metabolism		3947-38-4;	4621	589.3
1320			biliverdin	LC/MS neg	2137	C00500	HMDB01008	1.77	0.3183	0.3472	0.8351	1.4781	78	82	Cofactors and vitamins	Hemoglobin and porphyrin metabolism		55482-27-4;55482-27-4 ;	4671	581.2
1338		Nicotinate and nicotinamide metabolism	nicotinate	LC/MS pos	1504	C00253	HMDB01488	1.09	0.7868	0.5555	0.9846	1.0719	100	100	Cofactors and vitamins	Nicotinate and nicotinamide metabolism	938	59-67-6;	1221	122.1
1341			nicotinate ribonucleoside*	LC/MS pos	33471	C05841	HMDB06809	2.38	0.1614	0.2232	0.7024	1.6716	67	64	Cofactors and vitamins	Nicotinate and nicotinamide metabolism	161233	2625-49-2;	1105	256
1347			pantothenate	LC/MS pos	1508	C00864	HMDB00210	1.18	0.7212	0.5442	1.0302	1.2136	100	100	Cofactors and vitamins	Pantothenate and CoA metabolism	6613	137-08-6;	2218	220.1
1348	Cofactors and	Pantothenate and CoA metabolism	pantethine	LC/MS neg	39791	C12661	HMDB03828	1.49	0.0945	0.1535	0.8539	1.2734	89	91	Cofactors and vitamins	Pantothenate and CoA metabolism	452306	16816-67-4;	3605.3	553.3

1363	vitamins		2,3-dihydroxyisovalerate	GC/MS	38276	C04039		0.53	0.0490	0.1026	0.6949	0.3708	44	9	Cofactors and vitamins	Pantothenate and CoA metabolism	677	1756-18-9;	1422	131
1365		Riboflavin metabolism	flavin adenine dinucleotide (FAD)	LC/MS neg	2134	C00016	HMDB01248	1.07	0.1217	0.1817	0.8427	0.8989	11	27	Cofactors and vitamins	Riboflavin metabolism	643975	146-14-5;84366-81-4;	2413	784.1
1366			riboflavin (Vitamin B2)	LC/MS pos	1827	C00255	HMDB00244	1.11	0.6033	0.4919	0.9913	1.1029	100	100	Cofactors and vitamins	Riboflavin metabolism	493570	83-88-5;	3111	377.2
1372			alpha-tocopherol	GC/MS	1561	C02477	HMDB01893	0.40	0.0001	0.0011	1.6374	0.6596	100	100	Cofactors and vitamins	Tocopherol metabolism	14985	59-02-9;10191-41-0;	2305.4	502.5
1377		Tocopherol metabolism	gamma-tocopherol	GC/MS	42315	C02483	HMDB01492	0.42	0.0000	0.0000	1.6587	0.6899	100	100	Cofactors and vitamins	Tocopherol metabolism	14986	54-28-4;	2264	223.1
1378			alpha-tocotrienol	GC/MS	37502	C14153	HMDB06327	0.53	0.1720	0.2283	0.8461	0.4455	33	27	Cofactors and vitamins	Tocopherol metabolism	5282347	58864-81-6;	2346	237.1
1379			gamma-tocotrienol	GC/MS	37501	C14155	HMDB12958	0.54	0.0002	0.0024	1.0778	0.5872	100	18	Cofactors and vitamins	Tocopherol metabolism	5282349	14101-61-2;	2297	223.1
1389		Vitamin B6 metabolism	pyridoxate	LC/MS neg	31555	C00847	HMDB00017	0.30	0.0073	0.0275	2.4135	0.7328	100	100	Cofactors and vitamins	Vitamin B6 metabolism	6723	82-82-6 ;	2210	182.1
1408			benzoate	GC/MS	15778	C00180	HMDB01870	1.00	0.7691	0.5517	1.0475	1.0480	100	100	Xenobiotics	Benzoate metabolism	243	65-85-0;	1291.5	179
1409		Benzoate metabolism	3-hydroxybenzoate	GC/MS	15673	C00587	HMDB02466	0.97	0.4117	0.3992	0.9965	0.9709	89	55	Xenobiotics	Benzoate metabolism	7420	99-06-9;	1574.2	267
1417			p-aminobenzoate (PABA)	LC/MS pos	596	C00568	HMDB01392	0.33	0.0006	0.0045	1.6330	0.5469	100	73	Xenobiotics	Benzoate metabolism	7057990;978	150-13-0;	2120	138.1
1430			glycolate (hydroxyacetate)	GC/MS	15737	C00160	HMDB00115	5.20	0.0201	0.0523	0.8544	4.4453	78	100	Xenobiotics	Chemical	3698251;757	79-14-1;	1119	177
1451		Chemical	3-hydroxypyridine	GC/MS	21169			0.69	0.0425	0.0931	1.1564	0.7996	100	82	Xenobiotics	Chemical	7971	109-00-2;	1187.1	152
1518.93			2,8-quinolinediol	LC/MS pos	42975	C06342	C06342	0.35	0.0139	0.0396	1.8411	0.6503	100	100	Xenobiotics	Chemical	97250	15450-76-7;	3305	162.2
1583		Drug	pentobarbital	LC/MS neg	38156			1.36	0.9844	0.6185	0.9963	1.3548	100	82	Xenobiotics	Drug	4737	76-74-4;	4564	225.2
1658			sinapate	LC/MS neg	21150	C00482		1.02	0.9309	0.6080	1.1968	1.2258	100	100	Xenobiotics	Food component/Plant	637775	530-59-6;	1986	223.1
1664.2			1-methyl-beta-carboline-3-carboxylic acid	LC/MS pos	42938			0.29	0.0000	0.0002	1.4140	0.4165	100	55	Xenobiotics	Food component/Plant	5406157	22329-38-0;	3264.1	227.1
1670			daidzein	LC/MS neg	32453	C10208	HMDB03312	1.04	0.3848	0.3859	1.7050	1.7778	100	100	Xenobiotics	Food component/Plant	5281708	486-66-8 ;	3541	253.1
1672			genistein	LC/MS neg	32448	C06563	HMDB03217	1.34	0.1318	0.1908	1.6939	2.2668	100	100	Xenobiotics	Food component/Plant	5280961	446-72-0 ;	3745	269.1
1683			enterolactone	LC/MS neg	39626			0.38	0.0052	0.0208	1.9113	0.7315	100	100	Xenobiotics	Food component/Plant	10685477	78473-71-9;	4492.7	297.2
1697			apigenin	LC/MS neg	21189	C01477	HMDB02124	1.69	0.1748	0.2287	1.3909	2.3529	89	100	Xenobiotics	Food component/Plant	5280443	520-36-5;	3880	269.1
1700	Xenobiotics		ferulate	LC/MS neg	35630	C01494	HMDB00954	1.06	0.9550	0.6084	0.9383	0.9918	100	100	Xenobiotics	Food component/Plant	445858	537-98-4;	1810	193
1701			dihydroferulic acid	LC/MS neg	40481			6.28	0.0195	0.0520	0.6917	4.3451	100	100	Xenobiotics	Food component/Plant	14340	1135-23-5;	1929	195.1
1715			equol	LC/MS neg	36774	C14131	HMDB02209	0.86	0.5374	0.4573	1.1923	1.0208	100	100	Xenobiotics	Food component/Plant	382975	94105-90-5;	4502	241.1
1732		Food component/Plant	N-glycolylneuraminate	LC/MS neg	37123	C03410	HMDB00833	1.75	0.0774	0.1368	0.8648	1.5144	100	100	Xenobiotics	Food component/Plant	4603312	1113-83-3;	653	324
1736			syringic acid	LC/MS neg	37091	C10833	HMDB02085	1.09	0.8211	0.5696	0.9925	1.0782	89	82	Xenobiotics	Food component/Plant	10742	530-57-4;	1416	197
1753			stachydrine	LC/MS pos	34384	C10172	HMDB04827	1.46	0.0985	0.1567	0.8835	1.2937	100	100	Xenobiotics	Food component/Plant	115244	4136-37-2;	860	144.1
1754			homostachydrine*	LC/MS pos	33009	C08283		1.63	0.2209	0.2737	0.9423	1.5317	100	100	Xenobiotics	Food component/Plant	441447	1195-94-4;	1199	158.2
1759			sitostanol	GC/MS	37524	C19644		0.56	0.0001	0.0008	1.4580	0.8097	100	100	Xenobiotics	Food component/Plant		19466-47-8;	2388	215.2
1760			2-oxindole-3-acetate	LC/MS pos	40479			0.79	0.4677	0.4195	1.4003	1.1036	100	100	Xenobiotics	Food component/Plant	3080590	2971-31-5;	3217.2	192.1
1761.5			fumonisin B1	LC/MS pos	42597			1.56	0.1187	0.1802	1.0099	1.5752	78	91	Xenobiotics	Food component/Plant	3431	116355-83-0;	4502	722.5
1769			vanillin	GC/MS	37442	C00755	HMDB12308	0.91	0.4981	0.4333	1.0912	0.9885	78	45	Xenobiotics	Food component/Plant		121-33-5;	1550	194
1788			1H-quinolin-2-one	LC/MS pos	40464	C06415		0.44	0.0522	0.1062	1.9274	0.8502	100	100	Xenobiotics	Food component/Plant	6038	59-31-4;	3533	146.2
1796.3			Isobar: dihydrocaffeate, 3,4-dihydroxycinnamate	GC/MS	40515			3.80	0.4493	0.4158	0.8380	3.1833	56	55	Xenobiotics	Food component/Plant			1852.8	178.9
1818		Sugar, sugar substitute, starch	erythritol	GC/MS	20699	C00503	HMDB02994	1.33	0.9931	0.6201	0.8111	1.0766	78	73	Xenobiotics	Sugar, sugar substitute, starch		149-32-6;	1517.5	217
1824			galacturonate	GC/MS	20758	C08348	HMDB02545	2.51	0.0079	0.0287	0.7942	1.9930	100	100	Xenobiotics	Sugar, sugar substitute, starch	84740	91510-62-2;	1890	204
